# Using genomics to understand the origin and dispersion of multidrug and extensively drug resistant tuberculosis in Portugal

**DOI:** 10.1038/s41598-020-59558-3

**Published:** 2020-02-13

**Authors:** João Perdigão, Pedro Gomes, Anabela Miranda, Fernando Maltez, Diana Machado, Carla Silva, Jody E. Phelan, Laura Brum, Susana Campino, Isabel Couto, Miguel Viveiros, Taane G. Clark, Isabel Portugal

**Affiliations:** 1 Research Institute for Medicines (iMed.ULisboa), Faculdade de Farmácia, Universidade de Lisboa, Lisboa, Portugal; 2 Departamento de Doenças Infeciosas, Instituto Nacional de Saúde Dr. Ricardo Jorge, Porto, Portugal; 3 Serviço de Doenças Infecciosas, Hospital de Curry Cabral, Lisboa, Portugal; 4 Unidade de Microbiologia Médica, Global Health and Tropical Medicine, GHTM, Instituto de Higiene e Medicina Tropical, IHMT, Universidade Nova de Lisboa, UNL, Lisboa, Portugal; 5 London School of Hygiene & Tropical Medicine, Keppel Street, London, WC1E 7HT United Kingdom; 6 SYNLAB Portugal, Lisboa, Portugal

**Keywords:** Infectious diseases, Microbiology

## Abstract

Portugal is a low incidence country for tuberculosis (TB) disease. Now figuring among TB low incidence countries, it has since the 1990s reported multidrug resistant and extensively drug resistant (XDR) TB cases, driven predominantly by two strain-types: Lisboa3 and Q1. This study describes the largest characterization of the evolutionary trajectory of M/XDR-TB strains in Portugal, spanning a time-period of two decades. By combining whole-genome sequencing and phenotypic susceptibility data for 207 isolates, we report the geospatial patterns of drug resistant TB, particularly the dispersion of Lisboa3 and Q1 clades, which underly 64.2% and 94.0% of all MDR-TB and XDR-TB isolates, respectively. Genomic-based similarity and a phylogenetic analysis revealed multiple clusters (n = 16) reflecting ongoing and uncontrolled recent transmission of M/XDR-TB, predominantly associated with the Lisboa3 and Q1 clades. These clades are now thought to be evolving in a polycentric mode across multiple geographical districts. The inferred evolutionary history is compatible with MDR- and XDR-TB originating in Portugal in the 70’s and 80’s, respectively, but with subsequent multiple emergence events of MDR and XDR-TB particularly involving the Lisboa3 clade. A SNP barcode was defined for Lisboa3 and Q1 and comparison with a phylogeny of global strain-types (n = 28 385) revealed the presence of Lisboa3 and Q1 strains in Europe, South America and Africa. In summary, Portugal displays an unusual and unique epidemiological setting shaped by >40 years of uncontrolled circulation of two main phylogenetic clades, leading to a sympatric evolutionary trajectory towards XDR-TB with the potential for global reach.

## Introduction

Multidrug-resistant (MDR) tuberculosis (TB) poses a serious threat to the WHO goal of eliminating TB by 2035^1^. In Europe alone, the number of MDR-TB notified cases amounted to 35 224 cases in 2017^2^. MDR-TB treatment involves using second-line drugs which are more expensive, have longer treatment regimens and currently have lower success rates^1^. Extensively Drug Resistant (XDR) TB, which is MDR-TB with concomitant resistance to a fluoroquinolone and an injectable second-line drug, is associated with a much worse outcome^1^.

Portugal recently became a low TB incidence country by reporting a notification rate below 20 cases per 100 000 habitants (17.5/100 000 in 2017)^2^. Nonetheless, it still reports the highest incidence rate in Western Europe and while this rate shows a steady decline averaged at −7.9% per year, treatment success rates are decreasing. Additionally, according to the most recent data from the WHO, it is estimated that Portugal had 24 new incident cases per 100 000 habitants in 2018^1^. Ten MDR-TB cases were reported in 2017, but this is thought to be an underestimate, as only 61.4% of all TB cases are laboratory-confirmed, and only 66.7% of those underwent drug susceptibility testing (DST)^2^. During the 1990’s, a group of genetically similar MDR and XDR-TB strains circulating in Lisbon were reported, and were characterised using molecular methods into Lisboa3 (SIT20/LAM1) and Q1 clades (SIT1106/Q1)^3,4^. Both clades bear the RD^RIO^ deletion while comprising distinctive monophyletic clades^5,6^. These two strains are still uncontained and responsible for an important fraction of primary MDR-TB in Lisbon^7,8^. However, the extent by which these strains have disseminated throughout Portugal is unknown. Similarly, the clustering dynamics and range of dispersion of other strains that emerge transiently (e.g. Beijing) is also poorly understood due to the historical reliance on classical typing methods, which have limited discriminatory ability compared to whole genome sequencing (WGS)^5,9,10^.

WGS of clinical *Mycobacterium tuberculosis* strains has provided insights on the mode of transmission and dissemination, and a deeper understanding of the genetic determinants of drug resistance^11–14^. The most common mutations in drug resistance associated genes that underpin poly-drug resistance in Lisbon have been reported^3,7^. These mutations include double *inhA* mutations rather than *katG* mutations concerning isoniazid (INH) resistance or, *eis* promoter mutations and a frameshift mutation caused by a binucleotidic insertion on *tlyA* regarding kanamycin (KAN) and capreomycin (CAP) resistance, respectively^15,16^. However, to date there has been no large-scale study involving resistant strains sourced across Portugal. In the present study, we address this gap by analysing the largest WGS dataset on *M. tuberculosis* generated across the country. By investigating the genomic similarity within a phylogenetic framework, we attempt to understand strain diversity, its origin and evolution and, the potential for the established Lisboa3 and Q1 strains to spread outside of the country.

## Methods

### Clinical isolates

A total of 207 *M. tuberculosis* clinical isolates, sourced nationwide from 15 districts in Portugal, including the Madeira and Azores archipelagos, were selected for the present study. The study sample is composed of 191 isolates retrospectively selected between 2008–2016 as part of the TB National Laboratory Surveillance-VigLab program, across the National Institute for Health, Institute for Hygiene and Tropical Medicine of the NOVA University of Lisbon and, Faculty of Pharmacy of the University of Lisbon. This sample includes 71.1% of the MDR-TB cases notified by national public health authorities in the same period and comprises an excess of 31 XDR-TB isolates to those notified in the same period^17^. An additional 16 historical strains isolated between 1995–2007 were also included. All but 19 clinical isolates were resistant to at least one first-line anti-TB drug (Supplementary Table S1). Isolate collection was carried out irrespective of patient nationality or country of birth. All procedures involving the manipulation of *M. tuberculosis* strains were carried out under strict level 3 biosafety facilities within the above-mentioned institutions.

DST against first-line drugs was performed for all isolates using the standardized procedure of fluorometric BACTEC MGIT 960 system (Becton Dickinson Diagnostic Systems, Sparks, MD, USA) for INH, rifampicin (RIF), streptomycin (STR), ethambutol (EMB) and pyrazinamide (PZA). For strains isolated before 2000, susceptibility to PZA was not determined.

All isolates resistant to INH and RIF were classed as MDR-TB and, when available, subjected to second-line drug susceptibility testing using the same methodology applied to FQ (ciprofloxacin [CIP] and ofloxacin [OFX]) and injectable second-line drugs (amikacin [AMK], kanamycin [KAN] and capreomycin [CAP]). MDR-TB isolates displaying concomitant resistance to at least one FQ and one second-line injectable drug were instead classified as XDR-TB. All methods were performed in accordance with the relevant guidelines and regulations^18,19^.

### Whole genome sequencing and variant calling

Genomic DNA extraction was carried out from cultures on Lowenstein-Jensen slopes using the cetyl trimethylammonium bromide methodology^20^. All DNA samples were subjected to WGS using an Illumina HiSeq2500/4000 (Illumina) sequencing platform producing paired-end 100/150 bp reads. Raw sequence data obtained was trimmed and filtered using Trimmomatic and mapped to the reference genome of *M. tuberculosis* H37Rv (GenBank Accession NC000962.3) using the BWA-MEM algorithm^21^. Average sequencing depth (fold coverage) and reference coverage were 294.2 and 99.0%, respectively. SNPs and small indels were called using SAMtools/BCFtools and GATK as previously described^22,23^. Both variant sets obtained by each variant call method were combined and only the concordant set between both variant callers was retained for downstream analysis. SNP sites or samples having an excess of 10% missing calls were removed from the analysis^12^. SNP sites within PE/PPE genes were also removed. SNPs occurring in drug resistance associated genes were retained for increased resolution at the microevolutionary level. The final genome-wide SNP dataset was composed of 207 isolates and 26 767 high-quality SNP sites. Of those SNPs, 21 444 (80.1%) had no missing genotypes and SNPs occurring in drug resistance associated loci did not exceed 1% of the total SNP sites.

### Phylogenetic and dating analysis

The phylogenetic analysis was based on the final set of the segregating SNP sites. The best-fit nucleotide substitution model was selected using the modelTest function implemented in R phangorn package and evaluated under the Akaike Information Criterion^24^. The Generalised Time-Reversible (GTR) model without rate variation between sites or invariant sites was selected under a negative log-Likelihood of 148 753. A maximum-likelihood phylogenetic tree was constructed using PhyML, implemented in SeaView, using the approximate likelihood ratio test as a branch support metric^25^. Tree annotation was carried out using the Interactive Tree of Life online tool^26^.

BEAST was used to estimate the divergence dates across the Lisboa3 and Q1 clades. Several initial runs of 50 million iterations were carried out for different permutations of tree priors and clock models and compared by Marginal Likelihood Estimation (MLE) using both stepping-stone and path sampling methods. In both clades, a constant model under an uncorrelated relaxed lognormal molecular clock was found to best fit the data upon comparison by Bayes Factor estimation. Using the latter tree prior and molecular clock along with a GTR substitution model, BEAST was run for 50 million iterations with sampling at each 1000 iterations and a burn-in of 5 million (10%) iterations. The BEAST XML input file was generated using BEAUti. Tracer was used to evaluate the convergence of multiple runs as well as the Effective Sample Sizes (ESS). All statistics showed a value above the minimum ESS threshold herein considered (ESS = 100) with the analysis for Lisboa3 and Q1 clades always showing ESSs above 430 and 2300, respectively. Convergence was observed for all statistics over three independent runs under the same priors in each clade. LogCombiner was used to merge log and tree files from independent runs and, TreeAnnotator was used to generate a time-scaled maximum clade credibility tree.

### Strain-typing

Spoligotyping was carried out as initially described by Kamerbeek *et al*. (1997) for a subset of 106 isolates^27^. Briefly, oligonucleotide pair DRa (5′-Biotin-GGTTTTGGGTCTGACGAC-3′) and DRb (5′-CCGAGAGGGGACGAAAC-3′) was used to amplify all 43 target spacer regions of the Direct repeat (DR) locus on a single-tube multiplex PCR reaction from 20 ng of genomic DNA using NZYTaq II (NZYTech). Detection of spacer regions was carried out by reverse hybridization on an in-house produced membrane containing amino-linked immobilized probes. All spoligotyping profiles obtained were confirmed and carried out in silico for the remaining isolates using SpolPred and SpoTyping^28,29^. Spoligotyping profiles were assigned to lineage, clade and shared international type (SIT) using the rules described in SITVITWEB and SITVIT2 international databases^30–32^. Isolate sub-lineages were determined from whole genome sequencing using an established barcode^33^.

### Identification of clade-specific Barcode SNPs

A clade-specific barcode for Lisboa3 and Q1 strains was identified using two independent approaches based on ancestral sequence reconstruction and population genetics fixation index (F_ST_) metric^33^. Ancestral reconstruction at every node in the phylogenetic tree was accomplished using a Maximum Parsimony method through the ancestral.pars function in phangorn R package. Potential barcode SNPs were those found to segregate between the most recent common ancestor (MRCA) of the clade of interest, along with its closest outgroup isolate, and the MRCA of the clade of interest. For the F_ST_ approach, the entire dataset was divided into two populations, i.e, one including those descending from the MRCA of the clade of interest and, a second, encompassing all other isolates. The F_ST_ metric was calculated for each SNP as a measure of its ability to differentiate between populations. Candidate barcode SNPs were those found to have a F_ST_ value of 1. These candidate barcode SNPs were further refined by favouring SNPs that: i) are intragenic to essential genes thereby decreasing the probability of the site being lost due to genomic deletions; and ii), result in synonymous substitutions, which tend to be evolutionary neutral and therefore not under strong selective pressure.

Each barcode SNP was further validated by examining its distribution through different *M. tuberculosis* sublineages, as defined by Coll *et al*. (2014), in a global dataset composed of 28 385 isolates with publicly available sequence data^33^. The latter dataset is available at the iMed.ULisboa Laboratory of Molecular Mycobacteriology and is composed by isolates with publicly available raw sequence data at the European Nucleotide Archive (ENA), sequenced by Illumina sequencing platforms under a paired-end sequencing mode and, available until July 31^st^, 2018. (Supplementary Table S2). Variants were obtained by mapping raw sequence data downloaded from the public domain against *M. tuberculosis* H37Rv (NC000962.3) using the Snippy mapping and variant calling pipeline (https://github.com/tseemann/snippy).

### Statistical analyses

All statistical analyses were carried out using R statistical software. Odds ratios (OR) and confidence intervals were calculated for each mutation or genotype and its association to drug resistance.

## Results

### Drug resistant *M. tuberculosis* population structure in Portugal

The study includes a total of 207 *M. tuberculosis* clinical isolates: 191 were prospectively collected from 2008 to 2016 and 16 isolates selected retrospectively as to specifically form a sample enriched with drug resistant isolates. Of the 207 isolates, 19 (9.2%) were pan-susceptible isolates, 151 (72.9%) MDR-TB, of which 132 were subjected to routine second-line DST to fluoroquinolones and at least two second-line injectable drugs (SLIDs), and of these 49 (37.1%) were further classed as XDR-TB (Table 1). Seventy-four (49%) MDR-TB isolates displayed resistance to all five first-line drugs (Supplementary Table S1). Moreover, 37 isolates exhibited drug resistance to at least one first-line drug and showed resistance profiles other than MDR-TB. All isolates were obtained from different laboratories and hospital units nationwide from a total of 15 districts, including 143 (48%; 100 MDR/XDR-TB) from Lisbon, and 18 (8.7%; 16 MDR-TB) from Porto. XDR-TB isolates originated from multiple districts nationwide but, when considering MDR-TB isolates with SLD DST available, were particularly prevalent in Lisbon (41.2%, n = 35/85), Ponta Delgada (100%, n = 5/5) and Setubal (37.5%, n = 3/8) districts, both part of the Lisbon Health Region.

**Table 1:** Summary frequencies and distribution of isolates per district of origin, lineage and sub-lineage and spoligotyping clade.

	N (%)	Clade (%)			Drug Resistance (%)				
		Lisboa3	Q1	Other	Susceptible	Other	MDR	MDR with Second-line DST Available	XDR
**Origin (District)**									
Aveiro	1 (0.5)	0 (0)	0 (0)	1 (0.5)	0 (0)	0 (0)	1 (0.5)	1 (0.5)	0 (0)
Braga	5 (2.4)	2 (1)	0 (0)	3 (1.4)	1 (0.5)	0 (0)	4 (1.9)	4 (1.9)	0 (0)
Braganca	1 (0.5)	0 (0)	0 (0)	1 (0.5)	0 (0)	0 (0)	1 (0.5)	1 (0.5)	0 (0)
Coimbra	2 (1)	1 (0.5)	1 (0.5)	0 (0)	0 (0)	0 (0)	0 (0)	2 (1)	2 (100)
Evora	1 (0.5)	0 (0)	0 (0)	1 (0.5)	0 (0)	0 (0)	1 (0.5)	0 (0)	0 (0)
Faro	1 (0.5)	0 (0)	0 (0)	1 (0.5)	0 (0)	0 (0)	1 (0.5)	1 (0.5)	0 (0)
Funchal	1 (0.5)	1 (0.5)	0 (0)	0 (0)	0 (0)	0 (0)	1 (0.5)	1 (0.5)	0 (0)
Lisboa	143 (69.1)	40 (19.3)	32 (15.5)	71 (34.3)	17 (8.2)	26 (12.6)	65 (31.4)	85 (41.1)	35 (41.2)
Ponta Delgada	5 (2.4)	4 (1.9)	0 (0)	1 (0.5)	0 (0)	0 (0)	0 (0)	5 (2.4)	5 (100)
Porto	18 (8.7)	4 (1.9)	0 (0)	14 (6.8)	0 (0)	2 (1)	14 (6.8)	16 (7.7)	2 (12.5)
Santarem	4 (1.9)	2 (1)	0 (0)	2 (1)	0 (0)	1 (0.5)	3 (1.4)	3 (1.4)	0 (0)
Setubal	19 (9.2)	13 (6.3)	2 (1)	4 (1.9)	1 (0.5)	8 (3.9)	7 (3.4)	8 (3.9)	3 (37.5)
Viana do castelo	2 (1)	2 (1)	0 (0)	0 (0)	0 (0)	0 (0)	2 (1)	1 (0.5)	0 (0)
Vila Real	1 (0.5)	0 (0)	0 (0)	1 (0.5)	0 (0)	0 (0)	1 (0.5)	1 (0.5)	0 (0)
Viseu	3 (1.4)	3 (1.4)	0 (0)	0 (0)	0 (0)	0 (0)	1 (0.5)	3 (1.4)	2 (66.7)
**Total**	207 (100)	72 (34.8)	35 (16.9)	100 (48.3)	19 (9.2)	37 (17.9)	102 (49.3)	132 (63.8)	49 (37.1)
**Lineage/Sub-lineage**									
2.2.1	19 (9.2)	0 (0)	0 (0)	19 (9.2)	1 (0.5)	2 (1)	15 (7.2)	12 (5.8)	1 (8.3)
3	2 (1)	0 (0)	0 (0)	2 (1)	0 (0)	1 (0.5)	1 (0.5)	1 (0.5)	0 (0)
4.1	2 (1)	0 (0)	0 (0)	2 (1)	1 (0.5)	0 (0)	1 (0.5)	1 (0.5)	0 (0)
4.1.1.1	4 (1.9)	0 (0)	0 (0)	4 (1.9)	2 (1)	1 (0.5)	1 (0.5)	1 (0.5)	0 (0)
4.1.1.3	6 (2.9)	0 (0)	0 (0)	6 (2.9)	1 (0.5)	0 (0)	4 (1.9)	4 (1.9)	1 (25)
4.1.2.1	6 (2.9)	0 (0)	0 (0)	6 (2.9)	0 (0)	0 (0)	6 (2.9)	5 (2.4)	0 (0)
4.2.1	3 (1.4)	0 (0)	0 (0)	3 (1.4)	0 (0)	0 (0)	3 (1.4)	3 (1.4)	0 (0)
4.2.2.1	1 (0.5)	0 (0)	0 (0)	1 (0.5)	0 (0)	0 (0)	1 (0.5)	1 (0.5)	0 (0)
4.3.2	5 (2.4)	0 (0)	0 (0)	5 (2.4)	2 (1)	3 (1.4)	0 (0)	0 (0)	0 (0)
4.3.3	5 (2.4)	0 (0)	0 (0)	5 (2.4)	1 (0.5)	3 (1.4)	1 (0.5)	1 (0.5)	0 (0)
4.3.4.1	20 (9.7)	0 (0)	0 (0)	20 (9.7)	5 (2.4)	8 (3.9)	6 (2.9)	7 (3.4)	1 (14.3)
4.3.4.2	118 (57)	72 (34.8)	35 (16.9)	11 (5.3)	3 (1.4)	15 (7.2)	54 (26.1)	88 (42.5)	46 (52.3)
4.3.4.2.1	3 (1.4)	0 (0)	0 (0)	3 (1.4)	0 (0)	1 (0.5)	2 (1)	2 (1)	0 (0)
4.4.1.1	3 (1.4)	0 (0)	0 (0)	3 (1.4)	1 (0.5)	1 (0.5)	1 (0.5)	1 (0.5)	0 (0)
4.6.1.2	1 (0.5)	0 (0)	0 (0)	1 (0.5)	0 (0)	0 (0)	1 (0.5)	1 (0.5)	0 (0)
4.7	1 (0.5)	0 (0)	0 (0)	1 (0.5)	0 (0)	0 (0)	1 (0.5)	1 (0.5)	0 (0)
4.8	8 (3.9)	0 (0)	0 (0)	8 (3.9)	2 (1)	2 (1)	4 (1.9)	3 (1.4)	0 (0)
**Total**	207 (100)	72 (34.8)	35 (16.9)	100 (48.3)	19 (9.2)	37 (17.9)	102 (49.3)	132 (63.8)	49 (37.1)
**Spoligotyping Clade**									
BEIJING	19 (9.2)	0 (0)	0 (0)	19 (9.2)	1 (0.5)	2 (1)	15 (7.2)	12 (5.8)	1 (8.3)
CAS	1 (0.5)	0 (0)	0 (0)	1 (0.5)	0 (0)	0 (0)	1 (0.5)	1 (0.5)	0 (0)
CAS1-DELHI	1 (0.5)	0 (0)	0 (0)	1 (0.5)	0 (0)	1 (0.5)	0 (0)	0 (0)	0 (0)
H1	5 (2.4)	0 (0)	0 (0)	5 (2.4)	0 (0)	0 (0)	5 (2.4)	5 (2.4)	0 (0)
H3	3 (1.4)	0 (0)	0 (0)	3 (1.4)	0 (0)	0 (0)	3 (1.4)	3 (1.4)	0 (0)
LAM1	86 (41.5)	72 (34.8)	0 (0)	14 (6.8)	3 (1.4)	15 (7.2)	33 (15.9)	61 (29.5)	35 (57.4)
LAM11-ZWE	2 (1)	0 (0)	0 (0)	2 (1)	0 (0)	0 (0)	2 (1)	2 (1)	0 (0)
LAM2	4 (1.9)	0 (0)	0 (0)	4 (1.9)	1 (0.5)	1 (0.5)	2 (1)	2 (1)	0 (0)
LAM3	3 (1.4)	0 (0)	0 (0)	3 (1.4)	0 (0)	3 (1.4)	0 (0)	0 (0)	0 (0)
LAM4	36 (17.4)	0 (0)	35 (16.9)	1 (0.5)	0 (0)	2 (1)	22 (10.6)	29 (14)	12 (41.4)
LAM6	2 (1)	0 (0)	0 (0)	2 (1)	0 (0)	2 (1)	0 (0)	0 (0)	0 (0)
LAM7-TUR	1 (0.5)	0 (0)	0 (0)	1 (0.5)	0 (0)	0 (0)	1 (0.5)	1 (0.5)	0 (0)
LAM9	10 (4.8)	0 (0)	0 (0)	10 (4.8)	3 (1.4)	4 (1.9)	3 (1.4)	3 (1.4)	0 (0)
S	3 (1.4)	0 (0)	0 (0)	3 (1.4)	1 (0.5)	1 (0.5)	1 (0.5)	1 (0.5)	0 (0)
T1	8 (3.9)	0 (0)	0 (0)	8 (3.9)	1 (0.5)	2 (1)	5 (2.4)	4 (1.9)	0 (0)
T2	1 (0.5)	0 (0)	0 (0)	1 (0.5)	0 (0)	0 (0)	1 (0.5)	1 (0.5)	0 (0)
X1	2 (1)	0 (0)	0 (0)	2 (1)	0 (0)	0 (0)	1 (0.5)	1 (0.5)	1 (100)
X2	4 (1.9)	0 (0)	0 (0)	4 (1.9)	2 (1)	1 (0.5)	1 (0.5)	1 (0.5)	0 (0)
X3	4 (1.9)	0 (0)	0 (0)	4 (1.9)	1 (0.5)	0 (0)	3 (1.4)	3 (1.4)	0 (0)
Unclassified	12 (5.8)	0 (0)	0 (0)	12 (5.8)	6 (2.9)	3 (1.4)	3 (1.4)	2 (1)	0 (0)
**Total**	207 (100)	72 (34.8)	35 (16.9)	100 (48.3)	19 (9.2)	37 (17.9)	102 (49.3)	132 (63.8)	49 (37.1)

Data is shown stratified by resistance type and the main clades found in the study. Percentages shown are relative to the total sample size (n = 207) except for the number of XDR isolates for which the percentage is relative to the number of MDR-TB isolates with available second-line DST data.

Using WGS data, the 207 *M. tuberculosis* isolates were assigned to be predominantly lineage 4.3.4.2 strain-types (n = 118; 57.0%), but other lineage 4 (n = 68), 2 (n = 19; Beijing) and 3 (n = 2; Central Asian) were present (Table 1). Using a Spoligotyping-based classification, most strains (n = 144, 69.6%) were assigned to the Latin American and Mediterranean (LAM) lineage as most of the isolates in the study were classified as SIT20/LAM1 (n = 84, 40.6%) or SIT1106/LAM4 (n = 35, 16.9%).

### Multidrug resistance in Portugal is dominated by two phylogenetic clades nationwide

**Figure 1:**
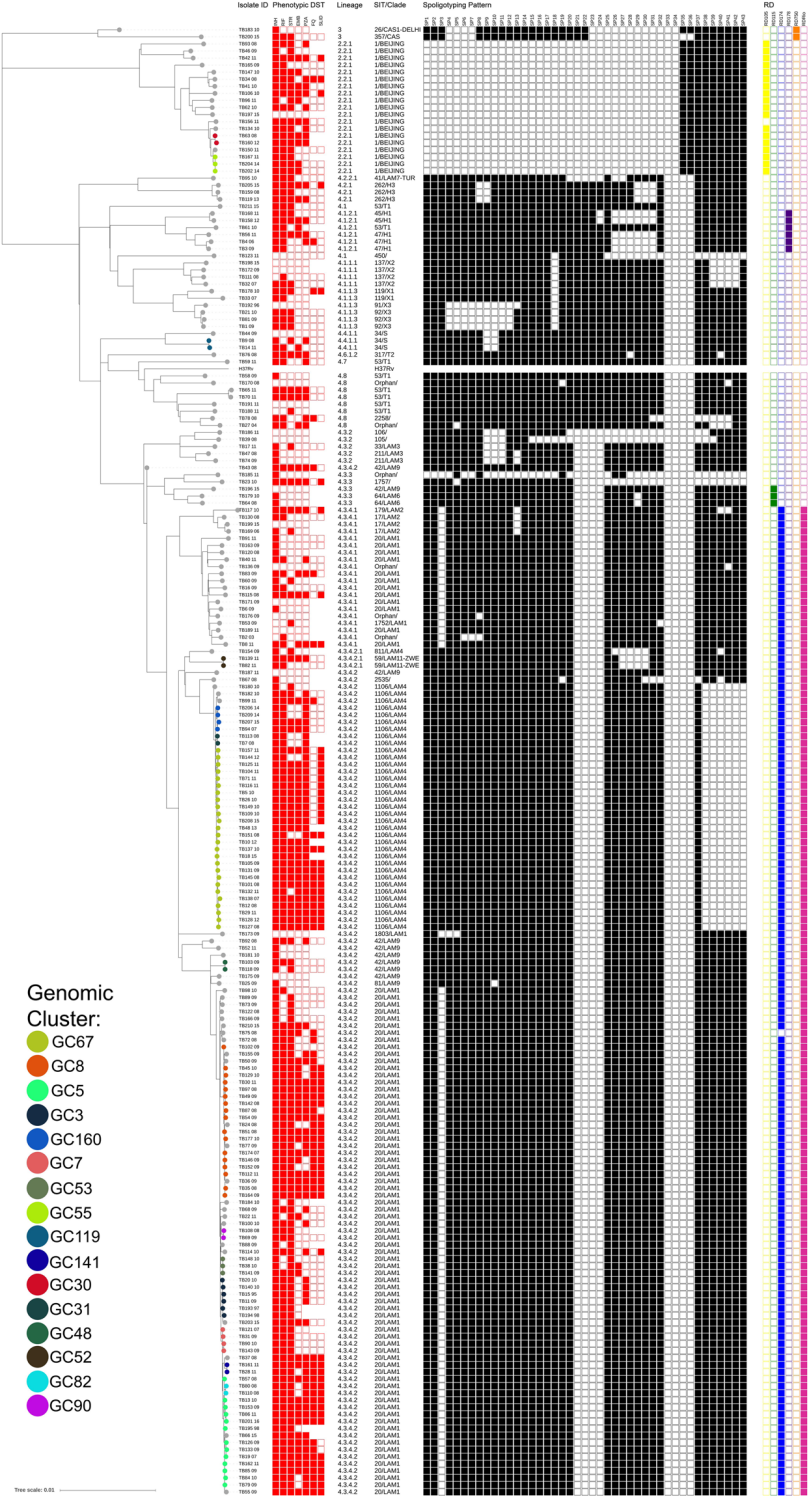
Genome-wide phylogenetic tree of all 207 clinical isolates, and *M. tuberculosis* H37Rv. The tree was based on 26 767 SNPs and rooted on *Mycobacterium cannettii*. The tree is shown annotated with; phenotypic drug susceptibility data for all first-line drugs, SLIDs and FQs (resistance – filled red squares, susceptible – empty squares, not tested – square absence); sub-lineage, SIT and spoligotyping profile; and, presence (filled squares) or absence (empty squares) of specific RDs: RD105, RD174, RD178, RD750 and RD^RIO^. Tips are colored according to genomic clustering (see legend).

A genome-wide phylogenetic tree based on 26 767 high-quality SNPs was reconstructed for all 207 clinical isolates included in the study (Fig. 1). The MDR-TB (n = 151) overall structure is dominated by two main phylogenetic clades in Lisbon: Lisboa3/SIT20/LAM1 (n = 72/151; 47.7%) and Q1/SIT1106/LAM4 strains (n = 35/151; 23.2%). Both clades comprise 64.2% (n = 97/151) and 94.0% (n = 46/49) of all MDR-TB and XDR-TB isolates, respectively, share a recent common ancestry with SIT42/LAM9/RD^RIO^ strains and, are therefore unrelated with other SIT20/LAM1 and LAM4 strains. The phylogenetic scenario denotes convergent evolution of the DR locus which is more notorious among the Lisboa3 clade as it originates an independent SIT20/LAM1 sub-branch. Herein, the closest pan-susceptible isolates to Lisboa3 and Q1 clades, TB175_09 and TB187_11, respectively, both SIT42/LAM9 isolates, show a mean distance of 62.9 SNPs (range: 50–71 SNPs) to the Lisboa3 clade (TB175_09/Lisboa3) and 176.9 SNPs (range: 140–184 SNPs) to the Q1 clade (TB187_11/Q1). Both clades appear to be highly prevalent in Lisbon and its surroundings but are not restricted to this region. In fact, Lisboa3 strains can be found across the entire continental territory and have even been detected in the insular regions of the Azores and Madeira (Supplementary Fig. S1). The distribution of Q1 strains is not as vast as Lisboa3 and is largely confined to the Lisbon Health Region, except for one isolate recovered from the centre region (Coimbra). This observation is consistent with a broader distribution of SNP pairwise distance within the Lisboa3 clade when compared to the Q1 clade which shows a narrower distribution of intra-clade SNP pairwise distances (Supplementary Fig. S2). In addition, this data suggests that both clades have emerged in the Lisbon region, where both are highly prevalent, having then disseminated to other regions in the country.

Other large spoligotyping families such as the Beijing, CAS, S and X showed phylogenetic congruence under the genome-wide phylogenetic scenario by forming deeply rooted monophyletic clades when compared to recent Lisboa3 and Q1 clades of the LAM lineage (Fig. 1). The LAM lineage itself also comprised a monophyletic branch in the tree although it included orphan isolates or SITs unclassified according to the present clade assignment rules^34^. In this regard, SIT106, SIT105, SIT1757 and SIT2535 should, in the present study, be regarded as LAM isolates as these bore the *fbpC*^103^ (Ag85C) synonymous polymorphism that is specific to the LAM lineage^35^. All other LAM strains also bore this LAM biomarker. The population structure is largely congruent with the distribution of phylogenetically-informative Regions of Difference: RD105 was associated with lineage 2 strains (n = 19); RD750 with lineage 3 (n = 2); RD174 and RD^RIO^ co-occurred across all isolates belonging to the 4.3.4 sub-lineage; RD115 was found on a monophyletic sub-branch of sub-lineage 4.3.3; and, RD178 was associated with the 4.1.2.1 sub-lineage isolates present in this study sample.

### Drug resistance in Portugal is driven by unique sets and combinations of drug resistance associated mutations

**Figure 2:**
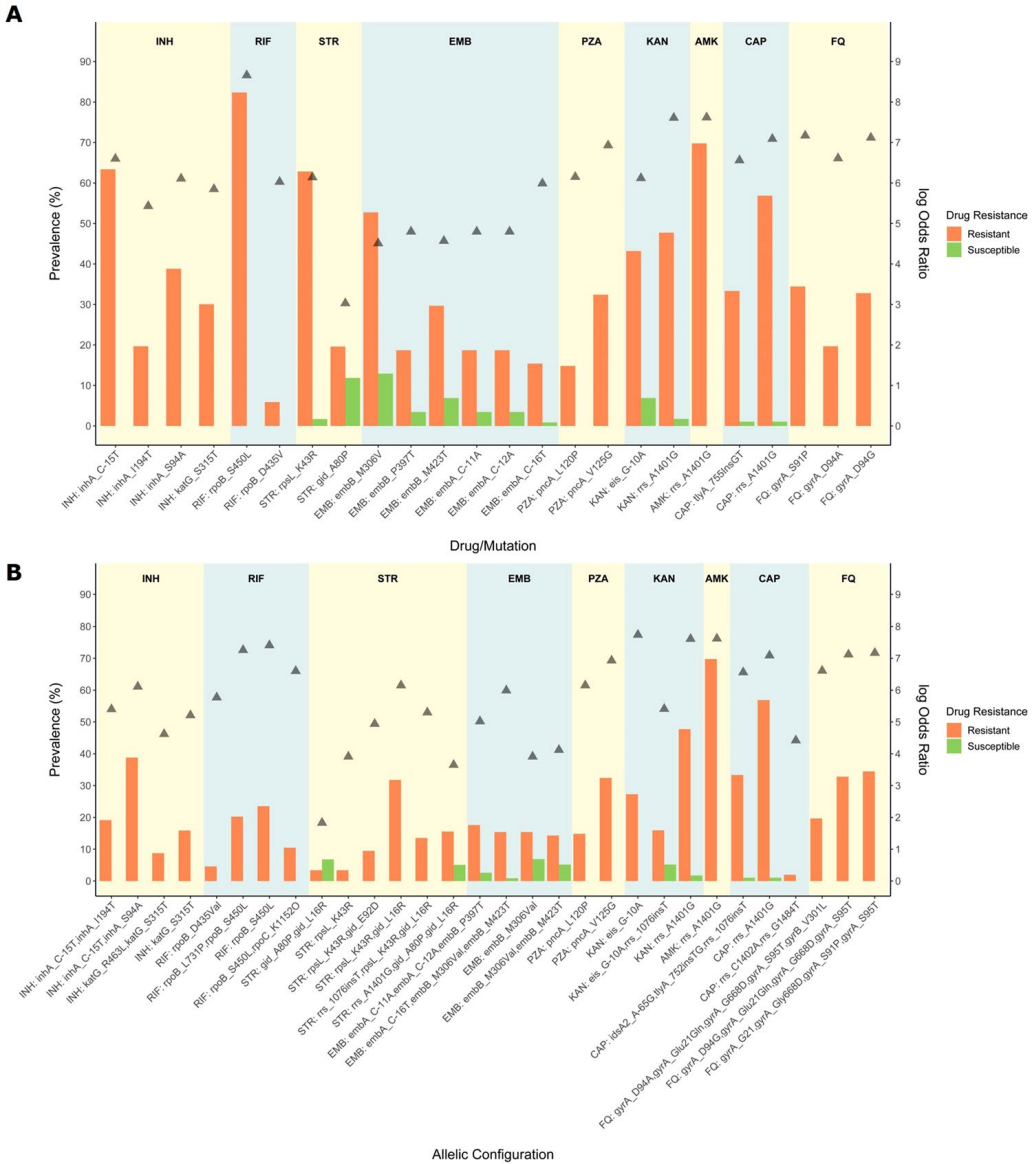
Main mutations (**A**) and respective allelic configurations (**B**) associated with drug resistance. Bars represent the specific relative frequency (left axis) of each mutation among susceptible (green) and resistant (orange) isolates. The positioning of each point (filled triangles) reflects the statistical association of the mutation with drug resistance (log Odds Ratio, right axis).

Based on WGS analysis and regarding INH resistance among 184 INH resistant and 23 susceptible isolates, the C-15T substitution at the *mabA*-*inhA* promoter region was by far the most prevalent mutation since it was detected in 116 isolates, all INH resistant (prevalence in resistant isolates [PR]: 63.0%) (Fig. 2, Supplementary Table S3). *inhA* S94A and I194T mutations, detected among 71 (PR: 38.6%) and 36 (PR: 19.6%) isolates, respectively, were never found as single-mutant genotypes within INH-resistant strains but almost exclusively as S94A/C-15T and I194T/C-15T double-mutant genotypes (Fig. 2, Supplementary Table S4). *katG* S315 T was detected in 55 (PR: 29.9%) isolates, all INH resistant. *inhA*/*katG* double mutants were also detected among five isolates resistant to INH and involved the combination of *inhA* C-15T mutation with either *katG* S315T or T380I, or *inhA* I194T and *katG* S315T (Supplementary Table S4). Three isolates showed nonsynonymous or a promoter mutation in *kasA* and *ahpC*, respectively, which have a questionable role in INH resistance. Also, six (PR: 3.2%) out of 184 phenotypically INH-resistant isolates showed no mutation among the genes classically associated with INH resistance. Upon additional inspection of reference coverage and alternate genes, two out of these six isolates were found to bear one *ndh* missense mutation each (A200G and S206P); one isolate was heterorresistant with a binucleotidic insertion on codon 90 of the *katG* gene; and, one isolate was found to harbor a 28 Kbp deletion encompassing the *katG* gene. All genes associated with INH resistance were deemed wild-type for the remainder two INH-resistant isolates.

As expected, among the 153 phenotypic RIF resistant strains, resistance is mainly driven by mutations occurring in the *rpoB* gene, with the S450L mutation being the most frequent (n = 126; PR: 82.4%) and, with D435V/Y and H445Y/D/L mutations also being present (Fig. 2). Noteworthy, second in frequency to the S450L mutation, the L731P substitution, also occurring at the *rpoB* gene, was detected in 31 (PR: 20.3%) RIF resistant isolates always co-occurring with a S450L substitution (Supplementary Table S4). This observation suggests a role for L731P as a compensatory mutation, which is likely paralleled by with other *rpoB* (V496A/M) or *rpoC* (V483G, K1152Q) mutations observed in at least three or more isolates^36^. Supporting this hypothesis, all the latter mutations occurred solely among RIF resistant isolates and co-occurred with an additional classical RIF resistant mutation (Supplementary Table S4). Two phenotypically resistant isolates showed no RIF resistance associated mutation whereas one phenotypic resistant isolate bore, in single-allelic configuration, a non-canonical mutation causing an in-frame deletion of 9 bp.

For STR resistance, among 148 and 59 phenotypically resistant and susceptible isolates, respectively, a high frequency of *rpsL* K43R mutations were found among 94 isolates, all but one resistant to STR (PR: 62.8%) (Fig. 2, Supplementary Table S3). The K88R mutation was found in four (2.7%) isolates, all resistant to STR. A total of six *rrs* mutations (C492T, C517T, C774A, A807C, A906G and C924T) in the region known to be associated with STR resistance were found across eleven isolates, four of which STR resistant and seven STR susceptible. We observed that: i) *rrs* A807C was concomitantly found with a *rpsL* K43R bearing isolate; ii) *rrs* C774A was found on a STR susceptible isolate; iii) *rrs* C492T and C924T were detected on both susceptible and resistant isolates; and iv) *rrs* C517T and A906G occurred in one STR-resistant isolate each, and were thus more likely to be associated with STR resistance. Aside from the *gidB* A80P mutation, previously described as associated with the Q1 clade and intermediate-level resistance to STR^37^, a total of eight low frequency variants were associated with STR resistance since these were found exclusively among STR-resistant isolates in single allelic combinations, i.e., without concomitant mutations on *rpsL* or *rrs* genes: R21W (n = 1), G34E (n = 2), W45X (n = 1), L79S (n = 1), E92Q (n = 2), V112G (n = 1), G130A (n = 1) and S149R (n = 1).

Among the 91 phenotypically EMB resistant strains, mutations occurring on codon 306 of the *embB* gene were highly prevalent, particularly the M306V mutation which was found in 48 EMB resistant isolates (PR: 52.7%) (Fig. 2, Supplementary Table S3). Mutations M306I and M306L were also detected in eight and one EMB resistant isolate, respectively (9.9%). Two other *embB* mutations were highly frequent among EMB resistant isolates: P397T (n = 17; PR: 18.7%) and M423T (n = 27; PR: 29.7%). *embA* promoter mutations C-16T/G/A in 18 (PR: 19.8%) EMB-resistant isolates and the co-occurrence of C-11A and C-12A in 17 (18.7%) EMB-resistant isolates were also observed. *embB* and *embA* promoter mutations were also detected among EMB susceptible isolates (Supplementary Table S3). The degree of *embB*/A association with resistance was found to depend on the association between individual mutations thereby combining different modes of resistance development^38^: alteration of target site and target overexpression. Regarding the *embB* M306V mutation, besides the 48 EMB-resistant isolates in which this mutation was detected, it was also detected among 15 (7.2%) EMB-susceptible isolates (prevalence among phenotypically susceptible isolates [PS]: 12.9%). Moreover, when in single allelic combination or, e.g., combined with M423T, the M306V mutation was found in 27 (PR: 29.6%) EMB-resistant isolates and 13 (PS: 11.2%) EMB susceptible isolates (Fig. 2, Supplementary Table S4). However, the allelic combination of *embB* M306V, M423T, and *embA* C-16T was detected in 14 (PR: 15.3%) EMB-resistant isolates and only once in an EMB-susceptible isolate (PS: 0.9%). Similarly, the *embB* P397T *embA* C-11A/C-12A allelic configuration was detected in 17 (PR: 18.7%) EMB-resistant isolates and 4 (PS: 3.4%) EMB-susceptible isolates (Supplementary Table S4). None of the latter was observed in a different allelic configuration. *Per se*, *embA* promoter mutations appear to confer some degree of resistance since *embA* C-16A/G single allelic genotypes were detected in two and one EMB-resistant and susceptible isolates, respectively. Although four Rv3806c/*ubiA* mutations were found among four isolates, none of such show a clear association with a resistance phenotype thereby suggesting that Rv3806c/*ubiA* mutations are not a major determinant of EMB resistance in Portugal (Supplementary Table S3). A resistance candidate K114N mutation in Rv2820c was present in Beijing isolates of unrelated origin pointing out to a Lineage 2 phylogenetic marker rather than EMB resistance driven mutation^12^.

Among 108 PZA-resistant and 95 PZA-susceptible isolates, a total of 40 (19.3%) non-synonymous and two promoter mutations were observed in the *pncA* gene, 34 of which exclusively present among phenotypically PZA-resistant isolates, revealing a highly diverse scenario associated with PZA resistance (Supplementary Table S3). Two previously described mutations: L120P (n = 16; PR: 14.8%) and V125G (n = 35; PR: 32.4%) were highly associated with PZA resistance since these were exclusively found among PZA-resistant isolates (Supplementary Table S3). Moreover, all mutations were always found in single allelic configuration and no role for *rpsA* mutations were found in this sample. Also, six PZA-resistant isolates did not bear any mutations on candidate genes associated with PZA resistance (PR: 5.6%). This rate of wild-type PZA-resistant isolates falls within the interval reported in the literature and can also be driven by false-resistance, a widely known and reported problem associated with MGIT-based PZA DST^38,39^.

Resistance to second-line injectable drugs KAN, AMK and CAP appears to be essentially driven by a combination of mutations in three different genes: *rrs* (4 mutations), *eis* (7) and *tlyA* (3). The sample included: 44 isolates phenotypically resistant to KAN and 58 susceptible isolates; 43 AMK-resistant and 104 AMK-susceptible isolates; and, 51 CAP-resistant and 96 CAP-susceptible isolates. Resistance to KAN was mainly associated with mutation A1401G (n = 21; PR: 47.7%) in the *rrs* gene and the promoter mutation G-10A (n = 19, 43.2%) on the *eis* gene (Supplementary Table S3). One additional isolate bearing the *rrs* A1401G mutation and an additional four with the *eis* G-10A mutation were found to be susceptible to KAN. Mutations on the *eis* open reading frame (ORF) did not show a clear association with KAN resistance, similar to a one-bp insertion in *rrs* which was detected in a subset of the isolates bearing the *rrs* G-10A mutation (Supplementary Table S3). The *rrs* A1401G mutation was detected only among 30 AMK-resistant isolates (PR: 69.8%), 29 of which were CAP-resistant. *rrs* C1402A (n = 1, 0.5%) was concomitantly detected with the G1484T mutation in an isolate resistant to KAN and CAP but susceptible to AMK. *rrs* G1484T in single allelic combination was found only among two isolates, all of which resistant to the three drugs (Supplementary Table S4). Resistance to CAP was further associated with a binucleotidic insertion in *tlyA* gene (Ins755GT, n = 18, PR: 33.3%), previously described in Portugal for Lisboa3 strains^7^. All but one isolate that bore this binucleotidic insertion at *tlyA* showed resistance to CAP. Two AMK-resistant isolates did not show any mutation on *rrs* or *eis* gene. The latter is traditionally associated with KAN resistance but not AMK resistance. In this study, nine AMK resistant isolates bore *eis* promoter mutations (G-10A, n = 8, PR: 18.6%; C-14T, n = 1, PR: 2.3%), six of which also carrying the 1-bp insertion at the *rrs* gene, whereas 30 AMK susceptible isolates also bore the same mutation profiles (*eis* G-10A, n = 28, PS: 26.9%; C-14T, n = 2, PS: 1.9%), 15 of which also carrying the *rrs* 1-bp insertion (Supplementary Table S3).

A total of 61 (29.5%) FQ-resistant isolates were included in study, along with 86 (41.5%) susceptible isolates. A clear association between FQ resistance and *gyrA* mutations S91P (n = 21, 34.4%), D94N (n = 1, 16.4%), D94G (n = 20, 32.8%) and D94A (n = 12, 19.7%) was observed (Supplementary Table S4). A *gyrB* D461H mutation was detected exclusively on two resistant isolates without any additional mutations associated with FQ resistance. Two FQ-resistant isolates showed no mutation on *gyrA* or *gyrB* other than the *gyrA* S95T or the *gyrA* G668D which are neutral polymorphisms that are not associated with FQ resistance^40,41^.

### Pinpointing genomic transmission clusters in Portugal

We sought to use the phylogenetic tree to identify clusters that reflect recent transmission. Having confirmed monophyletic nature of the Lisboa3 and Q1 strains, the long-term uncontained circulation of both clades is anticipated to produce local clusters of transmission. Using a conservative 5 SNP genome-wide cut-off distance between isolates^42^, we identified genomic clusters composed of clinical isolates recovered from patients that under this same cut-off are expected to be epidemiologically linked. Sixteen different clusters encompassing a total of 92 isolates were identified (Table 2). This approach yielded a clustering rate of 44.4% which increases to 58.3% when only MDR/XDR isolates are considered. Ten out of the 16 clusters identified are limited to one geographical district and 80 out of the 92 clustered isolates (87.0%) originated within the Lisbon Health Region (comprehending Lisbon, Setubal and Santarem districts - see geographical district column in Table 2). Four clusters encompassed non-MDR isolates (Supplementary Table S1), but these were always clustered with one additional MDR isolate suggesting *de novo* emergence of MDR from pre-MDR cases. This putative *de novo* emergence of MDR-TB was not, however, corroborated by the genotypic analysis where in all cases discordance was related to RIF resistance (Table 2). In the GC53 MDR-TB isolate (TB141_09) coverage analysis revealed one minority allele occurring at codon 9 (T9P), whose role in RIF resistance is unknown; the GC119 isolate phenotypically susceptible to RIF (TB9_08) showed in fact a *rpoB* H445N mutation; and, the GC48 discordant isolate (TB103_09) was either a false-RIF resistant or has an unidentified resistance mechanism. Despite this, the clustering rates obtained reiterate the notion conveyed by the overall phylogenetic structure in which MDR and XDR is being mostly driven by primary MDR/XDR: 49 (68.1%) and 32 (91.4%) of all isolates belonging to the Lisboa3 and Q1 clades were clustered, respectively. Whereas four clusters were found not to involve Lisboa3 and Q1 strains (Beijing [n = 2 clusters], S [1], LAM11-ZWE [1] and LAM9 [1]), eight and three clusters contained Lisboa3 and Q1 isolates, respectively (Table 2). The largest genomic cluster was composed of 26 Q1 isolates, all but two originating in Lisbon. Two additional large genomic clusters encompassed 17 and 13 Lisboa3 isolates from different districts nationwide, including, for example, Coimbra, Porto and Ponta Delgada (Azores). For the five isolates recovered in the Ponta Delgada (Azores), three are clustered with an additional nine isolates from Lisbon and one from Porto (GC5) which suggests potential dissemination from Lisbon to the Azores and Porto. Along with GC90, which is composed of two isolates identified in Viana do Castelo towards Portugal northern border, this implies that: i) transmission of these clusters is also taking place outside Lisbon Health Region, the epicentre of the original described outbreak^4,43^; ii) that these clusters have successfully spread to other regions in Portugal; and iii), the evolution of both Lisboa3 and Q1 is now occurring in a polycentric mode.

**Table 2:** Genomic clusters detected, number of isolates per cluster and geographic distribution per district and drug resistance class.

Genomic Cluster	No. of Isolates	Clade	Lineage^a^	District (No. of Isolates)	Drug Resistance (No. of Isolates)	
					Phenotypic	Genotypic^b^
GC67	26	Q1/SIT1106/LAM4	4.3.4.2	Setúbal (1), Coimbra (1), Lisboa (24)	MDR (14), XDR (12)	MDR (11), XDR (15)
GC8	17	Lisboa3/SIT20/LAM1	4.3.4.2	Porto (2), Lisboa (11), Setúbal (3), Coimbra (1)	MDR (2), XDR (15)	MDR (1), XDR (16)
GC5	13	Lisboa3/SIT20/LAM1	4.3.4.2	Lisboa (9), Porto (1), Ponta Delgada (3)	MDR (3), XDR (10)	XDR (13)
GC3	6	Lisboa3/SIT20/LAM1	4.3.4.2	Setúbal (4), Lisboa (2)	MDR (6)	MDR (6)
GC160	4	Q1/SIT1106/LAM4	4.3.4.2	Lisboa (4)	MDR (4)	MDR (4)
GC7	4	Lisboa3/SIT20/LAM1	4.3.4.2	Lisboa (3), Setúbal (1)	MDR (4)	MDR (4)
GC53	3	Lisboa3/SIT20/LAM1	4.3.4.2	Santarém (2), Lisboa (1)	MDR (1), Other (2)	Other (3)
GC55	3	SIT1/Beijing	2.2.1	Lisboa (3)	MDR (3)	MDR (3)
GC119	2	SIT34/S	4.4.1.1	Lisboa (2)	MDR (1), Other (1)	MDR (2)
GC141	2	Lisboa3/SIT20/LAM1	4.3.4.2	Viseu (2)	XDR (2)	XDR (2)
GC30	2	SIT1/Beijing	2.2.1	Lisboa (2)	MDR (2)	MDR (2)
GC31	2	Q1/SIT1106/LAM4	4.3.4.2	Lisboa (2)	MDR (2)	MDR (2)
GC48	2	SIT42/LAM9	4.3.4.2	Lisboa (2)	MDR (1), Other (1)	Other (2)
GC52	2	SIT59/LAM11-ZWE	4.3.4.2.1	Lisboa (2)	MDR (2)	Other (2)
GC82	2	Lisboa3/SIT20/LAM1	4.3.4.2	Lisboa (2)	XDR (2)	XDR (2)
GC90	2	Lisboa3/SIT20/LAM1	4.3.4.2	Viana do Castelo (2)	MDR (2)	MDR (2)

Distribution per drug resistance class is stratified according to a phenotypic-based classification and genotypic-based classification. Clade and lineage associated with clustered isolates are also shown.

^a^Lineage classification according to SNP Barcode proposed by Coll *et al*.^33^;

^b^Genotypic-based classification was carried out according to mutational analysis and upon comparison with the TB Profiler database as in Coll *et al*.^14^.

Thirteen out of the 16 clusters were found to form monophyletic sub-clades in the phylogenetic tree while three clusters formed paraphyletic groups as these did not include all isolates descending from their most recent common ancestor (MRCA) (Fig. 1). In these circumstances, isolates not included in the nearest genomic clusters were distanced from isolates included in such by an average of 7.4 SNPs, which ranged between 6–15 SNPs although 88.8% did not surpass 8 SNPs of pairwise distance.

### Lisboa3 and Q1 microevolutionary trajectory strains highlight multiple and independent evolutionary trajectories towards MDR/XDR

**Figure 3:**
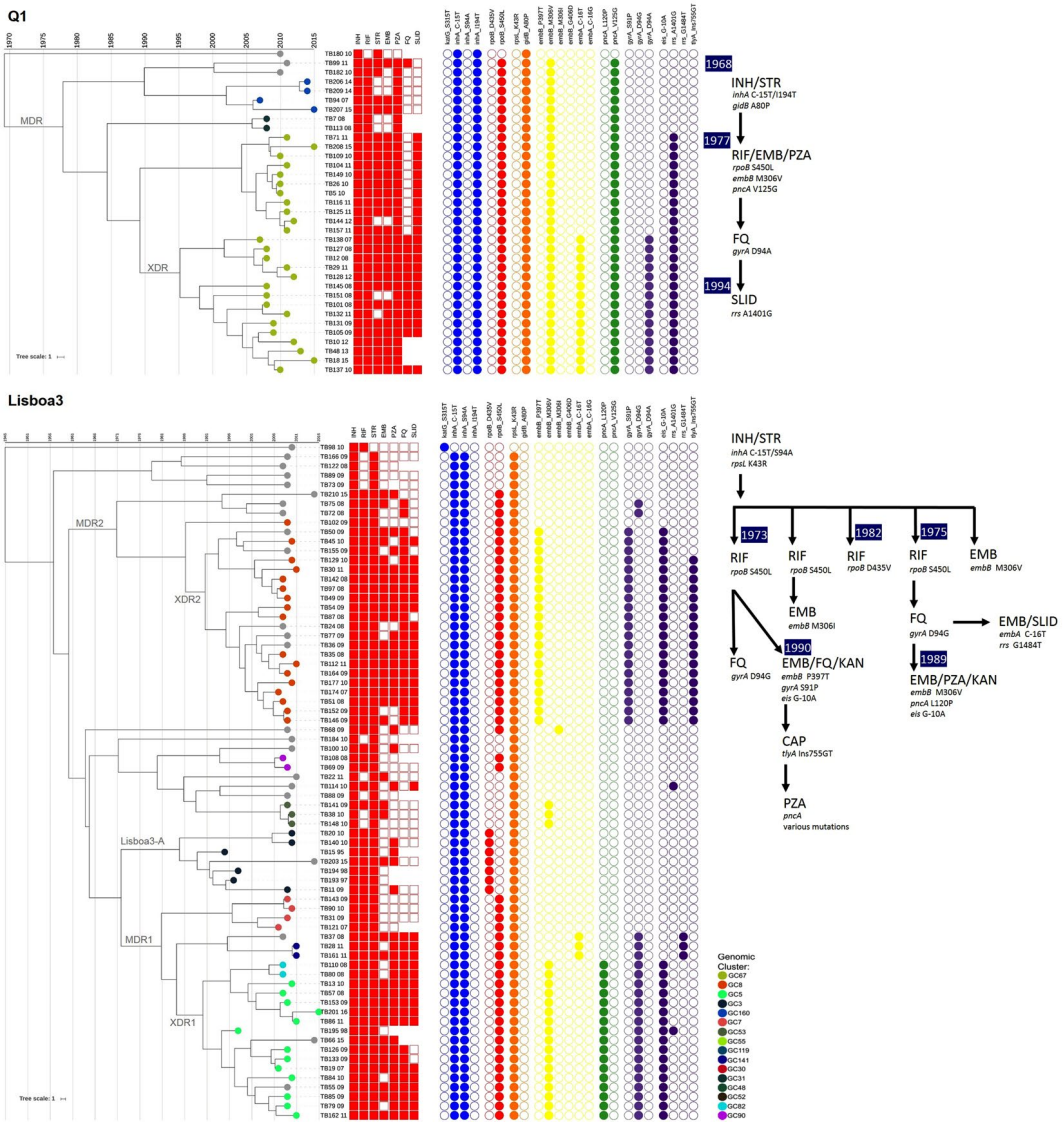
Time-scaled maximum clade credibility trees for the Lisboa3 and Q1 clades highlighting the microevolutionary trajectories in both clades. The tree is shown annotated with phenotypic drug susceptibility testing data along with presence/absence of the most relevant mutations associated with drug resistance. Tips are coloured according to genomic clustering (see legend) and, specific MDR and XDR branches are annotated for easy cross-reference with Table 3 and a resistance acquisition dynamic scheme is shown next to each tree.

The data already conveyed above supports the notion that Lisboa3 and Q1 *M. tuberculosis* strains have initially radiated from Lisbon while attaining high evolutionary success when compared to other well-known and widely dispersed isolates such as Beijing strains. Part of this epidemiological success is thought to derive from its association with MDR/XDR. The present scenario therefore poses as an unparalleled one in a setting where two monophyletic clades show a sympatric microevolutionary trajectory towards XDR. This microevolutionary trajectory towards XDR in the Q1 clade is, as demonstrated by the topological structure of its specific sub-branch, completely linear since resistance has been acquired successively (Fig. 3). The Q1/SIT1106 branch has likely started out with a common ancestor resistant to INH and STR driven by a *inhA* double mutation on its promoter region and on its ORF (C-15T and I194T) and a *gidB* missense mutation (A80P), which has been previously associated with this clade and the cause of intermediate-level resistance to STR^37^. Next, MDR emerged due to the classic S450L mutation on *rpoB* and, at this point also a mutation at *embB*306 codon (M306V) and the V125G at the *pncA* gene also leading to EMB and PZA resistance, respectively. The latter three mutations are shown to co-occur among all isolates of this clade, and it is therefore impossible to ascertain which resistance has emerged in the first place. Still in the Q1 clade, the next step leading to the emergence and split of a pre-XDR sub-branch is marked by the A1401G mutation on the *rrs* gene which is shown to occur in all cluster GC26 isolates. A subset of cluster GC67 split as XDR-TB strains driven by the D94A mutation on *gyrA*, while simultaneously acquiring an *embA* promoter mutation (C-16T) potentially leading to a higher EMB resistance level.

Contrarily to Q1, the Lisboa3 clade shows a more complex resistance acquisition dynamic, where MDR has emerged independently on at least 4 separate occasions and XDR emerged within these MDR sub-branches on three independent points in time (Fig. 3). The initial resistance acquisition pathway for this clade parallels with the Q1 in the sense that it is initially driven by resistance to INH and STR, with the INH phenotypic resistance being also due to a *inhA* double mutation on the promoter region and ORF (C-15T and S94A). STR resistance has been acquired by a different mechanism owing to the modification of the target site due to a K43R mutation on *rpsL*, which is associated with STR high-level resistance. Although some isolates resistant to INH and STR can still be detected, a sub-branch acquired EMB resistance hypothetically through an *embB* M306V mutation, it is hypothesized that most Lisboa3 isolates descend from three sub-branches that have split from the original INH^R^/STR^R^ isolates through the acquisition of RIF resistance either by a *rpoB* S450L mutation or a D435V mutation. A fourth minor and previously undetected sub-branch was herein identified in this study, corresponding to GC90 that has been detected in Viana do Castelo. The *rpoB* D435V sub-branch has been previously associated with the MIRU-VNTR cluster Lisboa3-A in Portugal and no additional detectable drug resistance was acquired by this specific branch^5^. For the latter, all but one isolate belonged to GC3 with a diverging and more recent isolate distancing six SNPS from its nearest isolate (TB193_97), and therefore marginally above the threshold of five SNPs.

On the two main S450L Lisboa3 sub-branches XDR resistance emerged in one of these sub-branches by acquisition of resistance to fluoroquinolones by a *gyrA* S91P mutation and by KAN resistance due a hypermorphic mutation in eis (G-10A). This same sub-branch putatively acquired EMB resistance through the *embB* P397T mutation and amplified its resistance spectrum by acquiring CAP resistance mediated by a frameshift mutation at *tlyA* (Ins755GT). The remaining S450L sub-branch evolved XDR twice through an initial acquisition of *gyrA* D94G yielding fluoroquinolone resistance and lastly, through the same eis hypermorphic mutation in (G-10A) or through the *rrs* G1484T mutation both conferring resistance to SLIDs. The two latter mutations cooccurred with an *embB* M306V and *embA* C-16T mutations, respectively, conveying the notion that EMB resistance followed the acquisition of FQ resistance and was therefore likely acquired during the final trajectory to XDR development (Fig. 3).

### A temporal structure for the Lisboa3 and Q1 association with M/XDR

The development of drug resistance was further investigated with the goal of determining the temporal structure of the Lisboa3 and Q1 clades. Using the genome-wide and high-quality sets of SNP data for each clade the time to the MRCA of each clade and some of its sub-branches was investigated under a Bayesian modelling approach by time-scaling each sub-tree independently according to its’ tip dates (Table 3). In both clades the putative dates for divergence across each node is consistent with drug resistance acquisition following the historical introduction of the respective drugs into anti-TB drug regimens. Under this evolutive scenario the Lisboa3/SIT20/LAM1 strains are thought to have emerged earlier in time when compared with the Q1/SIT1106/LAM4 strains: root age for the Lisboa3 clade falls in the 40’s while the root age for the Q1 clade is placed in the late 60’s. However, despite the age gap between both clades, emergence of the two main Lisboa3 MDR sub-clades and of the MDR-TB sub-branch of the Q1 clade is contemporary to the 70’s (Fig. 3). The third MDR-TB *rpoB*S450L Lisboa3 sub-branch is estimated to have emerged later during the early-middle 80’s from INH^R^/STR^R^ strains, along with the XDR-TB sub-branches derived from the two other Lisboa3 sub-clade which are estimated to have as well emerged in the early 80’s. Q1 XDR-TB strains are estimated to have emerged later in the middle 90’s (Table 3 and Fig. 3).

**Table 3:** Node divergence estimates for the Lisboa3 and Q1 clades along with some of its sub-branches, including drug resistance associated with node and respective mutational profiles.

Clade/Node	No. of Isolates Detected	Node Age Mean	95% HPD Interval	Intra-clade Median SNP Distance	Resistance Associated	Node Mutational Profile
**Lisboa3**	**72**	1950.0	1940.0–1964.5	23		
MDR1	24	1974.5	1956.1–1990.0	11	MDR/XDR	*inhA*:C-15T/S94A; *rpoB*:S450L; *rpsL*:K43R
MDR2	25	1972.9	1947.6–1993.6	10	MDR/XDR	*inhA*:C-15T/S94A; *rpoB*:S450L; *rpsL*:K43R
Lisboa3-A	7	1981.8	1968.7–1991.8	6	MDR	*inhA*:C-15T/S94A; *rpoB*:D435V; *rpsL*:K43R
XDR1	17	1987.8	1978.1–1995.8	8	XDR	*gyrA*:S91P; *eis*:G-10A
XDR2	21	1990.4	1979.1–2000.3	9	XDR	*gyrA*:D94G; *eis*:G-10A
**Q1**	35	1968.1	1942.1–1989.3	10		
MDR	34	1976.5	1954.6–1994.9	10	MDR/XDR	*inhA*:C-15T/I194T; *rpoB*:S450L; *gidB:*A80P
XDR	15	1994.3	1983.5–2003.0	5	XDR	*gyrA*:D94G; *rrs*:A1401G

### Barcoding lisbon and Q1 clades enables global tracking

The high importance of Lisboa3 and Q1 strains at the nationwide level elicits the need for a rapid molecular test based on specific molecular markers. The association of these strains with MDR/XDR-TB is of increasing public health concern. These strains have been circulating for several decades, and therefore might have gradually attained lower fitness costs due to compensatory evolution. We next sought to define a SNP barcode to rapidly identify these strains over large sets of data and to implement on future molecular diagnosis tests (Supplementary Fig. S3). Both phylogenetic tree ancestral reconstruction and population differentiation (F_ST_) approaches converged to the identification of the same sets of clade-defining SNPs. Briefly, 13 putative clade-defining SNPs were identified for the Lisboa3 clade, all of which were intragenic and five resulted in non-synonymous substitutions. In the Q1 clade, a single SNP was identified by both methods, a synonymous substitution to the *nuoH1* gene (Table 4).

**Table 4:** SNP barcode for Lisboa3 and Q1 clades. Clade-specific mutations are highlighted in bold upon validation against a global dataset.

Clade/SNP Position^a^	Gene	Reference Allele	Mutated Allele	Mutation	Mutation Type (Non-) Synonymous	Essential Gene^b^
**Q1**						
**3520172**	Rv3152 (nuoH1)	G	A	Leu297Leu	S	No
**Lisboa3**						
208287	Rv0176	A	C	Glu279Ala	NS	No
405812	Rv0338c	T	C	Ile10Met	NS	Yes
740561	Rv0646c (*lipG*)	C	T	Pro193Pro	S	No
**1057412**	Rv0947c	A	G	*Pseudogene*	—	No
**1153137**	Rv1029 (*kdpA*)	G	A	Ala376Thr	S	No
**1706439**	Rv1514c	G	A	Leu53Leu	S	No*
**1885422**	Rv1662 (*pks8*)	C	T	Ser1240Leu	NS	Yes
2123086	Rv1872c (*lldD2*)	C	A	Pro22Pro	S	No
**2255294**	Rv2006 (*otsB1*)	T	C	Ile198Thr	NS	No**
**2264189**	Rv2017	C	T	His64His	S	Yes
3544710	Rv3176 (*mesT*)	T	C	Pro197Pro	S	No
4034894	Rv3593 (*lpqF*)	G	A	Pro181Pro	S	Yes***
4104091	Rv3664c (*dppC*)	C	T	Ala129Thr	NS	No

^a^SNP position is relative to the *M. tuberculosis* H37Rv genome (GenBank Accession NC000962.3);

^b^Gene essentiality: *essential *in vivo*, non-essential in culture; **non-essential in *M. tuberculosis* H37Rv, essential in *M. tuberculosis* CDC1551; and ***essential by Sassetti *et al*.^45^, non-essential by DeJesus *et al*.^44^.

To validate the specificity of these SNPs to Lisboa3 and Q1 clades, we have screened a global dataset of publicly available *M. tuberculosis* sequence data (n = 28 385). Whereas the single SNP identified for the Q1 clade was exclusively found among strains of the 4.3.4.2 sub-lineage, which includes both Lisboa3 and Q1 clades, seven initial putative Lisboa3-specific SNP were detected among multiple sub-lineages (Supplementary Table S5). The remaining six Lisboa3-specific SNPs were comprised by two non-synonymous and four synonymous single nucleotide variants. Among these six variants, two occurred at genes described as essential to *in vitro* growth of *M. tuberculosis* H37Rv as evaluated through a saturated Himar1 transposon library^44^. The single Q1-specific SNP (synonymous) occurred at a non-essential gene (Rv3152/*nuoH1*)^45^. All six globally validated Lisboa3-specific SNPs were concordant in the set of isolates retrieved from the global dataset thereby demonstrating its robustness as unique Lisboa3 phylogenetic markers (Supplementary Table S5). Mining the country of origin of these isolates and excluding isolates from Portugal, one Q1 isolate is predicted to have been isolated in the United Kingdom; two Lisboa3 isolates from Brazil; two Lisboa3 isolates from Mali; and, one Lisboa3 isolate from Switzerland, respectively.

**Figure 4:**
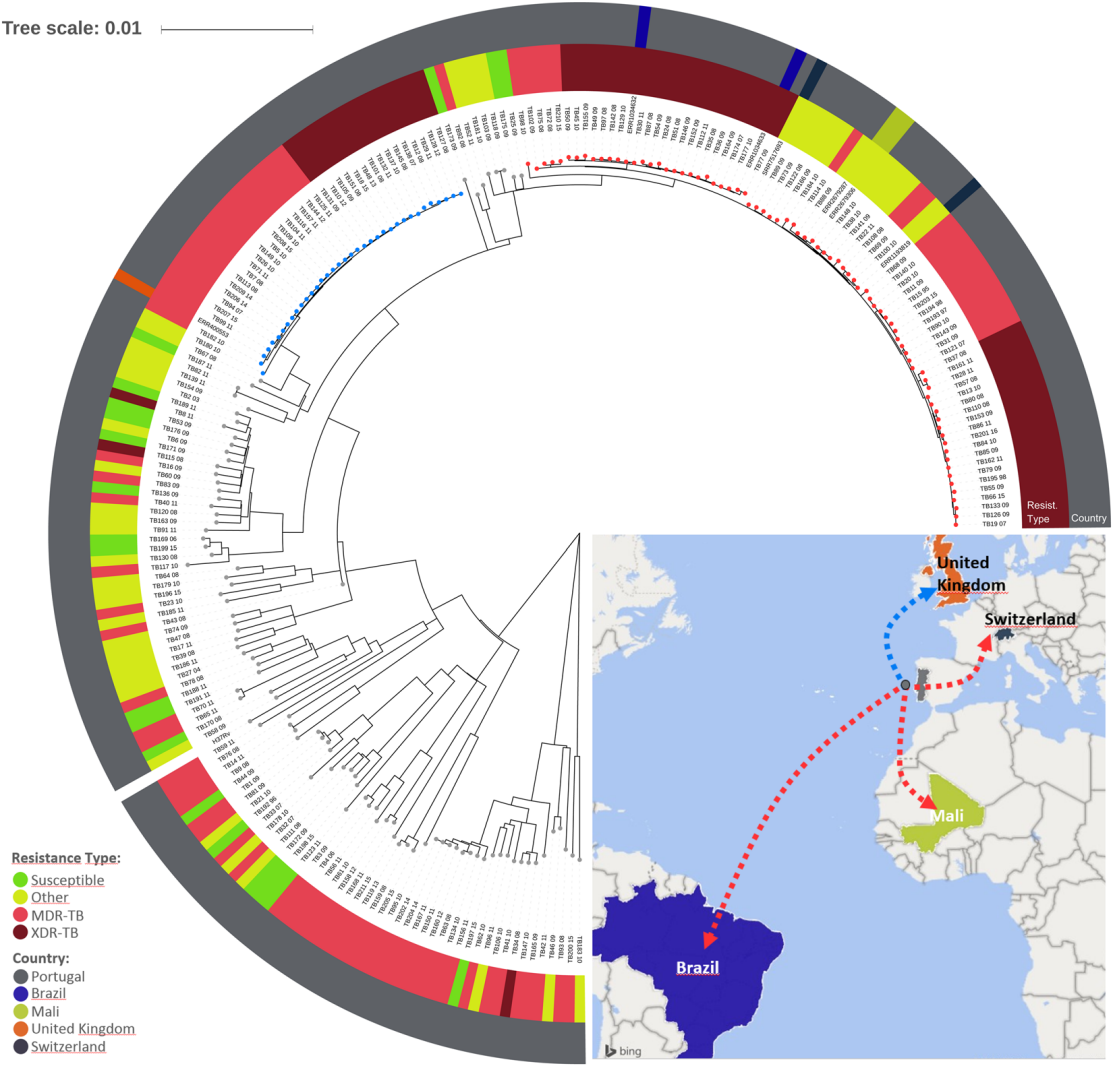
Phylogenetic positioning of international Lisboa3 and Q1 isolates tracked in this study. The tree is annotated with drug resistance type based on mutational profiling (inner ring) and country of origin (outer ring). The tree shows the phylogenetic positioning of six international Lisboa3 isolates among the other Lisboa3 isolates included in the study (red tips) and one international Q1 isolate among other MDR Q1 isolates (blue tips). A map summarizes the main dissemination routes uncovered in the study by Lisboa3 (red arrows) and Q1 (blue arrow) isolates. Figure generated using the Interactive Tree of Life v5 online tool (available at https://itol.embl.de), Microsoft PowerPoint 2016 (Version 1707) and Microsoft Excel 2016 (Version 1707), incl. Microsoft Power Map 3D Data Visualization Tool (https://products.office.com/en-us/business/office).

Initial mutational analysis of the publicly available sequence data for these seven international isolates, specifically of candidate genes associated with resistance, demonstrated that the mutational profiles of the Q1 isolate is congruent with a Q1 MDR sub-branch (Fig. 4). The six Lisboa3 international isolates included four non-MDR isolates and the remaining two isolates, from Brazil, showed a mutational profile compatible with the XDR *gyrA* S91P/*eis* G-10A/*tlyA* Ins755GT sub-branch of the Lisboa3 clade. The phylogenetic reconstruction for all isolates included in the study along with the seven international isolates putatively assigned to the Lisboa3 and Q1 isolates based on the global barcode screening confirmed the phylogenetic placing of these isolates among the Lisboa3 and Q1 isolates (Fig. 4). Interestingly, the Lisboa3 isolates recovered in Mali were at a pair-wise distance of 7 SNPs and the Lisboa3 isolates from Brazil were 12 SNPs apart. Genomic clustering analysis of the identified international isolates demonstrated the existence of cross-border international clustering involving one isolate from Brazil (ERR1034632) which grouped with GC8 isolates and, a newly formed genomic cluster involving the Lisboa3 isolate from Switzerland and TB100_10. Importantly, the Lisboa3 isolate from Switzerland was diagnosed in a Chinese patient and eventual links to Portugal are unknown, possibly suggesting missing links in the form of undetected/unsequenced strains within the international transmission network of Lisboa3 strains^46^.

## Discussion

The WHO has estimated that MDR-TB and XDR-TB are increasing globally, mostly in developing countries. Despite having a first-class health system, MDR-TB and XDR-TB has had a long presence in Portugal^16^, and no study has investigated the resistance transmission dynamics using high-resolution typing approaches at the Portuguese nationwide scope. In fact, in Portugal, the definitions of MDR-TB and XDR-TB were, at the time, new names for extremely old problems^16^. This study encompasses the largest *M. tuberculosis* clinical isolate collection (n = 207), temporally structured over a 20-year timeframe. The sampled isolates reflect the asymmetrical distribution of MDR-TB and XDR-TB in the country, where Lisbon and its surroundings (Lisbon Health Region) are responsible for most of the MDR-TB and XDR-TB reported cases with 80.2% of all isolates present in our study originating from this region. We show that the Lisboa3 and Q1 isolates dominate the MDR/XDR-TB population structure for more than 20 years and not only in this region but nationwide. The data suggests that both clades have emerged in Lisbon, where it had high prevalence, and disseminated to other regions in the country. While these strains can be found at lesser frequencies outside Lisbon and surrounding districts, these clades are the primary drivers of M/XDR-TB, transmission, and most likely to cause disease. The results also show a high predominance of LAM strains, which is consistent with previous data proposing that such strains comprise the hallmark of Portuguese-speaking countries.

Besides the distribution of Lisboa3 and Q1 main clades, the present study contributed with the identification of 16 genomic clusters associated with a clustering rate of 58.3% among M/XDR-TB patients. This high clustering rate reflects the underlying endemicity of the described clades in Portugal but, upon closer inspection of genomic clusters, the heterogenic composition of these clusters regarding drug resistance type and profiles also suggests the *de novo* emergence of MDR-TB in such clusters. Recent data obtained from MIRU-VNTR typing supports an epidemiological setting marked by uncontrolled MDR-TB transmission^47^. The situation is not novel, and the mostly represented MIRU-VNTR clusters therein identified have been previously described^5,48^. Our data corroborates these findings but enables a more in-depth view of transmission over time in Portugal. By including strains with non-MDR-TB phenotypic profiles, this study enabled the identification of transmission clusters in which an initial analysis suggests *de novo* emergence of MDR, which is less supported by individual mutational profiling analysis. Three distinct situations were detected concerning this intra-cluster discrepancy: a single mutation in *rpoB* showed a lower penetrance for drug resistance (H445D); an undescribed and uncalled minor allelic variant was detected upon coverage analysis (*rpoB* T9P) but its role in RIF resistance remains to be elucidated; or, a RIF resistant isolate without a recognizable resistance mechanism that could eventually be a false-resistant. Simultaneously, the microevolutionary topological structure of the tree and cluster association enables to infer on the directionality of drug resistance transmission and to discriminate inside previously described MIRU-VNTR clusters.

Another important point concerning TB transmission relies on the observed paraphyletic clustering for three clusters potentially due to missing epidemiological links in the tree. This phenomenon is likely due to unsampled isolates to the study, or undetected cases, and in this regard, we restress that in Portugal only 61.4% of notified cases are laboratory confirmed-cases and from these only 66.7% have available drug susceptibility data^2^. MDR/XDR-TB undetected cases may therefore be present in the community and acting as potential reservoirs for the dissemination of Lisboa3 and Q1 clades. This hypothesis warrants further investigation and reinforces the urgency in detecting such cases and rapidly screen for drug resistance and clade association to assist the control of these strains that are now evolving polycentrically in multiple districts across the country. Moreover, the identification of non-clustered Beijing strains can be associated with immigration since MDR-TB Beijing strains are not considered endemic in Portugal. The study sample is composed of isolates irrespectively of the patient country of birth and in Portugal, foreign-born patients presently account for 19.5% of all TB patients^2^. The detection of non-clustered MDR-TB Beijing strains might therefore be more associated with migratory movements from endemic countries such as those in Eastern Europe rather than be the result of ongoing transmission of primary MDR-TB in Portugal^34^.

Additionally, the molecular basis of drug resistance in Portugal is herein demonstrated to comprise a unique set and combination of drug resistance mutations. It is noteworthy the putative evolution from INH low level resistance towards intermediate/high-level resistance driven by the acquisition of additional mutations that can either promote target site alteration or prevent INH activation. This process of amplifying the resistance level was previously described in Lisbon through double mutants on the promoter and ORF of *inhA*^15^. Herein, this process is also observed on *inhA*/*katG* double mutants which, to our knowledge, has not been documented before in Portugal. A parallel situation is also thought to have occurred on EMB resistance development: the novel association of *embA* promoter mutations with *embB* mutations such as M306V or P397T is thought to result in increased EMB resistance levels leading to more robust association between phenotypic susceptibility and genotype. In this regard it is interesting to note that RV3806c/*ubiA* appears to have no clinical relevance for EMB resistance in Portugal since only four isolates were found to bear mutations in this gene without a robust association with EMB resistance. Rv3806c/*ubiA* codes for a decaprenyl-phosphate 5-phosphoribosyltransferase (DPPR synthase) whose overexpression leads to increased levels of decaprenylphosphoryl-d-arabinose (DPA) and higher resistance levels to EMB^49^. Mutations in this gene were proposed to cooperate synergistically with *embB* mutations leading to higher EMB resistance levels^50^. Conflicting reports do, however, highlight a variable correlation between specific *ubiA* mutations and EMB resistance while also stressing the association between *embB* and *embA* mutations such as the those herein reported and whose order of emergence could be traced at the microevolutionary level^51,52^.

By examining microevolutionary patterns, mutations leading to the amelioration of fitness costs imposed by RIF resistance acquisition were putatively identified to occur at multiple nodes in the phylogenetic tree. These compensatory mutations were detected among the *rpoB* (L731P, V496A/M) and *rpoC* (V483G, K1152Q) genes and fulfill the criteria for drug resistance compensatory candidate mutations since these were only found to occur after drug resistance acquisition. The role of compensatory mutations in reducing fitness costs has been previously established using *in vitro* competition assays suggesting that increased circulation can lead to the accumulation of such mutations^36^. The prolonged circulation of Lisboa3 and Q1 clades argues in favour of this previous notion: highly resistant and uncontained strains can achieve epidemiological success despite the accumulation of mutations in a high number of genes essential for *in vitro* and *in vivo* survival of *M. tuberculosis*.

Interestingly, the microevolutionary trajectory of *M. tuberculosis* Lisboa3 strains is set down a path that favoured the acquisition of mutations in *eis* promoter region and, in one sub-branch, resistance to CAP has emerged subsequently by a *tlyA* frameshift mutation due to a binucleotidic insertion. Increased TlyA levels are proposed to comprise a new resistance compensation mechanism upon development of *rrs* mutations associated with resistance to SLIDs^53^. Contrarily to a second *eis* G-10A sub-branch in the Lisboa3 clade, where at least one isolate was found to have acquired a *rrs* A1401G mutation that potentially leads to higher KAN/AMK resistance levels and CAP resistance, this was never observed for isolates which bear the *tlyA* Ins755GT. Here, we propose that it may be due to the inability of these strains to compensate such fitness deficits imposed by *rrs* mutations since these bear a non-functional TlyA methyltransferase. As CAP is being gradually replaced by AMK, our data suggests that from an evolutionary viewpoint CAP could eventually play a role in preserving AMK susceptibility since CAP resistance may be driven by *tlyA* loss of function which can pose an obstacle for the emergence of additional resistance to SLIDs mediated by *rrs* mutations^54^.

The epidemiological history of MDR and XDR-TB in Portugal can to some degree be paralleled with the also long-term evolutionary trajectory of the F15/LAM4/KZN strains towards XDR in KwaZulu-Natal, South Africa^55^. As for the KZN strains, in Lisboa3 and Q1 strains accumulation of drug resistance mutations leading to resistance to M/XDR-TB defining drugs appears to have taken place following its introduction to TB treatment regimens since MDR is predicted to have emerged in the 70’s and XDR-TB later during the 80’s or 90’s, depending on the clade and its sub-branch^56,57^. As with the KZN strain, it is interesting to note that MDR-TB in Portugal also started out before the global HIV pandemic became aware. Albeit the Lisboa3 and Q1 strains were highly associated with HIV-coinfected patients, we can speculate that it might have not played a major role in the emergence of MDR-TB or even XDR-TB but, as already reported in the first accounts, these strains became highly predominant among the HIV-infected population^4^. This latter factor might have accelerated the accumulation of resistance to other drugs thereby increasing the overall prevalence in the community. However, unlike to the KZN strains, both Lisboa3 belong to a distinct LAM branch (RD^RIO^), acquired INH resistance via *inhA* mutations and not *katG* and, co-occur in a setting where these sympatric microevolutionary trajectories towards XDR-TB show some degree of similarity. The combination of these factors turns the epidemiological history of MDR/XDR-TB in Portugal unique and alongside with other well-known emergence of MDR/XDR-TB epidemiological events that occurred in the last 20 years worldwide^57^.

Since detection of these strains is a crucial step towards its containment, we validate a set of Single Nucleotide Variants which can be used as a barcode for Lisboa3 and Q1 strains and, able to inform on the development of molecular tests that can take advantage of highly specific markers obtained from high-throughput sequencing data. Such strategies have already proved useful in multiple settings by assisting in the detection of highly prevalent strains and, in the Portuguese setting, should be complemented by extended contact tracing towards a more efficient active case finding strategy^58,59^. The importance of early detection can also inform on clinical decision and tailored therapeutic adjustment: Lisboa3 and Q1 strains are associated with a diminished treatment success and median survival rates^60^. Such, is likely associated with the increasing number of resistance mutations accumulated by these strains which clearly impacts on the treatment success rate and provides a challenge to early treatment initiation as most empirical treatment regimens are unlikely to adequately meet the drugs to which these strains are susceptible. In this regard, empirical treatment regimens for MDR-TB containing any first-line drug as well as ethionamide is discouraged since most 49% of all MDR-TB strains are resistant to all first-line drugs and the promoter/ORF mutations responsible for INH resistance among Lisboa3 and Q1 clades are also known to mediate ethionamide resistance^5,12,15^.

Recent evidence points out to MDR-TB as a mostly local problem, and one that emerges at the country-level and that cross-border transmission is minimal^61^. Although true that MDR-TB is locally driven situation, an increasingly number of MDR-TB cases have been shown to result of cross-border transmission, including those corroborated by genomic data^9,62,63^. Using the Lisboa3 and Q1 specific barcode to track Lisboa3 and Q1 strains across a global *M. tuberculosis* genomic dataset demonstrated the outreach of these two endemic strains to Portugal: its presence was detected in three continents, including countries other than Portugal. Previous data based on MIRU-VNTR surveillance conducted by the European Center for Disease Prevention and Control has previously identified the specific MtbC15–9 MIRU-VNTR types of the Lisboa3-B and Q1 clusters as European cross-border clusters due to its detection in France and the United Kingdom, respectively^64^. Herein we demonstrate a more extensive international spread using more robust epidemiological markers while positioning these Lisboa3 and Q1 international isolates in the microevolutionary context of both clades. This international spread was, nevertheless, more notorious among Lisboa3 strains, which can be potentially driven by a combination of the higher genetic divergence found in the study and longer presence in the community. In fact, Lisboa3 strains are thought to have emerged earlier than Q1 strains and have attained a considerably higher epidemiological success during the 90’s. In that point in time the genetic profile of Q1 strains among MDR clinical isolates were detected at a decreased frequency when compared with Lisboa strains. However, our data shows that over the last 15 years Q1 strains have gradually emerged as a highly success M/XDR-TB in Portugal, comparable to the Lisboa3 clade.

### Concluding remarks

The present study contributes with important and novel data on the TB epidemiology in Portugal and provides a framework for differentiation of the two most prevalent strains in this setting while enabling the study of its global dissemination. Overall, the data shows a highly endemic situation for MDR-TB in Portugal that is nevertheless partially fuelled by a minor proportion of non-clustered clinical isolates, such as those from Lineage2/Beijing, which can potentially emerge due to populational migratory events. The evolutionary history of the two main clades, Lisboa3 and Q1, highlights limitations in the diagnosis of drug resistance and implementation of adequate treatment regimens that existed for several decades prior to the deployment of robust phenotypic-based methods and molecular testing. Now, the control of MDR-TB in Portugal requires special attention with great emphasis in controlling primary MDR-TB by breaking ongoing transmission chains that continuously fuel the dissemination of these strains. Importantly, the present study also brings the realization that Lisboa3 and Q1 strains now occur and evolve outside Lisbon Health Region, a fact for which health professionals must be made aware of. In this context rapid screening based on the molecular markers uncovered in the present study can play a key role in fast tracking these strains, potentiating active case finding strategies over passive case finding, and thereby contributing to an improved programmatic management of MDR-TB.

## Supplementary information

Supplementary Information.

Supplementary Information 2.

Supplementary Information 3.

Supplementary Information 4.

Supplementary Information 5.

Supplementary Information 6.

## Data Availability

All raw sequence data used in this study is available at the European Nucleotide Archive under Study accessions ERP002611 and ERP011639.
