# Supplementary material for: Using genomics to understand the origin and dispersion of multidrug and extensively drug resistant tuberculosis in Portugal
Source: Sci Rep. 2020 Feb 13;10:2600. doi: 10.1038/s41598-020-59558-3 (PMC7018963; doi:10.1038/s41598-020-59558-3)
Supplement: Supplementary file 1 — Supplementary Information. [file 41598_2020_59558_MOESM1_ESM.pdf]

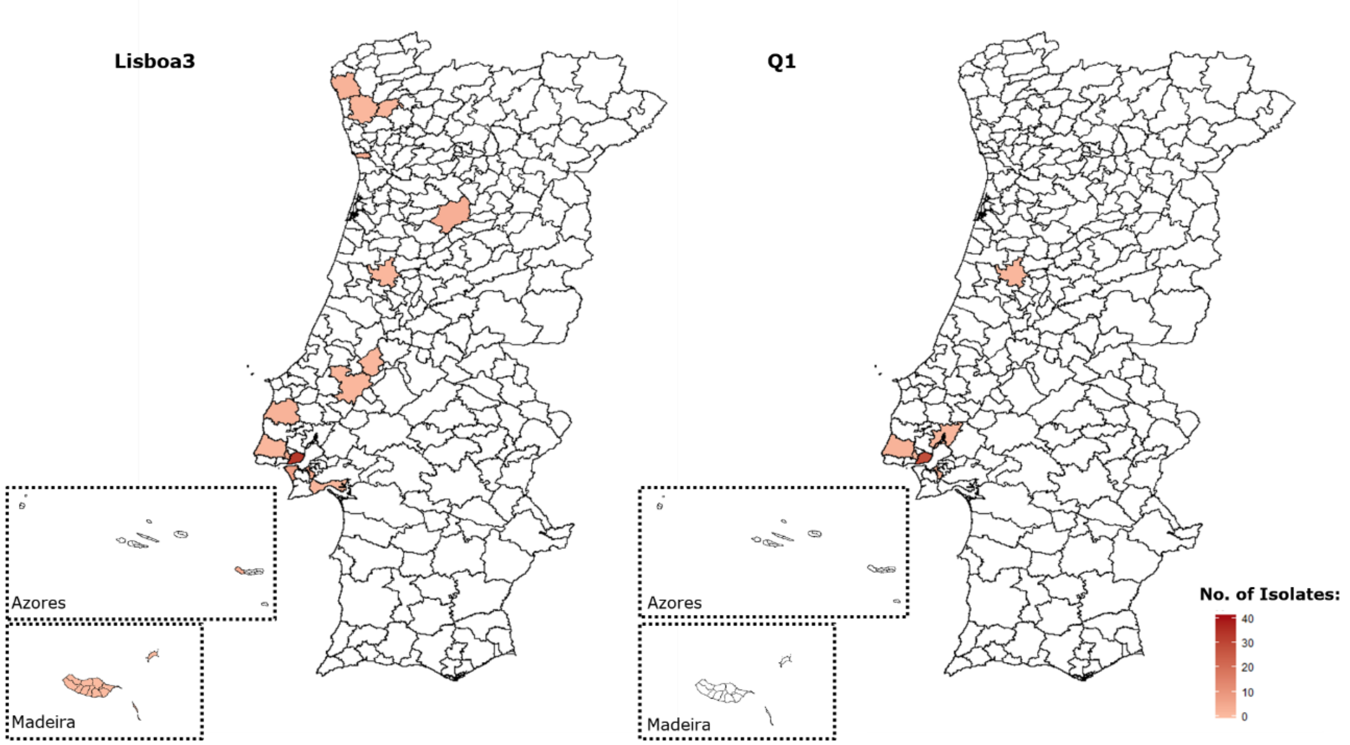

Supplementary Figure S1 – Geographical distribution of the two main clades in the study (Lisboa3 and Q1) across Portugal continental territory and islands. Distribution is herein shown at the sub-district level and clearly shows a widespread distribution of Lisboa3 strains at the nationwide level, including Azores and Madeira archipelagos. Figure generated using R software for statistical computing (version 3.6.0) along with the maptools, ggmap, rgeos and plotGoogleMaps packages (available at https://cran.r-project.org).

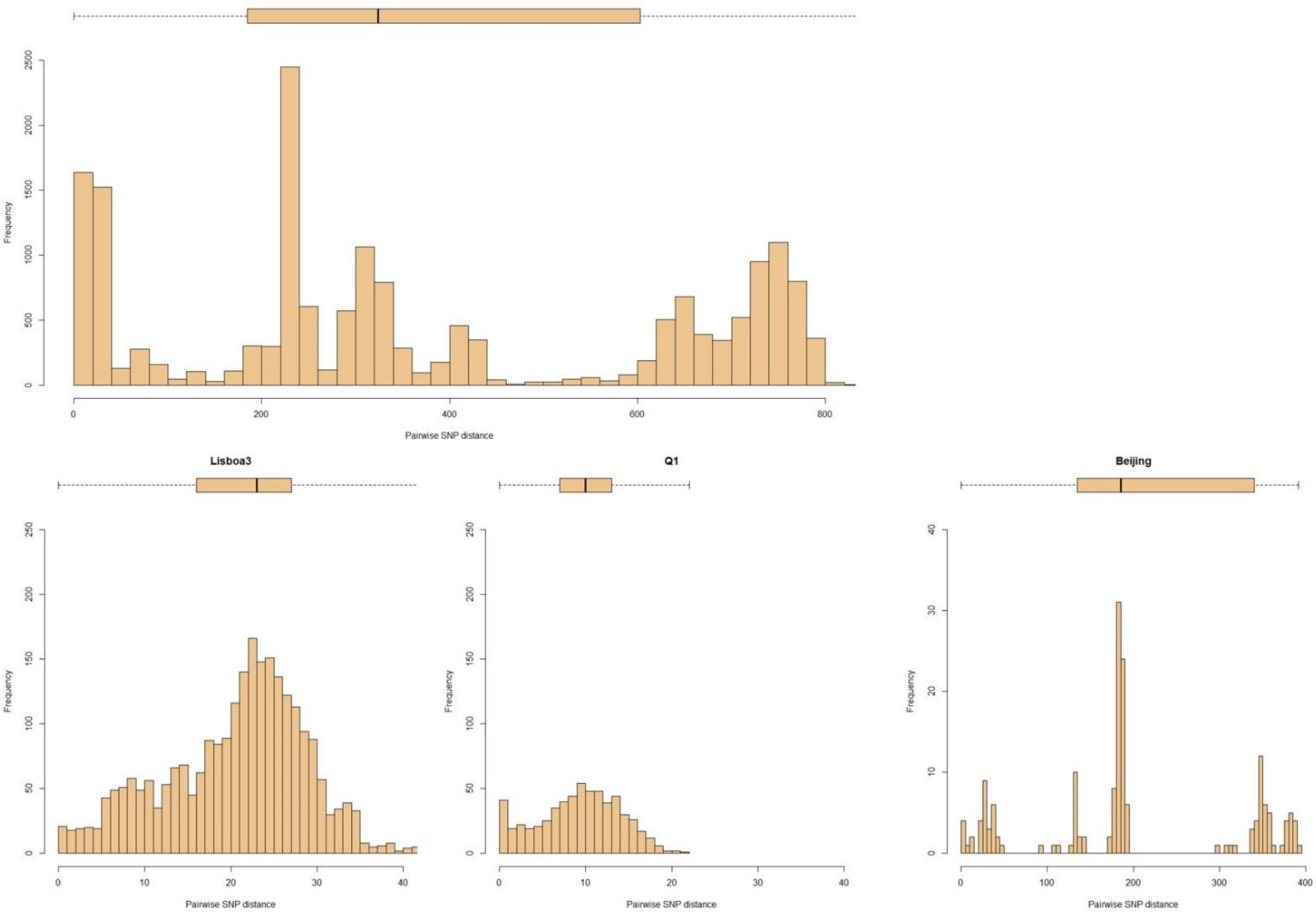

Supplementary Figure S2 – SNP pairwise distance distribution across all 207 *M. tuberculosis* clinical isolates included in the study (top panel) and, within the Lisboa3, Q1 and Beijing clade isolates present among the 207 clinical isolates.

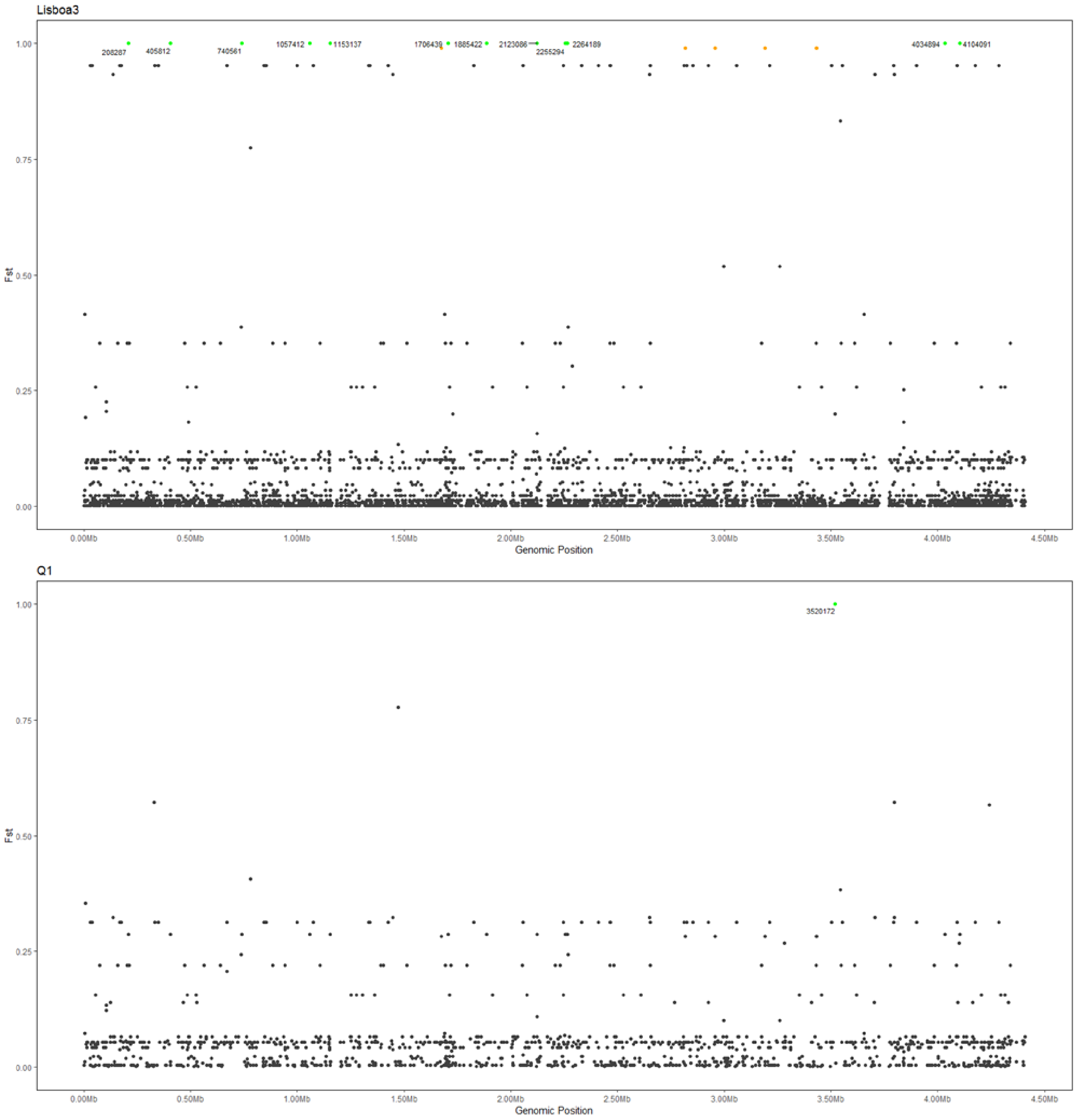

Supplementary Figure S3 – F_{ST} scores obtained for individual SNPs for the Lisboa3 and Q1 clades versus non-Lisboa3 and non-Q1 isolates, respectively. F_{ST} scores are plotted in function of the SNP genomic position relative to *M. tuberculosis* H37Rv (GenBank Accession NC000962.3). F_{ST} scores equal to 1.0 or 0.99 are highlighted in green or orange, respectively, and in both cases the genomic position is annotated next to the corresponding data point.
