# Supplementary material for: Using genomics to understand the origin and dispersion of multidrug and extensively drug resistant tuberculosis in Portugal
Source: Sci Rep. 2020 Feb 13;10:2600. doi: 10.1038/s41598-020-59558-3 (PMC7018963; doi:10.1038/s41598-020-59558-3)
Supplement: Supplementary file 2 — Supplementary Information 2. [file 41598_2020_59558_MOESM2_ESM.pdf]

Supplementary Table S1 - Individual characterization of the clinical isolates included in the study: year of isolation, district, genotypic characterization and phenotypic drug susceptibility data.

Isolate ID	Year of Isolation	District	SIT	Spoligotyping Clade	Clade	Sub-lineage	Genomic Cluster	Resistance	Phenotypic Drug Susceptibility										Mapping Statistics[a]	
									INH	RIF	STR	EMB	PZA	KAN	AMK	CAP	OFX	ETH	Mean Read Depth	Coverage (%)
TB1_09	2009	Porto	92	X3	Other	4.1.1.3	NC	MDR	R	R	R	S	S	S	S	S	S	S	367.93	99.49
TB10_12	2012	Setubal	1106	LAM4	Q1	4.3.4.2	CC67	MDR	R	R	R	R	R	ND	ND	ND	ND	ND	949.26	99.49
TB100_10	2010	Braga	20	LAM1	Lisboa3	4.3.4.2	NC	MDR	R	R	R	S	R	ND	S	S	S	R	362.53	98.78
TB101_08	2008	Lisboa	1106	LAM4	Q1	4.3.4.2	CC67	XDR	R	R	R	R	R	R	R	R	R	R	155.06	98.02
TB102_09	2009	Porto	20	LAM1	Lisboa3	4.3.4.2	CC8	MDR	R	R	R	S	S	S	S	S	S	R	443.40	98.83
TB103_09	2009	Lisboa	42	LAM9	Other	4.3.4.2	CC48	MDR	R	R	R	S	S	S	S	S	S	S	350.22	98.85
TB104_11	2011	Lisboa	1106	LAM4	Q1	4.3.4.2	CC67	MDR	R	R	R	R	R	ND	R	R	S	R	52.47	98.90
TB105_09	2009	Lisboa	1106	LAM4	Q1	4.3.4.2	CC67	XDR	R	R	R	R	R	ND	R	R	R	R	173.05	98.86
TB106_10	2010	Lisboa	1	BEIJING	Other	2.2.1	NC	MDR	R	R	R	R	R	R	R	S	S	R	313.99	99.67
TB108_08	2008	Viana do castelo	20	LAM1	Lisboa3	4.3.4.2	CC90	MDR	R	R	R	S	S	ND	ND	ND	ND	R	97.63	99.16
TB109_10	2010	Lisboa	1106	LAM4	Q1	4.3.4.2	CC67	MDR	R	R	R	R	R	R	R	R	S	R	314.72	98.87
TB11_09	2009	Setubal	20	LAM1	Lisboa3	4.3.4.2	CC3	MDR	R	R	R	S	R	ND	S	S	S	R	175.22	98.83
TB110_08	2008	Lisboa	20	LAM1	Lisboa3	4.3.4.2	CC82	XDR	R	R	R	S	R	R	S	S	R	R	159.99	98.32
TB111_08	2008	Porto	137	X2	Other	4.1.1.1	NC	Other	S	R	S	S	S	S	S	S	S	S	68.81	98.70
TB112_11	2011	Lisboa	20	LAM1	Lisboa3	4.3.4.2	CC8	XDR	R	R	R	R	R	ND	R	R	R	R	175.83	98.67
TB113_08	2008	Lisboa	1106	LAM4	Q1	4.3.4.2	CC31	MDR	R	R	S	S	R	ND	ND	ND	ND	ND	78.33	98.83
TB114_10	2010	Lisboa	20	LAM1	Lisboa3	4.3.4.2	NC	MDR	R	R	R	S	R	R	R	R	S	R	400.21	98.79
TB115_08	2008	Porto	20	LAM1	Other	4.3.4.1	NC	MDR	R	R	R	R	R	R	R	R	S	S	159.68	98.83
TB116_11	2011	Lisboa	1106	LAM4	Q1	4.3.4.2	CC67	MDR	R	R	R	R	R	R	R	R	S	R	131.76	98.84
TB117_10	2010	Porto	179	LAM2	Other	4.3.4.1	NC	MDR	R	R	R	R	R	R	R	R	S	R	338.90	98.97
TB118_09	2009	Lisboa	42	LAM9	Other	4.3.4.2	CC48	Other	R	S	R	S	S	S	S	S	S	S	210.89	98.82
TB119_13	2013	Lisboa	262	H3	Other	4.2.1	NC	MDR	R	R	R	R	R	ND	S	S	S	R	133.09	99.48
TB12_08	2008	Lisboa	1106	LAM4	Q1	4.3.4.2	CC67	XDR	R	R	R	R	R	R	R	R	R	R	73.75	98.00
TB120_08	2008	Setubal	20	LAM1	Other	4.3.4.1	NC	Other	R	S	S	S	S	ND	ND	ND	ND	ND	70.08	98.99
TB121_07	2007	Lisboa	20	LAM1	Lisboa3	4.3.4.2	CC7	MDR	R	R	R	S	S	ND	ND	ND	ND	ND	293.91	99.12
TB122_08	2008	Setubal	20	LAM1	Lisboa3	4.3.4.2	NC	Other	R	S	R	S	S	ND	ND	ND	ND	ND	75.69	98.92
TB123_11	2011	Braga	450		Other	4.1	NC	Susceptible	S	S	S	S	S	ND	S	S	S	S	58.35	99.15
TB125_11	2011	Lisboa	1106	LAM4	Q1	4.3.4.2	CC67	MDR	R	R	R	R	R	ND	R	R	S	R	38.61	98.83
TB126_09	2009	Lisboa	20	LAM1	Lisboa3	4.3.4.2	CC5	MDR	R	R	R	R	R	ND	S	S	R	R	143.45	98.75
TB127_08	2008	Lisboa	1106	LAM4	Q1	4.3.4.2	CC67	XDR	R	R	R	R	R	R	R	R	R	R	187.71	98.87
TB128_12	2012	Lisboa	1106	LAM4	Q1	4.3.4.2	CC67	XDR	R	R	R	R	R	ND	R	R	R	R	183.05	98.81
TB129_10	2010	Lisboa	20	LAM1	Lisboa3	4.3.4.2	CC8	XDR	R	R	R	R	S	S	S	R	R	R	567.14	98.79
TB13_10	2010	Porto	20	LAM1	Lisboa3	4.3.4.2	CC5	XDR	R	R	R	R	R	R	S	S	R	R	625.51	99.13
TB130_08	2008	Lisboa	17	LAM2	Other	4.3.4.1	NC	MDR	R	R	S	S	S	S	S	S	S	R	123.51	98.99
TB131_09	2009	Coimbra	1106	LAM4	Q1	4.3.4.2	CC67	XDR	R	R	R	R	R	R	R	R	R	R	368.91	98.87
TB132_11	2011	Lisboa	1106	LAM4	Q1	4.3.4.2	CC67	XDR	R	R	S	R	R	R	R	R	R	R	146.10	98.76
TB133_09	2009	Lisboa	20	LAM1	Lisboa3	4.3.4.2	CC5	MDR	R	R	R	R	R	ND	S	S	R	R	75.96	98.75
TB134_10	2010	Porto	1	BEIJING	Other	2.2.1	NC	MDR	R	R	R	S	R	S	S	S	S	S	455.99	99.34
TB136_09	2009	Lisboa	Orphan		Other	4.3.4.1	NC	Susceptible	S	S	S	S	S	S	S	S	S	S	348.06	98.98
TB137_10	2010	Lisboa	1106	LAM4	Q1	4.3.4.2	CC67	XDR	R	R	R	R	R	R	R	R	R	R	136.92	98.79
TB138_07	2007	Lisboa	1106	LAM4	Q1	4.3.4.2	CC67	XDR	R	R	R	R	R	ND	R	R	R	R	132.97	98.39
TB139_11	2011	Lisboa	59	LAM11-ZWE	Other	4.3.4.2.1	CC52	MDR	R	R	R	R	R	S	S	S	S	S	69.95	98.70
TB14_11	2011	Lisboa	34	S	Other	4.4.1.1	CC119	MDR	R	R	S	R	S	S	S	S	S	R	134.56	99.14
TB140_10	2010	Setubal	20	LAM1	Lisboa3	4.3.4.2	CC3	MDR	R	R	R	S	R	S	S	S	S	R	516.33	98.92
TB141_09	2009	Santarem	20	LAM1	Lisboa3	4.3.4.2	CC53	MDR	R	R	R	R	S	S	S	S	S	R	411.88	98.71
TB142_08	2008	Lisboa	20	LAM1	Lisboa3	4.3.4.2	CC8	XDR	R	R	R	R	R	R	S	R	R	R	94.68	98.22
TB143_09	2009	Lisboa	20	LAM1	Lisboa3	4.3.4.2	CC7	MDR	R	R	R	S	S	S	S	S	S	R	315.17	98.85
TB144_12	2012	Lisboa	1106	LAM4	Q1	4.3.4.2	CC67	MDR	R	R	S	S	R	R	R	R	S	R	110.13	98.83
TB145_08	2008	Lisboa	1106	LAM4	Q1	4.3.4.2	CC67	XDR	R	R	R	R	R	ND	R	R	R	R	583.79	98.83
TB146_09	2009	Lisboa	20	LAM1	Lisboa3	4.3.4.2	CC8	XDR	R	R	R	R	S	R	S	R	R	R	289.08	98.71
TB147_10	2010	Braga	1	BEIJING	Other	2.2.1	NC	MDR	R	R	R	R	S	S	S	S	S	S	543.37	99.28
TB148_10	2010	Santarem	20	LAM1	Lisboa3	4.3.4.2	CC53	Other	R	S	R	S	S	ND	S	S	S	R	496.12	98.84
TB149_10	2010	Lisboa	1106	LAM4	Q1	4.3.4.2	CC67	MDR	R	R	R	R	R	S	R	R	S	R	521.83	98.80
TB15_95	1995	Setubal	20	LAM1	Lisboa3	4.3.4.2	CC3	MDR	R	R	R	S	R	ND	ND	ND	ND	ND	159.78	98.78
TB150_11	2011	Santarem	1	BEIJING	Other	2.2.1	NC	MDR	R	R	R	S	S	ND	S	S	S	R	76.71	99.30
TB151_08	2008	Lisboa	1106	LAM4	Q1	4.3.4.2	CC67	XDR	R	R	S	S	R	ND	R	R	R	R	62.99	98.80
TB152_09	2009	Lisboa	20	LAM1	Lisboa3	4.3.4.2	CC8	XDR	R	R	R	S	S	ND	R	R	R	R	86.44	98.74
TB153_09	2009	Lisboa	20	LAM1	Lisboa3	4.3.4.2	CC5	XDR	R	R	R	R	R	R	S	S	R	R	353.77	98.84
TB154_09	2009	Lisboa	811	LAM4	Other	4.3.4.2.1	NC	Other	R	S	R	S	S	ND	ND	ND	ND	ND	60.33	98.55
TB155_09	2009	Porto	20	LAM1	Lisboa3	4.3.4.2	NC	MDR	R	R	R	S	R	S	S	S	R	R	463.73	98.74
TB156_11	2011	Porto	1	BEIJING	Other	2.2.1	NC	MDR	R	R	R	R	R	S	S	S	S	R	155.27	99.09
TB157_11	2011	Lisboa	1106	LAM4	Q1	4.3.4.2	CC67	MDR	R	R	R	R	R	ND	R	R	S	R	158.44	98.81
TB158_12	2012	Lisboa	45	H1	Other	4.1.2.1	NC	MDR	R	R	R	R	R	ND	S	S	S	R	742.97	99.93
TB159_08	2008	Lisboa	262	H3	Other	4.2.1	NC	MDR	R	R	R	S	S	S	S	S	S	R	135.17	99.33
TB16_09	2009	Porto	20	LAM1	Other	4.3.4.1	NC	MDR	R	R	S	S	S	S	S	S	S	S	323.25	98.87
TB160_12	2012	Lisboa	1	BEIJING	Other	2.2.1	CC30	MDR	R	R	R	R	R	ND	ND	ND	ND	ND	488.96	99.53
TB161_11	2011	Viseu	20	LAM1	Lisboa3	4.3.4.2	CC141	XDR	R	R	R	R	R	R	R	R	R	R	355.44	98.55
TB162_11	2011	Ponta Delgada	20	LAM1	Lisboa3	4.3.4.2	CC5	XDR	R	R	R	R	R	R	S	S	R	R	127.59	98.76
TB163_09	2009	Setubal	20	LAM1	Other	4.3.4.1	NC	Other	R	S	S	S	S	ND	ND	ND	ND	ND	212.38	98.90
TB164_09	2009	Lisboa	20	LAM1	Lisboa3	4.3.4.2	CC8	XDR	R	R	R	R	R	R	S	R	R	R	331.30	98.77
TB165_09	2009	Lisboa	1	BEIJING	Other	2.2.1	NC	MDR	R	R	S	S	S	ND	S	S	S	R	56.22	99.32
TB166_09	2009	Setubal	20	LAM1	Lisboa3	4.3.4.2	NC	Other	R	S	R	S	S	S	S	S	S	R	414.79	98.78
TB167_11	2011	Lisboa	1	BEIJING	Other	2.2.1	CC55	MDR	R	R	R	S	S	ND	ND	ND	ND	ND	79.09	99.13
TB168_11	2011	Lisboa	45	H1	Other	4.1.2.1	NC	MDR	R	R	R	S	S	S	S	S	S	S	175.00	99.56
TB169_06	2006	Lisboa	17	LAM2	Other	4.3.4.1	NC	Other	R	S	R	S	S	ND	ND	ND	ND	ND	81.51	98.83
TB17_11	2011	Porto	33	LAM3	Other	4.3.2	NC	Other	R	S	R	S	S	ND	S	S	S	S	120.76	99.75
TB170_08	2008	Lisboa	Orphan		Other	4.8	NC	Susceptible	S	S	S	S	S	ND	ND	ND	ND	ND	972.25	99.49
TB171_09	2009	Lisboa	20	LAM1	Other	4.3.4.1	NC	Susceptible	S	S	S	S	S	ND	ND	ND	ND	ND	246.95	98.86
TB172_09	2009	Lisboa	137	X2	Other	4.1.1.1	NC	Susceptible	S	S	S	S	S	ND	ND	ND	ND	ND	533.27	99.53
TB173_09	2009	Lisboa	1803	LAM1	Other	4.3.4.2	NC	Susceptible	S	S	S	S	S	ND	ND	ND	ND	ND	1146.58	99.55
TB174_07	2007	Lisboa	20	LAM1	Lisboa3	4.3.4.2	CC8	XDR	R	R	R	R	R	ND	R	R	R	R	115.92	98.41
TB175_09	2009	Lisboa	42	LAM9	Other	4.3.4.2	NC	Susceptible	S	S	S	S	S	ND	ND	ND	ND	ND	86.19	98.65
TB176_09	2009	Lisboa	Orphan		Other	4.3.4.1	NC	Susceptible	S	S	S	S	S	ND	ND	ND	ND	ND	76.35	98.67
TB177_10	2010	Lisboa	20	LAM1	Lisboa3	4.3.4.2	CC8	XDR	R	R	R	R	R	ND	S	R	R	R	140.73	98.55
TB178_10	2010	Lisboa	119	X1	Other	4.1.1.3	NC	XDR	R	R	R	S	S	ND	R	S	R	S	135.68	99.80
TB179_10	2010	Lisboa	64	LAM6	Other	4.3.3	NC	Other	R	S	S	S	S	ND	ND	ND	ND	ND	44.29	99.42
TB18_15	2015	Lisboa	1106	LAM4	Q1	4.3.4.2	CC67	MDR	R	R	R	R	R	ND	ND	ND	ND	ND	709.74	99.37
TB180_10	2010	Setubal	1106	LAM4	Q1	4.3.4.2	NC	Other	R	S	R	S	S	ND	ND	ND	ND	ND	91.37	98.81
TB181_10	2010	Lisboa	42	LAM9	Other	4.3.4.2	NC	Other	R	S	S	S	S	ND	ND	ND	ND	ND	76.03	98.57
TB182_10	2010	Lisboa	1106	LAM4	Q1	4.3.4.2	NC	MDR	R	R	R	S	R	ND	S	S	S	R	76.57	98.76
TB183_10	2010	Lisboa	26	CAS1-DELHI	Other	3	NC	Other	R	S	S	S	S	ND	ND	ND	ND	ND	34.90	99.78
TB184_10	2010	Lisboa	20	LAM1	Lisboa3	4.3.4.2	NC	Other	R	S	R	S	S	ND	ND	ND	ND	ND	52.14	98.78
TB185_11	2011	Lisboa	Orphan		Other	4.3.3	NC	Other	R	S	S	S	S	ND	ND	ND	ND	ND	70.54	99.71
TB186_11	2011	Lisboa	106		Other	4.3.2	NC	Susceptible	S	S	S	S	S	ND	ND	ND	ND	ND	134.83	99.75
TB187_11	2011	Lisboa	42	LAM9	Other	4.3.4.2	NC	Susceptible	S	S	S	S	S	ND	ND	ND	ND	ND	73.59	98.78
TB188_11	2011	Lisboa	53	T1	Other	4.8	NC	Other	S	S	R	S	S	ND	ND	ND	ND	ND	92.46	99.81
TB189_11	2011	Lisboa	20	LAM1	Other	4.3.4.1	NC	Susceptible	S	S	S	S	S	ND	ND	ND	ND	ND	144.83	98.73
TB19_07	2007	Lisboa	20	LAM1	Lisboa3	4.3.4.2	CC5	XDR	R	R	R	R	R	ND	R	S	R	R	136.61	98.41
TB191_11	2011	Lisboa	53	T1	Other	4.8	NC	Susceptible	S	S	S	S	S	ND	ND	ND	ND	ND	152.92	99.43
TB192_96	1996	Lisboa	91	X3	Other	4.1.1.3	NC	Susceptible	S	S	S	S	S	ND	ND	ND	ND	ND	825.92	99.71
TB193_97	1997	Lisboa	20	LAM1	Lisboa3	4.3.4.2	CC3	MDR	R	R	R	S	ND	ND	ND	ND	ND	ND	606.29	98.89
TB194_98	1998	Lisboa	20	LAM1	Lisboa3	4.3.4.2	CC3	MDR	R	R	R	S	ND	ND	ND	ND	ND	ND	575.74	98.89
TB195_98	1998	Lisboa	20	LAM1	Lisboa3	4.3.4.2	CC5	MDR	R	R	R	S	ND	ND	ND	ND	ND	ND	748.18	98.90
TB196_15	2015	Lisboa	42	LAM9	Other	4.3.3	NC	Susceptible	S	S	S	S	S	ND	ND	ND	ND	ND	810.38	99.83
TB197_15	2015	Lisboa	1	BEIJING	Other	2.2.1	NC	Susceptible	S	S	S	S	S	ND	ND	ND	ND	ND	518.16	99.58
TB198_15	2015	Lisboa	137	X2	Other	4.1.1.1	NC	Susceptible	S	S	S	S	S	ND	ND	ND	ND	ND	1121.02	99.80
TB199_15	2015	Lisboa	17	LAM2	Other	4.3.4.1	NC	Susceptible	S	S	S	S	S	ND	ND	ND	ND	ND	630.53	99.40
TB2_03	2003	Lisboa	Orphan		Other	4.3.4.1	NC	Other	S	R	S	S	S	ND	ND	ND	ND	ND	633.39	98.83
TB20_10	2010	Setubal	20	LAM1	Lisboa3	4.3.4.2	CC3	MDR	R	R	R	S	R	S	S	S	S	R	380.55	98.98
TB200_15	2015	Lisboa	357	CAS	Other	3	NC	MDR	R	R	R	S	S	S	S	S	S	S	1042.78	99.93
TB201_16	2016	Lisboa	20	LAM1	Lisboa3	4.3.4.2	CC5	XDR	R	R	R	R	R	R	S	S	R	R	676.04	99.38
TB202_14	2014	Lisboa	1	BEIJING	Other	2.2.1	CC55	MDR	R	R	R	R	S	S	S	S	S	R	1150.31	99.78
TB203_15	2015	Lisboa	20	LAM1	Lisboa3	4.3.4.2	NC	MDR	R	R	R	R	R	S	S	S	S	R	427.73	99.05
TB204_14	2014	Lisboa	1	BEIJING	Other	2.2.1	CC55	MDR	R	R	R	R	S	S	S	S	S	R	802.00	99.72
TB205_15	2015	Lisboa	262	H3	Other	4.2.1	NC	MDR	R	R	R	R	R	R	R	R	S	S	404.30	99.80
TB206_14	2014	Lisboa	1106	LAM4	Q1	4.3.4.2	CC160	MDR	R	R	S	S	R	S	S	S	S	R	322.63	99.03
TB207_15	2015	Lisboa	1106	LAM4	Q1	4.3.4.2	CC160	MDR	R	R	R	R	R	S	S	S	S	R	409.62	99.18

TB208_15	2015	Lisboa	1106	LAM4	Q1	4.3.4.2	CC67	MDR	R	R	R	R	R	R	R	R	S	R	378.09	99.07
TB209_14	2014	Lisboa	1106	LAM4	Q1	4.3.4.2	CC160	MDR	R	R	S	S	R	S	S	S	S	R	554.92	99.47
TB21_10	2010	Aveiro	92	X3	Other	4.1.1.3	NC	MDR	R	R	R	S	S	S	S	S	S	S	596.68	99.53
TB210_15	2015	Lisboa	20	LAM1	Lisboa3	4.3.4.2	NC	MDR	R	R	R	R	R	S	S	S	S	R	889.48	99.39
TB211_15	2015	Lisboa	53	T1	Other	4.1	NC	MDR	R	R	R	S	S	S	S	S	S	R	581.96	99.79
TB22_11	2011	Lisboa	20	LAM1	Lisboa3	4.3.4.2	NC	Other	R	S	R	R	S	ND	S	S	S	R	153.92	98.81
TB23_10	2010	Lisboa	1757		Other	4.3.3	NC	MDR	R	R	R	R	R	R	R	R	S	R	365.64	99.49
TB24_08	2008	Lisboa	20	LAM1	Lisboa3	4.3.4.2	NC	XDR	R	R	R	S	S	S	S	R	R	R	213.99	98.69
TB25_09	2009	Lisboa	81	LAM9	Other	4.3.4.2	NC	Other	R	S	S	S	S	ND	ND	ND	ND	ND	51.75	98.73
TB26_10	2010	Lisboa	1106	LAM4	Q1	4.3.4.2	CC67	MDR	R	R	R	R	R	R	R	R	S	R	438.67	98.85
TB27_04	2004	Lisboa	Orphan		Other	4.8	NC	MDR	R	R	S	R	R	ND	ND	ND	ND	ND	488.27	99.83
TB28_11	2011	Viseu	20	LAM1	Lisboa3	4.3.4.2	CC141	XDR	R	R	R	S	R	R	R	R	R	R	100.01	98.18
TB29_11	2011	Lisboa	1106	LAM4	Q1	4.3.4.2	CC67	XDR	R	R	R	R	R	R	R	R	R	R	131.09	98.75
TB3_09	2009	Porto	47	H1	Other	4.1.2.1	NC	MDR	R	R	S	S	S	S	S	S	S	S	474.65	99.66
TB30_11	2011	Setubal	20	LAM1	Lisboa3	4.3.4.2	CC8	XDR	R	R	R	R	R	ND	R	R	R	R	126.72	98.77
TB31_09	2009	Setubal	20	LAM1	Lisboa3	4.3.4.2	CC7	MDR	R	R	R	S	S	ND	S	S	S	R	123.73	98.81
TB32_07	2007	Lisboa	137	X2	Other	4.1.1.1	NC	MDR	R	R	R	S	S	ND	S	S	S	R	120.78	99.15
TB33_07	2007	Lisboa	119	X1	Other	4.1.1.3	NC	MDR	R	R	S	S	S	ND	ND	ND	ND	ND	677.07	99.76
TB34_08	2008	Lisboa	1	BEIJING	Other	2.2.1	NC	XDR	R	R	R	S	R	R	R	S	R	S	236.69	99.23
TB35_08	2008	Porto	20	LAM1	Lisboa3	4.3.4.2	CC8	XDR	R	R	R	R	R	R	S	R	R	R	228.32	98.77
TB36_09	2009	Lisboa	20	LAM1	Lisboa3	4.3.4.2	NC	XDR	R	R	R	R	R	ND	S	R	R	R	59.12	98.66
TB37_08	2008	Lisboa	20	LAM1	Lisboa3	4.3.4.2	NC	XDR	R	R	R	R	R	R	S	R	R	R	189.51	98.80
TB38_10	2010	Lisboa	20	LAM1	Lisboa3	4.3.4.2	CC53	Other	R	S	R	R	S	S	S	S	S	R	405.69	98.84
TB39_08	2008	Lisboa	105		Other	4.3.2	NC	Susceptible	S	S	S	S	S	ND	ND	ND	ND	ND	300.55	99.71
TB4_06	2006	Porto	47	H1	Other	4.1.2.1	NC	MDR	R	R	S	S	R	S	S	S	R		477.46	99.82
TB40_11	2011	Porto	20	LAM1	Other	4.3.4.1	NC	MDR	R	R	S	S	R	S	S	S	S	S	126.94	98.47
TB41_10	2010	Porto	1	BEIJING	Other	2.2.1	NC	MDR	R	R	R	R	R	S	S	S	S	R	353.60	99.28
TB42_11	2011	Lisboa	1	BEIJING	Other	2.2.1	NC	MDR	R	R	R	R	R	ND	R	R	S	R	170.46	99.32
TB43_08	2008	Lisboa	42	LAM9	Other	4.3.4.2	NC	MDR	R	R	R	R	R	S	S	S	R	R	133.37	98.83
TB44_09	2009	Setubal	34	S	Other	4.4.1.1	NC	Susceptible	S	S	S	S	S	S	S	S	S	S	439.69	99.25
TB45_10	2010	Coimbra	20	LAM1	Lisboa3	4.3.4.2	CC8	XDR	R	R	R	R	S	R	S	S	R	R	788.52	98.82
TB46_09	2009	Lisboa	1	BEIJING	Other	2.2.1	NC	Other	R	S	R	S	S	ND	ND	ND	ND	ND	91.92	99.25
TB47_08	2008	Lisboa	211	LAM3	Other	4.3.2	NC	Other	R	S	S	S	S	ND	ND	ND	ND	ND	41.48	98.71
TB48_13	2013	Lisboa	1106	LAM4	Q1	4.3.4.2	CC67	MDR	R	R	R	R	R	ND	ND	ND	ND	ND	821.72	99.37
TB49_09	2009	Lisboa	20	LAM1	Lisboa3	4.3.4.2	CC8	XDR	R	R	R	R	R	R	S	R	R	R	465.04	98.76
TB5_10	2010	Lisboa	1106	LAM4	Q1	4.3.4.2	CC67	MDR	R	R	R	R	R	R	R	R	S	R	384.10	98.85
TB50_09	2009	Lisboa	20	LAM1	Lisboa3	4.3.4.2	NC	MDR	R	R	R	R	R	S	S	S	R	R	403.64	98.76
TB51_08	2008	Lisboa	20	LAM1	Lisboa3	4.3.4.2	CC8	XDR	R	R	R	R	R	ND	S	R	R	R	125.85	98.69
TB52_11	2011	Setubal	42	LAM9	Other	4.3.4.2	NC	Other	R	S	S	S	S	ND	ND	ND	ND	ND	94.15	98.61
TB53_09	2009	Lisboa	1752	LAM1	Other	4.3.4.1	NC	Other	S	S	R	S	S	ND	ND	ND	ND	ND	520.06	98.84
TB54_09	2009	Setubal	20	LAM1	Lisboa3	4.3.4.2	CC8	XDR	R	R	R	R	R	ND	R	R	R	R	61.50	98.72
TB55_09	2009	Ponta Delgada	20	LAM1	Lisboa3	4.3.4.2	NC	XDR	R	R	R	R	R	R	S	S	R	R	404.10	98.85
TB56_11	2011	Braga	47	H1	Other	4.1.2.1	NC	MDR	R	R	R	R	R	ND	S	S	S	S	83.47	99.58
TB57_08	2008	Lisboa	20	LAM1	Lisboa3	4.3.4.2	CC5	XDR	R	R	R	R	R	R	R	R	R	R	60.09	98.05
TB58_09	2009	Lisboa	53	T1	Other	4.8	NC	Other	R	S	S	S	S	ND	ND	ND	ND	ND	65.25	99.49
TB59_11	2011	Lisboa	53	T1	Other	4.7	NC	MDR	R	R	S	S	R	ND	S	S	S	S	138.88	99.69
TB6_09	2009	Lisboa	20	LAM1	Other	4.3.4.1	NC	Other	R	S	S	S	S	ND	ND	ND	ND	ND	374.64	98.96
TB60_09	2009	Lisboa	20	LAM1	Other	4.3.4.1	NC	Other	R	S	R	S	S	ND	ND	ND	ND	ND	122.30	98.76
TB61_10	2010	Lisboa	53	T1	Other	4.1.2.1	NC	MDR	R	R	S	R	S	ND	ND	ND	ND	ND	175.21	99.55
TB62_10	2010	Faro	1	BEIJING	Other	2.2.1	NC	MDR	R	R	R	S	R	S	S	S	S	R	325.24	99.21
TB63_08	2008	Lisboa	1	BEIJING	Other	2.2.1	CC30	MDR	R	R	R	R	R	ND	ND	ND	ND	ND	91.29	99.22
TB64_08	2008	Lisboa	64	LAM6	Other	4.3.3	NC	Other	R	S	S	S	S	ND	ND	ND	ND	ND	54.50	99.34
TB65_11	2011	Vila Real	53	T1	Other	4.8	NC	MDR	R	R	R	R	R	S	S	S	S	S	115.04	98.96
TB66_15	2015	Lisboa	20	LAM1	Lisboa3	4.3.4.2	NC	MDR	R	R	R	R	R	ND	ND	ND	ND	ND	1284.92	99.65
TB67_08	2008	Lisboa	2535		Other	4.3.4.2	NC	Other	R	S	S	S	S	ND	ND	ND	ND	ND	152.71	98.81
TB68_09	2009	Viseu	20	LAM1	Lisboa3	4.3.4.2	NC	MDR	R	R	R	S	R	S	S	S	S	R	452.38	98.87
TB69_09	2009	Viana do castelo	20	LAM1	Lisboa3	4.3.4.2	CC90	MDR	R	R	R	S	S	S	S	S	S	R	235.66	98.81
TB7_08	2008	Lisboa	1106	LAM4	Q1	4.3.4.2	CC31	MDR	R	R	S	S	R	ND	ND	ND	ND	ND	212.71	98.83
TB70_11	2011	Braganca	53	T1	Other	4.8	NC	MDR	R	R	R	R	R	ND	S	S	S	S	158.47	99.79
TB71_11	2011	Lisboa	1106	LAM4	Q1	4.3.4.2	CC67	MDR	R	R	R	R	R	R	R	R	S	R	148.60	98.36
TB72_08	2008	Funchal	20	LAM1	Lisboa3	4.3.4.2	NC	MDR	R	R	R	S	S	S	S	S	R	R	186.32	97.87
TB73_09	2009	Setubal	20	LAM1	Lisboa3	4.3.4.2	NC	Other	R	S	R	S	ND	S	S	S	S	R	494.75	98.84
TB74_09	2009	Lisboa	211	LAM3	Other	4.3.2	NC	Other	R	S	S	S	S	ND	ND	ND	ND	ND	104.11	99.45
TB75_08	2008	Lisboa	20	LAM1	Lisboa3	4.3.4.2	NC	MDR	R	R	R	R	S	S	S	S	R	R	92.31	98.58
TB76_08	2008	Lisboa	317	T2	Other	4.6.1.2	NC	MDR	R	R	R	R	R	S	S	S	S	S	143.76	99.01
TB77_09	2009	Setubal	20	LAM1	Lisboa3	4.3.4.2	NC	XDR	R	R	R	S	R	ND	R	R	R	R	202.85	98.64
TB78_08	2008	Santarem	2258		Other	4.8	NC	MDR	R	R	S	S	R	ND	S	S	R	R	54.05	99.72
TB79_09	2009	Ponta Delgada	20	LAM1	Lisboa3	4.3.4.2	CC5	XDR	R	R	R	S	R	R	S	S	R	R	192.64	98.86
TB8_11	2011	Ponta Delgada	20	LAM1	Other	4.3.4.1	NC	XDR	R	R	S	R	R	R	R	R	R	S	58.80	98.65
TB80_08	2008	Lisboa	20	LAM1	Lisboa3	4.3.4.2	CC82	XDR	R	R	R	S	R	R	S	S	R	R	85.43	98.33
TB81_09	2009	Porto	92	X3	Other	4.1.1.3	NC	MDR	R	R	R	S	S	S	S	S	S	S	360.01	99.49
TB82_11	2011	Lisboa	59	LAM11-ZWE	Other	4.3.4.2.1	CC52	MDR	R	R	S	S	S	ND	S	S	S	S	135.33	98.07
TB83_09	2009	Lisboa	20	LAM1	Other	4.3.4.1	NC	MDR	R	R	S	R	R	ND	S	S	R	S	117.51	98.88
TB84_10	2010	Ponta Delgada	20	LAM1	Lisboa3	4.3.4.2	CC5	XDR	R	R	R	S	R	R	S	S	R	R	503.63	98.91
TB85_09	2009	Lisboa	20	LAM1	Lisboa3	4.3.4.2	CC5	XDR	R	R	R	R	R	R	S	S	R	R	438.70	98.97
TB86_11	2011	Lisboa	20	LAM1	Lisboa3	4.3.4.2	CC5	XDR	R	R	R	R	R	R	S	S	R	R	94.25	98.70
TB87_08	2008	Setubal	20	LAM1	Lisboa3	4.3.4.2	CC8	MDR	R	R	R	R	R	ND	S	S	R	R	57.49	98.84
TB88_09	2009	Lisboa	20	LAM1	Lisboa3	4.3.4.2	NC	Other	R	S	R	S	S	ND	ND	ND	ND	ND	220.20	98.83
TB89_09	2009	Setubal	20	LAM1	Lisboa3	4.3.4.2	NC	Other	R	S	R	S	S	S	S	S	S	R	590.28	98.86
TB9_08	2008	Lisboa	34	S	Other	4.4.1.1	CC119	Other	R	S	R	S	R	S	S	S	S	R	139.49	98.78
TB90_10	2010	Lisboa	20	LAM1	Lisboa3	4.3.4.2	CC7	MDR	R	R	R	S	S	ND	S	S	S	R	115.51	98.82
TB91_11	2011	Lisboa	20	LAM1	Other	4.3.4.1	NC	Other	R	S	S	S	S	S	S	S	S	S	185.31	99.56
TB92_08	2008	Lisboa	42	LAM9	Other	4.3.4.2	NC	MDR	R	R	R	S	R	ND	S	S	S	S	72.03	98.77
TB93_08	2008	Evora	1	BEIJING	Other	2.2.1	NC	MDR	R	R	R	S	R	ND	ND	ND	ND	ND	107.61	98.68
TB94_07	2007	Lisboa	1106	LAM4	Q1	4.3.4.2	CC160	MDR	R	R	R	R	R	ND	S	S	S	R	346.39	98.72
TB95_10	2010	Porto	41	LAM7-TUR	Other	4.2.2.1	NC	MDR	R	R	R	S	S	S	S	S	S	S	430.30	99.71
TB96_11	2011	Lisboa	1	BEIJING	Other	2.2.1	NC	Other	R	S	R	R	S	S	S	S	S	S	120.22	99.12
TB97_08	2008	Lisboa	20	LAM1	Lisboa3	4.3.4.2	CC8	XDR	R	R	R	R	R	R	S	R	R	R	152.70	98.66
TB98_10	2010	Braga	20	LAM1	Lisboa3	4.3.4.2	NC	MDR	R	R	S	S	S	S	S	S	S	R	472.27	98.91
TB99_11	2011	Lisboa	1106	LAM4	Q1	4.3.4.2	NC	MDR	R	R	R	R	R	S	S	S	R	R	120.81	98.18

[a] Mapping statistics relative to *M. tuberculosis* H37Rv (GenBank Accession NC000962.3)
