# Supplementary material for: Using genomics to understand the origin and dispersion of multidrug and extensively drug resistant tuberculosis in Portugal
Source: Sci Rep. 2020 Feb 13;10:2600. doi: 10.1038/s41598-020-59558-3 (PMC7018963; doi:10.1038/s41598-020-59558-3)
Supplement: Supplementary file 3 — Supplementary Information 3. [file 41598_2020_59558_MOESM3_ESM.pdf]

Supplementary Table S2 - Global dataset screened for the presence of Lisboa3 and Q1 isolates using the specific SNP barcode. NA - Not available.

ENA Run Accession	Country of Origin
DRR014508	NA
DRR019435	Japan
DRR019436	Japan
DRR019437	Japan
DRR019438	Japan
DRR019439	Japan
DRR019440	Japan
DRR019441	Japan
DRR019442	Japan
DRR019443	Japan
DRR019444	Japan
DRR041783	NA
DRR041784	NA
DRR041785	NA
DRR041786	NA
DRR041787	NA
DRR041788	NA
DRR041789	NA
DRR122094	Japan
DRR122095	Japan
DRR122096	Japan
ERR017777	NA
ERR017779	United Kingdom
ERR017781	NA
ERR017782	United Kingdom
ERR017783	Russia
ERR017784	Russia
ERR017785	Russia
ERR017786	Russia
ERR017787	United Kingdom
ERR017788	Estonia
ERR017789	United Kingdom
ERR017790	NA
ERR017791	Russia
ERR017793	Russia
ERR017794	United Kingdom
ERR017795	United Kingdom
ERR017796	United Kingdom
ERR017797	United Kingdom
ERR017799	NA
ERR017800	Russia
ERR017801	United Kingdom
ERR017802	United Kingdom

ERR019550	NA
ERR019551	Russia
ERR019552	Russia
ERR019553	Russia
ERR019554	NA
ERR019555	Russia
ERR019556	Russia
ERR019557	Russia
ERR019558	Russia
ERR019559	Russia
ERR019560	NA
ERR019561	NA
ERR019562	Russia
ERR019563	NA
ERR019564	Russia
ERR019565	United Kingdom
ERR019566	NA
ERR019567	Russia
ERR019568	United Kingdom
ERR019569	United Kingdom
ERR019570	United Kingdom
ERR019571	United Kingdom
ERR019572	Russia
ERR019573	United Kingdom
ERR019574	United Kingdom
ERR019575	Russia
ERR019851	NA
ERR019853	United Kingdom
ERR019855	NA
ERR019856	United Kingdom
ERR019857	Russia
ERR019858	Russia
ERR019859	Russia
ERR019860	Russia
ERR019861	United Kingdom
ERR019862	Estonia
ERR019863	United Kingdom
ERR019864	NA
ERR019865	Russia
ERR019867	Russia
ERR019868	United Kingdom
ERR019869	United Kingdom
ERR019870	United Kingdom
ERR019871	United Kingdom
ERR019873	NA
ERR019874	Russia
ERR019875	United Kingdom
ERR019876	United Kingdom
ERR023245	NA
ERR023246	NA

ERR023247	NA
ERR023248	NA
ERR023249	NA
ERR023250	NA
ERR023251	NA
ERR023252	NA
ERR023253	NA
ERR023254	NA
ERR023255	NA
ERR023256	NA
ERR023257	NA
ERR023258	NA
ERR023259	NA
ERR023260	NA
ERR023261	NA
ERR023728	NA
ERR023729	NA
ERR023730	NA
ERR023731	NA
ERR023732	NA
ERR023733	NA
ERR023734	NA
ERR023735	NA
ERR023736	NA
ERR023737	NA
ERR023738	NA
ERR023739	NA
ERR023740	NA
ERR023741	NA
ERR023742	NA
ERR023743	NA
ERR023744	NA
ERR023745	NA
ERR023746	NA
ERR023747	NA
ERR023748	NA
ERR023749	NA
ERR023750	NA
ERR023751	NA
ERR023752	NA
ERR023753	NA
ERR023754	NA
ERR023755	NA
ERR023756	NA
ERR023757	NA
ERR023758	NA
ERR023759	NA
ERR023760	NA
ERR023761	NA
ERR023762	NA

ERR023763	NA
ERR023764	NA
ERR024336	NA
ERR024337	NA
ERR024338	NA
ERR024339	NA
ERR024340	NA
ERR024341	NA
ERR024342	NA
ERR024343	NA
ERR024344	NA
ERR024345	NA
ERR024346	NA
ERR024347	NA
ERR024348	NA
ERR024349	NA
ERR024350	NA
ERR024351	NA
ERR024352	NA
ERR024353	NA
ERR024354	NA
ERR024355	NA
ERR024356	NA
ERR024357	NA
ERR024358	NA
ERR024359	NA
ERR025413	NA
ERR025414	Netherlands
ERR025415	Netherlands
ERR025416	Netherlands
ERR025417	Netherlands
ERR025418	Netherlands
ERR025419	Netherlands
ERR025420	Netherlands
ERR025421	Netherlands
ERR025422	Netherlands
ERR025423	Netherlands
ERR025424	Netherlands
ERR025425	NA
ERR025426	Netherlands
ERR025427	Netherlands
ERR025428	Netherlands
ERR025429	Netherlands
ERR025430	Netherlands
ERR025431	Netherlands
ERR025432	Netherlands
ERR025433	Netherlands
ERR025434	Netherlands
ERR025435	Netherlands
ERR025436	Netherlands

ERR025437	NA
ERR025438	Netherlands
ERR025439	Netherlands
ERR025440	Netherlands
ERR025441	Netherlands
ERR025442	Netherlands
ERR025443	Netherlands
ERR025444	Netherlands
ERR025445	Netherlands
ERR025446	Netherlands
ERR025447	Netherlands
ERR025448	Netherlands
ERR025449	NA
ERR025450	Netherlands
ERR025451	Netherlands
ERR025452	Netherlands
ERR025453	Netherlands
ERR025454	Netherlands
ERR025455	Netherlands
ERR025456	Netherlands
ERR025457	Netherlands
ERR025458	Netherlands
ERR025459	Netherlands
ERR025460	Netherlands
ERR025832	NA
ERR025833	United Kingdom
ERR025834	United Kingdom
ERR025835	United Kingdom
ERR025836	United Kingdom
ERR025837	United Kingdom
ERR025838	United Kingdom
ERR025839	United Kingdom
ERR025840	United Kingdom
ERR025841	NA
ERR025842	United Kingdom
ERR025843	United Kingdom
ERR025844	United Kingdom
ERR025845	NA
ERR025846	United Kingdom
ERR025847	United Kingdom
ERR025848	United Kingdom
ERR025874	NA
ERR025875	NA
ERR025876	NA
ERR025877	NA
ERR025878	NA
ERR025879	NA
ERR025880	NA
ERR025881	NA
ERR025882	NA

ERR025883	NA
ERR025884	NA
ERR025885	NA
ERR025886	NA
ERR025887	NA
ERR025888	NA
ERR025889	NA
ERR025890	NA
ERR025891	NA
ERR025892	NA
ERR025893	NA
ERR025894	NA
ERR025895	NA
ERR025896	NA
ERR025897	NA
ERR025898	NA
ERR025899	NA
ERR026471	NA
ERR026472	Netherlands
ERR026473	Netherlands
ERR026474	Netherlands
ERR026475	Netherlands
ERR026476	Netherlands
ERR026477	Netherlands
ERR026478	Netherlands
ERR026479	Netherlands
ERR026480	Netherlands
ERR026481	Netherlands
ERR026482	Netherlands
ERR026634	NA
ERR026635	Russia
ERR026637	Russia
ERR026638	United Kingdom
ERR026639	United Kingdom
ERR026640	United Kingdom
ERR026641	United Kingdom
ERR026643	NA
ERR026644	Russia
ERR026645	United Kingdom
ERR026646	United Kingdom
ERR027082	NA
ERR027083	NA
ERR027444	NA
ERR027445	Russia
ERR027446	Russia
ERR027447	Russia
ERR027448	Russia
ERR027449	Russia
ERR027450	Russia
ERR027451	Russia

ERR027452	Estonia
ERR027453	NA
ERR027454	Russia
ERR027455	Russia
ERR027456	Russia
ERR027457	NA
ERR027458	Russia
ERR027459	Russia
ERR027460	NA
ERR027461	NA
ERR027462	United Kingdom
ERR027463	NA
ERR027464	United Kingdom
ERR027465	NA
ERR027466	NA
ERR027467	NA
ERR027468	NA
ERR027469	Russia
ERR028317	NA
ERR028318	NA
ERR028319	NA
ERR028320	NA
ERR028321	NA
ERR028322	NA
ERR028323	NA
ERR028324	NA
ERR028325	NA
ERR028326	NA
ERR028327	NA
ERR028328	NA
ERR028329	NA
ERR028330	NA
ERR028331	NA
ERR028332	NA
ERR028333	NA
ERR028334	NA
ERR028335	NA
ERR028336	NA
ERR028337	NA
ERR028338	NA
ERR028339	NA
ERR028340	NA
ERR028341	NA
ERR028342	NA
ERR028343	NA
ERR028344	NA
ERR028345	NA
ERR028346	NA
ERR028347	NA
ERR028348	NA

ERR028349	NA
ERR028350	NA
ERR028351	NA
ERR028352	NA
ERR028353	NA
ERR028354	NA
ERR028355	NA
ERR028356	NA
ERR028357	NA
ERR028358	NA
ERR028359	NA
ERR028360	NA
ERR028361	NA
ERR028362	NA
ERR028363	NA
ERR028364	NA
ERR028365	NA
ERR028366	NA
ERR028367	NA
ERR028368	NA
ERR028606	NA
ERR028607	Estonia
ERR028608	Netherlands
ERR028609	Estonia
ERR028610	Netherlands
ERR028611	Netherlands
ERR028612	Netherlands
ERR028613	Netherlands
ERR028614	Netherlands
ERR028615	Netherlands
ERR028616	Netherlands
ERR028617	Netherlands
ERR028618	NA
ERR028619	Estonia
ERR028620	Netherlands
ERR028621	Netherlands
ERR028622	Netherlands
ERR028623	Netherlands
ERR028624	Netherlands
ERR028625	Netherlands
ERR028626	Netherlands
ERR028627	Netherlands
ERR028628	Netherlands
ERR028629	Netherlands
ERR029200	NA
ERR029201	Netherlands
ERR029202	Netherlands
ERR029203	South Africa
ERR029204	Netherlands
ERR029205	Netherlands

ERR029206	Netherlands
ERR029207	Netherlands
ERR029208	NA
ERR029209	Estonia
ERR029210	Netherlands
ERR029211	Netherlands
ERR031448	NA
ERR031449	NA
ERR031450	NA
ERR031451	NA
ERR031452	NA
ERR031453	NA
ERR031454	NA
ERR031455	NA
ERR031456	NA
ERR031457	NA
ERR031458	NA
ERR031459	NA
ERR031460	NA
ERR031461	NA
ERR031462	NA
ERR031463	NA
ERR031464	NA
ERR031465	NA
ERR031466	NA
ERR031467	NA
ERR031468	NA
ERR031469	NA
ERR031470	NA
ERR031471	NA
ERR031472	NA
ERR031473	NA
ERR031474	NA
ERR031475	NA
ERR031476	NA
ERR031477	NA
ERR031478	NA
ERR031479	NA
ERR031480	NA
ERR031481	NA
ERR031482	NA
ERR031483	NA
ERR031484	NA
ERR031485	NA
ERR031486	NA
ERR031487	NA
ERR031488	NA
ERR031489	NA
ERR031490	NA
ERR031491	NA

ERR031492	NA
ERR036185	NA
ERR036186	Malawi
ERR036187	Malawi
ERR036188	Malawi
ERR036189	Malawi
ERR036190	Malawi
ERR036191	Malawi
ERR036192	Malawi
ERR036193	Malawi
ERR036194	Malawi
ERR036195	Malawi
ERR036196	Malawi
ERR036197	Malawi
ERR036198	NA
ERR036199	Malawi
ERR036200	Malawi
ERR036201	Malawi
ERR036202	Malawi
ERR036203	Malawi
ERR036204	Malawi
ERR036205	Malawi
ERR036206	Malawi
ERR036207	Malawi
ERR036208	Malawi
ERR036209	Malawi
ERR036210	Malawi
ERR036211	NA
ERR036212	Malawi
ERR036213	Malawi
ERR036214	Malawi
ERR036215	Malawi
ERR036216	Malawi
ERR036217	Malawi
ERR036218	Malawi
ERR036219	Malawi
ERR036220	Malawi
ERR036221	Malawi
ERR036222	Malawi
ERR036223	Malawi
ERR036224	NA
ERR036225	Malawi
ERR036226	Malawi
ERR036227	Malawi
ERR036228	Malawi
ERR036229	Malawi
ERR036230	Malawi
ERR036231	Malawi
ERR036232	Malawi
ERR036233	Malawi

ERR036234	Malawi
ERR036235	Malawi
ERR036236	Malawi
ERR036237	NA
ERR036238	Malawi
ERR036239	Malawi
ERR036240	Malawi
ERR036241	Malawi
ERR036242	Malawi
ERR036243	Malawi
ERR036244	Malawi
ERR036245	Malawi
ERR036246	Malawi
ERR036247	Malawi
ERR036248	Malawi
ERR036249	Malawi
ERR037234	NA
ERR037235	NA
ERR037467	Malawi
ERR037468	Malawi
ERR037469	Malawi
ERR037470	Malawi
ERR037471	Malawi
ERR037472	Malawi
ERR037473	Malawi
ERR037474	Malawi
ERR037475	Malawi
ERR037476	Malawi
ERR037477	Malawi
ERR037478	Malawi
ERR037479	Malawi
ERR037480	Malawi
ERR037481	Malawi
ERR037482	Malawi
ERR037483	Malawi
ERR037484	Malawi
ERR037485	Malawi
ERR037486	Malawi
ERR037487	Malawi
ERR037488	Malawi
ERR037489	Malawi
ERR037490	Malawi
ERR037491	Malawi
ERR037492	Malawi
ERR037493	Malawi
ERR037494	Malawi
ERR037495	Malawi
ERR037496	Malawi
ERR037497	Malawi
ERR037498	Malawi

ERR037499	Malawi
ERR037500	Malawi
ERR037501	Malawi
ERR037502	Malawi
ERR037503	Malawi
ERR037504	Malawi
ERR037505	Malawi
ERR037506	Malawi
ERR037507	Malawi
ERR037508	Malawi
ERR037509	Malawi
ERR037510	Malawi
ERR037511	Malawi
ERR037512	Malawi
ERR037513	Malawi
ERR037514	Malawi
ERR037515	Malawi
ERR037516	Malawi
ERR037517	Malawi
ERR037518	Malawi
ERR037519	Malawi
ERR037520	Malawi
ERR037521	Malawi
ERR037522	Malawi
ERR037523	Malawi
ERR037524	Malawi
ERR037525	Malawi
ERR037526	Malawi
ERR037527	Malawi
ERR037528	Malawi
ERR037529	Malawi
ERR037530	Malawi
ERR037531	Malawi
ERR037532	Malawi
ERR037533	Malawi
ERR037534	Malawi
ERR037535	Malawi
ERR037536	Malawi
ERR037537	Malawi
ERR037538	Malawi
ERR037539	Malawi
ERR037540	Malawi
ERR037541	Malawi
ERR037542	Malawi
ERR037543	Malawi
ERR037544	Malawi
ERR037545	Malawi
ERR037546	Malawi
ERR037547	Malawi
ERR037548	Malawi

ERR037549	Malawi
ERR037550	Malawi
ERR037551	Malawi
ERR037552	Malawi
ERR037553	Malawi
ERR037554	Malawi
ERR037555	Malawi
ERR038253	United Kingdom
ERR038254	United Kingdom
ERR038255	United Kingdom
ERR038256	United Kingdom
ERR038257	United Kingdom
ERR038258	United Kingdom
ERR038259	United Kingdom
ERR038260	United Kingdom
ERR038261	United Kingdom
ERR038262	United Kingdom
ERR038263	United Kingdom
ERR038264	United Kingdom
ERR038265	United Kingdom
ERR038266	United Kingdom
ERR038267	NA
ERR038268	NA
ERR038269	United Kingdom
ERR038270	United Kingdom
ERR038271	United Kingdom
ERR038272	United Kingdom
ERR038273	United Kingdom
ERR038274	United Kingdom
ERR038275	United Kingdom
ERR038276	United Kingdom
ERR038277	United Kingdom
ERR038278	United Kingdom
ERR038279	United Kingdom
ERR038280	United Kingdom
ERR038281	United Kingdom
ERR038282	United Kingdom
ERR038283	United Kingdom
ERR038284	United Kingdom
ERR038285	United Kingdom
ERR038286	United Kingdom
ERR038287	United Kingdom
ERR038288	United Kingdom
ERR038289	NA
ERR038290	United Kingdom
ERR038291	United Kingdom
ERR038292	United Kingdom
ERR038293	United Kingdom
ERR038294	United Kingdom
ERR038295	United Kingdom

ERR038296	United Kingdom
ERR038297	United Kingdom
ERR038298	United Kingdom
ERR038299	United Kingdom
ERR038300	United Kingdom
ERR038736	Uganda
ERR038737	Uganda
ERR038738	Uganda
ERR038739	Uganda
ERR038740	Uganda
ERR038741	Uganda
ERR038742	Uganda
ERR038743	Uganda
ERR038744	Uganda
ERR038745	Uganda
ERR038746	Uganda
ERR038747	Uganda
ERR038748	Uganda
ERR038749	Uganda
ERR038750	Uganda
ERR038751	Uganda
ERR038752	Uganda
ERR038753	Uganda
ERR038754	Uganda
ERR038755	Uganda
ERR039323	United Kingdom
ERR039324	United Kingdom
ERR039325	United Kingdom
ERR039326	United Kingdom
ERR039327	United Kingdom
ERR039328	United Kingdom
ERR039329	United Kingdom
ERR039330	United Kingdom
ERR039331	United Kingdom
ERR039332	United Kingdom
ERR039333	United Kingdom
ERR039334	United Kingdom
ERR039335	United Kingdom
ERR039336	United Kingdom
ERR039337	United Kingdom
ERR039338	United Kingdom
ERR039339	United Kingdom
ERR039340	United Kingdom
ERR039341	United Kingdom
ERR039342	United Kingdom
ERR039343	United Kingdom
ERR039344	United Kingdom
ERR039345	United Kingdom
ERR039346	United Kingdom
ERR040086	United Kingdom

ERR040087	United Kingdom
ERR040088	United Kingdom
ERR040089	United Kingdom
ERR040090	United Kingdom
ERR040091	United Kingdom
ERR040092	NA
ERR040093	United Kingdom
ERR040094	United Kingdom
ERR040095	United Kingdom
ERR040096	United Kingdom
ERR040097	United Kingdom
ERR040098	United Kingdom
ERR040099	United Kingdom
ERR040100	United Kingdom
ERR040101	United Kingdom
ERR040102	United Kingdom
ERR040103	United Kingdom
ERR040104	United Kingdom
ERR040105	United Kingdom
ERR040106	United Kingdom
ERR040107	United Kingdom
ERR040108	United Kingdom
ERR040109	United Kingdom
ERR040112	Uganda
ERR040113	Uganda
ERR040114	Uganda
ERR040115	Uganda
ERR040116	Uganda
ERR040117	Uganda
ERR040118	Uganda
ERR040119	Uganda
ERR040120	Uganda
ERR040121	Uganda
ERR040122	Uganda
ERR040123	Uganda
ERR040124	Uganda
ERR040125	Uganda
ERR040126	Uganda
ERR040127	Uganda
ERR040128	Uganda
ERR040129	Uganda
ERR040130	Uganda
ERR040131	Uganda
ERR040132	Uganda
ERR040133	Uganda
ERR040134	Uganda
ERR040135	Uganda
ERR040136	Uganda
ERR040137	Uganda
ERR040138	Uganda

ERR040139	Uganda
ERR040140	Uganda
ERR040141	Uganda
ERR040142	Uganda
ERR045759	NA
ERR045760	NA
ERR045761	NA
ERR045762	NA
ERR045763	NA
ERR046729	United Kingdom
ERR046730	United Kingdom
ERR046731	NA
ERR046732	United Kingdom
ERR046733	United Kingdom
ERR046734	United Kingdom
ERR046735	United Kingdom
ERR046736	United Kingdom
ERR046737	United Kingdom
ERR046738	United Kingdom
ERR046739	United Kingdom
ERR046740	NA
ERR046741	United Kingdom
ERR046742	NA
ERR046743	United Kingdom
ERR046744	United Kingdom
ERR046745	United Kingdom
ERR046746	United Kingdom
ERR046747	United Kingdom
ERR046748	United Kingdom
ERR046749	United Kingdom
ERR046750	NA
ERR046751	United Kingdom
ERR046752	United Kingdom
ERR046753	United Kingdom
ERR046754	United Kingdom
ERR046755	United Kingdom
ERR046756	United Kingdom
ERR046757	NA
ERR046758	United Kingdom
ERR046759	United Kingdom
ERR046760	United Kingdom
ERR046761	United Kingdom
ERR046762	United Kingdom
ERR046763	United Kingdom
ERR046764	United Kingdom
ERR046765	United Kingdom
ERR046766	United Kingdom
ERR046767	United Kingdom
ERR046768	United Kingdom
ERR046769	United Kingdom

ERR046770	United Kingdom
ERR046771	United Kingdom
ERR046772	United Kingdom
ERR046773	United Kingdom
ERR046774	NA
ERR046775	United Kingdom
ERR046776	United Kingdom
ERR046777	United Kingdom
ERR046778	United Kingdom
ERR046779	United Kingdom
ERR046780	United Kingdom
ERR046781	United Kingdom
ERR046782	United Kingdom
ERR046783	United Kingdom
ERR046784	United Kingdom
ERR046785	United Kingdom
ERR046786	United Kingdom
ERR046787	United Kingdom
ERR046788	United Kingdom
ERR046789	United Kingdom
ERR046790	United Kingdom
ERR046791	United Kingdom
ERR046792	United Kingdom
ERR046793	United Kingdom
ERR046794	United Kingdom
ERR046795	United Kingdom
ERR046796	United Kingdom
ERR046797	United Kingdom
ERR046798	United Kingdom
ERR046799	United Kingdom
ERR046800	United Kingdom
ERR046801	NA
ERR046802	NA
ERR046803	NA
ERR046804	NA
ERR046805	NA
ERR046806	NA
ERR046807	NA
ERR046808	NA
ERR046809	NA
ERR046810	NA
ERR046811	NA
ERR046812	NA
ERR046813	NA
ERR046814	NA
ERR046815	NA
ERR046816	NA
ERR046817	NA
ERR046818	NA
ERR046819	United Kingdom

ERR046820	United Kingdom
ERR046821	United Kingdom
ERR046822	United Kingdom
ERR046823	United Kingdom
ERR046824	United Kingdom
ERR046825	United Kingdom
ERR046826	NA
ERR046827	NA
ERR046828	NA
ERR046829	NA
ERR046830	NA
ERR046831	United Kingdom
ERR046832	United Kingdom
ERR046833	United Kingdom
ERR046834	United Kingdom
ERR046835	NA
ERR046836	United Kingdom
ERR046837	United Kingdom
ERR046838	United Kingdom
ERR046839	United Kingdom
ERR046840	United Kingdom
ERR046841	United Kingdom
ERR046842	United Kingdom
ERR046843	United Kingdom
ERR046844	United Kingdom
ERR046845	United Kingdom
ERR046846	United Kingdom
ERR046847	United Kingdom
ERR046848	United Kingdom
ERR046849	United Kingdom
ERR046850	United Kingdom
ERR046851	United Kingdom
ERR046852	United Kingdom
ERR046853	United Kingdom
ERR046854	United Kingdom
ERR046855	United Kingdom
ERR046856	United Kingdom
ERR046857	United Kingdom
ERR046858	United Kingdom
ERR046859	United Kingdom
ERR046860	United Kingdom
ERR046861	United Kingdom
ERR046862	United Kingdom
ERR046863	United Kingdom
ERR046864	United Kingdom
ERR046865	United Kingdom
ERR046866	United Kingdom
ERR046867	United Kingdom
ERR046868	United Kingdom
ERR046869	United Kingdom

ERR046870	United Kingdom
ERR046871	United Kingdom
ERR046872	United Kingdom
ERR046873	United Kingdom
ERR046874	United Kingdom
ERR046875	United Kingdom
ERR046876	United Kingdom
ERR046877	United Kingdom
ERR046878	United Kingdom
ERR046879	United Kingdom
ERR046880	United Kingdom
ERR046881	United Kingdom
ERR046882	United Kingdom
ERR046883	United Kingdom
ERR046884	United Kingdom
ERR046885	United Kingdom
ERR046886	NA
ERR046887	United Kingdom
ERR046888	United Kingdom
ERR046889	United Kingdom
ERR046890	United Kingdom
ERR046891	United Kingdom
ERR046892	United Kingdom
ERR046893	United Kingdom
ERR046894	United Kingdom
ERR046895	United Kingdom
ERR046896	NA
ERR046897	United Kingdom
ERR046898	United Kingdom
ERR046899	NA
ERR046900	United Kingdom
ERR046901	United Kingdom
ERR046902	NA
ERR046903	United Kingdom
ERR046904	United Kingdom
ERR046905	United Kingdom
ERR046906	United Kingdom
ERR046907	United Kingdom
ERR046908	United Kingdom
ERR046909	NA
ERR046910	United Kingdom
ERR046911	United Kingdom
ERR046912	United Kingdom
ERR046913	United Kingdom
ERR046914	United Kingdom
ERR046915	United Kingdom
ERR046916	United Kingdom
ERR046917	United Kingdom
ERR046918	United Kingdom
ERR046919	United Kingdom

ERR046920	United Kingdom
ERR046921	United Kingdom
ERR046922	United Kingdom
ERR046923	United Kingdom
ERR046924	United Kingdom
ERR046925	United Kingdom
ERR046926	United Kingdom
ERR046927	United Kingdom
ERR046928	United Kingdom
ERR046929	United Kingdom
ERR046930	United Kingdom
ERR046931	NA
ERR046932	United Kingdom
ERR046933	United Kingdom
ERR046934	United Kingdom
ERR046935	United Kingdom
ERR046936	United Kingdom
ERR046937	United Kingdom
ERR046938	United Kingdom
ERR046939	United Kingdom
ERR046940	United Kingdom
ERR046941	United Kingdom
ERR046942	United Kingdom
ERR046943	United Kingdom
ERR046944	NA
ERR046945	United Kingdom
ERR046946	United Kingdom
ERR046947	United Kingdom
ERR046948	United Kingdom
ERR046949	United Kingdom
ERR046950	United Kingdom
ERR046951	United Kingdom
ERR046952	United Kingdom
ERR046953	United Kingdom
ERR046954	United Kingdom
ERR046955	NA
ERR046956	NA
ERR046957	United Kingdom
ERR046958	United Kingdom
ERR046959	United Kingdom
ERR046960	United Kingdom
ERR046961	United Kingdom
ERR046962	United Kingdom
ERR046963	United Kingdom
ERR046964	United Kingdom
ERR046965	United Kingdom
ERR046966	United Kingdom
ERR046967	United Kingdom
ERR046968	United Kingdom
ERR046969	United Kingdom

ERR046970	United Kingdom
ERR046971	United Kingdom
ERR046972	United Kingdom
ERR046973	NA
ERR046974	United Kingdom
ERR046975	United Kingdom
ERR046976	NA
ERR046977	NA
ERR046978	NA
ERR046979	NA
ERR046980	United Kingdom
ERR046981	United Kingdom
ERR046982	United Kingdom
ERR046983	United Kingdom
ERR046984	United Kingdom
ERR046985	NA
ERR046986	United Kingdom
ERR046987	NA
ERR046988	United Kingdom
ERR046989	United Kingdom
ERR046990	United Kingdom
ERR046991	United Kingdom
ERR046992	United Kingdom
ERR046993	United Kingdom
ERR046994	United Kingdom
ERR046995	United Kingdom
ERR046996	United Kingdom
ERR046997	United Kingdom
ERR046998	United Kingdom
ERR046999	United Kingdom
ERR047000	United Kingdom
ERR047001	United Kingdom
ERR047002	United Kingdom
ERR047003	United Kingdom
ERR047004	United Kingdom
ERR047005	United Kingdom
ERR047006	United Kingdom
ERR047007	United Kingdom
ERR047008	United Kingdom
ERR047009	United Kingdom
ERR047010	United Kingdom
ERR047011	United Kingdom
ERR047012	United Kingdom
ERR047013	United Kingdom
ERR047014	United Kingdom
ERR047015	NA
ERR047016	United Kingdom
ERR047880	NA
ERR047881	United Kingdom
ERR047882	NA

ERR047883	NA
ERR047884	United Kingdom
ERR047885	United Kingdom
ERR047886	United Kingdom
ERR047887	NA
ERR047888	NA
ERR047889	NA
ERR047890	NA
ERR047891	NA
ERR067576	Russia
ERR067577	Russia
ERR067578	Russia
ERR067579	Russia
ERR067580	Russia
ERR067581	Russia
ERR067582	Russia
ERR067583	Russia
ERR067584	Russia
ERR067585	Russia
ERR067586	Russia
ERR067587	Russia
ERR067588	Russia
ERR067589	Russia
ERR067590	Russia
ERR067591	Russia
ERR067592	Russia
ERR067593	Russia
ERR067594	Russia
ERR067595	Russia
ERR067596	Russia
ERR067597	Russia
ERR067598	Russia
ERR067599	Russia
ERR067600	Russia
ERR067601	Russia
ERR067602	Russia
ERR067603	Russia
ERR067604	Russia
ERR067605	Russia
ERR067606	Russia
ERR067607	Russia
ERR067608	Russia
ERR067609	Russia
ERR067610	Russia
ERR067611	Russia
ERR067612	Russia
ERR067613	Russia
ERR067614	Russia
ERR067615	Russia
ERR067616	Russia

ERR067617	Russia
ERR067618	Russia
ERR067619	Russia
ERR067620	Russia
ERR067621	Russia
ERR067622	Russia
ERR067623	Russia
ERR067624	Russia
ERR067625	Russia
ERR067626	Russia
ERR067627	Russia
ERR067628	NA
ERR067629	Russia
ERR067630	Russia
ERR067631	Russia
ERR067632	Russia
ERR067633	NA
ERR067634	Russia
ERR067635	Russia
ERR067636	Russia
ERR067637	Russia
ERR067638	Russia
ERR067639	Russia
ERR067640	Russia
ERR067641	Russia
ERR067642	NA
ERR067643	Russia
ERR067644	Russia
ERR067645	Russia
ERR067646	Russia
ERR067647	Russia
ERR067648	Russia
ERR067649	Russia
ERR067650	Russia
ERR067651	Russia
ERR067652	Russia
ERR067653	Russia
ERR067654	Russia
ERR067655	Russia
ERR067656	Russia
ERR067657	Russia
ERR067658	Russia
ERR067659	Russia
ERR067660	Russia
ERR067661	Russia
ERR067662	Russia
ERR067663	Russia
ERR067664	Russia
ERR067665	Russia
ERR067666	Russia

ERR067667	Russia
ERR067668	Russia
ERR067669	NA
ERR067670	Russia
ERR067671	Russia
ERR067672	Russia
ERR067673	Russia
ERR067674	Russia
ERR067675	Russia
ERR067676	Russia
ERR067677	Russia
ERR067678	Russia
ERR067679	Russia
ERR067680	Russia
ERR067681	NA
ERR067682	Russia
ERR067683	Russia
ERR067684	Russia
ERR067685	Russia
ERR067686	Russia
ERR067687	Russia
ERR067688	Russia
ERR067689	Russia
ERR067690	Russia
ERR067691	Russia
ERR067692	Russia
ERR067693	Russia
ERR067694	Russia
ERR067695	Russia
ERR067696	Russia
ERR067697	Russia
ERR067698	Russia
ERR067699	NA
ERR067700	Russia
ERR067701	NA
ERR067702	NA
ERR067703	Russia
ERR067704	Russia
ERR067705	Russia
ERR067706	Russia
ERR067707	Russia
ERR067708	Russia
ERR067709	Russia
ERR067710	Russia
ERR067711	Russia
ERR067712	NA
ERR067713	Russia
ERR067714	Russia
ERR067715	Russia
ERR067716	Russia

ERR067717	Russia
ERR067718	Russia
ERR067719	Russia
ERR067720	Russia
ERR067721	Russia
ERR067722	Russia
ERR067723	Russia
ERR067724	Russia
ERR067725	Russia
ERR067726	Russia
ERR067727	Russia
ERR067728	Russia
ERR067729	Russia
ERR067730	Russia
ERR067731	Russia
ERR067732	Russia
ERR067733	Russia
ERR067734	Russia
ERR067735	Russia
ERR067736	Russia
ERR067737	Russia
ERR067738	NA
ERR067739	Russia
ERR067740	Russia
ERR067741	Russia
ERR067742	Russia
ERR067743	Russia
ERR067744	Russia
ERR067745	Russia
ERR067746	Russia
ERR067747	Russia
ERR067748	Russia
ERR067749	NA
ERR067750	Russia
ERR067751	Russia
ERR067752	Russia
ERR067753	Russia
ERR067754	Russia
ERR067755	Russia
ERR067756	Russia
ERR067757	Russia
ERR067758	Russia
ERR067759	Russia
ERR067760	Russia
ERR067761	Russia
ERR067762	Russia
ERR067763	Russia
ERR067764	NA
ERR067765	Russia
ERR067766	Russia

ERR067767	Russia
ERR072013	NA
ERR072014	NA
ERR072015	NA
ERR072016	NA
ERR072017	NA
ERR072018	NA
ERR072019	United Kingdom
ERR072020	United Kingdom
ERR072021	United Kingdom
ERR072022	United Kingdom
ERR072023	United Kingdom
ERR072024	United Kingdom
ERR072025	United Kingdom
ERR072026	United Kingdom
ERR072027	United Kingdom
ERR072028	United Kingdom
ERR072029	United Kingdom
ERR072030	United Kingdom
ERR072031	United Kingdom
ERR072032	United Kingdom
ERR072033	NA
ERR072034	United Kingdom
ERR072035	United Kingdom
ERR072036	United Kingdom
ERR072037	United Kingdom
ERR072038	United Kingdom
ERR072039	United Kingdom
ERR072040	United Kingdom
ERR072041	United Kingdom
ERR072042	United Kingdom
ERR072043	NA
ERR072044	United Kingdom
ERR072045	United Kingdom
ERR072046	United Kingdom
ERR072047	United Kingdom
ERR072048	United Kingdom
ERR072049	NA
ERR072050	United Kingdom
ERR072051	United Kingdom
ERR072052	NA
ERR072053	NA
ERR072054	NA
ERR072055	NA
ERR072056	NA
ERR072057	NA
ERR072058	NA
ERR072059	NA
ERR072060	NA
ERR072061	NA

ERR072062	NA
ERR072063	NA
ERR072064	NA
ERR072065	United Kingdom
ERR072066	NA
ERR072067	NA
ERR072068	NA
ERR072069	NA
ERR072070	NA
ERR072071	NA
ERR072072	United Kingdom
ERR072073	NA
ERR072074	NA
ERR072075	NA
ERR072076	NA
ERR072077	United Kingdom
ERR072078	NA
ERR072079	NA
ERR072080	United Kingdom
ERR072081	NA
ERR072082	NA
ERR072083	NA
ERR072084	NA
ERR072085	NA
ERR072086	NA
ERR072087	United Kingdom
ERR072088	United Kingdom
ERR072089	United Kingdom
ERR072090	United Kingdom
ERR072091	NA
ERR072092	NA
ERR072093	NA
ERR072094	United Kingdom
ERR072095	United Kingdom
ERR072096	United Kingdom
ERR072097	NA
ERR072098	NA
ERR072099	NA
ERR072100	NA
ERR072101	NA
ERR072102	NA
ERR072103	NA
ERR072104	NA
ERR072105	NA
ERR072106	NA
ERR072107	NA
ERR072108	NA
ERR1007898	NA
ERR1013702	NA
ERR1013703	NA

ERR1013704	NA
ERR1023294	NA
ERR1023295	NA
ERR1023296	NA
ERR1023297	NA
ERR1023298	NA
ERR1023299	NA
ERR1023300	NA
ERR1023301	NA
ERR1023302	NA
ERR1023303	NA
ERR1023304	NA
ERR1023305	NA
ERR1023306	NA
ERR1023307	NA
ERR1023308	NA
ERR1023309	NA
ERR1023310	NA
ERR1023311	NA
ERR1023312	NA
ERR1023313	NA
ERR1023314	NA
ERR1023315	NA
ERR1023316	NA
ERR1023317	NA
ERR1023318	NA
ERR1023319	NA
ERR1023320	NA
ERR1023321	NA
ERR1023322	NA
ERR1023323	NA
ERR1023324	NA
ERR1023325	NA
ERR1023326	NA
ERR1023327	NA
ERR1023328	NA
ERR1023329	NA
ERR1023330	NA
ERR1023331	NA
ERR1023332	NA
ERR1023333	NA
ERR1023334	NA
ERR1023335	NA
ERR1023336	NA
ERR1023337	NA
ERR1023338	NA
ERR1023339	NA
ERR1023340	NA
ERR1023341	NA
ERR1023342	NA

ERR1023343	NA
ERR1023344	NA
ERR1023345	NA
ERR1023346	NA
ERR1023347	NA
ERR1023348	NA
ERR1023349	NA
ERR1023350	NA
ERR1023351	NA
ERR1023352	NA
ERR1023353	NA
ERR1023354	NA
ERR1023355	NA
ERR1023356	NA
ERR1023357	NA
ERR1023358	NA
ERR1023359	NA
ERR1023360	NA
ERR1023361	NA
ERR1023362	NA
ERR1023363	NA
ERR1023364	NA
ERR1023365	NA
ERR1023366	NA
ERR1023367	NA
ERR1023368	NA
ERR1023369	NA
ERR1023370	NA
ERR1023371	NA
ERR1023372	NA
ERR1023373	NA
ERR1023374	NA
ERR1023375	NA
ERR1023376	NA
ERR1023377	NA
ERR1023378	NA
ERR1023379	NA
ERR1023380	NA
ERR1023381	NA
ERR1023382	NA
ERR1023383	NA
ERR1023384	NA
ERR1023385	NA
ERR1023386	NA
ERR1023387	NA
ERR1023388	Sweden
ERR1023389	NA
ERR1023390	NA
ERR1023391	NA
ERR1023392	NA

ERR1023393	NA
ERR1023394	NA
ERR1023395	NA
ERR1023396	NA
ERR1023397	NA
ERR1023398	NA
ERR1023399	NA
ERR1023400	NA
ERR1023401	NA
ERR1023402	NA
ERR1023403	NA
ERR1023404	NA
ERR1023405	NA
ERR1023406	NA
ERR1023407	NA
ERR1023408	NA
ERR1023409	NA
ERR1023410	NA
ERR1023411	NA
ERR1023412	NA
ERR1023413	NA
ERR1023414	NA
ERR1023415	NA
ERR1023416	NA
ERR1023417	NA
ERR1023418	NA
ERR1023419	NA
ERR1023420	NA
ERR1023421	NA
ERR1023422	NA
ERR1023423	NA
ERR1023424	NA
ERR1023425	NA
ERR1023426	NA
ERR1023427	NA
ERR1023428	NA
ERR1023429	NA
ERR1023430	NA
ERR1023431	NA
ERR1023432	NA
ERR1023433	NA
ERR1023434	NA
ERR1023435	NA
ERR1023436	NA
ERR1023437	NA
ERR1023438	NA
ERR1023439	NA
ERR1023440	NA
ERR1023441	NA
ERR1023442	NA

ERR1023443	NA
ERR1023444	NA
ERR1023445	NA
ERR1023446	NA
ERR1023447	NA
ERR1023448	NA
ERR1023449	NA
ERR1023450	NA
ERR1023451	NA
ERR1023452	NA
ERR1023453	NA
ERR1023454	NA
ERR1023455	NA
ERR1023456	NA
ERR1023457	NA
ERR1023458	NA
ERR1023459	NA
ERR1023460	NA
ERR1023461	NA
ERR1023462	NA
ERR1023463	NA
ERR1023464	NA
ERR1023465	NA
ERR1023466	NA
ERR1023467	NA
ERR1023468	NA
ERR1023469	NA
ERR1023470	NA
ERR1023471	NA
ERR1023472	NA
ERR1023473	NA
ERR1023474	NA
ERR1023475	NA
ERR1023476	NA
ERR1023477	NA
ERR1023478	NA
ERR1023479	NA
ERR1023480	NA
ERR1023481	NA
ERR1023482	NA
ERR1023483	NA
ERR1023484	NA
ERR1023485	NA
ERR1023486	NA
ERR1023487	NA
ERR1023488	NA
ERR1023489	NA
ERR1023490	NA
ERR1023491	NA
ERR1023492	NA

ERR1023493	NA
ERR1023494	NA
ERR1023495	NA
ERR1023496	NA
ERR1023497	NA
ERR1023498	NA
ERR1023499	NA
ERR1023500	NA
ERR1023501	NA
ERR1023502	NA
ERR1023503	NA
ERR1023504	NA
ERR1023505	NA
ERR1023506	NA
ERR1023507	NA
ERR1023508	NA
ERR1023509	NA
ERR1023510	NA
ERR1023511	NA
ERR1023512	NA
ERR1023513	NA
ERR1023514	NA
ERR1023515	NA
ERR1023516	NA
ERR1023517	NA
ERR1023518	NA
ERR1023519	NA
ERR1023520	NA
ERR1023521	NA
ERR1023522	NA
ERR1023523	NA
ERR1023524	NA
ERR1023525	NA
ERR1023526	NA
ERR1023527	NA
ERR1023528	NA
ERR1023529	NA
ERR1023530	NA
ERR1023531	NA
ERR1034576	NA
ERR1034577	NA
ERR1034578	NA
ERR1034579	NA
ERR1034580	NA
ERR1034581	NA
ERR1034582	NA
ERR1034583	NA
ERR1034584	NA
ERR1034585	NA
ERR1034586	NA

ERR1034587	NA
ERR1034588	NA
ERR1034589	NA
ERR1034590	NA
ERR1034591	NA
ERR1034592	NA
ERR1034593	NA
ERR1034594	NA
ERR1034595	NA
ERR1034596	NA
ERR1034597	NA
ERR1034598	NA
ERR1034599	NA
ERR1034600	NA
ERR1034601	NA
ERR1034602	NA
ERR1034603	NA
ERR1034604	NA
ERR1034605	NA
ERR1034606	NA
ERR1034607	NA
ERR1034608	NA
ERR1034609	NA
ERR1034610	NA
ERR1034611	NA
ERR1034612	NA
ERR1034613	NA
ERR1034614	NA
ERR1034615	NA
ERR1034616	NA
ERR1034617	NA
ERR1034618	NA
ERR1034619	NA
ERR1034620	NA
ERR1034621	NA
ERR1034622	NA
ERR1034623	NA
ERR1034624	NA
ERR1034625	NA
ERR1034626	NA
ERR1034627	NA
ERR1034628	NA
ERR1034629	NA
ERR1034630	NA
ERR1034631	NA
ERR1034632	NA
ERR1034633	NA
ERR1034634	NA
ERR1034635	NA
ERR1034636	NA

ERR1034637	NA
ERR1034638	NA
ERR1034639	NA
ERR1034640	NA
ERR1034641	NA
ERR1034642	NA
ERR1034643	NA
ERR1034644	NA
ERR1034645	NA
ERR1034646	NA
ERR1034647	NA
ERR1034648	NA
ERR1034649	NA
ERR1034650	NA
ERR1034651	NA
ERR1034652	NA
ERR1034653	NA
ERR1034654	NA
ERR1034655	NA
ERR1034656	NA
ERR1034657	NA
ERR1034658	NA
ERR1034659	NA
ERR1034660	NA
ERR1034661	NA
ERR1034662	NA
ERR1034663	NA
ERR1034664	NA
ERR1034665	NA
ERR1034666	NA
ERR1034667	NA
ERR1034668	NA
ERR1034669	NA
ERR1034670	NA
ERR1034671	NA
ERR1034672	NA
ERR1034673	NA
ERR1034674	NA
ERR1034675	NA
ERR1034676	NA
ERR1034677	NA
ERR1034678	NA
ERR1034679	NA
ERR1034680	NA
ERR1034681	NA
ERR1034682	NA
ERR1034683	NA
ERR1034684	NA
ERR1034685	NA
ERR1034686	NA

ERR1034687	NA
ERR1034688	NA
ERR1034689	NA
ERR1034690	NA
ERR1034691	NA
ERR1034692	NA
ERR1034693	NA
ERR1034694	NA
ERR1034695	NA
ERR1034696	NA
ERR1034697	NA
ERR1034698	NA
ERR1034699	NA
ERR1034700	NA
ERR1034701	NA
ERR1034702	NA
ERR1034703	NA
ERR1034704	NA
ERR1034705	NA
ERR1034706	NA
ERR1034707	NA
ERR1034708	NA
ERR1034709	NA
ERR1034710	NA
ERR1034711	NA
ERR1034712	NA
ERR1034713	NA
ERR1034714	NA
ERR1034715	NA
ERR1034716	NA
ERR1034717	NA
ERR1034718	NA
ERR1034719	NA
ERR1034720	NA
ERR1034721	NA
ERR1034722	NA
ERR1034723	NA
ERR1034724	NA
ERR1034725	NA
ERR1034726	NA
ERR1034727	NA
ERR1034728	NA
ERR1034729	NA
ERR1034730	NA
ERR1034731	NA
ERR1034732	NA
ERR1034733	NA
ERR1034734	NA
ERR1034735	NA
ERR1034736	NA

ERR1034737	NA
ERR1034738	NA
ERR1034739	NA
ERR1034740	NA
ERR1034741	NA
ERR1034742	NA
ERR1034743	NA
ERR1034744	NA
ERR1034745	NA
ERR1034746	NA
ERR1034747	NA
ERR1034748	NA
ERR1034749	NA
ERR1034750	NA
ERR1034751	NA
ERR1034752	NA
ERR1034753	NA
ERR1034754	NA
ERR1034755	NA
ERR1034756	NA
ERR1034757	NA
ERR1034758	NA
ERR1034759	NA
ERR1034760	NA
ERR1034761	NA
ERR1034762	NA
ERR1034763	NA
ERR1034764	NA
ERR1034765	NA
ERR1034766	NA
ERR1034767	NA
ERR1034768	NA
ERR1034769	NA
ERR1034770	NA
ERR1034771	NA
ERR1034772	NA
ERR1034773	NA
ERR1034774	NA
ERR1034775	NA
ERR1034776	NA
ERR1034777	NA
ERR1034778	NA
ERR1034779	NA
ERR1034780	NA
ERR1034781	NA
ERR1034782	NA
ERR1034783	NA
ERR1034784	NA
ERR1034785	NA
ERR1034786	NA

ERR1034787	NA
ERR1034788	NA
ERR1034789	NA
ERR1034790	NA
ERR1034791	NA
ERR1034792	NA
ERR1034793	NA
ERR1034794	NA
ERR1034795	NA
ERR1034796	NA
ERR1034797	NA
ERR1034798	NA
ERR1034799	NA
ERR1034800	NA
ERR1034801	NA
ERR1034802	NA
ERR1034803	NA
ERR1034804	NA
ERR1034805	NA
ERR1034806	NA
ERR1034807	NA
ERR1034808	NA
ERR1034809	NA
ERR1034810	NA
ERR1034811	NA
ERR1034812	NA
ERR1034813	NA
ERR1034814	NA
ERR1034815	NA
ERR1034816	NA
ERR1034817	NA
ERR1034818	NA
ERR1034819	NA
ERR1034820	NA
ERR1034821	NA
ERR1034822	NA
ERR1034823	NA
ERR1034824	NA
ERR1034825	NA
ERR1034826	NA
ERR1034827	NA
ERR1034828	NA
ERR1034829	NA
ERR1034830	NA
ERR1034831	NA
ERR1034832	NA
ERR1034833	NA
ERR1034834	NA
ERR1034835	NA
ERR1034836	NA

ERR1034837	NA
ERR1034838	NA
ERR1034839	NA
ERR1034840	NA
ERR1034841	NA
ERR1034842	NA
ERR1034843	NA
ERR1034844	NA
ERR1034845	NA
ERR1034846	NA
ERR1034847	NA
ERR1034848	NA
ERR1034849	NA
ERR1034850	NA
ERR1034851	NA
ERR1034852	NA
ERR1034853	NA
ERR1034854	NA
ERR1034855	NA
ERR1034856	NA
ERR1034857	NA
ERR1034858	NA
ERR1034859	NA
ERR1034860	NA
ERR1034861	NA
ERR1034862	NA
ERR1034863	NA
ERR1034864	NA
ERR1034865	NA
ERR1034866	NA
ERR1034867	NA
ERR1034868	NA
ERR1034869	NA
ERR1034870	NA
ERR1034871	NA
ERR1034872	NA
ERR1034873	NA
ERR1034874	NA
ERR1034875	NA
ERR1034876	NA
ERR1034877	NA
ERR1034878	NA
ERR1034879	NA
ERR1034880	NA
ERR1034881	NA
ERR1034882	NA
ERR1034883	NA
ERR1034884	NA
ERR1034885	NA
ERR1034886	NA

ERR1034887	NA
ERR1034888	NA
ERR1034889	NA
ERR1034890	NA
ERR1034891	NA
ERR1034892	NA
ERR1034893	NA
ERR1034894	NA
ERR1034895	NA
ERR1034896	NA
ERR1034897	NA
ERR1034898	NA
ERR1034899	NA
ERR1034900	NA
ERR1034901	NA
ERR1034902	NA
ERR1034903	NA
ERR1034904	NA
ERR1034905	NA
ERR1034906	NA
ERR1034907	NA
ERR1034908	NA
ERR1034909	NA
ERR1034910	NA
ERR1034911	NA
ERR1034912	NA
ERR1034913	NA
ERR1034914	NA
ERR1034915	NA
ERR1034916	NA
ERR1034917	NA
ERR1034918	NA
ERR1034919	NA
ERR1034920	NA
ERR1034921	NA
ERR1034922	NA
ERR1034923	NA
ERR1034924	NA
ERR1034925	NA
ERR1034926	NA
ERR1034927	NA
ERR1034928	NA
ERR1034929	NA
ERR1034930	NA
ERR1034931	NA
ERR1034932	NA
ERR1034933	NA
ERR1034934	NA
ERR1034935	NA
ERR1034936	NA

ERR1034937	NA
ERR1034938	NA
ERR1034939	NA
ERR1034940	NA
ERR1034941	NA
ERR1034942	NA
ERR1034943	NA
ERR1034944	NA
ERR1034945	NA
ERR1034946	NA
ERR1034947	NA
ERR1034948	NA
ERR1034949	NA
ERR1034950	NA
ERR1034951	NA
ERR1034952	NA
ERR1034953	NA
ERR1034954	NA
ERR1034955	NA
ERR1034956	NA
ERR1034957	NA
ERR1034958	NA
ERR1034959	NA
ERR1034960	NA
ERR1034961	NA
ERR1034962	NA
ERR1034963	NA
ERR1034964	NA
ERR1034965	NA
ERR1034966	NA
ERR1034967	NA
ERR1034968	NA
ERR1034969	NA
ERR1034970	NA
ERR1034971	NA
ERR1034972	NA
ERR1034973	NA
ERR1034974	NA
ERR1034975	NA
ERR1034976	NA
ERR1034977	NA
ERR1034978	NA
ERR1034979	NA
ERR1034980	NA
ERR1034981	NA
ERR1034982	NA
ERR1034983	NA
ERR1034984	NA
ERR1034985	NA
ERR1034986	NA

ERR1034987	NA
ERR1034988	NA
ERR1034989	NA
ERR1034990	NA
ERR1034991	NA
ERR1034992	NA
ERR1034993	NA
ERR1034994	NA
ERR1034995	NA
ERR1034996	NA
ERR1034997	NA
ERR1034998	NA
ERR1034999	NA
ERR1035000	NA
ERR1035001	NA
ERR1035002	NA
ERR1035003	NA
ERR1035004	NA
ERR1035005	NA
ERR1035006	NA
ERR1035007	NA
ERR1035008	NA
ERR1035009	NA
ERR1035010	NA
ERR1035011	NA
ERR1035012	NA
ERR1035013	NA
ERR1035014	NA
ERR1035015	NA
ERR1035016	NA
ERR1035017	NA
ERR1035018	NA
ERR1035019	NA
ERR1035020	NA
ERR1035021	NA
ERR1035022	NA
ERR1035023	NA
ERR1035024	NA
ERR1035025	NA
ERR1035026	NA
ERR1035027	NA
ERR1035028	NA
ERR1035029	NA
ERR1035030	NA
ERR1035031	NA
ERR1035032	NA
ERR1035033	NA
ERR1035034	NA
ERR1035035	NA
ERR1035036	NA

ERR1035037	NA
ERR1035038	NA
ERR1035039	NA
ERR1035040	NA
ERR1035041	NA
ERR1035042	NA
ERR1035043	NA
ERR1035044	NA
ERR1035045	NA
ERR1035046	NA
ERR1035047	NA
ERR1035048	NA
ERR1035049	NA
ERR1035050	NA
ERR1035051	NA
ERR1035052	NA
ERR1035053	NA
ERR1035054	NA
ERR1035055	NA
ERR1035056	NA
ERR1035057	NA
ERR1035058	NA
ERR1035059	NA
ERR1035060	NA
ERR1035061	NA
ERR1035062	NA
ERR1035063	NA
ERR1035064	NA
ERR1035065	NA
ERR1035066	NA
ERR1035067	NA
ERR1035068	NA
ERR1035069	NA
ERR1035070	NA
ERR1035071	NA
ERR1035072	NA
ERR1035073	NA
ERR1035074	NA
ERR1035075	NA
ERR1035076	NA
ERR1035077	NA
ERR1035078	NA
ERR1035079	NA
ERR1035080	NA
ERR1035081	NA
ERR1035082	NA
ERR1035083	NA
ERR1035084	NA
ERR1035085	NA
ERR1035086	NA

ERR1035087	NA
ERR1035088	NA
ERR1035089	NA
ERR1035090	NA
ERR1035091	NA
ERR1035092	NA
ERR1035093	NA
ERR1035094	NA
ERR1035095	NA
ERR1035096	NA
ERR1035097	NA
ERR1035098	NA
ERR1035099	NA
ERR1035100	NA
ERR1035101	NA
ERR1035102	NA
ERR1035103	NA
ERR1035104	NA
ERR1035105	NA
ERR1035106	NA
ERR1035107	NA
ERR1035108	NA
ERR1035109	NA
ERR1035110	NA
ERR1035111	NA
ERR1035112	NA
ERR1035113	NA
ERR1035114	NA
ERR1035115	NA
ERR1035116	NA
ERR1035117	NA
ERR1035118	NA
ERR1035119	NA
ERR1035120	NA
ERR1035121	NA
ERR1035122	NA
ERR1035123	NA
ERR1035124	NA
ERR1035125	NA
ERR1035126	NA
ERR1035127	NA
ERR1035128	NA
ERR1035129	NA
ERR1035130	NA
ERR1035131	NA
ERR1035132	NA
ERR1035133	NA
ERR1035134	NA
ERR1035135	NA
ERR1035136	NA

ERR1035137	NA
ERR1035138	NA
ERR1035139	NA
ERR1035140	NA
ERR1035141	NA
ERR1035142	NA
ERR1035143	NA
ERR1035144	NA
ERR1035145	NA
ERR1035146	NA
ERR1035147	NA
ERR1035148	NA
ERR1035149	NA
ERR1035150	NA
ERR1035151	NA
ERR1035152	NA
ERR1035153	NA
ERR1035154	NA
ERR1035155	NA
ERR1035156	NA
ERR1035157	NA
ERR1035158	NA
ERR1035159	NA
ERR1035160	NA
ERR1035161	NA
ERR1035162	NA
ERR1035163	NA
ERR1035164	NA
ERR1035165	NA
ERR1035166	NA
ERR1035167	NA
ERR1035168	NA
ERR1035169	NA
ERR1035170	NA
ERR1035171	NA
ERR1035172	NA
ERR1035173	NA
ERR1035174	NA
ERR1035175	NA
ERR1035176	NA
ERR1035177	NA
ERR1035178	NA
ERR1035179	NA
ERR1035180	NA
ERR1035181	NA
ERR1035182	NA
ERR1035183	NA
ERR1035184	NA
ERR1035185	NA
ERR1035186	NA

ERR1035187	NA
ERR1035188	NA
ERR1035189	NA
ERR1035190	NA
ERR1035191	NA
ERR1035192	NA
ERR1035193	NA
ERR1035194	NA
ERR1035195	NA
ERR1035196	NA
ERR1035197	NA
ERR1035198	NA
ERR1035199	NA
ERR1035200	NA
ERR1035201	NA
ERR1035202	NA
ERR1035203	NA
ERR1035204	NA
ERR1035205	NA
ERR1035206	NA
ERR1035207	NA
ERR1035208	NA
ERR1035209	NA
ERR1035210	NA
ERR1035211	NA
ERR1035212	NA
ERR1035213	NA
ERR1035214	NA
ERR1035215	NA
ERR1035216	NA
ERR1035217	NA
ERR1035218	NA
ERR1035219	NA
ERR1035220	NA
ERR1035221	NA
ERR1035222	NA
ERR1035223	NA
ERR1035224	NA
ERR1035225	NA
ERR1035226	NA
ERR1035227	NA
ERR1035228	NA
ERR1035229	NA
ERR1035230	NA
ERR1035231	NA
ERR1035232	NA
ERR1035233	NA
ERR1035234	NA
ERR1035235	NA
ERR1035236	NA

ERR1035237	NA
ERR1035238	NA
ERR1035239	NA
ERR1035240	NA
ERR1035241	NA
ERR1035242	NA
ERR1035243	NA
ERR1035244	NA
ERR1035245	NA
ERR1035246	NA
ERR1035247	NA
ERR1035248	NA
ERR1035249	NA
ERR1035250	NA
ERR1035251	NA
ERR1035252	NA
ERR1035253	NA
ERR1035254	NA
ERR1035255	NA
ERR1035256	NA
ERR1035257	NA
ERR1035258	NA
ERR1035259	NA
ERR1035260	NA
ERR1035261	NA
ERR1035262	NA
ERR1035263	NA
ERR1035264	NA
ERR1035265	NA
ERR1035266	NA
ERR1035267	NA
ERR1035268	NA
ERR1035269	NA
ERR1035270	NA
ERR1035271	NA
ERR1035272	NA
ERR1035273	NA
ERR1035274	NA
ERR1035275	NA
ERR1035276	NA
ERR1035277	NA
ERR1035278	NA
ERR1035279	NA
ERR1035280	NA
ERR1035281	NA
ERR1035282	NA
ERR1035283	NA
ERR1035284	NA
ERR1035285	NA
ERR1035286	NA

ERR1035287	NA
ERR1035288	NA
ERR1035289	NA
ERR1035290	NA
ERR1035291	NA
ERR1035292	NA
ERR1035293	NA
ERR1035294	NA
ERR1035295	NA
ERR1035296	NA
ERR1035297	NA
ERR1035298	NA
ERR1035299	NA
ERR1035300	NA
ERR1035301	NA
ERR1035302	NA
ERR1035303	NA
ERR1035304	NA
ERR1035305	NA
ERR1035306	NA
ERR1035307	NA
ERR1035308	NA
ERR1035309	NA
ERR1035310	NA
ERR1035311	NA
ERR1035312	NA
ERR1035313	NA
ERR1035314	NA
ERR1035315	NA
ERR1035316	NA
ERR1035317	NA
ERR1035318	NA
ERR1035319	NA
ERR1035320	NA
ERR1035321	NA
ERR1035322	NA
ERR1035323	NA
ERR1035324	NA
ERR1035325	NA
ERR1035326	NA
ERR1035327	NA
ERR1035328	NA
ERR1035329	NA
ERR1035330	NA
ERR1035331	NA
ERR1035332	NA
ERR1035333	NA
ERR1035334	NA
ERR1035335	NA
ERR1035336	NA

ERR1035337	NA
ERR1035338	NA
ERR1035339	NA
ERR1035340	NA
ERR1035341	NA
ERR1035342	NA
ERR1035343	NA
ERR1035344	NA
ERR1035345	NA
ERR1035346	NA
ERR1035347	NA
ERR1035348	NA
ERR1035349	NA
ERR1035350	NA
ERR1035351	NA
ERR1035352	NA
ERR1035353	NA
ERR1035354	NA
ERR1035355	NA
ERR1035356	NA
ERR1035357	NA
ERR1035358	NA
ERR1035359	NA
ERR1035360	NA
ERR1035361	NA
ERR1035362	NA
ERR1035363	NA
ERR1035364	NA
ERR1035365	NA
ERR1035882	NA
ERR1036274	NA
ERR1036275	NA
ERR1036276	NA
ERR1036277	NA
ERR1036278	NA
ERR1036279	NA
ERR1036280	NA
ERR1036281	NA
ERR1036282	NA
ERR1036283	NA
ERR1036284	NA
ERR1036285	NA
ERR1036286	NA
ERR1036287	NA
ERR1036288	NA
ERR1036289	NA
ERR1036290	NA
ERR1036291	NA
ERR1036292	NA
ERR1036293	NA

ERR1036294	NA
ERR1036295	NA
ERR1036296	NA
ERR1036297	NA
ERR1036298	NA
ERR1063831	NA
ERR1063832	NA
ERR1063833	NA
ERR1063834	NA
ERR1063835	NA
ERR1063836	NA
ERR1063837	NA
ERR1063838	NA
ERR1063839	NA
ERR1063840	NA
ERR1063841	NA
ERR1063842	NA
ERR1063843	NA
ERR1063844	NA
ERR1063845	NA
ERR1063846	NA
ERR1063847	NA
ERR1063848	NA
ERR1063849	NA
ERR1063850	NA
ERR1063851	NA
ERR1063852	NA
ERR1063853	NA
ERR1063854	NA
ERR1063855	NA
ERR1063856	NA
ERR1063857	NA
ERR1063858	NA
ERR1063859	NA
ERR1063860	NA
ERR1063861	NA
ERR1063862	NA
ERR1063863	NA
ERR1063864	NA
ERR1063865	NA
ERR1063866	NA
ERR1063867	NA
ERR1063868	NA
ERR1063869	NA
ERR1063870	NA
ERR1063871	NA
ERR1063872	NA
ERR1063873	NA
ERR1063874	NA
ERR1063875	NA

ERR1063876	NA
ERR1063877	NA
ERR1063878	NA
ERR1063879	NA
ERR1063880	NA
ERR1063881	NA
ERR1063882	NA
ERR1063883	NA
ERR1063884	NA
ERR1063885	NA
ERR1063886	NA
ERR1063887	NA
ERR1063888	NA
ERR1063889	NA
ERR1063890	NA
ERR1063891	NA
ERR1063892	NA
ERR1063893	NA
ERR1063894	NA
ERR1063895	NA
ERR1063896	NA
ERR108125	South Africa
ERR108126	South Africa
ERR108127	South Africa
ERR108128	South Africa
ERR108129	South Africa
ERR108130	South Africa
ERR108131	South Africa
ERR108132	South Africa
ERR108133	South Africa
ERR108134	South Africa
ERR108135	South Africa
ERR108136	South Africa
ERR108137	South Africa
ERR108138	South Africa
ERR108139	South Africa
ERR108140	South Africa
ERR108141	South Africa
ERR108142	South Africa
ERR108143	South Africa
ERR108144	South Africa
ERR108145	South Africa
ERR108146	South Africa
ERR108147	South Africa
ERR108148	South Africa
ERR108149	South Africa
ERR108150	South Africa
ERR108151	South Africa
ERR108152	South Africa
ERR108153	South Africa

ERR108154	South Africa
ERR108155	South Africa
ERR108156	South Africa
ERR108157	South Africa
ERR108158	South Africa
ERR108159	South Africa
ERR108160	South Africa
ERR108161	South Africa
ERR108162	South Africa
ERR108163	South Africa
ERR108164	South Africa
ERR108165	South Africa
ERR108166	South Africa
ERR108167	South Africa
ERR108168	South Africa
ERR108169	South Africa
ERR108170	South Africa
ERR108171	South Africa
ERR108172	South Africa
ERR108173	South Africa
ERR108174	South Africa
ERR108175	South Africa
ERR108176	South Africa
ERR108177	South Africa
ERR108178	South Africa
ERR108179	South Africa
ERR108180	South Africa
ERR108181	South Africa
ERR108182	South Africa
ERR108183	South Africa
ERR108184	South Africa
ERR108185	South Africa
ERR108186	South Africa
ERR108420	Russia
ERR108421	Russia
ERR108422	Russia
ERR108423	Russia
ERR108424	NA
ERR108425	Russia
ERR108426	Russia
ERR108427	Russia
ERR108428	Russia
ERR108429	Russia
ERR108430	Russia
ERR108431	NA
ERR108432	Russia
ERR108433	Russia
ERR108434	Russia
ERR108435	Russia
ERR108436	Russia

ERR108437	Russia
ERR108438	NA
ERR108439	Russia
ERR108440	Russia
ERR108441	Russia
ERR108442	Russia
ERR108443	Russia
ERR108444	Russia
ERR108445	Russia
ERR108446	Russia
ERR108447	Russia
ERR108448	Russia
ERR108449	Russia
ERR108450	Russia
ERR108451	Russia
ERR108452	Russia
ERR108453	Russia
ERR108454	NA
ERR108455	Russia
ERR108456	Russia
ERR108457	Russia
ERR108458	Russia
ERR108459	Russia
ERR108460	Russia
ERR108461	Russia
ERR108462	Russia
ERR108463	Russia
ERR108464	NA
ERR108465	NA
ERR108466	Russia
ERR108467	Russia
ERR108468	Russia
ERR108469	Russia
ERR108470	Russia
ERR108471	NA
ERR108472	Russia
ERR108473	Russia
ERR108474	Russia
ERR108475	Russia
ERR108476	NA
ERR108477	NA
ERR108478	Russia
ERR108479	Russia
ERR108480	Russia
ERR108481	Russia
ERR108482	Russia
ERR108483	Russia
ERR108484	Russia
ERR108485	Russia
ERR108486	Russia

ERR108487	Russia
ERR108488	Russia
ERR108489	Russia
ERR108490	Russia
ERR108491	Russia
ERR108492	Russia
ERR108493	Russia
ERR108494	Russia
ERR108495	Russia
ERR108496	Russia
ERR108497	Russia
ERR108498	Russia
ERR108499	Russia
ERR108500	Russia
ERR108501	Russia
ERR108502	Russia
ERR108503	Russia
ERR108504	Russia
ERR108505	Russia
ERR108506	Russia
ERR108507	Russia
ERR108508	Russia
ERR108509	Russia
ERR108510	Russia
ERR108511	Russia
ERR108512	Russia
ERR108513	Russia
ERR108514	Russia
ERR108515	Russia
ERR114421	NA
ERR114422	NA
ERR114423	NA
ERR114424	NA
ERR114425	NA
ERR114426	NA
ERR114427	NA
ERR114428	NA
ERR114429	NA
ERR114430	NA
ERR114431	NA
ERR114432	NA
ERR114433	NA
ERR114434	NA
ERR114435	NA
ERR114436	NA
ERR114437	NA
ERR114438	NA
ERR114439	NA
ERR114440	NA
ERR114441	NA

ERR114442	NA
ERR114443	NA
ERR114444	NA
ERR114445	NA
ERR114446	NA
ERR114447	NA
ERR114448	NA
ERR114449	NA
ERR114450	NA
ERR114451	NA
ERR114452	NA
ERR114453	NA
ERR114454	NA
ERR114455	NA
ERR114456	NA
ERR114457	NA
ERR114458	NA
ERR114459	NA
ERR114460	NA
ERR114461	NA
ERR114462	NA
ERR114463	NA
ERR114464	NA
ERR114465	NA
ERR114466	NA
ERR114467	NA
ERR114468	NA
ERR114469	NA
ERR114470	NA
ERR114471	NA
ERR114472	NA
ERR114473	NA
ERR114474	NA
ERR114475	NA
ERR114476	NA
ERR114477	NA
ERR114478	NA
ERR114479	NA
ERR114480	NA
ERR114481	NA
ERR114482	NA
ERR114483	NA
ERR114484	NA
ERR114485	NA
ERR114486	NA
ERR114487	NA
ERR114488	NA
ERR114489	NA
ERR114490	NA
ERR114491	NA

ERR114492	NA
ERR114493	NA
ERR114494	NA
ERR114495	NA
ERR114496	NA
ERR1144974	Australia
ERR1144975	Australia
ERR1144976	Australia
ERR1144977	Australia
ERR1144978	Australia
ERR1144979	Australia
ERR1144980	Australia
ERR1144981	Australia
ERR1144982	Australia
ERR1144983	Australia
ERR1144984	Australia
ERR1144985	Australia
ERR1144986	Australia
ERR1144987	Australia
ERR1144988	Australia
ERR1144989	Australia
ERR1144990	Australia
ERR1144991	Australia
ERR1144992	Australia
ERR1144993	Australia
ERR1144994	Australia
ERR1144995	Australia
ERR114500	NA
ERR114501	NA
ERR114502	NA
ERR114503	NA
ERR114504	NA
ERR114505	NA
ERR114506	NA
ERR114507	NA
ERR114508	NA
ERR114509	NA
ERR114510	NA
ERR114511	NA
ERR114512	NA
ERR114513	NA
ERR114514	NA
ERR114515	NA
ERR114516	NA
ERR1161615	Estonia
ERR1161616	Estonia
ERR1161617	Estonia
ERR1161618	Estonia
ERR1161619	Estonia
ERR1161620	Estonia

ERR1161621	Estonia
ERR1161622	Estonia
ERR1161623	Estonia
ERR1161624	Estonia
ERR117449	Russia
ERR117450	Russia
ERR117451	Russia
ERR117452	Russia
ERR117453	Russia
ERR117454	Russia
ERR117455	Russia
ERR117456	Russia
ERR117457	Russia
ERR117458	Russia
ERR117459	Russia
ERR117460	Russia
ERR117461	NA
ERR117462	Russia
ERR117463	Russia
ERR117464	Russia
ERR117465	Russia
ERR117466	Russia
ERR117467	Russia
ERR117468	Russia
ERR117469	Russia
ERR117470	Russia
ERR117615	NA
ERR117616	NA
ERR117617	NA
ERR117618	NA
ERR117619	NA
ERR117620	NA
ERR117621	NA
ERR117622	NA
ERR117623	NA
ERR117624	NA
ERR117625	NA
ERR117626	NA
ERR117627	NA
ERR117628	NA
ERR117629	NA
ERR117630	NA
ERR117631	NA
ERR117632	NA
ERR117633	NA
ERR117634	NA
ERR117635	NA
ERR117636	NA
ERR117637	NA
ERR117638	NA

ERR117639	NA
ERR117640	NA
ERR117641	NA
ERR117642	NA
ERR117643	NA
ERR117644	NA
ERR117645	NA
ERR117646	NA
ERR117647	NA
ERR117648	NA
ERR117649	NA
ERR117650	NA
ERR117651	NA
ERR117652	NA
ERR117653	NA
ERR117654	NA
ERR117655	NA
ERR117656	NA
ERR117657	NA
ERR117658	NA
ERR117659	NA
ERR117660	NA
ERR117661	NA
ERR117662	NA
ERR117663	NA
ERR117664	NA
ERR117665	NA
ERR117666	NA
ERR117667	NA
ERR117668	NA
ERR117669	NA
ERR117670	NA
ERR117671	NA
ERR117672	NA
ERR117673	NA
ERR117674	NA
ERR117675	NA
ERR117676	NA
ERR117677	NA
ERR117678	NA
ERR117679	NA
ERR117680	NA
ERR117681	NA
ERR117682	NA
ERR117683	NA
ERR117684	NA
ERR117685	NA
ERR117686	NA
ERR117687	NA
ERR117688	NA

ERR117689	NA
ERR117690	NA
ERR117691	NA
ERR117692	NA
ERR117693	NA
ERR117694	NA
ERR117695	NA
ERR117696	NA
ERR117697	NA
ERR117698	NA
ERR117699	NA
ERR117700	NA
ERR117701	NA
ERR117702	NA
ERR117703	NA
ERR117704	NA
ERR117705	NA
ERR117706	NA
ERR117707	NA
ERR117708	NA
ERR117709	NA
ERR117710	NA
ERR117711	NA
ERR117712	NA
ERR117713	NA
ERR117714	NA
ERR117715	NA
ERR117716	NA
ERR117717	NA
ERR117718	NA
ERR117719	NA
ERR117720	NA
ERR117721	NA
ERR117722	NA
ERR117723	NA
ERR117724	NA
ERR117725	NA
ERR117726	NA
ERR117727	NA
ERR117728	NA
ERR117729	NA
ERR117730	NA
ERR117731	NA
ERR117732	NA
ERR117733	NA
ERR117734	NA
ERR117735	NA
ERR117736	NA
ERR117737	NA
ERR1193660	NA

ERR1193661	NA
ERR1193662	NA
ERR1193663	NA
ERR1193664	NA
ERR1193665	NA
ERR1193666	NA
ERR1193667	NA
ERR1193668	NA
ERR1193669	NA
ERR1193670	NA
ERR1193671	NA
ERR1193672	NA
ERR1193673	NA
ERR1193674	NA
ERR1193675	NA
ERR1193676	NA
ERR1193677	NA
ERR1193678	NA
ERR1193679	NA
ERR1193680	NA
ERR1193681	NA
ERR1193682	NA
ERR1193683	NA
ERR1193684	NA
ERR1193685	NA
ERR1193686	NA
ERR1193687	NA
ERR1193688	NA
ERR1193689	NA
ERR1193690	NA
ERR1193691	NA
ERR1193692	NA
ERR1193693	NA
ERR1193694	NA
ERR1193695	NA
ERR1193696	NA
ERR1193697	NA
ERR1193698	NA
ERR1193699	NA
ERR1193700	NA
ERR1193701	NA
ERR1193702	NA
ERR1193703	NA
ERR1193704	NA
ERR1193705	NA
ERR1193706	NA
ERR1193707	NA
ERR1193708	NA
ERR1193709	NA
ERR1193710	NA

ERR1193711	NA
ERR1193712	NA
ERR1193713	NA
ERR1193714	NA
ERR1193715	NA
ERR1193716	NA
ERR1193717	NA
ERR1193718	NA
ERR1193719	NA
ERR1193720	NA
ERR1193721	NA
ERR1193722	NA
ERR1193723	NA
ERR1193724	NA
ERR1193725	NA
ERR1193726	NA
ERR1193727	NA
ERR1193728	NA
ERR1193729	NA
ERR1193730	NA
ERR1193731	NA
ERR1193732	NA
ERR1193733	NA
ERR1193734	NA
ERR1193735	NA
ERR1193736	NA
ERR1193737	NA
ERR1193738	NA
ERR1193739	NA
ERR1193740	NA
ERR1193741	NA
ERR1193742	NA
ERR1193743	NA
ERR1193744	NA
ERR1193745	NA
ERR1193746	NA
ERR1193747	NA
ERR1193748	NA
ERR1193749	NA
ERR1193750	NA
ERR1193751	NA
ERR1193752	NA
ERR1193753	NA
ERR1193754	NA
ERR1193755	NA
ERR1193756	NA
ERR1193757	NA
ERR1193758	NA
ERR1193759	NA
ERR1193760	NA

ERR1193761	NA
ERR1193762	NA
ERR1193763	NA
ERR1193764	NA
ERR1193765	NA
ERR1193766	NA
ERR1193767	NA
ERR1193768	NA
ERR1193769	NA
ERR1193770	NA
ERR1193771	NA
ERR1193772	NA
ERR1193773	NA
ERR1193774	NA
ERR1193775	NA
ERR1193776	NA
ERR1193777	NA
ERR1193778	NA
ERR1193779	NA
ERR1193780	NA
ERR1193781	NA
ERR1193782	NA
ERR1193783	NA
ERR1193784	NA
ERR1193785	NA
ERR1193786	NA
ERR1193787	NA
ERR1193788	NA
ERR1193789	NA
ERR1193790	NA
ERR1193791	NA
ERR1193792	NA
ERR1193793	NA
ERR1193794	NA
ERR1193795	NA
ERR1193796	NA
ERR1193797	NA
ERR1193798	NA
ERR1193799	NA
ERR1193800	NA
ERR1193801	NA
ERR1193802	NA
ERR1193803	NA
ERR1193804	NA
ERR1193805	NA
ERR1193806	NA
ERR1193807	NA
ERR1193808	NA
ERR1193809	NA
ERR1193810	NA

ERR1193811	NA
ERR1193812	NA
ERR1193813	NA
ERR1193814	NA
ERR1193815	NA
ERR1193816	NA
ERR1193817	NA
ERR1193818	NA
ERR1193819	NA
ERR1193820	NA
ERR1193821	NA
ERR1193822	NA
ERR1193823	NA
ERR1193824	NA
ERR1193825	NA
ERR1193826	NA
ERR1193827	NA
ERR1193828	NA
ERR1193829	NA
ERR1193830	NA
ERR1193831	NA
ERR1193832	NA
ERR1193833	NA
ERR1193834	NA
ERR1193835	NA
ERR1193836	NA
ERR1193837	NA
ERR1193838	NA
ERR1193839	NA
ERR1193840	NA
ERR1193841	NA
ERR1193842	NA
ERR1193843	NA
ERR1193844	NA
ERR1193845	NA
ERR1193846	NA
ERR1193847	NA
ERR1193848	NA
ERR1193849	NA
ERR1193850	NA
ERR1193851	NA
ERR1193852	NA
ERR1193853	NA
ERR1193854	NA
ERR1193855	NA
ERR1193856	NA
ERR1193857	NA
ERR1193858	NA
ERR1193859	NA
ERR1193860	NA

ERR1193861	NA
ERR1193862	NA
ERR1193863	NA
ERR1193864	NA
ERR1193865	NA
ERR1193866	NA
ERR1193867	NA
ERR1193868	NA
ERR1193869	NA
ERR1193870	NA
ERR1193871	NA
ERR1193872	NA
ERR1193873	NA
ERR1193874	NA
ERR1193875	NA
ERR1193876	NA
ERR1193877	NA
ERR1193878	NA
ERR1193879	NA
ERR1193880	NA
ERR1193881	NA
ERR1193882	NA
ERR1193883	NA
ERR1193884	NA
ERR1193885	NA
ERR1193886	NA
ERR1193887	NA
ERR1193888	NA
ERR1193889	NA
ERR1193890	NA
ERR1193891	NA
ERR1193892	NA
ERR1193893	NA
ERR1193894	NA
ERR1193895	NA
ERR1193896	NA
ERR1193897	NA
ERR1193898	NA
ERR1193899	NA
ERR1193900	NA
ERR1193901	NA
ERR1193902	NA
ERR1193903	NA
ERR1193904	NA
ERR1193905	NA
ERR1194779	Norway
ERR1194780	Norway
ERR1194781	Norway
ERR1194782	Norway
ERR1194783	Norway

ERR1194784	Norway
ERR1194785	Norway
ERR1194786	Norway
ERR1194787	Norway
ERR1194788	Norway
ERR1194789	Norway
ERR1194790	Norway
ERR1194791	Norway
ERR1194792	Norway
ERR1194793	Norway
ERR1194794	Norway
ERR1194795	Norway
ERR1194796	Norway
ERR1194797	Norway
ERR1194798	Norway
ERR1194799	Norway
ERR1194800	Norway
ERR1194801	Norway
ERR1194802	Norway
ERR1194803	Norway
ERR1194804	Norway
ERR1194805	Norway
ERR1194806	Norway
ERR1194807	Norway
ERR1194808	Norway
ERR1194809	Norway
ERR1194810	Norway
ERR1194811	Norway
ERR1194812	Norway
ERR1194813	Norway
ERR1194814	Norway
ERR1194815	Norway
ERR1194816	Norway
ERR1194817	Norway
ERR1199093	NA
ERR1199094	NA
ERR1199095	NA
ERR1199096	NA
ERR1199097	NA
ERR1199098	NA
ERR1199099	NA
ERR1199100	NA
ERR1199101	NA
ERR1199102	NA
ERR1199103	NA
ERR1199104	NA
ERR1199105	NA
ERR1199106	NA
ERR1199107	NA
ERR1199108	NA

ERR1199109	NA
ERR1199110	NA
ERR1199111	NA
ERR1199112	NA
ERR1199113	NA
ERR1199114	NA
ERR1199115	NA
ERR1199116	NA
ERR1199117	NA
ERR1199118	NA
ERR1199119	NA
ERR1199120	NA
ERR1199121	NA
ERR1199122	NA
ERR1199123	NA
ERR1199124	NA
ERR1199125	NA
ERR1199126	NA
ERR1199127	NA
ERR1199128	NA
ERR1199129	NA
ERR1199130	NA
ERR1199131	NA
ERR1199132	NA
ERR1199133	NA
ERR1199134	NA
ERR1199135	NA
ERR1199136	NA
ERR1199137	NA
ERR1199138	NA
ERR1199139	NA
ERR1199140	NA
ERR1199141	NA
ERR1199142	NA
ERR1199143	NA
ERR1199144	NA
ERR1199145	NA
ERR1199146	NA
ERR1199147	NA
ERR1199148	NA
ERR1199149	NA
ERR1199150	NA
ERR1199151	NA
ERR1199152	NA
ERR1199153	NA
ERR1213824	NA
ERR1213825	NA
ERR1213826	NA
ERR1213827	NA
ERR1213828	NA

ERR1213829	NA
ERR1213830	NA
ERR1213831	NA
ERR1213832	NA
ERR1213833	NA
ERR1213834	NA
ERR1213835	NA
ERR1213836	NA
ERR1213837	NA
ERR1213838	NA
ERR1213839	NA
ERR1213840	NA
ERR1213841	NA
ERR1213842	NA
ERR1213843	NA
ERR1213844	NA
ERR1213845	NA
ERR1213846	NA
ERR1213847	NA
ERR1213848	NA
ERR1213849	NA
ERR1213850	NA
ERR1213851	NA
ERR1213852	NA
ERR1213853	NA
ERR1213854	NA
ERR1213855	NA
ERR1213856	NA
ERR1213857	NA
ERR1213858	NA
ERR1213859	NA
ERR1213860	NA
ERR1213861	NA
ERR1213862	NA
ERR1213863	NA
ERR1213864	NA
ERR1213865	NA
ERR1213866	NA
ERR1213867	NA
ERR1213868	NA
ERR1213869	NA
ERR1213870	NA
ERR1213871	NA
ERR1213872	NA
ERR1213873	NA
ERR1213874	NA
ERR1213877	NA
ERR1213878	NA
ERR1213879	NA
ERR1213880	NA

ERR1213881	NA
ERR1213882	NA
ERR1213883	NA
ERR1213884	NA
ERR1213885	NA
ERR1213886	NA
ERR1213887	NA
ERR1213888	NA
ERR1213890	NA
ERR1213891	NA
ERR1213892	NA
ERR1213893	NA
ERR1213894	NA
ERR1213896	NA
ERR1213897	NA
ERR1213898	NA
ERR1213899	NA
ERR1213900	NA
ERR1213901	NA
ERR1213902	NA
ERR1213903	NA
ERR1213904	NA
ERR1213905	NA
ERR1213906	NA
ERR1213907	NA
ERR1213908	NA
ERR1213909	NA
ERR1213910	NA
ERR1213911	NA
ERR1213912	NA
ERR1213913	NA
ERR1213914	NA
ERR1213915	NA
ERR1213916	NA
ERR1213917	NA
ERR1213918	NA
ERR1213919	NA
ERR1213920	NA
ERR1213921	NA
ERR1213922	NA
ERR1213923	NA
ERR1213924	NA
ERR1213925	NA
ERR1213926	NA
ERR1213927	NA
ERR1213928	NA
ERR1213929	NA
ERR1213930	NA
ERR1213931	NA
ERR1213932	NA

ERR1213933	NA
ERR1213934	NA
ERR1213935	NA
ERR1213936	NA
ERR1213937	NA
ERR1213938	NA
ERR1213939	NA
ERR1213940	NA
ERR1213941	NA
ERR1213942	NA
ERR1213943	NA
ERR1213944	NA
ERR1213945	NA
ERR1213946	NA
ERR1213947	NA
ERR1213948	NA
ERR1213949	NA
ERR1213950	NA
ERR1227512	NA
ERR1227513	NA
ERR1227514	NA
ERR1227515	NA
ERR1227516	NA
ERR1227517	NA
ERR1227518	NA
ERR1227519	NA
ERR1227520	NA
ERR1227521	NA
ERR1227522	NA
ERR1227523	NA
ERR1227524	NA
ERR1227525	NA
ERR1227526	NA
ERR1227527	NA
ERR1227528	NA
ERR1227529	NA
ERR1227530	NA
ERR1227531	NA
ERR1227532	NA
ERR1227533	NA
ERR123904	NA
ERR123905	NA
ERR123906	NA
ERR123907	NA
ERR123908	NA
ERR123909	NA
ERR123910	NA
ERR123911	NA
ERR123912	NA
ERR123913	NA

ERR123914	NA
ERR123915	NA
ERR123916	NA
ERR123917	NA
ERR123918	NA
ERR123919	NA
ERR123920	NA
ERR123921	NA
ERR123922	NA
ERR123923	NA
ERR123924	NA
ERR123925	NA
ERR123926	NA
ERR123927	NA
ERR123928	NA
ERR123929	NA
ERR123930	NA
ERR123931	NA
ERR123932	NA
ERR123933	NA
ERR123934	NA
ERR123935	NA
ERR123936	NA
ERR123937	NA
ERR123938	NA
ERR123939	NA
ERR123940	NA
ERR123941	NA
ERR123942	NA
ERR123943	NA
ERR123944	NA
ERR123945	NA
ERR123946	NA
ERR123947	NA
ERR123948	NA
ERR123949	NA
ERR123950	NA
ERR123951	NA
ERR124634	Malawi
ERR124635	Malawi
ERR124636	Malawi
ERR124637	Malawi
ERR124638	Malawi
ERR124639	Malawi
ERR124640	Malawi
ERR124641	Malawi
ERR124642	NA
ERR124643	Malawi
ERR124644	Malawi
ERR124645	Malawi

ERR124646	Malawi
ERR124647	Malawi
ERR124648	Malawi
ERR124649	Malawi
ERR124650	Malawi
ERR124651	Malawi
ERR124652	Malawi
ERR126597	Malawi
ERR126598	Malawi
ERR126599	Malawi
ERR126600	Malawi
ERR126601	Malawi
ERR126602	Malawi
ERR126603	Malawi
ERR126604	Malawi
ERR126605	Malawi
ERR126606	Malawi
ERR126607	Malawi
ERR126608	Malawi
ERR126609	Malawi
ERR126610	Malawi
ERR126611	Malawi
ERR126612	Malawi
ERR126613	Malawi
ERR126614	Malawi
ERR126615	Malawi
ERR126616	Malawi
ERR126617	Malawi
ERR126618	Malawi
ERR126619	Malawi
ERR126620	Malawi
ERR126621	Malawi
ERR126622	Malawi
ERR126623	Malawi
ERR126624	Malawi
ERR126625	Malawi
ERR126626	Malawi
ERR126627	Malawi
ERR126628	Malawi
ERR126629	Malawi
ERR126630	Malawi
ERR126631	Malawi
ERR126632	Malawi
ERR126633	Malawi
ERR126634	Malawi
ERR126635	Malawi
ERR126636	Malawi
ERR126637	Malawi
ERR126638	Malawi
ERR126639	Malawi

ERR126640	Malawi
ERR126641	Malawi
ERR126642	Malawi
ERR126643	Malawi
ERR126644	Malawi
ERR1274655	Canada
ERR1274656	Canada
ERR1274657	Canada
ERR1274658	Canada
ERR1274659	Canada
ERR1274660	Canada
ERR1274661	Canada
ERR1274662	Canada
ERR1274663	Canada
ERR1274664	Canada
ERR1274665	Canada
ERR1274667	Canada
ERR1274668	Canada
ERR1274669	Canada
ERR1274670	Canada
ERR1274671	Canada
ERR1274672	Canada
ERR1274673	Canada
ERR1274677	Canada
ERR1274678	Canada
ERR1274680	Canada
ERR1274685	Canada
ERR1274687	Canada
ERR1274688	Canada
ERR1274690	Canada
ERR1274691	Canada
ERR1274695	Canada
ERR1274696	Canada
ERR1274697	Canada
ERR1274699	Canada
ERR1274700	Canada
ERR1274701	Canada
ERR1274702	Canada
ERR1274703	Canada
ERR1274704	Canada
ERR1274705	Canada
ERR1274707	Canada
ERR1274708	Canada
ERR1274710	Canada
ERR1274711	Canada
ERR1274712	Canada
ERR1274713	Canada
ERR1274714	Canada
ERR1274716	Canada
ERR1274717	Canada

ERR1274718	Canada
ERR133798	Russia
ERR133799	Russia
ERR133800	Russia
ERR133801	Russia
ERR133802	Russia
ERR133803	Russia
ERR133804	Russia
ERR133805	Russia
ERR133806	Russia
ERR133807	Russia
ERR133808	Russia
ERR133809	Russia
ERR133810	Russia
ERR133811	Russia
ERR133812	Russia
ERR133813	Russia
ERR133814	Russia
ERR133815	Russia
ERR133816	NA
ERR133817	Russia
ERR133818	Russia
ERR133819	Russia
ERR133820	Russia
ERR133821	NA
ERR133822	Russia
ERR133823	Russia
ERR133824	Russia
ERR133825	NA
ERR133826	Russia
ERR133827	Russia
ERR133828	Russia
ERR133829	Russia
ERR133830	Russia
ERR133831	Russia
ERR133832	Russia
ERR133833	Russia
ERR133834	Russia
ERR133835	Russia
ERR133836	Russia
ERR133837	Russia
ERR133838	Russia
ERR133839	Russia
ERR133840	Russia
ERR133841	Russia
ERR133842	Russia
ERR133843	NA
ERR133844	Russia
ERR133845	Russia
ERR133846	Russia

ERR133847	Russia
ERR133848	Russia
ERR133849	Russia
ERR133850	Russia
ERR133851	Russia
ERR133852	Russia
ERR133853	Russia
ERR133854	Russia
ERR133855	Russia
ERR133856	Russia
ERR133857	Russia
ERR133858	Russia
ERR133859	Russia
ERR133860	Russia
ERR133861	Russia
ERR133862	Russia
ERR133863	Russia
ERR133864	Russia
ERR133865	Russia
ERR133866	Russia
ERR133867	Russia
ERR133868	Russia
ERR133869	Russia
ERR133870	Russia
ERR133871	Russia
ERR133872	Russia
ERR133873	Russia
ERR133874	Russia
ERR133875	Russia
ERR133876	Russia
ERR133877	Russia
ERR133878	Russia
ERR133879	Russia
ERR133880	Russia
ERR133881	Russia
ERR133882	Russia
ERR133883	Russia
ERR133884	Russia
ERR133885	Russia
ERR133886	Russia
ERR133887	Russia
ERR133888	Russia
ERR133889	Russia
ERR133890	Russia
ERR133891	Russia
ERR133892	Russia
ERR133893	Russia
ERR133894	Russia
ERR133895	Russia
ERR133896	Russia

ERR133897	Russia
ERR133898	Russia
ERR133899	Russia
ERR133900	Russia
ERR133901	Russia
ERR133902	Russia
ERR133903	Russia
ERR133904	Russia
ERR133905	Russia
ERR133906	Russia
ERR133907	Russia
ERR133908	Russia
ERR133909	Russia
ERR133910	Russia
ERR133911	Russia
ERR133912	Russia
ERR133913	Russia
ERR133914	Russia
ERR133915	Russia
ERR133916	NA
ERR133917	Russia
ERR133918	Russia
ERR133919	Russia
ERR133920	Russia
ERR133921	Russia
ERR133922	Russia
ERR133923	NA
ERR133924	Russia
ERR133925	Russia
ERR133926	Russia
ERR133927	Russia
ERR133928	NA
ERR133929	Russia
ERR133930	Russia
ERR133931	Russia
ERR133932	Russia
ERR133933	Russia
ERR133934	Russia
ERR133935	Russia
ERR133936	Russia
ERR133937	Russia
ERR133938	Russia
ERR133939	Russia
ERR133940	NA
ERR133941	Russia
ERR133942	Russia
ERR133943	NA
ERR133944	Russia
ERR133945	Russia
ERR133946	Russia

ERR133947	Russia
ERR133948	Russia
ERR133949	Russia
ERR133950	Russia
ERR133951	Russia
ERR133952	Russia
ERR133953	Russia
ERR133954	Russia
ERR133955	Russia
ERR133956	Russia
ERR133957	Russia
ERR133958	Russia
ERR133959	Russia
ERR133960	Russia
ERR133961	Russia
ERR133962	Russia
ERR133963	Russia
ERR133964	Russia
ERR133965	Russia
ERR133966	Russia
ERR133967	Russia
ERR133968	Russia
ERR133969	Russia
ERR133970	Russia
ERR133971	Russia
ERR133972	NA
ERR133973	Russia
ERR133974	Russia
ERR133975	Russia
ERR133976	Russia
ERR133977	Russia
ERR133978	Russia
ERR133979	Russia
ERR133980	Russia
ERR133981	Russia
ERR133982	Russia
ERR133983	Russia
ERR133984	Russia
ERR133985	Russia
ERR133986	NA
ERR133987	Russia
ERR133988	Russia
ERR133989	Russia
ERR1352298	China
ERR1352299	China
ERR1352300	China
ERR1352301	China
ERR1352302	China
ERR1352303	China
ERR1352304	China

ERR1352305	China
ERR1352306	China
ERR1352307	China
ERR1352308	China
ERR1352309	China
ERR1352310	China
ERR1352311	China
ERR1352312	China
ERR1352313	China
ERR1352314	China
ERR1352315	China
ERR1352316	China
ERR1352317	China
ERR1352318	China
ERR1352319	China
ERR1352320	China
ERR1352321	China
ERR1352322	China
ERR1352323	China
ERR1352324	China
ERR1352325	China
ERR1352326	China
ERR1352327	China
ERR1352328	China
ERR1352329	China
ERR1352330	China
ERR1352331	China
ERR1352332	China
ERR1352333	China
ERR1352334	China
ERR1352335	China
ERR1352336	China
ERR1352337	China
ERR1352338	China
ERR1352339	China
ERR1352340	China
ERR1352341	China
ERR1352342	China
ERR1352343	China
ERR1352344	China
ERR1352345	China
ERR1352346	China
ERR1352347	China
ERR1352348	China
ERR1352349	China
ERR1352350	China
ERR1352351	China
ERR1352352	China
ERR1352353	China
ERR1352354	China

ERR1352355	China
ERR1352356	China
ERR1352357	China
ERR1352358	China
ERR1367610	NA
ERR1367611	NA
ERR1367612	NA
ERR1367613	NA
ERR1367614	NA
ERR1367615	NA
ERR1367616	NA
ERR1367617	NA
ERR1367618	NA
ERR1367619	NA
ERR1367620	NA
ERR1367621	NA
ERR1367622	NA
ERR1367623	NA
ERR1367624	NA
ERR1367625	NA
ERR1367626	NA
ERR1367627	NA
ERR1367628	NA
ERR1367629	NA
ERR1367630	NA
ERR1367631	NA
ERR1367632	NA
ERR1367633	NA
ERR1367634	NA
ERR1367635	NA
ERR1367636	NA
ERR1367637	NA
ERR1367638	NA
ERR1367639	NA
ERR1367640	NA
ERR1367641	NA
ERR1367642	NA
ERR1367643	NA
ERR1367644	NA
ERR1367645	NA
ERR1367646	NA
ERR1367647	NA
ERR1367648	NA
ERR1367649	NA
ERR1367650	NA
ERR1367651	NA
ERR1367652	NA
ERR1367653	NA
ERR1367654	NA
ERR1367655	NA

ERR1367656	NA
ERR1367657	NA
ERR1367658	NA
ERR1367659	NA
ERR1367660	NA
ERR1367661	NA
ERR1367662	NA
ERR1367663	NA
ERR1367664	NA
ERR1367665	NA
ERR1367666	NA
ERR1367667	NA
ERR1367668	NA
ERR1367669	NA
ERR1367670	NA
ERR1367671	NA
ERR1367672	NA
ERR1367673	NA
ERR1367674	NA
ERR1367675	NA
ERR1367676	NA
ERR1367677	NA
ERR1367678	NA
ERR1367679	NA
ERR1367680	NA
ERR1367681	NA
ERR1367682	NA
ERR1367683	NA
ERR1367684	NA
ERR1367685	NA
ERR1367686	NA
ERR1367687	NA
ERR1367688	NA
ERR1367689	NA
ERR1367690	NA
ERR1367691	NA
ERR1367692	NA
ERR137191	Russia
ERR137192	Russia
ERR137193	Russia
ERR137194	NA
ERR137195	Russia
ERR137196	Russia
ERR137197	Russia
ERR137198	Russia
ERR137199	Russia
ERR137200	Russia
ERR137201	Russia
ERR137202	Russia
ERR137203	Russia

ERR137204	Russia
ERR137205	Russia
ERR137206	Russia
ERR137207	Russia
ERR137208	Russia
ERR137209	Russia
ERR137210	Russia
ERR137211	Russia
ERR137212	Russia
ERR137213	Russia
ERR137214	Russia
ERR137215	Russia
ERR137216	Russia
ERR137217	Russia
ERR137218	Russia
ERR137219	Russia
ERR137220	Russia
ERR137221	Russia
ERR137222	Russia
ERR137223	Russia
ERR137224	Russia
ERR137225	Russia
ERR137226	NA
ERR137227	Russia
ERR137228	Russia
ERR137229	Russia
ERR137230	Russia
ERR137231	Russia
ERR137232	Russia
ERR137233	Russia
ERR137234	Russia
ERR137235	Russia
ERR137236	Russia
ERR137237	Russia
ERR137238	Russia
ERR137239	Russia
ERR137240	Russia
ERR137241	Russia
ERR137242	Russia
ERR137243	Russia
ERR137244	Russia
ERR137245	Russia
ERR137246	Russia
ERR137247	Russia
ERR137248	Russia
ERR137249	Russia
ERR137250	Russia
ERR137251	Russia
ERR137252	Russia
ERR137253	Russia

ERR137254	Russia
ERR137255	Russia
ERR137256	Russia
ERR137257	Russia
ERR137258	Russia
ERR137259	Russia
ERR137260	Russia
ERR137261	Russia
ERR137262	Russia
ERR137263	Russia
ERR137264	Russia
ERR137265	Russia
ERR137266	Russia
ERR137267	Russia
ERR137268	Russia
ERR137269	Russia
ERR137270	Russia
ERR137271	Russia
ERR137272	Russia
ERR137273	Russia
ERR137274	Russia
ERR137275	Russia
ERR137276	NA
ERR137277	Russia
ERR137278	Russia
ERR137279	Russia
ERR137280	Russia
ERR137281	Russia
ERR137282	Russia
ERR137283	Russia
ERR137284	Russia
ERR137285	Russia
ERR137286	NA
ERR1394278	India
ERR1394279	India
ERR1394280	India
ERR1413470	United Kingdom
ERR1413471	United Kingdom
ERR1413472	United Kingdom
ERR1413473	United Kingdom
ERR1413474	United Kingdom
ERR1413475	United Kingdom
ERR1413476	United Kingdom
ERR1413477	United Kingdom
ERR1413478	United Kingdom
ERR1413479	United Kingdom
ERR1413480	United Kingdom
ERR1413481	United Kingdom
ERR1413482	United Kingdom
ERR1413483	United Kingdom

ERR1413484	United Kingdom
ERR1413485	United Kingdom
ERR1413486	United Kingdom
ERR144542	Russia
ERR144543	Russia
ERR144544	Russia
ERR144545	Russia
ERR144546	Russia
ERR144547	Russia
ERR144548	Russia
ERR144549	Russia
ERR144550	Russia
ERR144551	Russia
ERR144552	Russia
ERR144553	Russia
ERR144554	Russia
ERR144555	Russia
ERR144556	Russia
ERR144557	Russia
ERR144558	Russia
ERR144559	Russia
ERR144560	Russia
ERR144561	Russia
ERR144562	Russia
ERR144563	Russia
ERR144564	Russia
ERR144565	Russia
ERR144566	Russia
ERR144567	Russia
ERR144568	NA
ERR144569	Russia
ERR144570	Russia
ERR144571	Russia
ERR144572	Russia
ERR144573	Russia
ERR144574	Russia
ERR144575	Russia
ERR144576	Russia
ERR144577	Russia
ERR144578	Russia
ERR144579	Russia
ERR144580	Russia
ERR144581	Russia
ERR144582	Russia
ERR144583	NA
ERR144584	Russia
ERR144585	Russia
ERR144586	NA
ERR144587	Russia
ERR144588	Russia

ERR144589	NA
ERR144590	Russia
ERR144591	NA
ERR144592	Russia
ERR144593	Russia
ERR144594	Russia
ERR144595	Russia
ERR144596	Russia
ERR144597	Russia
ERR144598	Russia
ERR144599	Russia
ERR144600	Russia
ERR144601	NA
ERR144602	Russia
ERR144603	Russia
ERR144604	Russia
ERR144605	Russia
ERR144606	Russia
ERR144607	Russia
ERR144608	Russia
ERR144609	Russia
ERR144610	Russia
ERR144611	Russia
ERR144612	NA
ERR144613	Russia
ERR144614	Russia
ERR144615	Russia
ERR144616	Russia
ERR144617	NA
ERR144618	Russia
ERR144619	Russia
ERR144620	Russia
ERR144621	Russia
ERR144622	Russia
ERR144623	Russia
ERR144624	Russia
ERR144625	Russia
ERR144626	Russia
ERR144627	Russia
ERR144628	Russia
ERR144629	Russia
ERR144630	Russia
ERR144631	Russia
ERR144632	Russia
ERR144633	Russia
ERR144634	Russia
ERR144635	Russia
ERR144636	Russia
ERR1452609	South Africa
ERR1452610	South Africa

ERR1452611	South Africa
ERR1452612	South Africa
ERR1452613	South Africa
ERR1452614	South Africa
ERR1452615	South Africa
ERR1452616	South Africa
ERR1452617	South Africa
ERR1465722	NA
ERR1465723	NA
ERR1465724	NA
ERR1465725	NA
ERR1465726	NA
ERR1465727	NA
ERR1465728	NA
ERR1465729	NA
ERR1465730	NA
ERR1465731	NA
ERR1465732	NA
ERR1465733	NA
ERR1465734	NA
ERR1465735	NA
ERR1465736	NA
ERR1465737	NA
ERR1465738	NA
ERR1465739	NA
ERR1465740	NA
ERR1465741	NA
ERR1465742	NA
ERR1465743	NA
ERR1465744	NA
ERR1465745	NA
ERR1465746	NA
ERR1465747	NA
ERR1465748	NA
ERR1465749	NA
ERR1465750	NA
ERR1465751	NA
ERR1465752	NA
ERR1465753	NA
ERR1465754	NA
ERR1465755	NA
ERR1465756	NA
ERR1465757	NA
ERR1465758	NA
ERR1465759	NA
ERR1465760	NA
ERR1465761	NA
ERR1465762	NA
ERR1465763	NA
ERR1465764	NA

ERR1465765	NA
ERR1465766	NA
ERR1465767	NA
ERR1465768	NA
ERR1465769	NA
ERR1465770	NA
ERR1465771	NA
ERR1465772	NA
ERR1465773	NA
ERR1465774	NA
ERR1465775	NA
ERR1465776	NA
ERR1465777	NA
ERR1465778	NA
ERR1465779	NA
ERR1465780	NA
ERR1465781	NA
ERR1465782	NA
ERR1465783	NA
ERR1465784	NA
ERR1465785	NA
ERR1465786	NA
ERR1465787	NA
ERR1465788	NA
ERR1465789	NA
ERR1465790	NA
ERR1465791	NA
ERR1465792	NA
ERR1465793	NA
ERR1465794	NA
ERR1465795	NA
ERR1465796	NA
ERR1465797	NA
ERR1465798	NA
ERR1465799	NA
ERR1465800	NA
ERR1465801	NA
ERR1465802	NA
ERR1465803	NA
ERR1465804	NA
ERR1465805	NA
ERR1465806	NA
ERR1465807	NA
ERR1465808	NA
ERR1465809	NA
ERR1465810	NA
ERR1465811	NA
ERR1465812	NA
ERR1465813	NA
ERR1465814	NA

ERR1465815	NA
ERR1465816	NA
ERR1465817	NA
ERR1465818	NA
ERR1465819	NA
ERR1465820	NA
ERR1465821	NA
ERR1465822	NA
ERR1465823	NA
ERR1465824	NA
ERR1465825	NA
ERR1465826	NA
ERR1465827	NA
ERR1465828	NA
ERR1465829	NA
ERR1465830	NA
ERR1465831	NA
ERR1465832	NA
ERR1465833	NA
ERR1465834	NA
ERR1465835	NA
ERR1465836	NA
ERR1465837	NA
ERR1465838	NA
ERR1465839	NA
ERR1465840	NA
ERR1465841	NA
ERR1465842	NA
ERR1465843	NA
ERR1465844	NA
ERR1465845	NA
ERR1465846	NA
ERR1465847	NA
ERR1465848	NA
ERR1465849	NA
ERR1465850	NA
ERR1465851	NA
ERR1465852	NA
ERR1465853	NA
ERR1465854	NA
ERR1465855	NA
ERR1465856	NA
ERR1465857	NA
ERR1465858	NA
ERR1465859	NA
ERR1465860	NA
ERR1465861	NA
ERR1465862	NA
ERR1465863	NA
ERR1465864	NA

ERR1465865	NA
ERR1465866	NA
ERR1465867	NA
ERR1465868	NA
ERR1465869	NA
ERR1465870	NA
ERR1465871	NA
ERR1465872	NA
ERR1465873	NA
ERR1465874	NA
ERR1465875	NA
ERR1465876	NA
ERR1465877	NA
ERR1465878	NA
ERR1465879	NA
ERR1465880	NA
ERR1465881	NA
ERR1465882	NA
ERR1465883	NA
ERR1465884	NA
ERR1465885	NA
ERR1465886	NA
ERR1465887	NA
ERR1465888	NA
ERR1465889	NA
ERR1465890	NA
ERR1465891	NA
ERR1465892	NA
ERR1465893	NA
ERR1465894	NA
ERR1465895	NA
ERR1465896	NA
ERR1465897	NA
ERR1465898	NA
ERR1465899	NA
ERR1465900	NA
ERR1465901	NA
ERR1465902	NA
ERR1465903	NA
ERR1465904	NA
ERR1465905	NA
ERR1465906	NA
ERR1465907	NA
ERR1465908	NA
ERR1465909	NA
ERR1465910	NA
ERR1465911	NA
ERR1465912	NA
ERR1465913	NA
ERR1465914	NA

ERR1465915	NA
ERR1465916	NA
ERR1465917	NA
ERR1465918	NA
ERR1465919	NA
ERR1465920	NA
ERR1465921	NA
ERR1465922	NA
ERR1465923	NA
ERR1465924	NA
ERR1465925	NA
ERR1465926	NA
ERR1465927	NA
ERR1465928	NA
ERR1465929	NA
ERR1465930	NA
ERR1465931	NA
ERR1465932	NA
ERR1465933	NA
ERR1465934	NA
ERR1465935	NA
ERR1465936	NA
ERR1465937	NA
ERR1465938	NA
ERR1465939	NA
ERR1465940	NA
ERR1465941	NA
ERR1465942	NA
ERR1465943	NA
ERR1465944	NA
ERR1465945	NA
ERR1465946	NA
ERR1465947	NA
ERR1465948	NA
ERR1465949	NA
ERR1465950	NA
ERR1465951	NA
ERR1465952	NA
ERR1465953	NA
ERR1465954	NA
ERR1465955	NA
ERR1465956	NA
ERR1465957	NA
ERR1465958	NA
ERR1465959	NA
ERR1465960	NA
ERR1465961	NA
ERR1465962	NA
ERR1465963	NA
ERR1465964	NA

ERR1465965	NA
ERR1465966	NA
ERR1465967	NA
ERR1465968	NA
ERR1465969	NA
ERR1465970	NA
ERR1465971	NA
ERR1465972	NA
ERR1465973	NA
ERR1465974	NA
ERR1465975	NA
ERR1465976	NA
ERR1465977	NA
ERR1465978	NA
ERR1465979	NA
ERR1465980	NA
ERR1465981	NA
ERR1465982	NA
ERR1465983	NA
ERR1465984	NA
ERR1465985	NA
ERR1465986	NA
ERR1465987	NA
ERR1465988	NA
ERR1465989	NA
ERR1465990	NA
ERR152759	NA
ERR152760	United Kingdom
ERR152761	United Kingdom
ERR152762	NA
ERR1544431	Kazakhstan
ERR1544432	Kazakhstan
ERR1544433	Kazakhstan
ERR1544434	Kazakhstan
ERR1544435	Kazakhstan
ERR1544436	Kazakhstan
ERR1544437	Kazakhstan
ERR1544438	Kazakhstan
ERR1544439	Kazakhstan
ERR1544440	Kazakhstan
ERR1544441	Kazakhstan
ERR1555039	NA
ERR1555040	NA
ERR1555041	NA
ERR1555042	NA
ERR1555043	NA
ERR1555044	NA
ERR1555045	NA
ERR1555046	NA
ERR1555047	NA

ERR1555048	NA
ERR1555049	NA
ERR1555050	NA
ERR1555051	NA
ERR1555052	NA
ERR1555053	NA
ERR1555054	NA
ERR1555055	NA
ERR1555056	NA
ERR1555057	NA
ERR1555058	NA
ERR1555059	NA
ERR1555060	NA
ERR1555061	NA
ERR1555062	NA
ERR1559736	Kazakhstan
ERR1559737	Kazakhstan
ERR1559738	Kazakhstan
ERR1559739	Kazakhstan
ERR1559740	Kazakhstan
ERR1559741	Kazakhstan
ERR1559742	Kazakhstan
ERR1559743	Kazakhstan
ERR1577244	Norway
ERR1577245	Norway
ERR1577246	Norway
ERR1577247	Norway
ERR1577249	Norway
ERR1577250	Norway
ERR1577251	Denmark
ERR1577252	Norway
ERR158569	Russia
ERR158570	Russia
ERR158571	Russia
ERR158572	Russia
ERR158573	Russia
ERR158574	Russia
ERR158575	Russia
ERR158576	Russia
ERR158577	Russia
ERR158578	Russia
ERR158579	Russia
ERR158580	Russia
ERR158581	Russia
ERR158582	Russia
ERR158583	NA
ERR158584	Russia
ERR158585	Russia
ERR158586	Russia
ERR158587	Russia

ERR158588	Russia
ERR158589	Russia
ERR158590	Russia
ERR158591	Russia
ERR158592	Russia
ERR158593	Russia
ERR158594	Russia
ERR158595	Russia
ERR158596	Russia
ERR158597	Russia
ERR158598	Russia
ERR158599	United Kingdom
ERR158600	NA
ERR158601	United Kingdom
ERR158602	United Kingdom
ERR158603	United Kingdom
ERR158604	United Kingdom
ERR158605	United Kingdom
ERR158606	United Kingdom
ERR158607	United Kingdom
ERR158608	United Kingdom
ERR158609	United Kingdom
ERR158610	United Kingdom
ERR158611	Russia
ERR158612	Russia
ERR158613	Russia
ERR158614	Russia
ERR158615	Russia
ERR158616	Russia
ERR161012	Malawi
ERR161013	Malawi
ERR161014	Malawi
ERR161015	Malawi
ERR161016	Malawi
ERR161017	Malawi
ERR161018	Malawi
ERR161019	Malawi
ERR161020	Malawi
ERR161021	Malawi
ERR161022	Malawi
ERR161023	Malawi
ERR161024	Malawi
ERR161025	Malawi
ERR161026	Malawi
ERR161027	Malawi
ERR161028	Malawi
ERR161029	Malawi
ERR161030	Malawi
ERR161031	Malawi
ERR161032	Malawi

ERR161033	NA
ERR161034	Malawi
ERR161035	Malawi
ERR161036	Malawi
ERR161037	Malawi
ERR161038	Malawi
ERR161039	Malawi
ERR161040	Malawi
ERR161041	Malawi
ERR161042	Malawi
ERR161043	Malawi
ERR161044	Malawi
ERR161045	Malawi
ERR161046	Malawi
ERR161047	Malawi
ERR161048	Malawi
ERR161049	Malawi
ERR161050	Malawi
ERR161051	Malawi
ERR161052	Malawi
ERR161053	Malawi
ERR161054	Malawi
ERR161055	Malawi
ERR161056	Malawi
ERR161057	Malawi
ERR161058	Malawi
ERR161059	Malawi
ERR161060	Malawi
ERR161061	Malawi
ERR161062	Malawi
ERR161063	Malawi
ERR161064	Malawi
ERR161065	Malawi
ERR161066	Malawi
ERR161067	Malawi
ERR161068	Malawi
ERR161069	Malawi
ERR161070	Malawi
ERR161071	Malawi
ERR161072	Malawi
ERR161073	Malawi
ERR161074	Malawi
ERR161075	Malawi
ERR161076	Malawi
ERR161077	Malawi
ERR161078	Malawi
ERR161079	Malawi
ERR161080	Malawi
ERR161081	Malawi
ERR161082	Malawi

ERR161083	Malawi
ERR161084	Malawi
ERR161085	Malawi
ERR161086	Malawi
ERR161087	Malawi
ERR161088	Malawi
ERR161089	Malawi
ERR161090	Malawi
ERR161091	Malawi
ERR161092	Malawi
ERR161093	Malawi
ERR161094	Malawi
ERR161095	Malawi
ERR161096	Malawi
ERR161097	Malawi
ERR161098	Malawi
ERR161099	Malawi
ERR161100	Malawi
ERR161101	Malawi
ERR161102	Malawi
ERR161103	Malawi
ERR161104	Malawi
ERR161105	Malawi
ERR161106	Malawi
ERR161107	Malawi
ERR161108	Malawi
ERR161109	Malawi
ERR161110	Malawi
ERR161111	Malawi
ERR161112	Malawi
ERR161113	Malawi
ERR161114	Malawi
ERR161115	Malawi
ERR161116	Malawi
ERR161117	Malawi
ERR161118	Malawi
ERR161119	Malawi
ERR161120	Malawi
ERR161121	Malawi
ERR161122	Malawi
ERR161123	Malawi
ERR161124	Malawi
ERR161125	Malawi
ERR161126	Malawi
ERR161127	Malawi
ERR161128	Malawi
ERR161129	Malawi
ERR161130	Malawi
ERR161131	Malawi
ERR161132	Malawi

ERR161133	Malawi
ERR161134	Malawi
ERR161135	Malawi
ERR161136	Malawi
ERR161137	Malawi
ERR161138	Malawi
ERR161139	Malawi
ERR161140	Malawi
ERR161141	Malawi
ERR161142	Malawi
ERR161143	Malawi
ERR161144	Malawi
ERR161145	Malawi
ERR161146	Malawi
ERR161147	Malawi
ERR161148	Malawi
ERR161149	Malawi
ERR161150	Malawi
ERR161151	Malawi
ERR161152	Malawi
ERR161153	Malawi
ERR161154	Malawi
ERR161155	Malawi
ERR161156	Malawi
ERR161157	Malawi
ERR161158	Malawi
ERR161159	Malawi
ERR161160	Malawi
ERR161161	Malawi
ERR161162	Malawi
ERR161163	Malawi
ERR161164	Malawi
ERR161165	Malawi
ERR161166	Malawi
ERR161167	Malawi
ERR161168	Malawi
ERR161169	Malawi
ERR161170	Malawi
ERR161171	Malawi
ERR161172	Malawi
ERR161173	Malawi
ERR161174	Malawi
ERR161175	Malawi
ERR161176	Malawi
ERR161177	Malawi
ERR161178	Malawi
ERR161179	Malawi
ERR161180	Malawi
ERR161181	Malawi
ERR161182	Malawi

ERR161183	Malawi
ERR161184	Malawi
ERR161185	Malawi
ERR161186	Malawi
ERR161187	Malawi
ERR161188	Malawi
ERR161189	Malawi
ERR161190	Malawi
ERR161191	Malawi
ERR161192	Malawi
ERR161193	Malawi
ERR161194	Malawi
ERR161195	Malawi
ERR161196	Malawi
ERR161197	Malawi
ERR161198	Malawi
ERR161199	Malawi
ERR161200	Malawi
ERR161201	Malawi
ERR161202	Malawi
ERR161203	Malawi
ERR1633775	South Africa
ERR1633776	South Africa
ERR1633777	South Africa
ERR1633778	South Africa
ERR1633779	South Africa
ERR1633780	South Africa
ERR1633781	South Africa
ERR1633784	South Africa
ERR1633785	South Africa
ERR1633786	South Africa
ERR1633788	South Africa
ERR1633790	South Africa
ERR1633791	South Africa
ERR1633792	South Africa
ERR1633793	South Africa
ERR1633794	South Africa
ERR1633795	South Africa
ERR1633796	South Africa
ERR1633798	South Africa
ERR1633799	South Africa
ERR1633800	South Africa
ERR1633801	South Africa
ERR1633802	South Africa
ERR1633804	South Africa
ERR1633805	South Africa
ERR1633806	South Africa
ERR1633809	South Africa
ERR1633811	South Africa
ERR1633813	South Africa

ERR1633814	South Africa
ERR1633815	South Africa
ERR1633816	South Africa
ERR1633817	South Africa
ERR1633818	South Africa
ERR1633819	South Africa
ERR1633820	South Africa
ERR1633821	South Africa
ERR1633822	South Africa
ERR1633823	South Africa
ERR1633824	South Africa
ERR1633825	South Africa
ERR1633827	South Africa
ERR1633828	South Africa
ERR1633829	South Africa
ERR1633830	South Africa
ERR1633831	South Africa
ERR1633832	South Africa
ERR1633833	South Africa
ERR1633834	South Africa
ERR1633835	South Africa
ERR1633836	South Africa
ERR1633837	South Africa
ERR1633838	South Africa
ERR1633839	South Africa
ERR1633840	South Africa
ERR1633841	South Africa
ERR1633843	South Africa
ERR1633844	South Africa
ERR1633845	South Africa
ERR1633846	South Africa
ERR1633847	South Africa
ERR1633851	South Africa
ERR1633854	South Africa
ERR1633855	South Africa
ERR1633856	South Africa
ERR1633858	South Africa
ERR1633859	South Africa
ERR1633861	South Africa
ERR1633862	South Africa
ERR1633863	South Africa
ERR1633865	South Africa
ERR1633866	South Africa
ERR1633867	South Africa
ERR1633868	South Africa
ERR1633870	South Africa
ERR1633871	South Africa
ERR1633872	South Africa
ERR1633874	South Africa
ERR1633875	South Africa

ERR1633876	South Africa
ERR1633877	South Africa
ERR1633879	South Africa
ERR1633880	South Africa
ERR1633881	South Africa
ERR1633882	South Africa
ERR1633883	South Africa
ERR1633884	South Africa
ERR1633885	South Africa
ERR1633888	South Africa
ERR1633891	South Africa
ERR1633892	South Africa
ERR1633894	South Africa
ERR1633895	South Africa
ERR1633896	South Africa
ERR1633897	South Africa
ERR1633898	South Africa
ERR1633899	South Africa
ERR1633900	South Africa
ERR1633901	South Africa
ERR1633902	South Africa
ERR1633903	South Africa
ERR1633904	South Africa
ERR1633905	South Africa
ERR1633906	South Africa
ERR1633908	South Africa
ERR1633914	South Africa
ERR1633915	South Africa
ERR1633917	South Africa
ERR1633918	South Africa
ERR1633921	South Africa
ERR1633922	South Africa
ERR1633923	South Africa
ERR1633924	South Africa
ERR1633925	South Africa
ERR1633926	South Africa
ERR1633927	South Africa
ERR1633928	South Africa
ERR1633929	South Africa
ERR1633930	South Africa
ERR1633933	South Africa
ERR1633934	South Africa
ERR1633936	South Africa
ERR1633937	South Africa
ERR1633938	South Africa
ERR1633939	South Africa
ERR1633940	South Africa
ERR1633941	South Africa
ERR1633942	South Africa
ERR1633944	South Africa

ERR1633945	South Africa
ERR1633946	South Africa
ERR1633947	South Africa
ERR1633948	South Africa
ERR1633950	South Africa
ERR1633951	South Africa
ERR1633952	South Africa
ERR1633953	South Africa
ERR1633954	South Africa
ERR1633955	South Africa
ERR1633956	South Africa
ERR1633957	South Africa
ERR1633959	South Africa
ERR1633962	South Africa
ERR1633963	South Africa
ERR1633964	South Africa
ERR163928	Malawi
ERR163929	Malawi
ERR163930	Malawi
ERR163931	Malawi
ERR163932	Malawi
ERR163933	Malawi
ERR163934	Malawi
ERR163935	Malawi
ERR163936	Malawi
ERR163937	Malawi
ERR163938	Malawi
ERR163939	Malawi
ERR163940	Malawi
ERR163941	Malawi
ERR163942	Malawi
ERR163943	Malawi
ERR163944	Malawi
ERR163945	Malawi
ERR163946	Malawi
ERR163947	Malawi
ERR163948	Malawi
ERR163949	Malawi
ERR163950	Malawi
ERR163951	Malawi
ERR163952	Malawi
ERR163953	Malawi
ERR163954	Malawi
ERR163955	Malawi
ERR163956	Malawi
ERR163957	Malawi
ERR163958	Malawi
ERR163959	Malawi
ERR163960	Malawi
ERR163961	Malawi

ERR163962	Malawi
ERR163963	Malawi
ERR163964	Malawi
ERR163965	Malawi
ERR163966	Malawi
ERR163967	Malawi
ERR163968	Malawi
ERR163969	Malawi
ERR163970	Malawi
ERR163971	Malawi
ERR163972	Malawi
ERR163973	Malawi
ERR163974	Malawi
ERR163975	Malawi
ERR163976	Malawi
ERR163977	Malawi
ERR163978	Malawi
ERR163979	Malawi
ERR163980	Malawi
ERR163981	Malawi
ERR163982	Malawi
ERR163983	Malawi
ERR163984	Malawi
ERR163985	Malawi
ERR163986	Malawi
ERR163987	Malawi
ERR163988	Malawi
ERR163989	Malawi
ERR163990	Malawi
ERR163991	Malawi
ERR163992	Malawi
ERR163993	Malawi
ERR163994	Malawi
ERR163995	Malawi
ERR163996	Malawi
ERR163997	Malawi
ERR163998	Malawi
ERR163999	Malawi
ERR164000	Malawi
ERR164001	Malawi
ERR164002	Malawi
ERR164003	Malawi
ERR164004	Malawi
ERR164005	Malawi
ERR164006	Malawi
ERR164007	Malawi
ERR164008	Malawi
ERR164009	Malawi
ERR164010	Malawi
ERR164011	Malawi

ERR164012	Malawi
ERR164013	Malawi
ERR164014	Malawi
ERR164015	Malawi
ERR164016	Malawi
ERR164017	Malawi
ERR164018	Malawi
ERR164019	Malawi
ERR164020	Malawi
ERR164021	Malawi
ERR164022	Malawi
ERR164023	Malawi
ERR1664619	NA
ERR1664620	NA
ERR1664621	NA
ERR1664622	NA
ERR1664623	NA
ERR1664624	NA
ERR1664625	NA
ERR1664626	NA
ERR1664627	NA
ERR1664628	NA
ERR1664629	NA
ERR1664630	NA
ERR1664631	NA
ERR1664632	NA
ERR1664633	NA
ERR1664634	NA
ERR1664635	NA
ERR1664636	NA
ERR1664637	NA
ERR1664638	NA
ERR1664639	NA
ERR1664640	NA
ERR1664641	NA
ERR1664642	NA
ERR1664643	NA
ERR1664644	NA
ERR1664645	NA
ERR1664646	NA
ERR1664647	NA
ERR1664648	NA
ERR1664649	NA
ERR1664650	NA
ERR1664651	NA
ERR1664652	NA
ERR1664653	NA
ERR1664654	NA
ERR1664655	NA
ERR1664656	NA

ERR1664657	NA
ERR1664658	NA
ERR1664659	NA
ERR1664660	NA
ERR1664661	NA
ERR1664662	NA
ERR1664663	NA
ERR1664664	NA
ERR1664665	NA
ERR1664666	NA
ERR1664667	NA
ERR1664668	NA
ERR1664669	NA
ERR1665402	NA
ERR1665403	NA
ERR1665404	NA
ERR1665405	NA
ERR1679585	Nigeria
ERR1679586	Nigeria
ERR1679587	Nigeria
ERR1679588	Nigeria
ERR1679589	Nigeria
ERR1679590	Nigeria
ERR1679591	Nigeria
ERR1679592	Nigeria
ERR1679593	Nigeria
ERR1679594	Nigeria
ERR1679595	Nigeria
ERR1679596	Nigeria
ERR1679597	Nigeria
ERR1679598	Nigeria
ERR1679599	Nigeria
ERR1679600	Nigeria
ERR1679601	Nigeria
ERR1679603	Nigeria
ERR1679604	Nigeria
ERR1679605	Nigeria
ERR1679606	Nigeria
ERR1679607	Nigeria
ERR1679609	Nigeria
ERR1679610	Nigeria
ERR1679611	Nigeria
ERR1679614	Nigeria
ERR1679615	Nigeria
ERR1679616	Nigeria
ERR1679617	Nigeria
ERR1679618	Nigeria
ERR1679619	Nigeria
ERR1679621	Nigeria
ERR1679622	Nigeria

ERR1679624	Nigeria
ERR1679625	Nigeria
ERR1679626	Nigeria
ERR1679627	Nigeria
ERR1679628	Nigeria
ERR1679629	Nigeria
ERR1679630	Nigeria
ERR1679631	Nigeria
ERR1679632	Nigeria
ERR1679633	Nigeria
ERR1679634	Nigeria
ERR1679635	Nigeria
ERR1679636	Nigeria
ERR1679638	Nigeria
ERR1679639	Nigeria
ERR1679640	Nigeria
ERR1679641	Nigeria
ERR1679642	Nigeria
ERR1679643	Nigeria
ERR1679644	Nigeria
ERR1679645	Nigeria
ERR1679646	Nigeria
ERR1679647	Nigeria
ERR1679648	Nigeria
ERR1679649	Nigeria
ERR1679650	Nigeria
ERR1679651	Nigeria
ERR1679652	Nigeria
ERR1679653	Nigeria
ERR1679654	Nigeria
ERR1679655	Nigeria
ERR1679656	Nigeria
ERR1679657	Nigeria
ERR171128	South Africa
ERR171129	South Africa
ERR171130	South Africa
ERR171131	South Africa
ERR171132	South Africa
ERR171133	South Africa
ERR171134	South Africa
ERR171135	South Africa
ERR171136	South Africa
ERR171137	South Africa
ERR171138	South Africa
ERR171139	South Africa
ERR171140	South Africa
ERR171141	South Africa
ERR171142	South Africa
ERR171143	South Africa
ERR171144	South Africa

ERR171145	South Africa
ERR171146	South Africa
ERR171147	South Africa
ERR171148	South Africa
ERR171149	South Africa
ERR171150	South Africa
ERR171151	South Africa
ERR171152	South Africa
ERR171153	South Africa
ERR171154	Malaysia
ERR171155	Malaysia
ERR171156	Malaysia
ERR171157	Malaysia
ERR171158	Malaysia
ERR171159	Malaysia
ERR171160	Thailand
ERR171161	Thailand
ERR171162	Thailand
ERR171163	Thailand
ERR171164	Thailand
ERR171165	Thailand
ERR171166	South Africa
ERR171167	South Africa
ERR1726500	China
ERR1726501	China
ERR1726502	China
ERR1726503	China
ERR1750873	Botswana
ERR1750874	Botswana
ERR1750875	Zimbabwe
ERR1750876	Zimbabwe
ERR1750877	Zimbabwe
ERR1750878	Zimbabwe
ERR1750879	Zimbabwe
ERR1750880	Zimbabwe
ERR1750881	Zimbabwe
ERR1750882	Zimbabwe
ERR1750883	Zimbabwe
ERR1750884	Zimbabwe
ERR1750885	Zimbabwe
ERR1750886	Zimbabwe
ERR1750887	Zimbabwe
ERR1750888	Zimbabwe
ERR1750889	Zimbabwe
ERR1750890	Zimbabwe
ERR1750891	Zimbabwe
ERR1750892	Zimbabwe
ERR1750893	Zimbabwe
ERR1750894	Zimbabwe
ERR1750895	Zimbabwe

ERR1750896	Zimbabwe
ERR1750897	Zimbabwe
ERR1750898	Zimbabwe
ERR1750899	Zimbabwe
ERR1750900	Zimbabwe
ERR1750901	South Africa
ERR1750902	South Africa
ERR1750903	South Africa
ERR1750904	South Africa
ERR1750905	South Africa
ERR1750906	South Africa
ERR1750907	South Africa
ERR1750908	South Africa
ERR1750909	South Africa
ERR1750910	South Africa
ERR1750911	South Africa
ERR1750912	South Africa
ERR1750913	South Africa
ERR1750914	South Africa
ERR1750915	South Africa
ERR1750916	South Africa
ERR1750917	South Africa
ERR1750918	South Africa
ERR1750919	South Africa
ERR1750920	South Africa
ERR1750921	South Africa
ERR1750922	South Africa
ERR1750923	South Africa
ERR1750924	South Africa
ERR1750925	South Africa
ERR1750926	South Africa
ERR1750927	South Africa
ERR1750928	South Africa
ERR1750929	South Africa
ERR1750930	South Africa
ERR1750931	South Africa
ERR1750932	South Africa
ERR1750933	South Africa
ERR1750934	South Africa
ERR1750935	South Africa
ERR1750936	South Africa
ERR1750937	South Africa
ERR1750938	South Africa
ERR1750939	South Africa
ERR1750940	South Africa
ERR1750941	South Africa
ERR1750942	South Africa
ERR1750943	South Africa
ERR1750944	South Africa
ERR176446	Malawi

ERR176447	Malawi
ERR176448	Malawi
ERR176449	Malawi
ERR176450	Malawi
ERR176451	Malawi
ERR176452	Malawi
ERR176453	Malawi
ERR176454	Malawi
ERR176455	Malawi
ERR176456	Malawi
ERR176457	Malawi
ERR176458	Malawi
ERR176459	Malawi
ERR176460	Malawi
ERR176461	Malawi
ERR176462	Malawi
ERR176463	Malawi
ERR176464	Malawi
ERR176465	Malawi
ERR176466	Malawi
ERR176467	Malawi
ERR176468	Malawi
ERR176469	Malawi
ERR176470	Malawi
ERR176471	Malawi
ERR176472	Malawi
ERR176473	Malawi
ERR176474	Malawi
ERR176475	Malawi
ERR176476	Malawi
ERR176477	Malawi
ERR176478	Malawi
ERR176479	Malawi
ERR176480	Malawi
ERR176481	Malawi
ERR176482	Malawi
ERR176483	Malawi
ERR176484	Malawi
ERR176485	Malawi
ERR176486	Malawi
ERR176487	Malawi
ERR176488	Malawi
ERR176489	Malawi
ERR176490	Malawi
ERR176491	Malawi
ERR176492	Malawi
ERR176493	Malawi
ERR176494	Malawi
ERR176495	Malawi
ERR176496	Malawi

ERR176497	Malawi
ERR176498	Malawi
ERR176499	Malawi
ERR176500	Malawi
ERR176501	Malawi
ERR176502	Malawi
ERR176503	Malawi
ERR176504	Malawi
ERR176505	Malawi
ERR176506	Malawi
ERR176507	Malawi
ERR176508	Malawi
ERR176509	Malawi
ERR176510	Malawi
ERR176511	Malawi
ERR176512	Malawi
ERR176513	Malawi
ERR176514	Malawi
ERR176515	Malawi
ERR176516	Malawi
ERR176517	Malawi
ERR176518	Malawi
ERR176519	Malawi
ERR176520	Malawi
ERR176521	Malawi
ERR176522	Malawi
ERR176523	Malawi
ERR176524	Malawi
ERR176525	Malawi
ERR176526	Malawi
ERR176527	Malawi
ERR176528	Malawi
ERR176529	Malawi
ERR176530	Malawi
ERR176531	Malawi
ERR176532	Malawi
ERR176533	Malawi
ERR176534	Malawi
ERR176535	Malawi
ERR176536	Malawi
ERR176537	Malawi
ERR176538	Malawi
ERR176539	Malawi
ERR176540	Malawi
ERR176541	Malawi
ERR176542	Malawi
ERR176543	Malawi
ERR176544	Malawi
ERR176545	Malawi
ERR176546	Malawi

ERR176547	Malawi
ERR176548	Malawi
ERR176549	Malawi
ERR176550	Malawi
ERR176551	Malawi
ERR176552	Malawi
ERR176553	Malawi
ERR176554	Malawi
ERR176555	Malawi
ERR176556	Malawi
ERR176557	Malawi
ERR176558	Malawi
ERR176559	Malawi
ERR176560	Malawi
ERR176561	Malawi
ERR176562	Malawi
ERR176563	Malawi
ERR176564	Malawi
ERR176565	Malawi
ERR176566	Malawi
ERR176567	Malawi
ERR176568	Malawi
ERR176569	Malawi
ERR176570	Malawi
ERR176571	Malawi
ERR176572	Malawi
ERR176573	Malawi
ERR176574	Malawi
ERR176575	Malawi
ERR176576	Malawi
ERR176577	Malawi
ERR176578	Malawi
ERR176579	Malawi
ERR176580	Malawi
ERR176581	Malawi
ERR176582	Malawi
ERR176583	Malawi
ERR176584	Malawi
ERR176585	Malawi
ERR176586	Malawi
ERR176587	Malawi
ERR176588	Malawi
ERR176589	Malawi
ERR176590	Malawi
ERR176591	Malawi
ERR176592	Malawi
ERR176593	Malawi
ERR176594	Malawi
ERR176595	Malawi
ERR176596	Malawi

ERR176597	Malawi
ERR176598	Malawi
ERR176599	Malawi
ERR176600	Malawi
ERR176601	Malawi
ERR176602	Malawi
ERR176603	Malawi
ERR176604	Malawi
ERR176605	Malawi
ERR176606	Malawi
ERR176607	Malawi
ERR176608	Malawi
ERR176609	Malawi
ERR176610	Malawi
ERR176611	Malawi
ERR176612	Malawi
ERR176613	Malawi
ERR176614	Malawi
ERR176615	Malawi
ERR176616	Malawi
ERR176617	Malawi
ERR176618	Malawi
ERR176619	Malawi
ERR176620	Malawi
ERR176621	Malawi
ERR176622	Malawi
ERR176623	Malawi
ERR176624	Malawi
ERR176625	Malawi
ERR176626	Malawi
ERR176627	Malawi
ERR176628	Malawi
ERR176629	Malawi
ERR176630	Malawi
ERR176631	Malawi
ERR176632	Malawi
ERR176633	Malawi
ERR176634	Malawi
ERR176635	Malawi
ERR176636	Malawi
ERR176637	Malawi
ERR176638	Malawi
ERR176639	Malawi
ERR176640	Malawi
ERR176641	Malawi
ERR176642	Malawi
ERR176643	Malawi
ERR176644	Malawi
ERR176645	Malawi
ERR176646	Malawi

ERR176647	Malawi
ERR176648	Malawi
ERR176649	Malawi
ERR176650	Malawi
ERR176651	NA
ERR176652	Malawi
ERR176653	Malawi
ERR176654	Malawi
ERR176655	Malawi
ERR176656	Malawi
ERR176657	Malawi
ERR176658	Malawi
ERR176659	Malawi
ERR176660	Malawi
ERR176661	Malawi
ERR176662	Malawi
ERR176663	Malawi
ERR176664	Malawi
ERR176665	Malawi
ERR176666	Malawi
ERR176667	Malawi
ERR176668	Malawi
ERR176669	Malawi
ERR176670	Malawi
ERR176671	Malawi
ERR176672	Malawi
ERR176673	Malawi
ERR176674	Malawi
ERR176675	Malawi
ERR176676	Malawi
ERR176677	Malawi
ERR176678	Malawi
ERR176679	Malawi
ERR176680	Malawi
ERR176681	Malawi
ERR176682	Malawi
ERR176683	Malawi
ERR176684	Malawi
ERR176685	Malawi
ERR176686	Malawi
ERR176687	Malawi
ERR176688	Malawi
ERR176689	Malawi
ERR176690	Malawi
ERR176691	Malawi
ERR176692	Malawi
ERR176693	Malawi
ERR176694	Malawi
ERR176695	Malawi
ERR176696	Malawi

ERR176697	Malawi
ERR176698	Malawi
ERR176699	Malawi
ERR176700	Malawi
ERR176701	Malawi
ERR176702	Malawi
ERR176703	Malawi
ERR176704	Malawi
ERR176705	Malawi
ERR176706	Malawi
ERR176707	Malawi
ERR176708	Malawi
ERR176709	Malawi
ERR176710	Malawi
ERR176711	Malawi
ERR176712	Malawi
ERR176713	Malawi
ERR176714	Malawi
ERR176715	Malawi
ERR176716	Malawi
ERR176717	Malawi
ERR176718	Malawi
ERR176719	Malawi
ERR176720	Malawi
ERR176721	Malawi
ERR176722	Malawi
ERR176723	Malawi
ERR176724	Malawi
ERR176725	Malawi
ERR176726	Malawi
ERR176727	Malawi
ERR176728	Malawi
ERR176729	Malawi
ERR176730	Malawi
ERR176731	Malawi
ERR176732	Malawi
ERR176733	Malawi
ERR176734	Malawi
ERR176735	Malawi
ERR176736	Malawi
ERR176737	Malawi
ERR176738	Malawi
ERR176739	Malawi
ERR176740	Malawi
ERR176741	Malawi
ERR176742	Malawi
ERR176743	Malawi
ERR176744	Malawi
ERR176745	Malawi
ERR176746	Malawi

ERR176747	Malawi
ERR176748	Malawi
ERR176749	Malawi
ERR176750	Malawi
ERR176751	Malawi
ERR176752	Malawi
ERR176753	Malawi
ERR176754	Malawi
ERR176755	Malawi
ERR176756	Malawi
ERR176757	Malawi
ERR176758	Malawi
ERR176759	Malawi
ERR176760	Malawi
ERR176761	Malawi
ERR176762	Malawi
ERR176763	Malawi
ERR176764	Malawi
ERR176765	Malawi
ERR176766	Malawi
ERR176767	Malawi
ERR176768	Malawi
ERR176769	Malawi
ERR176770	Malawi
ERR176771	Malawi
ERR176772	Malawi
ERR176773	Malawi
ERR176774	Malawi
ERR176775	Malawi
ERR176776	Malawi
ERR176777	Malawi
ERR176778	Malawi
ERR176779	Malawi
ERR176780	Malawi
ERR176781	Malawi
ERR176782	Malawi
ERR176783	Malawi
ERR176784	Malawi
ERR176785	Malawi
ERR176786	Malawi
ERR176787	Malawi
ERR176788	Malawi
ERR176789	Malawi
ERR176790	Malawi
ERR176791	Malawi
ERR176792	Malawi
ERR176793	Malawi
ERR176794	Malawi
ERR176795	Malawi
ERR176796	Malawi

ERR176797	Malawi
ERR176798	Malawi
ERR176799	Malawi
ERR176800	Malawi
ERR176801	Malawi
ERR176802	Malawi
ERR176803	Malawi
ERR176804	Malawi
ERR176805	Malawi
ERR176806	Malawi
ERR176807	Malawi
ERR176808	Malawi
ERR176809	Malawi
ERR176810	Malawi
ERR176811	Malawi
ERR176812	Malawi
ERR176813	Malawi
ERR176814	Malawi
ERR176815	Malawi
ERR176816	Malawi
ERR176817	Malawi
ERR176818	Malawi
ERR176819	Malawi
ERR176820	Malawi
ERR176821	Malawi
ERR176822	Malawi
ERR176823	Malawi
ERR176824	Malawi
ERR176825	Malawi
ERR176826	Malawi
ERR176827	Malawi
ERR176828	Malawi
ERR176829	Malawi
ERR181314	NA
ERR181315	NA
ERR181316	NA
ERR181435	NA
ERR181440	NA
ERR1815551	NA
ERR1815552	NA
ERR1815553	NA
ERR1815554	NA
ERR1815555	NA
ERR1815556	NA
ERR1815557	NA
ERR181674	Malawi
ERR181675	Malawi
ERR181676	Malawi
ERR181677	Malawi
ERR181678	Malawi

ERR181679	Malawi
ERR181680	Malawi
ERR181681	Malawi
ERR181682	Malawi
ERR181683	Malawi
ERR181684	Malawi
ERR181685	Malawi
ERR181686	Malawi
ERR181687	Malawi
ERR181688	Malawi
ERR181689	Malawi
ERR181690	Malawi
ERR181691	Malawi
ERR181692	Malawi
ERR181693	Malawi
ERR181694	Malawi
ERR181695	Malawi
ERR181696	Malawi
ERR181697	Malawi
ERR181698	Malawi
ERR181699	Malawi
ERR181700	Malawi
ERR181701	Malawi
ERR181702	Malawi
ERR181703	Malawi
ERR181704	Malawi
ERR181705	Malawi
ERR181706	Malawi
ERR181707	Malawi
ERR181708	Malawi
ERR181709	Malawi
ERR181710	Malawi
ERR181711	Malawi
ERR181712	Malawi
ERR181713	Malawi
ERR181714	Malawi
ERR181715	Malawi
ERR181716	Malawi
ERR181717	Malawi
ERR181718	Malawi
ERR181719	Malawi
ERR181720	Malawi
ERR181721	Malawi
ERR181722	Malawi
ERR181723	Malawi
ERR181724	Malawi
ERR181725	Malawi
ERR181726	Malawi
ERR181727	Malawi
ERR181728	Malawi

ERR181729	Malawi
ERR181730	Malawi
ERR181731	Malawi
ERR181732	Malawi
ERR181733	Malawi
ERR181734	Malawi
ERR181735	Malawi
ERR181736	Malawi
ERR181737	Malawi
ERR181738	Malawi
ERR181739	Malawi
ERR181740	Malawi
ERR181741	Malawi
ERR181742	Malawi
ERR181743	Malawi
ERR181744	Malawi
ERR181745	Malawi
ERR181746	Malawi
ERR181747	Malawi
ERR181748	Malawi
ERR181749	Malawi
ERR181750	Malawi
ERR181751	Malawi
ERR181752	Malawi
ERR181753	Malawi
ERR181754	Malawi
ERR181755	Malawi
ERR181756	Malawi
ERR181757	Malawi
ERR181758	Malawi
ERR181759	Malawi
ERR181760	Malawi
ERR181761	Malawi
ERR181762	Malawi
ERR181763	Malawi
ERR181764	Malawi
ERR181766	Malawi
ERR181767	Malawi
ERR181768	Malawi
ERR181769	Malawi
ERR181770	Malawi
ERR181771	Malawi
ERR181772	Malawi
ERR181773	Malawi
ERR181774	Malawi
ERR181775	Malawi
ERR181776	Malawi
ERR181777	Malawi
ERR181778	Malawi
ERR181779	Malawi

ERR181780	Malawi
ERR181781	Malawi
ERR181782	Malawi
ERR181783	Malawi
ERR181784	Malawi
ERR181785	Malawi
ERR181786	Malawi
ERR181787	Malawi
ERR181788	Malawi
ERR181789	Malawi
ERR181790	Malawi
ERR181791	Malawi
ERR181792	Malawi
ERR181793	Malawi
ERR181794	Malawi
ERR181795	Malawi
ERR181796	Malawi
ERR181797	Malawi
ERR181798	Malawi
ERR181799	Malawi
ERR181800	Malawi
ERR181801	Malawi
ERR181802	Malawi
ERR181803	Malawi
ERR181804	Malawi
ERR181805	Malawi
ERR181806	Malawi
ERR181807	Malawi
ERR181808	Malawi
ERR181809	Malawi
ERR181810	Malawi
ERR181811	Malawi
ERR181812	Malawi
ERR181813	Malawi
ERR181814	Malawi
ERR181815	Malawi
ERR181816	Malawi
ERR181817	Malawi
ERR181818	Malawi
ERR181819	Malawi
ERR181820	Malawi
ERR181821	Malawi
ERR181822	Malawi
ERR181823	Malawi
ERR181824	Malawi
ERR181825	Malawi
ERR181826	Malawi
ERR181827	Malawi
ERR181828	Malawi
ERR181829	Malawi

ERR181830	Malawi
ERR181831	Malawi
ERR181832	Malawi
ERR181833	Malawi
ERR181834	Malawi
ERR181835	Malawi
ERR181836	Malawi
ERR181837	Malawi
ERR181838	Malawi
ERR181839	Malawi
ERR181840	Malawi
ERR181841	Malawi
ERR181842	Malawi
ERR181843	Malawi
ERR181844	Malawi
ERR181845	Malawi
ERR181846	Malawi
ERR181847	Malawi
ERR181848	Malawi
ERR181849	Malawi
ERR181850	Malawi
ERR181851	Malawi
ERR181852	Malawi
ERR181853	Malawi
ERR181854	Malawi
ERR181855	Malawi
ERR181856	Malawi
ERR181857	Malawi
ERR181858	Malawi
ERR181859	Malawi
ERR181860	Malawi
ERR181861	Malawi
ERR181862	Malawi
ERR181863	Malawi
ERR181864	Malawi
ERR181865	Malawi
ERR181866	Malawi
ERR181867	Malawi
ERR181868	Malawi
ERR181869	Malawi
ERR181870	Malawi
ERR181871	Malawi
ERR181872	Malawi
ERR181873	Malawi
ERR181874	Malawi
ERR181875	Malawi
ERR181876	Malawi
ERR181877	Malawi
ERR181878	Malawi
ERR181879	Malawi

ERR181880	Malawi
ERR181881	Malawi
ERR181882	Malawi
ERR181883	Malawi
ERR181884	Malawi
ERR181885	Malawi
ERR181886	Malawi
ERR181887	Malawi
ERR181888	Malawi
ERR181889	Malawi
ERR181890	Malawi
ERR181891	Malawi
ERR181892	Malawi
ERR181893	Malawi
ERR181894	Malawi
ERR181895	Malawi
ERR181896	Malawi
ERR181897	Malawi
ERR181898	Malawi
ERR181899	Malawi
ERR181900	Malawi
ERR181901	Malawi
ERR181902	Malawi
ERR181903	Malawi
ERR181904	Malawi
ERR181905	Malawi
ERR181906	Malawi
ERR181907	Malawi
ERR181908	Malawi
ERR181909	Malawi
ERR181910	Malawi
ERR181911	Malawi
ERR181912	Malawi
ERR181913	Malawi
ERR181914	Malawi
ERR181915	Malawi
ERR181916	Malawi
ERR181917	Malawi
ERR181918	Malawi
ERR181919	Malawi
ERR181920	Malawi
ERR181921	Malawi
ERR181922	Malawi
ERR181923	Malawi
ERR181924	Malawi
ERR181925	Malawi
ERR181926	Malawi
ERR181927	Malawi
ERR181928	Malawi
ERR181929	Malawi

ERR181930	Malawi
ERR181931	Malawi
ERR181932	Malawi
ERR181933	Malawi
ERR181934	Malawi
ERR181935	Malawi
ERR181936	Malawi
ERR181937	Malawi
ERR181938	Malawi
ERR181939	Malawi
ERR181940	Malawi
ERR181941	Malawi
ERR181942	Malawi
ERR181943	Malawi
ERR181944	Malawi
ERR181945	Malawi
ERR181946	Malawi
ERR181947	Malawi
ERR181948	Malawi
ERR181949	Malawi
ERR181950	Malawi
ERR181951	Malawi
ERR181952	Malawi
ERR181953	Malawi
ERR181954	Malawi
ERR181955	Malawi
ERR181956	Malawi
ERR181957	Malawi
ERR181958	Malawi
ERR181959	Malawi
ERR181960	Malawi
ERR181961	Malawi
ERR181962	Malawi
ERR181963	Malawi
ERR181964	Malawi
ERR181965	Malawi
ERR181966	Malawi
ERR181967	Malawi
ERR181968	Malawi
ERR181969	Malawi
ERR181970	Malawi
ERR181971	Malawi
ERR181972	Malawi
ERR181973	Malawi
ERR181974	Malawi
ERR181975	Malawi
ERR181976	Malawi
ERR181977	Malawi
ERR181978	Malawi
ERR181979	Malawi

ERR181980	Malawi
ERR181981	Malawi
ERR181982	Malawi
ERR181983	Malawi
ERR181984	Malawi
ERR181985	Malawi
ERR181986	Malawi
ERR181987	Malawi
ERR181988	Malawi
ERR181989	Malawi
ERR181990	Malawi
ERR181991	Malawi
ERR181992	Malawi
ERR181993	Malawi
ERR181994	Malawi
ERR181995	Malawi
ERR181996	Malawi
ERR181997	Malawi
ERR181998	Malawi
ERR181999	Malawi
ERR182000	Malawi
ERR182001	Malawi
ERR182002	Malawi
ERR182003	Malawi
ERR182004	Malawi
ERR182005	Malawi
ERR182006	Malawi
ERR182007	Malawi
ERR182008	Malawi
ERR182009	Malawi
ERR182010	Malawi
ERR182011	Malawi
ERR182012	Malawi
ERR182013	Malawi
ERR182014	Malawi
ERR182015	Malawi
ERR182016	Malawi
ERR182017	Malawi
ERR182018	Malawi
ERR182019	Malawi
ERR182020	Malawi
ERR182021	Malawi
ERR182022	Malawi
ERR182023	Malawi
ERR182024	Malawi
ERR182025	Malawi
ERR182026	Malawi
ERR182027	Malawi
ERR182028	Malawi
ERR182029	Malawi

ERR182030	Malawi
ERR182031	Malawi
ERR182032	Malawi
ERR182033	Malawi
ERR182034	Malawi
ERR182035	Malawi
ERR182036	Malawi
ERR182037	Malawi
ERR182038	Malawi
ERR182039	Malawi
ERR182040	Malawi
ERR182041	Malawi
ERR182042	Malawi
ERR182043	Malawi
ERR182044	Malawi
ERR182045	Malawi
ERR182046	Malawi
ERR182047	Malawi
ERR182048	Malawi
ERR182049	Malawi
ERR182050	Malawi
ERR182051	Malawi
ERR182052	Malawi
ERR182053	Malawi
ERR182054	Malawi
ERR182055	Malawi
ERR182056	Malawi
ERR182057	Malawi
ERR1873389	South Africa
ERR1873390	South Africa
ERR1873391	South Africa
ERR1873392	South Africa
ERR1873393	South Africa
ERR1873394	South Africa
ERR1873395	South Africa
ERR1873396	South Africa
ERR1873397	South Africa
ERR1873398	South Africa
ERR1873399	South Africa
ERR1873400	South Africa
ERR1873401	South Africa
ERR1873402	South Africa
ERR1873403	South Africa
ERR1873404	South Africa
ERR1873405	South Africa
ERR1873406	South Africa
ERR1873407	South Africa
ERR1873408	South Africa
ERR1873409	South Africa
ERR1873410	South Africa

ERR1873411	South Africa
ERR1873412	South Africa
ERR1873413	South Africa
ERR1873414	South Africa
ERR1873415	South Africa
ERR1873416	South Africa
ERR1873417	South Africa
ERR1873418	South Africa
ERR1873419	South Africa
ERR1873420	South Africa
ERR1873421	South Africa
ERR1873422	South Africa
ERR1873423	South Africa
ERR1873424	South Africa
ERR1873425	South Africa
ERR1873426	South Africa
ERR1873427	South Africa
ERR1873428	South Africa
ERR1873429	South Africa
ERR1873430	South Africa
ERR1873431	South Africa
ERR1873432	South Africa
ERR1873433	South Africa
ERR1873434	South Africa
ERR1873435	South Africa
ERR1873436	South Africa
ERR1873437	South Africa
ERR1873438	South Africa
ERR1873439	South Africa
ERR1873440	South Africa
ERR1873441	South Africa
ERR1873442	South Africa
ERR1873443	South Africa
ERR1873444	South Africa
ERR1873445	South Africa
ERR1873446	South Africa
ERR1873447	South Africa
ERR1873448	South Africa
ERR1873449	South Africa
ERR1873450	South Africa
ERR1873451	South Africa
ERR1873452	South Africa
ERR1873453	South Africa
ERR1873454	South Africa
ERR1873455	South Africa
ERR1873456	South Africa
ERR1873457	South Africa
ERR1873458	South Africa
ERR1873459	South Africa
ERR1873460	South Africa

ERR1873461	South Africa
ERR1873462	South Africa
ERR1873463	South Africa
ERR1873464	South Africa
ERR1873465	South Africa
ERR1873466	South Africa
ERR1873467	South Africa
ERR1873468	South Africa
ERR1873469	South Africa
ERR1873470	South Africa
ERR1873471	South Africa
ERR1873472	South Africa
ERR1873473	South Africa
ERR1873474	South Africa
ERR1873475	South Africa
ERR1873476	South Africa
ERR1873477	South Africa
ERR1873478	South Africa
ERR1873479	South Africa
ERR1873480	South Africa
ERR1873481	South Africa
ERR1873482	South Africa
ERR1873483	South Africa
ERR1873484	South Africa
ERR1873485	South Africa
ERR1873486	South Africa
ERR1873487	South Africa
ERR1873488	South Africa
ERR1873489	South Africa
ERR1873490	South Africa
ERR1873491	South Africa
ERR1873492	South Africa
ERR1873493	South Africa
ERR1873494	South Africa
ERR1873495	South Africa
ERR1873496	South Africa
ERR1873497	South Africa
ERR1873498	South Africa
ERR1873499	South Africa
ERR1873500	South Africa
ERR1873501	South Africa
ERR1873502	South Africa
ERR1873503	South Africa
ERR1873504	South Africa
ERR1873505	South Africa
ERR1873506	South Africa
ERR1873507	South Africa
ERR1873508	South Africa
ERR1873509	South Africa
ERR1873510	South Africa

ERR1873511	South Africa
ERR1873512	South Africa
ERR1873514	South Africa
ERR1873515	South Africa
ERR1873516	South Africa
ERR1873517	South Africa
ERR1873518	South Africa
ERR1873519	South Africa
ERR1873520	South Africa
ERR1873521	South Africa
ERR1873522	South Africa
ERR1873523	South Africa
ERR1873524	South Africa
ERR1873525	South Africa
ERR1873526	South Africa
ERR1873527	South Africa
ERR1873528	South Africa
ERR1873529	South Africa
ERR1873530	South Africa
ERR1873531	South Africa
ERR1873532	South Africa
ERR1873533	South Africa
ERR1873534	South Africa
ERR1873535	South Africa
ERR1873536	South Africa
ERR1873537	South Africa
ERR1873538	South Africa
ERR1873539	South Africa
ERR1873540	South Africa
ERR1873541	South Africa
ERR1873542	South Africa
ERR1873543	South Africa
ERR1873544	South Africa
ERR1873545	South Africa
ERR1873546	South Africa
ERR1873547	South Africa
ERR1873548	South Africa
ERR1873549	South Africa
ERR1873550	South Africa
ERR1873551	South Africa
ERR1873552	South Africa
ERR1873553	South Africa
ERR1873554	South Africa
ERR1873555	South Africa
ERR1873556	South Africa
ERR1873557	South Africa
ERR1873558	South Africa
ERR1873559	South Africa
ERR1873560	South Africa
ERR1873561	South Africa

ERR1873562	South Africa
ERR1873563	South Africa
ERR1873564	South Africa
ERR1873565	South Africa
ERR190328	Malawi
ERR190329	Malawi
ERR190330	Malawi
ERR190331	Malawi
ERR190332	Malawi
ERR190333	Malawi
ERR190334	Malawi
ERR190335	Malawi
ERR190336	Malawi
ERR190337	Malawi
ERR190338	Malawi
ERR190339	Malawi
ERR190340	Malawi
ERR190341	Malawi
ERR190342	Malawi
ERR190343	Malawi
ERR190344	Malawi
ERR190345	Malawi
ERR190346	Malawi
ERR190347	Malawi
ERR190348	Malawi
ERR190349	Malawi
ERR190350	Malawi
ERR190351	Malawi
ERR190352	Malawi
ERR190353	Malawi
ERR190354	Malawi
ERR190355	Malawi
ERR190356	Malawi
ERR190357	Malawi
ERR190358	Malawi
ERR190359	Malawi
ERR190360	Malawi
ERR190361	Malawi
ERR190362	Malawi
ERR190363	Malawi
ERR190364	Malawi
ERR190365	Malawi
ERR190366	Malawi
ERR190367	Malawi
ERR190368	Malawi
ERR190369	Malawi
ERR190370	Malawi
ERR190371	Malawi
ERR190372	Malawi
ERR190373	Malawi

ERR190374	Malawi
ERR190375	Malawi
ERR190376	Malawi
ERR190377	Malawi
ERR190378	Malawi
ERR190379	Malawi
ERR190380	Malawi
ERR190381	Malawi
ERR190382	Malawi
ERR190383	Malawi
ERR190384	Malawi
ERR190385	Malawi
ERR190386	Malawi
ERR190387	Malawi
ERR190388	Malawi
ERR190389	Malawi
ERR190390	Malawi
ERR190391	Malawi
ERR190392	Malawi
ERR190393	Malawi
ERR190394	Malawi
ERR190395	Malawi
ERR190396	Malawi
ERR190397	Malawi
ERR190398	Malawi
ERR190399	Malawi
ERR190400	Malawi
ERR190401	Malawi
ERR190402	Malawi
ERR190403	Malawi
ERR190404	Malawi
ERR190405	Malawi
ERR190406	Malawi
ERR190407	Malawi
ERR190408	Malawi
ERR190409	Malawi
ERR190410	Malawi
ERR192249	NA
ERR192250	NA
ERR192451	NA
ERR1949951	Denmark
ERR1949952	Denmark
ERR1949953	Denmark
ERR1949954	Denmark
ERR1949955	Denmark
ERR1949956	Denmark
ERR1949957	Denmark
ERR1949958	Denmark
ERR1949959	Denmark
ERR1949960	Denmark

ERR1949961	Denmark
ERR1949962	Denmark
ERR1949963	Denmark
ERR1949964	Denmark
ERR1949965	Denmark
ERR1949966	Denmark
ERR1949967	Denmark
ERR1949968	Denmark
ERR1949969	Denmark
ERR1949970	Denmark
ERR1949971	Denmark
ERR1949972	Denmark
ERR1949973	Denmark
ERR1949974	Denmark
ERR1949975	Denmark
ERR1949976	Denmark
ERR1949977	Denmark
ERR1949978	Denmark
ERR1949979	Denmark
ERR1949980	Denmark
ERR1949981	Denmark
ERR1949982	Denmark
ERR1949983	Denmark
ERR1949984	Denmark
ERR1949985	Denmark
ERR1949986	Denmark
ERR1949987	Denmark
ERR1949988	Denmark
ERR1949989	Denmark
ERR1949990	Denmark
ERR1949991	Denmark
ERR1949992	Denmark
ERR1949993	Denmark
ERR1949994	Denmark
ERR1949995	Denmark
ERR1949996	Denmark
ERR1949997	Denmark
ERR1949998	Denmark
ERR1949999	Denmark
ERR1950000	Denmark
ERR1950001	Denmark
ERR1950002	Denmark
ERR1950003	Denmark
ERR1950004	Denmark
ERR1950005	Denmark
ERR1950006	Denmark
ERR1950007	Denmark
ERR1950008	Denmark
ERR1950009	Denmark
ERR1950010	Denmark

ERR1950011	Denmark
ERR1950012	Denmark
ERR1950013	Denmark
ERR1950014	Denmark
ERR1950015	Denmark
ERR1950016	Denmark
ERR1950017	Denmark
ERR1950018	Denmark
ERR1950019	Denmark
ERR1950020	Denmark
ERR1950021	Denmark
ERR1950022	Denmark
ERR1950023	Denmark
ERR1950024	Denmark
ERR1950025	Denmark
ERR1950026	Denmark
ERR1950027	Denmark
ERR1950028	Denmark
ERR1950029	Denmark
ERR1950030	Denmark
ERR1950031	Denmark
ERR1950032	Denmark
ERR1950033	Denmark
ERR1950034	Denmark
ERR1950035	Denmark
ERR1950036	Denmark
ERR1950037	Denmark
ERR1950038	Denmark
ERR1950039	Denmark
ERR1950040	Denmark
ERR1950041	Denmark
ERR1950042	Denmark
ERR1950043	Denmark
ERR1950044	Denmark
ERR1950045	Denmark
ERR1950046	Denmark
ERR1950047	Denmark
ERR1950048	Denmark
ERR1950049	Denmark
ERR1950050	Denmark
ERR1950051	Denmark
ERR1950052	Denmark
ERR1950053	Denmark
ERR1950054	Denmark
ERR1950055	Denmark
ERR1950056	Denmark
ERR1950057	Denmark
ERR1950058	Denmark
ERR1950059	Denmark
ERR1950060	Denmark

ERR1950061	Denmark
ERR1950062	Denmark
ERR1950063	Denmark
ERR1950064	Denmark
ERR1950065	Denmark
ERR1950066	Denmark
ERR1950067	Denmark
ERR1950068	Denmark
ERR1950069	Denmark
ERR1950070	Denmark
ERR1950071	Denmark
ERR1950072	Denmark
ERR1950073	Denmark
ERR1950074	Denmark
ERR1950075	Denmark
ERR1950076	Denmark
ERR1950077	Denmark
ERR1950078	Denmark
ERR1950079	Denmark
ERR1950080	Denmark
ERR1950081	Denmark
ERR1950082	Denmark
ERR1950083	Denmark
ERR1950084	Denmark
ERR1950085	Denmark
ERR1950086	Denmark
ERR1950087	Denmark
ERR1950088	Denmark
ERR1950089	Denmark
ERR1950090	Denmark
ERR1952138	Ireland
ERR1952140	Ireland
ERR1952141	Ireland
ERR1952142	Ireland
ERR1971849	Ethiopia
ERR1971850	Ethiopia
ERR1971852	Ethiopia
ERR1971853	Ethiopia
ERR1971854	Ethiopia
ERR1971855	Ethiopia
ERR1971856	Ethiopia
ERR1971857	Ethiopia
ERR1971858	Ethiopia
ERR1971859	Ethiopia
ERR1971860	Ethiopia
ERR1971861	Ethiopia
ERR1971862	Ethiopia
ERR1971863	Ethiopia
ERR1971864	Ethiopia
ERR1971865	Ethiopia

ERR1971866	Ethiopia
ERR1971867	Ethiopia
ERR1971868	Ethiopia
ERR1971869	Ethiopia
ERR1971870	Ethiopia
ERR1971871	Ethiopia
ERR1971872	Ethiopia
ERR1971873	Ethiopia
ERR1971874	Ethiopia
ERR1971876	Ethiopia
ERR1971877	Ethiopia
ERR198708	NA
ERR1988847	South Africa
ERR2002984	NA
ERR2002985	NA
ERR202404	NA
ERR202405	NA
ERR202406	NA
ERR202407	NA
ERR202408	NA
ERR202409	NA
ERR202410	NA
ERR202411	NA
ERR202412	NA
ERR202413	NA
ERR202414	NA
ERR2027207	United Kingdom
ERR2027208	United Kingdom
ERR2027209	United Kingdom
ERR2027211	United Kingdom
ERR2027214	United Kingdom
ERR2027217	United Kingdom
ERR2027218	United Kingdom
ERR2027219	United Kingdom
ERR2027220	United Kingdom
ERR2027229	United Kingdom
ERR2027230	United Kingdom
ERR2027231	United Kingdom
ERR2027232	United Kingdom
ERR2027233	United Kingdom
ERR2027247	United Kingdom
ERR2027248	United Kingdom
ERR2027249	United Kingdom
ERR2027250	United Kingdom
ERR2027251	United Kingdom
ERR2027252	United Kingdom
ERR2027253	United Kingdom
ERR2027254	United Kingdom
ERR2027255	United Kingdom
ERR2027256	United Kingdom

ERR2027257	United Kingdom
ERR2027262	United Kingdom
ERR2027263	United Kingdom
ERR2027264	United Kingdom
ERR2027265	United Kingdom
ERR2027271	United Kingdom
ERR2027272	United Kingdom
ERR2027273	United Kingdom
ERR2027277	United Kingdom
ERR2027278	United Kingdom
ERR2027284	United Kingdom
ERR2027285	United Kingdom
ERR2027288	United Kingdom
ERR2027289	United Kingdom
ERR2027290	United Kingdom
ERR2027291	United Kingdom
ERR2027292	United Kingdom
ERR2027293	United Kingdom
ERR2027294	United Kingdom
ERR2027295	United Kingdom
ERR2027297	United Kingdom
ERR2027299	United Kingdom
ERR2027300	United Kingdom
ERR2027301	United Kingdom
ERR2027302	United Kingdom
ERR2027303	United Kingdom
ERR2027304	United Kingdom
ERR2027305	United Kingdom
ERR2036900	Switzerland
ERR2036902	Switzerland
ERR2036905	Switzerland
ERR2036906	Switzerland
ERR2036908	Switzerland
ERR2036912	Switzerland
ERR2036913	Switzerland
ERR2036914	Switzerland
ERR2036916	Switzerland
ERR2036919	Switzerland
ERR2036920	Switzerland
ERR211990	Malawi
ERR211991	Malawi
ERR211992	Malawi
ERR211993	Malawi
ERR211994	Malawi
ERR211995	Malawi
ERR211996	Malawi
ERR211997	Malawi
ERR211998	Malawi
ERR211999	Malawi
ERR212000	Malawi

ERR212001	Malawi
ERR212002	Malawi
ERR212003	Malawi
ERR212004	Malawi
ERR212005	Malawi
ERR212006	Malawi
ERR212007	Malawi
ERR212008	Malawi
ERR212009	Malawi
ERR212010	Malawi
ERR212011	Malawi
ERR212012	Malawi
ERR212013	Malawi
ERR212014	Malawi
ERR212015	Malawi
ERR212016	Malawi
ERR212017	Malawi
ERR212018	Malawi
ERR212019	Malawi
ERR212020	Malawi
ERR212021	Malawi
ERR212022	Malawi
ERR212023	Malawi
ERR212024	Malawi
ERR212025	Malawi
ERR212026	Malawi
ERR212027	Malawi
ERR212028	Malawi
ERR212029	Malawi
ERR212030	Malawi
ERR212031	Malawi
ERR212032	Malawi
ERR212033	Malawi
ERR212034	Malawi
ERR212035	Malawi
ERR212036	Malawi
ERR212037	Malawi
ERR212038	Malawi
ERR212039	Malawi
ERR212040	Malawi
ERR212041	Malawi
ERR212042	Malawi
ERR212043	Malawi
ERR212044	Malawi
ERR212045	Malawi
ERR212046	Malawi
ERR212047	Malawi
ERR212048	Malawi
ERR212049	Malawi
ERR212050	Malawi

ERR212051	Malawi
ERR212052	Malawi
ERR212053	Malawi
ERR212054	Malawi
ERR212055	Malawi
ERR212056	Malawi
ERR212057	Malawi
ERR212058	Malawi
ERR212059	Malawi
ERR212060	Malawi
ERR212061	Malawi
ERR212062	Malawi
ERR212063	Malawi
ERR212064	Malawi
ERR212065	Malawi
ERR212066	Malawi
ERR212067	Malawi
ERR212068	Malawi
ERR212069	Malawi
ERR212070	Malawi
ERR212071	Malawi
ERR212072	Malawi
ERR212073	Malawi
ERR212074	Malawi
ERR212075	Malawi
ERR212076	Malawi
ERR212077	Malawi
ERR212078	Malawi
ERR212079	Malawi
ERR212080	Malawi
ERR212081	Malawi
ERR212082	Malawi
ERR212083	Malawi
ERR212084	Malawi
ERR212085	Malawi
ERR212086	Malawi
ERR212087	Malawi
ERR212088	Malawi
ERR212089	Malawi
ERR212090	Malawi
ERR212091	Malawi
ERR212092	Malawi
ERR212093	Malawi
ERR212094	Malawi
ERR212095	Malawi
ERR212096	Malawi
ERR212097	Malawi
ERR212098	Malawi
ERR212099	Malawi
ERR212100	Malawi

ERR212101	Malawi
ERR212102	Malawi
ERR212103	Malawi
ERR212104	Malawi
ERR212105	Malawi
ERR212106	Malawi
ERR212107	Malawi
ERR212108	Malawi
ERR212109	Malawi
ERR212110	Malawi
ERR212111	Malawi
ERR212112	Malawi
ERR212113	Malawi
ERR212114	Malawi
ERR212115	Malawi
ERR212116	Malawi
ERR212117	Malawi
ERR212118	Malawi
ERR212119	Malawi
ERR212120	Malawi
ERR212121	Malawi
ERR212122	Malawi
ERR212123	Malawi
ERR212124	Malawi
ERR212125	Malawi
ERR212126	Malawi
ERR212127	Malawi
ERR212128	Malawi
ERR212129	Malawi
ERR212130	Malawi
ERR212131	Malawi
ERR212132	Malawi
ERR212133	Malawi
ERR212134	Malawi
ERR212135	Malawi
ERR212136	Malawi
ERR212137	Malawi
ERR212138	Malawi
ERR212139	Malawi
ERR212140	Malawi
ERR212141	Malawi
ERR212142	Malawi
ERR212143	Malawi
ERR212144	Malawi
ERR212145	Malawi
ERR212146	Malawi
ERR212147	Malawi
ERR212148	Malawi
ERR212149	Malawi
ERR212150	Malawi

ERR212151	Malawi
ERR212152	Malawi
ERR212153	Malawi
ERR212154	Malawi
ERR212155	Malawi
ERR212156	Malawi
ERR212157	Malawi
ERR212158	Malawi
ERR212159	Malawi
ERR212160	Malawi
ERR212161	Malawi
ERR212162	Malawi
ERR212163	Malawi
ERR212164	Malawi
ERR212165	Malawi
ERR212166	Malawi
ERR212167	Malawi
ERR212168	Malawi
ERR212169	Malawi
ERR212170	Malawi
ERR212171	Malawi
ERR212172	Malawi
ERR212173	Malawi
ERR212174	Malawi
ERR212175	Malawi
ERR212176	Malawi
ERR212177	Malawi
ERR212178	Malawi
ERR212179	Malawi
ERR212180	Malawi
ERR212181	Malawi
ERR2124075	NA
ERR2124076	NA
ERR2124077	NA
ERR2124078	NA
ERR2124079	NA
ERR2124080	NA
ERR2124081	NA
ERR2124082	NA
ERR2124083	NA
ERR2124084	NA
ERR2124085	NA
ERR2124086	NA
ERR2124087	NA
ERR2124088	NA
ERR2124089	NA
ERR2124090	NA
ERR2124091	NA
ERR2124092	NA
ERR2124093	NA

ERR2124094	NA
ERR2124095	NA
ERR2124096	NA
ERR2124097	NA
ERR2124098	NA
ERR2124099	NA
ERR2124100	NA
ERR2124101	NA
ERR2124102	NA
ERR2124103	NA
ERR2124104	NA
ERR2124105	NA
ERR2124106	NA
ERR2124107	NA
ERR2124108	NA
ERR2145485	NA
ERR2145486	NA
ERR2145487	NA
ERR2145488	NA
ERR2145489	NA
ERR2145490	NA
ERR2145491	NA
ERR2145492	NA
ERR2145493	NA
ERR2145494	NA
ERR2145495	NA
ERR2145496	NA
ERR2145497	NA
ERR2145498	NA
ERR2145499	NA
ERR2145500	NA
ERR2145501	NA
ERR2145502	NA
ERR2145503	NA
ERR2145504	NA
ERR2145505	NA
ERR2145506	NA
ERR2145507	NA
ERR2145508	NA
ERR2145509	NA
ERR2145510	NA
ERR2145511	NA
ERR2145512	NA
ERR2145513	NA
ERR2145514	NA
ERR2145515	NA
ERR2145516	NA
ERR2145517	NA
ERR2145518	NA
ERR2145519	NA

ERR2145520	NA
ERR2145521	NA
ERR2145522	NA
ERR2145523	NA
ERR2145524	NA
ERR216899	Malawi
ERR216900	Malawi
ERR216901	Malawi
ERR216902	Malawi
ERR216903	Malawi
ERR216904	Malawi
ERR216905	Malawi
ERR216906	Malawi
ERR216907	Malawi
ERR216908	Malawi
ERR216909	Malawi
ERR216910	Malawi
ERR216911	Malawi
ERR216912	Malawi
ERR216913	Malawi
ERR216914	Malawi
ERR216915	Malawi
ERR216916	Malawi
ERR216917	Malawi
ERR216918	Malawi
ERR216919	Malawi
ERR216920	Malawi
ERR216921	Malawi
ERR216922	Malawi
ERR216923	Malawi
ERR216924	Malawi
ERR216925	Malawi
ERR216926	Malawi
ERR216927	Malawi
ERR216928	Malawi
ERR216929	Malawi
ERR216930	Malawi
ERR216931	Malawi
ERR216932	Malawi
ERR216933	Malawi
ERR216934	Malawi
ERR216935	Malawi
ERR216936	Malawi
ERR216937	Malawi
ERR216938	Malawi
ERR216939	Malawi
ERR216940	Malawi
ERR216941	Malawi
ERR216942	Malawi
ERR216943	Malawi

ERR216944	Malawi
ERR216945	Malawi
ERR216946	Malawi
ERR216947	Malawi
ERR216948	Malawi
ERR216949	Malawi
ERR216950	Malawi
ERR216951	Malawi
ERR216952	Malawi
ERR216953	Malawi
ERR216954	Malawi
ERR216955	Malawi
ERR216956	Malawi
ERR216957	Malawi
ERR216958	Malawi
ERR216959	Malawi
ERR216960	Malawi
ERR216961	Malawi
ERR216962	Malawi
ERR216963	Malawi
ERR216964	Malawi
ERR216965	Malawi
ERR216966	Malawi
ERR216967	Malawi
ERR216968	Malawi
ERR216969	Malawi
ERR216970	Malawi
ERR216971	Malawi
ERR216972	Malawi
ERR216973	Malawi
ERR216974	Malawi
ERR216975	Malawi
ERR216976	Malawi
ERR216977	Malawi
ERR216978	Malawi
ERR216979	Malawi
ERR216980	Malawi
ERR216981	Malawi
ERR216982	Malawi
ERR216983	Malawi
ERR216984	Malawi
ERR216985	Malawi
ERR216986	Malawi
ERR216987	Malawi
ERR216988	Malawi
ERR216989	Malawi
ERR216990	Malawi
ERR216991	Malawi
ERR216992	Malawi
ERR218154	United States of America

ERR2196996	France
ERR2196997	France
ERR2196998	France
ERR2196999	France
ERR2197000	France
ERR2197001	France
ERR2202576	NA
ERR2202577	NA
ERR2202578	NA
ERR2202579	NA
ERR2202580	NA
ERR2202581	NA
ERR2202582	NA
ERR2202583	NA
ERR2202584	NA
ERR2202585	NA
ERR2202586	NA
ERR2202587	NA
ERR2202588	NA
ERR2202589	NA
ERR2202590	NA
ERR2202591	NA
ERR2202592	NA
ERR2202593	NA
ERR2202594	NA
ERR2202595	NA
ERR2202596	NA
ERR2202597	NA
ERR2202598	NA
ERR2202599	NA
ERR2202600	NA
ERR2202601	NA
ERR2202602	NA
ERR2202603	NA
ERR2202604	NA
ERR2202605	NA
ERR2202606	NA
ERR2202607	NA
ERR2202608	NA
ERR2202609	NA
ERR2202610	NA
ERR2202611	NA
ERR2202612	NA
ERR2202613	NA
ERR2202614	NA
ERR2202615	NA
ERR2202616	NA
ERR2202617	NA
ERR2202618	NA
ERR2202619	NA

ERR2202620	NA
ERR2202621	NA
ERR2202622	NA
ERR2202623	NA
ERR2202624	NA
ERR2202625	NA
ERR2202626	NA
ERR2202627	NA
ERR2202628	NA
ERR2202629	NA
ERR2202630	NA
ERR2202631	NA
ERR2202632	NA
ERR2202633	NA
ERR2202634	NA
ERR2202635	NA
ERR2202636	NA
ERR2202637	NA
ERR2202638	NA
ERR2202639	NA
ERR2202640	NA
ERR2202641	NA
ERR2202642	NA
ERR2202643	NA
ERR2202644	NA
ERR2202645	NA
ERR2202646	NA
ERR2206031	NA
ERR2206032	NA
ERR2206033	NA
ERR2206034	NA
ERR2206035	NA
ERR2206036	NA
ERR2206037	NA
ERR2206038	NA
ERR2206039	NA
ERR2206040	NA
ERR2206041	NA
ERR2206042	NA
ERR2206043	NA
ERR2206044	NA
ERR2206045	NA
ERR2206046	NA
ERR2206047	NA
ERR2206048	NA
ERR2206049	NA
ERR2206050	NA
ERR2206051	NA
ERR2206052	NA
ERR2206053	NA

ERR2206054	NA
ERR2206055	NA
ERR2206618	NA
ERR2206619	NA
ERR2206620	NA
ERR2206621	NA
ERR2206622	NA
ERR2206623	NA
ERR2206624	NA
ERR2206625	NA
ERR221524	Malawi
ERR221525	Malawi
ERR221526	Malawi
ERR221527	Malawi
ERR221528	Malawi
ERR221529	Malawi
ERR221530	Malawi
ERR221531	Malawi
ERR221532	Malawi
ERR221533	Malawi
ERR221534	Malawi
ERR221535	Malawi
ERR221536	Malawi
ERR221537	Malawi
ERR221538	Malawi
ERR221539	Malawi
ERR221540	Malawi
ERR221541	Malawi
ERR221542	Malawi
ERR221543	Malawi
ERR221544	Malawi
ERR221545	Malawi
ERR221546	Malawi
ERR221547	Malawi
ERR221548	Malawi
ERR221549	Malawi
ERR221550	Malawi
ERR221551	Malawi
ERR221552	Malawi
ERR221553	Malawi
ERR221554	Malawi
ERR221555	Malawi
ERR221556	Malawi
ERR221557	Malawi
ERR221558	Malawi
ERR221559	Malawi
ERR221560	Malawi
ERR221561	Malawi
ERR221562	Malawi
ERR221563	Malawi

ERR221564	Malawi
ERR221565	Malawi
ERR221566	Malawi
ERR221567	Malawi
ERR221568	Malawi
ERR221569	Malawi
ERR221570	Malawi
ERR221571	Malawi
ERR221572	Malawi
ERR221573	Malawi
ERR221574	Malawi
ERR221575	Malawi
ERR221576	Malawi
ERR221577	Malawi
ERR221578	Malawi
ERR221579	Malawi
ERR221580	Malawi
ERR221581	Malawi
ERR221582	Malawi
ERR221583	Malawi
ERR221584	Malawi
ERR221585	Malawi
ERR221586	Malawi
ERR221587	Malawi
ERR221588	Malawi
ERR221589	Malawi
ERR221590	Malawi
ERR221591	Malawi
ERR221592	Malawi
ERR221593	Malawi
ERR221594	Malawi
ERR221595	Malawi
ERR221596	Malawi
ERR221597	Malawi
ERR221598	Malawi
ERR221599	Malawi
ERR221600	Malawi
ERR221601	Malawi
ERR221602	Malawi
ERR221603	Malawi
ERR221604	Malawi
ERR221605	Malawi
ERR221606	Malawi
ERR221607	Malawi
ERR221608	Malawi
ERR221609	Malawi
ERR221610	Malawi
ERR221611	Malawi
ERR221612	Malawi
ERR221613	Malawi

ERR221614	Malawi
ERR221615	Malawi
ERR221616	Malawi
ERR221617	Malawi
ERR221618	Malawi
ERR221619	Malawi
ERR221620	NA
ERR221621	NA
ERR221622	NA
ERR221623	NA
ERR221624	NA
ERR221625	NA
ERR221626	NA
ERR221627	NA
ERR221628	NA
ERR221629	NA
ERR221630	NA
ERR221631	NA
ERR221632	NA
ERR221633	NA
ERR221634	NA
ERR221635	NA
ERR221636	NA
ERR221637	NA
ERR221638	NA
ERR221639	NA
ERR221640	NA
ERR221641	NA
ERR221642	NA
ERR221643	NA
ERR221644	NA
ERR221645	NA
ERR221646	NA
ERR221647	NA
ERR221648	NA
ERR221649	NA
ERR221650	NA
ERR221651	NA
ERR221652	NA
ERR221653	NA
ERR221654	NA
ERR221655	NA
ERR221656	NA
ERR221657	NA
ERR221658	NA
ERR221659	NA
ERR221660	NA
ERR221661	NA
ERR221662	NA
ERR221663	NA

ERR221664	NA
ERR221665	NA
ERR221666	NA
ERR221667	NA
ERR2228788	Denmark
ERR2228789	Denmark
ERR2228790	Denmark
ERR2228791	Denmark
ERR2228792	Denmark
ERR2228793	Denmark
ERR2228794	Denmark
ERR2228795	Denmark
ERR2228796	Denmark
ERR2228797	Denmark
ERR2228798	Denmark
ERR2228799	Denmark
ERR2228800	Denmark
ERR2228801	Denmark
ERR2228802	Denmark
ERR2228803	Denmark
ERR2228804	Denmark
ERR2228805	Denmark
ERR2228806	Denmark
ERR2228807	Denmark
ERR2228808	Denmark
ERR2228809	Denmark
ERR2228810	Denmark
ERR2228811	Denmark
ERR2228812	Denmark
ERR2228813	Denmark
ERR2228814	Denmark
ERR2228815	Denmark
ERR2228816	Denmark
ERR2228817	Denmark
ERR2228818	Denmark
ERR2228819	Denmark
ERR2228820	Denmark
ERR2228821	Denmark
ERR2228822	Denmark
ERR2228823	Denmark
ERR2228824	Denmark
ERR2228825	Denmark
ERR2228826	Denmark
ERR2228827	Denmark
ERR2228828	Denmark
ERR2228829	Denmark
ERR2228830	Denmark
ERR2228831	Denmark
ERR2228832	Denmark
ERR2228833	Denmark

ERR2228834	Denmark
ERR2228835	Denmark
ERR2228836	Denmark
ERR2228837	Denmark
ERR2228838	Denmark
ERR2228839	Denmark
ERR2228840	Denmark
ERR2228841	Denmark
ERR2228842	Denmark
ERR2228843	Denmark
ERR2228844	Denmark
ERR2228845	Denmark
ERR2228846	Denmark
ERR2228847	Denmark
ERR2228848	Denmark
ERR2228849	Denmark
ERR2228850	Denmark
ERR2228851	Denmark
ERR2228852	Denmark
ERR2228853	Denmark
ERR2228854	Denmark
ERR2228855	Denmark
ERR2228856	Denmark
ERR2228857	Denmark
ERR2228858	Denmark
ERR2228859	Denmark
ERR2228860	Denmark
ERR2228861	Denmark
ERR2228862	Denmark
ERR2228863	Denmark
ERR2228864	Denmark
ERR2228865	Denmark
ERR2228866	Denmark
ERR2228867	Denmark
ERR2228868	Denmark
ERR2228869	Denmark
ERR2228870	Denmark
ERR2228871	Denmark
ERR2228872	Denmark
ERR2228873	Denmark
ERR2228874	Denmark
ERR2228875	Denmark
ERR2228876	Denmark
ERR2228877	Denmark
ERR2228878	Denmark
ERR2228879	Denmark
ERR2228880	Denmark
ERR2228881	Denmark
ERR2228882	Denmark
ERR2228883	Denmark

ERR2228884	Denmark
ERR2228885	Denmark
ERR2228886	Denmark
ERR2228887	Denmark
ERR2228888	Denmark
ERR2228889	Denmark
ERR2228890	Denmark
ERR2228891	Denmark
ERR2228892	Denmark
ERR2228893	Denmark
ERR2228894	Denmark
ERR2228895	Denmark
ERR2228896	Denmark
ERR2228897	Denmark
ERR2228898	Denmark
ERR2228899	Denmark
ERR2228900	Denmark
ERR2228901	Denmark
ERR2228902	Denmark
ERR2228903	Denmark
ERR2228904	Denmark
ERR2228905	Denmark
ERR2228906	Denmark
ERR2228907	Denmark
ERR2228908	Denmark
ERR2228909	Denmark
ERR2228910	Denmark
ERR2228911	Denmark
ERR2228912	Denmark
ERR2228913	Denmark
ERR2228914	Denmark
ERR2228915	Denmark
ERR2228916	Denmark
ERR2228917	Denmark
ERR2228918	Denmark
ERR2228919	Denmark
ERR2228920	Denmark
ERR2228921	Denmark
ERR2228922	Denmark
ERR2228923	Denmark
ERR2228924	Denmark
ERR2228925	Denmark
ERR2228926	Denmark
ERR2228927	Denmark
ERR2228928	Denmark
ERR2228929	Denmark
ERR2228930	Denmark
ERR2228931	Denmark
ERR2228932	Denmark
ERR2228933	Denmark

ERR2228934	Denmark
ERR2228935	Denmark
ERR2228936	Denmark
ERR2228937	Denmark
ERR2228938	Denmark
ERR2228939	Denmark
ERR2228940	Denmark
ERR2228941	Denmark
ERR2228942	Denmark
ERR2228943	Denmark
ERR2228944	Denmark
ERR2228945	Denmark
ERR2228946	Denmark
ERR2228947	Denmark
ERR2228948	Denmark
ERR2228949	Denmark
ERR2228950	Denmark
ERR2228951	Denmark
ERR2228952	Denmark
ERR2228953	Denmark
ERR2228954	Denmark
ERR2228955	Denmark
ERR2228956	Denmark
ERR2228957	Denmark
ERR2228958	Denmark
ERR2228959	Denmark
ERR2228960	Denmark
ERR2228961	Denmark
ERR2228962	Denmark
ERR2228963	Denmark
ERR2228964	Denmark
ERR2228965	Denmark
ERR2228966	Denmark
ERR2228967	Denmark
ERR2228968	Denmark
ERR2228969	Denmark
ERR2228970	Denmark
ERR2228971	Denmark
ERR2228972	Denmark
ERR2228973	Denmark
ERR2228974	Denmark
ERR2228975	Denmark
ERR2228976	Denmark
ERR2228977	Denmark
ERR2228978	Denmark
ERR2228979	Denmark
ERR2228980	Denmark
ERR2228981	Denmark
ERR2228982	Denmark
ERR2228983	Denmark

ERR2228984	Denmark
ERR2228985	Denmark
ERR2228986	Denmark
ERR2228987	Denmark
ERR2228988	Denmark
ERR2228989	Denmark
ERR2228990	Denmark
ERR2228991	Denmark
ERR2228992	Denmark
ERR2228993	Denmark
ERR2228994	Denmark
ERR2228995	Denmark
ERR2228996	Denmark
ERR2228997	Denmark
ERR2228998	Denmark
ERR2228999	Denmark
ERR2229000	Denmark
ERR2229001	Denmark
ERR2229002	Denmark
ERR2229003	Denmark
ERR2229004	Denmark
ERR2229005	Denmark
ERR2229006	Denmark
ERR2229007	Denmark
ERR2229008	Denmark
ERR2229009	Denmark
ERR2229010	Denmark
ERR2229011	Denmark
ERR2229012	Denmark
ERR2229013	Denmark
ERR2229014	Denmark
ERR2229015	Denmark
ERR2229016	Denmark
ERR2229017	Denmark
ERR2229018	Denmark
ERR2229019	Denmark
ERR2229020	Denmark
ERR2229021	Denmark
ERR2229022	Denmark
ERR2229023	Denmark
ERR2229024	Denmark
ERR2229025	Denmark
ERR2229026	Denmark
ERR2229027	Denmark
ERR2229028	Denmark
ERR2229029	Denmark
ERR2229030	Denmark
ERR2229031	Denmark
ERR2229032	Denmark
ERR2229033	Denmark

ERR2229034	Denmark
ERR2229035	Denmark
ERR2229036	Denmark
ERR2229037	Denmark
ERR2229038	Denmark
ERR2229039	Denmark
ERR2229040	Denmark
ERR2229041	Denmark
ERR2229042	Denmark
ERR2229043	Denmark
ERR2229044	Denmark
ERR2229045	Denmark
ERR2229046	Denmark
ERR2229047	Denmark
ERR2229048	Denmark
ERR2229049	Denmark
ERR2229050	Denmark
ERR2229051	Denmark
ERR2229052	Denmark
ERR2229053	Denmark
ERR2229054	Denmark
ERR2229055	Denmark
ERR2229056	Denmark
ERR2229057	Denmark
ERR2229058	Denmark
ERR2229059	Denmark
ERR2229060	Denmark
ERR2229061	Denmark
ERR2229062	Denmark
ERR2229063	Denmark
ERR2229064	Denmark
ERR2229065	Denmark
ERR2229066	Denmark
ERR2229067	Denmark
ERR2229068	Denmark
ERR2229069	Denmark
ERR2229070	Denmark
ERR2229071	Denmark
ERR2229072	Denmark
ERR2229073	Denmark
ERR2229074	Denmark
ERR2229075	Denmark
ERR2229076	Denmark
ERR2229077	Denmark
ERR2229078	Denmark
ERR2229079	Denmark
ERR2229080	Denmark
ERR2229081	Denmark
ERR2229082	Denmark
ERR2229083	Denmark

ERR2229084	Denmark
ERR2229085	Denmark
ERR2229086	Denmark
ERR2229087	Denmark
ERR2229088	Denmark
ERR2229089	Denmark
ERR2229090	Denmark
ERR2229091	Denmark
ERR2229092	Denmark
ERR2229093	Denmark
ERR2229094	Denmark
ERR2229095	Denmark
ERR2229096	Denmark
ERR2229097	Denmark
ERR2229098	Denmark
ERR2229099	Denmark
ERR2229100	Denmark
ERR2229101	Denmark
ERR2229102	Denmark
ERR2229103	Denmark
ERR2229104	Denmark
ERR2229105	Denmark
ERR2229106	Denmark
ERR2229107	Denmark
ERR2229108	Denmark
ERR2229109	Denmark
ERR2229110	Denmark
ERR2229111	Denmark
ERR2229112	Denmark
ERR2229113	Denmark
ERR2229114	Denmark
ERR2229115	Denmark
ERR2229116	Denmark
ERR2229117	Denmark
ERR2229118	Denmark
ERR2229119	Denmark
ERR2229120	Denmark
ERR2229121	Denmark
ERR2229122	Denmark
ERR2229123	Denmark
ERR2229124	Denmark
ERR2229125	Denmark
ERR2229126	Denmark
ERR2229127	Denmark
ERR2229128	Denmark
ERR2229129	Denmark
ERR2229130	Denmark
ERR2229131	Denmark
ERR2229132	Denmark
ERR2229133	Denmark

ERR2229134	Denmark
ERR2229135	Denmark
ERR2229136	Denmark
ERR2229137	Denmark
ERR2229138	Denmark
ERR2229139	Denmark
ERR2229140	Denmark
ERR2229141	Denmark
ERR2229142	Denmark
ERR2229143	Denmark
ERR2229144	Denmark
ERR2229145	Denmark
ERR2229146	Denmark
ERR2229147	Denmark
ERR2229148	Denmark
ERR2229149	Denmark
ERR2229150	Denmark
ERR2229151	Denmark
ERR2229152	Denmark
ERR2229153	Denmark
ERR2229154	Denmark
ERR2229155	Denmark
ERR2229156	Denmark
ERR2229157	Denmark
ERR2229158	Denmark
ERR2229159	Denmark
ERR2229160	Denmark
ERR2229161	Denmark
ERR2229162	Denmark
ERR2229163	Denmark
ERR2229164	Denmark
ERR2229165	Denmark
ERR2229166	Denmark
ERR2229167	Denmark
ERR2229168	Denmark
ERR2229169	Denmark
ERR2229170	Denmark
ERR2229171	Denmark
ERR2229172	Denmark
ERR2229173	Denmark
ERR2229174	Denmark
ERR2229175	Denmark
ERR2229176	Denmark
ERR2229177	Denmark
ERR2229178	Denmark
ERR2229179	Denmark
ERR2229180	Denmark
ERR2229181	Denmark
ERR2229182	Denmark
ERR2229183	Denmark

ERR2229184	Denmark
ERR2229185	Denmark
ERR2229186	Denmark
ERR2229187	Denmark
ERR2229188	Denmark
ERR2229189	Denmark
ERR2229190	Denmark
ERR2229191	Denmark
ERR2229192	Denmark
ERR2229193	Denmark
ERR2229194	Denmark
ERR2229195	Denmark
ERR2229196	Denmark
ERR2229197	Denmark
ERR2229198	Denmark
ERR2229199	Denmark
ERR2229200	Denmark
ERR2229201	Denmark
ERR2229202	Denmark
ERR2229203	Denmark
ERR2229204	Denmark
ERR2229205	Denmark
ERR2229206	Denmark
ERR2229207	Denmark
ERR2229208	Denmark
ERR2229209	Denmark
ERR2229210	Denmark
ERR2229211	Denmark
ERR2229212	Denmark
ERR2229213	Denmark
ERR2229214	Denmark
ERR2229215	Denmark
ERR2229216	Denmark
ERR2229217	Denmark
ERR2229218	Denmark
ERR2229219	Denmark
ERR2229220	Denmark
ERR2229221	Denmark
ERR2229222	Denmark
ERR2229223	Denmark
ERR2229224	Denmark
ERR2229225	Denmark
ERR2229226	Denmark
ERR2229227	Denmark
ERR2229228	Denmark
ERR2229229	Denmark
ERR2229230	Denmark
ERR2229231	Denmark
ERR2229232	Denmark
ERR2229233	Denmark

ERR2229234	Denmark
ERR2229235	Denmark
ERR2229236	Denmark
ERR2229237	Denmark
ERR2229238	Denmark
ERR2229239	Denmark
ERR2229240	Denmark
ERR2229241	Denmark
ERR2229242	Denmark
ERR2229243	Denmark
ERR2229244	Denmark
ERR2229245	Denmark
ERR2229246	Denmark
ERR2229247	Denmark
ERR2229248	Denmark
ERR2229249	Denmark
ERR2229250	Denmark
ERR2229251	Denmark
ERR2229252	Denmark
ERR2229253	Denmark
ERR2229254	Denmark
ERR2229255	Denmark
ERR2229256	Denmark
ERR2229257	Denmark
ERR2229258	Denmark
ERR2229259	Denmark
ERR2229260	Denmark
ERR2229261	Denmark
ERR2229262	Denmark
ERR2229263	Denmark
ERR2229264	Denmark
ERR2229265	Denmark
ERR2229266	Denmark
ERR2229267	Denmark
ERR2229268	Denmark
ERR2229269	Denmark
ERR2229270	Denmark
ERR2229271	Denmark
ERR2229272	Denmark
ERR2229273	Denmark
ERR2229274	Denmark
ERR2229275	Denmark
ERR2229276	Denmark
ERR2229277	Denmark
ERR2229278	Denmark
ERR2229279	Denmark
ERR2229280	Denmark
ERR2229281	Denmark
ERR2229282	Denmark
ERR2229283	Denmark

ERR2229284	Denmark
ERR2229285	Denmark
ERR2229286	Denmark
ERR2229287	Denmark
ERR2229288	Denmark
ERR2229289	Denmark
ERR2229290	Denmark
ERR2229291	Denmark
ERR2229292	Denmark
ERR2229293	Denmark
ERR2229294	Denmark
ERR2229295	Denmark
ERR2229296	Denmark
ERR2229297	Denmark
ERR2229298	Denmark
ERR2229299	Denmark
ERR2229300	Denmark
ERR2229301	Denmark
ERR2229302	Denmark
ERR2229303	Denmark
ERR2229304	Denmark
ERR2229305	Denmark
ERR2229306	Denmark
ERR2229307	Denmark
ERR2229308	Denmark
ERR2229309	Denmark
ERR2229310	Denmark
ERR2229311	Denmark
ERR2229312	Denmark
ERR2229313	Denmark
ERR2229314	Denmark
ERR2229315	Denmark
ERR2229316	Denmark
ERR2229317	Denmark
ERR2229318	Denmark
ERR2229319	Denmark
ERR2229320	Denmark
ERR2229321	Denmark
ERR2229322	Denmark
ERR2229323	Denmark
ERR2229324	Denmark
ERR2229325	Denmark
ERR2229326	Denmark
ERR2229327	Denmark
ERR2229328	Denmark
ERR2229329	Denmark
ERR2229330	Denmark
ERR2229331	Denmark
ERR2229332	Denmark
ERR2229333	Denmark

ERR2229334	Denmark
ERR2229335	Denmark
ERR2229336	Denmark
ERR2229337	Denmark
ERR2229338	Denmark
ERR2229339	Denmark
ERR2229340	Denmark
ERR2229341	Denmark
ERR2229342	Denmark
ERR2229343	Denmark
ERR2229344	Denmark
ERR2229345	Denmark
ERR2229346	Denmark
ERR2229347	Denmark
ERR2229348	Denmark
ERR2229349	Denmark
ERR2229350	Denmark
ERR2229351	Denmark
ERR2229352	Denmark
ERR2229353	Denmark
ERR2229354	Denmark
ERR2229355	Denmark
ERR2229356	Denmark
ERR2229357	Denmark
ERR2229358	Denmark
ERR2229359	Denmark
ERR2229360	Denmark
ERR2229361	Denmark
ERR2229362	Denmark
ERR2229363	Denmark
ERR2229364	Denmark
ERR2229365	Denmark
ERR2229366	Denmark
ERR2229367	Denmark
ERR2229368	Denmark
ERR2229369	Denmark
ERR2229370	Denmark
ERR2229371	Denmark
ERR2229372	Denmark
ERR2229373	Denmark
ERR2229374	Denmark
ERR2229375	Denmark
ERR2229376	Denmark
ERR2229377	Denmark
ERR2229378	Denmark
ERR2229379	Denmark
ERR2229380	Denmark
ERR2229381	Denmark
ERR2229382	Denmark
ERR2229383	Denmark

ERR2229384	Denmark
ERR2229385	Denmark
ERR2229386	Denmark
ERR2229387	Denmark
ERR2229388	Denmark
ERR2229389	Denmark
ERR2229390	Denmark
ERR2229391	Denmark
ERR2229392	Denmark
ERR2229393	Denmark
ERR2229394	Denmark
ERR2229395	Denmark
ERR2229396	Denmark
ERR2229397	Denmark
ERR2229398	Denmark
ERR2229399	Denmark
ERR2229400	Denmark
ERR2229401	Denmark
ERR2229402	Denmark
ERR2229403	Denmark
ERR2229404	Denmark
ERR2229405	Denmark
ERR2229406	Denmark
ERR2229407	Denmark
ERR2229408	Denmark
ERR2229409	Denmark
ERR2229410	Denmark
ERR2229411	Denmark
ERR2229412	Denmark
ERR2229413	Denmark
ERR2229414	Denmark
ERR2229415	Denmark
ERR2229416	Denmark
ERR2229417	Denmark
ERR2229418	Denmark
ERR2229419	Denmark
ERR2229420	Denmark
ERR2229421	Denmark
ERR2229422	Denmark
ERR2229423	Denmark
ERR2229424	Denmark
ERR2229425	Denmark
ERR2229426	Denmark
ERR2229427	Denmark
ERR2229428	Denmark
ERR2229429	Denmark
ERR2229430	Denmark
ERR2229431	Denmark
ERR2229432	Denmark
ERR2229433	Denmark

ERR2229434	Denmark
ERR2229435	Denmark
ERR2229436	Denmark
ERR2229437	Denmark
ERR2229438	Denmark
ERR2229439	Denmark
ERR2229440	Denmark
ERR2229441	Denmark
ERR2229442	Denmark
ERR2229443	Denmark
ERR2229444	Denmark
ERR2229445	Denmark
ERR2229446	Denmark
ERR2229447	Denmark
ERR2229448	Denmark
ERR2229449	Denmark
ERR2229450	Denmark
ERR2229451	Denmark
ERR2229452	Denmark
ERR2229453	Denmark
ERR2229454	Denmark
ERR2229455	Denmark
ERR2229456	Denmark
ERR2229457	Denmark
ERR2229458	Denmark
ERR2229459	Denmark
ERR2229460	Denmark
ERR2229461	Denmark
ERR2229462	Denmark
ERR2229463	Denmark
ERR2229464	Denmark
ERR2229465	Denmark
ERR2229466	Denmark
ERR2229467	Denmark
ERR2229468	Denmark
ERR2229469	Denmark
ERR2229470	Denmark
ERR2229471	NA
ERR2229472	NA
ERR2229473	NA
ERR2229474	NA
ERR2229475	NA
ERR2229476	NA
ERR2229477	NA
ERR2229478	NA
ERR2229479	NA
ERR2229480	NA
ERR2229481	NA
ERR2229482	NA
ERR2229483	NA

ERR2229484	NA
ERR2229485	NA
ERR2229486	NA
ERR2229487	NA
ERR2229488	NA
ERR2229489	NA
ERR2229490	NA
ERR2229491	NA
ERR2229492	NA
ERR2229493	NA
ERR2229494	NA
ERR2229495	NA
ERR2229496	NA
ERR2229497	NA
ERR2229498	NA
ERR2229499	NA
ERR2229500	NA
ERR2229501	NA
ERR2229502	NA
ERR2229503	NA
ERR2229504	NA
ERR2229505	NA
ERR2229506	NA
ERR2229507	NA
ERR2229508	NA
ERR2229509	NA
ERR2229510	NA
ERR2229511	NA
ERR2229512	NA
ERR2229513	NA
ERR2229514	NA
ERR2229515	NA
ERR2229516	NA
ERR2229517	NA
ERR2229518	NA
ERR2229519	NA
ERR2229520	NA
ERR2229521	NA
ERR2229522	NA
ERR2229523	NA
ERR2229524	NA
ERR2229525	NA
ERR2229526	NA
ERR2229527	NA
ERR2229528	NA
ERR2229529	NA
ERR2229530	NA
ERR2229531	NA
ERR2229532	NA
ERR2229533	NA

ERR2229534	NA
ERR2229535	NA
ERR2229536	NA
ERR2229537	NA
ERR2229538	NA
ERR2229539	NA
ERR2229540	NA
ERR2229541	NA
ERR2229542	NA
ERR2229543	NA
ERR2229544	NA
ERR2229545	NA
ERR2229546	NA
ERR2229547	NA
ERR2229548	NA
ERR2229549	NA
ERR2229550	NA
ERR2229551	NA
ERR2229552	NA
ERR2229553	NA
ERR2229554	NA
ERR2229555	NA
ERR2229556	NA
ERR2229557	NA
ERR2229558	NA
ERR2229559	NA
ERR2229560	NA
ERR2229561	NA
ERR2229562	NA
ERR2229563	NA
ERR2229564	NA
ERR2229565	NA
ERR2229566	NA
ERR2229567	NA
ERR2229568	NA
ERR2229569	NA
ERR2229570	NA
ERR2229571	NA
ERR2229572	NA
ERR2229573	NA
ERR2229574	NA
ERR2229575	NA
ERR2229576	NA
ERR2229577	NA
ERR2229578	NA
ERR2229579	NA
ERR2229580	NA
ERR2229581	NA
ERR2229582	NA
ERR2229583	NA

ERR2229584	NA
ERR2229585	NA
ERR2229586	NA
ERR2229587	Denmark
ERR2229588	Denmark
ERR2229589	Denmark
ERR2229590	Denmark
ERR2229591	Denmark
ERR2229592	Denmark
ERR2229593	Denmark
ERR2229594	Denmark
ERR2229595	Denmark
ERR2229596	Denmark
ERR2229597	Denmark
ERR2229598	Denmark
ERR2229599	Denmark
ERR2229600	Denmark
ERR2229601	Denmark
ERR2229602	Denmark
ERR2229603	Denmark
ERR2229604	Denmark
ERR2229605	Denmark
ERR2229606	Denmark
ERR2229607	Denmark
ERR2229608	Denmark
ERR2229609	Denmark
ERR2229610	Denmark
ERR2229611	Denmark
ERR2229612	Denmark
ERR2229613	Denmark
ERR2229614	Denmark
ERR2229615	Denmark
ERR2229616	Denmark
ERR2229617	Denmark
ERR2229618	Denmark
ERR2229619	Denmark
ERR2229620	Denmark
ERR2229621	Denmark
ERR2229622	Denmark
ERR2229623	Denmark
ERR2229624	Denmark
ERR2229625	Denmark
ERR2229626	Denmark
ERR2229627	Denmark
ERR2229628	Denmark
ERR2229629	Denmark
ERR2229630	Denmark
ERR2229631	Denmark
ERR2229632	Denmark
ERR2229633	Denmark

ERR2229634	Denmark
ERR2229635	Denmark
ERR2229636	Denmark
ERR2229637	Denmark
ERR2229638	Denmark
ERR2229639	Denmark
ERR2229640	Denmark
ERR2229641	Denmark
ERR2229642	Denmark
ERR2229643	Denmark
ERR2229644	Denmark
ERR2229645	Denmark
ERR2229646	Denmark
ERR2229647	Denmark
ERR2229648	Denmark
ERR2229649	Denmark
ERR2229650	Denmark
ERR2229651	Denmark
ERR2229652	Denmark
ERR2229653	Denmark
ERR2229654	Denmark
ERR2229655	Denmark
ERR2229656	Denmark
ERR2229657	Denmark
ERR2229658	Denmark
ERR2229659	Denmark
ERR2229660	Denmark
ERR2229661	Denmark
ERR2229662	Denmark
ERR2229663	Denmark
ERR2229664	Denmark
ERR2229665	Denmark
ERR2229666	Denmark
ERR2229667	Denmark
ERR2229668	Denmark
ERR2229669	Denmark
ERR2229670	Denmark
ERR2229671	Denmark
ERR2229672	Denmark
ERR2229673	Denmark
ERR2229674	Denmark
ERR2229675	Denmark
ERR2229676	Denmark
ERR2229677	Denmark
ERR2229678	Denmark
ERR2229679	Denmark
ERR2229680	Denmark
ERR2229681	Denmark
ERR2229682	Denmark
ERR2229683	Denmark

ERR2229684	Denmark
ERR2229685	Denmark
ERR2229686	Denmark
ERR2229687	Denmark
ERR2229688	Denmark
ERR2229689	Denmark
ERR2229690	Denmark
ERR2229691	Denmark
ERR2229692	Denmark
ERR2229693	Denmark
ERR2229694	Denmark
ERR2229695	Denmark
ERR2229696	Denmark
ERR2229697	Denmark
ERR2229698	Denmark
ERR2229699	Denmark
ERR2229700	Denmark
ERR2229701	Denmark
ERR2229702	Denmark
ERR2229703	Denmark
ERR2229704	Denmark
ERR2229705	Denmark
ERR2229706	Denmark
ERR2229707	Denmark
ERR2229708	Denmark
ERR2229709	Denmark
ERR2229710	Denmark
ERR2229711	Denmark
ERR2229712	Denmark
ERR2229713	Denmark
ERR2229714	Denmark
ERR2229715	Denmark
ERR2229716	Denmark
ERR2229717	Denmark
ERR2229718	Denmark
ERR2229719	Denmark
ERR2229720	Denmark
ERR2229721	Denmark
ERR2229722	Denmark
ERR2229723	Denmark
ERR2229724	Denmark
ERR2229725	Denmark
ERR2229726	Denmark
ERR2229727	Denmark
ERR2229728	Denmark
ERR2229729	Denmark
ERR2229730	Denmark
ERR2229731	Denmark
ERR2229732	Denmark
ERR2229733	Denmark

ERR2229734	Denmark
ERR2229735	Denmark
ERR2229736	Denmark
ERR2229737	Denmark
ERR2229738	Denmark
ERR2229739	Denmark
ERR2229740	Denmark
ERR2229741	Denmark
ERR2229742	Denmark
ERR2229743	Denmark
ERR2229744	Denmark
ERR2229745	Denmark
ERR2229746	Denmark
ERR2229747	Denmark
ERR2229748	Denmark
ERR2229749	Denmark
ERR2229750	Denmark
ERR2229751	Denmark
ERR2229752	Denmark
ERR2229753	Denmark
ERR2229754	Denmark
ERR2229755	Denmark
ERR2229756	Denmark
ERR2229757	Denmark
ERR2229758	Denmark
ERR2229759	Denmark
ERR2229760	Denmark
ERR2229761	Denmark
ERR2229762	Denmark
ERR2229763	Denmark
ERR2229764	Denmark
ERR2229765	Denmark
ERR2229766	Denmark
ERR2229767	Denmark
ERR2229768	Denmark
ERR2229769	Denmark
ERR2229770	Denmark
ERR2229771	Denmark
ERR2229772	Denmark
ERR2229773	Denmark
ERR2229774	Denmark
ERR2229775	Denmark
ERR2229776	Denmark
ERR2229777	Denmark
ERR2229778	Denmark
ERR2229779	Denmark
ERR2229780	Denmark
ERR2229781	Denmark
ERR2229782	Denmark
ERR2229783	Denmark

ERR2229784	Denmark
ERR2229785	Denmark
ERR2229786	Denmark
ERR2229787	Denmark
ERR2229788	Denmark
ERR2229789	Denmark
ERR2229790	Denmark
ERR2229791	Denmark
ERR2229792	Denmark
ERR2229793	Denmark
ERR2229794	Denmark
ERR2229795	Denmark
ERR2229796	Denmark
ERR2229797	Denmark
ERR2229798	Denmark
ERR2229799	Denmark
ERR2229800	Denmark
ERR2229801	Denmark
ERR2229802	Denmark
ERR2229803	Denmark
ERR2229804	Denmark
ERR2229805	Denmark
ERR2229806	Denmark
ERR2229807	Denmark
ERR2229808	Denmark
ERR2229809	Denmark
ERR2229810	Denmark
ERR2229811	Denmark
ERR2229812	Denmark
ERR2229813	Denmark
ERR2229814	Denmark
ERR2229815	Denmark
ERR2229816	Denmark
ERR2229817	Denmark
ERR2229818	Denmark
ERR2229819	Denmark
ERR2229820	Denmark
ERR2245275	Norway
ERR2245276	Norway
ERR2245277	Norway
ERR2245278	Norway
ERR2245279	Norway
ERR2245280	Norway
ERR2245281	Norway
ERR2245282	Norway
ERR2245283	Norway
ERR2245284	Norway
ERR2245285	Norway
ERR2245286	Norway
ERR2245287	Norway

ERR2245288	Norway
ERR2245289	Norway
ERR2245290	Norway
ERR2245291	Norway
ERR2245292	Norway
ERR2245293	Norway
ERR2245294	Norway
ERR2245295	Norway
ERR2245296	Norway
ERR2245297	Norway
ERR2245298	Norway
ERR2245299	Norway
ERR2245300	Norway
ERR2245301	Norway
ERR2245302	Norway
ERR2245303	Norway
ERR2245304	Norway
ERR2245305	Norway
ERR2245306	Norway
ERR2245307	Norway
ERR2245332	Norway
ERR2245333	Norway
ERR2245334	Norway
ERR2245335	Norway
ERR2245336	Norway
ERR2245337	Norway
ERR2245338	Norway
ERR2245339	Norway
ERR2245340	Norway
ERR2245341	Norway
ERR2245342	Norway
ERR2245343	Norway
ERR2245344	Norway
ERR2245345	Norway
ERR2245346	Norway
ERR2245347	Norway
ERR2245348	Norway
ERR2245349	Norway
ERR2245350	Norway
ERR2245351	Norway
ERR2245352	Norway
ERR2245353	Norway
ERR2245354	Norway
ERR2245355	Norway
ERR2245356	Norway
ERR2245357	Norway
ERR2245358	Norway
ERR2245359	Norway
ERR2245360	Norway
ERR2245361	Norway

ERR2245362	Norway
ERR2245363	Norway
ERR2245364	Norway
ERR2245365	Norway
ERR2245366	Norway
ERR2245367	Norway
ERR2245372	Norway
ERR2245373	Norway
ERR2245374	Norway
ERR2245375	Norway
ERR2245376	Norway
ERR2245377	Norway
ERR2245378	Norway
ERR2245379	Norway
ERR2245380	Norway
ERR2245381	Norway
ERR2245382	Norway
ERR2245383	Norway
ERR2245384	Norway
ERR2245385	Norway
ERR2245386	Norway
ERR2245387	Norway
ERR2245388	Norway
ERR2245389	Norway
ERR2245390	Norway
ERR2245391	Norway
ERR2245392	Norway
ERR2245393	Norway
ERR2245394	Norway
ERR2245395	Norway
ERR2245396	Norway
ERR2245397	Norway
ERR2245398	Norway
ERR2245399	Norway
ERR2245400	Norway
ERR2245401	Norway
ERR2245402	Norway
ERR2245403	Norway
ERR2245404	Norway
ERR2245405	Norway
ERR2245406	Norway
ERR2245407	Norway
ERR2245408	Norway
ERR2245409	Norway
ERR2245410	Norway
ERR2245411	Norway
ERR2245412	Norway
ERR2245413	Norway
ERR2245414	Norway
ERR2245415	Norway

ERR2245416	Norway
ERR2245417	Norway
ERR2245418	Norway
ERR2245419	Norway
ERR2245420	Norway
ERR2245421	Norway
ERR2245422	Norway
ERR2245423	Norway
ERR2245424	Norway
ERR2245425	Norway
ERR2245426	Norway
ERR2245427	Norway
ERR227974	NA
ERR227975	Russia
ERR227976	Russia
ERR227977	Russia
ERR227978	Russia
ERR227979	Russia
ERR227980	Russia
ERR227981	Russia
ERR227982	Russia
ERR227983	Russia
ERR227984	Russia
ERR227985	Russia
ERR227986	NA
ERR227987	Russia
ERR227988	Russia
ERR227989	NA
ERR227990	Russia
ERR227991	Russia
ERR227992	Russia
ERR227993	NA
ERR227994	Russia
ERR227995	Russia
ERR227996	Russia
ERR227997	Russia
ERR227998	Russia
ERR227999	Russia
ERR228000	Russia
ERR228001	Russia
ERR228002	Russia
ERR228003	Russia
ERR228004	Russia
ERR228005	Russia
ERR228006	Russia
ERR228007	Russia
ERR228008	Russia
ERR228009	Russia
ERR228010	Russia
ERR228011	Russia

ERR228012	Russia
ERR228013	Russia
ERR228014	Russia
ERR228015	Russia
ERR228016	Russia
ERR228017	Russia
ERR228018	Russia
ERR228019	Russia
ERR228020	Russia
ERR228021	Russia
ERR228022	Russia
ERR228023	Russia
ERR228024	Russia
ERR228025	Russia
ERR228026	Russia
ERR228027	Russia
ERR228028	Russia
ERR228029	Russia
ERR228030	Russia
ERR228031	Russia
ERR228032	Russia
ERR228033	Russia
ERR228034	Russia
ERR228035	Russia
ERR228036	Russia
ERR228037	Russia
ERR228038	Russia
ERR228039	Russia
ERR228040	Russia
ERR228041	NA
ERR228042	Russia
ERR228043	Russia
ERR228044	Russia
ERR228045	Russia
ERR228046	Russia
ERR228047	Russia
ERR228048	Russia
ERR228049	Russia
ERR228050	Russia
ERR228051	Russia
ERR228052	Russia
ERR228053	Russia
ERR228054	Russia
ERR228055	Russia
ERR228056	Russia
ERR228057	Russia
ERR228058	Russia
ERR228059	Russia
ERR228060	Russia
ERR228061	Russia

ERR228062	Russia
ERR228063	Russia
ERR228064	Russia
ERR228065	Russia
ERR228066	Russia
ERR228067	Russia
ERR228068	Russia
ERR228069	Russia
ERR228102	NA
ERR228103	NA
ERR228104	NA
ERR228105	NA
ERR228106	NA
ERR228107	NA
ERR228108	NA
ERR228109	NA
ERR228110	NA
ERR228111	NA
ERR228112	NA
ERR228113	NA
ERR228114	NA
ERR228115	NA
ERR228116	NA
ERR228117	NA
ERR228118	NA
ERR228119	NA
ERR228120	NA
ERR228121	NA
ERR228122	NA
ERR228123	NA
ERR228124	NA
ERR228125	NA
ERR228126	NA
ERR228127	NA
ERR228128	NA
ERR228129	NA
ERR228130	NA
ERR228131	NA
ERR228132	NA
ERR228133	NA
ERR228134	NA
ERR228135	NA
ERR228136	NA
ERR228137	NA
ERR228138	NA
ERR228139	NA
ERR228140	NA
ERR228141	NA
ERR228142	NA
ERR228143	NA

ERR228144	NA
ERR228145	NA
ERR228146	NA
ERR228147	NA
ERR228148	NA
ERR228149	NA
ERR228150	NA
ERR228151	NA
ERR228152	NA
ERR228153	NA
ERR228154	NA
ERR228155	NA
ERR228156	NA
ERR228157	NA
ERR228158	NA
ERR228159	NA
ERR228160	NA
ERR228161	NA
ERR228162	NA
ERR228163	NA
ERR228164	NA
ERR228165	NA
ERR228166	NA
ERR228167	NA
ERR228168	NA
ERR228169	NA
ERR228170	NA
ERR228171	NA
ERR228172	NA
ERR228173	NA
ERR228174	NA
ERR228175	NA
ERR228176	NA
ERR228177	NA
ERR228178	NA
ERR228179	NA
ERR228180	NA
ERR228181	NA
ERR228182	NA
ERR228183	NA
ERR228184	NA
ERR228185	NA
ERR228186	NA
ERR228187	NA
ERR228188	NA
ERR228189	NA
ERR228190	NA
ERR228191	NA
ERR228192	NA
ERR228193	NA

ERR228194	NA
ERR228195	NA
ERR228196	NA
ERR228197	NA
ERR228198	NA
ERR228199	NA
ERR228200	NA
ERR228201	NA
ERR228202	NA
ERR228203	NA
ERR228204	NA
ERR228205	NA
ERR228206	NA
ERR228207	NA
ERR228208	NA
ERR228209	NA
ERR228210	NA
ERR228211	NA
ERR228212	NA
ERR228213	NA
ERR228214	NA
ERR228215	NA
ERR228216	NA
ERR228217	NA
ERR228218	NA
ERR228219	NA
ERR228220	NA
ERR228221	NA
ERR228222	NA
ERR228223	NA
ERR228224	NA
ERR228225	NA
ERR228226	NA
ERR228227	NA
ERR228228	NA
ERR228229	NA
ERR228230	NA
ERR228231	NA
ERR228232	NA
ERR228233	NA
ERR228234	NA
ERR228235	NA
ERR228236	NA
ERR228237	NA
ERR228238	NA
ERR228239	NA
ERR228240	NA
ERR228241	NA
ERR228242	NA
ERR228243	NA

ERR228244	NA
ERR228245	NA
ERR228246	NA
ERR228247	NA
ERR228248	NA
ERR228249	NA
ERR228250	NA
ERR228251	NA
ERR228252	NA
ERR228253	NA
ERR228254	NA
ERR228255	NA
ERR228256	NA
ERR228257	NA
ERR228258	NA
ERR228259	NA
ERR228260	NA
ERR228261	NA
ERR228262	NA
ERR228263	NA
ERR228264	NA
ERR228265	NA
ERR228266	NA
ERR228267	NA
ERR228268	NA
ERR228269	NA
ERR228270	NA
ERR228271	NA
ERR228272	NA
ERR228273	NA
ERR228274	NA
ERR228275	NA
ERR228276	NA
ERR228277	NA
ERR228278	NA
ERR228279	NA
ERR228280	NA
ERR228281	NA
ERR228282	NA
ERR228283	NA
ERR228284	NA
ERR228285	NA
ERR228286	NA
ERR228287	NA
ERR228288	NA
ERR228289	NA
ERR228290	NA
ERR228291	NA
ERR228292	NA
ERR228293	NA

ERR229915	Russia
ERR229916	Russia
ERR229917	Russia
ERR229918	Russia
ERR229919	Russia
ERR229920	Russia
ERR229921	Russia
ERR229922	Russia
ERR229923	Russia
ERR229924	Russia
ERR229925	Russia
ERR229926	Russia
ERR229927	Russia
ERR229928	Russia
ERR229929	Russia
ERR229930	NA
ERR229931	Russia
ERR229932	Russia
ERR229933	Russia
ERR229934	Russia
ERR229935	Russia
ERR229936	Russia
ERR229937	Russia
ERR229938	Russia
ERR229939	Russia
ERR229940	Russia
ERR229941	Russia
ERR229942	Russia
ERR229943	Russia
ERR229944	Russia
ERR229945	Russia
ERR229946	Russia
ERR229947	Russia
ERR229948	Russia
ERR229949	Russia
ERR229950	Russia
ERR229951	NA
ERR229952	NA
ERR229953	Russia
ERR229954	Russia
ERR229955	Russia
ERR229956	Russia
ERR229957	Russia
ERR229958	Russia
ERR229959	Russia
ERR229960	Russia
ERR229961	Russia
ERR229962	Russia
ERR229963	Russia
ERR229964	Russia

ERR229965	Russia
ERR229966	Russia
ERR229967	Russia
ERR229968	Russia
ERR229969	Russia
ERR229970	Russia
ERR229971	Russia
ERR229972	Russia
ERR229973	Russia
ERR229974	NA
ERR229975	Russia
ERR229976	Russia
ERR229977	Russia
ERR229978	Russia
ERR229979	Russia
ERR229980	Russia
ERR229981	Russia
ERR229982	Russia
ERR229983	Russia
ERR229984	Russia
ERR229985	Russia
ERR229986	Russia
ERR229987	Russia
ERR229988	Russia
ERR229989	Russia
ERR229990	Russia
ERR229991	Russia
ERR229992	Russia
ERR229993	Russia
ERR229994	Russia
ERR229995	Russia
ERR229996	NA
ERR229997	Russia
ERR229998	Russia
ERR229999	Russia
ERR230000	Russia
ERR230001	Russia
ERR230002	Russia
ERR230003	Russia
ERR230004	Russia
ERR230005	Russia
ERR230006	Russia
ERR230007	Russia
ERR230008	Russia
ERR230009	NA
ERR230010	Russia
ERR230018	NA
ERR230019	NA
ERR230020	NA
ERR230021	NA

ERR230022	NA
ERR230023	NA
ERR230024	NA
ERR230025	NA
ERR230026	NA
ERR230027	NA
ERR230028	NA
ERR230029	NA
ERR230030	NA
ERR230031	NA
ERR230032	NA
ERR230033	NA
ERR230034	NA
ERR230035	NA
ERR230036	NA
ERR230037	NA
ERR230038	NA
ERR230039	NA
ERR230040	NA
ERR230041	NA
ERR230042	NA
ERR230043	NA
ERR230044	NA
ERR230045	NA
ERR230046	NA
ERR230047	NA
ERR230048	NA
ERR230049	NA
ERR230050	NA
ERR230051	NA
ERR230052	NA
ERR230053	NA
ERR230054	NA
ERR230055	NA
ERR230056	NA
ERR230057	NA
ERR230058	NA
ERR230059	NA
ERR230060	NA
ERR230061	NA
ERR230062	NA
ERR230063	NA
ERR230064	NA
ERR230065	NA
ERR230066	NA
ERR230067	NA
ERR230068	NA
ERR230069	NA
ERR230070	NA
ERR230071	NA

ERR230072	NA
ERR230073	NA
ERR230074	NA
ERR230075	NA
ERR230076	NA
ERR230077	NA
ERR230078	NA
ERR230079	NA
ERR230080	NA
ERR230081	NA
ERR230082	NA
ERR230083	NA
ERR230084	NA
ERR230085	NA
ERR230086	NA
ERR230087	NA
ERR230088	NA
ERR230089	NA
ERR230090	NA
ERR230091	NA
ERR230092	NA
ERR230093	NA
ERR230094	NA
ERR230095	NA
ERR230096	NA
ERR230097	NA
ERR230098	NA
ERR230099	NA
ERR230100	NA
ERR230101	NA
ERR230102	NA
ERR230103	NA
ERR230104	NA
ERR230105	NA
ERR230106	NA
ERR230107	NA
ERR230108	NA
ERR230109	NA
ERR230110	NA
ERR230111	NA
ERR230112	NA
ERR230113	NA
ERR234097	NA
ERR234098	NA
ERR234099	NA
ERR234100	NA
ERR234101	NA
ERR234102	NA
ERR234103	NA
ERR234104	NA

ERR234105	NA
ERR234106	NA
ERR234107	NA
ERR234108	NA
ERR234109	NA
ERR234110	NA
ERR234111	NA
ERR234112	NA
ERR234113	NA
ERR234114	NA
ERR234115	NA
ERR234116	NA
ERR234117	NA
ERR234118	NA
ERR234119	NA
ERR234120	NA
ERR234121	NA
ERR234122	NA
ERR234123	NA
ERR234124	NA
ERR234125	NA
ERR234126	NA
ERR234127	NA
ERR234128	NA
ERR234129	NA
ERR234130	NA
ERR234131	NA
ERR234132	NA
ERR234133	NA
ERR234134	NA
ERR234135	NA
ERR234136	NA
ERR234137	NA
ERR234138	NA
ERR234139	NA
ERR234140	NA
ERR234141	NA
ERR234142	NA
ERR234143	NA
ERR234144	NA
ERR234145	NA
ERR234146	NA
ERR234147	NA
ERR234148	NA
ERR234149	NA
ERR234150	NA
ERR234151	NA
ERR234152	NA
ERR234153	NA
ERR234154	NA

ERR234155	NA
ERR234156	NA
ERR234157	NA
ERR234158	NA
ERR234159	NA
ERR234160	NA
ERR234161	NA
ERR234162	NA
ERR234163	NA
ERR234164	NA
ERR234165	NA
ERR234166	NA
ERR234167	NA
ERR234168	NA
ERR234169	NA
ERR234170	NA
ERR234171	NA
ERR234172	NA
ERR234173	NA
ERR234174	NA
ERR234175	NA
ERR234176	NA
ERR234177	NA
ERR234178	NA
ERR234179	NA
ERR234180	NA
ERR234181	NA
ERR234182	NA
ERR234183	NA
ERR234184	NA
ERR234185	NA
ERR234186	NA
ERR234187	NA
ERR234188	NA
ERR234189	NA
ERR234190	NA
ERR234191	NA
ERR234192	NA
ERR234193	NA
ERR234194	NA
ERR234195	NA
ERR234196	NA
ERR234197	NA
ERR234198	NA
ERR234199	NA
ERR234200	NA
ERR234201	NA
ERR234202	NA
ERR234203	NA
ERR234204	NA

ERR234205	NA
ERR234206	NA
ERR234207	NA
ERR234208	NA
ERR234209	NA
ERR234210	NA
ERR234211	NA
ERR234212	NA
ERR234213	NA
ERR234215	NA
ERR234216	NA
ERR234217	NA
ERR234224	NA
ERR234226	NA
ERR234232	NA
ERR234233	NA
ERR234234	NA
ERR234235	NA
ERR234236	NA
ERR234237	NA
ERR234238	NA
ERR234239	NA
ERR234240	NA
ERR234241	NA
ERR234242	NA
ERR234243	NA
ERR234244	NA
ERR234245	NA
ERR234246	NA
ERR234247	NA
ERR234248	NA
ERR234249	NA
ERR234250	NA
ERR234251	NA
ERR234252	NA
ERR234253	NA
ERR234254	NA
ERR234255	NA
ERR234256	NA
ERR234257	NA
ERR234258	NA
ERR234259	NA
ERR234260	NA
ERR234261	NA
ERR234262	NA
ERR234263	NA
ERR234264	NA
ERR234265	NA
ERR234266	NA
ERR234267	NA

ERR234268	NA
ERR234269	NA
ERR234270	NA
ERR234271	NA
ERR234272	NA
ERR234273	NA
ERR234556	Russia
ERR234557	Russia
ERR234558	Russia
ERR234559	Russia
ERR234560	Russia
ERR234561	Russia
ERR234562	Russia
ERR234563	Russia
ERR234564	Russia
ERR234565	Russia
ERR234566	NA
ERR234567	NA
ERR234568	Russia
ERR234569	Russia
ERR234570	Russia
ERR234571	NA
ERR234572	Russia
ERR234573	Russia
ERR234574	Russia
ERR234575	Russia
ERR234576	Russia
ERR234577	Russia
ERR234578	Russia
ERR234579	Russia
ERR234580	Russia
ERR234581	Russia
ERR234582	Russia
ERR234583	Russia
ERR234584	Russia
ERR234585	Russia
ERR234586	Russia
ERR234587	Russia
ERR234588	Russia
ERR234589	Russia
ERR234590	Russia
ERR234591	Russia
ERR234592	Russia
ERR234593	Russia
ERR234594	Russia
ERR234595	Russia
ERR234596	Russia
ERR234597	Russia
ERR234598	Russia
ERR234599	Russia

ERR234600	Russia
ERR234601	NA
ERR234602	Russia
ERR234603	Russia
ERR234604	NA
ERR234605	NA
ERR234606	Russia
ERR234607	Russia
ERR234608	Russia
ERR234609	Russia
ERR234610	Russia
ERR234611	NA
ERR234612	Russia
ERR234613	NA
ERR234614	Russia
ERR234615	Russia
ERR234616	Russia
ERR234617	Russia
ERR234618	Russia
ERR234619	Russia
ERR234620	Russia
ERR234621	NA
ERR234622	Russia
ERR234623	Russia
ERR234624	Russia
ERR234625	Russia
ERR234626	NA
ERR234627	Russia
ERR234628	Russia
ERR234629	NA
ERR234630	Russia
ERR234631	Russia
ERR234632	Russia
ERR234633	Russia
ERR234634	Russia
ERR234635	Russia
ERR234636	Russia
ERR234637	Russia
ERR234638	Russia
ERR234639	Russia
ERR234640	Russia
ERR234641	Russia
ERR234642	Russia
ERR234643	Russia
ERR234644	Russia
ERR234645	Russia
ERR234646	Russia
ERR234647	Russia
ERR234648	Russia
ERR234649	Russia

ERR234650	Russia
ERR234651	Russia
ERR234652	Russia
ERR234653	Russia
ERR234654	Russia
ERR234655	Russia
ERR234656	Russia
ERR234657	Russia
ERR234658	Russia
ERR234659	NA
ERR234660	Russia
ERR234661	Russia
ERR234662	Russia
ERR234663	Russia
ERR234664	Russia
ERR234665	Russia
ERR234666	Russia
ERR234667	Russia
ERR234668	Russia
ERR234669	Russia
ERR234670	NA
ERR234671	Russia
ERR234672	Russia
ERR234673	NA
ERR234674	Russia
ERR234675	NA
ERR234676	NA
ERR234677	NA
ERR234678	NA
ERR234679	NA
ERR234680	NA
ERR234681	NA
ERR234682	NA
ERR234683	Russia
ERR234684	Russia
ERR234685	Russia
ERR234686	Russia
ERR234687	Russia
ERR234688	Russia
ERR234689	Russia
ERR234690	Russia
ERR234691	Russia
ERR234692	Russia
ERR234693	Russia
ERR234694	Russia
ERR234695	Russia
ERR234696	Russia
ERR234697	Russia
ERR234698	Russia
ERR234699	Russia

ERR2432984	Brazil
ERR2432985	Brazil
ERR2432986	Brazil
ERR2432987	Brazil
ERR2432988	Brazil
ERR2432989	Brazil
ERR2432990	Brazil
ERR2432991	Brazil
ERR2432992	Brazil
ERR2432993	Brazil
ERR2432994	Brazil
ERR2432995	Brazil
ERR2432996	Brazil
ERR2432997	Brazil
ERR2432998	Brazil
ERR2432999	Brazil
ERR2433000	Brazil
ERR2433001	Brazil
ERR2433002	Brazil
ERR2433003	Brazil
ERR2433004	Brazil
ERR2433005	Brazil
ERR2433006	Brazil
ERR2433007	Brazil
ERR2433008	Brazil
ERR2433009	Brazil
ERR2433010	Brazil
ERR2433011	Brazil
ERR2433012	Brazil
ERR2433013	Brazil
ERR2451322	NA
ERR2451323	NA
ERR2451324	NA
ERR245646	Malawi
ERR245647	Malawi
ERR245648	Malawi
ERR245649	Malawi
ERR245650	Malawi
ERR245651	Malawi
ERR245652	Malawi
ERR245653	Malawi
ERR245654	Malawi
ERR245655	Malawi
ERR245656	Malawi
ERR245657	Malawi
ERR245658	Malawi
ERR245659	Malawi
ERR245660	Malawi
ERR245661	Malawi
ERR245662	Malawi

ERR245663	Malawi
ERR245664	Malawi
ERR245665	Malawi
ERR245666	Malawi
ERR245667	Malawi
ERR245668	Malawi
ERR245669	Malawi
ERR245670	Malawi
ERR245671	Malawi
ERR245672	Malawi
ERR245673	Malawi
ERR245674	Malawi
ERR245675	Malawi
ERR245676	Malawi
ERR245677	Malawi
ERR245678	Malawi
ERR245679	Malawi
ERR245680	Malawi
ERR245681	Malawi
ERR245682	Malawi
ERR245683	Malawi
ERR245684	Malawi
ERR245685	Malawi
ERR245686	Malawi
ERR245687	Malawi
ERR245688	Malawi
ERR245689	Malawi
ERR245690	Malawi
ERR245691	Malawi
ERR245692	Malawi
ERR245693	Malawi
ERR245694	Malawi
ERR245695	Malawi
ERR245696	Malawi
ERR245697	Malawi
ERR245698	Malawi
ERR245699	Malawi
ERR245700	Malawi
ERR245701	Malawi
ERR245702	Malawi
ERR245703	Malawi
ERR245704	Malawi
ERR245705	Malawi
ERR245706	Malawi
ERR245707	Malawi
ERR245708	Malawi
ERR245709	Malawi
ERR245710	Malawi
ERR245711	Malawi
ERR245712	Malawi

ERR245713	Malawi
ERR245714	Malawi
ERR245715	Malawi
ERR245716	Malawi
ERR245717	Malawi
ERR245718	Malawi
ERR245719	Malawi
ERR245720	Malawi
ERR245721	Malawi
ERR245722	Malawi
ERR245723	Malawi
ERR245724	Malawi
ERR245725	Malawi
ERR245726	Malawi
ERR245727	Malawi
ERR245728	Malawi
ERR245729	Malawi
ERR245730	Malawi
ERR245731	Malawi
ERR245732	Malawi
ERR245733	Malawi
ERR245734	Malawi
ERR245735	Malawi
ERR245736	Malawi
ERR245737	Malawi
ERR245738	Malawi
ERR245739	Malawi
ERR245740	Malawi
ERR245741	Malawi
ERR245742	Malawi
ERR245743	Malawi
ERR245744	Malawi
ERR245745	Malawi
ERR245746	Malawi
ERR245747	Malawi
ERR245748	Malawi
ERR245749	Malawi
ERR245750	Malawi
ERR245751	Malawi
ERR245752	Malawi
ERR245753	Malawi
ERR245754	Malawi
ERR245755	Malawi
ERR245756	Malawi
ERR245757	Malawi
ERR245758	Malawi
ERR245759	Malawi
ERR245760	Malawi
ERR245761	Malawi
ERR245762	Malawi

ERR245763	Malawi
ERR245764	Malawi
ERR245765	Malawi
ERR245766	Malawi
ERR245767	Malawi
ERR245768	Malawi
ERR245769	Malawi
ERR245770	Malawi
ERR245771	Malawi
ERR245772	Malawi
ERR245773	Malawi
ERR245774	Malawi
ERR245775	Malawi
ERR245776	Malawi
ERR245777	Malawi
ERR245778	Malawi
ERR245779	Malawi
ERR245780	Malawi
ERR245781	Malawi
ERR245782	Malawi
ERR245783	Malawi
ERR245784	Malawi
ERR245785	Malawi
ERR245786	Malawi
ERR245787	Malawi
ERR245788	Malawi
ERR245789	Malawi
ERR245790	Malawi
ERR245791	Malawi
ERR245792	Malawi
ERR245793	Malawi
ERR245794	Malawi
ERR245795	Malawi
ERR245796	Malawi
ERR245797	Malawi
ERR245798	Malawi
ERR245799	Malawi
ERR245800	Malawi
ERR245801	Malawi
ERR245802	Malawi
ERR245803	Malawi
ERR245804	Malawi
ERR245805	Malawi
ERR245806	Malawi
ERR245807	Malawi
ERR245808	Malawi
ERR245809	Malawi
ERR245810	Malawi
ERR245811	Malawi
ERR245812	Malawi

ERR245813	Malawi
ERR245814	Malawi
ERR245815	Malawi
ERR245816	Malawi
ERR245817	Malawi
ERR245818	Malawi
ERR245819	Malawi
ERR245820	Malawi
ERR245821	Malawi
ERR245822	Malawi
ERR245823	Malawi
ERR245824	Malawi
ERR245825	Malawi
ERR245826	Malawi
ERR245827	Malawi
ERR245828	Malawi
ERR245829	Malawi
ERR245830	Malawi
ERR245831	Malawi
ERR245832	Malawi
ERR245833	Malawi
ERR245834	Malawi
ERR245835	Malawi
ERR245836	Malawi
ERR245837	Malawi
ERR245838	Malawi
ERR245839	Malawi
ERR245840	Malawi
ERR245841	Malawi
ERR245842	Malawi
ERR245843	Malawi
ERR245844	Malawi
ERR245845	Malawi
ERR245846	Malawi
ERR245847	Malawi
ERR245848	Malawi
ERR245849	Malawi
ERR2486935	NA
ERR2486936	NA
ERR2486937	NA
ERR2486938	NA
ERR2486939	NA
ERR2486940	NA
ERR2486941	NA
ERR2486942	NA
ERR2486943	NA
ERR2486944	NA
ERR2486945	NA
ERR2486946	NA
ERR2486947	NA

ERR2486948	NA
ERR2486949	NA
ERR2486950	NA
ERR2486951	NA
ERR2486952	NA
ERR2486953	NA
ERR2486954	NA
ERR2486955	NA
ERR2486956	NA
ERR2486957	NA
ERR2486958	NA
ERR2486959	NA
ERR2486960	NA
ERR2486961	NA
ERR2486962	NA
ERR2486963	NA
ERR2486964	NA
ERR2486965	NA
ERR2486966	NA
ERR2486967	NA
ERR2486968	NA
ERR2486969	NA
ERR2486970	NA
ERR2486971	NA
ERR2486972	NA
ERR2486973	NA
ERR2486974	NA
ERR2486975	NA
ERR2486976	NA
ERR2486977	NA
ERR2486978	NA
ERR2486979	NA
ERR2486980	NA
ERR2486981	NA
ERR2486982	NA
ERR2486983	NA
ERR2486984	NA
ERR2486985	NA
ERR2486986	NA
ERR2503358	NA
ERR2503359	NA
ERR2503360	NA
ERR2503361	NA
ERR2503362	NA
ERR2503363	NA
ERR2503364	NA
ERR2503365	NA
ERR2503366	NA
ERR2503367	NA
ERR2503368	NA

ERR2503369	NA
ERR2503370	NA
ERR2503371	NA
ERR2503372	NA
ERR2503373	NA
ERR2503374	NA
ERR2503375	NA
ERR2503376	NA
ERR2503377	NA
ERR2503378	NA
ERR2503379	NA
ERR2503380	NA
ERR2503381	NA
ERR2503382	NA
ERR2503383	NA
ERR2503384	NA
ERR2503385	NA
ERR2503386	NA
ERR2503387	NA
ERR2503388	NA
ERR2503389	NA
ERR2503390	NA
ERR2503391	NA
ERR2503392	NA
ERR2503393	NA
ERR2503394	NA
ERR2503395	NA
ERR2503396	NA
ERR2503397	NA
ERR2503398	NA
ERR2503399	NA
ERR2503400	NA
ERR2503401	NA
ERR2503402	NA
ERR2503403	NA
ERR2503404	NA
ERR2503405	NA
ERR2503406	NA
ERR2503407	NA
ERR2503408	NA
ERR2503409	NA
ERR2503410	NA
ERR2503411	NA
ERR2503412	NA
ERR2503413	NA
ERR2503414	NA
ERR2503415	NA
ERR2503416	NA
ERR2503417	NA
ERR2503418	NA

ERR2503419	NA
ERR2503420	NA
ERR2503421	NA
ERR2503422	NA
ERR2503423	NA
ERR2503424	NA
ERR2503425	NA
ERR2503426	NA
ERR2503427	NA
ERR2503428	NA
ERR2503429	NA
ERR2503430	NA
ERR2503431	NA
ERR2503432	NA
ERR2503433	NA
ERR2503434	NA
ERR2503435	NA
ERR2503436	NA
ERR2503437	NA
ERR2503438	NA
ERR2503439	NA
ERR2503440	NA
ERR2503441	NA
ERR2503442	NA
ERR2503443	NA
ERR2503444	NA
ERR2503445	NA
ERR2503446	NA
ERR2503447	NA
ERR2503448	NA
ERR2503449	NA
ERR2503450	NA
ERR2503451	NA
ERR2503452	NA
ERR2503453	NA
ERR2503454	NA
ERR2503455	NA
ERR2503456	NA
ERR2503457	NA
ERR2503458	NA
ERR2503459	NA
ERR2503460	NA
ERR2503461	NA
ERR2503462	NA
ERR2503463	NA
ERR2503464	NA
ERR2503465	NA
ERR2503466	NA
ERR2503467	NA
ERR2503468	NA

ERR2503469	NA
ERR2503470	NA
ERR2503471	NA
ERR2503472	NA
ERR2503473	NA
ERR2503474	NA
ERR2503476	NA
ERR2503477	NA
ERR2503478	NA
ERR2503479	NA
ERR2503480	NA
ERR2503481	NA
ERR2503482	NA
ERR2503483	NA
ERR2503484	NA
ERR2503485	NA
ERR2503486	NA
ERR2503487	NA
ERR2503488	NA
ERR2503489	NA
ERR2503490	NA
ERR2503491	NA
ERR2503492	NA
ERR2503493	NA
ERR2503494	NA
ERR2503495	NA
ERR2503496	NA
ERR2503497	NA
ERR2503498	NA
ERR2503499	NA
ERR2503500	NA
ERR2503501	NA
ERR2503502	NA
ERR2503503	NA
ERR2503504	NA
ERR2503505	NA
ERR2503506	NA
ERR2503507	NA
ERR2503508	NA
ERR2503509	NA
ERR2503510	NA
ERR2503511	NA
ERR2503512	NA
ERR2503513	NA
ERR2503514	NA
ERR2503515	NA
ERR2503516	NA
ERR2503517	NA
ERR2503518	NA
ERR2503519	NA

ERR2503520	NA
ERR2503521	NA
ERR2503522	NA
ERR2503524	NA
ERR2503525	NA
ERR2503526	NA
ERR2503527	NA
ERR2503529	NA
ERR2503530	NA
ERR2503531	NA
ERR2503532	NA
ERR2503533	NA
ERR2503534	NA
ERR2503535	NA
ERR2503536	NA
ERR2503537	NA
ERR2503538	NA
ERR2503539	NA
ERR2503540	NA
ERR2503541	NA
ERR2503542	NA
ERR2503543	NA
ERR2503544	NA
ERR2503545	NA
ERR2503546	NA
ERR2503547	NA
ERR2503548	NA
ERR2503549	NA
ERR2503550	NA
ERR2503551	NA
ERR2503552	NA
ERR2503553	NA
ERR2503554	NA
ERR2503555	NA
ERR2503556	NA
ERR2503557	NA
ERR2503558	NA
ERR2503559	NA
ERR2503560	NA
ERR2503561	NA
ERR2503562	NA
ERR2503563	NA
ERR2503564	NA
ERR2503565	NA
ERR2503566	NA
ERR2503567	NA
ERR2503568	NA
ERR2503569	NA
ERR2512375	Norway
ERR2512376	Norway

ERR2512377	Norway
ERR2512378	Norway
ERR257880	Netherlands
ERR257881	Netherlands
ERR257882	Netherlands
ERR257883	Netherlands
ERR257884	Netherlands
ERR257885	Netherlands
ERR257886	Netherlands
ERR257887	Netherlands
ERR257888	Netherlands
ERR257889	Netherlands
ERR257890	Netherlands
ERR257891	Netherlands
ERR257892	Netherlands
ERR257893	Netherlands
ERR257894	Netherlands
ERR257895	Netherlands
ERR257896	Netherlands
ERR257897	Netherlands
ERR257898	Netherlands
ERR257899	Netherlands
ERR257900	Netherlands
ERR257901	Netherlands
ERR257902	Netherlands
ERR257903	Netherlands
ERR257904	Netherlands
ERR257905	Netherlands
ERR257906	Netherlands
ERR257907	Netherlands
ERR257908	Netherlands
ERR257909	Netherlands
ERR257910	Netherlands
ERR257911	Netherlands
ERR257912	Netherlands
ERR257913	Netherlands
ERR257914	Netherlands
ERR257915	Netherlands
ERR257916	Netherlands
ERR257917	Netherlands
ERR257918	Netherlands
ERR257919	Netherlands
ERR257920	Netherlands
ERR257921	Netherlands
ERR257922	Netherlands
ERR257923	Netherlands
ERR257924	Netherlands
ERR257925	Netherlands
ERR257926	Netherlands
ERR257927	Netherlands

ERR257928	Netherlands
ERR257929	Netherlands
ERR257930	Netherlands
ERR257931	Netherlands
ERR257932	Netherlands
ERR257933	Netherlands
ERR257934	Netherlands
ERR257935	Netherlands
ERR2652914	United States of America
ERR2652915	United States of America
ERR2652916	United States of America
ERR2652918	United States of America
ERR2652919	United States of America
ERR2652920	United States of America
ERR2652921	United States of America
ERR2652922	United States of America
ERR2652924	United States of America
ERR2652925	Canada
ERR2652926	Canada
ERR2652927	Canada
ERR2652928	Canada
ERR2652929	Canada
ERR2652930	Canada
ERR2652931	Canada
ERR2652932	Canada
ERR2652933	Canada
ERR2652934	Canada
ERR2652935	Canada
ERR2652936	Canada
ERR2652937	Canada
ERR2652938	Canada
ERR2652939	Canada
ERR2652940	Canada
ERR2652941	Canada
ERR2652942	Canada
ERR2652943	Canada
ERR2652944	Canada
ERR2652945	Canada
ERR2652946	Canada
ERR2652947	Canada
ERR2652948	Canada
ERR2652949	Canada
ERR2652950	Canada
ERR2652951	Canada
ERR2652952	Canada
ERR2652953	Canada
ERR2652954	Canada
ERR2652955	Canada
ERR2652956	Canada
ERR2652957	Canada

ERR2652958	Canada
ERR2652959	Canada
ERR2652960	Canada
ERR2652961	Canada
ERR2652962	Canada
ERR2652963	Canada
ERR2652964	Canada
ERR2652965	Canada
ERR2652966	Canada
ERR2652967	Canada
ERR2652968	Canada
ERR2652969	Canada
ERR2652970	Canada
ERR2652971	Canada
ERR2652972	Canada
ERR2652973	Canada
ERR2652974	Canada
ERR2652975	Canada
ERR2652976	Brazil
ERR2652977	Brazil
ERR2652978	Brazil
ERR2652979	Brazil
ERR2652980	Brazil
ERR2652981	Brazil
ERR2652982	Brazil
ERR2652983	Brazil
ERR2652984	Brazil
ERR2652985	Brazil
ERR2652986	Brazil
ERR2652987	Brazil
ERR2652988	Brazil
ERR2652989	Brazil
ERR2652990	Brazil
ERR2652991	Brazil
ERR2652992	Brazil
ERR2652993	Brazil
ERR2652994	Brazil
ERR2652995	Brazil
ERR2652996	Brazil
ERR2652997	Brazil
ERR2652998	Brazil
ERR2652999	Brazil
ERR2653000	Brazil
ERR2653001	Brazil
ERR2653002	Brazil
ERR2653003	Brazil
ERR2653004	Brazil
ERR2653005	Brazil
ERR2653006	Brazil
ERR2653007	Brazil

ERR2653008	Brazil
ERR2653009	Brazil
ERR2653010	Brazil
ERR2653011	Brazil
ERR2653012	Brazil
ERR2653013	Brazil
ERR2653014	Brazil
ERR2653015	Brazil
ERR2653016	Brazil
ERR2653017	Brazil
ERR2653018	Brazil
ERR2653019	Brazil
ERR2653020	Brazil
ERR2653021	Brazil
ERR2653022	Brazil
ERR2653023	Brazil
ERR2653024	Brazil
ERR2653025	Brazil
ERR2653026	Brazil
ERR2653027	Brazil
ERR2653028	Brazil
ERR2653029	Brazil
ERR2653030	Brazil
ERR2653031	Brazil
ERR2653032	Brazil
ERR2653033	Brazil
ERR2653034	Brazil
ERR2653035	Brazil
ERR2653036	Brazil
ERR2653037	Brazil
ERR2653038	Brazil
ERR2653039	Brazil
ERR2653040	Brazil
ERR2653041	Brazil
ERR2653042	Brazil
ERR2653043	Brazil
ERR2653044	Brazil
ERR2653045	Brazil
ERR2653046	Brazil
ERR2653047	Brazil
ERR2653048	Brazil
ERR2653049	Brazil
ERR2653050	Brazil
ERR2653051	Brazil
ERR2653052	Brazil
ERR2653053	Brazil
ERR2653054	Brazil
ERR2653055	Brazil
ERR2653056	Brazil
ERR2653057	Brazil

ERR2653058	Brazil
ERR2653059	Brazil
ERR2653060	Brazil
ERR2653061	Brazil
ERR2653062	Brazil
ERR2653063	Brazil
ERR2653064	Brazil
ERR2653065	Brazil
ERR2653066	Brazil
ERR2653067	Brazil
ERR2653068	Brazil
ERR2653069	Brazil
ERR2653070	Brazil
ERR2653071	Brazil
ERR2653072	Brazil
ERR2653073	Brazil
ERR2653074	Brazil
ERR2653075	Brazil
ERR2653076	Brazil
ERR2653077	Brazil
ERR2653078	Brazil
ERR2653079	Brazil
ERR2653080	Brazil
ERR2653081	Brazil
ERR2653082	Brazil
ERR2653083	Brazil
ERR2653084	Brazil
ERR2653085	Brazil
ERR2653086	Brazil
ERR2653087	Brazil
ERR2653088	Brazil
ERR2653089	Brazil
ERR2653090	Brazil
ERR2653091	Brazil
ERR2653092	Brazil
ERR2653093	Brazil
ERR2653094	Brazil
ERR2653095	Brazil
ERR2653096	Brazil
ERR2653097	Brazil
ERR2653098	Brazil
ERR2653099	Brazil
ERR2653100	Brazil
ERR2653101	Brazil
ERR2653102	Brazil
ERR2653103	Brazil
ERR2653104	Brazil
ERR2653105	Brazil
ERR2653106	Brazil
ERR2653107	Brazil

ERR2653108	Brazil
ERR2653109	Brazil
ERR2653110	Brazil
ERR2653111	Brazil
ERR2653112	Brazil
ERR2653113	Brazil
ERR2653114	Brazil
ERR2653115	Brazil
ERR2653116	Brazil
ERR2653117	Brazil
ERR2653118	Brazil
ERR2653119	Brazil
ERR2653120	Brazil
ERR2653121	Brazil
ERR2653122	Brazil
ERR2653123	Brazil
ERR2653124	Brazil
ERR2653125	Brazil
ERR2653126	Brazil
ERR2653127	Brazil
ERR2653128	Brazil
ERR2653129	Brazil
ERR2653130	Brazil
ERR2653131	Brazil
ERR2653132	Brazil
ERR2653133	Brazil
ERR2653134	Brazil
ERR2653135	Brazil
ERR2653136	Brazil
ERR2653137	Brazil
ERR2653138	Brazil
ERR2653139	Brazil
ERR2653140	Brazil
ERR2653141	Brazil
ERR2653142	Brazil
ERR2653143	Brazil
ERR2653144	Brazil
ERR2653145	Brazil
ERR2653146	Brazil
ERR2653147	Brazil
ERR2653148	Brazil
ERR2653149	Brazil
ERR2653150	Brazil
ERR2653151	Brazil
ERR2653152	Brazil
ERR2653153	Brazil
ERR2653154	Brazil
ERR2653155	Brazil
ERR2653156	Brazil
ERR2653157	Brazil

ERR2653158	Brazil
ERR2653159	Brazil
ERR2653160	Brazil
ERR2653161	Brazil
ERR2653162	Brazil
ERR2653163	Brazil
ERR2653164	Brazil
ERR2653165	Brazil
ERR2653166	Brazil
ERR2653167	Brazil
ERR2653168	Brazil
ERR2653169	Brazil
ERR2653170	Brazil
ERR2653171	Brazil
ERR2653172	Brazil
ERR2653173	Brazil
ERR2653174	Brazil
ERR2653175	Brazil
ERR2653176	Brazil
ERR2653177	Brazil
ERR2653178	Brazil
ERR2653179	Brazil
ERR2653180	Brazil
ERR2653181	Brazil
ERR2653182	Brazil
ERR2653183	Brazil
ERR2653184	Brazil
ERR2653185	Brazil
ERR2653186	Brazil
ERR2653187	Brazil
ERR2653188	Brazil
ERR2653189	Brazil
ERR2653190	Brazil
ERR2653191	Brazil
ERR2653192	Brazil
ERR2653193	Brazil
ERR2653194	Brazil
ERR2653195	Brazil
ERR2653196	Brazil
ERR2653197	Brazil
ERR2653198	Brazil
ERR2653199	Brazil
ERR2653200	Brazil
ERR2653201	Brazil
ERR2653202	Brazil
ERR2653203	Brazil
ERR2653204	Brazil
ERR2653205	Brazil
ERR2653206	Brazil
ERR2653207	Brazil

ERR2653208	Brazil
ERR2653209	Brazil
ERR2653210	Brazil
ERR2653211	Brazil
ERR2653212	Brazil
ERR2653213	Brazil
ERR2653214	Brazil
ERR2653215	Brazil
ERR2653216	Brazil
ERR2653217	Brazil
ERR2653218	Brazil
ERR2653219	Brazil
ERR2653220	Brazil
ERR2653221	Brazil
ERR2653222	Brazil
ERR2653223	Brazil
ERR2653224	Brazil
ERR2653225	Brazil
ERR2653226	Brazil
ERR2653227	Brazil
ERR2653228	Brazil
ERR2653229	Brazil
ERR2653230	Brazil
ERR2653231	Brazil
ERR2653232	Brazil
ERR2653233	Brazil
ERR2653234	Brazil
ERR2653235	Brazil
ERR2653236	Brazil
ERR2653237	Brazil
ERR2653238	Brazil
ERR2653239	Brazil
ERR2653240	Brazil
ERR2653241	Brazil
ERR266519	NA
ERR266520	NA
ERR266521	NA
ERR266522	NA
ERR266523	NA
ERR266524	NA
ERR266525	NA
ERR266526	NA
ERR266527	NA
ERR266528	NA
ERR266529	NA
ERR266530	NA
ERR266531	NA
ERR266532	NA
ERR266533	NA
ERR266534	NA

ERR266535	NA
ERR266536	NA
ERR266537	NA
ERR266538	NA
ERR266539	NA
ERR266540	NA
ERR266541	NA
ERR266542	NA
ERR266543	NA
ERR266544	NA
ERR266545	NA
ERR266546	NA
ERR266547	NA
ERR266548	NA
ERR266549	NA
ERR266550	NA
ERR266551	NA
ERR266552	NA
ERR266553	NA
ERR266554	NA
ERR266555	NA
ERR266556	NA
ERR266557	NA
ERR266558	NA
ERR266559	NA
ERR266560	NA
ERR266561	NA
ERR266562	NA
ERR266563	NA
ERR266564	NA
ERR266565	NA
ERR266566	NA
ERR266567	NA
ERR266568	NA
ERR266569	NA
ERR266570	NA
ERR266571	NA
ERR266572	NA
ERR266573	NA
ERR266574	NA
ERR266575	NA
ERR266576	NA
ERR266577	NA
ERR266578	NA
ERR266579	NA
ERR266580	NA
ERR266581	NA
ERR266582	NA
ERR266583	NA
ERR266584	NA

ERR266585	NA
ERR266586	NA
ERR266587	NA
ERR266588	NA
ERR266589	NA
ERR266590	NA
ERR266591	NA
ERR266592	NA
ERR266593	NA
ERR266594	NA
ERR266595	NA
ERR266596	NA
ERR266597	NA
ERR266598	NA
ERR266599	NA
ERR266600	NA
ERR266601	NA
ERR266602	NA
ERR266603	NA
ERR266604	NA
ERR266605	NA
ERR266606	NA
ERR266607	NA
ERR266608	NA
ERR266609	NA
ERR266610	NA
ERR266611	NA
ERR266612	NA
ERR266613	NA
ERR266614	NA
ERR2679242	NA
ERR2679243	NA
ERR2679244	NA
ERR2679245	NA
ERR2679247	NA
ERR2679248	NA
ERR2679249	NA
ERR2679251	NA
ERR2679252	NA
ERR2679253	NA
ERR2679254	NA
ERR2679255	NA
ERR2679256	NA
ERR2679257	NA
ERR2679259	NA
ERR2679260	NA
ERR2679261	NA
ERR2679262	NA
ERR2679263	NA
ERR2679264	NA

ERR2679265	NA
ERR2679266	NA
ERR2679267	NA
ERR2679268	NA
ERR2679269	NA
ERR2679270	NA
ERR2679271	NA
ERR2679272	NA
ERR2679273	NA
ERR2679274	NA
ERR2679275	NA
ERR2679276	NA
ERR2679277	NA
ERR2679279	NA
ERR2679280	NA
ERR2679281	NA
ERR2679283	NA
ERR2679284	NA
ERR2679285	NA
ERR2679286	NA
ERR2679287	NA
ERR2679288	NA
ERR2679289	NA
ERR2679290	NA
ERR2679291	NA
ERR2679292	NA
ERR2679293	NA
ERR2679294	NA
ERR2679295	NA
ERR2679296	NA
ERR2679297	NA
ERR2679298	NA
ERR2679299	NA
ERR2679300	NA
ERR2679301	NA
ERR2679302	NA
ERR2679303	NA
ERR2679304	NA
ERR2679305	NA
ERR2679306	NA
ERR2679307	NA
ERR2679308	NA
ERR2679309	NA
ERR2679310	NA
ERR2679311	NA
ERR2679312	NA
ERR2679313	NA
ERR2679314	NA
ERR2679315	NA
ERR270614	NA

ERR270615	NA
ERR270616	NA
ERR270617	NA
ERR270618	NA
ERR270619	NA
ERR270620	NA
ERR270621	NA
ERR270622	NA
ERR270623	NA
ERR270624	NA
ERR270625	NA
ERR270626	NA
ERR270627	NA
ERR270628	NA
ERR270629	NA
ERR270630	NA
ERR270631	NA
ERR270632	NA
ERR270633	NA
ERR270634	NA
ERR270635	NA
ERR270636	NA
ERR270637	NA
ERR270638	NA
ERR270639	NA
ERR270640	NA
ERR270641	NA
ERR270642	NA
ERR270643	NA
ERR270644	NA
ERR270645	NA
ERR270646	NA
ERR270647	NA
ERR270648	NA
ERR270649	NA
ERR270650	NA
ERR270651	NA
ERR270652	NA
ERR270653	NA
ERR270654	NA
ERR270655	NA
ERR270656	NA
ERR270657	NA
ERR270658	NA
ERR270659	NA
ERR270660	NA
ERR270661	NA
ERR270662	NA
ERR270663	NA
ERR270664	NA

ERR270665	NA
ERR270666	NA
ERR270667	NA
ERR270668	NA
ERR270669	NA
ERR270670	NA
ERR270671	NA
ERR270672	NA
ERR270673	NA
ERR270674	NA
ERR270675	NA
ERR270676	NA
ERR270677	NA
ERR270678	NA
ERR270679	NA
ERR270680	NA
ERR270681	NA
ERR270682	NA
ERR270683	NA
ERR270684	NA
ERR270685	NA
ERR270686	NA
ERR270687	NA
ERR270688	NA
ERR270689	NA
ERR270690	NA
ERR270691	NA
ERR270692	NA
ERR270693	NA
ERR270694	NA
ERR270695	NA
ERR270696	NA
ERR270697	NA
ERR270698	NA
ERR270699	NA
ERR270700	NA
ERR270701	NA
ERR270702	NA
ERR270703	NA
ERR270704	NA
ERR270705	NA
ERR270706	NA
ERR270707	NA
ERR270708	NA
ERR270709	NA
ERR270710	NA
ERR270711	NA
ERR270712	NA
ERR270713	NA
ERR270714	NA

ERR270715	NA
ERR270716	NA
ERR270717	NA
ERR270718	NA
ERR270719	NA
ERR270720	NA
ERR270721	NA
ERR270722	NA
ERR270723	NA
ERR270724	NA
ERR270725	NA
ERR270726	NA
ERR270727	NA
ERR270728	NA
ERR270729	NA
ERR270730	NA
ERR270731	NA
ERR270732	NA
ERR270733	NA
ERR270734	NA
ERR270735	NA
ERR270736	NA
ERR270737	NA
ERR270738	NA
ERR270739	NA
ERR270740	NA
ERR270741	NA
ERR270742	NA
ERR270743	NA
ERR270744	NA
ERR270745	NA
ERR270746	NA
ERR270747	NA
ERR270748	NA
ERR270749	NA
ERR270750	NA
ERR270751	NA
ERR270752	NA
ERR270753	NA
ERR270754	NA
ERR270755	NA
ERR270756	NA
ERR270757	NA
ERR270758	NA
ERR270759	NA
ERR270760	NA
ERR270761	NA
ERR270762	NA
ERR270763	NA
ERR270764	NA

ERR270765	NA
ERR270766	NA
ERR270767	NA
ERR270768	NA
ERR270769	NA
ERR270770	NA
ERR270771	NA
ERR270772	NA
ERR270773	NA
ERR270774	NA
ERR270775	NA
ERR270776	NA
ERR270777	NA
ERR270778	NA
ERR270779	NA
ERR270780	NA
ERR270781	NA
ERR270782	NA
ERR270783	NA
ERR270784	NA
ERR270785	NA
ERR270786	NA
ERR270787	NA
ERR270788	NA
ERR270789	NA
ERR270790	NA
ERR270791	NA
ERR270792	NA
ERR270793	NA
ERR270794	NA
ERR270795	NA
ERR270796	NA
ERR270797	NA
ERR270798	NA
ERR270799	NA
ERR270800	NA
ERR270801	NA
ERR270802	NA
ERR270803	NA
ERR270804	NA
ERR270805	NA
ERR275181	NA
ERR275182	NA
ERR275183	NA
ERR275184	NA
ERR275185	NA
ERR275186	NA
ERR275187	NA
ERR275188	NA
ERR275189	NA

ERR275190	NA
ERR275191	NA
ERR275192	NA
ERR275193	NA
ERR275194	NA
ERR275195	NA
ERR275196	NA
ERR275197	NA
ERR275198	NA
ERR275199	NA
ERR275200	NA
ERR275201	NA
ERR275202	NA
ERR275203	NA
ERR275204	NA
ERR275205	NA
ERR275206	NA
ERR275207	NA
ERR275208	NA
ERR275209	NA
ERR275210	NA
ERR275211	NA
ERR275212	NA
ERR275213	NA
ERR275214	NA
ERR275215	NA
ERR275216	NA
ERR275217	NA
ERR275218	NA
ERR275219	NA
ERR275220	NA
ERR275221	NA
ERR275222	NA
ERR275223	NA
ERR275224	NA
ERR275225	NA
ERR275226	NA
ERR275227	NA
ERR275228	NA
ERR275229	NA
ERR275230	NA
ERR275231	NA
ERR275232	NA
ERR275233	NA
ERR275234	NA
ERR275235	NA
ERR275236	NA
ERR278514	NA
ERR278515	NA
ERR278516	NA

ERR278517	NA
ERR278518	NA
ERR278519	NA
ERR278520	NA
ERR278521	NA
ERR278522	NA
ERR278523	NA
ERR278524	NA
ERR278525	NA
ERR278526	NA
ERR278527	NA
ERR278528	NA
ERR278529	NA
ERR278530	NA
ERR278531	NA
ERR278532	NA
ERR278533	NA
ERR278534	NA
ERR278535	NA
ERR278536	NA
ERR278537	NA
ERR278538	NA
ERR278539	NA
ERR278540	NA
ERR278541	NA
ERR278542	NA
ERR278543	NA
ERR278544	NA
ERR278545	NA
ERR278546	NA
ERR278547	NA
ERR278548	NA
ERR278549	NA
ERR278550	NA
ERR278551	NA
ERR278552	NA
ERR278553	NA
ERR278554	NA
ERR278555	NA
ERR278556	NA
ERR278557	NA
ERR278558	NA
ERR278559	NA
ERR278560	NA
ERR278561	NA
ERR278562	NA
ERR278563	NA
ERR278564	NA
ERR278565	NA
ERR278566	NA

ERR278567	NA
ERR278568	NA
ERR278569	NA
ERR278570	NA
ERR278571	NA
ERR278572	NA
ERR278573	NA
ERR278574	NA
ERR278575	NA
ERR278576	NA
ERR278577	NA
ERR278578	NA
ERR278579	NA
ERR278580	NA
ERR278581	NA
ERR278582	NA
ERR278583	NA
ERR278584	NA
ERR278585	NA
ERR278586	NA
ERR278587	NA
ERR278588	NA
ERR278589	NA
ERR278590	NA
ERR278591	NA
ERR278592	NA
ERR278593	NA
ERR278594	NA
ERR278595	NA
ERR278596	NA
ERR278597	NA
ERR278598	NA
ERR278599	NA
ERR278600	NA
ERR278601	NA
ERR278602	NA
ERR278603	NA
ERR278604	NA
ERR278605	NA
ERR278606	NA
ERR278607	NA
ERR278608	NA
ERR278609	NA
ERR279467	NA
ERR279468	NA
ERR279469	NA
ERR279470	NA
ERR279471	NA
ERR279472	NA
ERR279473	NA

ERR279474	NA
ERR279475	NA
ERR279476	NA
ERR279477	NA
ERR279478	NA
ERR279479	NA
ERR279480	NA
ERR279481	NA
ERR279482	NA
ERR279483	NA
ERR279484	NA
ERR279485	NA
ERR279486	NA
ERR279487	NA
ERR279488	NA
ERR279489	NA
ERR279490	NA
ERR279491	NA
ERR279492	NA
ERR279493	NA
ERR279494	NA
ERR279495	NA
ERR279496	NA
ERR279497	NA
ERR279498	NA
ERR279499	NA
ERR279500	NA
ERR279501	NA
ERR279502	NA
ERR279503	NA
ERR279504	NA
ERR279505	NA
ERR279506	NA
ERR279507	NA
ERR279508	NA
ERR279509	NA
ERR279510	NA
ERR279511	NA
ERR279512	NA
ERR279513	NA
ERR279514	NA
ERR279515	NA
ERR279516	NA
ERR279517	NA
ERR279518	NA
ERR279519	NA
ERR279520	NA
ERR279521	NA
ERR279522	NA
ERR279523	NA

ERR279524	NA
ERR279525	NA
ERR279526	NA
ERR279527	NA
ERR279528	NA
ERR279529	NA
ERR279530	NA
ERR279531	NA
ERR279532	NA
ERR279533	NA
ERR279534	NA
ERR279535	NA
ERR279536	NA
ERR279537	NA
ERR279538	NA
ERR279539	NA
ERR279540	NA
ERR279541	NA
ERR279542	NA
ERR279543	NA
ERR279544	NA
ERR279545	NA
ERR279546	NA
ERR279547	NA
ERR279548	NA
ERR279549	NA
ERR279550	NA
ERR279551	NA
ERR279552	NA
ERR279553	NA
ERR279554	NA
ERR279555	NA
ERR279556	NA
ERR279557	NA
ERR279558	NA
ERR279559	NA
ERR279560	NA
ERR279561	NA
ERR279562	NA
ERR279563	NA
ERR279564	NA
ERR279565	NA
ERR279566	NA
ERR279567	NA
ERR279568	NA
ERR279569	NA
ERR279570	NA
ERR279571	NA
ERR279572	NA
ERR279573	NA

ERR279574	NA
ERR279575	NA
ERR279576	NA
ERR279577	NA
ERR279578	NA
ERR279579	NA
ERR279580	NA
ERR279581	NA
ERR279582	NA
ERR279583	NA
ERR279584	NA
ERR279585	NA
ERR279586	NA
ERR279587	NA
ERR279588	NA
ERR279589	NA
ERR279590	NA
ERR279591	NA
ERR279592	NA
ERR279593	NA
ERR279594	NA
ERR279595	NA
ERR279596	NA
ERR279597	NA
ERR279598	NA
ERR279599	NA
ERR279600	NA
ERR279601	NA
ERR279602	NA
ERR279603	NA
ERR279604	NA
ERR279605	NA
ERR279606	NA
ERR279607	NA
ERR279608	NA
ERR279609	NA
ERR279610	NA
ERR279611	NA
ERR279612	NA
ERR279613	NA
ERR279614	NA
ERR279615	NA
ERR279616	NA
ERR279617	NA
ERR279618	NA
ERR279619	NA
ERR279620	NA
ERR279621	NA
ERR279622	NA
ERR279623	NA

ERR279624	NA
ERR279625	NA
ERR279626	NA
ERR279627	NA
ERR279628	NA
ERR279629	NA
ERR279630	NA
ERR279631	NA
ERR279632	NA
ERR279633	NA
ERR279634	NA
ERR279635	NA
ERR279636	NA
ERR279637	NA
ERR279638	NA
ERR279639	NA
ERR279640	NA
ERR279641	NA
ERR279642	NA
ERR279643	NA
ERR279644	NA
ERR279645	NA
ERR279646	NA
ERR279647	NA
ERR279648	NA
ERR279649	NA
ERR279650	NA
ERR279651	NA
ERR279652	NA
ERR279653	NA
ERR279654	NA
ERR279655	NA
ERR279656	NA
ERR279657	NA
ERR279658	NA
ERR294178	NA
ERR294179	NA
ERR294180	NA
ERR294181	NA
ERR294182	NA
ERR294183	NA
ERR294184	NA
ERR294185	NA
ERR294186	NA
ERR294187	NA
ERR294188	NA
ERR294189	NA
ERR294190	NA
ERR294191	NA
ERR294192	NA

ERR294193	NA
ERR294194	NA
ERR294195	NA
ERR294196	NA
ERR294197	NA
ERR294198	NA
ERR294199	NA
ERR294200	NA
ERR294201	NA
ERR294202	NA
ERR294203	NA
ERR294204	NA
ERR294205	NA
ERR294206	NA
ERR294207	NA
ERR294208	NA
ERR294209	NA
ERR294210	NA
ERR294211	NA
ERR294212	NA
ERR294213	NA
ERR294214	NA
ERR294215	NA
ERR294216	NA
ERR294217	NA
ERR294218	NA
ERR294219	NA
ERR294220	NA
ERR294221	NA
ERR294222	NA
ERR294223	NA
ERR294224	NA
ERR294225	NA
ERR294226	NA
ERR294227	NA
ERR294228	NA
ERR294229	NA
ERR294230	NA
ERR294231	NA
ERR294232	NA
ERR294233	NA
ERR294234	NA
ERR294235	NA
ERR294236	NA
ERR294237	NA
ERR294238	NA
ERR294239	NA
ERR294240	NA
ERR294241	NA
ERR294242	NA

ERR294243	NA
ERR294244	NA
ERR294245	NA
ERR294246	NA
ERR294247	NA
ERR294248	NA
ERR294249	NA
ERR294250	NA
ERR294251	NA
ERR294252	NA
ERR294253	NA
ERR294254	NA
ERR294255	NA
ERR294256	NA
ERR294257	NA
ERR294258	NA
ERR294259	NA
ERR294260	NA
ERR294261	NA
ERR294262	NA
ERR294263	NA
ERR294264	NA
ERR294265	NA
ERR294266	NA
ERR294267	NA
ERR294268	NA
ERR294269	NA
ERR294270	NA
ERR294271	NA
ERR294272	NA
ERR294273	NA
ERR323033	NA
ERR323034	NA
ERR323035	NA
ERR323036	NA
ERR323037	NA
ERR323038	NA
ERR323039	NA
ERR323040	NA
ERR323041	NA
ERR323042	NA
ERR323043	NA
ERR323044	NA
ERR323045	NA
ERR323046	NA
ERR323047	NA
ERR323048	NA
ERR323049	NA
ERR323050	NA
ERR323051	NA

ERR323052	NA
ERR323053	NA
ERR323054	NA
ERR323055	NA
ERR323056	NA
ERR323057	NA
ERR323058	NA
ERR323059	NA
ERR323060	NA
ERR323061	NA
ERR323062	NA
ERR323063	NA
ERR323064	NA
ERR323065	NA
ERR323066	NA
ERR323067	NA
ERR323068	NA
ERR323069	NA
ERR323070	NA
ERR323071	NA
ERR323072	NA
ERR323073	NA
ERR323074	NA
ERR323075	NA
ERR323076	NA
ERR323077	NA
ERR323078	NA
ERR323079	NA
ERR323080	NA
ERR323081	NA
ERR323082	NA
ERR323083	NA
ERR323084	NA
ERR323085	NA
ERR323086	NA
ERR323087	NA
ERR323088	NA
ERR323089	NA
ERR323090	NA
ERR323091	NA
ERR323092	NA
ERR323093	NA
ERR323094	NA
ERR323095	NA
ERR323096	NA
ERR323097	NA
ERR323098	NA
ERR323099	NA
ERR323100	NA
ERR323101	NA

ERR323102	NA
ERR323103	NA
ERR323104	NA
ERR323105	NA
ERR323106	NA
ERR323107	NA
ERR323108	NA
ERR323109	NA
ERR323110	NA
ERR323111	NA
ERR323112	NA
ERR323113	NA
ERR323114	NA
ERR323115	NA
ERR323116	NA
ERR323117	NA
ERR323118	NA
ERR323119	NA
ERR330650	NA
ERR330651	NA
ERR330652	NA
ERR330653	NA
ERR330654	NA
ERR330655	NA
ERR330656	NA
ERR330657	NA
ERR330658	NA
ERR330659	NA
ERR330660	NA
ERR330661	NA
ERR330662	NA
ERR330663	NA
ERR330664	NA
ERR330665	NA
ERR330666	NA
ERR330667	NA
ERR330668	NA
ERR330669	NA
ERR330670	NA
ERR330671	NA
ERR330672	NA
ERR330673	NA
ERR330674	NA
ERR330675	NA
ERR330676	NA
ERR330677	NA
ERR330678	NA
ERR330679	NA
ERR330680	NA
ERR330681	NA

ERR330682	NA
ERR330683	NA
ERR330684	NA
ERR330685	NA
ERR330686	NA
ERR330687	NA
ERR330688	NA
ERR330689	NA
ERR330690	NA
ERR330691	NA
ERR330692	NA
ERR330693	NA
ERR330694	NA
ERR330695	NA
ERR330696	NA
ERR330697	NA
ERR330698	NA
ERR330699	NA
ERR330700	NA
ERR330701	NA
ERR330702	NA
ERR330703	NA
ERR330704	NA
ERR330705	NA
ERR330706	NA
ERR330707	NA
ERR330708	NA
ERR330709	NA
ERR330710	NA
ERR330711	NA
ERR330712	NA
ERR330713	NA
ERR330714	NA
ERR330715	NA
ERR330716	NA
ERR330717	NA
ERR330718	NA
ERR330719	NA
ERR330720	NA
ERR330721	NA
ERR330722	NA
ERR330723	NA
ERR330724	NA
ERR330725	NA
ERR330726	NA
ERR330727	NA
ERR330728	NA
ERR330729	NA
ERR330730	NA
ERR330731	NA

ERR330732	NA
ERR330733	NA
ERR330734	NA
ERR330735	NA
ERR330736	NA
ERR330737	NA
ERR330738	NA
ERR330739	NA
ERR330740	NA
ERR330741	NA
ERR330742	NA
ERR330743	NA
ERR330744	NA
ERR330745	NA
ERR349264	NA
ERR349265	NA
ERR349266	NA
ERR349296	NA
ERR349298	NA
ERR349299	NA
ERR349300	NA
ERR349330	NA
ERR351845	NA
ERR351846	NA
ERR351847	NA
ERR351848	NA
ERR351849	NA
ERR351850	NA
ERR351851	NA
ERR351852	NA
ERR351853	NA
ERR351854	NA
ERR351855	NA
ERR351856	NA
ERR351857	NA
ERR351858	NA
ERR351859	NA
ERR351860	NA
ERR351861	NA
ERR351862	NA
ERR351863	NA
ERR351864	NA
ERR351865	NA
ERR351866	NA
ERR351867	NA
ERR351868	NA
ERR351869	NA
ERR351870	NA
ERR351871	NA
ERR351872	NA

ERR351873	NA
ERR351874	NA
ERR351875	NA
ERR351876	NA
ERR351877	NA
ERR351878	NA
ERR351879	NA
ERR351880	NA
ERR351881	NA
ERR351882	NA
ERR351883	NA
ERR351884	NA
ERR351885	NA
ERR351886	NA
ERR351887	NA
ERR351888	NA
ERR351889	NA
ERR351890	NA
ERR351891	NA
ERR351892	NA
ERR351893	NA
ERR351894	NA
ERR351895	NA
ERR351896	NA
ERR351897	NA
ERR351898	NA
ERR351899	NA
ERR351900	NA
ERR351901	NA
ERR351902	NA
ERR351903	NA
ERR351904	NA
ERR351905	NA
ERR351906	NA
ERR351907	NA
ERR351908	NA
ERR351909	NA
ERR351910	NA
ERR351911	NA
ERR351912	NA
ERR351913	NA
ERR351914	NA
ERR351915	NA
ERR351916	NA
ERR351917	NA
ERR351918	NA
ERR351919	NA
ERR351920	NA
ERR351921	NA
ERR351922	NA

ERR351923	NA
ERR351924	NA
ERR351925	NA
ERR351926	NA
ERR351927	NA
ERR351928	NA
ERR351929	NA
ERR351930	NA
ERR351931	NA
ERR351932	NA
ERR351933	NA
ERR351934	NA
ERR351935	NA
ERR351936	NA
ERR351937	NA
ERR351938	NA
ERR351939	NA
ERR351940	NA
ERR356389	NA
ERR356390	NA
ERR363421	NA
ERR363426	NA
ERR363437	NA
ERR363445	NA
ERR363446	NA
ERR363447	NA
ERR363449	NA
ERR363453	NA
ERR363456	NA
ERR363474	NA
ERR363479	NA
ERR363490	NA
ERR363498	NA
ERR363499	NA
ERR363500	NA
ERR363502	NA
ERR363506	NA
ERR363509	NA
ERR369151	NA
ERR369156	NA
ERR369157	NA
ERR369158	NA
ERR369159	NA
ERR369160	NA
ERR369161	NA
ERR369162	NA
ERR369163	NA
ERR369586	NA
ERR369587	NA
ERR369588	NA

ERR369589	NA
ERR369590	NA
ERR369591	NA
ERR369592	NA
ERR369593	NA
ERR369594	NA
ERR369595	NA
ERR369596	NA
ERR369597	NA
ERR369598	NA
ERR369599	NA
ERR369600	NA
ERR369601	NA
ERR369602	NA
ERR369603	NA
ERR369604	NA
ERR369605	NA
ERR369606	NA
ERR369607	NA
ERR369608	NA
ERR369609	NA
ERR369610	NA
ERR369611	NA
ERR369612	NA
ERR369613	NA
ERR369614	NA
ERR369615	NA
ERR369616	NA
ERR369617	NA
ERR369618	NA
ERR369619	NA
ERR369620	NA
ERR369621	NA
ERR369622	NA
ERR369623	NA
ERR369624	NA
ERR369625	NA
ERR369626	NA
ERR369627	NA
ERR369628	NA
ERR369629	NA
ERR369630	NA
ERR369631	NA
ERR369632	NA
ERR369633	NA
ERR369634	NA
ERR369635	NA
ERR369636	NA
ERR369637	NA
ERR369638	NA

ERR369639	NA
ERR369640	NA
ERR369641	NA
ERR369642	NA
ERR369643	NA
ERR369644	NA
ERR369645	NA
ERR369646	NA
ERR369647	NA
ERR369648	NA
ERR369649	NA
ERR369650	NA
ERR369651	NA
ERR369652	NA
ERR369653	NA
ERR369654	NA
ERR369655	NA
ERR369656	NA
ERR369657	NA
ERR369658	NA
ERR369659	NA
ERR369660	NA
ERR369661	NA
ERR369662	NA
ERR369663	NA
ERR369664	NA
ERR369665	NA
ERR369666	NA
ERR369667	NA
ERR369668	NA
ERR369669	NA
ERR369670	NA
ERR369671	NA
ERR369672	NA
ERR369673	NA
ERR369674	NA
ERR369675	NA
ERR369676	NA
ERR369677	NA
ERR369678	NA
ERR369679	NA
ERR369680	NA
ERR369681	NA
ERR369682	NA
ERR369683	NA
ERR369684	NA
ERR369685	NA
ERR369686	NA
ERR369687	NA
ERR369688	NA

ERR369689	NA
ERR369690	NA
ERR369691	NA
ERR369692	NA
ERR369693	NA
ERR369694	NA
ERR369695	NA
ERR369696	NA
ERR369697	NA
ERR369698	NA
ERR369699	NA
ERR369700	NA
ERR369701	NA
ERR369702	NA
ERR369703	NA
ERR369704	NA
ERR369705	NA
ERR369706	NA
ERR369707	NA
ERR369708	NA
ERR369709	NA
ERR369710	NA
ERR369711	NA
ERR369712	NA
ERR369713	NA
ERR369714	NA
ERR369715	NA
ERR369716	NA
ERR369717	NA
ERR369718	NA
ERR369719	NA
ERR369720	NA
ERR369721	NA
ERR369722	NA
ERR369723	NA
ERR369724	NA
ERR369725	NA
ERR369726	NA
ERR369727	NA
ERR369728	NA
ERR369729	NA
ERR369730	NA
ERR369731	NA
ERR369732	NA
ERR369733	NA
ERR369734	NA
ERR369735	NA
ERR369736	NA
ERR369737	NA
ERR369738	NA

ERR369739	NA
ERR369740	NA
ERR369741	NA
ERR369742	NA
ERR369743	NA
ERR369744	NA
ERR369745	NA
ERR369746	NA
ERR369747	NA
ERR369748	NA
ERR369749	NA
ERR369750	NA
ERR369751	NA
ERR369752	NA
ERR369753	NA
ERR369754	NA
ERR369755	NA
ERR369756	NA
ERR369757	NA
ERR369758	NA
ERR369759	NA
ERR373945	NA
ERR373946	NA
ERR373947	NA
ERR376096	NA
ERR376097	NA
ERR386777	NA
ERR386778	NA
ERR386779	NA
ERR386780	NA
ERR386781	NA
ERR386782	NA
ERR386783	NA
ERR386784	NA
ERR386785	NA
ERR386786	NA
ERR386787	NA
ERR386788	NA
ERR386789	NA
ERR386790	NA
ERR386791	NA
ERR386792	NA
ERR386793	NA
ERR386794	NA
ERR386795	NA
ERR386796	NA
ERR386797	NA
ERR386798	NA
ERR386799	NA
ERR386800	NA

ERR386801	NA
ERR386802	NA
ERR386803	NA
ERR386804	NA
ERR386805	NA
ERR386806	NA
ERR386807	NA
ERR386808	NA
ERR386809	NA
ERR386810	NA
ERR386811	NA
ERR386812	NA
ERR386813	NA
ERR386814	NA
ERR386815	NA
ERR386816	NA
ERR386817	NA
ERR386818	NA
ERR386819	NA
ERR386820	NA
ERR386821	NA
ERR386822	NA
ERR386823	NA
ERR386824	NA
ERR386825	NA
ERR386826	NA
ERR386827	NA
ERR386828	NA
ERR386829	NA
ERR386830	NA
ERR386831	NA
ERR386832	NA
ERR386833	NA
ERR386834	NA
ERR386835	NA
ERR386836	NA
ERR386837	NA
ERR386838	NA
ERR386839	NA
ERR386840	NA
ERR386841	NA
ERR386842	NA
ERR386843	NA
ERR386844	NA
ERR386845	NA
ERR386846	NA
ERR386847	NA
ERR386848	NA
ERR386849	NA
ERR386850	NA

ERR386851	NA
ERR386852	NA
ERR386853	NA
ERR386854	NA
ERR386855	NA
ERR386856	NA
ERR386857	NA
ERR386858	NA
ERR386859	NA
ERR386860	NA
ERR386861	NA
ERR386862	NA
ERR386863	NA
ERR386864	NA
ERR386865	NA
ERR386866	NA
ERR386867	NA
ERR386868	NA
ERR386869	NA
ERR386870	NA
ERR386871	NA
ERR386872	NA
ERR386873	NA
ERR386874	NA
ERR386875	NA
ERR386876	NA
ERR386877	NA
ERR386878	NA
ERR386879	NA
ERR386880	NA
ERR386881	NA
ERR386882	NA
ERR386883	NA
ERR386884	NA
ERR386885	NA
ERR386886	NA
ERR386887	NA
ERR386888	NA
ERR386889	NA
ERR386890	NA
ERR386891	NA
ERR386892	NA
ERR386893	NA
ERR386894	NA
ERR386895	NA
ERR386896	NA
ERR386897	NA
ERR386898	NA
ERR386899	NA
ERR386900	NA

ERR386901	NA
ERR386902	NA
ERR386903	NA
ERR386904	NA
ERR386905	NA
ERR386906	NA
ERR386907	NA
ERR386908	NA
ERR386909	NA
ERR386910	NA
ERR386911	NA
ERR386912	NA
ERR386913	NA
ERR386914	NA
ERR386915	NA
ERR386916	NA
ERR386917	NA
ERR386918	NA
ERR386919	NA
ERR386920	NA
ERR386921	NA
ERR386922	NA
ERR386923	NA
ERR386924	NA
ERR386925	NA
ERR386926	NA
ERR386927	NA
ERR386928	NA
ERR386929	NA
ERR386930	NA
ERR386931	NA
ERR386932	NA
ERR386933	NA
ERR386934	NA
ERR386935	NA
ERR386936	NA
ERR386937	NA
ERR386938	NA
ERR386939	NA
ERR386940	NA
ERR386941	NA
ERR386942	NA
ERR386943	NA
ERR386944	NA
ERR386945	NA
ERR386946	NA
ERR386947	NA
ERR386948	NA
ERR386949	NA
ERR386950	NA

ERR386951	NA
ERR386952	NA
ERR386953	NA
ERR386954	NA
ERR386955	NA
ERR386956	NA
ERR386957	NA
ERR386958	NA
ERR386959	NA
ERR386960	NA
ERR386961	NA
ERR386962	NA
ERR386963	NA
ERR386964	NA
ERR386965	NA
ERR386966	NA
ERR386967	NA
ERR386968	NA
ERR386969	NA
ERR386970	NA
ERR386971	NA
ERR386972	NA
ERR386973	NA
ERR386974	NA
ERR386975	NA
ERR386976	NA
ERR386977	NA
ERR386978	NA
ERR386979	NA
ERR386980	NA
ERR386981	NA
ERR386982	NA
ERR386983	NA
ERR386984	NA
ERR386985	NA
ERR386986	NA
ERR386987	NA
ERR386988	NA
ERR386989	NA
ERR386990	NA
ERR386991	NA
ERR386992	NA
ERR386993	NA
ERR386994	NA
ERR386995	NA
ERR386996	NA
ERR386997	NA
ERR386998	NA
ERR386999	NA
ERR387000	NA

ERR387001	NA
ERR387002	NA
ERR387003	NA
ERR387004	NA
ERR387005	NA
ERR387006	NA
ERR387007	NA
ERR387008	NA
ERR387009	NA
ERR387010	NA
ERR387011	NA
ERR387012	NA
ERR387013	NA
ERR387014	NA
ERR387015	NA
ERR387016	NA
ERR387017	NA
ERR387018	NA
ERR387019	NA
ERR387020	NA
ERR387021	NA
ERR387022	NA
ERR387023	NA
ERR387024	NA
ERR387025	NA
ERR387026	NA
ERR387027	NA
ERR387028	NA
ERR387029	NA
ERR387030	NA
ERR387031	NA
ERR387032	NA
ERR387033	NA
ERR387034	NA
ERR387035	NA
ERR387036	NA
ERR387037	NA
ERR387038	NA
ERR387039	NA
ERR387040	NA
ERR387041	NA
ERR387042	NA
ERR387043	NA
ERR387044	NA
ERR387045	NA
ERR387046	NA
ERR387047	NA
ERR387048	NA
ERR387049	NA
ERR387050	NA

ERR387051	NA
ERR387052	NA
ERR387053	NA
ERR387054	NA
ERR387055	NA
ERR387056	NA
ERR387057	NA
ERR387058	NA
ERR387059	NA
ERR387060	NA
ERR387061	NA
ERR387062	NA
ERR387063	NA
ERR392507	NA
ERR392508	NA
ERR392509	NA
ERR392510	NA
ERR392511	NA
ERR400306	NA
ERR400307	NA
ERR400308	NA
ERR400309	NA
ERR400310	NA
ERR400311	NA
ERR400312	NA
ERR400313	NA
ERR400314	NA
ERR400315	NA
ERR400316	NA
ERR400317	NA
ERR400318	NA
ERR400319	NA
ERR400320	NA
ERR400321	NA
ERR400322	NA
ERR400323	NA
ERR400324	NA
ERR400325	NA
ERR400326	NA
ERR400327	NA
ERR400328	NA
ERR400329	NA
ERR400330	NA
ERR400331	NA
ERR400332	NA
ERR400333	NA
ERR400334	NA
ERR400335	NA
ERR400336	NA
ERR400337	NA

ERR400338	NA
ERR400339	NA
ERR400340	NA
ERR400341	NA
ERR400342	NA
ERR400343	NA
ERR400344	NA
ERR400345	NA
ERR400346	NA
ERR400347	NA
ERR400348	NA
ERR400349	NA
ERR400350	NA
ERR400351	NA
ERR400352	NA
ERR400353	NA
ERR400354	NA
ERR400355	NA
ERR400356	NA
ERR400357	NA
ERR400358	NA
ERR400359	NA
ERR400360	NA
ERR400361	NA
ERR400362	NA
ERR400363	NA
ERR400364	NA
ERR400365	NA
ERR400366	NA
ERR400367	NA
ERR400368	NA
ERR400369	NA
ERR400370	NA
ERR400371	NA
ERR400372	NA
ERR400373	NA
ERR400374	NA
ERR400375	NA
ERR400376	NA
ERR400377	NA
ERR400378	NA
ERR400379	NA
ERR400380	NA
ERR400381	NA
ERR400382	NA
ERR400383	NA
ERR400384	NA
ERR400385	NA
ERR400386	NA
ERR400387	NA

ERR400388	NA
ERR400389	NA
ERR400390	NA
ERR400391	NA
ERR400392	NA
ERR400393	NA
ERR400394	NA
ERR400395	NA
ERR400396	NA
ERR400397	NA
ERR400398	NA
ERR400399	NA
ERR400400	NA
ERR400401	NA
ERR400402	NA
ERR400403	NA
ERR400404	NA
ERR400405	NA
ERR400406	NA
ERR400407	NA
ERR400408	NA
ERR400409	NA
ERR400410	NA
ERR400411	NA
ERR400412	NA
ERR400413	NA
ERR400414	NA
ERR400415	NA
ERR400416	NA
ERR400417	NA
ERR400418	NA
ERR400419	NA
ERR400420	NA
ERR400421	NA
ERR400422	NA
ERR400423	NA
ERR400424	NA
ERR400425	NA
ERR400426	NA
ERR400427	NA
ERR400428	NA
ERR400429	NA
ERR400430	NA
ERR400431	NA
ERR400432	NA
ERR400433	NA
ERR400434	NA
ERR400435	NA
ERR400436	NA
ERR400437	NA

ERR400438	NA
ERR400439	NA
ERR400440	NA
ERR400441	NA
ERR400442	NA
ERR400443	NA
ERR400444	NA
ERR400445	NA
ERR400446	NA
ERR400447	NA
ERR400448	NA
ERR400449	NA
ERR400450	NA
ERR400451	NA
ERR400452	NA
ERR400453	NA
ERR400454	NA
ERR400455	NA
ERR400456	NA
ERR400457	NA
ERR400458	NA
ERR400459	NA
ERR400460	NA
ERR400461	NA
ERR400462	NA
ERR400463	NA
ERR400464	NA
ERR400465	NA
ERR400466	NA
ERR400467	NA
ERR400468	NA
ERR400469	NA
ERR400470	NA
ERR400471	NA
ERR400472	NA
ERR400473	NA
ERR400474	NA
ERR400475	NA
ERR400476	NA
ERR400477	NA
ERR400478	NA
ERR400479	NA
ERR400480	NA
ERR400481	NA
ERR400482	NA
ERR400483	NA
ERR400484	NA
ERR400485	NA
ERR400486	NA
ERR400487	NA

ERR400488	NA
ERR400489	NA
ERR400490	NA
ERR400491	NA
ERR400492	NA
ERR400493	NA
ERR400494	NA
ERR400495	NA
ERR400496	NA
ERR400497	NA
ERR400498	NA
ERR400499	NA
ERR400500	NA
ERR400501	NA
ERR400502	NA
ERR400503	NA
ERR400504	NA
ERR400505	NA
ERR400506	NA
ERR400507	NA
ERR400508	NA
ERR400509	NA
ERR400510	NA
ERR400511	NA
ERR400512	NA
ERR400513	NA
ERR400514	NA
ERR400515	NA
ERR400516	NA
ERR400517	NA
ERR400518	NA
ERR400519	NA
ERR400520	NA
ERR400521	NA
ERR400522	NA
ERR400523	NA
ERR400524	NA
ERR400525	NA
ERR400526	NA
ERR400527	NA
ERR400528	NA
ERR400529	NA
ERR400530	NA
ERR400531	NA
ERR400532	NA
ERR400533	NA
ERR400534	NA
ERR400535	NA
ERR400536	NA
ERR400537	NA

ERR400538	NA
ERR400539	NA
ERR400540	NA
ERR400541	NA
ERR400542	NA
ERR400543	NA
ERR400544	NA
ERR400545	NA
ERR400546	NA
ERR400547	NA
ERR400548	NA
ERR400549	NA
ERR400550	NA
ERR400551	NA
ERR400552	NA
ERR400553	NA
ERR400554	NA
ERR400555	NA
ERR400556	NA
ERR400557	NA
ERR400558	NA
ERR403214	NA
ERR403215	NA
ERR403216	NA
ERR403217	NA
ERR403218	NA
ERR403219	NA
ERR403220	NA
ERR403221	NA
ERR403222	NA
ERR403223	NA
ERR403224	NA
ERR403225	NA
ERR403226	NA
ERR403227	NA
ERR403228	NA
ERR403229	NA
ERR403230	NA
ERR403231	NA
ERR403232	NA
ERR403233	NA
ERR403234	NA
ERR403235	NA
ERR403236	NA
ERR403237	NA
ERR403238	NA
ERR403239	NA
ERR403240	NA
ERR403241	NA
ERR403242	NA

ERR403243	NA
ERR403244	NA
ERR403245	NA
ERR403246	NA
ERR403247	NA
ERR403248	NA
ERR403249	NA
ERR403250	NA
ERR403251	NA
ERR403252	NA
ERR403253	NA
ERR403254	NA
ERR403255	NA
ERR403256	NA
ERR403257	NA
ERR403258	NA
ERR403259	NA
ERR403260	NA
ERR403261	NA
ERR403262	NA
ERR403263	NA
ERR403264	NA
ERR403265	NA
ERR403266	NA
ERR403267	NA
ERR403268	NA
ERR403269	NA
ERR403270	NA
ERR403271	NA
ERR403272	NA
ERR403273	NA
ERR403274	NA
ERR403275	NA
ERR403276	NA
ERR403277	NA
ERR403278	NA
ERR403279	NA
ERR403280	NA
ERR403281	NA
ERR403282	NA
ERR403283	NA
ERR403284	NA
ERR403285	NA
ERR403286	NA
ERR403287	NA
ERR403288	NA
ERR403289	NA
ERR403290	NA
ERR403291	NA
ERR403292	NA

ERR403293	NA
ERR403294	NA
ERR403295	NA
ERR403296	NA
ERR403297	NA
ERR403298	NA
ERR403299	NA
ERR403300	NA
ERR403301	NA
ERR403302	NA
ERR403303	NA
ERR403304	NA
ERR403305	NA
ERR403306	NA
ERR403307	NA
ERR403308	NA
ERR403309	NA
ERR403310	NA
ERR403311	NA
ERR403312	NA
ERR403313	NA
ERR403314	NA
ERR403315	NA
ERR403316	NA
ERR403317	NA
ERR403318	NA
ERR403319	NA
ERR403320	NA
ERR403321	NA
ERR403322	NA
ERR403323	NA
ERR403324	NA
ERR403325	NA
ERR403326	NA
ERR403327	NA
ERR403328	NA
ERR403329	NA
ERR403330	NA
ERR403331	NA
ERR403332	NA
ERR403333	NA
ERR403334	NA
ERR403335	NA
ERR403336	NA
ERR403337	NA
ERR403338	NA
ERR403339	NA
ERR403340	NA
ERR403341	NA
ERR403342	NA

ERR403343	NA
ERR403344	NA
ERR403345	NA
ERR403346	NA
ERR403347	NA
ERR403348	NA
ERR403349	NA
ERR403350	NA
ERR403351	NA
ERR403352	NA
ERR403353	NA
ERR403354	NA
ERR403355	NA
ERR403356	NA
ERR403357	NA
ERR403358	NA
ERR403359	NA
ERR403360	NA
ERR403361	NA
ERR403362	NA
ERR403363	NA
ERR403364	NA
ERR403365	NA
ERR403366	NA
ERR403367	NA
ERR403368	NA
ERR403369	NA
ERR403370	NA
ERR403371	NA
ERR403372	NA
ERR403373	NA
ERR403374	NA
ERR403375	NA
ERR403376	NA
ERR403377	NA
ERR403378	NA
ERR403379	NA
ERR403380	NA
ERR403381	NA
ERR403382	NA
ERR403383	NA
ERR403384	NA
ERR403385	NA
ERR403386	NA
ERR403387	NA
ERR403388	NA
ERR403389	NA
ERR403390	NA
ERR403391	NA
ERR403392	NA

ERR403393	NA
ERR403394	NA
ERR403395	NA
ERR403396	NA
ERR403397	NA
ERR403398	NA
ERR403399	NA
ERR403400	NA
ERR403401	NA
ERR403402	NA
ERR403403	NA
ERR403404	NA
ERR403405	NA
ERR403406	NA
ERR403407	NA
ERR403408	NA
ERR403409	NA
ERR403410	NA
ERR403411	NA
ERR403412	NA
ERR403413	NA
ERR403414	NA
ERR403415	NA
ERR403416	NA
ERR403417	NA
ERR403418	NA
ERR403419	NA
ERR403420	NA
ERR403421	NA
ERR405186	NA
ERR405187	NA
ERR405188	NA
ERR405189	NA
ERR405190	NA
ERR405191	NA
ERR405192	NA
ERR405193	NA
ERR405194	NA
ERR405195	NA
ERR405196	NA
ERR405197	NA
ERR405198	NA
ERR405199	NA
ERR405200	NA
ERR405201	NA
ERR405202	NA
ERR405203	NA
ERR405204	NA
ERR405205	NA
ERR405206	NA

ERR405207	NA
ERR405208	NA
ERR405209	NA
ERR405210	NA
ERR405211	NA
ERR405212	NA
ERR405213	NA
ERR405214	NA
ERR405215	NA
ERR405216	NA
ERR405217	NA
ERR405218	NA
ERR405219	NA
ERR405220	NA
ERR405221	NA
ERR405222	NA
ERR405223	NA
ERR433208	NA
ERR440680	NA
ERR459440	Norway
ERR459441	Norway
ERR459442	Norway
ERR459443	Norway
ERR459444	Norway
ERR459445	Norway
ERR459446	Norway
ERR459447	Norway
ERR459448	Norway
ERR473277	NA
ERR473278	NA
ERR473279	NA
ERR473280	NA
ERR473281	NA
ERR473282	NA
ERR473283	NA
ERR473284	NA
ERR473285	NA
ERR473286	NA
ERR473287	NA
ERR473288	NA
ERR473289	NA
ERR473290	NA
ERR473291	NA
ERR473292	NA
ERR473293	NA
ERR473294	NA
ERR473295	NA
ERR473296	NA
ERR473297	NA
ERR473298	NA

ERR473299	NA
ERR473300	NA
ERR473301	NA
ERR473302	NA
ERR473303	NA
ERR473304	NA
ERR473305	NA
ERR473306	NA
ERR473307	NA
ERR473308	NA
ERR473309	NA
ERR473310	NA
ERR473311	NA
ERR473312	NA
ERR473313	NA
ERR473314	NA
ERR473315	NA
ERR473316	NA
ERR473317	NA
ERR473318	NA
ERR473319	NA
ERR473320	NA
ERR473321	NA
ERR473322	NA
ERR473323	NA
ERR473324	NA
ERR473325	NA
ERR473326	NA
ERR473327	NA
ERR473328	NA
ERR473329	NA
ERR473330	NA
ERR473331	NA
ERR473332	NA
ERR473333	NA
ERR473334	NA
ERR473335	NA
ERR473336	NA
ERR473337	NA
ERR473338	NA
ERR473339	NA
ERR473340	NA
ERR473341	NA
ERR473342	NA
ERR473343	NA
ERR473344	NA
ERR473345	NA
ERR473346	NA
ERR473347	NA
ERR473348	NA

ERR473349	NA
ERR473350	NA
ERR473351	NA
ERR473352	NA
ERR473353	NA
ERR473354	NA
ERR473355	NA
ERR473356	NA
ERR473357	NA
ERR473358	NA
ERR473359	NA
ERR473360	NA
ERR473361	NA
ERR473362	NA
ERR473363	NA
ERR473364	NA
ERR473365	NA
ERR473366	NA
ERR473367	NA
ERR473368	NA
ERR473369	NA
ERR484733	NA
ERR484734	NA
ERR484739	NA
ERR484740	NA
ERR484741	NA
ERR484742	NA
ERR484743	NA
ERR484744	NA
ERR484745	NA
ERR484746	NA
ERR484747	NA
ERR484748	NA
ERR485006	NA
ERR485007	NA
ERR494998	NA
ERR494999	NA
ERR495000	NA
ERR495001	NA
ERR495002	NA
ERR495003	NA
ERR495004	NA
ERR495005	NA
ERR495006	NA
ERR495007	NA
ERR495008	NA
ERR495009	NA
ERR495010	NA
ERR495011	NA
ERR495012	NA

ERR495013	NA
ERR495014	NA
ERR495015	NA
ERR495016	NA
ERR495017	NA
ERR495018	NA
ERR495019	NA
ERR495020	NA
ERR495021	NA
ERR495022	NA
ERR495023	NA
ERR495024	NA
ERR495025	NA
ERR495026	NA
ERR495027	NA
ERR495028	NA
ERR495029	NA
ERR495030	NA
ERR495031	NA
ERR495032	NA
ERR495033	NA
ERR495034	NA
ERR495035	NA
ERR495036	NA
ERR495037	NA
ERR495038	NA
ERR495039	NA
ERR495040	NA
ERR495041	NA
ERR495042	NA
ERR495043	NA
ERR495044	NA
ERR495045	NA
ERR495046	NA
ERR495047	NA
ERR495048	NA
ERR495049	NA
ERR495050	NA
ERR495051	NA
ERR495052	NA
ERR495053	NA
ERR495054	NA
ERR495055	NA
ERR495056	NA
ERR495057	NA
ERR495058	NA
ERR495059	NA
ERR495060	NA
ERR495061	NA
ERR495062	NA

ERR495063	NA
ERR495064	NA
ERR495065	NA
ERR495066	NA
ERR495067	NA
ERR495068	NA
ERR495069	NA
ERR495070	NA
ERR495071	NA
ERR495072	NA
ERR495073	NA
ERR495074	NA
ERR495075	NA
ERR495076	NA
ERR495077	NA
ERR495078	NA
ERR495079	NA
ERR495080	NA
ERR495081	NA
ERR495082	NA
ERR495083	NA
ERR495084	NA
ERR495085	NA
ERR495086	NA
ERR495087	NA
ERR495088	NA
ERR495089	NA
ERR495090	NA
ERR495091	NA
ERR495092	NA
ERR495093	NA
ERR495094	NA
ERR495095	NA
ERR495096	NA
ERR495097	NA
ERR495098	NA
ERR495099	NA
ERR495100	NA
ERR495101	NA
ERR495102	NA
ERR495103	NA
ERR495104	NA
ERR495105	NA
ERR495106	NA
ERR495107	NA
ERR495108	NA
ERR495109	NA
ERR495110	NA
ERR495111	NA
ERR495112	NA

ERR495113	NA
ERR495114	NA
ERR495115	NA
ERR495116	NA
ERR495117	NA
ERR495118	NA
ERR495119	NA
ERR495120	NA
ERR495121	NA
ERR495122	NA
ERR495123	NA
ERR495124	NA
ERR495125	NA
ERR495126	NA
ERR495127	NA
ERR495128	NA
ERR495129	NA
ERR495130	NA
ERR495131	NA
ERR495132	NA
ERR495133	NA
ERR495134	NA
ERR495135	NA
ERR495136	NA
ERR495137	NA
ERR495138	NA
ERR495139	NA
ERR495140	NA
ERR495141	NA
ERR495142	NA
ERR495143	NA
ERR495144	NA
ERR495145	NA
ERR495146	NA
ERR495147	NA
ERR495148	NA
ERR495149	NA
ERR495150	NA
ERR495151	NA
ERR495152	NA
ERR495153	NA
ERR495154	NA
ERR495155	NA
ERR495156	NA
ERR495157	NA
ERR495158	NA
ERR495159	NA
ERR495160	NA
ERR495161	NA
ERR495162	NA

ERR495163	NA
ERR495164	NA
ERR495165	NA
ERR495166	NA
ERR495167	NA
ERR495168	NA
ERR495169	NA
ERR495170	NA
ERR495171	NA
ERR495172	NA
ERR495173	NA
ERR495174	NA
ERR495175	NA
ERR495176	NA
ERR495177	NA
ERR495178	NA
ERR495179	NA
ERR495180	NA
ERR495181	NA
ERR495182	NA
ERR495183	NA
ERR495184	NA
ERR495185	NA
ERR495186	NA
ERR495187	NA
ERR495188	NA
ERR495189	NA
ERR495190	NA
ERR495191	NA
ERR495192	NA
ERR495193	NA
ERR495194	NA
ERR495195	NA
ERR495196	NA
ERR495197	NA
ERR495198	NA
ERR495199	NA
ERR495200	NA
ERR495201	NA
ERR495202	NA
ERR495203	NA
ERR495204	NA
ERR501638	NA
ERR501639	NA
ERR501640	NA
ERR501641	NA
ERR501642	NA
ERR501643	NA
ERR501644	NA
ERR501645	NA

ERR501646	NA
ERR501647	NA
ERR502240	NA
ERR502241	NA
ERR502242	NA
ERR502243	NA
ERR502244	NA
ERR502245	NA
ERR502246	NA
ERR502247	NA
ERR502248	NA
ERR502249	NA
ERR502250	NA
ERR502251	NA
ERR502252	NA
ERR502253	NA
ERR502254	NA
ERR502255	NA
ERR502256	NA
ERR502257	NA
ERR502258	NA
ERR502259	NA
ERR502260	NA
ERR502261	NA
ERR502262	NA
ERR502263	NA
ERR502264	NA
ERR502265	NA
ERR502266	NA
ERR502267	NA
ERR502268	NA
ERR502269	NA
ERR502270	NA
ERR502271	NA
ERR502272	NA
ERR502273	NA
ERR502274	NA
ERR502275	NA
ERR502276	NA
ERR502277	NA
ERR502278	NA
ERR502279	NA
ERR502280	NA
ERR502281	NA
ERR502282	NA
ERR502283	NA
ERR502284	NA
ERR502285	NA
ERR502286	NA
ERR502287	NA

ERR502288	NA
ERR502289	NA
ERR502290	NA
ERR502291	NA
ERR502292	NA
ERR502293	NA
ERR502294	NA
ERR502295	NA
ERR502296	NA
ERR502297	NA
ERR502298	NA
ERR502299	NA
ERR502300	NA
ERR502301	NA
ERR502302	NA
ERR502303	NA
ERR502304	NA
ERR502305	NA
ERR502306	NA
ERR502307	NA
ERR502308	NA
ERR502309	NA
ERR502310	NA
ERR502311	NA
ERR502312	NA
ERR502313	NA
ERR502314	NA
ERR502315	NA
ERR502316	NA
ERR502317	NA
ERR502318	NA
ERR502319	NA
ERR502320	NA
ERR502321	NA
ERR502322	NA
ERR502323	NA
ERR502324	NA
ERR502325	NA
ERR502326	NA
ERR502327	NA
ERR502328	NA
ERR502329	NA
ERR502330	NA
ERR502331	NA
ERR502332	NA
ERR502333	NA
ERR502334	NA
ERR502335	NA
ERR502336	NA
ERR502337	NA

ERR502338	NA
ERR502339	NA
ERR502340	NA
ERR502341	NA
ERR502342	NA
ERR502343	NA
ERR502344	NA
ERR502345	NA
ERR502346	NA
ERR502347	NA
ERR502348	NA
ERR502349	NA
ERR502350	NA
ERR502351	NA
ERR502352	NA
ERR502353	NA
ERR502354	NA
ERR502355	NA
ERR502356	NA
ERR502357	NA
ERR502358	NA
ERR502359	NA
ERR502360	NA
ERR502361	NA
ERR502362	NA
ERR502363	NA
ERR502364	NA
ERR502365	NA
ERR502366	NA
ERR502367	NA
ERR502368	NA
ERR502369	NA
ERR502370	NA
ERR502371	NA
ERR502372	NA
ERR502373	NA
ERR502374	NA
ERR502375	NA
ERR502376	NA
ERR502377	NA
ERR502378	NA
ERR502379	NA
ERR502380	NA
ERR502381	NA
ERR502382	NA
ERR502383	NA
ERR502384	NA
ERR502385	NA
ERR502386	NA
ERR502387	NA

ERR502388	NA
ERR502389	NA
ERR502390	NA
ERR502391	NA
ERR502392	NA
ERR502393	NA
ERR502394	NA
ERR502395	NA
ERR502396	NA
ERR502397	NA
ERR502398	NA
ERR502399	NA
ERR502400	NA
ERR502401	NA
ERR502402	NA
ERR502403	NA
ERR502404	NA
ERR502405	NA
ERR502406	NA
ERR502407	NA
ERR502408	NA
ERR502409	NA
ERR502410	NA
ERR502411	NA
ERR502412	NA
ERR502413	NA
ERR502414	NA
ERR502415	NA
ERR502416	NA
ERR502417	NA
ERR502418	NA
ERR502419	NA
ERR502420	NA
ERR502421	NA
ERR502422	NA
ERR502423	NA
ERR502424	NA
ERR502425	NA
ERR502426	NA
ERR502427	NA
ERR502428	NA
ERR502429	NA
ERR502884	NA
ERR502885	NA
ERR502886	NA
ERR502887	NA
ERR502888	NA
ERR502889	NA
ERR502890	NA
ERR502891	NA

ERR502892	NA
ERR502893	NA
ERR502894	NA
ERR502895	NA
ERR502896	NA
ERR502897	NA
ERR502898	NA
ERR502899	NA
ERR502900	NA
ERR502901	NA
ERR502902	NA
ERR502903	NA
ERR502904	NA
ERR502905	NA
ERR502906	NA
ERR502907	NA
ERR502908	NA
ERR502909	NA
ERR502910	NA
ERR502911	NA
ERR502912	NA
ERR502913	NA
ERR502914	NA
ERR502915	NA
ERR502916	NA
ERR502917	NA
ERR502918	NA
ERR502919	NA
ERR502920	NA
ERR502921	NA
ERR502922	NA
ERR502923	NA
ERR502924	NA
ERR502925	NA
ERR502926	NA
ERR502927	NA
ERR502928	NA
ERR502929	NA
ERR502930	NA
ERR502931	NA
ERR502932	NA
ERR502933	NA
ERR502934	NA
ERR502935	NA
ERR502936	NA
ERR502937	NA
ERR502938	NA
ERR502939	NA
ERR502940	NA
ERR502941	NA

ERR502942	NA
ERR502943	NA
ERR502944	NA
ERR502945	NA
ERR502946	NA
ERR502947	NA
ERR502948	NA
ERR502949	NA
ERR502950	NA
ERR502951	NA
ERR502952	NA
ERR502953	NA
ERR502954	NA
ERR502955	NA
ERR502956	NA
ERR502957	NA
ERR502958	NA
ERR502959	NA
ERR517399	NA
ERR517400	NA
ERR538422	NA
ERR538423	NA
ERR538424	NA
ERR538425	NA
ERR538426	NA
ERR538427	NA
ERR538428	NA
ERR538429	NA
ERR538430	NA
ERR538431	NA
ERR538432	NA
ERR550405	NA
ERR562804	Singapore
ERR562805	Singapore
ERR562806	Singapore
ERR562807	Singapore
ERR562808	Singapore
ERR562809	Singapore
ERR568778	NA
ERR568779	NA
ERR600630	NA
ERR600631	NA
ERR600632	NA
ERR600633	NA
ERR600634	NA
ERR600635	NA
ERR600636	NA
ERR600637	NA
ERR600638	NA
ERR600639	NA

ERR600640	NA
ERR600641	NA
ERR600642	NA
ERR600643	NA
ERR600644	NA
ERR600645	NA
ERR600646	NA
ERR600647	NA
ERR600648	NA
ERR600649	NA
ERR600650	NA
ERR600651	NA
ERR600652	NA
ERR600653	NA
ERR600654	NA
ERR600655	NA
ERR600656	NA
ERR600657	NA
ERR600658	NA
ERR600659	NA
ERR600660	NA
ERR600661	NA
ERR600662	NA
ERR600663	NA
ERR600664	NA
ERR600665	NA
ERR600666	NA
ERR600667	NA
ERR600668	NA
ERR600669	NA
ERR600670	NA
ERR600671	NA
ERR600672	NA
ERR600673	NA
ERR600674	NA
ERR600675	NA
ERR600676	NA
ERR654964	NA
ERR654965	NA
ERR654966	NA
ERR654967	NA
ERR688008	NA
ERR688009	NA
ERR688010	NA
ERR688011	NA
ERR688012	NA
ERR688013	NA
ERR688014	NA
ERR688015	NA
ERR688016	NA

ERR688017	NA
ERR688018	NA
ERR688019	NA
ERR688020	NA
ERR688021	NA
ERR688022	NA
ERR688023	NA
ERR688024	NA
ERR688025	NA
ERR688026	NA
ERR688027	NA
ERR688028	NA
ERR688029	NA
ERR688030	NA
ERR688031	NA
ERR688032	NA
ERR688033	NA
ERR688034	NA
ERR688035	NA
ERR688036	NA
ERR688037	NA
ERR688038	NA
ERR688039	NA
ERR688040	NA
ERR688041	NA
ERR688042	NA
ERR688043	NA
ERR688044	NA
ERR688045	NA
ERR688046	NA
ERR688047	NA
ERR688048	NA
ERR688049	NA
ERR713575	Cote d'Ivoire
ERR718192	NA
ERR718193	NA
ERR718194	NA
ERR718195	NA
ERR718196	NA
ERR718197	NA
ERR718198	NA
ERR718199	NA
ERR718200	NA
ERR718201	NA
ERR718202	NA
ERR718203	NA
ERR718204	NA
ERR718205	NA
ERR718206	NA
ERR718207	NA

ERR718208	NA
ERR718209	NA
ERR718210	NA
ERR718211	NA
ERR718212	NA
ERR718213	NA
ERR718214	NA
ERR718215	NA
ERR718216	NA
ERR718217	NA
ERR718218	NA
ERR718219	NA
ERR718220	NA
ERR718221	NA
ERR718222	NA
ERR718223	NA
ERR718224	NA
ERR718225	NA
ERR718226	NA
ERR718227	NA
ERR718228	NA
ERR718229	NA
ERR718230	NA
ERR718231	NA
ERR718232	NA
ERR718233	NA
ERR718234	NA
ERR718235	NA
ERR718236	NA
ERR718237	NA
ERR718238	NA
ERR718239	NA
ERR718240	NA
ERR718241	NA
ERR718242	NA
ERR718243	NA
ERR718244	NA
ERR718245	NA
ERR718246	NA
ERR718247	NA
ERR718248	NA
ERR718249	NA
ERR718250	NA
ERR718251	NA
ERR718252	NA
ERR718253	NA
ERR718254	NA
ERR718255	NA
ERR718256	NA
ERR718257	NA

ERR718258	NA
ERR718259	NA
ERR718260	NA
ERR718261	NA
ERR718262	NA
ERR718263	NA
ERR718264	NA
ERR718265	NA
ERR718266	NA
ERR718267	NA
ERR718268	NA
ERR718269	NA
ERR718270	NA
ERR718271	NA
ERR718272	NA
ERR718273	NA
ERR718274	NA
ERR718275	NA
ERR718276	NA
ERR718277	NA
ERR718278	NA
ERR718279	NA
ERR718280	NA
ERR718281	NA
ERR718282	NA
ERR718283	NA
ERR718284	NA
ERR718285	NA
ERR718286	NA
ERR718287	NA
ERR718288	NA
ERR718289	NA
ERR718290	NA
ERR718291	NA
ERR718292	NA
ERR718293	NA
ERR718294	NA
ERR718295	NA
ERR718296	NA
ERR718297	NA
ERR718298	NA
ERR718299	NA
ERR718300	NA
ERR718301	NA
ERR718302	NA
ERR718303	NA
ERR718304	NA
ERR718305	NA
ERR718306	NA
ERR718307	NA

ERR718308	NA
ERR718309	NA
ERR718310	NA
ERR718311	NA
ERR718312	NA
ERR718313	NA
ERR718314	NA
ERR718315	NA
ERR718316	NA
ERR718317	NA
ERR718318	NA
ERR718319	NA
ERR718320	NA
ERR718321	NA
ERR718322	NA
ERR718323	NA
ERR718324	NA
ERR718325	NA
ERR718326	NA
ERR718327	NA
ERR718328	NA
ERR718329	NA
ERR718330	NA
ERR718331	NA
ERR718332	NA
ERR718333	NA
ERR718334	NA
ERR718335	NA
ERR718336	NA
ERR718337	NA
ERR718338	NA
ERR718339	NA
ERR718340	NA
ERR718341	NA
ERR718342	NA
ERR718343	NA
ERR718344	NA
ERR718345	NA
ERR718346	NA
ERR718347	NA
ERR718348	NA
ERR718349	NA
ERR718350	NA
ERR718351	NA
ERR718352	NA
ERR718353	NA
ERR718354	NA
ERR718355	NA
ERR718356	NA
ERR718357	NA

ERR718358	NA
ERR718359	NA
ERR718360	NA
ERR718361	NA
ERR718362	NA
ERR718363	NA
ERR718364	NA
ERR718365	NA
ERR718366	NA
ERR718367	NA
ERR718368	NA
ERR718369	NA
ERR718370	NA
ERR718371	NA
ERR718372	NA
ERR718373	NA
ERR718374	NA
ERR718375	NA
ERR718376	NA
ERR718377	NA
ERR718378	NA
ERR718379	NA
ERR718380	NA
ERR718381	NA
ERR718382	NA
ERR718383	NA
ERR718384	NA
ERR718385	NA
ERR718386	NA
ERR718387	NA
ERR718388	NA
ERR718389	NA
ERR718390	NA
ERR718391	NA
ERR718392	NA
ERR718393	NA
ERR718394	NA
ERR718395	NA
ERR718396	NA
ERR718397	NA
ERR718398	NA
ERR718399	NA
ERR718400	NA
ERR718401	NA
ERR718402	NA
ERR718403	NA
ERR718404	NA
ERR718405	NA
ERR718406	NA
ERR718407	NA

ERR718408	NA
ERR718409	NA
ERR718410	NA
ERR718411	NA
ERR718412	NA
ERR718413	NA
ERR718414	NA
ERR718415	NA
ERR718416	NA
ERR718417	NA
ERR718418	NA
ERR718419	NA
ERR718420	NA
ERR718421	NA
ERR718422	NA
ERR718423	NA
ERR718424	NA
ERR718425	NA
ERR718426	NA
ERR718427	NA
ERR718428	NA
ERR718429	NA
ERR718430	NA
ERR718431	NA
ERR718432	NA
ERR718433	NA
ERR718434	NA
ERR718435	NA
ERR718436	NA
ERR718437	NA
ERR718438	NA
ERR718439	NA
ERR718440	NA
ERR718441	NA
ERR718442	NA
ERR718443	NA
ERR718444	NA
ERR718445	NA
ERR718446	NA
ERR718447	NA
ERR718448	NA
ERR718449	NA
ERR718450	NA
ERR718451	NA
ERR718452	NA
ERR718453	NA
ERR718454	NA
ERR718455	NA
ERR718456	NA
ERR718457	NA

ERR718458	NA
ERR718459	NA
ERR718460	NA
ERR718461	NA
ERR718462	NA
ERR718463	NA
ERR718464	NA
ERR718465	NA
ERR718466	NA
ERR718467	NA
ERR718468	NA
ERR718469	NA
ERR718470	NA
ERR718471	NA
ERR718472	NA
ERR718473	NA
ERR718474	NA
ERR718475	NA
ERR718476	NA
ERR718477	NA
ERR718478	NA
ERR718479	NA
ERR718480	NA
ERR718481	NA
ERR718482	NA
ERR718483	NA
ERR718484	NA
ERR718485	NA
ERR718486	NA
ERR718487	NA
ERR718488	NA
ERR718489	NA
ERR718490	NA
ERR718491	NA
ERR718492	NA
ERR718493	NA
ERR718494	NA
ERR718495	NA
ERR718496	NA
ERR718497	NA
ERR718498	NA
ERR718499	NA
ERR718500	NA
ERR718501	NA
ERR718502	NA
ERR718503	NA
ERR718504	NA
ERR718505	NA
ERR718506	NA
ERR718507	NA

ERR718508	NA
ERR718509	NA
ERR718510	NA
ERR718511	NA
ERR718512	NA
ERR718513	NA
ERR718514	NA
ERR718515	NA
ERR718516	NA
ERR718517	NA
ERR718518	NA
ERR718519	NA
ERR718520	NA
ERR718521	NA
ERR718522	NA
ERR718523	NA
ERR718524	NA
ERR718525	NA
ERR718526	NA
ERR718527	NA
ERR718528	NA
ERR718529	NA
ERR718530	NA
ERR718531	NA
ERR718532	NA
ERR718533	NA
ERR718534	NA
ERR718535	NA
ERR718536	NA
ERR718537	NA
ERR718538	NA
ERR718539	NA
ERR718540	NA
ERR718541	NA
ERR718542	NA
ERR718543	NA
ERR718544	NA
ERR718545	NA
ERR718546	NA
ERR718547	NA
ERR718548	NA
ERR718549	NA
ERR718550	NA
ERR718551	NA
ERR718552	NA
ERR718553	NA
ERR718554	NA
ERR718555	NA
ERR718556	NA
ERR718557	NA

ERR718558	NA
ERR718559	NA
ERR718560	NA
ERR718561	NA
ERR718562	NA
ERR718563	NA
ERR736803	NA
ERR736804	NA
ERR736805	NA
ERR736806	NA
ERR736807	NA
ERR736808	NA
ERR736809	NA
ERR736810	NA
ERR736811	NA
ERR736812	NA
ERR736813	NA
ERR736814	NA
ERR736815	NA
ERR736816	NA
ERR736817	NA
ERR736818	NA
ERR736819	NA
ERR736820	NA
ERR736821	NA
ERR736822	NA
ERR736823	NA
ERR736824	NA
ERR736825	NA
ERR736826	NA
ERR736827	NA
ERR736828	NA
ERR736829	NA
ERR736830	NA
ERR736831	NA
ERR736832	NA
ERR736833	NA
ERR736834	NA
ERR736835	NA
ERR736836	NA
ERR736837	NA
ERR736838	NA
ERR736839	NA
ERR736840	NA
ERR736841	NA
ERR736842	NA
ERR736843	NA
ERR736844	NA
ERR736845	NA
ERR736846	NA

ERR736847	NA
ERR736848	NA
ERR736849	NA
ERR736850	NA
ERR736851	NA
ERR736852	NA
ERR736853	NA
ERR736854	NA
ERR736855	NA
ERR736856	NA
ERR736857	NA
ERR736858	NA
ERR736859	NA
ERR736860	NA
ERR736861	NA
ERR736862	NA
ERR736863	NA
ERR736864	NA
ERR736865	NA
ERR736866	NA
ERR736867	NA
ERR736868	NA
ERR736869	NA
ERR736870	NA
ERR736871	NA
ERR736872	NA
ERR736873	NA
ERR736874	NA
ERR736875	NA
ERR736876	NA
ERR736877	NA
ERR736878	NA
ERR736879	NA
ERR736880	NA
ERR736881	NA
ERR736882	NA
ERR744331	NA
ERR744332	NA
ERR744333	NA
ERR744334	NA
ERR744335	NA
ERR744336	NA
ERR744337	NA
ERR744338	NA
ERR744339	NA
ERR744340	NA
ERR751350	Peru
ERR751351	Peru
ERR751352	Peru
ERR751353	Peru

ERR751354	Peru
ERR751355	Peru
ERR751356	Peru
ERR751357	Peru
ERR751358	Peru
ERR751359	Peru
ERR751360	Peru
ERR751361	Peru
ERR751362	Peru
ERR751363	Peru
ERR751364	Peru
ERR751365	Peru
ERR751366	Peru
ERR751367	Peru
ERR751368	Peru
ERR751369	Peru
ERR751370	Peru
ERR751371	Peru
ERR751372	Peru
ERR751373	Peru
ERR751374	Peru
ERR751375	Peru
ERR751376	Peru
ERR751377	Peru
ERR751378	Peru
ERR751379	Peru
ERR751380	Peru
ERR751381	Peru
ERR751382	Peru
ERR751383	Peru
ERR751384	Peru
ERR751385	Peru
ERR751386	Peru
ERR751387	Peru
ERR751388	Peru
ERR751389	Peru
ERR751390	Peru
ERR751391	Peru
ERR751392	Peru
ERR751393	Peru
ERR751394	Peru
ERR751395	Peru
ERR751396	Peru
ERR751397	Peru
ERR751398	Peru
ERR751399	Peru
ERR751400	Peru
ERR751401	Peru
ERR751402	Peru
ERR751403	Peru

ERR751404	Peru
ERR751405	Peru
ERR751406	Peru
ERR751407	Peru
ERR751408	Peru
ERR751409	Peru
ERR751410	Peru
ERR751411	Peru
ERR751412	Peru
ERR751413	Peru
ERR751414	Peru
ERR751415	Peru
ERR751416	Peru
ERR751417	Peru
ERR751418	Peru
ERR751419	Peru
ERR751420	Peru
ERR751421	Peru
ERR751422	Peru
ERR751423	Peru
ERR751424	Peru
ERR751425	Peru
ERR751426	Peru
ERR751427	Peru
ERR751428	Peru
ERR751429	Peru
ERR751430	Peru
ERR751431	Peru
ERR751432	Peru
ERR751433	Peru
ERR751434	Peru
ERR751435	Peru
ERR751436	Peru
ERR751437	Peru
ERR751438	Peru
ERR751439	Peru
ERR751440	Peru
ERR751441	Peru
ERR751442	Peru
ERR751443	Peru
ERR751444	Peru
ERR751445	Peru
ERR751446	Peru
ERR751447	Peru
ERR751448	Peru
ERR751449	Peru
ERR751450	Peru
ERR751451	Peru
ERR751452	Peru
ERR751453	Peru

ERR751454	Peru
ERR751455	Peru
ERR751456	Peru
ERR751457	Peru
ERR751458	Peru
ERR751459	Peru
ERR751460	Peru
ERR751461	Peru
ERR751462	Peru
ERR751463	Peru
ERR751464	Peru
ERR751465	Peru
ERR751466	Peru
ERR751467	Peru
ERR751468	Peru
ERR751469	Peru
ERR751470	Peru
ERR751471	Peru
ERR751472	Peru
ERR751473	Peru
ERR751474	Peru
ERR751475	Peru
ERR751476	Peru
ERR751477	Peru
ERR751478	Peru
ERR751479	Peru
ERR751480	Peru
ERR751481	Peru
ERR751482	Peru
ERR751483	Peru
ERR751484	Peru
ERR751485	Peru
ERR751486	Peru
ERR751487	Peru
ERR751488	Peru
ERR751489	Peru
ERR751490	Peru
ERR751491	Peru
ERR751492	Peru
ERR751493	Peru
ERR751494	Peru
ERR751495	Peru
ERR751496	Peru
ERR751497	Peru
ERR751498	Peru
ERR751499	Peru
ERR751500	Peru
ERR751501	Peru
ERR751502	Peru
ERR751503	Peru

ERR751504	Peru
ERR751505	Peru
ERR751506	Peru
ERR751507	Peru
ERR751508	Peru
ERR751509	Peru
ERR751510	Peru
ERR751511	Peru
ERR751512	Peru
ERR751513	Peru
ERR751514	Peru
ERR751515	Peru
ERR751516	Peru
ERR751517	Peru
ERR751518	Peru
ERR751519	Peru
ERR751520	Peru
ERR751521	Peru
ERR751522	Peru
ERR751523	Peru
ERR751524	Peru
ERR751525	Peru
ERR751526	Peru
ERR751527	Peru
ERR751528	Peru
ERR751529	Peru
ERR751530	Peru
ERR751531	Peru
ERR751532	Peru
ERR751533	Peru
ERR751534	Peru
ERR751535	Peru
ERR751536	Peru
ERR751537	Peru
ERR751538	Peru
ERR751539	Peru
ERR751540	Peru
ERR751541	Peru
ERR751542	Peru
ERR751543	Peru
ERR751544	Peru
ERR751545	Peru
ERR751546	Peru
ERR751547	Peru
ERR751548	Peru
ERR751549	Peru
ERR751550	Peru
ERR751551	Peru
ERR751552	Peru
ERR751553	Peru

ERR751554	Peru
ERR751555	Peru
ERR751556	Peru
ERR751557	Peru
ERR751558	Peru
ERR751559	Peru
ERR751560	Peru
ERR751561	Peru
ERR751562	Peru
ERR751563	Peru
ERR751564	Peru
ERR751565	Peru
ERR751566	Peru
ERR751567	Peru
ERR751568	Peru
ERR751569	Peru
ERR751570	Peru
ERR751571	Peru
ERR751572	Peru
ERR751573	Peru
ERR751574	Peru
ERR751575	Peru
ERR751576	Peru
ERR751577	Peru
ERR751578	Peru
ERR751579	Peru
ERR751580	Peru
ERR751581	Peru
ERR751582	Peru
ERR751583	Peru
ERR751584	Peru
ERR751585	Peru
ERR751586	Peru
ERR751587	Peru
ERR751588	Peru
ERR751589	Peru
ERR751590	Peru
ERR751591	Peru
ERR751592	Peru
ERR751593	Peru
ERR751594	Peru
ERR751595	Peru
ERR751596	Peru
ERR751597	Peru
ERR751598	Peru
ERR751599	Peru
ERR751600	Peru
ERR751601	Peru
ERR751602	Peru
ERR751603	Peru

ERR751604	Peru
ERR751605	Peru
ERR751606	Peru
ERR751607	Peru
ERR751608	Peru
ERR751609	Peru
ERR751610	Peru
ERR751611	Peru
ERR751612	Peru
ERR751613	Peru
ERR751614	Peru
ERR751615	Peru
ERR751616	Peru
ERR751617	Peru
ERR751618	Peru
ERR751619	Peru
ERR751620	Peru
ERR751621	Peru
ERR751622	Peru
ERR751623	Peru
ERR751624	Peru
ERR751625	Peru
ERR751626	Peru
ERR751627	Peru
ERR751628	Peru
ERR751629	Peru
ERR751630	Peru
ERR751631	Peru
ERR751632	Peru
ERR751633	Peru
ERR751634	Peru
ERR751635	NA
ERR751636	NA
ERR751637	NA
ERR751638	NA
ERR751639	NA
ERR751640	NA
ERR751641	NA
ERR751642	NA
ERR751643	NA
ERR751644	NA
ERR751645	NA
ERR751646	NA
ERR751647	NA
ERR751648	NA
ERR751649	NA
ERR751650	NA
ERR751651	NA
ERR751652	NA
ERR751653	NA

ERR751654	NA
ERR751655	NA
ERR751656	NA
ERR751657	NA
ERR751658	NA
ERR751659	NA
ERR751660	NA
ERR751661	NA
ERR751662	NA
ERR751663	NA
ERR751664	NA
ERR751665	NA
ERR751666	NA
ERR751667	NA
ERR751668	NA
ERR751669	NA
ERR751670	NA
ERR751671	NA
ERR751672	NA
ERR751673	NA
ERR751674	NA
ERR751675	NA
ERR751676	NA
ERR751677	NA
ERR751678	NA
ERR751679	NA
ERR751680	NA
ERR751681	NA
ERR751682	NA
ERR751683	NA
ERR751684	NA
ERR751685	NA
ERR751686	NA
ERR751687	NA
ERR751688	NA
ERR751689	NA
ERR751690	NA
ERR751691	NA
ERR751692	NA
ERR751693	NA
ERR751694	NA
ERR751695	NA
ERR751696	NA
ERR751697	NA
ERR751698	NA
ERR751699	NA
ERR751700	NA
ERR751701	NA
ERR751702	NA
ERR751703	NA

ERR751704	NA
ERR751705	NA
ERR751706	NA
ERR751707	NA
ERR751708	NA
ERR751709	NA
ERR751710	NA
ERR751711	NA
ERR751712	NA
ERR751713	NA
ERR751714	NA
ERR751715	NA
ERR751716	NA
ERR751717	NA
ERR751718	NA
ERR751719	NA
ERR751720	NA
ERR751721	NA
ERR751722	NA
ERR751723	NA
ERR751724	NA
ERR751725	NA
ERR751726	NA
ERR751727	NA
ERR751728	NA
ERR751729	NA
ERR751730	NA
ERR751731	NA
ERR751732	NA
ERR751733	NA
ERR751734	NA
ERR751735	NA
ERR751736	NA
ERR751737	NA
ERR751738	NA
ERR751739	NA
ERR751740	NA
ERR751741	NA
ERR751742	NA
ERR751743	NA
ERR751744	NA
ERR751745	NA
ERR751746	NA
ERR751747	NA
ERR751748	NA
ERR751749	NA
ERR751750	NA
ERR751751	NA
ERR751752	NA
ERR751753	NA

ERR751754	NA
ERR751755	NA
ERR751756	NA
ERR751757	NA
ERR751758	NA
ERR751759	NA
ERR751760	NA
ERR751761	NA
ERR751762	NA
ERR751763	NA
ERR751764	NA
ERR751765	NA
ERR751766	NA
ERR751767	NA
ERR751768	NA
ERR751769	NA
ERR751770	NA
ERR751771	NA
ERR751772	NA
ERR751773	NA
ERR751774	NA
ERR751775	NA
ERR751776	NA
ERR751777	NA
ERR751778	NA
ERR751779	NA
ERR751780	NA
ERR751781	NA
ERR751782	NA
ERR751783	NA
ERR751784	NA
ERR751785	NA
ERR751786	NA
ERR751787	NA
ERR751788	NA
ERR751789	NA
ERR751790	NA
ERR751791	NA
ERR751792	NA
ERR751793	NA
ERR751794	NA
ERR751795	NA
ERR751796	NA
ERR751797	NA
ERR751798	NA
ERR751799	NA
ERR751800	NA
ERR751801	NA
ERR751802	NA
ERR751803	NA

ERR751804	NA
ERR751805	NA
ERR751806	NA
ERR751807	NA
ERR751808	NA
ERR751809	NA
ERR751810	NA
ERR751811	NA
ERR751812	NA
ERR751813	NA
ERR751814	NA
ERR751815	NA
ERR751816	NA
ERR751817	NA
ERR751818	NA
ERR751819	NA
ERR751820	NA
ERR751821	NA
ERR751822	NA
ERR751823	NA
ERR751824	NA
ERR751825	NA
ERR751826	NA
ERR751827	NA
ERR751828	NA
ERR751829	NA
ERR751830	NA
ERR751831	NA
ERR751832	NA
ERR751833	NA
ERR751834	NA
ERR751835	NA
ERR751836	NA
ERR751837	NA
ERR751838	NA
ERR751839	NA
ERR751840	NA
ERR751841	NA
ERR751842	NA
ERR751843	NA
ERR751844	NA
ERR751845	NA
ERR751846	NA
ERR751847	NA
ERR751848	NA
ERR751849	NA
ERR751850	NA
ERR751851	NA
ERR751852	NA
ERR751853	NA

ERR751854	NA
ERR751855	NA
ERR751856	NA
ERR751857	NA
ERR751858	NA
ERR751859	NA
ERR751860	NA
ERR751861	NA
ERR751862	NA
ERR751863	NA
ERR751864	NA
ERR751865	NA
ERR751866	NA
ERR751867	NA
ERR751868	NA
ERR751869	NA
ERR751870	NA
ERR751871	NA
ERR751872	NA
ERR751873	NA
ERR751874	NA
ERR751875	NA
ERR751876	NA
ERR751877	NA
ERR751878	NA
ERR751879	NA
ERR751880	NA
ERR751881	NA
ERR751882	NA
ERR751883	NA
ERR751884	NA
ERR751885	NA
ERR751886	NA
ERR751887	NA
ERR751888	NA
ERR751889	NA
ERR751890	NA
ERR751891	NA
ERR751892	NA
ERR751893	NA
ERR751894	NA
ERR751895	NA
ERR751896	NA
ERR751897	NA
ERR751898	NA
ERR751899	NA
ERR751900	NA
ERR751901	NA
ERR751902	NA
ERR751903	NA

ERR751904	NA
ERR751905	NA
ERR751906	NA
ERR751907	NA
ERR751908	NA
ERR751909	NA
ERR751910	NA
ERR751911	NA
ERR751912	NA
ERR751913	NA
ERR751914	NA
ERR751915	NA
ERR751916	NA
ERR751917	NA
ERR751918	NA
ERR751919	NA
ERR751920	NA
ERR751921	NA
ERR751922	NA
ERR751923	NA
ERR751924	NA
ERR751925	NA
ERR751926	NA
ERR751927	NA
ERR751928	NA
ERR751929	NA
ERR751930	NA
ERR751931	NA
ERR751932	NA
ERR751933	NA
ERR751934	NA
ERR751935	NA
ERR751936	NA
ERR751937	NA
ERR751938	NA
ERR751939	NA
ERR751940	NA
ERR751941	NA
ERR751942	NA
ERR751943	NA
ERR751944	NA
ERR751945	NA
ERR751946	NA
ERR751947	NA
ERR751948	NA
ERR751949	NA
ERR751950	NA
ERR751951	NA
ERR751952	NA
ERR751953	NA

ERR751954	NA
ERR751955	NA
ERR751956	NA
ERR751957	NA
ERR751958	NA
ERR751959	NA
ERR751960	NA
ERR751961	NA
ERR751962	NA
ERR751963	NA
ERR751964	NA
ERR751965	NA
ERR751966	NA
ERR751967	NA
ERR751968	NA
ERR751969	NA
ERR751970	NA
ERR751971	NA
ERR751972	NA
ERR751973	NA
ERR751974	NA
ERR751975	NA
ERR751976	NA
ERR751977	NA
ERR751978	NA
ERR751979	NA
ERR751980	NA
ERR751981	NA
ERR751982	NA
ERR751983	NA
ERR751984	NA
ERR751985	NA
ERR751986	NA
ERR751987	NA
ERR751988	NA
ERR751989	NA
ERR751990	NA
ERR751991	NA
ERR751992	NA
ERR751993	NA
ERR751994	NA
ERR751995	NA
ERR751996	NA
ERR751997	NA
ERR751998	NA
ERR751999	NA
ERR752000	NA
ERR752001	NA
ERR752002	NA
ERR752003	NA

ERR752004	NA
ERR752005	NA
ERR752006	NA
ERR752007	NA
ERR752008	NA
ERR752009	NA
ERR752010	NA
ERR752011	NA
ERR752012	NA
ERR752013	NA
ERR752014	NA
ERR752015	NA
ERR752016	NA
ERR752017	NA
ERR752018	NA
ERR752019	NA
ERR752020	NA
ERR752021	NA
ERR752022	NA
ERR752023	NA
ERR752024	NA
ERR752025	NA
ERR752026	NA
ERR752027	NA
ERR752028	NA
ERR752029	NA
ERR752030	NA
ERR752031	NA
ERR752032	NA
ERR752033	NA
ERR752034	NA
ERR752035	NA
ERR752036	NA
ERR752037	NA
ERR752038	NA
ERR752039	NA
ERR752040	NA
ERR752041	NA
ERR752042	NA
ERR752043	NA
ERR752044	NA
ERR752045	NA
ERR752046	NA
ERR752047	NA
ERR752048	NA
ERR752049	NA
ERR752050	NA
ERR752051	NA
ERR752052	NA
ERR752053	NA

ERR752054	NA
ERR752055	NA
ERR752056	NA
ERR752057	NA
ERR752058	NA
ERR752059	NA
ERR752060	NA
ERR752061	NA
ERR752062	NA
ERR752063	NA
ERR752064	NA
ERR752065	NA
ERR752066	NA
ERR752067	NA
ERR752068	NA
ERR752069	NA
ERR752070	NA
ERR752071	NA
ERR752072	NA
ERR752073	NA
ERR752074	NA
ERR752075	NA
ERR752076	NA
ERR752077	NA
ERR752078	NA
ERR752079	NA
ERR752080	NA
ERR752081	NA
ERR752082	NA
ERR752083	NA
ERR752084	NA
ERR752085	NA
ERR752086	NA
ERR752087	NA
ERR752088	NA
ERR752089	NA
ERR752090	NA
ERR752091	NA
ERR752092	NA
ERR752093	NA
ERR752094	NA
ERR752095	NA
ERR752096	NA
ERR752097	NA
ERR752098	NA
ERR752099	NA
ERR752100	NA
ERR752101	NA
ERR752102	NA
ERR752103	NA

ERR752104	NA
ERR752105	NA
ERR752106	NA
ERR752107	NA
ERR752108	NA
ERR752109	NA
ERR752110	NA
ERR752111	NA
ERR752112	NA
ERR752113	NA
ERR752114	NA
ERR752115	NA
ERR752116	NA
ERR752117	NA
ERR752118	NA
ERR752119	NA
ERR752120	NA
ERR752121	NA
ERR752122	NA
ERR752123	NA
ERR752124	NA
ERR752125	NA
ERR752126	NA
ERR752127	NA
ERR752128	NA
ERR752129	NA
ERR752130	NA
ERR752131	NA
ERR752132	NA
ERR752133	NA
ERR752134	NA
ERR752135	NA
ERR752136	NA
ERR752137	NA
ERR752138	NA
ERR752139	NA
ERR752140	NA
ERR752141	NA
ERR752142	NA
ERR752143	NA
ERR752144	NA
ERR752145	NA
ERR752146	NA
ERR752147	NA
ERR752148	NA
ERR752149	NA
ERR752150	NA
ERR752151	NA
ERR752152	NA
ERR752153	NA

ERR752154	NA
ERR752155	NA
ERR752156	NA
ERR752157	NA
ERR752158	NA
ERR752159	NA
ERR752160	NA
ERR752161	NA
ERR752162	NA
ERR752163	NA
ERR752164	NA
ERR752165	NA
ERR752166	NA
ERR752167	NA
ERR752168	NA
ERR752169	NA
ERR752170	NA
ERR752171	NA
ERR752172	NA
ERR752173	NA
ERR752174	NA
ERR752175	NA
ERR752176	NA
ERR752177	NA
ERR752178	NA
ERR752179	NA
ERR752180	NA
ERR752181	NA
ERR752182	NA
ERR752183	NA
ERR752184	NA
ERR752185	NA
ERR752186	NA
ERR752187	NA
ERR752188	NA
ERR752189	NA
ERR752190	NA
ERR752191	NA
ERR752192	NA
ERR752193	NA
ERR752194	NA
ERR752195	NA
ERR752196	NA
ERR752197	NA
ERR752198	NA
ERR752199	NA
ERR752200	NA
ERR752201	NA
ERR752202	NA
ERR752203	NA

ERR752204	NA
ERR752205	NA
ERR752206	NA
ERR752207	NA
ERR752208	NA
ERR752209	NA
ERR752210	NA
ERR752211	NA
ERR752212	NA
ERR752213	NA
ERR752214	NA
ERR752215	NA
ERR752216	NA
ERR752217	NA
ERR752218	NA
ERR752219	NA
ERR752220	NA
ERR752221	NA
ERR752222	NA
ERR752223	NA
ERR752224	NA
ERR752225	NA
ERR752226	NA
ERR752227	NA
ERR752228	NA
ERR752229	NA
ERR752230	NA
ERR752231	NA
ERR752232	NA
ERR752233	NA
ERR752234	NA
ERR752235	NA
ERR752236	NA
ERR752237	NA
ERR752238	NA
ERR752239	NA
ERR752240	NA
ERR752241	NA
ERR752242	NA
ERR752243	NA
ERR752244	NA
ERR752245	NA
ERR752246	NA
ERR752247	NA
ERR752248	NA
ERR752249	NA
ERR752250	NA
ERR752251	NA
ERR752252	NA
ERR752253	NA

ERR752254	NA
ERR752255	NA
ERR752256	NA
ERR752257	NA
ERR752258	NA
ERR752259	NA
ERR752260	NA
ERR752261	NA
ERR752262	NA
ERR752263	NA
ERR752264	NA
ERR752265	NA
ERR752266	NA
ERR752267	NA
ERR752268	NA
ERR752269	NA
ERR752270	NA
ERR752271	NA
ERR752272	NA
ERR752273	NA
ERR752274	NA
ERR752275	NA
ERR752276	NA
ERR752277	NA
ERR752278	NA
ERR752279	NA
ERR752280	NA
ERR752281	NA
ERR752282	NA
ERR752283	NA
ERR752284	NA
ERR752285	NA
ERR752286	NA
ERR752287	NA
ERR752288	NA
ERR752289	NA
ERR752290	NA
ERR752291	NA
ERR752398	NA
ERR752399	NA
ERR752400	NA
ERR752401	NA
ERR752402	NA
ERR752403	NA
ERR752404	NA
ERR752405	NA
ERR752406	NA
ERR752407	NA
ERR756344	Ethiopia
ERR756344	NA

ERR756345	Ethiopia
ERR756346	Ethiopia
ERR756347	Ethiopia
ERR756348	Ethiopia
ERR757145	Argentina
ERR757146	Argentina
ERR757147	Argentina
ERR757148	Argentina
ERR757149	Argentina
ERR757150	Argentina
ERR757151	Argentina
ERR757152	Argentina
ERR757159	Argentina
ERR757160	Argentina
ERR757161	Argentina
ERR757162	Argentina
ERR757163	Argentina
ERR757164	Argentina
ERR757165	Argentina
ERR757166	Argentina
ERR757167	Argentina
ERR757168	Argentina
ERR757169	Argentina
ERR757170	Argentina
ERR757171	Argentina
ERR757172	Argentina
ERR757173	Argentina
ERR757174	Argentina
ERR757175	Argentina
ERR757176	Argentina
ERR757177	Argentina
ERR757178	Argentina
ERR757179	Argentina
ERR757180	Argentina
ERR757181	Argentina
ERR757182	Argentina
ERR757183	Argentina
ERR757184	Argentina
ERR757185	Argentina
ERR757186	Argentina
ERR757187	Argentina
ERR757188	Argentina
ERR757189	Argentina
ERR757353	NA
ERR757417	NA
ERR758379	Argentina
ERR760595	Argentina
ERR760596	Argentina
ERR760597	Argentina
ERR760598	Argentina

ERR760599	Argentina
ERR760600	Argentina
ERR760601	Argentina
ERR760602	Argentina
ERR760603	Argentina
ERR760604	Argentina
ERR760605	Argentina
ERR760607	Argentina
ERR760608	Argentina
ERR760609	Argentina
ERR760610	Argentina
ERR760611	Argentina
ERR760612	Argentina
ERR760731	Argentina
ERR760732	Argentina
ERR760733	Argentina
ERR760734	Argentina
ERR760735	Argentina
ERR760736	Argentina
ERR760737	Argentina
ERR760738	Argentina
ERR760739	Argentina
ERR760740	Argentina
ERR760741	Argentina
ERR760742	Argentina
ERR760743	Argentina
ERR760744	Argentina
ERR760745	Argentina
ERR760746	Argentina
ERR760747	Argentina
ERR760748	Argentina
ERR760749	Argentina
ERR760750	Argentina
ERR760751	Argentina
ERR760752	Argentina
ERR760753	Argentina
ERR760754	Argentina
ERR760755	Argentina
ERR760756	Argentina
ERR760757	Argentina
ERR760758	Argentina
ERR760759	Argentina
ERR760760	Argentina
ERR760761	Argentina
ERR760762	Argentina
ERR760763	Argentina
ERR760764	Argentina
ERR760765	Argentina
ERR760766	Argentina
ERR760767	Argentina

ERR760768	Argentina
ERR760778	Argentina
ERR760779	Argentina
ERR760781	Argentina
ERR760782	Argentina
ERR760783	Argentina
ERR760784	Argentina
ERR760785	Argentina
ERR760786	Argentina
ERR760787	Argentina
ERR760788	Argentina
ERR760789	Argentina
ERR760790	Argentina
ERR760791	Argentina
ERR760792	Argentina
ERR760793	Argentina
ERR760794	Argentina
ERR760795	Argentina
ERR760796	Argentina
ERR760797	Argentina
ERR760798	Argentina
ERR760799	Argentina
ERR760800	Argentina
ERR760801	Argentina
ERR760802	Argentina
ERR760803	Argentina
ERR760809	Argentina
ERR760810	Argentina
ERR760811	Argentina
ERR760812	Argentina
ERR760813	Argentina
ERR760814	Argentina
ERR760815	Argentina
ERR760816	Argentina
ERR760817	Argentina
ERR760818	Argentina
ERR760819	Argentina
ERR760820	Argentina
ERR760821	Argentina
ERR760822	Argentina
ERR760823	Argentina
ERR760824	Argentina
ERR760825	Argentina
ERR760826	Argentina
ERR760827	Argentina
ERR760828	Argentina
ERR760829	Argentina
ERR760830	Argentina
ERR760831	Argentina
ERR760832	Argentina

ERR760833	Argentina
ERR760834	Argentina
ERR760835	Argentina
ERR760836	Argentina
ERR760837	Argentina
ERR760838	Argentina
ERR760839	Argentina
ERR760840	Argentina
ERR760841	Argentina
ERR760842	Argentina
ERR760843	Argentina
ERR760844	Argentina
ERR760845	Argentina
ERR760846	Argentina
ERR760847	Argentina
ERR760848	Argentina
ERR760849	Argentina
ERR760850	Argentina
ERR760851	Argentina
ERR760852	Argentina
ERR760853	Argentina
ERR760854	Argentina
ERR760855	Argentina
ERR760856	Argentina
ERR760857	Argentina
ERR760858	Argentina
ERR760859	Argentina
ERR760860	Argentina
ERR760861	Argentina
ERR760862	Argentina
ERR760863	Argentina
ERR760864	Argentina
ERR760865	Argentina
ERR760866	Argentina
ERR760867	Argentina
ERR760868	Argentina
ERR760869	Argentina
ERR760870	Argentina
ERR760871	Argentina
ERR760872	Argentina
ERR760873	Argentina
ERR760874	Argentina
ERR760875	Argentina
ERR760876	Argentina
ERR760877	Argentina
ERR760878	Argentina
ERR760879	Argentina
ERR760880	Argentina
ERR760881	Argentina
ERR760882	Argentina

ERR760883	Argentina
ERR760884	Argentina
ERR760885	Argentina
ERR760886	Argentina
ERR760887	Argentina
ERR760888	Argentina
ERR760889	Argentina
ERR760890	Argentina
ERR760891	Argentina
ERR760892	Argentina
ERR760893	Argentina
ERR760894	Argentina
ERR760895	Argentina
ERR760896	Argentina
ERR760897	Argentina
ERR760899	Argentina
ERR760900	Argentina
ERR760901	Argentina
ERR760902	Argentina
ERR760903	Argentina
ERR760904	Argentina
ERR760905	Argentina
ERR760906	Argentina
ERR760907	Argentina
ERR760908	Argentina
ERR760909	Argentina
ERR760910	Argentina
ERR760911	Argentina
ERR760912	Argentina
ERR760913	Argentina
ERR760914	Argentina
ERR760915	Argentina
ERR760916	Argentina
ERR760917	Argentina
ERR760918	Argentina
ERR760919	Argentina
ERR760920	Argentina
ERR760921	Argentina
ERR760922	Argentina
ERR760923	Argentina
ERR760924	Argentina
ERR760925	Argentina
ERR760926	Argentina
ERR760927	Argentina
ERR762352	Argentina
ERR762353	Argentina
ERR767957	NA
ERR767958	NA
ERR767959	NA
ERR767960	NA

ERR767961	NA
ERR767962	NA
ERR767963	NA
ERR767964	NA
ERR767965	NA
ERR767966	NA
ERR767967	NA
ERR767968	NA
ERR767969	NA
ERR767970	NA
ERR767971	NA
ERR767972	NA
ERR767973	NA
ERR767974	NA
ERR767975	NA
ERR767976	NA
ERR767977	NA
ERR767978	NA
ERR767979	NA
ERR767980	NA
ERR767981	NA
ERR767982	NA
ERR767983	NA
ERR767984	NA
ERR767985	NA
ERR767986	NA
ERR767987	NA
ERR767988	NA
ERR767989	NA
ERR767990	NA
ERR767991	NA
ERR767992	NA
ERR767993	NA
ERR767994	NA
ERR767995	NA
ERR767996	NA
ERR767997	NA
ERR767998	NA
ERR767999	NA
ERR768000	NA
ERR768001	NA
ERR768002	NA
ERR768003	NA
ERR768004	NA
ERR768005	NA
ERR768006	NA
ERR768007	NA
ERR768008	NA
ERR768009	NA
ERR768010	NA

ERR768011	NA
ERR768012	NA
ERR768013	NA
ERR768014	NA
ERR768015	NA
ERR768016	NA
ERR768017	NA
ERR768018	NA
ERR768019	NA
ERR768020	NA
ERR768021	NA
ERR768022	NA
ERR768023	NA
ERR768024	NA
ERR768025	NA
ERR768026	NA
ERR768027	NA
ERR768028	NA
ERR768029	NA
ERR768030	NA
ERR768031	NA
ERR768032	NA
ERR768033	NA
ERR768034	NA
ERR768035	NA
ERR768036	NA
ERR768037	NA
ERR768038	NA
ERR768039	NA
ERR768040	NA
ERR768041	NA
ERR768042	NA
ERR768043	NA
ERR768044	NA
ERR768045	NA
ERR768046	NA
ERR768047	NA
ERR768048	NA
ERR768049	NA
ERR768050	NA
ERR768051	NA
ERR772254	Argentina
ERR772255	Argentina
ERR772256	Argentina
ERR772257	Argentina
ERR772258	Argentina
ERR772259	Argentina
ERR772260	Argentina
ERR772261	Argentina
ERR772262	Argentina

ERR772263	Argentina
ERR772264	Argentina
ERR772265	Argentina
ERR773784	NA
ERR773785	NA
ERR773786	NA
ERR773787	NA
ERR773788	NA
ERR773789	NA
ERR773790	NA
ERR773791	NA
ERR773792	NA
ERR773793	NA
ERR773794	NA
ERR773795	NA
ERR773796	NA
ERR773797	NA
ERR773798	NA
ERR773799	NA
ERR773800	NA
ERR773801	NA
ERR773802	NA
ERR773803	NA
ERR773804	NA
ERR773805	NA
ERR773806	NA
ERR773807	NA
ERR773808	NA
ERR773809	NA
ERR773810	NA
ERR775293	Peru
ERR775294	Peru
ERR775295	Peru
ERR775296	Peru
ERR775297	Peru
ERR775298	Peru
ERR775299	Peru
ERR775300	Peru
ERR775301	Peru
ERR775302	Peru
ERR775303	Peru
ERR775304	Peru
ERR775305	Peru
ERR775306	Peru
ERR775307	Peru
ERR775308	Peru
ERR775309	Peru
ERR775310	Peru
ERR775311	Peru
ERR775312	Peru

ERR775313	Peru
ERR775314	Peru
ERR775315	Peru
ERR775316	Peru
ERR775317	Peru
ERR775318	Peru
ERR775319	Peru
ERR775320	Peru
ERR775321	Peru
ERR775322	Peru
ERR775323	Peru
ERR775324	Peru
ERR775325	Peru
ERR775326	Peru
ERR775327	Peru
ERR775328	Peru
ERR775329	Peru
ERR775330	Peru
ERR775331	Peru
ERR775332	Peru
ERR775333	Peru
ERR775334	Peru
ERR775335	Peru
ERR775336	Peru
ERR775337	Peru
ERR775338	Peru
ERR775339	Peru
ERR775340	Peru
ERR775341	Peru
ERR775342	Peru
ERR775343	Peru
ERR775344	Peru
ERR775345	Peru
ERR775346	Peru
ERR775347	Peru
ERR775348	Peru
ERR775349	Peru
ERR775350	Peru
ERR775351	Peru
ERR775352	Peru
ERR775353	Peru
ERR775354	Peru
ERR775355	Peru
ERR775356	Peru
ERR775357	Peru
ERR775358	Peru
ERR775359	Peru
ERR775360	Peru
ERR775361	Peru
ERR775362	Peru

ERR775363	Peru
ERR775364	Peru
ERR775365	Peru
ERR775366	Peru
ERR775367	Peru
ERR775368	Peru
ERR775369	Peru
ERR775370	Peru
ERR775371	Peru
ERR775372	Peru
ERR775373	Peru
ERR775374	Peru
ERR775375	Peru
ERR775376	Peru
ERR775377	Peru
ERR775378	Peru
ERR775379	Peru
ERR775380	Peru
ERR775381	Peru
ERR775382	Peru
ERR775383	Peru
ERR775384	Peru
ERR775385	Peru
ERR775386	Peru
ERR775387	Peru
ERR775388	Peru
ERR775389	Peru
ERR775753	NA
ERR775754	NA
ERR775755	NA
ERR775756	NA
ERR775757	NA
ERR775758	NA
ERR775759	NA
ERR775760	NA
ERR775761	NA
ERR775762	NA
ERR775763	NA
ERR775764	NA
ERR775765	NA
ERR775766	NA
ERR775767	NA
ERR775768	NA
ERR775769	NA
ERR775770	NA
ERR775771	NA
ERR775772	NA
ERR775773	NA
ERR775774	NA
ERR775775	NA

ERR775776	NA
ERR775777	NA
ERR775778	NA
ERR775779	NA
ERR775780	NA
ERR775781	NA
ERR775782	NA
ERR775783	NA
ERR775784	NA
ERR775785	NA
ERR775786	NA
ERR775787	NA
ERR775788	NA
ERR775789	NA
ERR775790	NA
ERR775791	NA
ERR775792	NA
ERR775793	NA
ERR775794	NA
ERR775795	NA
ERR775796	NA
ERR775797	NA
ERR775798	NA
ERR775799	NA
ERR775800	NA
ERR775801	NA
ERR775802	NA
ERR775803	NA
ERR775804	NA
ERR775805	NA
ERR775806	NA
ERR775807	NA
ERR776452	Argentina
ERR776453	Argentina
ERR776454	Argentina
ERR776455	Argentina
ERR776456	Argentina
ERR776457	Argentina
ERR776458	Argentina
ERR776459	Argentina
ERR776460	Argentina
ERR776461	Argentina
ERR776462	Argentina
ERR776463	Argentina
ERR776464	Argentina
ERR776465	Argentina
ERR776466	Argentina
ERR776467	Argentina
ERR776468	Argentina
ERR776469	Argentina

ERR776470	Argentina
ERR776471	Argentina
ERR776472	Argentina
ERR776473	Argentina
ERR776474	Argentina
ERR776475	Argentina
ERR776476	Argentina
ERR776659	Argentina
ERR776660	Argentina
ERR776661	Argentina
ERR776662	Argentina
ERR776663	Argentina
ERR776664	Argentina
ERR776665	Argentina
ERR776666	Argentina
ERR776667	Argentina
ERR776668	Argentina
ERR776669	Argentina
ERR776670	Argentina
ERR776671	Argentina
ERR776672	Argentina
ERR776673	Argentina
ERR776674	Argentina
ERR776675	Argentina
ERR779651	NA
ERR779652	NA
ERR779653	NA
ERR779654	NA
ERR779655	NA
ERR779656	NA
ERR779657	NA
ERR779658	NA
ERR779659	NA
ERR779660	NA
ERR779661	NA
ERR779662	NA
ERR779663	NA
ERR779664	NA
ERR779665	NA
ERR779666	NA
ERR779667	NA
ERR779668	NA
ERR779669	NA
ERR779670	NA
ERR779671	NA
ERR779672	NA
ERR779673	NA
ERR779674	NA
ERR779675	NA
ERR779676	NA

ERR779677	NA
ERR779678	NA
ERR779679	NA
ERR779680	NA
ERR779842	Peru
ERR779843	Peru
ERR779844	Peru
ERR779845	Peru
ERR779846	Peru
ERR779847	Peru
ERR779848	Peru
ERR779849	Peru
ERR779850	Peru
ERR779851	Peru
ERR779852	NA
ERR779853	Peru
ERR779854	Peru
ERR779855	Peru
ERR779856	Peru
ERR779857	Peru
ERR779858	Peru
ERR779859	Peru
ERR779860	Peru
ERR779861	Peru
ERR779862	Peru
ERR779863	Peru
ERR779864	Peru
ERR779865	Peru
ERR779866	Peru
ERR779867	Peru
ERR779868	Peru
ERR779869	Peru
ERR779870	Peru
ERR779871	Peru
ERR779872	Peru
ERR779873	Peru
ERR779874	Peru
ERR779875	Peru
ERR779876	Peru
ERR779877	Peru
ERR779878	Peru
ERR779879	Peru
ERR779880	Peru
ERR779881	Peru
ERR779882	Peru
ERR779883	Peru
ERR779884	Peru
ERR779885	Peru
ERR779886	Peru
ERR779887	Peru

ERR779888	Peru
ERR779889	Peru
ERR779890	Peru
ERR779891	Peru
ERR779892	Peru
ERR779893	Peru
ERR779894	Peru
ERR779895	Peru
ERR779896	Peru
ERR779897	Peru
ERR779898	Peru
ERR779899	Peru
ERR779900	Peru
ERR779901	Peru
ERR779902	Peru
ERR779903	Peru
ERR779904	Peru
ERR779905	Peru
ERR779906	Peru
ERR779907	Peru
ERR779908	Peru
ERR779909	Peru
ERR779910	Peru
ERR779911	Peru
ERR779912	Peru
ERR779913	Peru
ERR779914	Peru
ERR779915	Peru
ERR779916	Peru
ERR779917	Peru
ERR779918	Peru
ERR779919	Peru
ERR779920	Peru
ERR779921	Peru
ERR779922	Peru
ERR779923	Peru
ERR779924	Peru
ERR779925	Peru
ERR789234	Switzerland
ERR789235	Switzerland
ERR789236	Switzerland
ERR789237	Switzerland
ERR789238	Switzerland
ERR789239	Switzerland
ERR841490	NA
ERR841491	NA
ERR841492	NA
ERR841493	NA
ERR841494	NA
ERR841495	NA

ERR841496	NA
ERR845303	Argentina
ERR845304	Argentina
ERR845305	Argentina
ERR845306	Argentina
ERR845307	Argentina
ERR845327	Argentina
ERR845328	Argentina
ERR845329	Argentina
ERR845330	Argentina
ERR845932	NA
ERR845933	NA
ERR845934	Peru
ERR845935	Peru
ERR845936	Peru
ERR845937	Peru
ERR845938	NA
ERR845939	Peru
ERR845940	NA
ERR845941	Peru
ERR845942	NA
ERR846990	NA
ERR846991	NA
ERR846992	NA
ERR846993	NA
ERR846994	NA
ERR846995	NA
ERR846996	NA
ERR846997	NA
ERR846998	NA
ERR850078	Argentina
ERR850262	Argentina
ERR867518	United Kingdom
ERR867519	United Kingdom
ERR867520	United Kingdom
ERR867521	United Kingdom
ERR867522	United Kingdom
ERR867523	United Kingdom
ERR867524	United Kingdom
ERR867525	United Kingdom
ERR867526	United Kingdom
ERR867527	United Kingdom
ERR867528	United Kingdom
ERR867529	United Kingdom
ERR867530	United Kingdom
ERR867531	United Kingdom
ERR867532	United Kingdom
ERR867533	United Kingdom
ERR867534	United Kingdom
ERR867535	United Kingdom

ERR867536	United Kingdom
ERR867537	United Kingdom
ERR867538	United Kingdom
ERR867539	United Kingdom
ERR867540	United Kingdom
ERR867541	United Kingdom
ERR867542	United Kingdom
ERR867543	United Kingdom
ERR867544	United Kingdom
ERR867545	United Kingdom
ERR867546	United Kingdom
ERR867547	United Kingdom
ERR867548	United Kingdom
ERR867549	United Kingdom
ERR867550	United Kingdom
ERR867551	United Kingdom
ERR867552	United Kingdom
ERR867553	United Kingdom
ERR867554	United Kingdom
ERR867555	United Kingdom
ERR867556	United Kingdom
ERR867557	United Kingdom
ERR867558	United Kingdom
ERR867559	United Kingdom
ERR867560	United Kingdom
ERR867561	United Kingdom
ERR867562	United Kingdom
ERR867563	United Kingdom
ERR867564	United Kingdom
ERR867565	United Kingdom
ERR867566	United Kingdom
ERR867567	United Kingdom
ERR867568	United Kingdom
ERR867569	United Kingdom
ERR867570	United Kingdom
ERR867571	United Kingdom
ERR867572	United Kingdom
ERR867573	United Kingdom
ERR867574	United Kingdom
ERR867575	United Kingdom
ERR894411	NA
ERR894412	NA
ERR894413	NA
ERR894414	NA
ERR894415	NA
ERR894416	NA
ERR894417	NA
ERR894418	NA
ERR894419	NA
ERR894420	NA

ERR894421	NA
ERR894422	NA
ERR894423	NA
ERR894424	NA
ERR894425	NA
ERR894426	NA
ERR894427	NA
ERR894428	NA
ERR894429	NA
ERR894430	NA
ERR894431	NA
ERR894432	NA
ERR894433	NA
ERR894434	NA
ERR894435	NA
ERR894436	NA
ERR894437	NA
ERR894438	NA
ERR894439	NA
ERR894440	NA
ERR894441	NA
ERR894442	NA
ERR894443	NA
ERR894444	NA
ERR966619	South Africa
ERR966620	South Africa
ERR966621	South Africa
ERR970413	NA
ERR970414	NA
ERR970441	NA
ERR970442	NA
ERR970443	NA
ERR970444	NA
ERR970445	NA
ERR970446	NA
ERR970447	NA
ERR970448	NA
ERR970449	NA
ERR970450	NA
ERR970451	NA
ERR970452	NA
ERR970453	NA
ERR970454	NA
ERR970455	NA
ERR970456	NA
ERR970457	NA
ERR970458	NA
ERR970459	NA
ERR970460	NA
ERR970461	NA

ERR970462	NA
ERR970463	NA
ERR972795	NA
ERR972796	NA
ERR972797	NA
ERR972798	NA
ERR972799	NA
ERR972800	NA
ERR972801	NA
ERR972802	NA
ERR972803	NA
ERR972804	NA
ERR973451	Australia
ERR973452	Australia
ERR973453	Australia
ERR973454	Australia
ERR973455	Australia
ERR973456	Australia
ERR973457	Australia
ERR973458	Australia
ERR979062	Denmark
ERR979063	Denmark
ERR979064	Denmark
ERR979065	Denmark
ERR979066	Denmark
ERR979067	Denmark
ERR979068	Denmark
ERR979069	Denmark
ERR979070	Denmark
ERR979071	Denmark
ERR979072	Denmark
ERR979073	Denmark
ERR979074	Denmark
ERR979075	Denmark
ERR983228	Myanmar
ERR983229	Myanmar
ERR983230	Myanmar
ERR983231	Myanmar
ERR983232	Myanmar
ERR983233	Myanmar
ERR983234	Myanmar
ERR983235	Myanmar
ERR983236	Myanmar
ERR983237	Myanmar
ERR983238	Myanmar
ERR983239	Myanmar
ERR983240	Myanmar
ERR983241	Myanmar
ERR983250	NA
ERR983251	NA

ERR983252	NA
ERR983253	NA
ERR983254	NA
ERR983255	NA
ERR983256	NA
ERR985421	NA
ERR985422	NA
ERR985423	NA
ERR985424	NA
ERR985425	NA
ERR985426	NA
ERR987695	Uganda
ERR987696	Uganda
ERR987697	Uganda
ERR987698	Uganda
ERR987699	Uganda
ERR987700	Uganda
ERR987701	Uganda
ERR987702	Uganda
ERR987703	Uganda
ERR987704	Uganda
ERR987705	Uganda
ERR987706	Uganda
ERR987707	Uganda
ERR987708	Uganda
ERR987709	Uganda
ERR987710	Uganda
ERR987711	Uganda
ERR987712	Uganda
ERR987713	Uganda
ERR987714	Uganda
ERR987715	Uganda
ERR987716	Uganda
ERR987717	Uganda
ERR987718	Uganda
ERR987719	Uganda
ERR987720	Uganda
ERR987721	Uganda
ERR987722	Uganda
ERR987723	Uganda
ERR987724	Uganda
ERR987725	Uganda
ERR987726	Uganda
ERR987727	Uganda
ERR987728	Uganda
ERR987729	Uganda
ERR987730	Uganda
ERR987731	Uganda
ERR987732	Uganda
ERR987733	Uganda

ERR987734	Uganda
ERR987735	Uganda
ERR987736	Uganda
ERR987737	Uganda
ERR987738	Uganda
ERR987739	Uganda
ERR987740	Uganda
ERR987741	Uganda
ERR987742	Uganda
ERR987743	Uganda
ERR987744	Uganda
ERR987745	Uganda
ERR987746	Uganda
ERR987747	Uganda
ERR987748	Uganda
ERR987749	Uganda
ERR987750	Uganda
ERR987751	Uganda
ERR987752	Uganda
ERR987753	Uganda
ERR987754	Uganda
ERR987755	Uganda
ERR987756	Uganda
ERR987757	Uganda
ERR987758	Uganda
ERR987759	Uganda
ERR987760	Uganda
ERR987761	Uganda
ERR987762	Uganda
ERR987764	Uganda
ERR987765	Uganda
ERR987766	Uganda
ERR987767	Uganda
ERR987768	Uganda
ERR987769	Uganda
ERR987770	Uganda
ERR987771	Uganda
ERR987772	Uganda
ERR987774	Uganda
ERR987775	Uganda
ERR987778	Uganda
ERR987779	Uganda
ERR987780	Uganda
ERR987781	Uganda
ERR987783	Uganda
ERR987784	Uganda
ERR987785	Uganda
ERR987786	Uganda
ERR987787	Uganda
ERR987788	Uganda

ERR990531	NA
ERR990532	NA
ERR990533	NA
ERR990534	NA
ERR990535	NA
ERR990536	NA
ERR990537	NA
ERR990538	NA
ERR990539	NA
ERR990540	NA
ERR990541	NA
ERR990542	NA
ERR990543	NA
ERR990544	NA
ERR990545	NA
ERR990546	NA
ERR990547	NA
ERR990548	NA
ERR990549	NA
ERR990550	NA
ERR990551	NA
ERR990552	NA
ERR990553	NA
ERR990554	NA
ERR990555	NA
ERR990556	NA
SRR023449	NA
SRR023450	NA
SRR023451	NA
SRR023452	NA
SRR023453	NA
SRR023454	NA
SRR023455	NA
SRR023475	NA
SRR023476	NA
SRR023477	NA
SRR023478	NA
SRR023479	NA
SRR023480	NA
SRR023481	NA
SRR023482	NA
SRR023483	NA
SRR023484	NA
SRR023485	NA
SRR023486	NA
SRR023487	NA
SRR023492	NA
SRR023589	NA
SRR023590	NA
SRR026441	NA

SRR026442	NA
SRR026443	NA
SRR026444	NA
SRR026445	NA
SRR026446	NA
SRR029823	NA
SRR057510	NA
SRR057595	NA
SRR057610	NA
SRR057619	NA
SRR057659	NA
SRR057660	NA
SRR057725	NA
SRR057731	NA
SRR057732	NA
SRR057733	NA
SRR057734	NA
SRR057768	NA
SRR057770	NA
SRR057771	NA
SRR058081	NA
SRR058116	NA
SRR058117	NA
SRR058369	NA
SRR058370	NA
SRR058371	NA
SRR058372	NA
SRR058373	NA
SRR058374	NA
SRR058375	NA
SRR058376	NA
SRR058377	NA
SRR058378	NA
SRR058399	NA
SRR058400	NA
SRR058401	NA
SRR058402	NA
SRR058415	NA
SRR058417	NA
SRR058418	NA
SRR058419	NA
SRR058420	NA
SRR1186316	Canada
SRR1186993	Canada
SRR1187001	Canada
SRR1187011	Canada
SRR1187028	Canada
SRR1187037	Canada
SRR1187076	Canada
SRR1187082	Canada

SRR1187085	Canada
SRR1187086	Canada
SRR1187087	Canada
SRR1187088	Canada
SRR1187089	Canada
SRR1187181	Canada
SRR1187183	Canada
SRR1187184	Canada
SRR1187186	Canada
SRR1187192	Canada
SRR1187195	Canada
SRR1187196	Canada
SRR1187251	Canada
SRR1187252	Canada
SRR1187295	Canada
SRR1187296	Canada
SRR1187297	Canada
SRR1187380	Canada
SRR1187393	Canada
SRR1187423	Canada
SRR1187442	Canada
SRR1187576	Canada
SRR1187598	Canada
SRR1187618	Canada
SRR1187619	Canada
SRR1187620	Canada
SRR1187627	Canada
SRR1187628	Canada
SRR1187629	Canada
SRR1187630	Canada
SRR1187631	Canada
SRR1187633	Canada
SRR1187945	Canada
SRR1187946	Canada
SRR1187983	Canada
SRR1188082	Canada
SRR1188083	Canada
SRR1188084	Canada
SRR1188085	Canada
SRR1188086	Canada
SRR1188087	Canada
SRR1188119	Canada
SRR1188121	Canada
SRR1188127	Canada
SRR1188130	Canada
SRR1188131	Canada
SRR1188133	Canada
SRR1188137	Canada
SRR1188138	Canada
SRR1188143	Canada

SRR1188170	Canada
SRR1188174	Canada
SRR1188175	Canada
SRR1188181	Canada
SRR1188183	Canada
SRR1188184	Canada
SRR1188186	Canada
SRR1188188	Canada
SRR1188220	Canada
SRR1188259	Canada
SRR1188284	Canada
SRR1188286	Canada
SRR1188339	Canada
SRR1188341	Canada
SRR1188343	Canada
SRR1188359	Canada
SRR1188439	Canada
SRR1188459	Canada
SRR1188479	Canada
SRR1188486	Canada
SRR1188487	Canada
SRR1188492	Canada
SRR1188530	Canada
SRR1190432	Canada
SRR1190434	Canada
SRR1190442	Canada
SRR1190468	Canada
SRR1190470	Canada
SRR1190473	Canada
SRR1190474	Canada
SRR1190475	Canada
SRR1190477	Canada
SRR1190478	Canada
SRR1190480	Canada
SRR1191277	Canada
SRR1191298	Canada
SRR1191488	Canada
SRR1191489	Canada
SRR1191546	Canada
SRR1191547	Canada
SRR1191666	Canada
SRR1191727	Canada
SRR1200251	Canada
SRR1200797	Canada
SRR1200798	NA
SRR1200811	Canada
SRR1206023	NA
SRR1210498	NA
SRR1367196	China
SRR1367197	China

SRR1367216	China
SRR1367217	China
SRR1367219	China
SRR1367220	China
SRR1367221	China
SRR1367225	China
SRR1367226	China
SRR1368332	China
SRR1368333	China
SRR1368334	China
SRR1368335	China
SRR1368336	China
SRR1368337	China
SRR1368338	China
SRR1368339	China
SRR1368340	China
SRR1368341	China
SRR1510036	Canada
SRR1510037	Canada
SRR1510038	Canada
SRR1510039	Canada
SRR1510040	Canada
SRR1510041	Canada
SRR1510042	Canada
SRR1510043	Canada
SRR1510044	Canada
SRR1510045	Canada
SRR1510046	Canada
SRR1510047	Canada
SRR1510048	Canada
SRR1510049	Canada
SRR1510050	Canada
SRR1510051	Canada
SRR1510052	Canada
SRR1510053	Canada
SRR1510054	Canada
SRR1510055	Canada
SRR1510056	Canada
SRR1510057	Canada
SRR1510058	Canada
SRR1510059	Canada
SRR1510060	Canada
SRR1510061	Canada
SRR1510062	Canada
SRR1510063	Canada
SRR1510064	Canada
SRR1510065	Canada
SRR1510066	Canada
SRR1510067	Canada
SRR1510068	Canada

SRR1510069	Canada
SRR1510070	Canada
SRR1510071	Canada
SRR1510072	Canada
SRR1564305	Thailand
SRR1573680	Canada
SRR1573681	Canada
SRR1573682	Canada
SRR1573683	Canada
SRR1573684	Canada
SRR1573685	Canada
SRR1573686	Canada
SRR1573687	Canada
SRR1573688	Canada
SRR1573689	Canada
SRR1573690	Canada
SRR1573691	Canada
SRR1573692	Canada
SRR1573693	Canada
SRR1573694	Canada
SRR1573695	Canada
SRR1573696	Canada
SRR1573697	Canada
SRR1573698	Canada
SRR1573699	Canada
SRR1573700	Canada
SRR1573701	Canada
SRR1573702	Canada
SRR1573703	Canada
SRR1573704	Canada
SRR1573705	Canada
SRR1573706	Canada
SRR1573707	Canada
SRR1573708	Canada
SRR1573709	Canada
SRR1573710	Canada
SRR1573711	Canada
SRR1573712	Canada
SRR1573713	Canada
SRR1573714	Canada
SRR1573715	Canada
SRR1573716	Canada
SRR1573717	Canada
SRR1573718	Canada
SRR1573719	Canada
SRR1573720	Canada
SRR1573721	Canada
SRR1573722	Canada
SRR1573723	Canada
SRR1573724	Canada

SRR1573725	Canada
SRR1573726	Canada
SRR1573727	Canada
SRR1573728	Canada
SRR1573729	Canada
SRR1573730	Canada
SRR1573731	Canada
SRR1573732	Canada
SRR1573733	Canada
SRR1573734	Canada
SRR1577806	Mali
SRR1577808	Mali
SRR1577809	Mali
SRR1577810	Mali
SRR1577811	Mali
SRR1577821	Mali
SRR1577822	Mali
SRR1577823	Mali
SRR1577824	Mali
SRR1577825	Mali
SRR1577826	Mali
SRR1577827	Mali
SRR1577829	Mali
SRR1577830	Mali
SRR1577832	Mali
SRR1577835	Mali
SRR1577836	Mali
SRR1595970	Thailand
SRR1595971	Thailand
SRR1595972	Thailand
SRR1640242	Canada
SRR1640292	Canada
SRR1640293	Canada
SRR1640294	Canada
SRR1640295	Canada
SRR1640316	Canada
SRR1640321	Canada
SRR1640326	Canada
SRR1640327	Canada
SRR1640334	Canada
SRR1640335	Canada
SRR1640336	Canada
SRR1640338	Canada
SRR1640340	Canada
SRR1640342	Canada
SRR1640343	Canada
SRR1640345	Canada
SRR1640346	Canada
SRR1640347	Canada
SRR1640348	Canada

SRR1640349	Canada
SRR1640539	Canada
SRR1640540	Canada
SRR1640541	Canada
SRR1640542	Canada
SRR1640543	Canada
SRR1640547	Canada
SRR1640549	Canada
SRR1640551	Canada
SRR1640562	Canada
SRR1640563	Canada
SRR1640564	Canada
SRR1640565	Canada
SRR1640566	Canada
SRR1640567	Canada
SRR1640569	Canada
SRR1640570	Canada
SRR1640571	Canada
SRR1640574	Canada
SRR1640696	Canada
SRR1640697	Canada
SRR1640701	Canada
SRR1640706	Canada
SRR1640707	Canada
SRR1640708	Canada
SRR1640709	Canada
SRR1640710	Canada
SRR1640711	Canada
SRR1640712	Canada
SRR1640713	Canada
SRR1640714	Canada
SRR1640749	Canada
SRR1640750	Canada
SRR1640751	Canada
SRR1640752	Canada
SRR1649373	Switzerland
SRR1710057	China
SRR1710058	China
SRR1710059	China
SRR1710060	China
SRR1710061	China
SRR1710062	China
SRR1710063	China
SRR1710064	China
SRR1710065	China
SRR1710066	China
SRR1710067	China
SRR1710068	China
SRR1710069	China
SRR1710070	China

SRR1710071	China
SRR1710072	China
SRR1710073	China
SRR1710074	China
SRR1710075	China
SRR1710076	China
SRR1710077	China
SRR1710078	China
SRR1710079	China
SRR1710080	China
SRR1710081	China
SRR1710082	China
SRR1710083	China
SRR1710084	China
SRR1710085	China
SRR1710086	China
SRR1710087	China
SRR1710088	China
SRR1710089	China
SRR1710090	China
SRR1710091	China
SRR1710092	China
SRR1710093	China
SRR1710094	China
SRR1710095	China
SRR1710096	China
SRR1710097	China
SRR1710098	China
SRR1710099	China
SRR1710100	China
SRR1710101	China
SRR1710102	China
SRR1710103	China
SRR1710104	China
SRR1710105	China
SRR1710106	China
SRR1710107	China
SRR1710108	China
SRR1710109	China
SRR1710110	China
SRR1710111	China
SRR1710112	China
SRR1722712	Russia
SRR1722867	Russia
SRR1735570	United Kingdom
SRR1735571	United Kingdom
SRR1735573	United Kingdom
SRR1735581	United Kingdom
SRR1735582	United Kingdom
SRR1735585	United Kingdom

SRR1735586	United Kingdom
SRR1735587	United Kingdom
SRR1735588	United Kingdom
SRR1735591	United Kingdom
SRR1735596	United Kingdom
SRR1735605	United Kingdom
SRR1735610	United Kingdom
SRR1735611	United Kingdom
SRR1735615	United Kingdom
SRR1735624	United Kingdom
SRR1735634	United Kingdom
SRR1735640	United Kingdom
SRR1735713	United Kingdom
SRR1735716	United Kingdom
SRR1735717	United Kingdom
SRR1765871	Guatemala
SRR1765872	Guatemala
SRR1765874	Guatemala
SRR1765877	Guatemala
SRR1765879	Guatemala
SRR2010299	China
SRR2024879	China
SRR2024880	China
SRR2024881	China
SRR2024882	China
SRR2024883	China
SRR2024884	China
SRR2024885	China
SRR2024886	China
SRR2024887	China
SRR2024888	China
SRR2024889	China
SRR2024890	China
SRR2024891	China
SRR2024892	China
SRR2024893	China
SRR2024894	China
SRR2024895	China
SRR2024896	China
SRR2024897	China
SRR2024898	China
SRR2024899	China
SRR2024900	China
SRR2024901	China
SRR2024902	China
SRR2024903	China
SRR2024904	China
SRR2024905	China
SRR2024906	China
SRR2024907	China

SRR2024908	China
SRR2024909	China
SRR2024910	China
SRR2024911	China
SRR2024912	China
SRR2024913	China
SRR2024914	China
SRR2024915	China
SRR2024916	China
SRR2024917	China
SRR2024918	China
SRR2024919	China
SRR2024920	China
SRR2024921	China
SRR2024922	China
SRR2024923	China
SRR2024924	China
SRR2024925	China
SRR2024926	China
SRR2024927	China
SRR2024928	China
SRR2024929	China
SRR2024930	China
SRR2024931	China
SRR2024932	China
SRR2024933	China
SRR2024934	China
SRR2024935	China
SRR2024936	China
SRR2024937	China
SRR2024938	China
SRR2024939	China
SRR2024940	China
SRR2024941	China
SRR2024942	China
SRR2024943	China
SRR2024944	China
SRR2024945	China
SRR2024946	China
SRR2024947	China
SRR2024948	China
SRR2024949	China
SRR2024950	China
SRR2024951	China
SRR2024952	China
SRR2024953	China
SRR2024954	China
SRR2024955	China
SRR2024956	China
SRR2024957	China

SRR2024958	China
SRR2024959	China
SRR2024960	China
SRR2024961	China
SRR2024962	China
SRR2024963	China
SRR2024964	China
SRR2024965	China
SRR2024966	China
SRR2024967	China
SRR2024968	China
SRR2024969	China
SRR2024970	China
SRR2024971	China
SRR2024972	China
SRR2024973	China
SRR2024974	China
SRR2024975	China
SRR2024976	China
SRR2024977	China
SRR2024978	China
SRR2024979	China
SRR2024980	China
SRR2024981	China
SRR2024982	China
SRR2024983	China
SRR2024984	China
SRR2024985	China
SRR2024986	China
SRR2024987	China
SRR2024988	China
SRR2024989	China
SRR2024990	China
SRR2024991	China
SRR2024992	China
SRR2024993	China
SRR2024994	China
SRR2024995	China
SRR2024996	China
SRR2024997	China
SRR2024998	China
SRR2024999	China
SRR2025000	China
SRR2052578	Thailand
SRR2062305	China
SRR2063840	China
SRR2063842	China
SRR2063843	China
SRR2063844	China
SRR2075817	Korea

SRR2075818	Korea
SRR2075820	Korea
SRR2075822	Korea
SRR2075823	Korea
SRR2086486	Thailand
SRR2086487	Thailand
SRR2086488	Thailand
SRR2086489	Thailand
SRR2099924	NA
SRR2099925	NA
SRR2099926	NA
SRR2099927	NA
SRR2099928	NA
SRR2099929	NA
SRR2099930	NA
SRR2099931	NA
SRR2099932	NA
SRR2099933	NA
SRR2099934	NA
SRR2099935	NA
SRR2099936	NA
SRR2099937	NA
SRR2099938	NA
SRR2099939	NA
SRR2099940	NA
SRR2099941	NA
SRR2099942	NA
SRR2099943	NA
SRR2099944	NA
SRR2099945	NA
SRR2099946	NA
SRR2099947	NA
SRR2099948	NA
SRR2099949	NA
SRR2099950	NA
SRR2099951	NA
SRR2099952	NA
SRR2099953	NA
SRR2099954	NA
SRR2099955	NA
SRR2099956	NA
SRR2099957	NA
SRR2099958	NA
SRR2099959	NA
SRR2099960	NA
SRR2099961	NA
SRR2099962	NA
SRR2099963	NA
SRR2099964	NA
SRR2099965	NA

SRR2099966	NA
SRR2099967	NA
SRR2099968	NA
SRR2099969	NA
SRR2099970	NA
SRR2099971	NA
SRR2099972	NA
SRR2099973	NA
SRR2099974	NA
SRR2099975	NA
SRR2099976	NA
SRR2099977	NA
SRR2099978	NA
SRR2099979	NA
SRR2099980	NA
SRR2099981	NA
SRR2099982	NA
SRR2099983	NA
SRR2099984	NA
SRR2099985	NA
SRR2099986	NA
SRR2099987	NA
SRR2099988	NA
SRR2099989	NA
SRR2099990	NA
SRR2099991	NA
SRR2099992	NA
SRR2099993	NA
SRR2099994	NA
SRR2099995	NA
SRR2099996	NA
SRR2099997	NA
SRR2099998	NA
SRR2099999	NA
SRR2100000	NA
SRR2100001	NA
SRR2100002	NA
SRR2100003	NA
SRR2100004	NA
SRR2100005	NA
SRR2100006	NA
SRR2100007	NA
SRR2100008	NA
SRR2100009	NA
SRR2100010	NA
SRR2100011	NA
SRR2100012	NA
SRR2100013	NA
SRR2100014	NA
SRR2100015	NA

SRR2100016	NA
SRR2100017	NA
SRR2100018	NA
SRR2100019	NA
SRR2100020	NA
SRR2100021	NA
SRR2100022	NA
SRR2100023	NA
SRR2100024	NA
SRR2100025	NA
SRR2100026	NA
SRR2100027	NA
SRR2100028	NA
SRR2100029	NA
SRR2100030	NA
SRR2100031	NA
SRR2100032	NA
SRR2100033	NA
SRR2100034	NA
SRR2100035	NA
SRR2100036	NA
SRR2100037	NA
SRR2100038	NA
SRR2100039	NA
SRR2100040	NA
SRR2100041	NA
SRR2100042	NA
SRR2100043	NA
SRR2100044	NA
SRR2100045	NA
SRR2100046	NA
SRR2100047	NA
SRR2100048	NA
SRR2100049	NA
SRR2100050	NA
SRR2100051	NA
SRR2100052	NA
SRR2100053	NA
SRR2100054	NA
SRR2100055	NA
SRR2100056	NA
SRR2100057	NA
SRR2100058	NA
SRR2100059	NA
SRR2100060	NA
SRR2100061	NA
SRR2100062	NA
SRR2100063	NA
SRR2100064	NA
SRR2100065	NA

SRR2100066	NA
SRR2100067	NA
SRR2100068	NA
SRR2100069	NA
SRR2100070	NA
SRR2100071	NA
SRR2100072	NA
SRR2100073	NA
SRR2100074	NA
SRR2100075	NA
SRR2100076	NA
SRR2100077	NA
SRR2100078	NA
SRR2100079	NA
SRR2100080	NA
SRR2100081	NA
SRR2100082	NA
SRR2100083	NA
SRR2100084	NA
SRR2100085	NA
SRR2100086	NA
SRR2100087	NA
SRR2100088	NA
SRR2100089	NA
SRR2100090	NA
SRR2100091	NA
SRR2100092	NA
SRR2100093	NA
SRR2100094	NA
SRR2100095	NA
SRR2100096	NA
SRR2100097	NA
SRR2100098	NA
SRR2100099	NA
SRR2100100	NA
SRR2100101	NA
SRR2100102	NA
SRR2100103	NA
SRR2100104	NA
SRR2100105	NA
SRR2100106	NA
SRR2100107	NA
SRR2100108	NA
SRR2100109	NA
SRR2100110	NA
SRR2100111	NA
SRR2100112	NA
SRR2100113	NA
SRR2100114	NA
SRR2100115	NA

SRR2100116	NA
SRR2100117	NA
SRR2100118	NA
SRR2100119	NA
SRR2100120	NA
SRR2100121	NA
SRR2100122	NA
SRR2100123	NA
SRR2100124	NA
SRR2100125	NA
SRR2100126	NA
SRR2100127	NA
SRR2100128	NA
SRR2100129	NA
SRR2100130	NA
SRR2100131	NA
SRR2100132	NA
SRR2100133	NA
SRR2100134	NA
SRR2100135	NA
SRR2100136	NA
SRR2100137	NA
SRR2100138	NA
SRR2100139	NA
SRR2100140	NA
SRR2100141	NA
SRR2100142	NA
SRR2100143	NA
SRR2100144	NA
SRR2100145	NA
SRR2100146	NA
SRR2100147	NA
SRR2100148	NA
SRR2100149	NA
SRR2100150	NA
SRR2100151	NA
SRR2100152	NA
SRR2100153	NA
SRR2100154	NA
SRR2100155	NA
SRR2100156	NA
SRR2100157	NA
SRR2100158	NA
SRR2100159	NA
SRR2100160	NA
SRR2100161	NA
SRR2100162	NA
SRR2100163	NA
SRR2100164	NA
SRR2100165	NA

SRR2100166	NA
SRR2100167	NA
SRR2100168	NA
SRR2100169	NA
SRR2100170	NA
SRR2100171	NA
SRR2100172	NA
SRR2100173	NA
SRR2100174	NA
SRR2100175	NA
SRR2100176	NA
SRR2100177	NA
SRR2100178	NA
SRR2100179	NA
SRR2100180	NA
SRR2100181	NA
SRR2100182	NA
SRR2100183	NA
SRR2100184	NA
SRR2100185	NA
SRR2100186	NA
SRR2100187	NA
SRR2100188	NA
SRR2100189	NA
SRR2100190	NA
SRR2100191	NA
SRR2100192	NA
SRR2100193	NA
SRR2100194	NA
SRR2100195	NA
SRR2100196	NA
SRR2100197	NA
SRR2100198	NA
SRR2100199	NA
SRR2100200	NA
SRR2100201	NA
SRR2100202	NA
SRR2100203	NA
SRR2100204	NA
SRR2100205	NA
SRR2100206	NA
SRR2100207	NA
SRR2100208	NA
SRR2100209	NA
SRR2100210	NA
SRR2100211	NA
SRR2100212	NA
SRR2100213	NA
SRR2100214	NA
SRR2100215	NA

SRR2100216	NA
SRR2100217	NA
SRR2100218	NA
SRR2100219	NA
SRR2100220	NA
SRR2100221	NA
SRR2100222	NA
SRR2100223	NA
SRR2100224	NA
SRR2100225	NA
SRR2100226	NA
SRR2100227	NA
SRR2100228	NA
SRR2100229	NA
SRR2100230	NA
SRR2100231	NA
SRR2100232	NA
SRR2100233	NA
SRR2100234	NA
SRR2100235	NA
SRR2100236	NA
SRR2100237	NA
SRR2100238	NA
SRR2100239	NA
SRR2100240	NA
SRR2100241	NA
SRR2100242	NA
SRR2100243	NA
SRR2100244	NA
SRR2100245	NA
SRR2100246	NA
SRR2100247	NA
SRR2100248	NA
SRR2100249	NA
SRR2100250	NA
SRR2100251	NA
SRR2100252	NA
SRR2100253	NA
SRR2100254	NA
SRR2100255	NA
SRR2100256	NA
SRR2100257	NA
SRR2100258	NA
SRR2100259	NA
SRR2100260	NA
SRR2100261	NA
SRR2100262	NA
SRR2100263	NA
SRR2100264	NA
SRR2100265	NA

SRR2100266	NA
SRR2100267	NA
SRR2100268	NA
SRR2100269	NA
SRR2100270	NA
SRR2100271	NA
SRR2100272	NA
SRR2100273	NA
SRR2100274	NA
SRR2100275	NA
SRR2100276	NA
SRR2100277	NA
SRR2100278	NA
SRR2100279	NA
SRR2100280	NA
SRR2100281	NA
SRR2100282	NA
SRR2100283	NA
SRR2100284	NA
SRR2100285	NA
SRR2100286	NA
SRR2100287	NA
SRR2100288	NA
SRR2100289	NA
SRR2100290	NA
SRR2100291	NA
SRR2100292	NA
SRR2100293	NA
SRR2100294	NA
SRR2100295	NA
SRR2100296	NA
SRR2100297	NA
SRR2100298	NA
SRR2100299	NA
SRR2100300	NA
SRR2100301	NA
SRR2100302	NA
SRR2100303	NA
SRR2100304	NA
SRR2100305	NA
SRR2100306	NA
SRR2100307	NA
SRR2100308	NA
SRR2100309	NA
SRR2100310	NA
SRR2100311	NA
SRR2100312	NA
SRR2100313	NA
SRR2100314	NA
SRR2100315	NA

SRR2100316	NA
SRR2100317	NA
SRR2100318	NA
SRR2100319	NA
SRR2100320	NA
SRR2100321	NA
SRR2100322	NA
SRR2100323	NA
SRR2100324	NA
SRR2100325	NA
SRR2100326	NA
SRR2100327	NA
SRR2100328	NA
SRR2100329	NA
SRR2100330	NA
SRR2100331	NA
SRR2100332	NA
SRR2100333	NA
SRR2100334	NA
SRR2100335	NA
SRR2100336	NA
SRR2100337	NA
SRR2100338	NA
SRR2100339	NA
SRR2100340	NA
SRR2100341	NA
SRR2100342	NA
SRR2100343	NA
SRR2100344	NA
SRR2100345	NA
SRR2100346	NA
SRR2100347	NA
SRR2100348	NA
SRR2100349	NA
SRR2100350	NA
SRR2100351	NA
SRR2100352	NA
SRR2100353	NA
SRR2100354	NA
SRR2100355	NA
SRR2100356	NA
SRR2100357	NA
SRR2100358	NA
SRR2100359	NA
SRR2100360	NA
SRR2100361	NA
SRR2100362	NA
SRR2100363	NA
SRR2100364	NA
SRR2100365	NA

SRR2100366	NA
SRR2100367	NA
SRR2100368	NA
SRR2100369	NA
SRR2100370	NA
SRR2100371	NA
SRR2100372	NA
SRR2100373	NA
SRR2100374	NA
SRR2100375	NA
SRR2100376	NA
SRR2100377	NA
SRR2100378	NA
SRR2100379	NA
SRR2100380	NA
SRR2100381	NA
SRR2100382	NA
SRR2100383	NA
SRR2100384	NA
SRR2100385	NA
SRR2100386	NA
SRR2100387	NA
SRR2100388	NA
SRR2100389	NA
SRR2100390	NA
SRR2100391	NA
SRR2100392	NA
SRR2100393	NA
SRR2100394	NA
SRR2100395	NA
SRR2100396	NA
SRR2100397	NA
SRR2100398	NA
SRR2100399	NA
SRR2100400	NA
SRR2100401	NA
SRR2100402	NA
SRR2100403	NA
SRR2100404	NA
SRR2100405	NA
SRR2100406	NA
SRR2100407	NA
SRR2100408	NA
SRR2100409	NA
SRR2100410	NA
SRR2100411	NA
SRR2100412	NA
SRR2100413	NA
SRR2100414	NA
SRR2100415	NA

SRR2100416	NA
SRR2100417	NA
SRR2100418	NA
SRR2100419	NA
SRR2100420	NA
SRR2100421	NA
SRR2100422	NA
SRR2100423	NA
SRR2100424	NA
SRR2100425	NA
SRR2100426	NA
SRR2100427	NA
SRR2100428	NA
SRR2100429	NA
SRR2100430	NA
SRR2100431	NA
SRR2100432	NA
SRR2100433	NA
SRR2100434	NA
SRR2100435	NA
SRR2100436	NA
SRR2100437	NA
SRR2100438	NA
SRR2100439	NA
SRR2100440	NA
SRR2100441	NA
SRR2100442	NA
SRR2100443	NA
SRR2100444	NA
SRR2100445	NA
SRR2100446	NA
SRR2100447	NA
SRR2100448	NA
SRR2100449	NA
SRR2100450	NA
SRR2100451	NA
SRR2100452	NA
SRR2100453	NA
SRR2100454	NA
SRR2100455	NA
SRR2100456	NA
SRR2100457	NA
SRR2100458	NA
SRR2100459	NA
SRR2100460	NA
SRR2100461	NA
SRR2100462	NA
SRR2100463	NA
SRR2100464	NA
SRR2100465	NA

SRR2100466	NA
SRR2100467	NA
SRR2100468	NA
SRR2100469	NA
SRR2100470	NA
SRR2100471	NA
SRR2100472	NA
SRR2100473	NA
SRR2100474	NA
SRR2100475	NA
SRR2100476	NA
SRR2100477	NA
SRR2100478	NA
SRR2100479	NA
SRR2100480	NA
SRR2100481	NA
SRR2100482	NA
SRR2100483	NA
SRR2100484	NA
SRR2100485	NA
SRR2100486	NA
SRR2100487	NA
SRR2100488	NA
SRR2100489	NA
SRR2100490	NA
SRR2100491	NA
SRR2100492	NA
SRR2100493	NA
SRR2100494	NA
SRR2100495	NA
SRR2100496	NA
SRR2100497	NA
SRR2100498	NA
SRR2100499	NA
SRR2100500	NA
SRR2100501	NA
SRR2100502	NA
SRR2100503	NA
SRR2100504	NA
SRR2100505	NA
SRR2100506	NA
SRR2100507	NA
SRR2100508	NA
SRR2100509	NA
SRR2100510	NA
SRR2100511	NA
SRR2100512	NA
SRR2100513	NA
SRR2100514	NA
SRR2100515	NA

SRR2100516	NA
SRR2100517	NA
SRR2100518	NA
SRR2100519	NA
SRR2100520	NA
SRR2100521	NA
SRR2100522	NA
SRR2100523	NA
SRR2100524	NA
SRR2100525	NA
SRR2100526	NA
SRR2100527	NA
SRR2100528	NA
SRR2100529	NA
SRR2100530	NA
SRR2100531	NA
SRR2100532	NA
SRR2100533	NA
SRR2100534	NA
SRR2100535	NA
SRR2100536	NA
SRR2100537	NA
SRR2100538	NA
SRR2100539	NA
SRR2100540	NA
SRR2100541	NA
SRR2100542	NA
SRR2100543	NA
SRR2100544	NA
SRR2100545	NA
SRR2100546	NA
SRR2100547	NA
SRR2100548	NA
SRR2100549	NA
SRR2100550	NA
SRR2100551	NA
SRR2100552	NA
SRR2100553	NA
SRR2100554	NA
SRR2100555	NA
SRR2100556	NA
SRR2100557	NA
SRR2100558	NA
SRR2100559	NA
SRR2100560	NA
SRR2100561	NA
SRR2100562	NA
SRR2100563	NA
SRR2100564	NA
SRR2100565	NA

SRR2100566	NA
SRR2100567	NA
SRR2100568	NA
SRR2100569	NA
SRR2100570	NA
SRR2100571	NA
SRR2100572	NA
SRR2100573	NA
SRR2100574	NA
SRR2100575	NA
SRR2100576	NA
SRR2100577	NA
SRR2100578	NA
SRR2100579	NA
SRR2100580	NA
SRR2100581	NA
SRR2100582	NA
SRR2100583	NA
SRR2100584	NA
SRR2100585	NA
SRR2100586	NA
SRR2100587	NA
SRR2100588	NA
SRR2100589	NA
SRR2100590	NA
SRR2100591	NA
SRR2100592	NA
SRR2100593	NA
SRR2100594	NA
SRR2100595	NA
SRR2100596	NA
SRR2100597	NA
SRR2100598	NA
SRR2100599	NA
SRR2100600	NA
SRR2100601	NA
SRR2100602	NA
SRR2100603	NA
SRR2100604	NA
SRR2100605	NA
SRR2100606	NA
SRR2100607	NA
SRR2100608	NA
SRR2100609	NA
SRR2100610	NA
SRR2100611	NA
SRR2100612	NA
SRR2100613	NA
SRR2100614	NA
SRR2100615	NA

SRR2100616	NA
SRR2100617	NA
SRR2100618	NA
SRR2100619	NA
SRR2100620	NA
SRR2100621	NA
SRR2100622	NA
SRR2100623	NA
SRR2100624	NA
SRR2100625	NA
SRR2100626	NA
SRR2100627	NA
SRR2100628	NA
SRR2100629	NA
SRR2100630	NA
SRR2100631	NA
SRR2100632	NA
SRR2100633	NA
SRR2100634	NA
SRR2100635	NA
SRR2100636	NA
SRR2100637	NA
SRR2100638	NA
SRR2100639	NA
SRR2100640	NA
SRR2100641	NA
SRR2100642	NA
SRR2100643	NA
SRR2100644	NA
SRR2100645	NA
SRR2100646	NA
SRR2100647	NA
SRR2100648	NA
SRR2100649	NA
SRR2100650	NA
SRR2100651	NA
SRR2100652	NA
SRR2100653	NA
SRR2100654	NA
SRR2100655	NA
SRR2100656	NA
SRR2100657	NA
SRR2100658	NA
SRR2100659	NA
SRR2100660	NA
SRR2100661	NA
SRR2100662	NA
SRR2100663	NA
SRR2100664	NA
SRR2100665	NA

SRR2100666	NA
SRR2100667	NA
SRR2100668	NA
SRR2100669	NA
SRR2100670	NA
SRR2100671	NA
SRR2100672	NA
SRR2100673	NA
SRR2100674	NA
SRR2100675	NA
SRR2100676	NA
SRR2100677	NA
SRR2100678	NA
SRR2100679	NA
SRR2100680	NA
SRR2100681	NA
SRR2100682	NA
SRR2100683	NA
SRR2100684	NA
SRR2100685	NA
SRR2100686	NA
SRR2100687	NA
SRR2100688	NA
SRR2100689	NA
SRR2100690	NA
SRR2100691	NA
SRR2100692	NA
SRR2100693	NA
SRR2100694	NA
SRR2100695	NA
SRR2100696	NA
SRR2100697	NA
SRR2100698	NA
SRR2100699	NA
SRR2100700	NA
SRR2100701	NA
SRR2100702	NA
SRR2100703	NA
SRR2100704	NA
SRR2100705	NA
SRR2100706	NA
SRR2100707	NA
SRR2100708	NA
SRR2100709	NA
SRR2100710	NA
SRR2100711	NA
SRR2100712	NA
SRR2100713	NA
SRR2100714	NA
SRR2100715	NA

SRR2100716	NA
SRR2100717	NA
SRR2100718	NA
SRR2100719	NA
SRR2100720	NA
SRR2100721	NA
SRR2100722	NA
SRR2100723	NA
SRR2100724	NA
SRR2100725	NA
SRR2100726	NA
SRR2100727	NA
SRR2100728	NA
SRR2100729	NA
SRR2100730	NA
SRR2100731	NA
SRR2100732	NA
SRR2100733	NA
SRR2100734	NA
SRR2100735	NA
SRR2100736	NA
SRR2100737	NA
SRR2100738	NA
SRR2100739	NA
SRR2100740	NA
SRR2100741	NA
SRR2100742	NA
SRR2100743	NA
SRR2100744	NA
SRR2100745	NA
SRR2100746	NA
SRR2100747	NA
SRR2100748	NA
SRR2100749	NA
SRR2100750	NA
SRR2100751	NA
SRR2100752	NA
SRR2100753	NA
SRR2100754	NA
SRR2100755	NA
SRR2100756	NA
SRR2100757	NA
SRR2100758	NA
SRR2100759	NA
SRR2100760	NA
SRR2100761	NA
SRR2100762	NA
SRR2100763	NA
SRR2100764	NA
SRR2100765	NA

SRR2100766	NA
SRR2100767	NA
SRR2100768	NA
SRR2100769	NA
SRR2100770	NA
SRR2100771	NA
SRR2100772	NA
SRR2100773	NA
SRR2100774	NA
SRR2100775	NA
SRR2100776	NA
SRR2100777	NA
SRR2100778	NA
SRR2100779	NA
SRR2100780	NA
SRR2100781	NA
SRR2100782	NA
SRR2100783	NA
SRR2100784	NA
SRR2100785	NA
SRR2100786	NA
SRR2100787	NA
SRR2100788	NA
SRR2100789	NA
SRR2100790	NA
SRR2100791	NA
SRR2100792	NA
SRR2100793	NA
SRR2100794	NA
SRR2100795	NA
SRR2100796	NA
SRR2100797	NA
SRR2100798	NA
SRR2100799	NA
SRR2100800	NA
SRR2100801	NA
SRR2100802	NA
SRR2100803	NA
SRR2100804	NA
SRR2100805	NA
SRR2100806	NA
SRR2100807	NA
SRR2100808	NA
SRR2100809	NA
SRR2100810	NA
SRR2100811	NA
SRR2100812	NA
SRR2100813	NA
SRR2100814	NA
SRR2100815	NA

SRR2100816	NA
SRR2100817	NA
SRR2100818	NA
SRR2100819	NA
SRR2100820	NA
SRR2100821	NA
SRR2100822	NA
SRR2100823	NA
SRR2100824	NA
SRR2100825	NA
SRR2100826	NA
SRR2100827	NA
SRR2100828	NA
SRR2100829	NA
SRR2100830	NA
SRR2100831	NA
SRR2100832	NA
SRR2100833	NA
SRR2100834	NA
SRR2100835	NA
SRR2100836	NA
SRR2100837	NA
SRR2100838	NA
SRR2100839	NA
SRR2100840	NA
SRR2100841	NA
SRR2100842	NA
SRR2100843	NA
SRR2100844	NA
SRR2100845	NA
SRR2100846	NA
SRR2100847	NA
SRR2100848	NA
SRR2100849	NA
SRR2100850	NA
SRR2100851	NA
SRR2100852	NA
SRR2100853	NA
SRR2100854	NA
SRR2100855	NA
SRR2100856	NA
SRR2100857	NA
SRR2100858	NA
SRR2100859	NA
SRR2100860	NA
SRR2100861	NA
SRR2100862	NA
SRR2100863	NA
SRR2100864	NA
SRR2100865	NA

SRR2100866	NA
SRR2100867	NA
SRR2100868	NA
SRR2100869	NA
SRR2100870	NA
SRR2100871	NA
SRR2100872	NA
SRR2100873	NA
SRR2100874	NA
SRR2100875	NA
SRR2100876	NA
SRR2100877	NA
SRR2100878	NA
SRR2100879	NA
SRR2100880	NA
SRR2100881	NA
SRR2100882	NA
SRR2100883	NA
SRR2100884	NA
SRR2100885	NA
SRR2100886	NA
SRR2100887	NA
SRR2100888	NA
SRR2100889	NA
SRR2100890	NA
SRR2100891	NA
SRR2100892	NA
SRR2100893	NA
SRR2100894	NA
SRR2100895	NA
SRR2100896	NA
SRR2100897	NA
SRR2100898	NA
SRR2100899	NA
SRR2100900	NA
SRR2100901	NA
SRR2100902	NA
SRR2100903	NA
SRR2100904	NA
SRR2100905	NA
SRR2100906	NA
SRR2100907	NA
SRR2100908	NA
SRR2100909	NA
SRR2100910	NA
SRR2100911	NA
SRR2100912	NA
SRR2100913	NA
SRR2100914	NA
SRR2100915	NA

SRR2100916	NA
SRR2100917	NA
SRR2100918	NA
SRR2100919	NA
SRR2100920	NA
SRR2100921	NA
SRR2100922	NA
SRR2100923	NA
SRR2100924	NA
SRR2100925	NA
SRR2100926	NA
SRR2100927	NA
SRR2100928	NA
SRR2100929	NA
SRR2100930	NA
SRR2100931	NA
SRR2100932	NA
SRR2100933	NA
SRR2100934	NA
SRR2100935	NA
SRR2100936	NA
SRR2100937	NA
SRR2100938	NA
SRR2100939	NA
SRR2100940	NA
SRR2100941	NA
SRR2100942	NA
SRR2100943	NA
SRR2100944	NA
SRR2100945	NA
SRR2100946	NA
SRR2100947	NA
SRR2100948	NA
SRR2100949	NA
SRR2100950	NA
SRR2100951	NA
SRR2100952	NA
SRR2100953	NA
SRR2100954	NA
SRR2100955	NA
SRR2100956	NA
SRR2100957	NA
SRR2100958	NA
SRR2100959	NA
SRR2100960	NA
SRR2100961	NA
SRR2100962	NA
SRR2100963	NA
SRR2100964	NA
SRR2100965	NA

SRR2100966	NA
SRR2100967	NA
SRR2100968	NA
SRR2100969	NA
SRR2100970	NA
SRR2100971	NA
SRR2100972	NA
SRR2100973	NA
SRR2100974	NA
SRR2100975	NA
SRR2100976	NA
SRR2100977	NA
SRR2100978	NA
SRR2100979	NA
SRR2100980	NA
SRR2100981	NA
SRR2100982	NA
SRR2100983	NA
SRR2100984	NA
SRR2100985	NA
SRR2100986	NA
SRR2100987	NA
SRR2100988	NA
SRR2100989	NA
SRR2100990	NA
SRR2100991	NA
SRR2100992	NA
SRR2100993	NA
SRR2100994	NA
SRR2100995	NA
SRR2100996	NA
SRR2100997	NA
SRR2100998	NA
SRR2100999	NA
SRR2101000	NA
SRR2101001	NA
SRR2101002	NA
SRR2101003	NA
SRR2101004	NA
SRR2101005	NA
SRR2101006	NA
SRR2101007	NA
SRR2101008	NA
SRR2101009	NA
SRR2101010	NA
SRR2101011	NA
SRR2101012	NA
SRR2101013	NA
SRR2101014	NA
SRR2101015	NA

SRR2101016	NA
SRR2101017	NA
SRR2101018	NA
SRR2101019	NA
SRR2101020	NA
SRR2101021	NA
SRR2101022	NA
SRR2101023	NA
SRR2101024	NA
SRR2101025	NA
SRR2101026	NA
SRR2101027	NA
SRR2101028	NA
SRR2101029	NA
SRR2101030	NA
SRR2101031	NA
SRR2101032	NA
SRR2101033	NA
SRR2101034	NA
SRR2101035	NA
SRR2101036	NA
SRR2101037	NA
SRR2101038	NA
SRR2101039	NA
SRR2101040	NA
SRR2101041	NA
SRR2101042	NA
SRR2101043	NA
SRR2101044	NA
SRR2101045	NA
SRR2101046	NA
SRR2101047	NA
SRR2101048	NA
SRR2101049	NA
SRR2101050	NA
SRR2101051	NA
SRR2101052	NA
SRR2101053	NA
SRR2101054	NA
SRR2101055	NA
SRR2101056	NA
SRR2101057	NA
SRR2101058	NA
SRR2101059	NA
SRR2101060	NA
SRR2101061	NA
SRR2101062	NA
SRR2101063	NA
SRR2101064	NA
SRR2101065	NA

SRR2101066	NA
SRR2101067	NA
SRR2101068	NA
SRR2101069	NA
SRR2101070	NA
SRR2101071	NA
SRR2101072	NA
SRR2101073	NA
SRR2101074	NA
SRR2101075	NA
SRR2101076	NA
SRR2101077	NA
SRR2101078	NA
SRR2101079	NA
SRR2101080	NA
SRR2101081	NA
SRR2101082	NA
SRR2101083	NA
SRR2101084	NA
SRR2101085	NA
SRR2101086	NA
SRR2101087	NA
SRR2101088	NA
SRR2101089	NA
SRR2101090	NA
SRR2101091	NA
SRR2101092	NA
SRR2101093	NA
SRR2101094	NA
SRR2101095	NA
SRR2101096	NA
SRR2101097	NA
SRR2101098	NA
SRR2101099	NA
SRR2101100	NA
SRR2101101	NA
SRR2101102	NA
SRR2101103	NA
SRR2101104	NA
SRR2101105	NA
SRR2101106	NA
SRR2101107	NA
SRR2101108	NA
SRR2101109	NA
SRR2101110	NA
SRR2101111	NA
SRR2101112	NA
SRR2101113	NA
SRR2101114	NA
SRR2101115	NA

SRR2101116	NA
SRR2101117	NA
SRR2101118	NA
SRR2101119	NA
SRR2101120	NA
SRR2101121	NA
SRR2101122	NA
SRR2101123	NA
SRR2101124	NA
SRR2101125	NA
SRR2101126	NA
SRR2101127	NA
SRR2101128	NA
SRR2101129	NA
SRR2101130	NA
SRR2101131	NA
SRR2101132	NA
SRR2101133	NA
SRR2101134	NA
SRR2101135	NA
SRR2101136	NA
SRR2101137	NA
SRR2101138	NA
SRR2101139	NA
SRR2101140	NA
SRR2101141	NA
SRR2101142	NA
SRR2101143	NA
SRR2101144	NA
SRR2101145	NA
SRR2101146	NA
SRR2101147	NA
SRR2101148	NA
SRR2101149	NA
SRR2101150	NA
SRR2101151	NA
SRR2101152	NA
SRR2101153	NA
SRR2101154	NA
SRR2101155	NA
SRR2101156	NA
SRR2101157	NA
SRR2101158	NA
SRR2101159	NA
SRR2101160	NA
SRR2101161	NA
SRR2101162	NA
SRR2101163	NA
SRR2101164	NA
SRR2101165	NA

SRR2101166	NA
SRR2101167	NA
SRR2101168	NA
SRR2101169	NA
SRR2101170	NA
SRR2101171	NA
SRR2101172	NA
SRR2101173	NA
SRR2101174	NA
SRR2101175	NA
SRR2101176	NA
SRR2101177	NA
SRR2101178	NA
SRR2101179	NA
SRR2101180	NA
SRR2101181	NA
SRR2101182	NA
SRR2101183	NA
SRR2101184	NA
SRR2101185	NA
SRR2101186	NA
SRR2101187	NA
SRR2101188	NA
SRR2101189	NA
SRR2101190	NA
SRR2101191	NA
SRR2101192	NA
SRR2101193	NA
SRR2101194	NA
SRR2101195	NA
SRR2101196	NA
SRR2101197	NA
SRR2101198	NA
SRR2101199	NA
SRR2101200	NA
SRR2101201	NA
SRR2101202	NA
SRR2101203	NA
SRR2101204	NA
SRR2101205	NA
SRR2101206	NA
SRR2101207	NA
SRR2101208	NA
SRR2101209	NA
SRR2101210	NA
SRR2101211	NA
SRR2101212	NA
SRR2101213	NA
SRR2101214	NA
SRR2101215	NA

SRR2101216	NA
SRR2101217	NA
SRR2101218	NA
SRR2101219	NA
SRR2101220	NA
SRR2101221	NA
SRR2101222	NA
SRR2101223	NA
SRR2101224	NA
SRR2101225	NA
SRR2101226	NA
SRR2101227	NA
SRR2101228	NA
SRR2101229	NA
SRR2101230	NA
SRR2101231	NA
SRR2101232	NA
SRR2101233	NA
SRR2101234	NA
SRR2101235	NA
SRR2101236	NA
SRR2101237	NA
SRR2101238	NA
SRR2101239	NA
SRR2101240	NA
SRR2101241	NA
SRR2101242	NA
SRR2101243	NA
SRR2101244	NA
SRR2101245	NA
SRR2101246	NA
SRR2101247	NA
SRR2101248	NA
SRR2101249	NA
SRR2101250	NA
SRR2101251	NA
SRR2101252	NA
SRR2101253	NA
SRR2101254	NA
SRR2101255	NA
SRR2101256	NA
SRR2101257	NA
SRR2101258	NA
SRR2101259	NA
SRR2101260	NA
SRR2101261	NA
SRR2101262	NA
SRR2101263	NA
SRR2101264	NA
SRR2101265	NA

SRR2101266	NA
SRR2101267	NA
SRR2101268	NA
SRR2101269	NA
SRR2101270	NA
SRR2101271	NA
SRR2101272	NA
SRR2101273	NA
SRR2101274	NA
SRR2101275	NA
SRR2101276	NA
SRR2101277	NA
SRR2101278	NA
SRR2101279	NA
SRR2101280	NA
SRR2101281	NA
SRR2101282	NA
SRR2101283	NA
SRR2101284	NA
SRR2101285	NA
SRR2101286	NA
SRR2101287	NA
SRR2101288	NA
SRR2101289	NA
SRR2101290	NA
SRR2101291	NA
SRR2101292	NA
SRR2101293	NA
SRR2101294	NA
SRR2101295	NA
SRR2101296	NA
SRR2101297	NA
SRR2101298	NA
SRR2101299	NA
SRR2101300	NA
SRR2101301	NA
SRR2101302	NA
SRR2101303	NA
SRR2101304	NA
SRR2101305	NA
SRR2101306	NA
SRR2101307	NA
SRR2101308	NA
SRR2101309	NA
SRR2101310	NA
SRR2101311	NA
SRR2101312	NA
SRR2101313	NA
SRR2101314	NA
SRR2101315	NA

SRR2101316	NA
SRR2101317	NA
SRR2101318	NA
SRR2101319	NA
SRR2101320	NA
SRR2101321	NA
SRR2101322	NA
SRR2101323	NA
SRR2101324	NA
SRR2101325	NA
SRR2101326	NA
SRR2101327	NA
SRR2101328	NA
SRR2101329	NA
SRR2101330	NA
SRR2101331	NA
SRR2101332	NA
SRR2101333	NA
SRR2101334	NA
SRR2101335	NA
SRR2101336	NA
SRR2101337	NA
SRR2101338	NA
SRR2101339	NA
SRR2101340	NA
SRR2101341	NA
SRR2101342	NA
SRR2101343	NA
SRR2101344	NA
SRR2101345	NA
SRR2101346	NA
SRR2101347	NA
SRR2101348	NA
SRR2101349	NA
SRR2101350	NA
SRR2101351	NA
SRR2101352	NA
SRR2101353	NA
SRR2101354	NA
SRR2101355	NA
SRR2101356	NA
SRR2101357	NA
SRR2101358	NA
SRR2101359	NA
SRR2101360	NA
SRR2101361	NA
SRR2101362	NA
SRR2101363	NA
SRR2101364	NA
SRR2101365	NA

SRR2101366	NA
SRR2101367	NA
SRR2101368	NA
SRR2101369	NA
SRR2101370	NA
SRR2101371	NA
SRR2101372	NA
SRR2101373	NA
SRR2101374	NA
SRR2101375	NA
SRR2101376	NA
SRR2101377	NA
SRR2101378	NA
SRR2101379	NA
SRR2101380	NA
SRR2101381	NA
SRR2101382	NA
SRR2101383	NA
SRR2101384	NA
SRR2101385	NA
SRR2101386	NA
SRR2101387	NA
SRR2101388	NA
SRR2101389	NA
SRR2101390	NA
SRR2101391	NA
SRR2101392	NA
SRR2101393	NA
SRR2101394	NA
SRR2101395	NA
SRR2101396	NA
SRR2101397	NA
SRR2101398	NA
SRR2101399	NA
SRR2101400	NA
SRR2101401	NA
SRR2101402	NA
SRR2101403	NA
SRR2101404	NA
SRR2101405	NA
SRR2101406	NA
SRR2101407	NA
SRR2101408	NA
SRR2101409	NA
SRR2101410	NA
SRR2101411	NA
SRR2101412	NA
SRR2101413	NA
SRR2101414	NA
SRR2101415	NA

SRR2101416	NA
SRR2101417	NA
SRR2101418	NA
SRR2101419	NA
SRR2101420	NA
SRR2101421	NA
SRR2101422	NA
SRR2101423	NA
SRR2101424	NA
SRR2101425	NA
SRR2101426	NA
SRR2101427	NA
SRR2101428	NA
SRR2101429	NA
SRR2101430	NA
SRR2101431	NA
SRR2101432	NA
SRR2101433	NA
SRR2101434	NA
SRR2101435	NA
SRR2101436	NA
SRR2101437	NA
SRR2101438	NA
SRR2101439	NA
SRR2101440	NA
SRR2101441	NA
SRR2101442	NA
SRR2101443	NA
SRR2101444	NA
SRR2101445	NA
SRR2101446	NA
SRR2101447	NA
SRR2101448	NA
SRR2101449	NA
SRR2101450	NA
SRR2101451	NA
SRR2101452	NA
SRR2101453	NA
SRR2101454	NA
SRR2101455	NA
SRR2101456	NA
SRR2101457	NA
SRR2101458	NA
SRR2101459	NA
SRR2101460	NA
SRR2101461	NA
SRR2101462	NA
SRR2101463	NA
SRR2101464	NA
SRR2101465	NA

SRR2101466	NA
SRR2101467	NA
SRR2101468	NA
SRR2101469	NA
SRR2101470	NA
SRR2101471	NA
SRR2101472	NA
SRR2101473	NA
SRR2101474	NA
SRR2101475	NA
SRR2101476	NA
SRR2101477	NA
SRR2101478	NA
SRR2101479	NA
SRR2101480	NA
SRR2101481	NA
SRR2101482	NA
SRR2101483	NA
SRR2101484	NA
SRR2101485	NA
SRR2101486	NA
SRR2101487	NA
SRR2101488	NA
SRR2101489	NA
SRR2101490	NA
SRR2101491	NA
SRR2101492	NA
SRR2101493	NA
SRR2101494	NA
SRR2101495	NA
SRR2101496	NA
SRR2101497	NA
SRR2101498	NA
SRR2101499	NA
SRR2101500	NA
SRR2101501	NA
SRR2101502	NA
SRR2101503	NA
SRR2101504	NA
SRR2101505	NA
SRR2101506	NA
SRR2101507	NA
SRR2101508	NA
SRR2101509	NA
SRR2101510	NA
SRR2101511	NA
SRR2101512	NA
SRR2101513	NA
SRR2101514	NA
SRR2101515	NA

SRR2101516	NA
SRR2101517	NA
SRR2101518	NA
SRR2101519	NA
SRR2101520	NA
SRR2101521	NA
SRR2101522	NA
SRR2101523	NA
SRR2101524	NA
SRR2101525	NA
SRR2101526	NA
SRR2101527	NA
SRR2101528	NA
SRR2101529	NA
SRR2101530	NA
SRR2101531	NA
SRR2101532	NA
SRR2101533	NA
SRR2101534	NA
SRR2101535	NA
SRR2101536	NA
SRR2101537	NA
SRR2101538	NA
SRR2101539	NA
SRR2101540	NA
SRR2101541	NA
SRR2101542	NA
SRR2101543	NA
SRR2101544	NA
SRR2101545	NA
SRR2101546	NA
SRR2101547	NA
SRR2101548	NA
SRR2101549	NA
SRR2101550	NA
SRR2101551	NA
SRR2101552	NA
SRR2101553	NA
SRR2101554	NA
SRR2101555	NA
SRR2101556	NA
SRR2101557	NA
SRR2101558	NA
SRR2101559	NA
SRR2101560	NA
SRR2101561	NA
SRR2101562	NA
SRR2101563	NA
SRR2101564	NA
SRR2101565	NA

SRR2101566	NA
SRR2101567	NA
SRR2101568	NA
SRR2101569	NA
SRR2101570	NA
SRR2101571	NA
SRR2101572	NA
SRR2101573	NA
SRR2101574	NA
SRR2101575	NA
SRR2101576	NA
SRR2101577	NA
SRR2101578	NA
SRR2101579	NA
SRR2101580	NA
SRR2101581	NA
SRR2101582	NA
SRR2101583	NA
SRR2101584	NA
SRR2101585	NA
SRR2101586	NA
SRR2101587	NA
SRR2101588	NA
SRR2101589	NA
SRR2101590	NA
SRR2101591	NA
SRR2101592	NA
SRR2101593	NA
SRR2101594	NA
SRR2101595	NA
SRR2101596	NA
SRR2101597	NA
SRR2101598	NA
SRR2101599	NA
SRR2101600	NA
SRR2101601	NA
SRR2101602	NA
SRR2101603	NA
SRR2101604	NA
SRR2101605	NA
SRR2101606	NA
SRR2101607	NA
SRR2101608	NA
SRR2101609	NA
SRR2101610	NA
SRR2101611	NA
SRR2101612	NA
SRR2101613	NA
SRR2101614	NA
SRR2101615	NA

SRR2101616	NA
SRR2101617	NA
SRR2101618	NA
SRR2101619	NA
SRR2101620	NA
SRR2101621	NA
SRR2101622	NA
SRR2101623	NA
SRR2101624	NA
SRR2101625	NA
SRR2101626	NA
SRR2101627	NA
SRR2101628	NA
SRR2101629	NA
SRR2101630	NA
SRR2101631	NA
SRR2101632	NA
SRR2101633	NA
SRR2101634	NA
SRR2101635	NA
SRR2101636	NA
SRR2101637	NA
SRR2101638	NA
SRR2101639	NA
SRR2101640	NA
SRR2101641	NA
SRR2101642	NA
SRR2101643	NA
SRR2101644	NA
SRR2101645	NA
SRR2101646	NA
SRR2101647	NA
SRR2101648	NA
SRR2101649	NA
SRR2101650	NA
SRR2101651	NA
SRR2101652	NA
SRR2101653	NA
SRR2101654	NA
SRR2101655	NA
SRR2101656	NA
SRR2101657	NA
SRR2101658	NA
SRR2101659	NA
SRR2101660	NA
SRR2101661	NA
SRR2101662	NA
SRR2101663	NA
SRR2101664	NA
SRR2101665	NA

SRR2101666	NA
SRR2101667	NA
SRR2101668	NA
SRR2101669	NA
SRR2101670	NA
SRR2101671	NA
SRR2101672	NA
SRR2101673	NA
SRR2101674	NA
SRR2101675	NA
SRR2101676	NA
SRR2101677	NA
SRR2101678	NA
SRR2101679	NA
SRR2101680	NA
SRR2101681	NA
SRR2101682	NA
SRR2101683	NA
SRR2101684	NA
SRR2101685	NA
SRR2101686	NA
SRR2101687	NA
SRR2101688	NA
SRR2101689	NA
SRR2101690	NA
SRR2101691	NA
SRR2101692	NA
SRR2101693	NA
SRR2101694	NA
SRR2101695	NA
SRR2101696	NA
SRR2101697	NA
SRR2101698	NA
SRR2101699	NA
SRR2101700	NA
SRR2101701	NA
SRR2101702	NA
SRR2101703	NA
SRR2101704	NA
SRR2101705	NA
SRR2101706	NA
SRR2101707	NA
SRR2101708	NA
SRR2101709	NA
SRR2101710	NA
SRR2101711	NA
SRR2101712	NA
SRR2101713	NA
SRR2101714	NA
SRR2101715	NA

SRR2101716	NA
SRR2101717	NA
SRR2101718	NA
SRR2101719	NA
SRR2101720	NA
SRR2101721	NA
SRR2101722	NA
SRR2101723	NA
SRR2101724	NA
SRR2101725	NA
SRR2101726	NA
SRR2101727	NA
SRR2101728	NA
SRR2101729	NA
SRR2101730	NA
SRR2101731	NA
SRR2101732	NA
SRR2101733	NA
SRR2101734	NA
SRR2101735	NA
SRR2101736	NA
SRR2101737	NA
SRR2101738	NA
SRR2101739	NA
SRR2101740	NA
SRR2101741	NA
SRR2101742	NA
SRR2101743	NA
SRR2101744	NA
SRR2101745	NA
SRR2101746	NA
SRR2101747	NA
SRR2101748	NA
SRR2101749	NA
SRR2101750	NA
SRR2101751	NA
SRR2101752	NA
SRR2101753	NA
SRR2101754	NA
SRR2101755	NA
SRR2101756	NA
SRR2101757	NA
SRR2101758	NA
SRR2101759	NA
SRR2101760	NA
SRR2101761	NA
SRR2101762	NA
SRR2101763	NA
SRR2101764	NA
SRR2101765	NA

SRR2101766	NA
SRR2101767	NA
SRR2101768	NA
SRR2101769	NA
SRR2101770	NA
SRR2101771	NA
SRR2101772	NA
SRR2101773	NA
SRR2101774	NA
SRR2101775	NA
SRR2101776	NA
SRR2101777	NA
SRR2101778	NA
SRR2101779	NA
SRR2101780	NA
SRR2101781	NA
SRR2101782	NA
SRR2101783	NA
SRR2101784	NA
SRR2101785	NA
SRR2101786	NA
SRR2101787	NA
SRR2101788	NA
SRR2101789	NA
SRR2101790	NA
SRR2101791	NA
SRR2101792	NA
SRR2101793	NA
SRR2101794	NA
SRR2101795	NA
SRR2101796	NA
SRR2101797	NA
SRR2101798	NA
SRR2101799	NA
SRR2101800	NA
SRR2101801	NA
SRR2101802	NA
SRR2101803	NA
SRR2101804	NA
SRR2101805	NA
SRR2101806	NA
SRR2101807	NA
SRR2101808	NA
SRR2101809	NA
SRR2101810	NA
SRR2101811	NA
SRR2101812	NA
SRR2101813	NA
SRR2101814	NA
SRR2101815	NA

SRR2101816	NA
SRR2101817	NA
SRR2101818	NA
SRR2101819	NA
SRR2101820	NA
SRR2101821	NA
SRR2101822	NA
SRR2101823	NA
SRR2101824	NA
SRR2101825	NA
SRR2101826	NA
SRR2101827	NA
SRR2101828	NA
SRR2120751	India
SRR2120772	India
SRR2328057	Australia
SRR2333215	Australia
SRR2467264	NA
SRR2467265	NA
SRR2467266	NA
SRR2467267	NA
SRR2467268	NA
SRR2467269	NA
SRR2467270	NA
SRR2467271	NA
SRR2469365	NA
SRR2469366	NA
SRR2469368	NA
SRR2469370	NA
SRR2469371	NA
SRR2469373	NA
SRR2469374	NA
SRR2469376	NA
SRR2469377	NA
SRR2469378	NA
SRR2469380	NA
SRR2502770	Canada
SRR2502771	Canada
SRR2502772	Canada
SRR2502773	Canada
SRR2502774	Canada
SRR2502775	Canada
SRR2502776	Canada
SRR2502777	Canada
SRR2502778	Canada
SRR2502779	Canada
SRR2502780	Canada
SRR2502781	Canada
SRR2502782	Canada
SRR2502783	Canada

SRR2502784	Canada
SRR2502785	Canada
SRR2502786	Canada
SRR2502787	Canada
SRR2502788	Canada
SRR2502789	Canada
SRR2502790	Canada
SRR2502791	Canada
SRR2502792	Canada
SRR2502793	Canada
SRR2502794	Canada
SRR2502795	Canada
SRR2502796	Canada
SRR2502797	Canada
SRR2502798	Canada
SRR2502799	Canada
SRR2502800	Canada
SRR2502801	Canada
SRR2502802	Canada
SRR2502803	Canada
SRR2502804	Canada
SRR2502805	Canada
SRR2502806	Canada
SRR2502807	Canada
SRR2502808	Canada
SRR2502809	Canada
SRR2502810	Canada
SRR2502811	Canada
SRR2502812	Canada
SRR2502813	Canada
SRR2502814	Canada
SRR2502815	Canada
SRR2502816	Canada
SRR2502817	Canada
SRR2502818	Canada
SRR2502819	Canada
SRR2502820	Canada
SRR2502821	Canada
SRR2502822	Canada
SRR2502823	Canada
SRR2502824	Canada
SRR2502825	Canada
SRR2502826	Canada
SRR2502827	Canada
SRR2502828	Canada
SRR2502829	Canada
SRR2502830	Canada
SRR2502831	Canada
SRR2502832	Canada
SRR2502833	Canada

SRR2502834	Canada
SRR2502835	Canada
SRR2502836	Canada
SRR2502837	Canada
SRR2502838	Canada
SRR2502839	Canada
SRR2502840	Canada
SRR2502841	Canada
SRR2502842	Canada
SRR2502843	Canada
SRR2502844	Canada
SRR2502845	Canada
SRR2502846	Canada
SRR2502847	Canada
SRR2502848	Canada
SRR2502849	Canada
SRR2502850	Canada
SRR2502851	Canada
SRR2502852	Canada
SRR2502853	Canada
SRR2502854	Canada
SRR2502855	Canada
SRR2502856	Canada
SRR2502857	Canada
SRR2502858	Canada
SRR2502859	Canada
SRR2502860	Canada
SRR2502861	Canada
SRR2502862	Canada
SRR2502863	Canada
SRR2502864	Canada
SRR2502865	Canada
SRR2502866	Canada
SRR2502867	Canada
SRR2993037	Russia
SRR2993039	Russia
SRR3055239	NA
SRR3055240	NA
SRR3055241	NA
SRR3055242	NA
SRR3055243	NA
SRR3055244	NA
SRR3055245	NA
SRR3055246	NA
SRR3055247	NA
SRR3055248	NA
SRR3055249	NA
SRR3055250	NA
SRR3055251	NA
SRR3055252	NA

SRR3055711	South Africa
SRR3055712	South Africa
SRR3055713	South Africa
SRR3055714	South Africa
SRR3055715	South Africa
SRR3055716	South Africa
SRR3055717	South Africa
SRR3055718	South Africa
SRR3055719	South Africa
SRR3055720	South Africa
SRR3082066	NA
SRR3082067	Albania
SRR3082068	Albania
SRR3082069	Albania
SRR3082070	Tajikistan
SRR3082071	Tajikistan
SRR3082072	Djibouti
SRR3082073	Djibouti
SRR3082074	Djibouti
SRR3082075	Djibouti
SRR3082076	Djibouti
SRR3082077	Djibouti
SRR3082078	Albania
SRR3082079	Djibouti
SRR3082080	Djibouti
SRR3082081	Djibouti
SRR3082082	Djibouti
SRR3082083	Djibouti
SRR3082084	Djibouti
SRR3082085	Djibouti
SRR3082086	Djibouti
SRR3082087	Djibouti
SRR3082088	Djibouti
SRR3082089	Albania
SRR3082090	Djibouti
SRR3082091	Djibouti
SRR3082092	Djibouti
SRR3082093	Djibouti
SRR3082094	Djibouti
SRR3082095	Tajikistan
SRR3082096	Italy
SRR3082097	Italy
SRR3082098	Italy
SRR3082099	Tunisia
SRR3082100	Albania
SRR3082101	Tunisia
SRR3082102	Tajikistan
SRR3082103	Tajikistan
SRR3082104	Tajikistan
SRR3082105	Tajikistan

SRR3082106	Tajikistan
SRR3082107	Tunisia
SRR3082108	Tajikistan
SRR3082109	Tajikistan
SRR3082110	Italy
SRR3082111	Albania
SRR3082112	Italy
SRR3082113	Italy
SRR3082114	Italy
SRR3082115	Tajikistan
SRR3082116	Tajikistan
SRR3082117	Tajikistan
SRR3082118	Tajikistan
SRR3082119	Italy
SRR3082120	Italy
SRR3082121	Italy
SRR3082122	Albania
SRR3082123	Italy
SRR3082124	Italy
SRR3082125	Tajikistan
SRR3082126	Tajikistan
SRR3082127	Tajikistan
SRR3082128	Tajikistan
SRR3082129	Tajikistan
SRR3082130	Tajikistan
SRR3082131	Albania
SRR3082132	Albania
SRR3082133	Albania
SRR3082134	Tunisia
SRR3082135	Tunisia
SRR3082136	Albania
SRR3082137	Djibouti
SRR3082138	Italy
SRR3082139	Tunisia
SRR3082140	Italy
SRR3082141	Tunisia
SRR3082142	Tunisia
SRR3082143	Albania
SRR3082144	Albania
SRR3085246	Tajikistan
SRR3085247	Tajikistan
SRR3085248	Italy
SRR3085249	Italy
SRR3085250	Italy
SRR3085251	Italy
SRR3085252	Italy
SRR3085253	Italy
SRR3085254	Italy
SRR3085255	Tajikistan
SRR3085256	Tajikistan

SRR3085257	Tajikistan
SRR3085258	Tajikistan
SRR3085260	Italy
SRR3085261	Tajikistan
SRR3085262	Tajikistan
SRR3085263	Tajikistan
SRR3085264	Tajikistan
SRR3085265	Tajikistan
SRR3085266	Tunisia
SRR3085267	Tunisia
SRR3085268	Tajikistan
SRR3085269	Tajikistan
SRR3085270	Tajikistan
SRR3085271	Tajikistan
SRR3085272	Tajikistan
SRR3085273	Tajikistan
SRR3085274	Tajikistan
SRR3085275	Italy
SRR3085276	Tajikistan
SRR3085277	Tajikistan
SRR3085278	Tajikistan
SRR3085280	Tajikistan
SRR3085281	Italy
SRR3085282	Italy
SRR3085283	Italy
SRR3085284	Italy
SRR3085285	Tajikistan
SRR3085286	Tajikistan
SRR3085287	Tajikistan
SRR3085288	Tajikistan
SRR3085289	Tajikistan
SRR3085290	Italy
SRR3085291	Tajikistan
SRR3085292	Italy
SRR3085293	Tajikistan
SRR3085294	Italy
SRR3085295	Tajikistan
SRR3085296	Tajikistan
SRR3085297	Italy
SRR3085298	Mozambique
SRR3085299	Italy
SRR3085300	Mozambique
SRR3085301	Italy
SRR3085302	Tajikistan
SRR3085303	Tunisia
SRR3085304	Tunisia
SRR3085305	Tunisia
SRR3085306	Tunisia
SRR3085307	Italy
SRR3085308	Djibouti

SRR3085309	Djibouti
SRR3085310	Djibouti
SRR3085311	Djibouti
SRR3085312	Djibouti
SRR3085313	Italy
SRR3085314	Italy
SRR3085315	Italy
SRR3085316	Italy
SRR3085317	Italy
SRR3085318	Italy
SRR3085319	Italy
SRR3085320	Italy
SRR3085321	Italy
SRR3085322	Italy
SRR3085323	Italy
SRR3085324	Italy
SRR3085325	Italy
SRR3085326	Albania
SRR3085327	Albania
SRR3085328	Albania
SRR3085329	Tajikistan
SRR3085330	Djibouti
SRR3085331	Djibouti
SRR3085332	Albania
SRR3085333	Djibouti
SRR3085334	Djibouti
SRR3085335	Djibouti
SRR3085336	Djibouti
SRR3085337	Djibouti
SRR3085338	Djibouti
SRR3085339	Djibouti
SRR3085340	Djibouti
SRR3085342	Djibouti
SRR3085343	Italy
SRR3085344	Italy
SRR3085345	Tajikistan
SRR3085346	Tajikistan
SRR3085347	Tajikistan
SRR3085348	Tunisia
SRR3085349	Tajikistan
SRR3085350	Italy
SRR3085351	Italy
SRR3085352	Italy
SRR3085353	Tajikistan
SRR3085354	Italy
SRR3085355	Italy
SRR3085356	Italy
SRR3085357	Albania
SRR3085358	Italy
SRR3085359	Tajikistan

SRR3085360	Tajikistan
SRR3085361	Tajikistan
SRR3085363	Albania
SRR3085364	Albania
SRR3085365	Tunisia
SRR3085366	Tunisia
SRR3085367	Albania
SRR3085368	Italy
SRR3085369	Italy
SRR3085370	Tunisia
SRR3085371	Albania
SRR3085500	NA
SRR3085514	NA
SRR3085561	NA
SRR3086341	Italy
SRR3086350	Tajikistan
SRR3086352	Italy
SRR3086353	Tajikistan
SRR3086354	Tajikistan
SRR3086355	Tunisia
SRR3086356	Tajikistan
SRR3086357	Tajikistan
SRR3086358	Tajikistan
SRR3086359	Italy
SRR3086360	Tajikistan
SRR3086362	Italy
SRR3086363	Tajikistan
SRR3086364	Tajikistan
SRR3086365	Italy
SRR3086366	Tajikistan
SRR3086367	Tajikistan
SRR3086368	Italy
SRR3086369	Mozambique
SRR3086370	Italy
SRR3086371	Mozambique
SRR3086372	Italy
SRR3086373	Tajikistan
SRR3086374	Tunisia
SRR3086376	Tunisia
SRR3086377	Italy
SRR3086378	Djibouti
SRR3086379	Italy
SRR3086380	Italy
SRR3086403	Italy
SRR3086404	Italy
SRR3086405	Italy
SRR3086406	Italy
SRR3086407	Italy
SRR3103478	Canada
SRR3103479	Canada

SRR3103480	Canada
SRR3103481	Canada
SRR3103482	Canada
SRR3103483	Canada
SRR3103484	Canada
SRR3103485	Canada
SRR3103486	Canada
SRR3103487	Canada
SRR3103488	Canada
SRR3103489	Canada
SRR3103490	Canada
SRR3103491	Canada
SRR3103492	Canada
SRR3103493	Canada
SRR3103494	Canada
SRR3103495	Canada
SRR3103496	Canada
SRR3103497	Canada
SRR3103498	Canada
SRR3103499	Canada
SRR3103500	Canada
SRR3103501	Canada
SRR3103502	Canada
SRR3103503	Canada
SRR3103504	Canada
SRR3103505	Canada
SRR3103506	Canada
SRR3103507	Canada
SRR3103508	Canada
SRR3103509	Canada
SRR3103510	Canada
SRR3103511	Canada
SRR3103512	Canada
SRR3103513	Canada
SRR3103514	Canada
SRR3103515	Canada
SRR3103516	Canada
SRR3103517	Canada
SRR3103518	Canada
SRR3103519	Canada
SRR3103520	Canada
SRR3103521	Canada
SRR3103522	Canada
SRR3103523	Canada
SRR3105730	India
SRR3105731	India
SRR3105732	India
SRR3105733	India
SRR3105734	India
SRR3105735	India

SRR3105736	India
SRR3105737	India
SRR3105738	India
SRR3105739	India
SRR3105740	India
SRR3105741	India
SRR3105742	India
SRR3105743	India
SRR3105744	India
SRR3105745	India
SRR3105746	India
SRR3105747	India
SRR3105748	India
SRR3105749	India
SRR3105750	India
SRR3105751	India
SRR3105752	India
SRR3105753	India
SRR3105754	India
SRR3105755	India
SRR3105756	India
SRR3105757	India
SRR3105758	India
SRR3105759	India
SRR3105760	India
SRR3105761	India
SRR3105762	India
SRR3105763	India
SRR3105764	India
SRR3105765	India
SRR3105766	India
SRR3105767	India
SRR3105768	India
SRR3105769	India
SRR3105770	India
SRR3105771	India
SRR3105772	India
SRR3105773	India
SRR3105774	India
SRR3105775	India
SRR3105776	India
SRR3105777	India
SRR3105778	India
SRR3105779	India
SRR3105780	India
SRR3105781	India
SRR3105782	India
SRR3105783	India
SRR3105784	India
SRR3105785	India

SRR3105786	India
SRR3105787	India
SRR3105788	India
SRR3105789	India
SRR3105790	India
SRR3105791	India
SRR3105792	India
SRR3105793	India
SRR3105794	India
SRR3105795	India
SRR3105796	India
SRR3105797	India
SRR3105798	India
SRR3105799	India
SRR3105800	India
SRR3105801	India
SRR3105802	India
SRR3105803	India
SRR3105804	India
SRR3130008	Israel
SRR3136446	Israel
SRR3136699	Israel
SRR3142001	Israel
SRR3142010	Israel
SRR3142011	Israel
SRR3142012	Israel
SRR3142013	Israel
SRR3205958	Thailand
SRR3205959	Thailand
SRR3205960	Thailand
SRR3205961	Thailand
SRR3205962	Thailand
SRR3205963	Thailand
SRR3205964	Thailand
SRR3391819	Mozambique
SRR3391820	Mozambique
SRR3391832	Mozambique
SRR3391833	Mozambique
SRR3416776	China
SRR3510602	Georgia
SRR3544716	Georgia
SRR3544717	Georgia
SRR3544718	Georgia
SRR3544719	Romania
SRR3544720	Georgia
SRR3544721	Romania
SRR3544722	Georgia
SRR3544723	Romania
SRR3544724	Georgia
SRR3544725	Georgia

SRR3544726	Georgia
SRR3544727	Georgia
SRR3544728	Georgia
SRR3544729	Georgia
SRR3544730	Georgia
SRR3544731	Georgia
SRR3544732	Georgia
SRR3544733	Georgia
SRR3544734	Georgia
SRR3544735	Georgia
SRR3544736	Georgia
SRR3544737	Georgia
SRR3544738	Georgia
SRR3544739	Georgia
SRR3544740	Georgia
SRR3544741	Georgia
SRR3544742	Georgia
SRR3544743	Georgia
SRR3544744	Georgia
SRR3544745	Georgia
SRR3544746	Georgia
SRR3544747	Georgia
SRR3544748	Georgia
SRR3544749	Georgia
SRR3544750	Georgia
SRR3544751	Georgia
SRR3544752	Georgia
SRR3587409	United States of America
SRR3587410	United States of America
SRR3587411	United States of America
SRR3587412	United States of America
SRR3587414	United States of America
SRR3587415	United States of America
SRR3587416	United States of America
SRR3587417	United States of America
SRR3587418	United States of America
SRR3587419	United States of America
SRR3587420	United States of America
SRR3587421	United States of America
SRR3587422	United States of America
SRR3587423	United States of America
SRR3587425	United States of America
SRR3587426	United States of America
SRR3587439	United States of America
SRR3587486	United States of America
SRR3619764	Myanmar
SRR3619841	Myanmar
SRR3647351	NA
SRR3647362	China
SRR3675208	United Kingdom

SRR3675209	United Kingdom
SRR3675210	United Kingdom
SRR3675211	United Kingdom
SRR3675212	United Kingdom
SRR3675213	United Kingdom
SRR3675214	United Kingdom
SRR3675215	United Kingdom
SRR3675216	United Kingdom
SRR3675217	United Kingdom
SRR3675218	United Kingdom
SRR3675219	United Kingdom
SRR3675220	United Kingdom
SRR3675221	United Kingdom
SRR3675222	United Kingdom
SRR3675223	United Kingdom
SRR3675224	United Kingdom
SRR3675225	United Kingdom
SRR3675226	United Kingdom
SRR3675227	United Kingdom
SRR3675228	United Kingdom
SRR3675229	United Kingdom
SRR3675230	United Kingdom
SRR3675231	United Kingdom
SRR3675232	United Kingdom
SRR3675233	United Kingdom
SRR3675234	United Kingdom
SRR3675235	United Kingdom
SRR3675236	United Kingdom
SRR3675237	United Kingdom
SRR3675238	United Kingdom
SRR3675239	United Kingdom
SRR3675240	United Kingdom
SRR3675241	United Kingdom
SRR3675242	United Kingdom
SRR3675243	United Kingdom
SRR3675244	United Kingdom
SRR3675245	United Kingdom
SRR3675246	United Kingdom
SRR3675247	United Kingdom
SRR3675248	United Kingdom
SRR3675249	United Kingdom
SRR3675250	United Kingdom
SRR3675251	United Kingdom
SRR3675252	United Kingdom
SRR3675253	United Kingdom
SRR3675254	United Kingdom
SRR3675255	United Kingdom
SRR3675256	United Kingdom
SRR3675257	United Kingdom
SRR3675258	United Kingdom

SRR3675259	United Kingdom
SRR3675260	United Kingdom
SRR3675261	United Kingdom
SRR3675262	United Kingdom
SRR3675263	United Kingdom
SRR3675264	United Kingdom
SRR3675265	United Kingdom
SRR3675266	United Kingdom
SRR3675267	United Kingdom
SRR3675269	United Kingdom
SRR3675270	United Kingdom
SRR3675271	United Kingdom
SRR3675272	United Kingdom
SRR3675273	United Kingdom
SRR3675274	United Kingdom
SRR3675275	United Kingdom
SRR3675276	United Kingdom
SRR3675277	United Kingdom
SRR3675278	United Kingdom
SRR3675279	United Kingdom
SRR3675280	United Kingdom
SRR3675281	United Kingdom
SRR3675282	United Kingdom
SRR3675283	United Kingdom
SRR3675284	United Kingdom
SRR3675285	United Kingdom
SRR3675286	United Kingdom
SRR3675287	United Kingdom
SRR3675288	United Kingdom
SRR3675289	United Kingdom
SRR3675290	United Kingdom
SRR3675291	United Kingdom
SRR3675292	United Kingdom
SRR3675311	United Kingdom
SRR3675312	United Kingdom
SRR3675313	United Kingdom
SRR3675314	United Kingdom
SRR3675315	United Kingdom
SRR3675316	United Kingdom
SRR3675317	United Kingdom
SRR3675318	United Kingdom
SRR3675319	United Kingdom
SRR3675320	United Kingdom
SRR3675321	United Kingdom
SRR3675322	United Kingdom
SRR3675323	United Kingdom
SRR3675336	United Kingdom
SRR3675361	United Kingdom
SRR3675383	United Kingdom
SRR3675384	United Kingdom

SRR3675385	United Kingdom
SRR3675386	United Kingdom
SRR3675387	United Kingdom
SRR3675388	United Kingdom
SRR3675389	United Kingdom
SRR3675390	United Kingdom
SRR3675391	United Kingdom
SRR3675412	United Kingdom
SRR3675439	United Kingdom
SRR3675440	United Kingdom
SRR3675441	United Kingdom
SRR3675442	United Kingdom
SRR3675443	United Kingdom
SRR3675444	United Kingdom
SRR3675445	United Kingdom
SRR3675446	United Kingdom
SRR3675447	United Kingdom
SRR3675448	United Kingdom
SRR3675449	United Kingdom
SRR3675450	United Kingdom
SRR3675451	United Kingdom
SRR3675452	United Kingdom
SRR3675453	United Kingdom
SRR3675454	United Kingdom
SRR3675455	United Kingdom
SRR3675456	United Kingdom
SRR3675457	United Kingdom
SRR3675458	United Kingdom
SRR3675459	United Kingdom
SRR3675460	United Kingdom
SRR3675461	United Kingdom
SRR3675462	United Kingdom
SRR3675463	United Kingdom
SRR3675464	United Kingdom
SRR3675465	United Kingdom
SRR3675466	United Kingdom
SRR3675467	United Kingdom
SRR3675468	United Kingdom
SRR3675469	United Kingdom
SRR3675471	United Kingdom
SRR3675472	United Kingdom
SRR3675473	United Kingdom
SRR3675474	United Kingdom
SRR3675475	United Kingdom
SRR3675476	United Kingdom
SRR3675477	United Kingdom
SRR3675478	United Kingdom
SRR3675479	United Kingdom
SRR3675480	United Kingdom
SRR3675481	United Kingdom

SRR3675482	United Kingdom
SRR3675483	United Kingdom
SRR3675484	United Kingdom
SRR3675485	United Kingdom
SRR3675486	United Kingdom
SRR3675487	United Kingdom
SRR3675488	United Kingdom
SRR3675489	United Kingdom
SRR3675490	United Kingdom
SRR3675491	United Kingdom
SRR3675492	United Kingdom
SRR3675493	United Kingdom
SRR3675494	United Kingdom
SRR3675495	United Kingdom
SRR3675496	United Kingdom
SRR3675497	United Kingdom
SRR3675498	United Kingdom
SRR3675499	United Kingdom
SRR3675500	United Kingdom
SRR3675501	United Kingdom
SRR3675502	United Kingdom
SRR3675503	United Kingdom
SRR3675504	United Kingdom
SRR3675505	United Kingdom
SRR3675506	United Kingdom
SRR3675507	United Kingdom
SRR3675508	United Kingdom
SRR3675509	United Kingdom
SRR3675510	United Kingdom
SRR3675511	United Kingdom
SRR3675512	United Kingdom
SRR3675513	United Kingdom
SRR3675514	United Kingdom
SRR3675515	United Kingdom
SRR3675517	United Kingdom
SRR3675518	United Kingdom
SRR3675519	United Kingdom
SRR3675520	United Kingdom
SRR3675521	United Kingdom
SRR3675522	United Kingdom
SRR3675523	United Kingdom
SRR3675524	United Kingdom
SRR3675525	United Kingdom
SRR3675526	United Kingdom
SRR3675527	United Kingdom
SRR3675528	United Kingdom
SRR3675529	United Kingdom
SRR3675530	United Kingdom
SRR3675531	United Kingdom
SRR3675532	United Kingdom

SRR3675533	United Kingdom
SRR3675534	United Kingdom
SRR3675535	United Kingdom
SRR3675536	United Kingdom
SRR3675537	United Kingdom
SRR3675538	United Kingdom
SRR3675539	United Kingdom
SRR3675540	United Kingdom
SRR3675541	United Kingdom
SRR3675542	United Kingdom
SRR3675543	United Kingdom
SRR3675544	United Kingdom
SRR3675545	United Kingdom
SRR3675546	United Kingdom
SRR3675547	United Kingdom
SRR3675548	United Kingdom
SRR3675549	United Kingdom
SRR3675550	United Kingdom
SRR3675552	United Kingdom
SRR3675553	United Kingdom
SRR3675554	United Kingdom
SRR3675555	United Kingdom
SRR3675556	United Kingdom
SRR3675557	United Kingdom
SRR3675558	United Kingdom
SRR3675559	United Kingdom
SRR3675560	United Kingdom
SRR3675561	United Kingdom
SRR3675562	United Kingdom
SRR3675563	United Kingdom
SRR3675564	United Kingdom
SRR3675565	United Kingdom
SRR3675566	United Kingdom
SRR3675567	United Kingdom
SRR3675568	United Kingdom
SRR3675569	United Kingdom
SRR3675570	United Kingdom
SRR3675571	United Kingdom
SRR3675572	United Kingdom
SRR3675573	United Kingdom
SRR3675574	United Kingdom
SRR3675575	United Kingdom
SRR3675576	United Kingdom
SRR3675577	United Kingdom
SRR3675578	United Kingdom
SRR3675579	United Kingdom
SRR3675580	United Kingdom
SRR3675581	United Kingdom
SRR3675582	United Kingdom
SRR3675583	United Kingdom

SRR3675584	United Kingdom
SRR3675585	United Kingdom
SRR3675586	United Kingdom
SRR3675587	United Kingdom
SRR3675588	United Kingdom
SRR3675589	United Kingdom
SRR3675590	United Kingdom
SRR3675591	United Kingdom
SRR3732567	Peru
SRR3732568	Peru
SRR3732569	Peru
SRR3732570	Peru
SRR3732571	Peru
SRR3732572	Peru
SRR3732573	Peru
SRR3732574	Peru
SRR3732575	Peru
SRR3732576	Peru
SRR3732577	Peru
SRR3732578	Peru
SRR3732579	Peru
SRR3732580	Peru
SRR3732581	Peru
SRR3732582	Peru
SRR3732583	Peru
SRR3732584	Peru
SRR3732585	Peru
SRR3732586	Peru
SRR3732587	Peru
SRR3732588	Peru
SRR3732589	Peru
SRR3732590	Peru
SRR3732591	Peru
SRR3732592	Peru
SRR3732593	Peru
SRR3732594	Peru
SRR3732595	Peru
SRR3732596	Peru
SRR3732620	Peru
SRR3732621	Peru
SRR3732622	Peru
SRR3732641	Peru
SRR3732642	Peru
SRR3732643	Cote d'Ivoire
SRR3732644	Cote d'Ivoire
SRR3732645	Peru
SRR3732646	Cote d'Ivoire
SRR3732647	Cote d'Ivoire
SRR3732648	Peru
SRR3732649	Peru

SRR3732650	Peru
SRR3732651	Peru
SRR3732652	Cote d'Ivoire
SRR3732653	Peru
SRR3732654	Cote d'Ivoire
SRR3732655	Cote d'Ivoire
SRR3732678	Peru
SRR3732679	South Africa
SRR3732680	South Africa
SRR3732681	Peru
SRR3732682	Peru
SRR3732683	Peru
SRR3732684	Cote d'Ivoire
SRR3732685	Peru
SRR3732686	Peru
SRR3732687	Peru
SRR3732689	South Africa
SRR3732690	Peru
SRR3732691	Cote d'Ivoire
SRR3732692	Peru
SRR3732693	Peru
SRR3732694	Cote d'Ivoire
SRR3732695	Cote d'Ivoire
SRR3732696	Kenya
SRR3732697	Peru
SRR3732698	Cote d'Ivoire
SRR3732699	Peru
SRR3732700	Peru
SRR3732701	Peru
SRR3732702	Peru
SRR3732703	Cote d'Ivoire
SRR3732704	Cote d'Ivoire
SRR3732705	Peru
SRR3732706	Cote d'Ivoire
SRR3732707	Cote d'Ivoire
SRR3732708	South Africa
SRR3732709	South Africa
SRR3732710	Peru
SRR3732711	Peru
SRR3732712	Peru
SRR3732713	South Africa
SRR3732714	Peru
SRR3732715	Cote d'Ivoire
SRR3732716	Peru
SRR3732717	Cote d'Ivoire
SRR3732718	Democratic Republic of the Congo
SRR3732719	Cote d'Ivoire
SRR3732720	Peru
SRR3732721	Peru
SRR3732722	Peru

SRR3732723	Peru
SRR3732724	Peru
SRR3732725	Cote d'Ivoire
SRR3732726	Cote d'Ivoire
SRR3742653	China
SRR3742654	China
SRR3742655	China
SRR3742656	China
SRR3742657	China
SRR3742658	China
SRR3742659	China
SRR3742660	China
SRR3742661	China
SRR3742662	China
SRR3742663	China
SRR3742664	China
SRR3742665	China
SRR3742666	China
SRR3742667	China
SRR3742668	China
SRR3742669	China
SRR3742670	China
SRR3743189	Romania
SRR3743190	Romania
SRR3743191	Georgia
SRR3743192	Romania
SRR3743199	Romania
SRR3743200	Romania
SRR3743201	Romania
SRR3743202	Romania
SRR3743203	Georgia
SRR3743365	Moldova
SRR3743366	Moldova
SRR3743367	Moldova
SRR3743368	Moldova
SRR3743369	Moldova
SRR3743370	Moldova
SRR3743371	Moldova
SRR3743372	Moldova
SRR3743373	Moldova
SRR3743374	Moldova
SRR3743375	Moldova
SRR3743376	Moldova
SRR3743377	Moldova
SRR3743378	Moldova
SRR3743379	Moldova
SRR3743380	Moldova
SRR3743381	Moldova
SRR3743382	Moldova
SRR3743383	Moldova

SRR3743384	Moldova
SRR3743385	Moldova
SRR3743386	Moldova
SRR3743387	Moldova
SRR3743388	Moldova
SRR3743389	Moldova
SRR3743390	Moldova
SRR3743391	Moldova
SRR3743392	Moldova
SRR3743393	Moldova
SRR3743394	Moldova
SRR3743395	Moldova
SRR3743396	Moldova
SRR3743397	Moldova
SRR3743398	Moldova
SRR3743399	Moldova
SRR3743400	Moldova
SRR3743401	Moldova
SRR3743402	Moldova
SRR3743403	Moldova
SRR3743404	Moldova
SRR3743405	Moldova
SRR3743406	Moldova
SRR3743407	Moldova
SRR3743408	Moldova
SRR3743409	Moldova
SRR3743410	Moldova
SRR3743411	Moldova
SRR3743412	Moldova
SRR3743413	Moldova
SRR3743414	Moldova
SRR3743415	Moldova
SRR3743416	Moldova
SRR3743433	Moldova
SRR3743434	Moldova
SRR3743435	Moldova
SRR3743436	Moldova
SRR3743437	Moldova
SRR3743438	Moldova
SRR3743449	Moldova
SRR3743450	Moldova
SRR3743457	Moldova
SRR3743458	Moldova
SRR3743459	Moldova
SRR3743460	Moldova
SRR3743461	Moldova
SRR3743462	Moldova
SRR3743463	Moldova
SRR3743472	Moldova
SRR3743473	Moldova

SRR3743474	Moldova
SRR3743475	Moldova
SRR3743476	Moldova
SRR3743477	Moldova
SRR3743478	Moldova
SRR3743479	Moldova
SRR3743480	Moldova
SRR3743481	Moldova
SRR3743482	Moldova
SRR3743483	Moldova
SRR3743484	Moldova
SRR3743485	Moldova
SRR3743486	Moldova
SRR3743487	Moldova
SRR3743488	Moldova
SRR3743489	Moldova
SRR3743490	Moldova
SRR3743491	Moldova
SRR3743492	Moldova
SRR3743493	Moldova
SRR3743494	Moldova
SRR3743495	Moldova
SRR3743496	Moldova
SRR3743497	Moldova
SRR3743498	Moldova
SRR3743499	Moldova
SRR3743500	Moldova
SRR3880247	United States of America
SRR4033087	South Africa
SRR4033088	South Africa
SRR4033089	South Africa
SRR4033090	South Africa
SRR4033091	South Africa
SRR4033092	South Africa
SRR4033093	South Africa
SRR4033094	South Africa
SRR4033095	South Africa
SRR4033096	South Africa
SRR4033097	South Africa
SRR4033098	South Africa
SRR4033099	South Africa
SRR4033100	South Africa
SRR4033101	South Africa
SRR4033102	South Africa
SRR4033103	South Africa
SRR4033105	South Africa
SRR4033106	South Africa
SRR4033107	South Africa
SRR4033108	South Africa
SRR4033109	South Africa

SRR4033110	South Africa
SRR4033111	South Africa
SRR4033112	South Africa
SRR4033113	South Africa
SRR4033114	South Africa
SRR4033115	South Africa
SRR4033116	South Africa
SRR4033117	South Africa
SRR4033118	South Africa
SRR4033119	South Africa
SRR4033120	South Africa
SRR4033121	South Africa
SRR4033122	South Africa
SRR4033123	South Africa
SRR4033124	South Africa
SRR4033125	South Africa
SRR4033126	South Africa
SRR4033127	South Africa
SRR4033128	South Africa
SRR4033129	South Africa
SRR4033130	South Africa
SRR4033131	South Africa
SRR4033132	South Africa
SRR4033133	South Africa
SRR4033134	South Africa
SRR4033135	South Africa
SRR4033136	South Africa
SRR4033137	South Africa
SRR4033138	South Africa
SRR4033139	South Africa
SRR4033140	South Africa
SRR4033141	South Africa
SRR4033142	South Africa
SRR4033143	South Africa
SRR4033144	South Africa
SRR4033145	South Africa
SRR4033146	South Africa
SRR4033147	South Africa
SRR4033148	South Africa
SRR4033149	South Africa
SRR4033150	South Africa
SRR4033151	South Africa
SRR4033152	South Africa
SRR4033153	South Africa
SRR4033154	South Africa
SRR4033155	South Africa
SRR4033156	South Africa
SRR4033157	South Africa
SRR4033158	South Africa
SRR4033159	South Africa

SRR4033160	South Africa
SRR4033161	South Africa
SRR4033163	South Africa
SRR4033164	South Africa
SRR4033165	South Africa
SRR4033166	South Africa
SRR4033167	South Africa
SRR4033168	South Africa
SRR4033169	South Africa
SRR4033170	South Africa
SRR4033171	South Africa
SRR4033172	South Africa
SRR4033173	South Africa
SRR4033174	South Africa
SRR4033175	South Africa
SRR4033176	South Africa
SRR4033177	South Africa
SRR4033178	South Africa
SRR4033179	South Africa
SRR4033180	South Africa
SRR4033181	South Africa
SRR4033182	South Africa
SRR4033183	South Africa
SRR4033184	South Africa
SRR4033185	South Africa
SRR4033186	South Africa
SRR4033187	South Africa
SRR4033188	South Africa
SRR4033189	South Africa
SRR4033190	South Africa
SRR4033191	South Africa
SRR4033192	South Africa
SRR4033193	South Africa
SRR4033194	South Africa
SRR4033195	South Africa
SRR4033196	South Africa
SRR4033197	South Africa
SRR4033198	South Africa
SRR4033199	South Africa
SRR4033201	South Africa
SRR4033202	South Africa
SRR4033203	South Africa
SRR4033204	South Africa
SRR4033205	South Africa
SRR4033206	South Africa
SRR4033207	South Africa
SRR4033208	South Africa
SRR4033209	South Africa
SRR4033210	South Africa
SRR4033211	South Africa

SRR4033212	South Africa
SRR4033213	South Africa
SRR4033214	South Africa
SRR4033215	South Africa
SRR4033216	South Africa
SRR4033217	South Africa
SRR4033218	South Africa
SRR4033219	South Africa
SRR4033220	South Africa
SRR4033221	South Africa
SRR4033222	South Africa
SRR4033223	South Africa
SRR4033224	South Africa
SRR4033225	South Africa
SRR4033226	South Africa
SRR4033227	South Africa
SRR4033228	South Africa
SRR4033229	South Africa
SRR4033230	South Africa
SRR4033231	South Africa
SRR4033232	South Africa
SRR4033233	South Africa
SRR4033234	South Africa
SRR4033236	South Africa
SRR4033237	South Africa
SRR4033238	South Africa
SRR4033239	South Africa
SRR4033240	South Africa
SRR4033241	South Africa
SRR4033242	South Africa
SRR4033243	South Africa
SRR4033244	South Africa
SRR4033245	South Africa
SRR4033246	South Africa
SRR4033247	South Africa
SRR4033248	South Africa
SRR4033249	South Africa
SRR4033250	South Africa
SRR4033251	South Africa
SRR4033253	South Africa
SRR4033254	South Africa
SRR4033255	South Africa
SRR4033256	South Africa
SRR4033257	South Africa
SRR4033258	South Africa
SRR4033259	South Africa
SRR4033260	South Africa
SRR4033261	South Africa
SRR4033262	South Africa
SRR4033263	South Africa

SRR4033264	South Africa
SRR4033265	South Africa
SRR4033266	South Africa
SRR4033267	South Africa
SRR4033268	South Africa
SRR4033269	South Africa
SRR4033270	South Africa
SRR4033271	South Africa
SRR4033272	South Africa
SRR4033273	South Africa
SRR4033274	South Africa
SRR4033275	South Africa
SRR4033277	South Africa
SRR4033278	South Africa
SRR4033279	South Africa
SRR4033280	South Africa
SRR4033281	South Africa
SRR4033282	South Africa
SRR4033283	South Africa
SRR4033284	South Africa
SRR4033285	South Africa
SRR4033286	South Africa
SRR4033287	South Africa
SRR4033288	South Africa
SRR4033289	South Africa
SRR4033290	South Africa
SRR4033291	South Africa
SRR4033292	South Africa
SRR4033293	South Africa
SRR4033294	South Africa
SRR4033295	South Africa
SRR4033296	South Africa
SRR4033297	South Africa
SRR4033298	South Africa
SRR4033299	South Africa
SRR4033300	South Africa
SRR4033301	South Africa
SRR4033302	South Africa
SRR4033303	South Africa
SRR4033304	South Africa
SRR4033305	South Africa
SRR4033306	South Africa
SRR4033307	South Africa
SRR4033308	South Africa
SRR4033310	South Africa
SRR4033311	South Africa
SRR4033312	South Africa
SRR4033313	South Africa
SRR4033314	South Africa
SRR4033315	South Africa

SRR4033316	South Africa
SRR4033317	South Africa
SRR4033318	South Africa
SRR4033319	South Africa
SRR4033320	South Africa
SRR4033321	South Africa
SRR4033322	South Africa
SRR4033323	South Africa
SRR4033324	South Africa
SRR4033325	South Africa
SRR4033326	South Africa
SRR4033328	South Africa
SRR4033329	South Africa
SRR4033330	South Africa
SRR4033331	South Africa
SRR4033332	South Africa
SRR4033333	South Africa
SRR4033334	South Africa
SRR4033335	South Africa
SRR4033336	South Africa
SRR4033337	South Africa
SRR4033338	South Africa
SRR4033339	South Africa
SRR4033340	South Africa
SRR4033341	South Africa
SRR4033342	South Africa
SRR4033344	South Africa
SRR4033345	South Africa
SRR4033346	South Africa
SRR4033347	South Africa
SRR4033348	South Africa
SRR4033349	South Africa
SRR4033350	South Africa
SRR4033351	South Africa
SRR4033352	South Africa
SRR4033353	South Africa
SRR4033354	South Africa
SRR4033355	South Africa
SRR4033356	South Africa
SRR4033357	South Africa
SRR4033358	South Africa
SRR4033359	South Africa
SRR4033360	South Africa
SRR4033361	South Africa
SRR4033362	South Africa
SRR4033363	South Africa
SRR4033365	South Africa
SRR4033366	South Africa
SRR4033367	South Africa
SRR4033368	South Africa

SRR4033369	South Africa
SRR4033370	South Africa
SRR4033371	South Africa
SRR4033372	South Africa
SRR4033373	South Africa
SRR4033374	South Africa
SRR4033375	South Africa
SRR4033376	South Africa
SRR4033377	South Africa
SRR4033378	South Africa
SRR4033379	South Africa
SRR4033381	South Africa
SRR4033382	South Africa
SRR4033383	South Africa
SRR4033384	South Africa
SRR4033385	South Africa
SRR4033386	South Africa
SRR4033387	South Africa
SRR4033388	South Africa
SRR4033389	South Africa
SRR4033390	South Africa
SRR4033391	South Africa
SRR4033392	South Africa
SRR4033393	South Africa
SRR4033394	South Africa
SRR4033395	South Africa
SRR4033396	South Africa
SRR4033397	South Africa
SRR4033398	South Africa
SRR4033399	South Africa
SRR4033400	South Africa
SRR4033402	South Africa
SRR4033403	South Africa
SRR4033404	South Africa
SRR4033405	South Africa
SRR4033406	South Africa
SRR4033407	South Africa
SRR4033408	South Africa
SRR4033409	South Africa
SRR4033410	South Africa
SRR4033411	South Africa
SRR4033412	South Africa
SRR4033413	South Africa
SRR4033414	South Africa
SRR4033415	South Africa
SRR4033416	South Africa
SRR4033417	South Africa
SRR4033419	South Africa
SRR4033420	South Africa
SRR4033421	South Africa

SRR4033422	South Africa
SRR4033423	South Africa
SRR4033424	South Africa
SRR4033425	South Africa
SRR4033426	South Africa
SRR4033427	South Africa
SRR4033428	South Africa
SRR4033429	South Africa
SRR4033430	South Africa
SRR4033431	South Africa
SRR4033432	South Africa
SRR4033433	South Africa
SRR4033434	South Africa
SRR4033435	South Africa
SRR4033436	South Africa
SRR4033437	South Africa
SRR4033438	South Africa
SRR4033439	South Africa
SRR4033440	South Africa
SRR4033441	South Africa
SRR4033442	South Africa
SRR4033443	South Africa
SRR4033444	South Africa
SRR4033445	South Africa
SRR4033446	South Africa
SRR4033447	South Africa
SRR4033448	South Africa
SRR4033449	South Africa
SRR4033450	South Africa
SRR4033451	South Africa
SRR4033452	South Africa
SRR4033453	South Africa
SRR4033454	South Africa
SRR4033455	South Africa
SRR4033456	South Africa
SRR4033457	South Africa
SRR4033458	South Africa
SRR4033459	South Africa
SRR4033460	South Africa
SRR4033461	South Africa
SRR4033462	South Africa
SRR4033463	South Africa
SRR4033464	South Africa
SRR4033465	South Africa
SRR4033466	South Africa
SRR4033467	South Africa
SRR4033468	South Africa
SRR4033469	South Africa
SRR4033470	South Africa
SRR4033471	South Africa

SRR4033472	South Africa
SRR4033473	South Africa
SRR4033474	South Africa
SRR4033475	South Africa
SRR4033476	South Africa
SRR4033477	South Africa
SRR4033478	South Africa
SRR4033479	South Africa
SRR4033480	South Africa
SRR4033481	South Africa
SRR4033482	South Africa
SRR4033483	South Africa
SRR4033484	South Africa
SRR4033485	South Africa
SRR4033486	South Africa
SRR4033487	South Africa
SRR4033488	South Africa
SRR4033489	South Africa
SRR4033490	South Africa
SRR4033491	South Africa
SRR4033492	South Africa
SRR4033493	South Africa
SRR4033494	South Africa
SRR4033495	South Africa
SRR4033496	South Africa
SRR4033497	South Africa
SRR4033498	South Africa
SRR4033499	South Africa
SRR4033500	South Africa
SRR4033501	South Africa
SRR4033502	South Africa
SRR4033503	South Africa
SRR4033504	South Africa
SRR4033505	South Africa
SRR4033506	South Africa
SRR4033507	South Africa
SRR4033508	South Africa
SRR4033509	South Africa
SRR4033510	South Africa
SRR4033511	South Africa
SRR4033512	South Africa
SRR4033513	South Africa
SRR4033514	South Africa
SRR4033515	South Africa
SRR4033516	South Africa
SRR4033517	South Africa
SRR4033518	South Africa
SRR4033519	South Africa
SRR4033520	South Africa
SRR4033521	South Africa

SRR4033522	South Africa
SRR4033523	South Africa
SRR4033524	South Africa
SRR4033525	South Africa
SRR4033526	South Africa
SRR4033527	South Africa
SRR4033528	South Africa
SRR4033529	South Africa
SRR4033530	South Africa
SRR4033531	South Africa
SRR4033532	South Africa
SRR4033533	South Africa
SRR4033534	South Africa
SRR4033535	South Africa
SRR4033536	South Africa
SRR4033537	South Africa
SRR4033538	South Africa
SRR4033539	South Africa
SRR4033540	South Africa
SRR4033541	South Africa
SRR4033542	South Africa
SRR4033543	South Africa
SRR4033544	South Africa
SRR4033545	South Africa
SRR4033546	South Africa
SRR4033547	South Africa
SRR4033548	South Africa
SRR4033549	South Africa
SRR4033550	South Africa
SRR4033551	South Africa
SRR4033552	South Africa
SRR4033553	South Africa
SRR4033554	South Africa
SRR4033555	South Africa
SRR4033556	South Africa
SRR4033557	South Africa
SRR4033558	South Africa
SRR4033559	South Africa
SRR4033560	South Africa
SRR4033561	South Africa
SRR4033562	South Africa
SRR4033563	South Africa
SRR4033564	South Africa
SRR4033565	South Africa
SRR4033566	South Africa
SRR4033567	South Africa
SRR4033568	South Africa
SRR4033569	South Africa
SRR4033570	South Africa
SRR4033571	South Africa

SRR4033572	South Africa
SRR4033573	South Africa
SRR4033574	South Africa
SRR4033575	South Africa
SRR4033576	South Africa
SRR4033577	South Africa
SRR4033578	South Africa
SRR4033579	South Africa
SRR4033580	South Africa
SRR4033581	South Africa
SRR4033582	South Africa
SRR4033583	South Africa
SRR4033584	South Africa
SRR4033585	South Africa
SRR4033586	South Africa
SRR4033587	South Africa
SRR4033588	South Africa
SRR4033589	South Africa
SRR4033590	South Africa
SRR4033591	South Africa
SRR4033592	South Africa
SRR4033593	South Africa
SRR4033594	South Africa
SRR4033595	South Africa
SRR4033596	South Africa
SRR4033597	South Africa
SRR4033598	South Africa
SRR4033605	South Africa
SRR4033606	South Africa
SRR4033607	South Africa
SRR4033608	South Africa
SRR4033609	South Africa
SRR4033610	South Africa
SRR4033611	South Africa
SRR4033612	South Africa
SRR4033613	South Africa
SRR4033614	South Africa
SRR4033615	South Africa
SRR4033616	South Africa
SRR4033617	South Africa
SRR4033618	South Africa
SRR4033619	South Africa
SRR4033620	South Africa
SRR4033621	South Africa
SRR4033622	South Africa
SRR4033623	South Africa
SRR4033624	South Africa
SRR4033625	South Africa
SRR4033626	South Africa
SRR4033627	South Africa

SRR4033628	South Africa
SRR4033629	South Africa
SRR4033630	South Africa
SRR4033631	South Africa
SRR4033648	South Africa
SRR4033649	South Africa
SRR4033650	South Africa
SRR4033651	South Africa
SRR4033652	South Africa
SRR4033653	South Africa
SRR4033654	South Africa
SRR4033655	South Africa
SRR4033656	South Africa
SRR4033657	South Africa
SRR4033658	South Africa
SRR4033659	South Africa
SRR4033660	South Africa
SRR4033661	South Africa
SRR4033662	South Africa
SRR4033663	South Africa
SRR4033664	South Africa
SRR4033665	South Africa
SRR4033666	South Africa
SRR4033667	South Africa
SRR4033668	South Africa
SRR4033669	South Africa
SRR4033670	South Africa
SRR4033671	South Africa
SRR4033672	South Africa
SRR4033673	South Africa
SRR4033674	South Africa
SRR4033675	South Africa
SRR4033676	South Africa
SRR4033677	South Africa
SRR4033678	South Africa
SRR4033679	South Africa
SRR4033680	South Africa
SRR4033681	South Africa
SRR4033682	South Africa
SRR4033683	South Africa
SRR4033684	South Africa
SRR4033685	South Africa
SRR4033686	South Africa
SRR4033687	South Africa
SRR4033688	South Africa
SRR4033689	South Africa
SRR4033690	South Africa
SRR4033691	South Africa
SRR4033692	South Africa
SRR4033693	South Africa

SRR4033694	South Africa
SRR4033695	South Africa
SRR4033696	South Africa
SRR4033697	South Africa
SRR4033698	South Africa
SRR4033699	South Africa
SRR4033700	South Africa
SRR4033701	South Africa
SRR4033702	South Africa
SRR4033703	South Africa
SRR4033704	South Africa
SRR4033705	South Africa
SRR4033706	South Africa
SRR4033707	South Africa
SRR4033708	South Africa
SRR4033709	South Africa
SRR4033710	South Africa
SRR4033711	South Africa
SRR4033712	South Africa
SRR4033713	South Africa
SRR4033714	South Africa
SRR4033715	South Africa
SRR4033716	South Africa
SRR4033717	South Africa
SRR4033718	South Africa
SRR4033719	South Africa
SRR4033720	South Africa
SRR4033721	South Africa
SRR4033722	South Africa
SRR4033723	South Africa
SRR4033724	South Africa
SRR4033725	South Africa
SRR4033726	South Africa
SRR4033727	South Africa
SRR4033728	South Africa
SRR4033729	South Africa
SRR4033730	South Africa
SRR4033731	South Africa
SRR4033732	South Africa
SRR4033733	South Africa
SRR4033734	South Africa
SRR4033735	South Africa
SRR4033736	South Africa
SRR4033737	South Africa
SRR4033738	South Africa
SRR4033739	South Africa
SRR4033740	South Africa
SRR4033741	South Africa
SRR4033742	South Africa
SRR4033743	South Africa

SRR4033744	South Africa
SRR4033745	South Africa
SRR4033746	South Africa
SRR4033747	South Africa
SRR4033748	South Africa
SRR4033749	South Africa
SRR4033750	South Africa
SRR4033751	South Africa
SRR4033752	South Africa
SRR4033753	South Africa
SRR4033754	South Africa
SRR4033755	South Africa
SRR4033756	South Africa
SRR4033757	South Africa
SRR4033758	South Africa
SRR4033759	South Africa
SRR4033760	South Africa
SRR4033761	South Africa
SRR4033762	South Africa
SRR4033763	South Africa
SRR4033764	South Africa
SRR4033765	South Africa
SRR4033766	South Africa
SRR4033767	South Africa
SRR4033768	South Africa
SRR4033769	South Africa
SRR4033770	South Africa
SRR4033771	South Africa
SRR4033772	South Africa
SRR4033773	South Africa
SRR4033774	South Africa
SRR4033775	South Africa
SRR4033776	South Africa
SRR4033777	South Africa
SRR4033778	South Africa
SRR4033779	South Africa
SRR4033780	South Africa
SRR4033781	South Africa
SRR4033782	South Africa
SRR4033783	South Africa
SRR4033784	South Africa
SRR4033785	South Africa
SRR4033786	South Africa
SRR4033787	South Africa
SRR4033788	South Africa
SRR4033789	South Africa
SRR4033790	South Africa
SRR4033791	South Africa
SRR4033792	South Africa
SRR4033793	South Africa

SRR4033794	South Africa
SRR4033795	South Africa
SRR4033796	South Africa
SRR4033797	South Africa
SRR4033798	South Africa
SRR4033799	South Africa
SRR4033800	South Africa
SRR4033801	South Africa
SRR4033802	South Africa
SRR4033803	South Africa
SRR4033804	South Africa
SRR4033805	South Africa
SRR4033806	South Africa
SRR4033807	South Africa
SRR4033808	South Africa
SRR4033809	South Africa
SRR4033810	South Africa
SRR4033811	South Africa
SRR4033812	South Africa
SRR4033813	South Africa
SRR4033814	South Africa
SRR4033815	South Africa
SRR4033816	South Africa
SRR4033817	South Africa
SRR4033818	South Africa
SRR4033819	South Africa
SRR4033820	South Africa
SRR4033821	South Africa
SRR4033822	South Africa
SRR4033823	South Africa
SRR4033824	South Africa
SRR4033825	South Africa
SRR4033826	South Africa
SRR4033827	South Africa
SRR4033828	South Africa
SRR4033829	South Africa
SRR4033830	South Africa
SRR4033831	South Africa
SRR4033832	South Africa
SRR4033833	South Africa
SRR4033834	South Africa
SRR4033835	South Africa
SRR4033836	South Africa
SRR4033837	South Africa
SRR4033838	South Africa
SRR4033839	South Africa
SRR4033840	South Africa
SRR4033841	South Africa
SRR4033842	South Africa
SRR4033843	South Africa

SRR4033844	South Africa
SRR4033845	South Africa
SRR4033846	South Africa
SRR4033847	South Africa
SRR4033848	South Africa
SRR4033849	South Africa
SRR4033850	South Africa
SRR4033851	South Africa
SRR4033852	South Africa
SRR4033853	South Africa
SRR4033854	South Africa
SRR4033855	South Africa
SRR4033856	South Africa
SRR4033857	South Africa
SRR4033858	South Africa
SRR4033859	South Africa
SRR4033860	South Africa
SRR4033861	South Africa
SRR4033862	South Africa
SRR4033863	South Africa
SRR4033864	South Africa
SRR4033865	South Africa
SRR4033866	South Africa
SRR4033867	South Africa
SRR4033868	South Africa
SRR4033869	South Africa
SRR4033870	South Africa
SRR4033871	South Africa
SRR4033872	South Africa
SRR4033873	South Africa
SRR4033874	South Africa
SRR4033875	South Africa
SRR4033876	South Africa
SRR4033877	South Africa
SRR4033878	South Africa
SRR4033879	South Africa
SRR4033880	South Africa
SRR4033881	South Africa
SRR4033882	South Africa
SRR4033883	South Africa
SRR4033884	South Africa
SRR4033885	South Africa
SRR4033886	South Africa
SRR4033887	South Africa
SRR4033888	South Africa
SRR4033889	South Africa
SRR4033890	South Africa
SRR4033891	South Africa
SRR4033892	South Africa
SRR4033893	South Africa

SRR4033894	South Africa
SRR4033895	South Africa
SRR4033896	South Africa
SRR4033897	South Africa
SRR4033898	South Africa
SRR4033899	South Africa
SRR4033900	South Africa
SRR4033901	South Africa
SRR4033902	South Africa
SRR4033903	South Africa
SRR4033904	South Africa
SRR4033905	South Africa
SRR4033906	South Africa
SRR4033907	South Africa
SRR4033908	South Africa
SRR4033909	South Africa
SRR4033910	South Africa
SRR4033911	South Africa
SRR4033912	South Africa
SRR4033913	South Africa
SRR4033914	South Africa
SRR4033915	South Africa
SRR4033916	South Africa
SRR4033917	South Africa
SRR4033918	South Africa
SRR4033919	South Africa
SRR4033920	South Africa
SRR4033921	South Africa
SRR4033922	South Africa
SRR4033923	South Africa
SRR4033924	South Africa
SRR4033925	South Africa
SRR4033926	South Africa
SRR4033927	South Africa
SRR4033928	South Africa
SRR4033929	South Africa
SRR4033930	South Africa
SRR4033931	South Africa
SRR4033932	South Africa
SRR4033933	South Africa
SRR4033934	South Africa
SRR4033935	South Africa
SRR4033936	South Africa
SRR4033937	South Africa
SRR4033938	South Africa
SRR4033939	South Africa
SRR4033940	South Africa
SRR4033941	South Africa
SRR4033942	South Africa
SRR4033943	South Africa

SRR4033944	South Africa
SRR4033945	South Africa
SRR4033946	South Africa
SRR4033947	South Africa
SRR4033948	South Africa
SRR4033949	South Africa
SRR4033950	South Africa
SRR4033951	South Africa
SRR4033952	South Africa
SRR4033953	South Africa
SRR4033954	South Africa
SRR4033955	South Africa
SRR4033956	South Africa
SRR4033957	South Africa
SRR4033958	South Africa
SRR4033959	South Africa
SRR4033960	South Africa
SRR4033961	South Africa
SRR4033962	South Africa
SRR4033963	South Africa
SRR4033964	South Africa
SRR4033965	South Africa
SRR4033966	South Africa
SRR4033967	South Africa
SRR4033968	South Africa
SRR4033969	South Africa
SRR4033970	South Africa
SRR4033971	South Africa
SRR4033972	South Africa
SRR4033973	South Africa
SRR4033974	South Africa
SRR4033975	South Africa
SRR4033976	South Africa
SRR4033977	South Africa
SRR4033978	South Africa
SRR4033979	South Africa
SRR4033980	South Africa
SRR4033981	South Africa
SRR4033982	South Africa
SRR4033983	South Africa
SRR4033984	South Africa
SRR4033985	South Africa
SRR4033986	South Africa
SRR4033987	South Africa
SRR4033988	South Africa
SRR4033989	South Africa
SRR4033990	South Africa
SRR4033991	South Africa
SRR4033992	South Africa
SRR4033993	South Africa

SRR4033994	South Africa
SRR4033995	South Africa
SRR4033996	South Africa
SRR4033997	South Africa
SRR4033998	South Africa
SRR4033999	South Africa
SRR4034000	South Africa
SRR4034001	South Africa
SRR4034002	South Africa
SRR4034003	South Africa
SRR4034004	South Africa
SRR4034005	South Africa
SRR4034006	South Africa
SRR4034007	South Africa
SRR4034008	South Africa
SRR4034009	South Africa
SRR4034010	South Africa
SRR4034011	South Africa
SRR4034012	South Africa
SRR4034013	South Africa
SRR4034014	South Africa
SRR4034015	South Africa
SRR4034016	South Africa
SRR4034017	South Africa
SRR4034018	South Africa
SRR4034019	South Africa
SRR4034020	South Africa
SRR4034021	South Africa
SRR4034022	South Africa
SRR4034023	South Africa
SRR4034024	South Africa
SRR4034025	South Africa
SRR4034026	South Africa
SRR4034027	South Africa
SRR4034028	South Africa
SRR4034029	South Africa
SRR4034030	South Africa
SRR4034031	South Africa
SRR4034032	South Africa
SRR4034033	South Africa
SRR4034034	South Africa
SRR4034035	South Africa
SRR4034036	South Africa
SRR4034037	South Africa
SRR4034038	South Africa
SRR4034039	South Africa
SRR4034040	South Africa
SRR4034041	South Africa
SRR4034042	South Africa
SRR4034043	South Africa

SRR4034044	South Africa
SRR4034045	South Africa
SRR4034046	South Africa
SRR4034047	South Africa
SRR4034048	South Africa
SRR4034049	South Africa
SRR4034050	South Africa
SRR4034051	South Africa
SRR4034052	South Africa
SRR4034053	South Africa
SRR4034054	South Africa
SRR4034055	South Africa
SRR4034056	South Africa
SRR4034057	South Africa
SRR4034058	South Africa
SRR4034059	South Africa
SRR4034060	South Africa
SRR4034061	South Africa
SRR4034062	South Africa
SRR4034063	South Africa
SRR4034064	South Africa
SRR4034065	South Africa
SRR4034066	South Africa
SRR4034067	South Africa
SRR4034068	South Africa
SRR4034069	South Africa
SRR4034070	South Africa
SRR4034071	South Africa
SRR4034072	South Africa
SRR4034073	South Africa
SRR4034074	South Africa
SRR4034075	South Africa
SRR4034076	South Africa
SRR4034077	South Africa
SRR4034078	South Africa
SRR4034079	South Africa
SRR4034080	South Africa
SRR4034081	South Africa
SRR4034082	South Africa
SRR4034083	South Africa
SRR4034084	South Africa
SRR4034085	South Africa
SRR4034086	South Africa
SRR4034087	South Africa
SRR4034088	South Africa
SRR4034089	South Africa
SRR4034090	South Africa
SRR4034091	South Africa
SRR4034092	South Africa
SRR4034093	South Africa

SRR4034094	South Africa
SRR4034095	South Africa
SRR4034096	South Africa
SRR4034097	South Africa
SRR4034098	South Africa
SRR4034099	South Africa
SRR4034100	South Africa
SRR4034101	South Africa
SRR4034102	South Africa
SRR4034103	South Africa
SRR4034104	South Africa
SRR4034105	South Africa
SRR4034106	South Africa
SRR4034107	South Africa
SRR4034108	South Africa
SRR4034109	South Africa
SRR4034110	South Africa
SRR4034111	South Africa
SRR4034112	South Africa
SRR4034113	South Africa
SRR4034114	South Africa
SRR4034115	South Africa
SRR4034116	South Africa
SRR4034117	South Africa
SRR4034118	South Africa
SRR4034119	South Africa
SRR4034146	South Africa
SRR4034147	South Africa
SRR4034148	South Africa
SRR4034149	South Africa
SRR4034150	South Africa
SRR4034151	South Africa
SRR4034152	South Africa
SRR4034153	South Africa
SRR4034154	South Africa
SRR4034155	South Africa
SRR4034156	South Africa
SRR4034157	South Africa
SRR4034158	South Africa
SRR4034159	South Africa
SRR4034160	South Africa
SRR4034161	South Africa
SRR4034162	South Africa
SRR4034163	South Africa
SRR4034164	South Africa
SRR4034165	South Africa
SRR4034166	South Africa
SRR4034167	South Africa
SRR4034168	South Africa
SRR4034169	South Africa

SRR4034170	South Africa
SRR4034171	South Africa
SRR4034172	South Africa
SRR4034173	South Africa
SRR4034174	South Africa
SRR4034175	South Africa
SRR4034176	South Africa
SRR4034177	South Africa
SRR4034178	South Africa
SRR4034179	South Africa
SRR4034180	South Africa
SRR4034181	South Africa
SRR4034182	South Africa
SRR4034183	South Africa
SRR4034184	South Africa
SRR4034185	South Africa
SRR4034186	South Africa
SRR4034187	South Africa
SRR4034188	South Africa
SRR4034189	South Africa
SRR4034190	South Africa
SRR4034191	South Africa
SRR4034192	South Africa
SRR4034193	South Africa
SRR4034194	South Africa
SRR4034195	South Africa
SRR4034196	South Africa
SRR4034197	South Africa
SRR4034198	South Africa
SRR4034199	South Africa
SRR4034200	South Africa
SRR4034201	South Africa
SRR4034202	South Africa
SRR4034203	South Africa
SRR4034204	South Africa
SRR4034205	South Africa
SRR4034206	South Africa
SRR4034207	South Africa
SRR4034208	South Africa
SRR4034209	South Africa
SRR4034210	South Africa
SRR4034211	South Africa
SRR4034212	South Africa
SRR4034213	South Africa
SRR4034214	South Africa
SRR4034215	South Africa
SRR4034216	South Africa
SRR4034217	South Africa
SRR4034218	South Africa
SRR4034219	South Africa

SRR4034220	South Africa
SRR4034221	South Africa
SRR4034222	South Africa
SRR4034223	South Africa
SRR4034224	South Africa
SRR4034225	South Africa
SRR4034226	South Africa
SRR4034227	South Africa
SRR4034228	South Africa
SRR4034229	South Africa
SRR4034230	South Africa
SRR4034231	South Africa
SRR4034232	South Africa
SRR4034233	South Africa
SRR4034234	South Africa
SRR4034235	South Africa
SRR4034236	South Africa
SRR4034237	South Africa
SRR4034238	South Africa
SRR4034239	South Africa
SRR4034240	South Africa
SRR4034241	South Africa
SRR4034242	South Africa
SRR4034243	South Africa
SRR4034244	South Africa
SRR4034245	South Africa
SRR4034246	South Africa
SRR4034247	South Africa
SRR4034248	South Africa
SRR4034249	South Africa
SRR4034250	South Africa
SRR4034251	South Africa
SRR4034252	South Africa
SRR4034253	South Africa
SRR4034254	South Africa
SRR4034255	South Africa
SRR4034256	South Africa
SRR4034257	South Africa
SRR4034258	South Africa
SRR4034259	South Africa
SRR4034260	South Africa
SRR4034261	South Africa
SRR4034262	South Africa
SRR4034263	South Africa
SRR4034264	South Africa
SRR4034265	South Africa
SRR4034266	South Africa
SRR4034267	South Africa
SRR4034268	South Africa
SRR4034269	South Africa

SRR4034270	South Africa
SRR4034271	South Africa
SRR4034272	South Africa
SRR4034273	South Africa
SRR4034274	South Africa
SRR4034275	South Africa
SRR4034276	South Africa
SRR4034277	South Africa
SRR4034278	South Africa
SRR4034279	South Africa
SRR4034280	South Africa
SRR4034281	South Africa
SRR4034282	South Africa
SRR4034283	South Africa
SRR4034284	South Africa
SRR4034285	South Africa
SRR4034286	South Africa
SRR4034287	South Africa
SRR4034288	South Africa
SRR4034289	South Africa
SRR4034290	South Africa
SRR4034291	South Africa
SRR4034292	South Africa
SRR4034293	South Africa
SRR4034294	South Africa
SRR4034295	South Africa
SRR4034296	South Africa
SRR4034297	South Africa
SRR4034298	South Africa
SRR4034299	South Africa
SRR4034300	South Africa
SRR4034301	South Africa
SRR4034302	South Africa
SRR4034303	South Africa
SRR4034304	South Africa
SRR4034305	South Africa
SRR4034306	South Africa
SRR4034307	South Africa
SRR4034308	South Africa
SRR4034309	South Africa
SRR4034310	South Africa
SRR4034311	South Africa
SRR4034312	South Africa
SRR4034313	South Africa
SRR4034314	South Africa
SRR4034315	South Africa
SRR4034316	South Africa
SRR4034317	South Africa
SRR4034318	South Africa
SRR4034319	South Africa

SRR4034320	South Africa
SRR4034321	South Africa
SRR4034322	South Africa
SRR4034323	South Africa
SRR4034324	South Africa
SRR4034325	South Africa
SRR4034326	South Africa
SRR4034327	South Africa
SRR4034328	South Africa
SRR4034329	South Africa
SRR4034330	South Africa
SRR4034331	South Africa
SRR4034332	South Africa
SRR4034333	South Africa
SRR4034334	South Africa
SRR4034335	South Africa
SRR4034336	South Africa
SRR4034337	South Africa
SRR4034338	South Africa
SRR4034339	South Africa
SRR4034340	South Africa
SRR4034341	South Africa
SRR4034342	South Africa
SRR4034343	South Africa
SRR4034344	South Africa
SRR4034345	South Africa
SRR4034346	South Africa
SRR4034347	South Africa
SRR4034348	South Africa
SRR4034349	South Africa
SRR4034350	South Africa
SRR4034351	South Africa
SRR4034352	South Africa
SRR4034353	South Africa
SRR4034354	South Africa
SRR4034355	South Africa
SRR4034356	South Africa
SRR4034357	South Africa
SRR4034358	South Africa
SRR4034359	South Africa
SRR4034360	South Africa
SRR4034361	South Africa
SRR4034362	South Africa
SRR4034363	South Africa
SRR4034364	South Africa
SRR4034365	South Africa
SRR4034366	South Africa
SRR4034367	South Africa
SRR4034368	South Africa
SRR4034369	South Africa

SRR4034370	South Africa
SRR4034371	South Africa
SRR4034372	South Africa
SRR4034373	South Africa
SRR4034374	South Africa
SRR4034375	South Africa
SRR4034376	South Africa
SRR4034377	South Africa
SRR4034378	South Africa
SRR4034379	South Africa
SRR4034380	South Africa
SRR4034381	South Africa
SRR4034382	South Africa
SRR4034383	South Africa
SRR4034384	South Africa
SRR4034385	South Africa
SRR4034386	South Africa
SRR4034387	South Africa
SRR4034388	South Africa
SRR4034389	South Africa
SRR4034390	South Africa
SRR4034392	South Africa
SRR4034393	South Africa
SRR4034394	South Africa
SRR4034395	South Africa
SRR4034396	South Africa
SRR4034397	South Africa
SRR4034398	South Africa
SRR4034399	South Africa
SRR4034400	South Africa
SRR4034401	South Africa
SRR4034402	South Africa
SRR4034403	South Africa
SRR4034404	South Africa
SRR4034405	South Africa
SRR4034406	South Africa
SRR4034407	South Africa
SRR4034408	South Africa
SRR4034409	South Africa
SRR4034410	South Africa
SRR4034411	South Africa
SRR4034412	South Africa
SRR4034413	South Africa
SRR4034414	South Africa
SRR4034415	South Africa
SRR4034416	South Africa
SRR4034417	South Africa
SRR4034418	South Africa
SRR4034419	South Africa
SRR4034420	South Africa

SRR4034421	South Africa
SRR4034422	South Africa
SRR4034423	South Africa
SRR4034424	South Africa
SRR4034425	South Africa
SRR4034426	South Africa
SRR4034427	South Africa
SRR4034428	South Africa
SRR4034429	South Africa
SRR4034430	South Africa
SRR4034431	South Africa
SRR4034432	South Africa
SRR4034433	South Africa
SRR4034434	South Africa
SRR4034435	South Africa
SRR4034436	South Africa
SRR4034437	South Africa
SRR4034438	South Africa
SRR4034439	South Africa
SRR4034440	South Africa
SRR4034441	South Africa
SRR4034442	South Africa
SRR4034443	South Africa
SRR4034444	South Africa
SRR4034445	South Africa
SRR4034446	South Africa
SRR4034447	South Africa
SRR4034448	South Africa
SRR4034449	South Africa
SRR4034450	South Africa
SRR4034451	South Africa
SRR4034452	South Africa
SRR4034453	South Africa
SRR4034454	South Africa
SRR4034455	South Africa
SRR4034456	South Africa
SRR4034457	South Africa
SRR4034458	South Africa
SRR4034459	South Africa
SRR4034460	South Africa
SRR4034461	South Africa
SRR4034462	South Africa
SRR4034463	South Africa
SRR4034464	South Africa
SRR4034465	South Africa
SRR4034466	South Africa
SRR4034467	South Africa
SRR4034468	South Africa
SRR4034469	South Africa
SRR4034470	South Africa

SRR4034471	South Africa
SRR4034472	South Africa
SRR4034473	South Africa
SRR4034474	South Africa
SRR4034475	South Africa
SRR4034476	South Africa
SRR4034477	South Africa
SRR4034478	South Africa
SRR4034479	South Africa
SRR4034480	South Africa
SRR4034481	South Africa
SRR4034482	South Africa
SRR4034483	South Africa
SRR4034484	South Africa
SRR4034485	South Africa
SRR4034486	South Africa
SRR4034487	South Africa
SRR4034488	South Africa
SRR4034489	South Africa
SRR4034490	South Africa
SRR4034491	South Africa
SRR4034492	South Africa
SRR4034493	South Africa
SRR4034494	South Africa
SRR4034495	South Africa
SRR4034496	South Africa
SRR4034497	South Africa
SRR4034498	South Africa
SRR4034499	South Africa
SRR4034500	South Africa
SRR4034501	South Africa
SRR4034502	South Africa
SRR4034503	South Africa
SRR4034504	South Africa
SRR4034505	South Africa
SRR4034506	South Africa
SRR4034507	South Africa
SRR4034508	South Africa
SRR4034509	South Africa
SRR4034510	South Africa
SRR4034511	South Africa
SRR4034512	South Africa
SRR4034513	South Africa
SRR4034514	South Africa
SRR4034515	South Africa
SRR4034516	South Africa
SRR4034517	South Africa
SRR4034518	South Africa
SRR4034519	South Africa
SRR4034520	South Africa

SRR4034521	South Africa
SRR4034522	South Africa
SRR4034523	South Africa
SRR4034524	South Africa
SRR4034525	South Africa
SRR4034526	South Africa
SRR4034527	South Africa
SRR4034528	South Africa
SRR4034529	South Africa
SRR4034530	South Africa
SRR4034531	South Africa
SRR4034532	South Africa
SRR4034533	South Africa
SRR4034534	South Africa
SRR4034535	South Africa
SRR4034536	South Africa
SRR4034537	South Africa
SRR4034538	South Africa
SRR4034539	South Africa
SRR4034540	South Africa
SRR4034541	South Africa
SRR4034542	South Africa
SRR4034543	South Africa
SRR4034544	South Africa
SRR4034545	South Africa
SRR4034546	South Africa
SRR4034547	South Africa
SRR4034548	South Africa
SRR4034549	South Africa
SRR4034550	South Africa
SRR4034551	South Africa
SRR4034552	South Africa
SRR4034553	South Africa
SRR4034554	South Africa
SRR4034555	South Africa
SRR4034556	South Africa
SRR4034557	South Africa
SRR4034558	South Africa
SRR4034559	South Africa
SRR4034560	South Africa
SRR4034561	South Africa
SRR4034562	South Africa
SRR4034563	South Africa
SRR4034564	South Africa
SRR4034565	South Africa
SRR4034566	South Africa
SRR4034567	South Africa
SRR4034568	South Africa
SRR4034569	South Africa
SRR4034570	South Africa

SRR4034571	South Africa
SRR4034572	South Africa
SRR4034573	South Africa
SRR4034574	South Africa
SRR4034575	South Africa
SRR4034576	South Africa
SRR4034577	South Africa
SRR4034578	South Africa
SRR4034579	South Africa
SRR4034580	South Africa
SRR4034581	South Africa
SRR4034582	South Africa
SRR4034583	South Africa
SRR4034584	South Africa
SRR4034585	South Africa
SRR4034586	South Africa
SRR4034587	South Africa
SRR4034588	South Africa
SRR4034589	South Africa
SRR4034590	South Africa
SRR4034591	South Africa
SRR4034592	South Africa
SRR4034593	South Africa
SRR4034594	South Africa
SRR4034595	South Africa
SRR4034596	South Africa
SRR4034597	South Africa
SRR4034598	South Africa
SRR4034599	South Africa
SRR4034600	South Africa
SRR4034601	South Africa
SRR4034602	South Africa
SRR4034603	South Africa
SRR4034604	South Africa
SRR4034605	South Africa
SRR4034606	South Africa
SRR4034607	South Africa
SRR4034608	South Africa
SRR4034609	South Africa
SRR4034610	South Africa
SRR4034611	South Africa
SRR4034612	South Africa
SRR4034613	South Africa
SRR4034614	South Africa
SRR4034615	South Africa
SRR4034616	South Africa
SRR4034617	South Africa
SRR4034618	South Africa
SRR4034619	South Africa
SRR4034620	South Africa

SRR4034621	South Africa
SRR4034622	South Africa
SRR4034623	South Africa
SRR4034624	South Africa
SRR4034625	South Africa
SRR4034626	South Africa
SRR4034627	South Africa
SRR4034628	South Africa
SRR4034629	South Africa
SRR4034630	South Africa
SRR4034631	South Africa
SRR4034632	South Africa
SRR4034633	South Africa
SRR4034634	South Africa
SRR4034635	South Africa
SRR4034636	South Africa
SRR4034637	South Africa
SRR4034638	South Africa
SRR4034639	South Africa
SRR4034640	South Africa
SRR4034641	South Africa
SRR4034642	South Africa
SRR4034643	South Africa
SRR4034644	South Africa
SRR4034647	South Africa
SRR4034648	South Africa
SRR4034649	South Africa
SRR4034650	South Africa
SRR4034651	South Africa
SRR4034652	South Africa
SRR4034653	South Africa
SRR4034654	South Africa
SRR4034655	South Africa
SRR4034656	South Africa
SRR4034657	South Africa
SRR4034658	South Africa
SRR4034659	South Africa
SRR4034660	South Africa
SRR4034661	South Africa
SRR4034662	South Africa
SRR4034663	South Africa
SRR4034664	South Africa
SRR4034665	South Africa
SRR4034666	South Africa
SRR4034667	South Africa
SRR4034668	South Africa
SRR4034669	South Africa
SRR4034670	South Africa
SRR4034671	South Africa
SRR4034672	South Africa

SRR4034673	South Africa
SRR4034674	South Africa
SRR4034675	South Africa
SRR4034676	South Africa
SRR4034677	South Africa
SRR4034678	South Africa
SRR4034679	South Africa
SRR4034680	South Africa
SRR4034681	South Africa
SRR4034682	South Africa
SRR4034683	South Africa
SRR4034684	South Africa
SRR4034685	South Africa
SRR4034686	South Africa
SRR4034687	South Africa
SRR4034688	South Africa
SRR4034689	South Africa
SRR4034690	South Africa
SRR4034691	South Africa
SRR4034692	South Africa
SRR4034693	South Africa
SRR4034694	South Africa
SRR4034695	South Africa
SRR4034696	South Africa
SRR4034697	South Africa
SRR4034698	South Africa
SRR4034699	South Africa
SRR4034700	South Africa
SRR4034701	South Africa
SRR4034702	South Africa
SRR4034703	South Africa
SRR4034704	South Africa
SRR4034705	South Africa
SRR4034706	South Africa
SRR4034707	South Africa
SRR4034708	South Africa
SRR4034709	South Africa
SRR4034710	South Africa
SRR4034711	South Africa
SRR4034712	South Africa
SRR4034713	South Africa
SRR4034714	South Africa
SRR4034715	South Africa
SRR4034716	South Africa
SRR4034717	South Africa
SRR4034718	South Africa
SRR4034719	South Africa
SRR4034720	South Africa
SRR4034721	South Africa
SRR4034722	South Africa

SRR4034723	South Africa
SRR4034724	South Africa
SRR4034725	South Africa
SRR4034726	South Africa
SRR4034727	South Africa
SRR4034728	South Africa
SRR4034729	South Africa
SRR4034730	South Africa
SRR4034731	South Africa
SRR4034732	South Africa
SRR4034733	South Africa
SRR4034734	South Africa
SRR4034735	South Africa
SRR4034736	South Africa
SRR4034737	South Africa
SRR4034738	South Africa
SRR4034739	South Africa
SRR4034740	South Africa
SRR4034741	South Africa
SRR4034742	South Africa
SRR4034743	South Africa
SRR4034744	South Africa
SRR4034745	South Africa
SRR4034746	South Africa
SRR4034747	South Africa
SRR4034748	South Africa
SRR4034749	South Africa
SRR4034750	South Africa
SRR4034751	South Africa
SRR4034752	South Africa
SRR4034753	South Africa
SRR4034754	South Africa
SRR4034755	South Africa
SRR4034756	South Africa
SRR4034757	South Africa
SRR4034758	South Africa
SRR4034759	South Africa
SRR4034760	South Africa
SRR4034761	South Africa
SRR4034762	South Africa
SRR4034763	South Africa
SRR4034764	South Africa
SRR4034765	South Africa
SRR4034766	South Africa
SRR4034767	South Africa
SRR4034768	South Africa
SRR4034769	South Africa
SRR4034770	South Africa
SRR4034771	South Africa
SRR4034772	South Africa

SRR4034773	South Africa
SRR4034774	South Africa
SRR4034775	South Africa
SRR4034776	South Africa
SRR4034777	South Africa
SRR4034778	South Africa
SRR4034779	South Africa
SRR4034780	South Africa
SRR4034781	South Africa
SRR4034782	South Africa
SRR4034783	South Africa
SRR4034784	South Africa
SRR4034785	South Africa
SRR4034786	South Africa
SRR4034787	South Africa
SRR4034788	South Africa
SRR4034789	South Africa
SRR4034790	South Africa
SRR4034791	South Africa
SRR4034792	South Africa
SRR4034793	South Africa
SRR4034794	South Africa
SRR4034795	South Africa
SRR4034796	South Africa
SRR4034797	South Africa
SRR4034798	South Africa
SRR4034799	South Africa
SRR4034800	South Africa
SRR4034801	South Africa
SRR4034802	South Africa
SRR4034803	South Africa
SRR4034804	South Africa
SRR4034805	South Africa
SRR4034806	South Africa
SRR4034807	South Africa
SRR4034808	South Africa
SRR4034809	South Africa
SRR4034810	South Africa
SRR4034811	South Africa
SRR4034812	South Africa
SRR4034813	South Africa
SRR4034814	South Africa
SRR4034815	South Africa
SRR4034816	South Africa
SRR4034817	South Africa
SRR4034818	South Africa
SRR4034819	South Africa
SRR4034820	South Africa
SRR4034821	South Africa
SRR4034822	South Africa

SRR4034823	South Africa
SRR4034824	South Africa
SRR4034825	South Africa
SRR4034826	South Africa
SRR4034827	South Africa
SRR4034828	South Africa
SRR4034829	South Africa
SRR4034830	South Africa
SRR4034831	South Africa
SRR4034832	South Africa
SRR4034833	South Africa
SRR4034834	South Africa
SRR4034835	South Africa
SRR4034836	South Africa
SRR4034837	South Africa
SRR4034838	South Africa
SRR4034839	South Africa
SRR4034840	South Africa
SRR4034841	South Africa
SRR4034842	South Africa
SRR4034843	South Africa
SRR4034844	South Africa
SRR4035486	South Africa
SRR4035487	South Africa
SRR4035488	South Africa
SRR4035489	South Africa
SRR4035490	South Africa
SRR4035491	South Africa
SRR4035492	South Africa
SRR4035493	South Africa
SRR4035494	South Africa
SRR4035495	South Africa
SRR4035496	South Africa
SRR4035497	South Africa
SRR4035498	South Africa
SRR4035499	South Africa
SRR4035500	South Africa
SRR4035501	South Africa
SRR4035502	South Africa
SRR4035503	South Africa
SRR4035504	South Africa
SRR4035505	South Africa
SRR4035506	South Africa
SRR4035507	South Africa
SRR4035508	South Africa
SRR4035509	South Africa
SRR4035510	South Africa
SRR4035511	South Africa
SRR4035512	South Africa
SRR4035513	South Africa

SRR4035514	South Africa
SRR4035515	South Africa
SRR4035516	South Africa
SRR4035517	South Africa
SRR4035518	South Africa
SRR4035519	South Africa
SRR4035520	South Africa
SRR4035521	South Africa
SRR4035522	South Africa
SRR4035523	South Africa
SRR4035524	South Africa
SRR4035525	South Africa
SRR4035526	South Africa
SRR4035527	South Africa
SRR4035528	South Africa
SRR4035529	South Africa
SRR4035530	South Africa
SRR4035531	South Africa
SRR4035532	South Africa
SRR4035533	South Africa
SRR4035534	South Africa
SRR4035535	South Africa
SRR4035536	South Africa
SRR4035537	South Africa
SRR4035538	South Africa
SRR4035539	South Africa
SRR4035540	South Africa
SRR4035541	South Africa
SRR4035542	South Africa
SRR4035543	South Africa
SRR4035544	South Africa
SRR4035545	South Africa
SRR4035546	South Africa
SRR4035547	South Africa
SRR4035548	South Africa
SRR4035549	South Africa
SRR4035550	South Africa
SRR4035551	South Africa
SRR4035552	South Africa
SRR4035553	South Africa
SRR4035554	South Africa
SRR4035555	South Africa
SRR4035556	South Africa
SRR4035557	South Africa
SRR4035558	South Africa
SRR4035559	South Africa
SRR4035560	South Africa
SRR4035561	South Africa
SRR4035562	South Africa
SRR4035563	South Africa

SRR4035564	South Africa
SRR4035565	South Africa
SRR4035566	South Africa
SRR4035567	South Africa
SRR4035568	South Africa
SRR4035569	South Africa
SRR4035570	South Africa
SRR4035571	South Africa
SRR4035572	South Africa
SRR4035573	South Africa
SRR4035574	South Africa
SRR4035575	South Africa
SRR4035576	South Africa
SRR4035577	South Africa
SRR4035578	South Africa
SRR4035579	South Africa
SRR4035580	South Africa
SRR4035581	South Africa
SRR4035582	South Africa
SRR4035583	South Africa
SRR4035584	South Africa
SRR4035585	South Africa
SRR4035586	South Africa
SRR4035587	South Africa
SRR4035588	South Africa
SRR4035589	South Africa
SRR4035590	South Africa
SRR4035591	South Africa
SRR4035592	South Africa
SRR4035593	South Africa
SRR4035594	South Africa
SRR4035595	South Africa
SRR4035596	South Africa
SRR4035597	South Africa
SRR4035598	South Africa
SRR4035599	South Africa
SRR4035600	South Africa
SRR4035601	South Africa
SRR4035602	South Africa
SRR4035603	South Africa
SRR4035604	South Africa
SRR4035605	South Africa
SRR4035606	South Africa
SRR4035607	South Africa
SRR4035608	South Africa
SRR4035609	South Africa
SRR4035610	South Africa
SRR4035611	South Africa
SRR4035612	South Africa
SRR4035613	South Africa

SRR4035614	South Africa
SRR4035615	South Africa
SRR4035616	South Africa
SRR4035617	South Africa
SRR4035618	South Africa
SRR4035619	South Africa
SRR4035620	South Africa
SRR4035621	South Africa
SRR4035622	South Africa
SRR4035623	South Africa
SRR4035624	South Africa
SRR4035625	South Africa
SRR4035626	South Africa
SRR4035627	South Africa
SRR4035628	South Africa
SRR4035629	South Africa
SRR4035630	South Africa
SRR4035631	South Africa
SRR4035650	South Africa
SRR4035651	South Africa
SRR4035652	South Africa
SRR4035653	South Africa
SRR4035654	South Africa
SRR4035655	South Africa
SRR4035656	South Africa
SRR4035657	South Africa
SRR4035658	South Africa
SRR4035659	South Africa
SRR4035660	South Africa
SRR4035661	South Africa
SRR4035662	South Africa
SRR4035663	South Africa
SRR4035664	South Africa
SRR4035665	South Africa
SRR4035666	South Africa
SRR4035667	South Africa
SRR4035668	South Africa
SRR4035669	South Africa
SRR4035670	South Africa
SRR4035671	South Africa
SRR4035672	South Africa
SRR4035673	South Africa
SRR4035683	South Africa
SRR4035701	South Africa
SRR4035702	South Africa
SRR4035703	South Africa
SRR4035704	South Africa
SRR4035705	South Africa
SRR4035706	South Africa
SRR4035707	South Africa

SRR4035709	South Africa
SRR4035710	South Africa
SRR4035711	South Africa
SRR4035712	South Africa
SRR4035713	South Africa
SRR4035714	South Africa
SRR4035715	South Africa
SRR4035716	South Africa
SRR4035717	South Africa
SRR4035718	South Africa
SRR4035719	South Africa
SRR4035720	South Africa
SRR4035721	South Africa
SRR4035722	South Africa
SRR4035723	South Africa
SRR4035724	South Africa
SRR4035725	South Africa
SRR4035726	South Africa
SRR4035727	South Africa
SRR4035728	South Africa
SRR4035729	South Africa
SRR4035730	South Africa
SRR4035731	South Africa
SRR4035732	South Africa
SRR4035733	South Africa
SRR4035734	South Africa
SRR4035735	South Africa
SRR4035736	South Africa
SRR4035737	South Africa
SRR4035738	South Africa
SRR4035739	South Africa
SRR4035740	South Africa
SRR4035741	South Africa
SRR4035742	South Africa
SRR4035743	South Africa
SRR4035744	South Africa
SRR4035745	South Africa
SRR4035746	South Africa
SRR4035747	South Africa
SRR4035748	South Africa
SRR4035749	South Africa
SRR4035750	South Africa
SRR4035751	South Africa
SRR4035752	South Africa
SRR4035753	South Africa
SRR4035754	South Africa
SRR4035755	South Africa
SRR4035756	South Africa
SRR4035757	South Africa
SRR4035758	South Africa

SRR4035759	South Africa
SRR4035760	South Africa
SRR4035761	South Africa
SRR4035762	South Africa
SRR4035763	South Africa
SRR4035764	South Africa
SRR4035765	South Africa
SRR4035766	South Africa
SRR4035767	South Africa
SRR4035768	South Africa
SRR4035769	South Africa
SRR4035770	South Africa
SRR4035771	South Africa
SRR4035772	South Africa
SRR4035773	South Africa
SRR4035775	South Africa
SRR4035776	South Africa
SRR4035777	South Africa
SRR4035778	South Africa
SRR4035779	South Africa
SRR4035780	South Africa
SRR4035781	South Africa
SRR4035782	South Africa
SRR4035783	South Africa
SRR4035785	South Africa
SRR4035786	South Africa
SRR4035787	South Africa
SRR4035788	South Africa
SRR4035789	South Africa
SRR4035790	South Africa
SRR4035791	South Africa
SRR4035792	South Africa
SRR4035793	South Africa
SRR4035794	South Africa
SRR4035795	South Africa
SRR4035796	South Africa
SRR4035797	South Africa
SRR4035798	South Africa
SRR4035799	South Africa
SRR4035800	South Africa
SRR4035801	South Africa
SRR4035802	South Africa
SRR4035803	South Africa
SRR4035804	South Africa
SRR4035805	South Africa
SRR4035806	South Africa
SRR4035807	South Africa
SRR4035808	South Africa
SRR4035809	South Africa
SRR4035810	South Africa

SRR4035811	South Africa
SRR4035812	South Africa
SRR4035813	South Africa
SRR4035814	South Africa
SRR4035815	South Africa
SRR4035816	South Africa
SRR4035817	South Africa
SRR4035818	South Africa
SRR4035819	South Africa
SRR4035820	South Africa
SRR4035821	South Africa
SRR4035822	South Africa
SRR4035823	South Africa
SRR4035824	South Africa
SRR4035825	South Africa
SRR4035826	South Africa
SRR4035827	South Africa
SRR4035828	South Africa
SRR4035829	South Africa
SRR4035830	South Africa
SRR4035831	South Africa
SRR4035832	South Africa
SRR4035833	South Africa
SRR4035834	South Africa
SRR4035835	South Africa
SRR4035836	South Africa
SRR4035837	South Africa
SRR4035838	South Africa
SRR4035839	South Africa
SRR4035840	South Africa
SRR4035841	South Africa
SRR4035842	South Africa
SRR4035843	South Africa
SRR4035844	South Africa
SRR4035845	South Africa
SRR4035846	South Africa
SRR4035847	South Africa
SRR4035848	South Africa
SRR4035849	South Africa
SRR4035850	South Africa
SRR4035851	South Africa
SRR4035852	South Africa
SRR4035853	South Africa
SRR4035854	South Africa
SRR4035855	South Africa
SRR4035856	South Africa
SRR4035857	South Africa
SRR4035858	South Africa
SRR4035859	South Africa
SRR4035860	South Africa

SRR4035861	South Africa
SRR4035862	South Africa
SRR4035863	South Africa
SRR4035864	South Africa
SRR4035865	South Africa
SRR4035866	South Africa
SRR4035867	South Africa
SRR4035868	South Africa
SRR4035869	South Africa
SRR4035870	South Africa
SRR4035871	South Africa
SRR4035872	South Africa
SRR4035873	South Africa
SRR4035874	South Africa
SRR4035875	South Africa
SRR4035876	South Africa
SRR4035877	South Africa
SRR4035878	South Africa
SRR4035879	South Africa
SRR4035880	South Africa
SRR4035881	South Africa
SRR4035882	South Africa
SRR4035883	South Africa
SRR4035884	South Africa
SRR4035885	South Africa
SRR4035886	South Africa
SRR4035887	South Africa
SRR4035888	South Africa
SRR4035889	South Africa
SRR4035890	South Africa
SRR4035891	South Africa
SRR4035892	South Africa
SRR4035893	South Africa
SRR4035894	South Africa
SRR4035895	South Africa
SRR4035896	South Africa
SRR4035897	South Africa
SRR4035898	South Africa
SRR4035899	South Africa
SRR4035900	South Africa
SRR4035901	South Africa
SRR4035902	South Africa
SRR4035903	South Africa
SRR4035904	South Africa
SRR4035905	South Africa
SRR4035906	South Africa
SRR4035907	South Africa
SRR4035908	South Africa
SRR4035909	South Africa
SRR4035910	South Africa

SRR4035911	South Africa
SRR4035912	South Africa
SRR4035913	South Africa
SRR4035914	South Africa
SRR4035915	South Africa
SRR4035916	South Africa
SRR4035917	South Africa
SRR4035918	South Africa
SRR4035919	South Africa
SRR4035920	South Africa
SRR4035921	South Africa
SRR4035922	South Africa
SRR4035923	South Africa
SRR4035924	South Africa
SRR4035925	South Africa
SRR4035926	South Africa
SRR4035927	South Africa
SRR4035928	South Africa
SRR4035929	South Africa
SRR4035930	South Africa
SRR4035931	South Africa
SRR4035932	South Africa
SRR4035933	South Africa
SRR4035934	South Africa
SRR4035935	South Africa
SRR4035936	South Africa
SRR4035937	South Africa
SRR4035938	South Africa
SRR4035939	South Africa
SRR4035940	South Africa
SRR4035941	South Africa
SRR4035942	South Africa
SRR4035943	South Africa
SRR4035944	South Africa
SRR4035945	South Africa
SRR4035946	South Africa
SRR4035947	South Africa
SRR4035948	South Africa
SRR4035949	South Africa
SRR4035950	South Africa
SRR4035951	South Africa
SRR4035952	South Africa
SRR4035953	South Africa
SRR4035954	South Africa
SRR4035955	South Africa
SRR4035956	South Africa
SRR4035957	South Africa
SRR4035958	South Africa
SRR4035959	South Africa
SRR4035960	South Africa

SRR4035961	South Africa
SRR4035962	South Africa
SRR4035963	South Africa
SRR4035964	South Africa
SRR4035965	South Africa
SRR4035966	South Africa
SRR4035967	South Africa
SRR4035968	South Africa
SRR4035969	South Africa
SRR4035970	South Africa
SRR4035971	South Africa
SRR4035972	South Africa
SRR4035973	South Africa
SRR4035974	South Africa
SRR4035975	South Africa
SRR4035976	South Africa
SRR4035977	South Africa
SRR4035978	South Africa
SRR4035979	South Africa
SRR4035980	South Africa
SRR4035981	South Africa
SRR4035982	South Africa
SRR4035983	South Africa
SRR4035984	South Africa
SRR4035985	South Africa
SRR4035986	South Africa
SRR4035987	South Africa
SRR4035988	South Africa
SRR4035989	South Africa
SRR4035990	South Africa
SRR4035991	South Africa
SRR4035992	South Africa
SRR4035993	South Africa
SRR4035994	South Africa
SRR4035995	South Africa
SRR4035996	South Africa
SRR4035997	South Africa
SRR4035998	South Africa
SRR4035999	South Africa
SRR4036000	South Africa
SRR4036001	South Africa
SRR4036002	South Africa
SRR4036003	South Africa
SRR4036004	South Africa
SRR4036005	South Africa
SRR4036006	South Africa
SRR4036007	South Africa
SRR4036008	South Africa
SRR4036009	South Africa
SRR4036010	South Africa

SRR4036011	South Africa
SRR4036012	South Africa
SRR4036013	South Africa
SRR4036014	South Africa
SRR4036015	South Africa
SRR4036016	South Africa
SRR4036017	South Africa
SRR4036018	South Africa
SRR4036019	South Africa
SRR4036020	South Africa
SRR4036021	South Africa
SRR4036022	South Africa
SRR4036023	South Africa
SRR4036024	South Africa
SRR4036025	South Africa
SRR4036026	South Africa
SRR4036027	South Africa
SRR4036028	South Africa
SRR4037485	South Africa
SRR4037486	South Africa
SRR4037487	South Africa
SRR4037488	South Africa
SRR4037489	South Africa
SRR4037490	South Africa
SRR4037491	South Africa
SRR4037492	South Africa
SRR4037493	South Africa
SRR4037494	South Africa
SRR4037495	South Africa
SRR4037496	South Africa
SRR4037497	South Africa
SRR4037498	South Africa
SRR4037499	South Africa
SRR4037500	South Africa
SRR4037501	South Africa
SRR4037502	South Africa
SRR4037503	South Africa
SRR4037504	South Africa
SRR4037505	South Africa
SRR4037506	South Africa
SRR4037507	South Africa
SRR4037508	South Africa
SRR4037509	South Africa
SRR4037510	South Africa
SRR4037511	South Africa
SRR4037512	South Africa
SRR4037513	South Africa
SRR4037514	South Africa
SRR4037515	South Africa
SRR4037516	South Africa

SRR4037517	South Africa
SRR4037518	South Africa
SRR4037519	South Africa
SRR4037520	South Africa
SRR4037521	South Africa
SRR4037522	South Africa
SRR4037523	South Africa
SRR4037524	South Africa
SRR4037525	South Africa
SRR4037526	South Africa
SRR4037527	South Africa
SRR4037528	South Africa
SRR4037529	South Africa
SRR4037530	South Africa
SRR4037531	South Africa
SRR4037532	South Africa
SRR4037533	South Africa
SRR4037534	South Africa
SRR4037535	South Africa
SRR4037536	South Africa
SRR4037537	South Africa
SRR4037538	South Africa
SRR4037539	South Africa
SRR4037540	South Africa
SRR4037541	South Africa
SRR4037542	South Africa
SRR4037543	South Africa
SRR4037544	South Africa
SRR4037545	South Africa
SRR4037546	South Africa
SRR4037547	South Africa
SRR4037548	South Africa
SRR4037549	South Africa
SRR4037550	South Africa
SRR4037551	South Africa
SRR4037552	South Africa
SRR4037553	South Africa
SRR4037554	South Africa
SRR4037555	South Africa
SRR4037556	South Africa
SRR4037557	South Africa
SRR4037558	South Africa
SRR4037559	South Africa
SRR4037560	South Africa
SRR4037561	South Africa
SRR4037562	South Africa
SRR4037563	South Africa
SRR4037564	South Africa
SRR4037565	South Africa
SRR4037566	South Africa

SRR4037567	South Africa
SRR4037568	South Africa
SRR4037569	South Africa
SRR4037570	South Africa
SRR4037571	South Africa
SRR4037572	South Africa
SRR4037573	South Africa
SRR4037574	South Africa
SRR4037575	South Africa
SRR4037576	South Africa
SRR4037577	South Africa
SRR4037578	South Africa
SRR4037579	South Africa
SRR4037580	South Africa
SRR4037581	South Africa
SRR4037582	South Africa
SRR4037583	South Africa
SRR4037584	South Africa
SRR4037585	South Africa
SRR4037586	South Africa
SRR4037587	South Africa
SRR4037588	South Africa
SRR4037589	South Africa
SRR4037590	South Africa
SRR4037591	South Africa
SRR4037592	South Africa
SRR4037593	South Africa
SRR4037594	South Africa
SRR4037595	South Africa
SRR4037596	South Africa
SRR4037597	South Africa
SRR4037598	South Africa
SRR4037599	South Africa
SRR4037600	South Africa
SRR4037601	South Africa
SRR4037602	South Africa
SRR4037603	South Africa
SRR4037604	South Africa
SRR4037605	South Africa
SRR4037606	South Africa
SRR4037607	South Africa
SRR4037608	South Africa
SRR4037609	South Africa
SRR4037610	South Africa
SRR4037611	South Africa
SRR4037612	South Africa
SRR4037613	South Africa
SRR4037614	South Africa
SRR4037615	South Africa
SRR4037616	South Africa

SRR4037617	South Africa
SRR4037618	South Africa
SRR4037619	South Africa
SRR4037620	South Africa
SRR4037621	South Africa
SRR4037622	South Africa
SRR4037623	South Africa
SRR4037624	South Africa
SRR4037625	South Africa
SRR4037626	South Africa
SRR4037627	South Africa
SRR4037628	South Africa
SRR4037629	South Africa
SRR4037630	South Africa
SRR4037631	South Africa
SRR4037632	South Africa
SRR4037633	South Africa
SRR4037634	South Africa
SRR4037635	South Africa
SRR4037636	South Africa
SRR4037637	South Africa
SRR4037638	South Africa
SRR4037639	South Africa
SRR4037640	South Africa
SRR4037641	South Africa
SRR4037642	South Africa
SRR4037643	South Africa
SRR4037644	South Africa
SRR4037645	South Africa
SRR4037646	South Africa
SRR4037647	South Africa
SRR4037648	South Africa
SRR4037649	South Africa
SRR4037650	South Africa
SRR4037651	South Africa
SRR4037652	South Africa
SRR4037653	South Africa
SRR4037654	South Africa
SRR4037655	South Africa
SRR4037656	South Africa
SRR4037657	South Africa
SRR4037658	South Africa
SRR4037659	South Africa
SRR4037660	South Africa
SRR4037661	South Africa
SRR4037662	South Africa
SRR4037663	South Africa
SRR4037664	South Africa
SRR4037665	South Africa
SRR4037666	South Africa

SRR4037667	South Africa
SRR4037668	South Africa
SRR4037669	South Africa
SRR4037670	South Africa
SRR4037671	South Africa
SRR4037672	South Africa
SRR4037673	South Africa
SRR4037674	South Africa
SRR4037675	South Africa
SRR4037676	South Africa
SRR4037677	South Africa
SRR4037678	South Africa
SRR4037679	South Africa
SRR4037680	South Africa
SRR4037681	South Africa
SRR4037682	South Africa
SRR4037683	South Africa
SRR4037684	South Africa
SRR4037685	South Africa
SRR4037686	South Africa
SRR4037687	South Africa
SRR4037688	South Africa
SRR4037689	South Africa
SRR4037690	South Africa
SRR4037691	South Africa
SRR4037692	South Africa
SRR4037693	South Africa
SRR4037694	South Africa
SRR4037695	South Africa
SRR4037696	South Africa
SRR4037697	South Africa
SRR4037698	South Africa
SRR4037699	South Africa
SRR4037700	South Africa
SRR4037701	South Africa
SRR4037702	South Africa
SRR4037703	South Africa
SRR4037704	South Africa
SRR4037705	South Africa
SRR4037706	South Africa
SRR4037707	South Africa
SRR4037708	South Africa
SRR4037709	South Africa
SRR4037710	South Africa
SRR4037711	South Africa
SRR4037712	South Africa
SRR4037713	South Africa
SRR4037714	South Africa
SRR4037715	South Africa
SRR4037716	South Africa

SRR4037717	South Africa
SRR4037718	South Africa
SRR4037719	South Africa
SRR4037720	South Africa
SRR4037721	South Africa
SRR4037722	South Africa
SRR4037723	South Africa
SRR4037724	South Africa
SRR4037725	South Africa
SRR4037726	South Africa
SRR4037727	South Africa
SRR4037728	South Africa
SRR4037729	South Africa
SRR4037730	South Africa
SRR4037731	South Africa
SRR4037732	South Africa
SRR4037733	South Africa
SRR4037734	South Africa
SRR4037735	South Africa
SRR4037736	South Africa
SRR4037737	South Africa
SRR4037738	South Africa
SRR4037739	South Africa
SRR4037740	South Africa
SRR4037741	South Africa
SRR4037742	South Africa
SRR4037743	South Africa
SRR4037744	South Africa
SRR4037746	South Africa
SRR4037747	South Africa
SRR4037748	South Africa
SRR4037749	South Africa
SRR4037750	South Africa
SRR4037751	South Africa
SRR4037753	South Africa
SRR4037754	South Africa
SRR4037755	South Africa
SRR4037756	South Africa
SRR4037757	South Africa
SRR4037758	South Africa
SRR4037759	South Africa
SRR4037760	South Africa
SRR4037761	South Africa
SRR4037762	South Africa
SRR4037763	South Africa
SRR4037764	South Africa
SRR4037765	South Africa
SRR4037766	South Africa
SRR4037767	South Africa
SRR4037768	South Africa

SRR4037769	South Africa
SRR4037770	South Africa
SRR4037771	South Africa
SRR4037772	South Africa
SRR4037773	South Africa
SRR4037774	South Africa
SRR4037775	South Africa
SRR4037776	South Africa
SRR4037778	South Africa
SRR4037779	South Africa
SRR4037780	South Africa
SRR4037781	South Africa
SRR4037782	South Africa
SRR4037783	South Africa
SRR4037784	South Africa
SRR4037785	South Africa
SRR4037786	South Africa
SRR4037787	South Africa
SRR4037788	South Africa
SRR4037789	South Africa
SRR4037790	South Africa
SRR4037791	South Africa
SRR4037792	South Africa
SRR4037793	South Africa
SRR4037794	South Africa
SRR4037795	South Africa
SRR4037796	South Africa
SRR4037797	South Africa
SRR4037798	South Africa
SRR4037799	South Africa
SRR4037800	South Africa
SRR4037801	South Africa
SRR4037802	South Africa
SRR4037803	South Africa
SRR4037804	South Africa
SRR4037805	South Africa
SRR4037806	South Africa
SRR4037807	South Africa
SRR4037808	South Africa
SRR4037809	South Africa
SRR4037810	South Africa
SRR4037811	South Africa
SRR4037812	South Africa
SRR4037813	South Africa
SRR4037814	South Africa
SRR4037815	South Africa
SRR4037816	South Africa
SRR4037817	South Africa
SRR4037818	South Africa
SRR4037819	South Africa

SRR4037820	South Africa
SRR4037821	South Africa
SRR4037822	South Africa
SRR4037823	South Africa
SRR4037824	South Africa
SRR4037825	South Africa
SRR4037826	South Africa
SRR4037827	South Africa
SRR4037828	South Africa
SRR4037829	South Africa
SRR4037830	South Africa
SRR4037831	South Africa
SRR4037832	South Africa
SRR4037833	South Africa
SRR4037834	South Africa
SRR4037835	South Africa
SRR4037836	South Africa
SRR4037837	South Africa
SRR4037838	South Africa
SRR4037839	South Africa
SRR4037840	South Africa
SRR4037841	South Africa
SRR4037842	South Africa
SRR4037843	South Africa
SRR4037844	South Africa
SRR4037845	South Africa
SRR4037846	South Africa
SRR4037847	South Africa
SRR4037848	South Africa
SRR4037849	South Africa
SRR4037850	South Africa
SRR4037851	South Africa
SRR4037852	South Africa
SRR4037853	South Africa
SRR4037854	South Africa
SRR4037855	South Africa
SRR4037856	South Africa
SRR4037857	South Africa
SRR4037858	South Africa
SRR4037859	South Africa
SRR4037860	South Africa
SRR4037861	South Africa
SRR4037862	South Africa
SRR4037863	South Africa
SRR4037864	South Africa
SRR4037865	South Africa
SRR4037866	South Africa
SRR4037867	South Africa
SRR4037868	South Africa
SRR4037869	South Africa

SRR4037870	South Africa
SRR4037871	South Africa
SRR4037872	South Africa
SRR4037873	South Africa
SRR4037874	South Africa
SRR4037875	South Africa
SRR4037877	South Africa
SRR4037878	South Africa
SRR4037879	South Africa
SRR4037880	South Africa
SRR4037881	South Africa
SRR4037882	South Africa
SRR4037883	South Africa
SRR4037884	South Africa
SRR4037885	South Africa
SRR4037886	South Africa
SRR4037887	South Africa
SRR4037888	South Africa
SRR4037889	South Africa
SRR4037890	South Africa
SRR4037891	South Africa
SRR4037892	South Africa
SRR4037893	South Africa
SRR4037894	South Africa
SRR4037895	South Africa
SRR4037896	South Africa
SRR4037897	South Africa
SRR4037898	South Africa
SRR4037899	South Africa
SRR4037900	South Africa
SRR4037902	South Africa
SRR4037903	South Africa
SRR4037904	South Africa
SRR4037905	South Africa
SRR4037906	South Africa
SRR4037907	South Africa
SRR4037908	South Africa
SRR4037909	South Africa
SRR4037910	South Africa
SRR4037911	South Africa
SRR4037912	South Africa
SRR4037913	South Africa
SRR4037914	South Africa
SRR4037915	South Africa
SRR4037916	South Africa
SRR4037917	South Africa
SRR4037918	South Africa
SRR4037919	South Africa
SRR4037920	South Africa
SRR4037921	South Africa

SRR4037922	South Africa
SRR4037923	South Africa
SRR4037924	South Africa
SRR4037925	South Africa
SRR4037926	South Africa
SRR4037927	South Africa
SRR4037928	South Africa
SRR4037929	South Africa
SRR4037930	South Africa
SRR4037931	South Africa
SRR4037932	South Africa
SRR4037933	South Africa
SRR4037934	South Africa
SRR4037935	South Africa
SRR4037936	South Africa
SRR4037937	South Africa
SRR4037938	South Africa
SRR4037939	South Africa
SRR4037940	South Africa
SRR4037941	South Africa
SRR4037942	South Africa
SRR4037943	South Africa
SRR4037944	South Africa
SRR4037945	South Africa
SRR4037946	South Africa
SRR4037947	South Africa
SRR4037948	South Africa
SRR4037949	South Africa
SRR4037950	South Africa
SRR4037951	South Africa
SRR4037952	South Africa
SRR4037953	South Africa
SRR4037954	South Africa
SRR4037955	South Africa
SRR4037956	South Africa
SRR4037957	South Africa
SRR4037958	South Africa
SRR4037960	South Africa
SRR4037961	South Africa
SRR4037962	South Africa
SRR4037963	South Africa
SRR4037964	South Africa
SRR4037965	South Africa
SRR4037966	South Africa
SRR4037967	South Africa
SRR4037968	South Africa
SRR4037969	South Africa
SRR4037970	South Africa
SRR4037971	South Africa
SRR4037972	South Africa

SRR4037973	South Africa
SRR4037974	South Africa
SRR4037975	South Africa
SRR4037976	South Africa
SRR4037977	South Africa
SRR4037978	South Africa
SRR4037979	South Africa
SRR4037980	South Africa
SRR4037981	South Africa
SRR4037982	South Africa
SRR4037983	South Africa
SRR4037984	South Africa
SRR4114384	NA
SRR4114385	NA
SRR4114386	NA
SRR4114387	NA
SRR4114388	NA
SRR4114390	NA
SRR4114391	NA
SRR4114392	NA
SRR413209	NA
SRR413210	NA
SRR413211	NA
SRR413213	NA
SRR413214	NA
SRR413216	NA
SRR413217	NA
SRR413218	NA
SRR413219	NA
SRR413221	NA
SRR413222	NA
SRR413224	NA
SRR413226	NA
SRR413227	NA
SRR413228	NA
SRR413229	NA
SRR413231	NA
SRR413232	NA
SRR413266	NA
SRR4190350	NA
SRR4423135	Bangladesh
SRR4423136	Bangladesh
SRR4423137	Bangladesh
SRR4423138	Bangladesh
SRR4423139	Bangladesh
SRR4423140	Bangladesh
SRR4423141	Bangladesh
SRR4423142	Bangladesh
SRR4423143	Bangladesh
SRR4423144	Bangladesh

SRR4423145	Bangladesh
SRR4423146	Bangladesh
SRR4423147	Bangladesh
SRR4423148	Bangladesh
SRR4423149	Bangladesh
SRR4423150	Bangladesh
SRR4423151	Bangladesh
SRR4423152	Bangladesh
SRR4423153	Bangladesh
SRR4423154	Bangladesh
SRR4423155	Bangladesh
SRR4423156	Bangladesh
SRR4423157	Bangladesh
SRR4423158	Bangladesh
SRR4423159	Bangladesh
SRR4423160	Bangladesh
SRR4423161	Bangladesh
SRR4423162	Bangladesh
SRR4423163	Bangladesh
SRR4423164	Bangladesh
SRR4423165	Bangladesh
SRR4423166	Bangladesh
SRR4423167	Bangladesh
SRR4423168	Bangladesh
SRR4423169	Bangladesh
SRR4423170	Bangladesh
SRR4423171	Bangladesh
SRR4423172	Bangladesh
SRR4423173	Bangladesh
SRR4423174	Bangladesh
SRR4423175	Bangladesh
SRR4423176	Bangladesh
SRR4423177	Bangladesh
SRR4423178	Bangladesh
SRR4423179	Bangladesh
SRR4423180	Bangladesh
SRR4423181	Bangladesh
SRR475257	NA
SRR5007155	United Kingdom
SRR5007156	United Kingdom
SRR5007157	United Kingdom
SRR5007158	United Kingdom
SRR5007159	United Kingdom
SRR5007160	United Kingdom
SRR5007161	United Kingdom
SRR5007162	United Kingdom
SRR5007163	United Kingdom
SRR5007164	United Kingdom
SRR5007165	United Kingdom
SRR5007166	United Kingdom

SRR5007167	United Kingdom
SRR5007168	United Kingdom
SRR5007169	United Kingdom
SRR5007170	United Kingdom
SRR5007171	United Kingdom
SRR5007172	United Kingdom
SRR5007173	United Kingdom
SRR5007174	United Kingdom
SRR5007175	United Kingdom
SRR5007176	United Kingdom
SRR5007177	United Kingdom
SRR5007178	United Kingdom
SRR5007179	United Kingdom
SRR5007180	United Kingdom
SRR5007181	United Kingdom
SRR5007182	United Kingdom
SRR5007183	United Kingdom
SRR5007184	United Kingdom
SRR5007185	United Kingdom
SRR5007186	United Kingdom
SRR5007187	United Kingdom
SRR5007188	United Kingdom
SRR5007189	United Kingdom
SRR5007190	United Kingdom
SRR5007191	United Kingdom
SRR5007192	United Kingdom
SRR5007193	United Kingdom
SRR5007195	United Kingdom
SRR5007196	United Kingdom
SRR5007197	United Kingdom
SRR5007198	United Kingdom
SRR5007199	United Kingdom
SRR5007200	United Kingdom
SRR5007201	United Kingdom
SRR5007202	United Kingdom
SRR5007203	United Kingdom
SRR5007204	United Kingdom
SRR5007205	United Kingdom
SRR5065200	Vietnam
SRR5065201	Vietnam
SRR5065202	Vietnam
SRR5065203	Vietnam
SRR5065204	Vietnam
SRR5065205	Vietnam
SRR5065206	Vietnam
SRR5065207	Vietnam
SRR5065208	Vietnam
SRR5065209	Vietnam
SRR5065210	Vietnam
SRR5065211	Vietnam

SRR5065212	Vietnam
SRR5065213	Vietnam
SRR5065214	Vietnam
SRR5065215	Vietnam
SRR5065216	Vietnam
SRR5065217	Vietnam
SRR5065218	Vietnam
SRR5065219	Vietnam
SRR5065220	Vietnam
SRR5065221	Vietnam
SRR5065222	Vietnam
SRR5065223	Vietnam
SRR5065224	Vietnam
SRR5065225	Vietnam
SRR5065226	Vietnam
SRR5065227	Vietnam
SRR5065228	Vietnam
SRR5065229	Vietnam
SRR5065230	Vietnam
SRR5065231	Vietnam
SRR5065232	Vietnam
SRR5065233	Vietnam
SRR5065234	Vietnam
SRR5065235	Vietnam
SRR5065236	Vietnam
SRR5065237	Vietnam
SRR5065238	Vietnam
SRR5065239	Vietnam
SRR5065240	Vietnam
SRR5065241	Vietnam
SRR5065242	Vietnam
SRR5065243	Vietnam
SRR5065244	Vietnam
SRR5065245	Vietnam
SRR5065246	Vietnam
SRR5065247	Vietnam
SRR5065248	Vietnam
SRR5065249	Vietnam
SRR5065250	Vietnam
SRR5065251	Vietnam
SRR5065252	Vietnam
SRR5065253	Vietnam
SRR5065254	Vietnam
SRR5065255	Vietnam
SRR5065256	Vietnam
SRR5065257	Vietnam
SRR5065258	Vietnam
SRR5065259	Vietnam
SRR5065260	Vietnam
SRR5065261	Vietnam

SRR5065262	Vietnam
SRR5065263	Vietnam
SRR5065264	Vietnam
SRR5065265	Vietnam
SRR5065266	Vietnam
SRR5065267	Vietnam
SRR5065268	Vietnam
SRR5065269	Vietnam
SRR5065270	Vietnam
SRR5065271	Vietnam
SRR5065272	Vietnam
SRR5065273	Vietnam
SRR5065274	Vietnam
SRR5065275	Vietnam
SRR5065276	Vietnam
SRR5065277	Vietnam
SRR5065278	Vietnam
SRR5065279	Vietnam
SRR5065280	Vietnam
SRR5065281	Vietnam
SRR5065282	Vietnam
SRR5065283	Vietnam
SRR5065284	Vietnam
SRR5065285	Vietnam
SRR5065286	Vietnam
SRR5065287	Vietnam
SRR5065288	Vietnam
SRR5065289	Vietnam
SRR5065290	Vietnam
SRR5065291	Vietnam
SRR5065292	Vietnam
SRR5065293	Vietnam
SRR5065294	Vietnam
SRR5065295	Vietnam
SRR5065296	Vietnam
SRR5065297	Vietnam
SRR5065298	Vietnam
SRR5065299	Vietnam
SRR5065300	Vietnam
SRR5065301	Vietnam
SRR5065302	Vietnam
SRR5065303	Vietnam
SRR5065304	Vietnam
SRR5065305	Vietnam
SRR5065306	Vietnam
SRR5065307	Vietnam
SRR5065308	Vietnam
SRR5065309	Vietnam
SRR5065310	Vietnam
SRR5065311	Vietnam

SRR5065312	Vietnam
SRR5065313	Vietnam
SRR5065314	Vietnam
SRR5065315	Vietnam
SRR5065316	Vietnam
SRR5065317	Vietnam
SRR5065318	Vietnam
SRR5065319	Vietnam
SRR5065320	Vietnam
SRR5065321	Vietnam
SRR5065322	Vietnam
SRR5065323	Vietnam
SRR5065324	Vietnam
SRR5065325	Vietnam
SRR5065326	Vietnam
SRR5065327	Vietnam
SRR5065328	Vietnam
SRR5065329	Vietnam
SRR5065330	Vietnam
SRR5065331	Vietnam
SRR5065332	Vietnam
SRR5065333	Vietnam
SRR5065334	Vietnam
SRR5065335	Vietnam
SRR5065336	Vietnam
SRR5065337	Vietnam
SRR5065338	Vietnam
SRR5065339	Vietnam
SRR5065340	Vietnam
SRR5065341	Vietnam
SRR5065342	Vietnam
SRR5065343	Vietnam
SRR5065344	Vietnam
SRR5065345	Vietnam
SRR5065346	Vietnam
SRR5065347	Vietnam
SRR5065348	Vietnam
SRR5065349	Vietnam
SRR5065350	Vietnam
SRR5065351	Vietnam
SRR5065352	Vietnam
SRR5065353	Vietnam
SRR5065354	Vietnam
SRR5065355	Vietnam
SRR5065356	Vietnam
SRR5065357	Vietnam
SRR5065358	Vietnam
SRR5065359	Vietnam
SRR5065360	Vietnam
SRR5065361	Vietnam

SRR5065362	Vietnam
SRR5065363	Vietnam
SRR5065364	Vietnam
SRR5065365	Vietnam
SRR5065366	Vietnam
SRR5065367	Vietnam
SRR5065368	Vietnam
SRR5065369	Vietnam
SRR5065370	Vietnam
SRR5065371	Vietnam
SRR5065372	Vietnam
SRR5065373	Vietnam
SRR5065374	Vietnam
SRR5065375	Vietnam
SRR5065376	Vietnam
SRR5065377	Vietnam
SRR5065378	Vietnam
SRR5065379	Vietnam
SRR5065380	Vietnam
SRR5065381	Vietnam
SRR5065382	Vietnam
SRR5065383	Vietnam
SRR5065384	Vietnam
SRR5065385	Vietnam
SRR5065386	Vietnam
SRR5065387	Vietnam
SRR5065388	Vietnam
SRR5065395	Vietnam
SRR5065396	Vietnam
SRR5065397	Vietnam
SRR5065398	Vietnam
SRR5065399	Vietnam
SRR5065400	Vietnam
SRR5065401	Vietnam
SRR5065402	Vietnam
SRR5065403	Vietnam
SRR5065404	Vietnam
SRR5065405	Vietnam
SRR5065406	Vietnam
SRR5065407	Vietnam
SRR5065408	Vietnam
SRR5065409	Vietnam
SRR5065410	Vietnam
SRR5065411	Vietnam
SRR5065412	Vietnam
SRR5065413	Vietnam
SRR5065414	Vietnam
SRR5065415	Vietnam
SRR5065416	Vietnam
SRR5065417	Vietnam

SRR5065418	Vietnam
SRR5065419	Vietnam
SRR5065420	Vietnam
SRR5065421	Vietnam
SRR5065422	Vietnam
SRR5065423	Vietnam
SRR5065424	Vietnam
SRR5065425	Vietnam
SRR5065426	Vietnam
SRR5065427	Vietnam
SRR5065428	Vietnam
SRR5065429	Vietnam
SRR5065430	Vietnam
SRR5065431	Vietnam
SRR5065432	Vietnam
SRR5065433	Vietnam
SRR5065434	Vietnam
SRR5065435	Vietnam
SRR5065436	Vietnam
SRR5065437	Vietnam
SRR5065438	Vietnam
SRR5065439	Vietnam
SRR5065440	Vietnam
SRR5065441	Vietnam
SRR5065442	Vietnam
SRR5065443	Vietnam
SRR5065444	Vietnam
SRR5065445	Vietnam
SRR5065446	Vietnam
SRR5065447	Vietnam
SRR5065448	Vietnam
SRR5065449	Vietnam
SRR5065450	Vietnam
SRR5065451	Vietnam
SRR5065452	Vietnam
SRR5065453	Vietnam
SRR5065454	Vietnam
SRR5065455	Vietnam
SRR5065456	Vietnam
SRR5065457	Vietnam
SRR5065458	Vietnam
SRR5065459	Vietnam
SRR5065460	Vietnam
SRR5065461	Vietnam
SRR5065462	Vietnam
SRR5065463	Vietnam
SRR5065464	Vietnam
SRR5065465	Vietnam
SRR5065466	Vietnam
SRR5065467	Vietnam

SRR5065468	Vietnam
SRR5065469	Vietnam
SRR5065470	Vietnam
SRR5065471	Vietnam
SRR5065472	Vietnam
SRR5065473	Vietnam
SRR5065474	Vietnam
SRR5065475	Vietnam
SRR5065476	Vietnam
SRR5065477	Vietnam
SRR5065478	Vietnam
SRR5065479	Vietnam
SRR5065480	Vietnam
SRR5065481	Vietnam
SRR5065482	Vietnam
SRR5065483	Vietnam
SRR5065484	Vietnam
SRR5065485	Vietnam
SRR5065486	Vietnam
SRR5065487	Vietnam
SRR5065488	Vietnam
SRR5065489	Vietnam
SRR5065490	Vietnam
SRR5065491	Vietnam
SRR5065492	Vietnam
SRR5065493	Vietnam
SRR5065494	Vietnam
SRR5065495	Vietnam
SRR5065496	Vietnam
SRR5065497	Vietnam
SRR5065498	Vietnam
SRR5065499	Vietnam
SRR5065500	Vietnam
SRR5065501	Vietnam
SRR5065502	Vietnam
SRR5065503	Vietnam
SRR5065504	Vietnam
SRR5065505	Vietnam
SRR5065506	Vietnam
SRR5065507	Vietnam
SRR5065508	Vietnam
SRR5065509	Vietnam
SRR5065510	Vietnam
SRR5065511	Vietnam
SRR5065512	Vietnam
SRR5065513	Vietnam
SRR5065514	Vietnam
SRR5065515	Vietnam
SRR5065516	Vietnam
SRR5065517	Vietnam

SRR5065518	Vietnam
SRR5065519	Vietnam
SRR5065520	Vietnam
SRR5065521	Vietnam
SRR5065522	Vietnam
SRR5065523	Vietnam
SRR5065524	Vietnam
SRR5065525	Vietnam
SRR5065526	Vietnam
SRR5065527	Vietnam
SRR5065528	Vietnam
SRR5065529	Vietnam
SRR5065530	Vietnam
SRR5065531	Vietnam
SRR5065532	Vietnam
SRR5065533	Vietnam
SRR5065534	Vietnam
SRR5065535	Vietnam
SRR5065536	Vietnam
SRR5065537	Vietnam
SRR5065538	Vietnam
SRR5065539	Vietnam
SRR5065540	Vietnam
SRR5065541	Vietnam
SRR5065542	Vietnam
SRR5065543	Vietnam
SRR5065544	Vietnam
SRR5065545	Vietnam
SRR5065546	Vietnam
SRR5065547	Vietnam
SRR5065548	Vietnam
SRR5065549	Vietnam
SRR5065550	Vietnam
SRR5065551	Vietnam
SRR5065554	Vietnam
SRR5065555	Vietnam
SRR5065556	Vietnam
SRR5065557	Vietnam
SRR5065558	Vietnam
SRR5065559	Vietnam
SRR5065560	Vietnam
SRR5065561	Vietnam
SRR5065562	Vietnam
SRR5065563	Vietnam
SRR5065564	Vietnam
SRR5065565	Vietnam
SRR5065566	Vietnam
SRR5065567	Vietnam
SRR5065568	Vietnam
SRR5065569	Vietnam

SRR5065570	Vietnam
SRR5065571	Vietnam
SRR5065572	Vietnam
SRR5065573	Vietnam
SRR5065574	Vietnam
SRR5065575	Vietnam
SRR5065576	Vietnam
SRR5065577	Vietnam
SRR5065578	Vietnam
SRR5065579	Vietnam
SRR5065580	Vietnam
SRR5065581	Vietnam
SRR5065582	Vietnam
SRR5065583	Vietnam
SRR5065584	Vietnam
SRR5065585	Vietnam
SRR5065586	Vietnam
SRR5065587	Vietnam
SRR5065588	Vietnam
SRR5065589	Vietnam
SRR5065590	Vietnam
SRR5065591	Vietnam
SRR5065592	Vietnam
SRR5065593	Vietnam
SRR5065594	Vietnam
SRR5065595	Vietnam
SRR5065596	Vietnam
SRR5065597	Vietnam
SRR5065598	Vietnam
SRR5065599	Vietnam
SRR5065600	Vietnam
SRR5065601	Vietnam
SRR5065602	Vietnam
SRR5065603	Vietnam
SRR5065604	Vietnam
SRR5065605	Vietnam
SRR5065606	Vietnam
SRR5065607	Vietnam
SRR5065608	Vietnam
SRR5065609	Vietnam
SRR5065610	Vietnam
SRR5065611	Vietnam
SRR5065612	Vietnam
SRR5065613	Vietnam
SRR5065614	Vietnam
SRR5065615	Vietnam
SRR5065616	Vietnam
SRR5065617	Vietnam
SRR5065618	Vietnam
SRR5065619	Vietnam

SRR5065620	Vietnam
SRR5065621	Vietnam
SRR5065622	Vietnam
SRR5065623	Vietnam
SRR5065624	Vietnam
SRR5065625	Vietnam
SRR5065626	Vietnam
SRR5065627	Vietnam
SRR5065628	Vietnam
SRR5065629	Vietnam
SRR5065630	Vietnam
SRR5065631	Vietnam
SRR5065632	Vietnam
SRR5065633	Vietnam
SRR5065634	Vietnam
SRR5065635	Vietnam
SRR5065636	Vietnam
SRR5065637	Vietnam
SRR5065638	Vietnam
SRR5065639	Vietnam
SRR5065640	Vietnam
SRR5065641	Vietnam
SRR5065642	Vietnam
SRR5065643	Vietnam
SRR5065644	Vietnam
SRR5065645	Vietnam
SRR5065646	Vietnam
SRR5065647	Vietnam
SRR5065648	Vietnam
SRR5065649	Vietnam
SRR5065650	Vietnam
SRR5065651	Vietnam
SRR5065652	Vietnam
SRR5065653	Vietnam
SRR5065654	Vietnam
SRR5065655	Vietnam
SRR5065656	Vietnam
SRR5065657	Vietnam
SRR5065658	Vietnam
SRR5065659	Vietnam
SRR5065660	Vietnam
SRR5065661	Vietnam
SRR5065662	Vietnam
SRR5065663	Vietnam
SRR5065664	Vietnam
SRR5065665	Vietnam
SRR5065666	Vietnam
SRR5065667	Vietnam
SRR5065668	Vietnam
SRR5065669	Vietnam

SRR5065670	Vietnam
SRR5065671	Vietnam
SRR5065672	Vietnam
SRR5065673	Vietnam
SRR5065674	Vietnam
SRR5065675	Vietnam
SRR5065676	Vietnam
SRR5065677	Vietnam
SRR5065678	Vietnam
SRR5065679	Vietnam
SRR5065680	Vietnam
SRR5065681	Vietnam
SRR5065682	Vietnam
SRR5065683	Vietnam
SRR5065684	Vietnam
SRR5065685	Vietnam
SRR5065686	Vietnam
SRR5065687	Vietnam
SRR5065688	Vietnam
SRR5065689	Vietnam
SRR5065690	Vietnam
SRR5065691	Vietnam
SRR5065692	Vietnam
SRR5065693	Vietnam
SRR5065694	Vietnam
SRR5065695	Vietnam
SRR5065696	Vietnam
SRR5065697	Vietnam
SRR5065698	Vietnam
SRR5065699	Vietnam
SRR5065700	Vietnam
SRR5065701	Vietnam
SRR5065702	Vietnam
SRR5065703	Vietnam
SRR5065704	Vietnam
SRR5065705	Vietnam
SRR5065706	Vietnam
SRR5067220	Vietnam
SRR5067221	Vietnam
SRR5067222	Vietnam
SRR5067223	Vietnam
SRR5067224	Vietnam
SRR5067225	Vietnam
SRR5067226	Vietnam
SRR5067227	Vietnam
SRR5067228	Vietnam
SRR5067229	Vietnam
SRR5067230	Vietnam
SRR5067231	Vietnam
SRR5067232	Vietnam

SRR5067233	Vietnam
SRR5067234	Vietnam
SRR5067235	Vietnam
SRR5067236	Vietnam
SRR5067237	Vietnam
SRR5067238	Vietnam
SRR5067239	Vietnam
SRR5067240	Vietnam
SRR5067241	Vietnam
SRR5067242	Vietnam
SRR5067243	Vietnam
SRR5067244	Vietnam
SRR5067245	Vietnam
SRR5067246	Vietnam
SRR5067247	Vietnam
SRR5067248	Vietnam
SRR5067249	Vietnam
SRR5067250	Vietnam
SRR5067251	Vietnam
SRR5067252	Vietnam
SRR5067253	Vietnam
SRR5067254	Vietnam
SRR5067255	Vietnam
SRR5067256	Vietnam
SRR5067257	Vietnam
SRR5067258	Vietnam
SRR5067259	Vietnam
SRR5067260	Vietnam
SRR5067261	Vietnam
SRR5067262	Vietnam
SRR5067263	Vietnam
SRR5067264	Vietnam
SRR5067265	Vietnam
SRR5067266	Vietnam
SRR5067267	Vietnam
SRR5067268	Vietnam
SRR5067269	Vietnam
SRR5067270	Vietnam
SRR5067271	Vietnam
SRR5067272	Vietnam
SRR5067273	Vietnam
SRR5067274	Vietnam
SRR5067275	Vietnam
SRR5067276	Vietnam
SRR5067277	Vietnam
SRR5067278	Vietnam
SRR5067279	Vietnam
SRR5067280	Vietnam
SRR5067281	Vietnam
SRR5067282	Vietnam

SRR5067283	Vietnam
SRR5067284	Vietnam
SRR5067285	Vietnam
SRR5067286	Vietnam
SRR5067287	Vietnam
SRR5067288	Vietnam
SRR5067289	Vietnam
SRR5067290	Vietnam
SRR5067291	Vietnam
SRR5067292	Vietnam
SRR5067293	Vietnam
SRR5067294	Vietnam
SRR5067295	Vietnam
SRR5067296	Vietnam
SRR5067297	Vietnam
SRR5067298	Vietnam
SRR5067299	Vietnam
SRR5067300	Vietnam
SRR5067301	Vietnam
SRR5067302	Vietnam
SRR5067303	Vietnam
SRR5067304	Vietnam
SRR5067305	Vietnam
SRR5067306	Vietnam
SRR5067307	Vietnam
SRR5067308	Vietnam
SRR5067309	Vietnam
SRR5067310	Vietnam
SRR5067311	Vietnam
SRR5067312	Vietnam
SRR5067313	Vietnam
SRR5067314	Vietnam
SRR5067315	Vietnam
SRR5067316	Vietnam
SRR5067317	Vietnam
SRR5067318	Vietnam
SRR5067319	Vietnam
SRR5067320	Vietnam
SRR5067321	Vietnam
SRR5067322	Vietnam
SRR5067323	Vietnam
SRR5067324	Vietnam
SRR5067325	Vietnam
SRR5067326	Vietnam
SRR5067327	Vietnam
SRR5067328	Vietnam
SRR5067329	Vietnam
SRR5067330	Vietnam
SRR5067331	Vietnam
SRR5067332	Vietnam

SRR5067333	Vietnam
SRR5067334	Vietnam
SRR5067335	Vietnam
SRR5067336	Vietnam
SRR5067337	Vietnam
SRR5067338	Vietnam
SRR5067339	Vietnam
SRR5067340	Vietnam
SRR5067341	Vietnam
SRR5067342	Vietnam
SRR5067343	Vietnam
SRR5067344	Vietnam
SRR5067345	Vietnam
SRR5067346	Vietnam
SRR5067347	Vietnam
SRR5067348	Vietnam
SRR5067349	Vietnam
SRR5067350	Vietnam
SRR5067351	Vietnam
SRR5067352	Vietnam
SRR5067353	Vietnam
SRR5067354	Vietnam
SRR5067355	Vietnam
SRR5067356	Vietnam
SRR5067357	Vietnam
SRR5067358	Vietnam
SRR5067359	Vietnam
SRR5067360	Vietnam
SRR5067361	Vietnam
SRR5067362	Vietnam
SRR5067363	Vietnam
SRR5067364	Vietnam
SRR5067365	Vietnam
SRR5067366	Vietnam
SRR5067367	Vietnam
SRR5067368	Vietnam
SRR5067369	Vietnam
SRR5067370	Vietnam
SRR5067371	Vietnam
SRR5067372	Vietnam
SRR5067373	Vietnam
SRR5067374	Vietnam
SRR5067375	Vietnam
SRR5067376	Vietnam
SRR5067377	Vietnam
SRR5067378	Vietnam
SRR5067379	Vietnam
SRR5067380	Vietnam
SRR5067381	Vietnam
SRR5067382	Vietnam

SRR5067383	Vietnam
SRR5067384	Vietnam
SRR5067385	Vietnam
SRR5067386	Vietnam
SRR5067387	Vietnam
SRR5067388	Vietnam
SRR5067389	Vietnam
SRR5067390	Vietnam
SRR5067391	Vietnam
SRR5067392	Vietnam
SRR5067393	Vietnam
SRR5067394	Vietnam
SRR5067395	Vietnam
SRR5067396	Vietnam
SRR5067397	Vietnam
SRR5067398	Vietnam
SRR5067399	Vietnam
SRR5067400	Vietnam
SRR5067401	Vietnam
SRR5067402	Vietnam
SRR5067403	Vietnam
SRR5067404	Vietnam
SRR5067405	Vietnam
SRR5067406	Vietnam
SRR5067407	Vietnam
SRR5067408	Vietnam
SRR5067409	Vietnam
SRR5067410	Vietnam
SRR5067411	Vietnam
SRR5067412	Vietnam
SRR5067413	Vietnam
SRR5067414	Vietnam
SRR5067415	Vietnam
SRR5067416	Vietnam
SRR5067417	Vietnam
SRR5067418	Vietnam
SRR5067419	Vietnam
SRR5067420	Vietnam
SRR5067421	Vietnam
SRR5067422	Vietnam
SRR5067423	Vietnam
SRR5067424	Vietnam
SRR5067425	Vietnam
SRR5067426	Vietnam
SRR5067427	Vietnam
SRR5067428	Vietnam
SRR5067429	Vietnam
SRR5067430	Vietnam
SRR5067431	Vietnam
SRR5067432	Vietnam

SRR5067433	Vietnam
SRR5067434	Vietnam
SRR5067435	Vietnam
SRR5067436	Vietnam
SRR5067437	Vietnam
SRR5067438	Vietnam
SRR5067439	Vietnam
SRR5067440	Vietnam
SRR5067441	Vietnam
SRR5067442	Vietnam
SRR5067443	Vietnam
SRR5067444	Vietnam
SRR5067445	Vietnam
SRR5067446	Vietnam
SRR5067447	Vietnam
SRR5067448	Vietnam
SRR5067449	Vietnam
SRR5067450	Vietnam
SRR5067451	Vietnam
SRR5067452	Vietnam
SRR5067453	Vietnam
SRR5067454	Vietnam
SRR5067455	Vietnam
SRR5067456	Vietnam
SRR5067457	Vietnam
SRR5067458	Vietnam
SRR5067459	Vietnam
SRR5067460	Vietnam
SRR5067461	Vietnam
SRR5067462	Vietnam
SRR5067463	Vietnam
SRR5067464	Vietnam
SRR5067465	Vietnam
SRR5067466	Vietnam
SRR5067467	Vietnam
SRR5067468	Vietnam
SRR5067469	Vietnam
SRR5067470	Vietnam
SRR5067471	Vietnam
SRR5067472	Vietnam
SRR5067473	Vietnam
SRR5067474	Vietnam
SRR5067475	Vietnam
SRR5067476	Vietnam
SRR5067477	Vietnam
SRR5067478	Vietnam
SRR5067479	Vietnam
SRR5067480	Vietnam
SRR5067481	Vietnam
SRR5067482	Vietnam

SRR5067483	Vietnam
SRR5067484	Vietnam
SRR5067485	Vietnam
SRR5067486	Vietnam
SRR5067487	Vietnam
SRR5067488	Vietnam
SRR5067489	Vietnam
SRR5067490	Vietnam
SRR5067491	Vietnam
SRR5067492	Vietnam
SRR5067493	Vietnam
SRR5067494	Vietnam
SRR5067495	Vietnam
SRR5067496	Vietnam
SRR5067497	Vietnam
SRR5067498	Vietnam
SRR5067499	Vietnam
SRR5067500	Vietnam
SRR5067501	Vietnam
SRR5067502	Vietnam
SRR5067503	Vietnam
SRR5067504	Vietnam
SRR5067505	Vietnam
SRR5067506	Vietnam
SRR5067507	Vietnam
SRR5067508	Vietnam
SRR5067509	Vietnam
SRR5067510	Vietnam
SRR5067511	Vietnam
SRR5067512	Vietnam
SRR5067513	Vietnam
SRR5067514	Vietnam
SRR5067515	Vietnam
SRR5067516	Vietnam
SRR5067517	Vietnam
SRR5067518	Vietnam
SRR5067519	Vietnam
SRR5067520	Vietnam
SRR5067521	Vietnam
SRR5067522	Vietnam
SRR5067523	Vietnam
SRR5067524	Vietnam
SRR5067525	Vietnam
SRR5067526	Vietnam
SRR5067527	Vietnam
SRR5067528	Vietnam
SRR5067529	Vietnam
SRR5067530	Vietnam
SRR5067531	Vietnam
SRR5067532	Vietnam

SRR5067533	Vietnam
SRR5067534	Vietnam
SRR5067535	Vietnam
SRR5067536	Vietnam
SRR5067537	Vietnam
SRR5067538	Vietnam
SRR5067539	Vietnam
SRR5067540	Vietnam
SRR5067541	Vietnam
SRR5067542	Vietnam
SRR5067543	Vietnam
SRR5067544	Vietnam
SRR5067545	Vietnam
SRR5067546	Vietnam
SRR5067547	Vietnam
SRR5067548	Vietnam
SRR5067549	Vietnam
SRR5067550	Vietnam
SRR5067551	Vietnam
SRR5067552	Vietnam
SRR5067553	Vietnam
SRR5067554	Vietnam
SRR5067555	Vietnam
SRR5067556	Vietnam
SRR5067557	Vietnam
SRR5067558	Vietnam
SRR5067559	Vietnam
SRR5067560	Vietnam
SRR5067561	Vietnam
SRR5067562	Vietnam
SRR5067563	Vietnam
SRR5067564	Vietnam
SRR5067565	Vietnam
SRR5067566	Vietnam
SRR5067567	Vietnam
SRR5067568	Vietnam
SRR5067569	Vietnam
SRR5067570	Vietnam
SRR5067571	Vietnam
SRR5067572	Vietnam
SRR5067573	Vietnam
SRR5067574	Vietnam
SRR5067575	Vietnam
SRR5067576	Vietnam
SRR5067577	Vietnam
SRR5067578	Vietnam
SRR5067579	Vietnam
SRR5067580	Vietnam
SRR5067581	Vietnam
SRR5067582	Vietnam

SRR5067583	Vietnam
SRR5067584	Vietnam
SRR5067585	Vietnam
SRR5067586	Vietnam
SRR5067587	Vietnam
SRR5067588	Vietnam
SRR5067589	Vietnam
SRR5067590	Vietnam
SRR5067591	Vietnam
SRR5067592	Vietnam
SRR5067593	Vietnam
SRR5067594	Vietnam
SRR5067595	Vietnam
SRR5067596	Vietnam
SRR5067597	Vietnam
SRR5067598	Vietnam
SRR5067599	Vietnam
SRR5067600	Vietnam
SRR5067601	Vietnam
SRR5067602	Vietnam
SRR5067603	Vietnam
SRR5067604	Vietnam
SRR5067605	Vietnam
SRR5067606	Vietnam
SRR5067607	Vietnam
SRR5067608	Vietnam
SRR5067609	Vietnam
SRR5067610	Vietnam
SRR5067611	Vietnam
SRR5067612	Vietnam
SRR5067613	Vietnam
SRR5067614	Vietnam
SRR5067615	Vietnam
SRR5067616	Vietnam
SRR5067617	Vietnam
SRR5067618	Vietnam
SRR5067619	Vietnam
SRR5067620	Vietnam
SRR5067621	Vietnam
SRR5067622	Vietnam
SRR5067623	Vietnam
SRR5067624	Vietnam
SRR5067625	Vietnam
SRR5067626	Vietnam
SRR5067627	Vietnam
SRR5067628	Vietnam
SRR5067629	Vietnam
SRR5067630	Vietnam
SRR5067631	Vietnam
SRR5067632	Vietnam

SRR5067633	Vietnam
SRR5067634	Vietnam
SRR5067635	Vietnam
SRR5067636	Vietnam
SRR5067637	Vietnam
SRR5067638	Vietnam
SRR5067639	Vietnam
SRR5067640	Vietnam
SRR5067641	Vietnam
SRR5067642	Vietnam
SRR5067643	Vietnam
SRR5067644	Vietnam
SRR5067645	Vietnam
SRR5067646	Vietnam
SRR5067647	Vietnam
SRR5067648	Vietnam
SRR5067649	Vietnam
SRR5067650	Vietnam
SRR5067651	Vietnam
SRR5067652	Vietnam
SRR5067653	Vietnam
SRR5067654	Vietnam
SRR5067655	Vietnam
SRR5067656	Vietnam
SRR5067657	Vietnam
SRR5067658	Vietnam
SRR5067659	Vietnam
SRR5067660	Vietnam
SRR5067661	Vietnam
SRR5067662	Vietnam
SRR5067663	Vietnam
SRR5067664	Vietnam
SRR5067665	Vietnam
SRR5067666	Vietnam
SRR5067667	Vietnam
SRR5067668	Vietnam
SRR5067669	Vietnam
SRR5067670	Vietnam
SRR5067671	Vietnam
SRR5067672	Vietnam
SRR5067673	Vietnam
SRR5067674	Vietnam
SRR5067675	Vietnam
SRR5067676	Vietnam
SRR5067677	Vietnam
SRR5067678	Vietnam
SRR5067679	Vietnam
SRR5067680	Vietnam
SRR5067681	Vietnam
SRR5067682	Vietnam

SRR5067683	Vietnam
SRR5067684	Vietnam
SRR5067685	Vietnam
SRR5067686	Vietnam
SRR5067687	Vietnam
SRR5067688	Vietnam
SRR5067689	Vietnam
SRR5067690	Vietnam
SRR5067691	Vietnam
SRR5067692	Vietnam
SRR5067693	Vietnam
SRR5067694	Vietnam
SRR5067695	Vietnam
SRR5067696	Vietnam
SRR5067697	Vietnam
SRR5067698	Vietnam
SRR5067699	Vietnam
SRR5067700	Vietnam
SRR5067701	Vietnam
SRR5067702	Vietnam
SRR5067703	Vietnam
SRR5067704	Vietnam
SRR5067705	Vietnam
SRR5067706	Vietnam
SRR5067707	Vietnam
SRR5067708	Vietnam
SRR5067709	Vietnam
SRR5067710	Vietnam
SRR5067711	Vietnam
SRR5067712	Vietnam
SRR5067713	Vietnam
SRR5067714	Vietnam
SRR5067715	Vietnam
SRR5067716	Vietnam
SRR5067717	Vietnam
SRR5067718	Vietnam
SRR5073499	Vietnam
SRR5073500	Vietnam
SRR5073501	Vietnam
SRR5073502	Vietnam
SRR5073503	Vietnam
SRR5073504	Vietnam
SRR5073505	Vietnam
SRR5073506	Vietnam
SRR5073507	Vietnam
SRR5073508	Vietnam
SRR5073509	Vietnam
SRR5073510	Vietnam
SRR5073511	Vietnam
SRR5073512	Vietnam

SRR5073513	Vietnam
SRR5073514	Vietnam
SRR5073515	Vietnam
SRR5073516	Vietnam
SRR5073517	Vietnam
SRR5073518	Vietnam
SRR5073519	Vietnam
SRR5073520	Vietnam
SRR5073521	Vietnam
SRR5073522	Vietnam
SRR5073523	Vietnam
SRR5073524	Vietnam
SRR5073525	Vietnam
SRR5073526	Vietnam
SRR5073527	Vietnam
SRR5073528	Vietnam
SRR5073529	Vietnam
SRR5073530	Vietnam
SRR5073531	Vietnam
SRR5073532	Vietnam
SRR5073533	Vietnam
SRR5073534	Vietnam
SRR5073535	Vietnam
SRR5073536	Vietnam
SRR5073537	Vietnam
SRR5073538	Vietnam
SRR5073539	Vietnam
SRR5073540	Vietnam
SRR5073541	Vietnam
SRR5073542	Vietnam
SRR5073543	Vietnam
SRR5073544	Vietnam
SRR5073545	Vietnam
SRR5073546	Vietnam
SRR5073547	Vietnam
SRR5073548	Vietnam
SRR5073549	Vietnam
SRR5073550	Vietnam
SRR5073551	Vietnam
SRR5073552	Vietnam
SRR5073553	Vietnam
SRR5073554	Vietnam
SRR5073555	Vietnam
SRR5073556	Vietnam
SRR5073557	Vietnam
SRR5073561	Vietnam
SRR5073562	Vietnam
SRR5073563	Vietnam
SRR5073564	Vietnam
SRR5073565	Vietnam

SRR5073566	Vietnam
SRR5073567	Vietnam
SRR5073568	Vietnam
SRR5073569	Vietnam
SRR5073570	Vietnam
SRR5073571	Vietnam
SRR5073572	Vietnam
SRR5073573	Vietnam
SRR5073574	Vietnam
SRR5073575	Vietnam
SRR5073576	Vietnam
SRR5073577	Vietnam
SRR5073578	Vietnam
SRR5073579	Vietnam
SRR5073580	Vietnam
SRR5073581	Vietnam
SRR5073582	Vietnam
SRR5073583	Vietnam
SRR5073584	Vietnam
SRR5073585	Vietnam
SRR5073586	Vietnam
SRR5073587	Vietnam
SRR5073588	Vietnam
SRR5073589	Vietnam
SRR5073590	Vietnam
SRR5073591	Vietnam
SRR5073592	Vietnam
SRR5073593	Vietnam
SRR5073594	Vietnam
SRR5073595	Vietnam
SRR5073596	Vietnam
SRR5073597	Vietnam
SRR5073598	Vietnam
SRR5073599	Vietnam
SRR5073600	Vietnam
SRR5073601	Vietnam
SRR5073602	Vietnam
SRR5073603	Vietnam
SRR5073604	Vietnam
SRR5073605	Vietnam
SRR5073606	Vietnam
SRR5073607	Vietnam
SRR5073608	Vietnam
SRR5073609	Vietnam
SRR5073610	Vietnam
SRR5073611	Vietnam
SRR5073612	Vietnam
SRR5073613	Vietnam
SRR5073614	Vietnam
SRR5073615	Vietnam

SRR5073616	Vietnam
SRR5073617	Vietnam
SRR5073618	Vietnam
SRR5073619	Vietnam
SRR5073620	Vietnam
SRR5073621	Vietnam
SRR5073622	Vietnam
SRR5073623	Vietnam
SRR5073624	Vietnam
SRR5073625	Vietnam
SRR5073626	Vietnam
SRR5073627	Vietnam
SRR5073628	Vietnam
SRR5073629	Vietnam
SRR5073630	Vietnam
SRR5073631	Vietnam
SRR5073632	Vietnam
SRR5073633	Vietnam
SRR5073634	Vietnam
SRR5073635	Vietnam
SRR5073636	Vietnam
SRR5073637	Vietnam
SRR5073638	Vietnam
SRR5073639	Vietnam
SRR5073640	Vietnam
SRR5073641	Vietnam
SRR5073642	Vietnam
SRR5073643	Vietnam
SRR5073644	Vietnam
SRR5073645	Vietnam
SRR5073646	Vietnam
SRR5073647	Vietnam
SRR5073648	Vietnam
SRR5073649	Vietnam
SRR5073650	Vietnam
SRR5073651	Vietnam
SRR5073652	Vietnam
SRR5073653	Vietnam
SRR5073654	Vietnam
SRR5073655	Vietnam
SRR5073656	Vietnam
SRR5073657	Vietnam
SRR5073658	Vietnam
SRR5073659	Vietnam
SRR5073660	Vietnam
SRR5073661	Vietnam
SRR5073662	Vietnam
SRR5073663	Vietnam
SRR5073664	Vietnam
SRR5073665	Vietnam

SRR5073666	Vietnam
SRR5073667	Vietnam
SRR5073668	Vietnam
SRR5073669	Vietnam
SRR5073670	Vietnam
SRR5073671	Vietnam
SRR5073672	Vietnam
SRR5073673	Vietnam
SRR5073674	Vietnam
SRR5073675	Vietnam
SRR5073676	Vietnam
SRR5073677	Vietnam
SRR5073678	Vietnam
SRR5073679	Vietnam
SRR5073680	Vietnam
SRR5073681	Vietnam
SRR5073682	Vietnam
SRR5073683	Vietnam
SRR5073684	Vietnam
SRR5073685	Vietnam
SRR5073686	Vietnam
SRR5073687	Vietnam
SRR5073688	Vietnam
SRR5073689	Vietnam
SRR5073690	Vietnam
SRR5073691	Vietnam
SRR5073692	Vietnam
SRR5073693	Vietnam
SRR5073694	Vietnam
SRR5073695	Vietnam
SRR5073696	Vietnam
SRR5073697	Vietnam
SRR5073698	Vietnam
SRR5073699	Vietnam
SRR5073700	Vietnam
SRR5073701	Vietnam
SRR5073702	Vietnam
SRR5073703	Vietnam
SRR5073704	Vietnam
SRR5073705	Vietnam
SRR5073706	Vietnam
SRR5073707	Vietnam
SRR5073708	Vietnam
SRR5073709	Vietnam
SRR5073710	Vietnam
SRR5073711	Vietnam
SRR5073712	Vietnam
SRR5073713	Vietnam
SRR5073714	Vietnam
SRR5073715	Vietnam

SRR5073716	Vietnam
SRR5073717	Vietnam
SRR5073718	Vietnam
SRR5073719	Vietnam
SRR5073720	Vietnam
SRR5073721	Vietnam
SRR5073722	Vietnam
SRR5073723	Vietnam
SRR5073724	Vietnam
SRR5073725	Vietnam
SRR5073726	Vietnam
SRR5073727	Vietnam
SRR5073728	Vietnam
SRR5073729	Vietnam
SRR5073730	Vietnam
SRR5073731	Vietnam
SRR5073732	Vietnam
SRR5073733	Vietnam
SRR5073734	Vietnam
SRR5073735	Vietnam
SRR5073736	Vietnam
SRR5073737	Vietnam
SRR5073738	Vietnam
SRR5073739	Vietnam
SRR5073740	Vietnam
SRR5073741	Vietnam
SRR5073742	Vietnam
SRR5073743	Vietnam
SRR5073744	Vietnam
SRR5073745	Vietnam
SRR5073746	Vietnam
SRR5073747	Vietnam
SRR5073748	Vietnam
SRR5073749	Vietnam
SRR5073750	Vietnam
SRR5073751	Vietnam
SRR5073752	Vietnam
SRR5073753	Vietnam
SRR5073754	Vietnam
SRR5073755	Vietnam
SRR5073756	Vietnam
SRR5073757	Vietnam
SRR5073758	Vietnam
SRR5073759	Vietnam
SRR5073760	Vietnam
SRR5073761	Vietnam
SRR5073762	Vietnam
SRR5073763	Vietnam
SRR5073764	Vietnam
SRR5073765	Vietnam

SRR5073766	Vietnam
SRR5073767	Vietnam
SRR5073768	Vietnam
SRR5073769	Vietnam
SRR5073770	Vietnam
SRR5073771	Vietnam
SRR5073772	Vietnam
SRR5073773	Vietnam
SRR5073774	Vietnam
SRR5073775	Vietnam
SRR5073776	Vietnam
SRR5073777	Vietnam
SRR5073778	Vietnam
SRR5073779	Vietnam
SRR5073780	Vietnam
SRR5073781	Vietnam
SRR5073782	Vietnam
SRR5073783	Vietnam
SRR5073784	Vietnam
SRR5073785	Vietnam
SRR5073786	Vietnam
SRR5073787	Vietnam
SRR5073788	Vietnam
SRR5073789	Vietnam
SRR5073790	Vietnam
SRR5073791	Vietnam
SRR5073792	Vietnam
SRR5073793	Vietnam
SRR5073794	Vietnam
SRR5073795	Vietnam
SRR5073796	Vietnam
SRR5073797	Vietnam
SRR5073798	Vietnam
SRR5073799	Vietnam
SRR5073800	Vietnam
SRR5073801	Vietnam
SRR5073802	Vietnam
SRR5073803	Vietnam
SRR5073804	Vietnam
SRR5073805	Vietnam
SRR5073806	Vietnam
SRR5073807	Vietnam
SRR5073808	Vietnam
SRR5073809	Vietnam
SRR5073810	Vietnam
SRR5073811	Vietnam
SRR5073812	Vietnam
SRR5073813	Vietnam
SRR5073814	Vietnam
SRR5073815	Vietnam

SRR5073816	Vietnam
SRR5073817	Vietnam
SRR5073818	Vietnam
SRR5073819	Vietnam
SRR5073820	Vietnam
SRR5073821	Vietnam
SRR5073822	Vietnam
SRR5073823	Vietnam
SRR5073824	Vietnam
SRR5073825	Vietnam
SRR5073826	Vietnam
SRR5073827	Vietnam
SRR5073828	Vietnam
SRR5073829	Vietnam
SRR5073830	Vietnam
SRR5073831	Vietnam
SRR5073832	Vietnam
SRR5073833	Vietnam
SRR5073834	Vietnam
SRR5073835	Vietnam
SRR5073836	Vietnam
SRR5073837	Vietnam
SRR5073838	Vietnam
SRR5073839	Vietnam
SRR5073840	Vietnam
SRR5073841	Vietnam
SRR5073842	Vietnam
SRR5073843	Vietnam
SRR5073844	Vietnam
SRR5073845	Vietnam
SRR5073846	Vietnam
SRR5073847	Vietnam
SRR5073848	Vietnam
SRR5073849	Vietnam
SRR5073850	Vietnam
SRR5073851	Vietnam
SRR5073852	Vietnam
SRR5073853	Vietnam
SRR5073854	Vietnam
SRR5073855	Vietnam
SRR5073856	Vietnam
SRR5073857	Vietnam
SRR5073858	Vietnam
SRR5073859	Vietnam
SRR5073860	Vietnam
SRR5073861	Vietnam
SRR5073862	Vietnam
SRR5073863	Vietnam
SRR5073864	Vietnam
SRR5073865	Vietnam

SRR5073866	Vietnam
SRR5073867	Vietnam
SRR5073868	Vietnam
SRR5073869	Vietnam
SRR5073870	Vietnam
SRR5073871	Vietnam
SRR5073872	Vietnam
SRR5073873	Vietnam
SRR5073874	Vietnam
SRR5073875	Vietnam
SRR5073876	Vietnam
SRR5073877	Vietnam
SRR5073878	Vietnam
SRR5073879	Vietnam
SRR5073880	Vietnam
SRR5073881	Vietnam
SRR5073882	Vietnam
SRR5073883	Vietnam
SRR5073884	Vietnam
SRR5073885	Vietnam
SRR5073886	Vietnam
SRR5073887	Vietnam
SRR5073888	Vietnam
SRR5073889	Vietnam
SRR5073890	Vietnam
SRR5073891	Vietnam
SRR5073892	Vietnam
SRR5073893	Vietnam
SRR5073894	Vietnam
SRR5073895	Vietnam
SRR5073896	Vietnam
SRR5073897	Vietnam
SRR5073898	Vietnam
SRR5073899	Vietnam
SRR5073900	Vietnam
SRR5073901	Vietnam
SRR5073902	Vietnam
SRR5073903	Vietnam
SRR5073904	Vietnam
SRR5073905	Vietnam
SRR5073906	Vietnam
SRR5073907	Vietnam
SRR5073908	Vietnam
SRR5073909	Vietnam
SRR5073910	Vietnam
SRR5073911	Vietnam
SRR5073912	Vietnam
SRR5073913	Vietnam
SRR5073914	Vietnam
SRR5073915	Vietnam

SRR5073916	Vietnam
SRR5073917	Vietnam
SRR5073918	Vietnam
SRR5073919	Vietnam
SRR5073920	Vietnam
SRR5073921	Vietnam
SRR5073922	Vietnam
SRR5073923	Vietnam
SRR5073924	Vietnam
SRR5073925	Vietnam
SRR5073926	Vietnam
SRR5073927	Vietnam
SRR5073928	Vietnam
SRR5073929	Vietnam
SRR5073930	Vietnam
SRR5073931	Vietnam
SRR5073932	Vietnam
SRR5073933	Vietnam
SRR5073934	Vietnam
SRR5073935	Vietnam
SRR5073936	Vietnam
SRR5073937	Vietnam
SRR5073938	Vietnam
SRR5073939	Vietnam
SRR5073940	Vietnam
SRR5073941	Vietnam
SRR5073942	Vietnam
SRR5073943	Vietnam
SRR5073944	Vietnam
SRR5073945	Vietnam
SRR5073946	Vietnam
SRR5073947	Vietnam
SRR5073948	Vietnam
SRR5073949	Vietnam
SRR5073950	Vietnam
SRR5073951	Vietnam
SRR5073952	Vietnam
SRR5073953	Vietnam
SRR5073954	Vietnam
SRR5073955	Vietnam
SRR5073956	Vietnam
SRR5073957	Vietnam
SRR5073958	Vietnam
SRR5073959	Vietnam
SRR5073960	Vietnam
SRR5073961	Vietnam
SRR5073962	Vietnam
SRR5073963	Vietnam
SRR5073964	Vietnam
SRR5073965	Vietnam

SRR5073966	Vietnam
SRR5073967	Vietnam
SRR5073968	Vietnam
SRR5073969	Vietnam
SRR5073970	Vietnam
SRR5073971	Vietnam
SRR5073972	Vietnam
SRR5073973	Vietnam
SRR5073974	Vietnam
SRR5073975	Vietnam
SRR5073976	Vietnam
SRR5073977	Vietnam
SRR5073978	Vietnam
SRR5073979	Vietnam
SRR5073980	Vietnam
SRR5073981	Vietnam
SRR5073982	Vietnam
SRR5073983	Vietnam
SRR5073984	Vietnam
SRR5073985	Vietnam
SRR5073986	Vietnam
SRR5073987	Vietnam
SRR5073988	Vietnam
SRR5073989	Vietnam
SRR5073990	Vietnam
SRR5073991	Vietnam
SRR5073992	Vietnam
SRR5073993	Vietnam
SRR5073994	Vietnam
SRR5073995	Vietnam
SRR5073996	Vietnam
SRR5073997	Vietnam
SRR5073998	Vietnam
SRR5073999	Vietnam
SRR5074000	Vietnam
SRR5074053	Vietnam
SRR5074054	Vietnam
SRR5074055	Vietnam
SRR5074056	Vietnam
SRR5074057	Vietnam
SRR5074058	Vietnam
SRR5074059	Vietnam
SRR5074060	Vietnam
SRR5074061	Vietnam
SRR5074062	Vietnam
SRR5074063	Vietnam
SRR5074064	Vietnam
SRR5074065	Vietnam
SRR5074066	Vietnam
SRR5074067	Vietnam

SRR5074068	Vietnam
SRR5074069	Vietnam
SRR5074070	Vietnam
SRR5074071	Vietnam
SRR5074072	Vietnam
SRR5074073	Vietnam
SRR5074074	Vietnam
SRR5074075	Vietnam
SRR5074076	Vietnam
SRR5074078	Vietnam
SRR5074079	Vietnam
SRR5074080	Vietnam
SRR5074081	Vietnam
SRR5074082	Vietnam
SRR5074083	Vietnam
SRR5074084	Vietnam
SRR5074085	Vietnam
SRR5074086	Vietnam
SRR5074087	Vietnam
SRR5074088	Vietnam
SRR5074089	Vietnam
SRR5074090	Vietnam
SRR5074091	Vietnam
SRR5074092	Vietnam
SRR5074093	Vietnam
SRR5074094	Vietnam
SRR5074095	Vietnam
SRR5074096	Vietnam
SRR5074097	Vietnam
SRR5074098	Vietnam
SRR5074099	Vietnam
SRR5074100	Vietnam
SRR5074101	Vietnam
SRR5074102	Vietnam
SRR5074103	Vietnam
SRR5074104	Vietnam
SRR5074105	Vietnam
SRR5074106	Vietnam
SRR5074107	Vietnam
SRR5074108	Vietnam
SRR5074109	Vietnam
SRR5074110	Vietnam
SRR5074111	Vietnam
SRR5074112	Vietnam
SRR5074113	Vietnam
SRR5074114	Vietnam
SRR5074115	Vietnam
SRR5074116	Vietnam
SRR5074117	Vietnam
SRR5074118	Vietnam

SRR5074119	Vietnam
SRR5074120	Vietnam
SRR5074122	Vietnam
SRR5074123	Vietnam
SRR5074124	Vietnam
SRR5074125	Vietnam
SRR5074126	Vietnam
SRR5074127	Vietnam
SRR5074128	Vietnam
SRR5074129	Vietnam
SRR5074130	Vietnam
SRR5074131	Vietnam
SRR5074132	Vietnam
SRR5074133	Vietnam
SRR5074134	Vietnam
SRR5074135	Vietnam
SRR5074136	Vietnam
SRR5074137	Vietnam
SRR5074138	Vietnam
SRR5074139	Vietnam
SRR5074140	Vietnam
SRR5074141	Vietnam
SRR5074142	Vietnam
SRR5074143	Vietnam
SRR5074144	Vietnam
SRR5074145	Vietnam
SRR5074146	Vietnam
SRR5074147	Vietnam
SRR5074148	Vietnam
SRR5074149	Vietnam
SRR5074150	Vietnam
SRR5074151	Vietnam
SRR5074152	Vietnam
SRR5074153	Vietnam
SRR5074154	Vietnam
SRR5074155	Vietnam
SRR5074156	Vietnam
SRR5074157	Vietnam
SRR5074158	Vietnam
SRR5074159	Vietnam
SRR5074160	Vietnam
SRR5074161	Vietnam
SRR5074162	Vietnam
SRR5074163	Vietnam
SRR5074164	Vietnam
SRR5074165	Vietnam
SRR5074166	Vietnam
SRR5074167	Vietnam
SRR5074168	Vietnam
SRR5074169	Vietnam

SRR5074170	Vietnam
SRR5074171	Vietnam
SRR5074172	Vietnam
SRR5074173	Vietnam
SRR5074174	Vietnam
SRR5074175	Vietnam
SRR5074176	Vietnam
SRR5074177	Vietnam
SRR5074178	Vietnam
SRR5074179	Vietnam
SRR5074180	Vietnam
SRR5074181	Vietnam
SRR5074182	Vietnam
SRR5074183	Vietnam
SRR5074184	Vietnam
SRR5074185	Vietnam
SRR5074187	Vietnam
SRR5074188	Vietnam
SRR5074189	Vietnam
SRR5074190	Vietnam
SRR5074191	Vietnam
SRR5074192	Vietnam
SRR5074193	Vietnam
SRR5074294	Netherlands
SRR5074709	Netherlands
SRR5074710	Netherlands
SRR5074711	Netherlands
SRR5074712	Netherlands
SRR5074713	Netherlands
SRR5110822	Denmark
SRR5114017	Thailand
SRR5114018	Thailand
SRR5114019	Thailand
SRR5114020	Thailand
SRR5114021	Thailand
SRR5114022	Thailand
SRR5125074	India
SRR5125075	India
SRR5125077	India
SRR5125078	India
SRR5149238	Myanmar
SRR5152895	Georgia
SRR5152896	Georgia
SRR5152897	Georgia
SRR5152898	Georgia
SRR5152901	Georgia
SRR5152902	Georgia
SRR5152903	Georgia
SRR5152904	Georgia
SRR5152905	Georgia

SRR5152906	Georgia
SRR5152907	Georgia
SRR5152908	Georgia
SRR5152909	Georgia
SRR5152910	Georgia
SRR5152912	Georgia
SRR5152914	Georgia
SRR5152915	Georgia
SRR5152916	Georgia
SRR5152917	Georgia
SRR5152918	Georgia
SRR5152919	Georgia
SRR5152920	Georgia
SRR5152921	Georgia
SRR5152922	Georgia
SRR5152923	Georgia
SRR5152924	Georgia
SRR5152925	Georgia
SRR5152926	Georgia
SRR5152927	Georgia
SRR5152928	Georgia
SRR5152929	Georgia
SRR5152930	Georgia
SRR5152936	Georgia
SRR5152937	Georgia
SRR5152938	Georgia
SRR5152939	Georgia
SRR5152940	Azerbaijan
SRR5152941	Georgia
SRR5152942	Azerbaijan
SRR5152943	Azerbaijan
SRR5152944	Azerbaijan
SRR5152945	Azerbaijan
SRR5152946	Azerbaijan
SRR5152947	Azerbaijan
SRR5152948	Azerbaijan
SRR5152949	Azerbaijan
SRR5152950	Azerbaijan
SRR5152951	Georgia
SRR5152952	Azerbaijan
SRR5152953	Georgia
SRR5152954	Georgia
SRR5152956	Georgia
SRR5152957	Azerbaijan
SRR5152958	Azerbaijan
SRR5152959	Georgia
SRR5152963	Azerbaijan
SRR5152973	Azerbaijan
SRR5153032	Azerbaijan
SRR5153072	Azerbaijan

SRR5153073	Azerbaijan
SRR5153074	Azerbaijan
SRR5153075	Azerbaijan
SRR5153076	Azerbaijan
SRR5153077	Azerbaijan
SRR5153078	Azerbaijan
SRR5153079	Georgia
SRR5153080	Azerbaijan
SRR5153081	Georgia
SRR5153082	Azerbaijan
SRR5153083	Azerbaijan
SRR5153084	Azerbaijan
SRR5153085	Azerbaijan
SRR5153086	Azerbaijan
SRR5153087	Azerbaijan
SRR5153088	Georgia
SRR5153089	Georgia
SRR5153090	Georgia
SRR5153091	Georgia
SRR5153092	Georgia
SRR5153093	Georgia
SRR5153094	Georgia
SRR5153095	Georgia
SRR5153131	Georgia
SRR5153132	Georgia
SRR5153134	Georgia
SRR5153194	Georgia
SRR5153206	Georgia
SRR5153208	Georgia
SRR5153213	Georgia
SRR5153214	Georgia
SRR5153215	Georgia
SRR5153216	Georgia
SRR5153217	Georgia
SRR5153218	Georgia
SRR5153219	Georgia
SRR5153220	Georgia
SRR5153221	Georgia
SRR5153222	Georgia
SRR5153223	Georgia
SRR5153224	Georgia
SRR5153225	Georgia
SRR5153226	Georgia
SRR5153227	Georgia
SRR5153228	Georgia
SRR5153229	Georgia
SRR5153230	Georgia
SRR5153231	Georgia
SRR5153232	Georgia
SRR5153233	Georgia

SRR5153234	Georgia
SRR5153235	Georgia
SRR5153236	Georgia
SRR5153237	Georgia
SRR5153240	Georgia
SRR5153242	Georgia
SRR5153245	Georgia
SRR5153252	Georgia
SRR5153253	Georgia
SRR5153254	Georgia
SRR5153255	Georgia
SRR5153256	Georgia
SRR5153257	Georgia
SRR5153258	Georgia
SRR5153259	Georgia
SRR5153261	Georgia
SRR5153262	Georgia
SRR5153263	Georgia
SRR5153264	Georgia
SRR5153265	Georgia
SRR5153266	Georgia
SRR5153267	Georgia
SRR5153268	Georgia
SRR5153269	Georgia
SRR5153270	Georgia
SRR5153271	Georgia
SRR5153272	Georgia
SRR5153273	Georgia
SRR5153274	Georgia
SRR5153275	Georgia
SRR5153276	Georgia
SRR5153278	Georgia
SRR5153279	Georgia
SRR5153291	Georgia
SRR5153303	Georgia
SRR5153307	Georgia
SRR5153308	Georgia
SRR5153309	Georgia
SRR5153310	Georgia
SRR5153311	Georgia
SRR5153312	Georgia
SRR5153313	Georgia
SRR5153314	Georgia
SRR5153315	Georgia
SRR5153316	Georgia
SRR5153317	Georgia
SRR5153318	Georgia
SRR5153319	Georgia
SRR5153320	Georgia
SRR5153321	Georgia

SRR5153322	Georgia
SRR5153323	Georgia
SRR5153324	Georgia
SRR5153325	Georgia
SRR5153326	Georgia
SRR5153327	Georgia
SRR5153328	Georgia
SRR5153329	Georgia
SRR5153330	Georgia
SRR5153331	Georgia
SRR5153332	Georgia
SRR5153333	Georgia
SRR5153334	Georgia
SRR5153335	Georgia
SRR5153336	Georgia
SRR5153423	Georgia
SRR5153424	Georgia
SRR5153509	Georgia
SRR5153594	Azerbaijan
SRR5153595	Georgia
SRR5153596	Georgia
SRR5153597	Azerbaijan
SRR5153598	Azerbaijan
SRR5153599	Azerbaijan
SRR5153600	Azerbaijan
SRR5153601	Georgia
SRR5153602	Georgia
SRR5153603	Azerbaijan
SRR5153604	Azerbaijan
SRR5153605	Azerbaijan
SRR5153606	Azerbaijan
SRR5153607	Azerbaijan
SRR5153608	Azerbaijan
SRR5153609	Georgia
SRR5153610	Azerbaijan
SRR5153611	Georgia
SRR5153612	Azerbaijan
SRR5153613	Georgia
SRR5153614	Georgia
SRR5153615	Azerbaijan
SRR5153616	Georgia
SRR5153617	Azerbaijan
SRR5153618	Azerbaijan
SRR5153619	Georgia
SRR5153620	Azerbaijan
SRR5153621	Georgia
SRR5153622	Azerbaijan
SRR5153707	Azerbaijan
SRR5153708	Azerbaijan
SRR5153709	Azerbaijan

SRR5153710	Azerbaijan
SRR5153711	Azerbaijan
SRR5153712	Azerbaijan
SRR5153713	Azerbaijan
SRR5153716	Azerbaijan
SRR5153717	Azerbaijan
SRR5153718	Azerbaijan
SRR5153719	Azerbaijan
SRR5153720	Azerbaijan
SRR5153721	Azerbaijan
SRR5153722	Azerbaijan
SRR5153723	Azerbaijan
SRR5153808	Azerbaijan
SRR5153809	Azerbaijan
SRR5153810	Azerbaijan
SRR5153811	Azerbaijan
SRR5153812	Azerbaijan
SRR5153813	Azerbaijan
SRR5153814	Azerbaijan
SRR5153815	Azerbaijan
SRR5153816	Moldova
SRR5153817	Moldova
SRR5153818	Moldova
SRR5153819	Moldova
SRR5153820	Azerbaijan
SRR5153821	Moldova
SRR5153822	Moldova
SRR5153823	Moldova
SRR5153824	Moldova
SRR5153825	Azerbaijan
SRR5153826	Azerbaijan
SRR5153827	Moldova
SRR5153828	Moldova
SRR5153829	Azerbaijan
SRR5153830	Moldova
SRR5153831	Moldova
SRR5153832	Moldova
SRR5153833	Moldova
SRR5153834	Moldova
SRR5153835	Moldova
SRR5153836	Moldova
SRR5153837	Moldova
SRR5153838	Moldova
SRR5153839	Moldova
SRR5153840	Moldova
SRR5153841	Moldova
SRR5153842	Moldova
SRR5153843	Moldova
SRR5153844	Moldova
SRR5153845	Moldova

SRR5153846	Moldova
SRR5153847	Moldova
SRR5153848	Moldova
SRR5153849	Moldova
SRR5153850	Moldova
SRR5153851	Moldova
SRR5153852	Moldova
SRR5153853	Moldova
SRR5153854	Moldova
SRR5153855	Moldova
SRR5153856	Moldova
SRR5153857	Moldova
SRR5153858	Moldova
SRR5153859	Moldova
SRR5153860	Moldova
SRR5153861	Moldova
SRR5153862	Moldova
SRR5153863	Moldova
SRR5153864	Moldova
SRR5153865	Moldova
SRR5153866	Moldova
SRR5153867	Moldova
SRR5153868	Moldova
SRR5153869	Moldova
SRR5153870	Moldova
SRR5153877	Moldova
SRR5153878	Moldova
SRR5153879	Moldova
SRR5153880	Moldova
SRR5153881	Moldova
SRR5153882	Moldova
SRR5153883	Moldova
SRR5153884	Moldova
SRR5153885	Moldova
SRR5153886	Moldova
SRR5153887	Moldova
SRR5153888	Moldova
SRR5153900	Moldova
SRR5153901	Moldova
SRR5153902	Moldova
SRR5153903	Moldova
SRR5153904	Moldova
SRR5153905	Moldova
SRR5153906	Moldova
SRR5153907	Moldova
SRR5153908	Moldova
SRR5153909	Moldova
SRR5153910	Moldova
SRR5153911	Moldova
SRR5153912	Moldova

SRR5153913	Moldova
SRR5153914	Moldova
SRR5153915	Moldova
SRR5153916	Moldova
SRR5153917	Moldova
SRR5153918	Moldova
SRR5153919	Moldova
SRR5153920	Moldova
SRR5153921	Moldova
SRR5153922	Moldova
SRR5153923	Moldova
SRR5153924	Moldova
SRR5153925	Moldova
SRR5153926	Moldova
SRR5153927	Moldova
SRR5153928	Moldova
SRR5153929	Moldova
SRR5153930	Moldova
SRR5163781	Moldova
SRR5266534	NA
SRR5266535	NA
SRR5266536	NA
SRR5266537	NA
SRR5266538	NA
SRR5266539	NA
SRR5266540	Russia
SRR5266541	NA
SRR5266542	NA
SRR5266543	NA
SRR5266544	NA
SRR5266545	NA
SRR5266546	NA
SRR5266547	NA
SRR5266548	NA
SRR5266549	NA
SRR5266550	Russia
SRR5266551	NA
SRR5266552	NA
SRR5266553	NA
SRR5266554	NA
SRR5266555	NA
SRR5266556	NA
SRR5266557	NA
SRR5266558	NA
SRR5266559	NA
SRR5266560	NA
SRR5266561	NA
SRR5266562	NA
SRR5266563	NA
SRR5266564	NA

SRR5266565	NA
SRR5306022	Myanmar
SRR5314267	India
SRR5314268	India
SRR5314269	India
SRR5314270	India
SRR5314271	India
SRR5341194	India
SRR5341195	India
SRR5341196	India
SRR5341197	India
SRR5341198	India
SRR5341199	India
SRR5341200	India
SRR5341201	India
SRR5341202	India
SRR5341203	India
SRR5341204	India
SRR5341205	India
SRR5341206	India
SRR5341207	India
SRR5341208	India
SRR5341209	India
SRR5341210	India
SRR5341211	India
SRR5341212	India
SRR5341213	India
SRR5341214	India
SRR5341215	India
SRR5341216	India
SRR5341217	India
SRR5341218	India
SRR5341219	India
SRR5341220	India
SRR5341221	India
SRR5341222	India
SRR5341223	India
SRR5341224	India
SRR5341225	India
SRR5341226	India
SRR5341227	India
SRR5341228	India
SRR5341229	India
SRR5341230	India
SRR5341231	India
SRR5341232	India
SRR5341233	India
SRR5341234	India
SRR5341235	India
SRR5341236	India

SRR5341237	India
SRR5341238	India
SRR5341239	India
SRR5341240	India
SRR5341241	India
SRR5341242	India
SRR5341243	India
SRR5341244	India
SRR5341245	India
SRR5341246	India
SRR5341247	India
SRR5341248	India
SRR5341249	India
SRR5341250	India
SRR5341251	India
SRR5341252	India
SRR5341253	India
SRR5341254	India
SRR5341255	India
SRR5341256	India
SRR5341257	India
SRR5341258	India
SRR5341259	India
SRR5341260	India
SRR5341261	India
SRR5341262	India
SRR5341263	India
SRR5341264	India
SRR5341265	India
SRR5341266	India
SRR5341267	India
SRR5481411	NA
SRR5481412	NA
SRR5481413	NA
SRR5481414	NA
SRR5481415	NA
SRR5481416	NA
SRR5481417	NA
SRR5481418	NA
SRR5481419	NA
SRR5481420	NA
SRR5481421	NA
SRR5481422	NA
SRR5481423	NA
SRR5481424	NA
SRR5481425	NA
SRR5481426	NA
SRR5481427	NA
SRR5481428	NA
SRR5481429	NA

SRR5481430	NA
SRR5481431	NA
SRR5481432	NA
SRR5481433	NA
SRR5481434	NA
SRR5481435	NA
SRR5481436	NA
SRR5481437	NA
SRR5481438	NA
SRR5481439	NA
SRR5481440	NA
SRR5481441	NA
SRR5481442	NA
SRR5481443	NA
SRR5481444	NA
SRR5481445	NA
SRR5481446	NA
SRR5520557	Papua New Guinea
SRR5520572	Papua New Guinea
SRR5520573	Papua New Guinea
SRR5520575	Papua New Guinea
SRR5520594	Papua New Guinea
SRR5520596	Papua New Guinea
SRR5520597	Papua New Guinea
SRR5520599	Papua New Guinea
SRR5520600	Papua New Guinea
SRR5520603	Papua New Guinea
SRR5520605	Papua New Guinea
SRR5520607	Papua New Guinea
SRR5520608	Papua New Guinea
SRR5520609	Papua New Guinea
SRR5520615	Papua New Guinea
SRR5525980	Papua New Guinea
SRR5525981	Papua New Guinea
SRR5525982	Papua New Guinea
SRR5525983	Papua New Guinea
SRR5525984	Papua New Guinea
SRR5525985	Papua New Guinea
SRR5525986	Papua New Guinea
SRR5525987	Papua New Guinea
SRR5525995	Papua New Guinea
SRR5525996	Papua New Guinea
SRR5525997	Papua New Guinea
SRR5525998	Papua New Guinea
SRR5526019	Papua New Guinea
SRR5526143	Papua New Guinea
SRR5526157	Papua New Guinea
SRR5526158	Papua New Guinea
SRR5526332	Papua New Guinea
SRR5526351	Papua New Guinea

SRR5526360	Papua New Guinea
SRR5526639	Papua New Guinea
SRR5526642	Papua New Guinea
SRR5526696	Papua New Guinea
SRR5526775	Papua New Guinea
SRR5526782	Papua New Guinea
SRR5526784	Papua New Guinea
SRR5526785	Papua New Guinea
SRR5526787	Papua New Guinea
SRR5526788	Papua New Guinea
SRR5526839	Papua New Guinea
SRR5526841	Papua New Guinea
SRR5526842	Papua New Guinea
SRR5526843	Papua New Guinea
SRR5526844	Papua New Guinea
SRR5526845	Papua New Guinea
SRR5526846	Papua New Guinea
SRR5526865	Papua New Guinea
SRR5526903	Papua New Guinea
SRR5526904	Papua New Guinea
SRR5526905	Papua New Guinea
SRR5526915	Papua New Guinea
SRR5526916	Papua New Guinea
SRR5526917	Papua New Guinea
SRR5526922	Papua New Guinea
SRR5527175	Papua New Guinea
SRR5527176	Papua New Guinea
SRR5527177	Papua New Guinea
SRR5527178	Papua New Guinea
SRR5527179	Papua New Guinea
SRR5527196	Papua New Guinea
SRR5527198	Papua New Guinea
SRR5527199	Papua New Guinea
SRR5527200	Papua New Guinea
SRR5527201	Papua New Guinea
SRR5527202	Papua New Guinea
SRR5533129	Papua New Guinea
SRR5533130	Papua New Guinea
SRR5533131	Papua New Guinea
SRR5533143	Papua New Guinea
SRR5533151	Papua New Guinea
SRR5533152	Papua New Guinea
SRR5533153	Papua New Guinea
SRR5533176	Papua New Guinea
SRR5533179	Papua New Guinea
SRR5533249	Papua New Guinea
SRR5535697	Peru
SRR5535698	Peru
SRR5535699	Peru
SRR5535701	Peru

SRR5535703	Peru
SRR5535704	South Africa
SRR5535705	Peru
SRR5535707	Peru
SRR5535709	Peru
SRR5535711	Peru
SRR5535775	Cote d'Ivoire
SRR5535777	Peru
SRR5535782	Cote d'Ivoire
SRR5536057	Papua New Guinea
SRR5548263	Papua New Guinea
SRR5549699	Papua New Guinea
SRR5550401	Papua New Guinea
SRR5550936	Papua New Guinea
SRR5551225	Papua New Guinea
SRR5551477	Papua New Guinea
SRR5551664	Papua New Guinea
SRR5551666	Papua New Guinea
SRR5551667	Papua New Guinea
SRR5551764	Papua New Guinea
SRR5552263	Papua New Guinea
SRR5552376	Papua New Guinea
SRR5552377	Papua New Guinea
SRR5552415	Papua New Guinea
SRR5552458	Papua New Guinea
SRR5552459	Papua New Guinea
SRR5552560	Papua New Guinea
SRR5552561	Papua New Guinea
SRR5552562	Papua New Guinea
SRR5552663	Papua New Guinea
SRR5709738	Thailand
SRR5709739	Thailand
SRR5709740	Thailand
SRR5709741	Thailand
SRR5709742	Thailand
SRR5709743	Thailand
SRR5709744	Thailand
SRR5709745	Thailand
SRR5709746	Thailand
SRR5709747	Thailand
SRR5709748	Thailand
SRR5709749	Thailand
SRR5709750	Thailand
SRR5709751	Thailand
SRR5709752	Thailand
SRR5709753	Thailand
SRR5709754	Thailand
SRR5709755	Thailand
SRR5709756	Thailand
SRR5709757	Thailand

SRR5709758	Thailand
SRR5709759	Thailand
SRR5709760	Thailand
SRR5709761	Thailand
SRR5709762	Thailand
SRR5709763	Thailand
SRR5709764	Thailand
SRR5709765	Thailand
SRR5709766	Thailand
SRR5709767	Thailand
SRR5709768	Thailand
SRR5709769	Thailand
SRR5709770	Thailand
SRR5709771	Thailand
SRR5709772	Thailand
SRR5709773	Thailand
SRR5709775	Thailand
SRR5709776	Thailand
SRR5709777	Thailand
SRR5709778	Thailand
SRR5709779	Thailand
SRR5709780	Thailand
SRR5709781	Thailand
SRR5709782	Thailand
SRR5709783	Thailand
SRR5709784	Thailand
SRR5709785	Thailand
SRR5709786	Thailand
SRR5709787	Thailand
SRR5709788	Thailand
SRR5709789	Thailand
SRR5709790	Thailand
SRR5709791	Thailand
SRR5709792	Thailand
SRR5709793	Thailand
SRR5709794	Thailand
SRR5709795	Thailand
SRR5709796	Thailand
SRR5709797	Thailand
SRR5709798	Thailand
SRR5709799	Thailand
SRR5709800	Thailand
SRR5709801	Thailand
SRR5709802	Thailand
SRR5709803	Thailand
SRR5709804	Thailand
SRR5709805	Thailand
SRR5709806	Thailand
SRR5709807	Thailand
SRR5709808	Thailand

SRR5709809	Thailand
SRR5709810	Thailand
SRR5709811	Thailand
SRR5709812	Thailand
SRR5709813	Thailand
SRR5709814	Thailand
SRR5709815	Thailand
SRR5709816	Thailand
SRR5709817	Thailand
SRR5709818	Thailand
SRR5709819	Thailand
SRR5709820	Thailand
SRR5709821	Thailand
SRR5709822	Thailand
SRR5709823	Thailand
SRR5709824	Thailand
SRR5709825	Thailand
SRR5709826	Thailand
SRR5709827	Thailand
SRR5709828	Thailand
SRR5709829	Thailand
SRR5709830	Thailand
SRR5709831	Thailand
SRR5709832	Thailand
SRR5709833	Thailand
SRR5709834	Thailand
SRR5709835	Thailand
SRR5709836	Thailand
SRR5709837	Thailand
SRR5709838	Thailand
SRR5709839	Thailand
SRR5709840	Thailand
SRR5709841	Thailand
SRR5709842	Thailand
SRR5709843	Thailand
SRR5709844	Thailand
SRR5709845	Thailand
SRR5709846	Thailand
SRR5709847	Thailand
SRR5709848	Thailand
SRR5709849	Thailand
SRR5709850	Thailand
SRR5709852	Thailand
SRR5709853	Thailand
SRR5709854	Thailand
SRR5709855	Thailand
SRR5709856	Thailand
SRR5709857	Thailand
SRR5709858	Thailand
SRR5709859	Thailand

SRR5709860	Thailand
SRR5709861	Thailand
SRR5709863	Thailand
SRR5709864	Thailand
SRR5709865	Thailand
SRR5709866	Thailand
SRR5709868	Thailand
SRR5709870	Thailand
SRR5709871	Thailand
SRR5709872	Thailand
SRR5709873	Thailand
SRR5709874	Thailand
SRR5709875	Thailand
SRR5709876	Thailand
SRR5709877	Thailand
SRR5709878	Thailand
SRR5709879	Thailand
SRR5709880	Thailand
SRR5709881	Thailand
SRR5709882	Thailand
SRR5709883	Thailand
SRR5709884	Thailand
SRR5709885	Thailand
SRR5709886	Thailand
SRR5709887	Thailand
SRR5709888	Thailand
SRR5709889	Thailand
SRR5709890	Thailand
SRR5709891	Thailand
SRR5709892	Thailand
SRR5709893	Thailand
SRR5709894	Thailand
SRR5709895	Thailand
SRR5709896	Thailand
SRR5709897	Thailand
SRR5709898	Thailand
SRR5709899	Thailand
SRR5709900	Thailand
SRR5709901	Thailand
SRR5709902	Thailand
SRR5709903	Thailand
SRR5709904	Thailand
SRR5709905	Thailand
SRR5709906	Thailand
SRR5709907	Thailand
SRR5709908	Thailand
SRR5709909	Thailand
SRR5709910	Thailand
SRR5709911	Thailand
SRR5709912	Thailand

SRR5709913	Thailand
SRR5709914	Thailand
SRR5709915	Thailand
SRR5709916	Thailand
SRR5709917	Thailand
SRR5709918	Thailand
SRR5709919	Thailand
SRR5709920	Thailand
SRR5709921	Thailand
SRR5709922	Thailand
SRR5709923	Thailand
SRR5709924	Thailand
SRR5709925	Thailand
SRR5709926	Thailand
SRR5709927	Thailand
SRR5709928	Thailand
SRR5709929	Thailand
SRR5709930	Thailand
SRR5709931	Thailand
SRR5709932	Thailand
SRR5709933	Thailand
SRR5709934	Thailand
SRR5709935	Thailand
SRR5709936	Thailand
SRR5709937	Thailand
SRR5709938	Thailand
SRR5709939	Thailand
SRR5709940	Thailand
SRR5709941	Thailand
SRR5709942	Thailand
SRR5709944	Thailand
SRR5709945	Thailand
SRR5709946	Thailand
SRR5709947	Thailand
SRR5709948	Thailand
SRR5709950	Thailand
SRR5709951	Thailand
SRR5709953	Thailand
SRR5709954	Thailand
SRR5709955	Thailand
SRR5709956	Thailand
SRR5709957	Thailand
SRR5709958	Thailand
SRR5709959	Thailand
SRR5709961	Thailand
SRR5709962	Thailand
SRR5709963	Thailand
SRR5709964	Thailand
SRR5709965	Thailand
SRR5709966	Thailand

SRR5709967	Thailand
SRR5709968	Thailand
SRR5709969	Thailand
SRR5709970	Thailand
SRR5709971	Thailand
SRR5709972	Thailand
SRR5709973	Thailand
SRR5709974	Thailand
SRR5709975	Thailand
SRR5709976	Thailand
SRR5709978	Thailand
SRR5709979	Thailand
SRR5709980	Thailand
SRR5709981	Thailand
SRR5709982	Thailand
SRR5709983	Thailand
SRR5709984	Thailand
SRR5709985	Thailand
SRR5709986	Thailand
SRR5709987	Thailand
SRR5709988	Thailand
SRR5709989	Thailand
SRR5709990	Thailand
SRR5709991	Thailand
SRR5709992	Thailand
SRR5709993	Thailand
SRR5709994	Thailand
SRR5709995	Thailand
SRR5709996	Thailand
SRR5709997	Thailand
SRR5709998	Thailand
SRR5709999	Thailand
SRR5710000	Thailand
SRR5710001	Thailand
SRR5710003	Thailand
SRR5710004	Thailand
SRR5710005	Thailand
SRR5710006	Thailand
SRR5710007	Thailand
SRR5710008	Thailand
SRR5710010	Thailand
SRR5710011	Thailand
SRR5710012	Thailand
SRR5710013	Thailand
SRR5710014	Thailand
SRR5710015	Thailand
SRR5710016	Thailand
SRR5710017	Thailand
SRR5710018	Thailand
SRR5710019	Thailand

SRR5710020	Thailand
SRR5710021	Thailand
SRR5710022	Thailand
SRR5710024	Thailand
SRR5710026	Thailand
SRR5710027	Thailand
SRR5710028	Thailand
SRR5710029	Thailand
SRR5710030	Thailand
SRR5808261	Canada
SRR5808262	Canada
SRR5817462	Australia
SRR5817463	Australia
SRR5817464	Australia
SRR5817465	Australia
SRR5817466	Australia
SRR5817467	Australia
SRR5817468	Australia
SRR5817469	Australia
SRR5817470	Australia
SRR5817471	Australia
SRR5817472	Australia
SRR5817473	Australia
SRR5817474	Australia
SRR5817475	Australia
SRR5817476	Australia
SRR5817477	Australia
SRR5817478	Australia
SRR5817479	Australia
SRR5817480	Australia
SRR5817481	Australia
SRR5818385	Djibouti
SRR5818389	Djibouti
SRR5818390	Djibouti
SRR5818391	Djibouti
SRR5818393	Djibouti
SRR5818394	Djibouti
SRR5818395	Djibouti
SRR5818396	Djibouti
SRR5818397	Djibouti
SRR5818398	Djibouti
SRR5818399	Djibouti
SRR5818400	Djibouti
SRR5818403	Djibouti
SRR5818405	Djibouti
SRR5818406	Djibouti
SRR5818408	Djibouti
SRR5818411	Djibouti
SRR5818415	Djibouti
SRR5818425	Djibouti

SRR5818426	Djibouti
SRR5818428	Djibouti
SRR5818429	Djibouti
SRR5818430	Djibouti
SRR5818431	Djibouti
SRR5818432	Djibouti
SRR5818433	Djibouti
SRR5818435	Djibouti
SRR5818437	Djibouti
SRR5818439	Djibouti
SRR5818440	Djibouti
SRR5818441	Djibouti
SRR5818442	Djibouti
SRR5818443	Djibouti
SRR5818444	Djibouti
SRR5818445	Djibouti
SRR5818447	Djibouti
SRR5818451	Djibouti
SRR5818455	Djibouti
SRR5818459	Djibouti
SRR5818461	Djibouti
SRR5818462	Djibouti
SRR5818464	Djibouti
SRR5818465	Djibouti
SRR5818467	Djibouti
SRR5818468	Djibouti
SRR5818470	Djibouti
SRR5818568	Djibouti
SRR5818569	Djibouti
SRR5818571	Djibouti
SRR5818573	Djibouti
SRR5818574	Djibouti
SRR5818576	Djibouti
SRR5818586	Djibouti
SRR5818588	Djibouti
SRR5818595	Djibouti
SRR5818600	Djibouti
SRR5818601	Djibouti
SRR5818603	Djibouti
SRR5818607	Djibouti
SRR5818608	Djibouti
SRR5818609	Djibouti
SRR5818612	Djibouti
SRR5818619	Djibouti
SRR5818620	Djibouti
SRR5818629	Djibouti
SRR5818634	Djibouti
SRR5818635	Djibouti
SRR5818636	Djibouti
SRR5818637	Djibouti

SRR5818638	Djibouti
SRR5818641	Djibouti
SRR5818646	Djibouti
SRR5818647	Djibouti
SRR5818650	Djibouti
SRR5818653	Djibouti
SRR5818654	Djibouti
SRR5818657	Djibouti
SRR5818658	Djibouti
SRR5818659	Djibouti
SRR5818663	Djibouti
SRR5818666	Djibouti
SRR5818667	Djibouti
SRR5818670	Djibouti
SRR5818673	Djibouti
SRR5818680	Djibouti
SRR5818685	Djibouti
SRR5818687	Djibouti
SRR5818695	Djibouti
SRR5818698	Djibouti
SRR5818699	Djibouti
SRR5818704	Djibouti
SRR5818705	Djibouti
SRR5894881	China
SRR5894882	China
SRR5894883	China
SRR5894884	China
SRR5894885	China
SRR5894886	China
SRR5985664	United States of America
SRR5985665	United States of America
SRR5985667	United States of America
SRR5985668	United States of America
SRR5985671	United States of America
SRR5985672	United States of America
SRR5985674	United States of America
SRR5985676	United States of America
SRR5985681	United States of America
SRR5985682	United States of America
SRR5985683	United States of America
SRR6003544	Australia
SRR6040100	Australia
SRR6040101	Australia
SRR6040103	Australia
SRR6040104	Australia
SRR6040105	Australia
SRR6044768	United Kingdom
SRR6044769	United Kingdom
SRR6044770	United Kingdom
SRR6044772	United Kingdom

SRR6044774	United Kingdom
SRR6044775	United Kingdom
SRR6044778	United Kingdom
SRR6044780	United Kingdom
SRR6044784	United Kingdom
SRR6044787	United Kingdom
SRR6044790	United Kingdom
SRR6044802	United Kingdom
SRR6044803	United Kingdom
SRR6044805	United Kingdom
SRR6044807	United Kingdom
SRR6044814	United Kingdom
SRR6044815	United Kingdom
SRR6044817	United Kingdom
SRR6044819	United Kingdom
SRR6044821	United Kingdom
SRR6044829	United Kingdom
SRR6044830	United Kingdom
SRR6044832	United Kingdom
SRR6044835	United Kingdom
SRR6044837	United Kingdom
SRR6044838	United Kingdom
SRR6044840	United Kingdom
SRR6044841	United Kingdom
SRR6044842	United Kingdom
SRR6044847	United Kingdom
SRR6044852	United Kingdom
SRR6044855	United Kingdom
SRR6044856	United Kingdom
SRR6044857	United Kingdom
SRR6044862	United Kingdom
SRR6044863	United Kingdom
SRR6044867	United Kingdom
SRR6044871	United Kingdom
SRR6044872	United Kingdom
SRR6044873	United Kingdom
SRR6044875	United Kingdom
SRR6044876	United Kingdom
SRR6044882	United Kingdom
SRR6044883	United Kingdom
SRR6044884	United Kingdom
SRR6044887	United Kingdom
SRR6044888	United Kingdom
SRR6044889	United Kingdom
SRR6044890	United Kingdom
SRR6044891	United Kingdom
SRR6044893	United Kingdom
SRR6044896	United Kingdom
SRR6044897	United Kingdom
SRR6044904	United Kingdom

SRR6044905	United Kingdom
SRR6044909	United Kingdom
SRR6044910	United Kingdom
SRR6044911	United Kingdom
SRR6044912	United Kingdom
SRR6044914	United Kingdom
SRR6044916	United Kingdom
SRR6044917	United Kingdom
SRR6044918	United Kingdom
SRR6044924	United Kingdom
SRR6044925	United Kingdom
SRR6044933	United Kingdom
SRR6044936	United Kingdom
SRR6044937	United Kingdom
SRR6044938	United Kingdom
SRR6044940	United Kingdom
SRR6044942	United Kingdom
SRR6044945	United Kingdom
SRR6044946	United Kingdom
SRR6044949	United Kingdom
SRR6044952	United Kingdom
SRR6044955	United Kingdom
SRR6044956	United Kingdom
SRR6044957	United Kingdom
SRR6044958	United Kingdom
SRR6044959	United Kingdom
SRR6044963	United Kingdom
SRR6044964	United Kingdom
SRR6044971	United Kingdom
SRR6044975	United Kingdom
SRR6044976	United Kingdom
SRR6044977	United Kingdom
SRR6044978	United Kingdom
SRR6044981	United Kingdom
SRR6044982	United Kingdom
SRR6044983	United Kingdom
SRR6044985	United Kingdom
SRR6044987	United Kingdom
SRR6044989	United Kingdom
SRR6044991	United Kingdom
SRR6044993	United Kingdom
SRR6044999	United Kingdom
SRR6045000	United Kingdom
SRR6045002	United Kingdom
SRR6045003	United Kingdom
SRR6045004	United Kingdom
SRR6045006	United Kingdom
SRR6045010	United Kingdom
SRR6045012	United Kingdom
SRR6045014	United Kingdom

SRR6045017	United Kingdom
SRR6045018	United Kingdom
SRR6045022	United Kingdom
SRR6045024	United Kingdom
SRR6045028	United Kingdom
SRR6045033	United Kingdom
SRR6045034	United Kingdom
SRR6045036	United Kingdom
SRR6045039	United Kingdom
SRR6045043	United Kingdom
SRR6045045	United Kingdom
SRR6045046	United Kingdom
SRR6045050	United Kingdom
SRR6045052	United Kingdom
SRR6045053	United Kingdom
SRR6045056	United Kingdom
SRR6045057	United Kingdom
SRR6045058	United Kingdom
SRR6045063	United Kingdom
SRR6045064	United Kingdom
SRR6045065	United Kingdom
SRR6045071	United Kingdom
SRR6045072	United Kingdom
SRR6045073	United Kingdom
SRR6045074	United Kingdom
SRR6045085	United Kingdom
SRR6045086	United Kingdom
SRR6045089	United Kingdom
SRR6045092	United Kingdom
SRR6045093	United Kingdom
SRR6045097	United Kingdom
SRR6045099	United Kingdom
SRR6045102	United Kingdom
SRR6045104	United Kingdom
SRR6045105	United Kingdom
SRR6045106	United Kingdom
SRR6045108	United Kingdom
SRR6045109	United Kingdom
SRR6045110	United Kingdom
SRR6045116	United Kingdom
SRR6045119	United Kingdom
SRR6045123	United Kingdom
SRR6045124	United Kingdom
SRR6045125	United Kingdom
SRR6045128	United Kingdom
SRR6045131	United Kingdom
SRR6045133	United Kingdom
SRR6045135	United Kingdom
SRR6045138	United Kingdom
SRR6045141	United Kingdom

SRR6045142	United Kingdom
SRR6045144	United Kingdom
SRR6045145	United Kingdom
SRR6045146	United Kingdom
SRR6045148	United Kingdom
SRR6045149	United Kingdom
SRR6045150	United Kingdom
SRR6045155	United Kingdom
SRR6045158	United Kingdom
SRR6045159	United Kingdom
SRR6045162	United Kingdom
SRR6045163	United Kingdom
SRR6045164	United Kingdom
SRR6045165	United Kingdom
SRR6045168	United Kingdom
SRR6045169	United Kingdom
SRR6045172	United Kingdom
SRR6045173	United Kingdom
SRR6045174	United Kingdom
SRR6045175	United Kingdom
SRR6045177	United Kingdom
SRR6045178	United Kingdom
SRR6045181	United Kingdom
SRR6045185	United Kingdom
SRR6045186	United Kingdom
SRR6045189	United Kingdom
SRR6045191	United Kingdom
SRR6045197	United Kingdom
SRR6045198	United Kingdom
SRR6045199	United Kingdom
SRR6045200	United Kingdom
SRR6045201	United Kingdom
SRR6045205	United Kingdom
SRR6045206	United Kingdom
SRR6045209	United Kingdom
SRR6045210	United Kingdom
SRR6045211	United Kingdom
SRR6045217	United Kingdom
SRR6045218	United Kingdom
SRR6045219	United Kingdom
SRR6045222	United Kingdom
SRR6045230	United Kingdom
SRR6045232	United Kingdom
SRR6045233	United Kingdom
SRR6045237	United Kingdom
SRR6045238	United Kingdom
SRR6045240	United Kingdom
SRR6045241	United Kingdom
SRR6045242	United Kingdom
SRR6045243	United Kingdom

SRR6045244	United Kingdom
SRR6045245	United Kingdom
SRR6045247	United Kingdom
SRR6045248	United Kingdom
SRR6045249	United Kingdom
SRR6045255	United Kingdom
SRR6045256	United Kingdom
SRR6045261	United Kingdom
SRR6045263	United Kingdom
SRR6045264	United Kingdom
SRR6045265	United Kingdom
SRR6045267	United Kingdom
SRR6045271	United Kingdom
SRR6045277	United Kingdom
SRR6045279	United Kingdom
SRR6045281	United Kingdom
SRR6045283	United Kingdom
SRR6045284	United Kingdom
SRR6045285	United Kingdom
SRR6045288	United Kingdom
SRR6045289	United Kingdom
SRR6045290	United Kingdom
SRR6045291	United Kingdom
SRR6045292	United Kingdom
SRR6045293	United Kingdom
SRR6045294	United Kingdom
SRR6045295	United Kingdom
SRR6045298	United Kingdom
SRR6045304	United Kingdom
SRR6045308	United Kingdom
SRR6045309	United Kingdom
SRR6045311	United Kingdom
SRR6045312	United Kingdom
SRR6045314	United Kingdom
SRR6045316	United Kingdom
SRR6045320	United Kingdom
SRR6045321	United Kingdom
SRR6045323	United Kingdom
SRR6045325	United Kingdom
SRR6045326	United Kingdom
SRR6045327	United Kingdom
SRR6045331	United Kingdom
SRR6045332	United Kingdom
SRR6045333	United Kingdom
SRR6045335	United Kingdom
SRR6045336	United Kingdom
SRR6045338	United Kingdom
SRR6045341	United Kingdom
SRR6045342	United Kingdom
SRR6045343	United Kingdom

SRR6045347	United Kingdom
SRR6045348	United Kingdom
SRR6045350	United Kingdom
SRR6045351	United Kingdom
SRR6045356	United Kingdom
SRR6045358	United Kingdom
SRR6045359	United Kingdom
SRR6045362	United Kingdom
SRR6045365	United Kingdom
SRR6045366	United Kingdom
SRR6045367	United Kingdom
SRR6045369	United Kingdom
SRR6045371	United Kingdom
SRR6045376	United Kingdom
SRR6045379	United Kingdom
SRR6045380	United Kingdom
SRR6045381	United Kingdom
SRR6045384	United Kingdom
SRR6045386	United Kingdom
SRR6045387	United Kingdom
SRR6045388	United Kingdom
SRR6045391	United Kingdom
SRR6045396	United Kingdom
SRR6045399	United Kingdom
SRR6045400	United Kingdom
SRR6045409	United Kingdom
SRR6045414	United Kingdom
SRR6045415	United Kingdom
SRR6045417	United Kingdom
SRR6045418	United Kingdom
SRR6045419	United Kingdom
SRR6045420	United Kingdom
SRR6045421	United Kingdom
SRR6045422	United Kingdom
SRR6045423	United Kingdom
SRR6045424	United Kingdom
SRR6045430	United Kingdom
SRR6045432	United Kingdom
SRR6045433	United Kingdom
SRR6045438	United Kingdom
SRR6045439	United Kingdom
SRR6045441	United Kingdom
SRR6045445	United Kingdom
SRR6045447	United Kingdom
SRR6045448	United Kingdom
SRR6045449	United Kingdom
SRR6045450	United Kingdom
SRR6045453	United Kingdom
SRR6045455	United Kingdom
SRR6045458	United Kingdom

SRR6045460	United Kingdom
SRR6045461	United Kingdom
SRR6045462	United Kingdom
SRR6045464	United Kingdom
SRR6045465	United Kingdom
SRR6045466	United Kingdom
SRR6045467	United Kingdom
SRR6045470	United Kingdom
SRR6045472	United Kingdom
SRR6045473	United Kingdom
SRR6045474	United Kingdom
SRR6045476	United Kingdom
SRR6045480	United Kingdom
SRR6045481	United Kingdom
SRR6045482	United Kingdom
SRR6045485	United Kingdom
SRR6045486	United Kingdom
SRR6045492	United Kingdom
SRR6045495	United Kingdom
SRR6045502	United Kingdom
SRR6045503	United Kingdom
SRR6045505	United Kingdom
SRR6045507	United Kingdom
SRR6045516	United Kingdom
SRR6045519	United Kingdom
SRR6045520	United Kingdom
SRR6045521	United Kingdom
SRR6045523	United Kingdom
SRR6045526	United Kingdom
SRR6045529	United Kingdom
SRR6045533	United Kingdom
SRR6045535	United Kingdom
SRR6045536	United Kingdom
SRR6045537	United Kingdom
SRR6045542	United Kingdom
SRR6045543	United Kingdom
SRR6045544	United Kingdom
SRR6045545	United Kingdom
SRR6045547	United Kingdom
SRR6045548	United Kingdom
SRR6045549	United Kingdom
SRR6045552	United Kingdom
SRR6045553	United Kingdom
SRR6045555	United Kingdom
SRR6045556	United Kingdom
SRR6045557	United Kingdom
SRR6045559	United Kingdom
SRR6045563	United Kingdom
SRR6045565	United Kingdom
SRR6045572	United Kingdom

SRR6045574	United Kingdom
SRR6045576	United Kingdom
SRR6045577	United Kingdom
SRR6045579	United Kingdom
SRR6045584	United Kingdom
SRR6045586	United Kingdom
SRR6045587	United Kingdom
SRR6045590	United Kingdom
SRR6045591	United Kingdom
SRR6045593	United Kingdom
SRR6045597	United Kingdom
SRR6045599	United Kingdom
SRR6045600	United Kingdom
SRR6045602	United Kingdom
SRR6045603	United Kingdom
SRR6045608	United Kingdom
SRR6045613	United Kingdom
SRR6045614	United Kingdom
SRR6045616	United Kingdom
SRR6045618	United Kingdom
SRR6045619	United Kingdom
SRR6045620	United Kingdom
SRR6045621	United Kingdom
SRR6045622	United Kingdom
SRR6045625	United Kingdom
SRR6045627	United Kingdom
SRR6045628	United Kingdom
SRR6045634	United Kingdom
SRR6045635	United Kingdom
SRR6045638	United Kingdom
SRR6045639	United Kingdom
SRR6045640	United Kingdom
SRR6045641	United Kingdom
SRR6045642	United Kingdom
SRR6045647	United Kingdom
SRR6045655	United Kingdom
SRR6045660	United Kingdom
SRR6045664	United Kingdom
SRR6045670	United Kingdom
SRR6045671	United Kingdom
SRR6045673	United Kingdom
SRR6045680	United Kingdom
SRR6045683	United Kingdom
SRR6045685	United Kingdom
SRR6045686	United Kingdom
SRR6045688	United Kingdom
SRR6045689	United Kingdom
SRR6045690	United Kingdom
SRR6045691	United Kingdom
SRR6045694	United Kingdom

SRR6045696	United Kingdom
SRR6045699	United Kingdom
SRR6045704	United Kingdom
SRR6045705	United Kingdom
SRR6045706	United Kingdom
SRR6045709	United Kingdom
SRR6045713	United Kingdom
SRR6045714	United Kingdom
SRR6045715	United Kingdom
SRR6045723	United Kingdom
SRR6045726	United Kingdom
SRR6045729	United Kingdom
SRR6045730	United Kingdom
SRR6045731	United Kingdom
SRR6045734	United Kingdom
SRR6045735	United Kingdom
SRR6045736	United Kingdom
SRR6045739	United Kingdom
SRR6045741	United Kingdom
SRR6045742	United Kingdom
SRR6045743	United Kingdom
SRR6045744	United Kingdom
SRR6045746	United Kingdom
SRR6045749	United Kingdom
SRR6045750	United Kingdom
SRR6045752	United Kingdom
SRR6045753	United Kingdom
SRR6045758	United Kingdom
SRR6045759	United Kingdom
SRR6045763	United Kingdom
SRR6045764	United Kingdom
SRR6045767	United Kingdom
SRR6045768	United Kingdom
SRR6045769	United Kingdom
SRR6045774	United Kingdom
SRR6045777	United Kingdom
SRR6045778	United Kingdom
SRR6045779	United Kingdom
SRR6045781	United Kingdom
SRR6045782	United Kingdom
SRR6045783	United Kingdom
SRR6045785	United Kingdom
SRR6045787	United Kingdom
SRR6045790	United Kingdom
SRR6045793	United Kingdom
SRR6045795	United Kingdom
SRR6045796	United Kingdom
SRR6045797	United Kingdom
SRR6045798	United Kingdom
SRR6045799	United Kingdom

SRR6045802	United Kingdom
SRR6045803	United Kingdom
SRR6045805	United Kingdom
SRR6045808	United Kingdom
SRR6045810	United Kingdom
SRR6045814	United Kingdom
SRR6045817	United Kingdom
SRR6045819	United Kingdom
SRR6045824	United Kingdom
SRR6045825	United Kingdom
SRR6045828	United Kingdom
SRR6045831	United Kingdom
SRR6045832	United Kingdom
SRR6045833	United Kingdom
SRR6045835	United Kingdom
SRR6045837	United Kingdom
SRR6045838	United Kingdom
SRR6045841	United Kingdom
SRR6045843	United Kingdom
SRR6045844	United Kingdom
SRR6045846	United Kingdom
SRR6045847	United Kingdom
SRR6045849	United Kingdom
SRR6045855	United Kingdom
SRR6045857	United Kingdom
SRR6045859	United Kingdom
SRR6045860	United Kingdom
SRR6045866	United Kingdom
SRR6045868	United Kingdom
SRR6045870	United Kingdom
SRR6045871	United Kingdom
SRR6045872	United Kingdom
SRR6045873	United Kingdom
SRR6045876	United Kingdom
SRR6045880	United Kingdom
SRR6045882	United Kingdom
SRR6045885	United Kingdom
SRR6045887	United Kingdom
SRR6045890	United Kingdom
SRR6045892	United Kingdom
SRR6045894	United Kingdom
SRR6045896	United Kingdom
SRR6045899	United Kingdom
SRR6045900	United Kingdom
SRR6045905	United Kingdom
SRR6045907	United Kingdom
SRR6045909	United Kingdom
SRR6045910	United Kingdom
SRR6045912	United Kingdom
SRR6045915	United Kingdom

SRR6045918	United Kingdom
SRR6045920	United Kingdom
SRR6045924	United Kingdom
SRR6045925	United Kingdom
SRR6045928	United Kingdom
SRR6045929	United Kingdom
SRR6045930	United Kingdom
SRR6045932	United Kingdom
SRR6045934	United Kingdom
SRR6045936	United Kingdom
SRR6045941	United Kingdom
SRR6045942	United Kingdom
SRR6045944	United Kingdom
SRR6045945	United Kingdom
SRR6045947	United Kingdom
SRR6045948	United Kingdom
SRR6045950	United Kingdom
SRR6045951	United Kingdom
SRR6045952	United Kingdom
SRR6045957	United Kingdom
SRR6045958	United Kingdom
SRR6045966	United Kingdom
SRR6045967	United Kingdom
SRR6045968	United Kingdom
SRR6045971	United Kingdom
SRR6045972	United Kingdom
SRR6045976	United Kingdom
SRR6045978	United Kingdom
SRR6045979	United Kingdom
SRR6045981	United Kingdom
SRR6045984	United Kingdom
SRR6045986	United Kingdom
SRR6045989	United Kingdom
SRR6045991	United Kingdom
SRR6045992	United Kingdom
SRR6045993	United Kingdom
SRR6045997	United Kingdom
SRR6045999	United Kingdom
SRR6046000	United Kingdom
SRR6046002	United Kingdom
SRR6046003	United Kingdom
SRR6046011	United Kingdom
SRR6046015	United Kingdom
SRR6046016	United Kingdom
SRR6046019	United Kingdom
SRR6046020	United Kingdom
SRR6046023	United Kingdom
SRR6046026	United Kingdom
SRR6046030	United Kingdom
SRR6046034	United Kingdom

SRR6046036	United Kingdom
SRR6046037	United Kingdom
SRR6046038	United Kingdom
SRR6046039	United Kingdom
SRR6046040	United Kingdom
SRR6046042	United Kingdom
SRR6046044	United Kingdom
SRR6046046	United Kingdom
SRR6046047	United Kingdom
SRR6046048	United Kingdom
SRR6046050	United Kingdom
SRR6046051	United Kingdom
SRR6046052	United Kingdom
SRR6046057	United Kingdom
SRR6046062	United Kingdom
SRR6046064	United Kingdom
SRR6046065	United Kingdom
SRR6046066	United Kingdom
SRR6046067	United Kingdom
SRR6046072	United Kingdom
SRR6046075	United Kingdom
SRR6046076	United Kingdom
SRR6046077	United Kingdom
SRR6046078	United Kingdom
SRR6046081	United Kingdom
SRR6046082	United Kingdom
SRR6046083	United Kingdom
SRR6046084	United Kingdom
SRR6046087	United Kingdom
SRR6046088	United Kingdom
SRR6046089	United Kingdom
SRR6046090	United Kingdom
SRR6046092	United Kingdom
SRR6046093	United Kingdom
SRR6046095	United Kingdom
SRR6046096	United Kingdom
SRR6046097	United Kingdom
SRR6046098	United Kingdom
SRR6046099	United Kingdom
SRR6046102	United Kingdom
SRR6046105	United Kingdom
SRR6046106	United Kingdom
SRR6046111	United Kingdom
SRR6046112	United Kingdom
SRR6046113	United Kingdom
SRR6046115	United Kingdom
SRR6046117	United Kingdom
SRR6046122	United Kingdom
SRR6046127	United Kingdom
SRR6046130	United Kingdom

SRR6046133	United Kingdom
SRR6046135	United Kingdom
SRR6046137	United Kingdom
SRR6046140	United Kingdom
SRR6046141	United Kingdom
SRR6046145	United Kingdom
SRR6046149	United Kingdom
SRR6046150	United Kingdom
SRR6046151	United Kingdom
SRR6046152	United Kingdom
SRR6046158	United Kingdom
SRR6046159	United Kingdom
SRR6046160	United Kingdom
SRR6046162	United Kingdom
SRR6046164	United Kingdom
SRR6046171	United Kingdom
SRR6046173	United Kingdom
SRR6046176	United Kingdom
SRR6046177	United Kingdom
SRR6046179	United Kingdom
SRR6046181	United Kingdom
SRR6046185	United Kingdom
SRR6046189	United Kingdom
SRR6046190	United Kingdom
SRR6046192	United Kingdom
SRR6046193	United Kingdom
SRR6046196	United Kingdom
SRR6046197	United Kingdom
SRR6046208	United Kingdom
SRR6046210	United Kingdom
SRR6046212	United Kingdom
SRR6046217	United Kingdom
SRR6046219	United Kingdom
SRR6046220	United Kingdom
SRR6046221	United Kingdom
SRR6046222	United Kingdom
SRR6046223	United Kingdom
SRR6046225	United Kingdom
SRR6046226	United Kingdom
SRR6046232	United Kingdom
SRR6046236	United Kingdom
SRR6046237	United Kingdom
SRR6046239	United Kingdom
SRR6046243	United Kingdom
SRR6046245	United Kingdom
SRR6046246	United Kingdom
SRR6046248	United Kingdom
SRR6046250	United Kingdom
SRR6046253	United Kingdom
SRR6046256	United Kingdom

SRR6046258	United Kingdom
SRR6046259	United Kingdom
SRR6046260	United Kingdom
SRR6046262	United Kingdom
SRR6046269	United Kingdom
SRR6046270	United Kingdom
SRR6046273	United Kingdom
SRR6046275	United Kingdom
SRR6046276	United Kingdom
SRR6046278	United Kingdom
SRR6046279	United Kingdom
SRR6046280	United Kingdom
SRR6046281	United Kingdom
SRR6046284	United Kingdom
SRR6046285	United Kingdom
SRR6046287	United Kingdom
SRR6046288	United Kingdom
SRR6046290	United Kingdom
SRR6046291	United Kingdom
SRR6046295	United Kingdom
SRR6046297	United Kingdom
SRR6046299	United Kingdom
SRR6046305	United Kingdom
SRR6046306	United Kingdom
SRR6046308	United Kingdom
SRR6046313	United Kingdom
SRR6046314	United Kingdom
SRR6046320	United Kingdom
SRR6046321	United Kingdom
SRR6046324	United Kingdom
SRR6046329	United Kingdom
SRR6046332	United Kingdom
SRR6046343	United Kingdom
SRR6046351	United Kingdom
SRR6046354	United Kingdom
SRR6046358	United Kingdom
SRR6046359	United Kingdom
SRR6046362	United Kingdom
SRR6046364	United Kingdom
SRR6046369	United Kingdom
SRR6046373	United Kingdom
SRR6046374	United Kingdom
SRR6046376	United Kingdom
SRR6046382	United Kingdom
SRR6046384	United Kingdom
SRR6046385	United Kingdom
SRR6046387	United Kingdom
SRR6046389	United Kingdom
SRR6046391	United Kingdom
SRR6046395	United Kingdom

SRR6046397	United Kingdom
SRR6046398	United Kingdom
SRR6046400	United Kingdom
SRR6046403	United Kingdom
SRR6046405	United Kingdom
SRR6046410	United Kingdom
SRR6046413	United Kingdom
SRR6046415	United Kingdom
SRR6046417	United Kingdom
SRR6046418	United Kingdom
SRR6046420	United Kingdom
SRR6046422	United Kingdom
SRR6046423	United Kingdom
SRR6046426	United Kingdom
SRR6046428	United Kingdom
SRR6046430	United Kingdom
SRR6046431	United Kingdom
SRR6046433	United Kingdom
SRR6046434	United Kingdom
SRR6046435	United Kingdom
SRR6046436	United Kingdom
SRR6046440	United Kingdom
SRR6046442	United Kingdom
SRR6046447	United Kingdom
SRR6046450	United Kingdom
SRR6046454	United Kingdom
SRR6046456	United Kingdom
SRR6046457	United Kingdom
SRR6046458	United Kingdom
SRR6046459	United Kingdom
SRR6046460	United Kingdom
SRR6046461	United Kingdom
SRR6046463	United Kingdom
SRR6046466	United Kingdom
SRR6046475	United Kingdom
SRR6046476	United Kingdom
SRR6046481	United Kingdom
SRR6046482	United Kingdom
SRR6046483	United Kingdom
SRR6046484	United Kingdom
SRR6046485	United Kingdom
SRR6046489	United Kingdom
SRR6046492	United Kingdom
SRR6046494	United Kingdom
SRR6046496	United Kingdom
SRR6046497	United Kingdom
SRR6046504	United Kingdom
SRR6046505	United Kingdom
SRR6046508	United Kingdom
SRR6046509	United Kingdom

SRR6046512	United Kingdom
SRR6046514	United Kingdom
SRR6046515	United Kingdom
SRR6046516	United Kingdom
SRR6046517	United Kingdom
SRR6046518	United Kingdom
SRR6046521	United Kingdom
SRR6046526	United Kingdom
SRR6046528	United Kingdom
SRR6046530	United Kingdom
SRR6046531	United Kingdom
SRR6046534	United Kingdom
SRR6046540	United Kingdom
SRR6046541	United Kingdom
SRR6046544	United Kingdom
SRR6046550	United Kingdom
SRR6046551	United Kingdom
SRR6046553	United Kingdom
SRR6046555	United Kingdom
SRR6046556	United Kingdom
SRR6046558	United Kingdom
SRR6046562	United Kingdom
SRR6046564	United Kingdom
SRR6046565	United Kingdom
SRR6046572	United Kingdom
SRR6046573	United Kingdom
SRR6046574	United Kingdom
SRR6046575	United Kingdom
SRR6046576	United Kingdom
SRR6046577	United Kingdom
SRR6046578	United Kingdom
SRR6046579	United Kingdom
SRR6046583	United Kingdom
SRR6046585	United Kingdom
SRR6046586	United Kingdom
SRR6046587	United Kingdom
SRR6046592	United Kingdom
SRR6046593	United Kingdom
SRR6046594	United Kingdom
SRR6046601	United Kingdom
SRR6046602	United Kingdom
SRR6046603	United Kingdom
SRR6046604	United Kingdom
SRR6046607	United Kingdom
SRR6046608	United Kingdom
SRR6046609	United Kingdom
SRR6046610	United Kingdom
SRR6046611	United Kingdom
SRR6046612	United Kingdom
SRR6046613	United Kingdom

SRR6046616	United Kingdom
SRR6046617	United Kingdom
SRR6046618	United Kingdom
SRR6046624	United Kingdom
SRR6046626	United Kingdom
SRR6046627	United Kingdom
SRR6046628	United Kingdom
SRR6046629	United Kingdom
SRR6046631	United Kingdom
SRR6046632	United Kingdom
SRR6046634	United Kingdom
SRR6046636	United Kingdom
SRR6046639	United Kingdom
SRR6046641	United Kingdom
SRR6046642	United Kingdom
SRR6046644	United Kingdom
SRR6046645	United Kingdom
SRR6046646	United Kingdom
SRR6046649	United Kingdom
SRR6046652	United Kingdom
SRR6046655	United Kingdom
SRR6046656	United Kingdom
SRR6046658	United Kingdom
SRR6046659	United Kingdom
SRR6046662	United Kingdom
SRR6046664	United Kingdom
SRR6046666	United Kingdom
SRR6046668	United Kingdom
SRR6046670	United Kingdom
SRR6046673	United Kingdom
SRR6046679	United Kingdom
SRR6046680	United Kingdom
SRR6046681	United Kingdom
SRR6046683	United Kingdom
SRR6046691	United Kingdom
SRR6046693	United Kingdom
SRR6046697	United Kingdom
SRR6046701	United Kingdom
SRR6046703	United Kingdom
SRR6046706	United Kingdom
SRR6046710	United Kingdom
SRR6046716	United Kingdom
SRR6046717	United Kingdom
SRR6046718	United Kingdom
SRR6046720	United Kingdom
SRR6046728	United Kingdom
SRR6046729	United Kingdom
SRR6046730	United Kingdom
SRR6046733	United Kingdom
SRR6046735	United Kingdom

SRR6046736	United Kingdom
SRR6046737	United Kingdom
SRR6046739	United Kingdom
SRR6046743	United Kingdom
SRR6046746	United Kingdom
SRR6046750	NA
SRR6046752	NA
SRR6046753	NA
SRR6046756	NA
SRR6046761	NA
SRR6046762	NA
SRR6046763	NA
SRR6046764	NA
SRR6046766	NA
SRR6046767	NA
SRR6046774	NA
SRR6046775	NA
SRR6046778	NA
SRR6046779	NA
SRR6046781	NA
SRR6046782	NA
SRR6046783	NA
SRR6046785	NA
SRR6046786	NA
SRR6046790	NA
SRR6046791	NA
SRR6046792	NA
SRR6046796	NA
SRR6046799	NA
SRR6046802	NA
SRR6046803	NA
SRR6046807	NA
SRR6046812	NA
SRR6046815	NA
SRR6046818	NA
SRR6046819	NA
SRR6046824	NA
SRR6046825	NA
SRR6046827	NA
SRR6046828	NA
SRR6046834	NA
SRR6046837	NA
SRR6046838	NA
SRR6046839	NA
SRR6046841	NA
SRR6046842	NA
SRR6046845	NA
SRR6046846	NA
SRR6046848	NA
SRR6046850	NA

SRR6046851	NA
SRR6046852	NA
SRR6046855	NA
SRR6046860	NA
SRR6046861	NA
SRR6046863	NA
SRR6046864	NA
SRR6046865	NA
SRR6046868	NA
SRR6046869	NA
SRR6046870	NA
SRR6046872	NA
SRR6046873	NA
SRR6046874	NA
SRR6046878	NA
SRR6046886	NA
SRR6046888	NA
SRR6046891	NA
SRR6046894	NA
SRR6046895	NA
SRR6046897	NA
SRR6046898	NA
SRR6046900	NA
SRR6046901	NA
SRR6046902	NA
SRR6046905	NA
SRR6046907	NA
SRR6046919	NA
SRR6061380	NA
SRR6061381	NA
SRR6061382	NA
SRR6061383	NA
SRR6061384	NA
SRR6061385	NA
SRR6061386	NA
SRR6061387	NA
SRR6061388	Canada
SRR6061389	Canada
SRR6061390	Canada
SRR6061391	Canada
SRR6061392	Canada
SRR6061393	Canada
SRR6061394	Canada
SRR6061395	Canada
SRR6061396	Canada
SRR6061397	Canada
SRR6061398	Canada
SRR6061399	Canada
SRR6061400	Canada
SRR6061401	Canada

SRR6061402	Canada
SRR6061403	Canada
SRR6061404	Canada
SRR6061405	Canada
SRR6061406	Canada
SRR6061407	Canada
SRR6061408	Canada
SRR6061409	Canada
SRR6061410	Canada
SRR6061411	Canada
SRR6061412	Canada
SRR6061413	Canada
SRR6061414	Canada
SRR6061415	Canada
SRR6061416	Canada
SRR6061417	Canada
SRR6061418	Canada
SRR6061419	Canada
SRR6061420	Canada
SRR6061421	Canada
SRR6061422	Canada
SRR6061423	Canada
SRR6061424	Canada
SRR6061425	Canada
SRR6061426	Canada
SRR6061427	Canada
SRR6061428	Canada
SRR6061429	Canada
SRR6063471	Canada
SRR6063472	Canada
SRR6063473	Canada
SRR6063474	Canada
SRR6063475	Canada
SRR6063476	Canada
SRR6063477	Canada
SRR6063478	Canada
SRR6063479	Canada
SRR6063480	Canada
SRR6063481	Canada
SRR6063482	Canada
SRR6063483	Canada
SRR6063484	Canada
SRR6063485	Canada
SRR6063486	Canada
SRR6063487	Canada
SRR6063488	Canada
SRR6063489	Canada
SRR6063490	Canada
SRR6063491	Canada
SRR6063492	Canada

SRR6063493	Canada
SRR6063494	Canada
SRR6063495	Canada
SRR6063496	Canada
SRR6063497	Canada
SRR6063498	Canada
SRR6063499	Canada
SRR6063500	Canada
SRR6063501	Canada
SRR6063502	Canada
SRR6063503	Canada
SRR6063504	Canada
SRR6063505	Canada
SRR6063506	Canada
SRR6063507	Canada
SRR6063508	Canada
SRR6063509	Canada
SRR6063510	Canada
SRR6063511	Canada
SRR6063512	Canada
SRR6063513	Canada
SRR6063514	Canada
SRR6063515	Canada
SRR6063516	Canada
SRR6063517	Canada
SRR6063518	Canada
SRR6063519	Canada
SRR6063520	Canada
SRR6064817	Canada
SRR6064818	Canada
SRR6064819	Canada
SRR6064820	Canada
SRR6064821	Canada
SRR6064822	Canada
SRR6064823	Canada
SRR6064824	Canada
SRR6064825	Canada
SRR6064826	Canada
SRR6064827	Canada
SRR6064828	Canada
SRR6064829	Canada
SRR6064830	Canada
SRR6064831	Canada
SRR6064832	Canada
SRR6064833	Canada
SRR6064834	Canada
SRR6064835	Canada
SRR6064836	Canada
SRR6064837	Canada
SRR6064838	Canada

SRR6064839	Canada
SRR6064840	Canada
SRR6064841	Canada
SRR6064842	Canada
SRR6064843	Canada
SRR6064844	Canada
SRR6064845	Canada
SRR6064846	Canada
SRR6064847	Canada
SRR6064848	Canada
SRR6064849	Canada
SRR6064850	Canada
SRR6064851	Canada
SRR6064852	Canada
SRR6064853	Canada
SRR6064854	Canada
SRR6064855	Canada
SRR6064856	Canada
SRR6064857	Canada
SRR6064858	Canada
SRR6064859	Canada
SRR6064860	Canada
SRR6064861	Canada
SRR6064862	Canada
SRR6064863	Canada
SRR6064864	Canada
SRR6064865	Canada
SRR6064866	Canada
SRR6067237	Canada
SRR6067238	Canada
SRR6067239	Canada
SRR6067240	Canada
SRR6067241	Canada
SRR6067242	Canada
SRR6067243	Canada
SRR6067244	Canada
SRR6067245	Canada
SRR6067246	Canada
SRR6067247	Canada
SRR6067248	Canada
SRR6067249	Canada
SRR6067250	Canada
SRR6067251	Canada
SRR6067252	Canada
SRR6067253	Canada
SRR6067254	Canada
SRR6067255	Canada
SRR6067256	Canada
SRR6067257	Canada
SRR6067258	Canada

SRR6067259	Canada
SRR6067260	Canada
SRR6067261	Canada
SRR6067262	Canada
SRR6067263	Canada
SRR6067264	Canada
SRR6067265	Canada
SRR6067266	Canada
SRR6067267	Canada
SRR6067268	Canada
SRR6067269	Canada
SRR6067270	Canada
SRR6067271	Canada
SRR6067272	Canada
SRR6067273	Canada
SRR6067274	Canada
SRR6067275	Canada
SRR6067276	Canada
SRR6067277	Canada
SRR6067278	Canada
SRR6067279	Canada
SRR6067280	Canada
SRR6067281	Canada
SRR6067282	Canada
SRR6067283	Canada
SRR6067284	Canada
SRR6067285	Canada
SRR6067286	Canada
SRR6074042	Canada
SRR6074043	Canada
SRR6074044	Canada
SRR6074045	Canada
SRR6074046	Canada
SRR6074047	Canada
SRR6074048	Canada
SRR6074049	Canada
SRR6074050	Canada
SRR6074051	Canada
SRR6074052	Canada
SRR6074053	Canada
SRR6074054	Canada
SRR6074055	Canada
SRR6074056	Canada
SRR6074057	Canada
SRR6074058	Canada
SRR6074059	Canada
SRR6074060	Canada
SRR6074061	Canada
SRR6074062	Canada
SRR6074063	Canada

SRR6074064	Canada
SRR6074065	Canada
SRR6074066	Canada
SRR6074067	Canada
SRR6074068	Canada
SRR6074069	Canada
SRR6074070	Canada
SRR6074071	Canada
SRR6074072	Canada
SRR6074073	Canada
SRR6074074	Canada
SRR6079914	Australia
SRR6079915	Australia
SRR6079916	Australia
SRR6079917	Australia
SRR6079918	Australia
SRR6079919	Australia
SRR6079920	Australia
SRR6079921	Australia
SRR6079922	Australia
SRR6079923	Australia
SRR6079924	Australia
SRR6079925	Australia
SRR6079926	Australia
SRR6079927	Australia
SRR6079928	Australia
SRR6079929	Australia
SRR6079930	Australia
SRR6079931	Australia
SRR6079932	Australia
SRR6079933	Australia
SRR6079934	Australia
SRR6079935	Australia
SRR6079936	Australia
SRR6079937	Australia
SRR6079938	Australia
SRR6079939	Australia
SRR6079940	Australia
SRR6079941	Australia
SRR6079942	Australia
SRR6079943	Australia
SRR6079944	Australia
SRR6079945	Australia
SRR6079946	Australia
SRR6079947	Australia
SRR6079948	Australia
SRR6079949	Australia
SRR6079950	Australia
SRR6079951	Australia
SRR6079952	Australia

SRR6079953	Australia
SRR6079954	Australia
SRR6079955	Australia
SRR6079956	Australia
SRR6079958	Australia
SRR6079959	Australia
SRR6079960	Australia
SRR6079961	Australia
SRR6079962	Australia
SRR6079963	Australia
SRR6079964	Australia
SRR6079965	Australia
SRR6079966	Australia
SRR6079967	Australia
SRR6079968	Australia
SRR6079969	Australia
SRR6079970	Australia
SRR6079971	Australia
SRR6079972	Australia
SRR6079973	Australia
SRR6079974	Australia
SRR6079975	Australia
SRR610904	NA
SRR610905	NA
SRR611020	NA
SRR611415	NA
SRR611416	NA
SRR611417	NA
SRR611418	NA
SRR611419	NA
SRR611420	NA
SRR611421	NA
SRR611423	NA
SRR611425	NA
SRR611426	NA
SRR6117320	Australia
SRR6117321	Australia
SRR6117322	Australia
SRR6117323	Australia
SRR6117324	Australia
SRR6117325	Australia
SRR6117326	Australia
SRR6117327	Australia
SRR6117328	Australia
SRR6117329	Australia
SRR6117330	Australia
SRR6117331	Australia
SRR6117332	Australia
SRR6117333	Australia
SRR6117334	Australia

SRR6117335	Australia
SRR6117336	Australia
SRR6117337	Australia
SRR6117338	Australia
SRR6117339	Australia
SRR6117340	Australia
SRR6117341	Australia
SRR6117342	Australia
SRR6117343	Australia
SRR6117344	Australia
SRR6117345	Australia
SRR6117346	Australia
SRR6117347	Australia
SRR6117348	Australia
SRR6117349	Australia
SRR6117350	Australia
SRR6152639	Canada
SRR6152640	Canada
SRR6152641	Canada
SRR6152642	Canada
SRR6152643	Canada
SRR6152644	Canada
SRR6152645	Canada
SRR6152646	Canada
SRR6152647	Canada
SRR6152648	Canada
SRR6152649	Canada
SRR6152650	Canada
SRR6152651	Canada
SRR6152652	Canada
SRR6152653	Canada
SRR6152654	Canada
SRR6152655	Canada
SRR6152656	Canada
SRR6152657	Canada
SRR6152658	Canada
SRR6152659	Canada
SRR6152660	Canada
SRR6152661	Canada
SRR6152662	Canada
SRR6152663	Canada
SRR6152664	Canada
SRR6152665	Canada
SRR6152666	Canada
SRR6152667	Canada
SRR6152668	Canada
SRR6152669	Canada
SRR6152670	Canada
SRR6152671	Canada
SRR6152672	Canada

SRR6152673	Canada
SRR6152674	Canada
SRR6152675	Canada
SRR6152676	Canada
SRR6152677	Canada
SRR6152678	Canada
SRR6152679	Canada
SRR6152680	Canada
SRR6152681	Canada
SRR6152682	Canada
SRR6152683	Canada
SRR6152684	Canada
SRR6152685	Canada
SRR6152686	Canada
SRR6152687	Canada
SRR6152688	Canada
SRR6152689	Canada
SRR6152690	Canada
SRR6152691	Canada
SRR6152692	Canada
SRR6152693	Canada
SRR6152694	Canada
SRR6152695	Canada
SRR6152696	Canada
SRR6152697	Canada
SRR6152698	Canada
SRR6152699	Canada
SRR6152700	Canada
SRR6152701	Canada
SRR6152702	Canada
SRR6152703	Canada
SRR6152704	Canada
SRR6152705	Canada
SRR6152706	Canada
SRR6152707	Canada
SRR6152708	Canada
SRR6152709	Canada
SRR6152710	Canada
SRR6152711	Canada
SRR6152712	Canada
SRR6152713	Canada
SRR6152714	Canada
SRR6152715	Canada
SRR6152716	Canada
SRR6152717	Canada
SRR6152718	Canada
SRR6152719	Canada
SRR6152720	Canada
SRR6152721	Canada
SRR6152722	Canada

SRR6152723	Canada
SRR6152724	Canada
SRR6152725	Canada
SRR6152726	Canada
SRR6152727	Canada
SRR6152728	Canada
SRR6152729	Canada
SRR6152730	Canada
SRR6152731	Canada
SRR6152732	Canada
SRR6152733	Canada
SRR6152734	Canada
SRR6152735	Canada
SRR6152736	Canada
SRR6152737	Canada
SRR6152738	Canada
SRR6152739	Canada
SRR6152740	Canada
SRR6152741	Canada
SRR6152742	Canada
SRR6152743	Canada
SRR6152744	Canada
SRR6152745	Canada
SRR6152746	Canada
SRR6152747	Canada
SRR6152748	Canada
SRR6152749	Canada
SRR6152750	Canada
SRR6152751	Canada
SRR6152752	Canada
SRR6152753	Canada
SRR6152754	Canada
SRR6152755	Canada
SRR6152756	Canada
SRR6152757	Canada
SRR6152758	Canada
SRR6152759	Canada
SRR6152760	Canada
SRR6152761	Canada
SRR6152762	Canada
SRR6152763	Canada
SRR6152764	Canada
SRR6152765	Canada
SRR6152766	Canada
SRR6152767	Canada
SRR6152768	Canada
SRR6152769	Canada
SRR6152770	Canada
SRR6152771	Canada
SRR6152772	Canada

SRR6152773	Canada
SRR6152774	Canada
SRR6152775	Canada
SRR6152776	Canada
SRR6152777	Canada
SRR6152778	Canada
SRR6152779	Canada
SRR6152780	Canada
SRR6152781	Canada
SRR6152782	Canada
SRR6152783	Canada
SRR6152784	Canada
SRR6152785	Canada
SRR6152786	Canada
SRR6152787	Canada
SRR6152788	Canada
SRR6152789	Canada
SRR6152790	Canada
SRR6152791	Canada
SRR6152792	Canada
SRR6152793	Canada
SRR6152794	Canada
SRR6152795	Canada
SRR6152796	Canada
SRR6152797	Canada
SRR6152798	Canada
SRR6152799	Canada
SRR6152800	Canada
SRR6152801	Canada
SRR6152802	Canada
SRR6152803	Canada
SRR6152804	Canada
SRR6152805	Canada
SRR6152806	Canada
SRR6152807	Canada
SRR6152808	Canada
SRR6152809	Canada
SRR6152810	Canada
SRR6152811	Canada
SRR6152812	Canada
SRR6152813	Canada
SRR6152814	Canada
SRR6152815	Canada
SRR6152816	Canada
SRR6152817	Canada
SRR6152818	Canada
SRR6152819	Canada
SRR6152820	Canada
SRR6152821	Canada
SRR6152822	Canada

SRR6152823	Canada
SRR6152824	Canada
SRR6152825	Canada
SRR6152826	Canada
SRR6152827	Canada
SRR6152828	Canada
SRR6152829	Canada
SRR6152830	Canada
SRR6152831	Canada
SRR6152832	Canada
SRR6152833	Canada
SRR6152834	Canada
SRR6152835	Canada
SRR6152836	Canada
SRR6152837	Canada
SRR6152838	Canada
SRR6152839	Canada
SRR6152840	Canada
SRR6152841	Canada
SRR6152842	Canada
SRR6152843	Canada
SRR6152844	Canada
SRR6152845	Canada
SRR6152846	Canada
SRR6152847	Canada
SRR6152848	Canada
SRR6152849	Canada
SRR6152850	Canada
SRR6152851	Canada
SRR6152852	Canada
SRR6152853	Canada
SRR6152854	Canada
SRR6152855	Canada
SRR6152856	Canada
SRR6152857	Canada
SRR6152858	Canada
SRR6152859	Canada
SRR6152860	Canada
SRR6152861	Canada
SRR6152862	Canada
SRR6152863	Canada
SRR6152864	Canada
SRR6152865	Canada
SRR6152866	Canada
SRR6152867	Canada
SRR6152868	Canada
SRR6152869	Canada
SRR6152870	Canada
SRR6152871	Canada
SRR6152872	Canada

SRR6152873	Canada
SRR6152874	Canada
SRR6152875	Canada
SRR6152876	Canada
SRR6152877	Canada
SRR6152878	Canada
SRR6152879	Canada
SRR6152880	Canada
SRR6152881	Canada
SRR6152882	Canada
SRR6152883	Canada
SRR6152884	Canada
SRR6152885	Canada
SRR6152886	Canada
SRR6152887	Canada
SRR6152888	Canada
SRR6152889	Canada
SRR6152890	Canada
SRR6152891	Canada
SRR6152892	Canada
SRR6152893	Canada
SRR6152894	Canada
SRR6152895	Canada
SRR6152896	Canada
SRR6152897	Canada
SRR6152898	Canada
SRR6152899	Canada
SRR6152900	Canada
SRR6152901	Canada
SRR6152902	Canada
SRR6152903	Canada
SRR6152904	Canada
SRR6152905	Canada
SRR6152906	Canada
SRR6152907	Canada
SRR6152908	Canada
SRR6152909	Canada
SRR6152910	Canada
SRR6152911	Canada
SRR6152912	Canada
SRR6152913	Canada
SRR6152914	Canada
SRR6152915	Canada
SRR6152916	Canada
SRR6152917	Canada
SRR6152918	Canada
SRR6152919	Canada
SRR6152920	Canada
SRR6152921	Canada
SRR6152922	Canada

SRR6152923	Canada
SRR6152924	Canada
SRR6152925	Canada
SRR6152926	Canada
SRR6152927	Canada
SRR6152928	Canada
SRR6152929	Canada
SRR6152930	Canada
SRR6152931	Canada
SRR6152932	Canada
SRR6152933	Canada
SRR6152934	Canada
SRR6152935	Canada
SRR6152936	Canada
SRR6152937	Canada
SRR6152938	Canada
SRR6152939	Canada
SRR6152940	Canada
SRR6152941	Canada
SRR6152942	Canada
SRR6152943	Canada
SRR6152944	Canada
SRR6152945	Canada
SRR6152946	Canada
SRR6152947	Canada
SRR6152948	Canada
SRR6152949	Canada
SRR6152950	Canada
SRR6152951	Canada
SRR6152952	Canada
SRR6152953	Canada
SRR6152954	Canada
SRR6152955	Canada
SRR6152956	Canada
SRR6152957	Canada
SRR6152958	Canada
SRR6152959	Canada
SRR6152960	Canada
SRR6152961	Canada
SRR6152962	Canada
SRR6152963	Canada
SRR6152964	Canada
SRR6152965	Canada
SRR6152966	Canada
SRR6152967	Canada
SRR6152968	Canada
SRR6152969	Canada
SRR6152970	Canada
SRR6152971	Canada
SRR6152972	Canada

SRR6152973	Canada
SRR6152974	Canada
SRR6152975	Canada
SRR6152976	Canada
SRR6152977	Canada
SRR6152978	Canada
SRR6152979	Canada
SRR6152980	Canada
SRR6152981	Canada
SRR6152982	Canada
SRR6152983	Canada
SRR6152984	Canada
SRR6152985	Canada
SRR6152986	Canada
SRR6152987	Canada
SRR6152988	Canada
SRR6152989	Canada
SRR6152990	Canada
SRR6152991	Canada
SRR6152992	Canada
SRR6152993	Canada
SRR6152994	Canada
SRR6152995	Canada
SRR6152996	Canada
SRR6152997	Canada
SRR6152998	Canada
SRR6152999	Canada
SRR6153000	Canada
SRR6153001	Canada
SRR6153002	Canada
SRR6153003	Canada
SRR6153004	Canada
SRR6153005	Canada
SRR6153006	Canada
SRR6153007	Canada
SRR6153008	Canada
SRR6153009	Canada
SRR6153010	Canada
SRR6153011	Canada
SRR6153012	Canada
SRR6153013	Canada
SRR6153014	Canada
SRR6153015	Canada
SRR6153016	Canada
SRR6153017	Canada
SRR6153018	Canada
SRR6153019	Canada
SRR6153020	Canada
SRR6153021	Canada
SRR6153022	Canada

SRR6153023	Canada
SRR6153024	Canada
SRR6153025	Canada
SRR6153026	Canada
SRR6153027	Canada
SRR6153028	Canada
SRR6153029	Canada
SRR6153030	Canada
SRR6153031	Canada
SRR6153032	Canada
SRR6153033	Canada
SRR6153034	Canada
SRR6153035	Canada
SRR6153036	Canada
SRR6153037	Canada
SRR6153038	Canada
SRR6153039	Canada
SRR6153040	Canada
SRR6153041	Canada
SRR6153042	Canada
SRR6153043	Canada
SRR6153044	Canada
SRR6153045	Canada
SRR6153046	Canada
SRR6153047	Canada
SRR6153048	Canada
SRR6153049	Canada
SRR6153050	Canada
SRR6153051	Canada
SRR6153052	Canada
SRR6153053	Canada
SRR6153054	Canada
SRR6153055	Canada
SRR6153056	Canada
SRR6153057	Canada
SRR6153058	Canada
SRR6153059	Canada
SRR6153060	Canada
SRR6153061	Canada
SRR6153062	Canada
SRR6153063	Canada
SRR6153064	Canada
SRR6153065	Canada
SRR6153066	Canada
SRR6153067	Canada
SRR6153068	Canada
SRR6153069	Canada
SRR6153070	Canada
SRR6153071	Canada
SRR6153072	Canada

SRR6153073	Canada
SRR6153074	Canada
SRR6153075	Canada
SRR6153076	Canada
SRR6153077	Canada
SRR6153078	Canada
SRR6153079	Canada
SRR6153080	Canada
SRR6153081	Canada
SRR6153082	Canada
SRR6153083	Canada
SRR6153084	Canada
SRR6153085	Canada
SRR6153086	Canada
SRR6153087	Canada
SRR6153088	Canada
SRR6153089	Canada
SRR6153090	Canada
SRR6153091	Canada
SRR6153092	Canada
SRR6153093	Canada
SRR6153094	Canada
SRR6153095	Canada
SRR6153096	Canada
SRR6153097	Canada
SRR6153098	Canada
SRR6153099	Canada
SRR6153100	Canada
SRR6153101	Canada
SRR6153102	Canada
SRR6153103	Canada
SRR6153104	Canada
SRR6153105	Canada
SRR6153106	Canada
SRR6153107	Canada
SRR6153108	Canada
SRR6153109	Canada
SRR6153110	Canada
SRR6153111	Canada
SRR6153112	Canada
SRR6153113	Canada
SRR6153114	Canada
SRR6153115	Canada
SRR6153116	Canada
SRR6153117	Canada
SRR6153118	Canada
SRR6153119	Canada
SRR6153120	Canada
SRR6153121	Canada
SRR6153122	Canada

SRR6153123	Canada
SRR6153124	Canada
SRR6153125	Canada
SRR6153126	Canada
SRR6153127	Canada
SRR6153128	Canada
SRR6153129	Canada
SRR6153130	Canada
SRR6153131	Canada
SRR6153132	Canada
SRR6153133	Canada
SRR6153134	Canada
SRR6153135	Canada
SRR6153136	Canada
SRR6153137	Canada
SRR6153138	Canada
SRR6153139	Canada
SRR6153140	Canada
SRR6153141	Canada
SRR6153142	Canada
SRR6153143	Canada
SRR6153144	Canada
SRR6153145	Canada
SRR6153146	Canada
SRR6153147	Canada
SRR6153148	Canada
SRR6153149	Canada
SRR6153150	Canada
SRR6153151	Canada
SRR6153152	Canada
SRR6153153	Canada
SRR6153154	Canada
SRR6153155	Canada
SRR6153156	Canada
SRR6153157	Canada
SRR6153158	Canada
SRR6153159	Canada
SRR6153160	Canada
SRR6153161	Canada
SRR6153162	Canada
SRR6153163	Canada
SRR6153164	Canada
SRR6153165	Canada
SRR6153166	Canada
SRR6153167	Canada
SRR6153168	Canada
SRR6153169	Canada
SRR6153170	Canada
SRR6153171	Canada
SRR6153172	Canada

SRR6153173	Canada
SRR6153174	Canada
SRR6153175	Canada
SRR6153176	Canada
SRR6153177	Canada
SRR6153178	Canada
SRR6153179	Canada
SRR6153180	Canada
SRR6153181	Canada
SRR6153182	Canada
SRR6153183	Canada
SRR6153184	Canada
SRR6153185	Canada
SRR6153186	Canada
SRR6153187	Canada
SRR6153188	Canada
SRR6153189	Canada
SRR6153190	Canada
SRR6153191	Canada
SRR6153192	Canada
SRR6153193	Canada
SRR6153194	Canada
SRR6153195	Canada
SRR6153196	Canada
SRR6153197	Canada
SRR6153198	Canada
SRR6153199	Canada
SRR6153200	Canada
SRR6153201	Canada
SRR6153202	Canada
SRR6153203	Canada
SRR6153204	Canada
SRR6153205	Canada
SRR6153206	Canada
SRR6153207	Canada
SRR6153208	Canada
SRR6153209	Canada
SRR6153210	Canada
SRR6153211	Canada
SRR6153212	Canada
SRR6153213	Canada
SRR6153214	Canada
SRR6153215	Canada
SRR6153216	Canada
SRR6153217	Canada
SRR6153218	Canada
SRR6153219	Canada
SRR6153220	Canada
SRR6153221	Canada
SRR6153222	Canada

SRR6153223	Canada
SRR6153224	Canada
SRR6153225	Canada
SRR6153226	Canada
SRR6153227	Canada
SRR6153228	Canada
SRR6153229	Canada
SRR6153230	Canada
SRR6153231	Canada
SRR6153232	Canada
SRR6153233	Canada
SRR6153234	Canada
SRR6153235	Canada
SRR6153236	Canada
SRR6153237	Canada
SRR6153238	Canada
SRR6153239	Canada
SRR6153240	Canada
SRR6153241	Canada
SRR6153242	Canada
SRR6153243	Canada
SRR6153244	Canada
SRR6153245	Canada
SRR6153246	Canada
SRR6153247	Canada
SRR6153248	Canada
SRR6153249	Canada
SRR6153250	Canada
SRR6153251	Canada
SRR6153252	Canada
SRR6153253	Canada
SRR6153254	Canada
SRR6153255	Canada
SRR6153256	Canada
SRR6153257	Canada
SRR6153258	Canada
SRR6153259	Canada
SRR6153260	Canada
SRR6153261	Canada
SRR6153262	Canada
SRR6153263	Canada
SRR6153264	Canada
SRR6153265	Canada
SRR6153266	Canada
SRR6153267	Canada
SRR6153268	Canada
SRR6153269	Canada
SRR6153270	Canada
SRR6153271	Canada
SRR6153272	Canada

SRR6153273	Canada
SRR6153274	Canada
SRR6153275	Canada
SRR6153276	Canada
SRR6187068	Italy
SRR6187069	Italy
SRR6187070	Italy
SRR6187071	Italy
SRR6256975	Russia
SRR6256976	Russia
SRR6256977	Russia
SRR6256978	Russia
SRR6256979	Russia
SRR6256980	Russia
SRR6256981	Russia
SRR6256982	Russia
SRR6256983	Russia
SRR6256984	Russia
SRR6257035	Russia
SRR6257036	Russia
SRR6257037	Russia
SRR6257038	Russia
SRR6257039	Russia
SRR6257040	Russia
SRR6257041	Russia
SRR6257042	Russia
SRR6257043	Russia
SRR6257044	Russia
SRR6257061	Russia
SRR6257062	Russia
SRR6257063	Russia
SRR6257064	Russia
SRR6257065	Russia
SRR6257066	Russia
SRR6257067	Russia
SRR6257068	Russia
SRR6257069	Russia
SRR6325442	United States of America
SRR6325443	United States of America
SRR6325444	United States of America
SRR6325445	United States of America
SRR6325446	United States of America
SRR6325447	United States of America
SRR6325448	United States of America
SRR6325449	United States of America
SRR6325450	United States of America
SRR6325451	United States of America
SRR6325452	United States of America
SRR6325453	United States of America
SRR6325454	United States of America

SRR6325455	United States of America
SRR6325456	United States of America
SRR6339636	Australia
SRR6339637	Australia
SRR6339638	Australia
SRR6339639	Australia
SRR6339640	Australia
SRR6339641	Australia
SRR6339642	Australia
SRR6339643	Australia
SRR6339644	Australia
SRR6339645	Australia
SRR6339646	Australia
SRR6339647	Australia
SRR6339648	Australia
SRR6339649	Australia
SRR6339650	Australia
SRR6339651	Australia
SRR6339652	Australia
SRR6339653	Australia
SRR6339654	Australia
SRR6339655	Australia
SRR6339656	Australia
SRR6339657	Australia
SRR6339658	Australia
SRR6339659	Australia
SRR6339660	Australia
SRR6339661	Australia
SRR6339662	Australia
SRR6339663	Australia
SRR6339664	Australia
SRR6339665	Australia
SRR6339666	Australia
SRR6339667	Australia
SRR6353870	Russia
SRR6353871	Russia
SRR6353872	Russia
SRR6353873	Russia
SRR6353874	Russia
SRR6353875	Russia
SRR6353876	Russia
SRR6353877	Russia
SRR6353878	Russia
SRR6353879	Russia
SRR6353880	Russia
SRR6353881	Russia
SRR6353882	Russia
SRR6353883	Russia
SRR6353884	Russia
SRR6353885	Russia

SRR6353886	Russia
SRR6353887	Russia
SRR6353888	Russia
SRR6353889	Russia
SRR6356923	Azerbaijan
SRR6356924	Azerbaijan
SRR6356925	Azerbaijan
SRR6356926	Azerbaijan
SRR6356927	Azerbaijan
SRR6356928	Azerbaijan
SRR6356929	Azerbaijan
SRR6356930	Azerbaijan
SRR6356931	Azerbaijan
SRR6356932	Azerbaijan
SRR6356933	Azerbaijan
SRR6356934	Azerbaijan
SRR6356935	Azerbaijan
SRR6356936	Azerbaijan
SRR6356937	Azerbaijan
SRR6356938	Azerbaijan
SRR6356939	Azerbaijan
SRR6356940	Azerbaijan
SRR6356941	Azerbaijan
SRR6356942	Azerbaijan
SRR6356943	Azerbaijan
SRR6356944	Azerbaijan
SRR6356945	Azerbaijan
SRR6356946	Azerbaijan
SRR6356947	Azerbaijan
SRR6356948	Azerbaijan
SRR6356949	Azerbaijan
SRR6356950	Azerbaijan
SRR6356951	Azerbaijan
SRR6356952	Azerbaijan
SRR6356953	Azerbaijan
SRR6356954	Azerbaijan
SRR6356955	Azerbaijan
SRR6356956	Azerbaijan
SRR6356957	Azerbaijan
SRR6356958	Azerbaijan
SRR6356959	Azerbaijan
SRR6356960	Azerbaijan
SRR6356961	Azerbaijan
SRR6356962	Azerbaijan
SRR6356963	Azerbaijan
SRR6356964	Azerbaijan
SRR6356965	Azerbaijan
SRR6356966	Azerbaijan
SRR6356967	Azerbaijan
SRR6356968	Azerbaijan

SRR6356969	Azerbaijan
SRR6356970	Azerbaijan
SRR6356971	Azerbaijan
SRR6356972	Azerbaijan
SRR6356973	Azerbaijan
SRR6356974	Azerbaijan
SRR6356975	Azerbaijan
SRR6356976	Azerbaijan
SRR6356977	Azerbaijan
SRR6356978	Azerbaijan
SRR6356979	Azerbaijan
SRR6356980	Azerbaijan
SRR6356981	Azerbaijan
SRR6356982	Azerbaijan
SRR6356983	Azerbaijan
SRR6356984	Azerbaijan
SRR6356985	Azerbaijan
SRR6356986	Azerbaijan
SRR6356987	Azerbaijan
SRR6356988	Azerbaijan
SRR6356989	Azerbaijan
SRR6356990	Azerbaijan
SRR6356991	Azerbaijan
SRR6356992	Azerbaijan
SRR6356993	Azerbaijan
SRR6356994	Azerbaijan
SRR6356995	Azerbaijan
SRR6356996	Azerbaijan
SRR6356997	Azerbaijan
SRR6356998	Azerbaijan
SRR6356999	Azerbaijan
SRR6357000	Azerbaijan
SRR6357001	Azerbaijan
SRR6357002	Azerbaijan
SRR6357003	Azerbaijan
SRR6357004	Azerbaijan
SRR6357005	Azerbaijan
SRR6357006	Azerbaijan
SRR6357007	Azerbaijan
SRR6357008	Azerbaijan
SRR6384962	Azerbaijan
SRR6384963	Azerbaijan
SRR6384964	Azerbaijan
SRR6384965	Azerbaijan
SRR6384966	Azerbaijan
SRR6384967	Azerbaijan
SRR6384968	Azerbaijan
SRR6384969	Azerbaijan
SRR6384970	Azerbaijan
SRR6389897	Guatemala

SRR6389898	Guatemala
SRR6389899	Guatemala
SRR6389900	Guatemala
SRR6389901	Guatemala
SRR6389902	Guatemala
SRR6389903	Guatemala
SRR6389904	Guatemala
SRR6389905	Guatemala
SRR6389906	Guatemala
SRR6389907	Guatemala
SRR6389908	Guatemala
SRR6389909	Guatemala
SRR6389910	Guatemala
SRR6389911	Guatemala
SRR6397324	Canada
SRR6397325	Canada
SRR6397326	Canada
SRR6397327	Canada
SRR6397328	Canada
SRR6397329	Canada
SRR6397330	Canada
SRR6397331	Canada
SRR6397332	Canada
SRR6397333	Canada
SRR6397334	Canada
SRR6397335	Canada
SRR6397336	Canada
SRR6397337	Canada
SRR6397338	Canada
SRR6397339	Canada
SRR6397340	Canada
SRR6397341	Canada
SRR6397342	Canada
SRR6397343	Canada
SRR6397344	Canada
SRR6397345	Canada
SRR6397346	Canada
SRR6397347	Canada
SRR6397348	Canada
SRR6397349	Canada
SRR6397350	Canada
SRR6397351	Canada
SRR6397352	Canada
SRR6397353	Canada
SRR6397354	Canada
SRR6397355	Canada
SRR6397356	Canada
SRR6397357	Canada
SRR6397358	Canada
SRR6397359	Canada

SRR6397360	Canada
SRR6397361	Canada
SRR6397362	Canada
SRR6397363	Canada
SRR6397364	Canada
SRR6397365	Canada
SRR6397366	Canada
SRR6397367	Canada
SRR6397368	Canada
SRR6397369	Canada
SRR6397370	Canada
SRR6397371	Canada
SRR6397372	Canada
SRR6397373	Canada
SRR6397374	Canada
SRR6397375	Canada
SRR6397376	Canada
SRR6397377	Canada
SRR6397378	Canada
SRR6397379	Canada
SRR6397380	Canada
SRR6397381	Canada
SRR6397382	Canada
SRR6397383	Canada
SRR6397384	Canada
SRR6397385	Canada
SRR6397386	Canada
SRR6397387	Canada
SRR6397388	Canada
SRR6397389	Canada
SRR6397390	Canada
SRR6397391	Canada
SRR6397392	Canada
SRR6397393	Canada
SRR6397394	Canada
SRR6397395	Canada
SRR6397396	Canada
SRR6397397	Canada
SRR6397398	Canada
SRR6397399	Canada
SRR6397400	Canada
SRR6397401	Canada
SRR6397402	Canada
SRR6397403	Canada
SRR6397404	Canada
SRR6397405	Canada
SRR6397406	Canada
SRR6397407	Canada
SRR6397408	Canada
SRR6397409	Canada

SRR6397410	Canada
SRR6397411	Canada
SRR6397412	Canada
SRR6397413	Canada
SRR6397414	Canada
SRR6397415	Canada
SRR6397416	Canada
SRR6397417	Canada
SRR6397418	Canada
SRR6397419	Canada
SRR6397420	Canada
SRR6397421	Canada
SRR6397422	Canada
SRR6397423	Canada
SRR6397424	Canada
SRR6397425	Canada
SRR6397426	Canada
SRR6397427	Canada
SRR6397428	Canada
SRR6397429	Canada
SRR6397430	Canada
SRR6397431	Canada
SRR6397432	Canada
SRR6397433	Canada
SRR6397434	Canada
SRR6397435	Canada
SRR6397436	Canada
SRR6397437	Canada
SRR6397438	Canada
SRR6397439	Canada
SRR6397440	Canada
SRR6397441	Canada
SRR6397442	Canada
SRR6397443	Canada
SRR6397444	Canada
SRR6397445	Canada
SRR6397446	Canada
SRR6397447	Canada
SRR6397448	Canada
SRR6397449	Canada
SRR6397450	Canada
SRR6397451	Canada
SRR6397452	Canada
SRR6397453	Canada
SRR6397454	Canada
SRR6397455	Canada
SRR6397456	Canada
SRR6397457	Canada
SRR6397458	Canada
SRR6397459	Canada

SRR6397460	Canada
SRR6397461	Canada
SRR6397462	Canada
SRR6397463	Canada
SRR6397464	Canada
SRR6397465	Canada
SRR6397466	Canada
SRR6397467	Canada
SRR6397468	Canada
SRR6397469	Canada
SRR6397470	Canada
SRR6397471	Canada
SRR6397472	Canada
SRR6397473	Canada
SRR6397474	Canada
SRR6397475	Canada
SRR6397476	Canada
SRR6397477	Canada
SRR6397478	Canada
SRR6397479	Canada
SRR6397480	Canada
SRR6397481	Canada
SRR6397482	Canada
SRR6397483	Canada
SRR6397484	Canada
SRR6397485	Canada
SRR6397486	Canada
SRR6397487	Canada
SRR6397488	Canada
SRR6397489	Canada
SRR6397490	Canada
SRR6397491	Canada
SRR6397492	Canada
SRR6397493	Canada
SRR6397494	Canada
SRR6397495	Canada
SRR6397496	Canada
SRR6397497	Canada
SRR6397498	Canada
SRR6397499	Canada
SRR6397500	Canada
SRR6397501	Canada
SRR6397502	Canada
SRR6397503	Canada
SRR6397504	Canada
SRR6397505	Canada
SRR6397506	Canada
SRR6397507	Canada
SRR6397508	Canada
SRR6397509	Canada

SRR6397510	Canada
SRR6397511	Canada
SRR6397512	Canada
SRR6397513	Canada
SRR6397514	Canada
SRR6397515	Canada
SRR6397516	Canada
SRR6397517	Canada
SRR6397518	Canada
SRR6397519	Canada
SRR6397520	Canada
SRR6397521	Canada
SRR6397522	Canada
SRR6397523	Canada
SRR6397524	Canada
SRR6397525	Canada
SRR6397526	Canada
SRR6397527	Canada
SRR6397528	Canada
SRR6397529	Canada
SRR6397530	Canada
SRR6397531	Canada
SRR6397532	Canada
SRR6397533	Canada
SRR6397534	Canada
SRR6397535	Canada
SRR6397536	Canada
SRR6397537	Canada
SRR6397538	Canada
SRR6397539	Canada
SRR6397540	Canada
SRR6397541	Canada
SRR6397542	Canada
SRR6397543	Canada
SRR6397544	Canada
SRR6397545	Canada
SRR6397546	Canada
SRR6397547	Canada
SRR6397548	Canada
SRR6397549	Canada
SRR6397550	Canada
SRR6397551	Canada
SRR6397552	Canada
SRR6397553	Canada
SRR6397554	Canada
SRR6397555	Canada
SRR6397556	Canada
SRR6397557	Canada
SRR6397558	Canada
SRR6397559	Canada

SRR6397560	Canada
SRR6397561	Canada
SRR6397562	Canada
SRR6397563	Canada
SRR6397564	Canada
SRR6397565	Canada
SRR6397566	Canada
SRR6397567	Canada
SRR6397568	Canada
SRR6397569	Canada
SRR6397570	Canada
SRR6397571	Canada
SRR6397572	Canada
SRR6397573	Canada
SRR6397574	Canada
SRR6397575	Canada
SRR6397576	Canada
SRR6397577	Canada
SRR6397578	Canada
SRR6397579	Canada
SRR6397580	Canada
SRR6397581	Canada
SRR6397582	Canada
SRR6397583	Canada
SRR6397584	Canada
SRR6397585	Canada
SRR6397586	Canada
SRR6397587	Canada
SRR6397588	Canada
SRR6397589	Canada
SRR6397590	Canada
SRR6397591	Canada
SRR6397592	Canada
SRR6397593	Canada
SRR6397594	Canada
SRR6397595	Canada
SRR6397596	Canada
SRR6397597	Canada
SRR6397598	Canada
SRR6397599	Canada
SRR6397600	Canada
SRR6397601	Canada
SRR6397602	Canada
SRR6397603	Canada
SRR6397604	Canada
SRR6397605	Canada
SRR6397606	Canada
SRR6397607	Canada
SRR6397608	Canada
SRR6397609	Canada

SRR6397610	Canada
SRR6397611	Canada
SRR6397612	Canada
SRR6397613	Canada
SRR6397614	Canada
SRR6397615	Canada
SRR6397616	Canada
SRR6397617	Canada
SRR6397618	Canada
SRR6397619	Canada
SRR6397620	Canada
SRR6397621	Canada
SRR6397622	Canada
SRR6397623	Canada
SRR6397624	Canada
SRR6397625	Canada
SRR6397626	Canada
SRR6397627	Canada
SRR6397628	Canada
SRR6397629	Canada
SRR6397630	Canada
SRR6397631	Canada
SRR6397632	Canada
SRR6397633	Canada
SRR6397634	Canada
SRR6397635	Canada
SRR6397636	Canada
SRR6397637	Canada
SRR6397638	Canada
SRR6397639	Canada
SRR6397640	Canada
SRR6397641	Canada
SRR6397642	Canada
SRR6397643	Canada
SRR6397644	Canada
SRR6397645	Canada
SRR6397646	Canada
SRR6397647	Canada
SRR6397648	Canada
SRR6397649	Canada
SRR6397650	Canada
SRR6397651	Canada
SRR6397652	Canada
SRR6397653	Canada
SRR6397654	Canada
SRR6397655	Canada
SRR6397656	Canada
SRR6397657	Canada
SRR6397658	Canada
SRR6397659	Canada

SRR6397660	Canada
SRR6397661	Canada
SRR6397662	Canada
SRR6397663	Canada
SRR6397664	Canada
SRR6397665	Canada
SRR6397666	Canada
SRR6397667	Canada
SRR6397668	Canada
SRR6397669	Canada
SRR6397670	Canada
SRR6397671	Canada
SRR6397672	Canada
SRR6397673	Canada
SRR6397674	Canada
SRR6397675	Canada
SRR6397676	Canada
SRR6397677	Canada
SRR6397678	Canada
SRR6397679	Canada
SRR6397680	Canada
SRR6397681	Canada
SRR6397682	Canada
SRR6397683	Canada
SRR6397684	Canada
SRR6397685	Canada
SRR6397686	Canada
SRR6397687	Canada
SRR6397688	Canada
SRR6397689	Canada
SRR6397690	Canada
SRR6397691	Canada
SRR6397692	Canada
SRR6397693	Canada
SRR6397694	Canada
SRR6397695	Canada
SRR6397696	Canada
SRR6397697	Canada
SRR6397698	Canada
SRR6397699	Canada
SRR6397700	Canada
SRR6397701	Canada
SRR6397702	Canada
SRR6397703	Canada
SRR6397704	Canada
SRR6397705	Canada
SRR6397706	Canada
SRR6397707	Canada
SRR6397708	Canada
SRR6397709	Canada

SRR6397710	Canada
SRR6397711	Canada
SRR6397712	Canada
SRR6397713	Canada
SRR6397714	Canada
SRR6397715	Canada
SRR6397716	Canada
SRR6397717	Canada
SRR6397718	Canada
SRR6397719	Canada
SRR6397720	Canada
SRR6397721	Canada
SRR6397722	Canada
SRR6397723	Canada
SRR6397724	Canada
SRR6397725	Canada
SRR6397726	Canada
SRR6397727	Canada
SRR6397728	Canada
SRR6397729	Canada
SRR6397730	Canada
SRR6397731	Canada
SRR6397732	Canada
SRR6397733	Canada
SRR6397734	Canada
SRR6397735	Canada
SRR6397736	Canada
SRR6397737	Canada
SRR6397738	Canada
SRR6397739	Canada
SRR6397740	Canada
SRR6397741	Canada
SRR6397742	Canada
SRR6397743	Canada
SRR6397744	Canada
SRR6397745	Canada
SRR6397746	Canada
SRR6397747	Canada
SRR6397748	Canada
SRR6397749	Canada
SRR6397750	Canada
SRR6397751	Canada
SRR6397752	Canada
SRR6397753	Canada
SRR6397754	Canada
SRR6397755	Canada
SRR6397756	Canada
SRR6397757	Canada
SRR6397758	Canada
SRR6397759	Canada

SRR6397760	Canada
SRR6397761	Canada
SRR6397762	Canada
SRR6397763	Canada
SRR6397764	Canada
SRR6397765	Canada
SRR6397766	Canada
SRR6397767	Canada
SRR6397768	Canada
SRR6397769	Canada
SRR6397770	Canada
SRR6397771	Canada
SRR6397772	Canada
SRR6397773	Canada
SRR6397774	Canada
SRR6397775	Canada
SRR6397776	Canada
SRR6397777	Canada
SRR6397778	Canada
SRR6397779	Canada
SRR6397780	Canada
SRR6397781	Canada
SRR6397782	Canada
SRR6397783	Canada
SRR6397784	Canada
SRR6397785	Canada
SRR6397786	Canada
SRR6397787	Canada
SRR6397788	Canada
SRR6397789	Canada
SRR6397790	Canada
SRR6397791	Canada
SRR6397792	Canada
SRR6397793	Canada
SRR6397794	Canada
SRR6397795	Canada
SRR6397796	Canada
SRR6397797	Canada
SRR6397798	Canada
SRR6397799	Canada
SRR6397800	Canada
SRR6397801	Canada
SRR6397802	Canada
SRR6397803	Canada
SRR6397804	Canada
SRR6397805	Canada
SRR6397806	Canada
SRR6397807	Canada
SRR6397808	Canada
SRR6397809	Canada

SRR6397810	Canada
SRR6397811	Canada
SRR6397812	Canada
SRR6397813	Canada
SRR6397814	Canada
SRR6397815	Canada
SRR6397816	Canada
SRR6397817	Canada
SRR6397818	Canada
SRR6397819	Canada
SRR6397820	Canada
SRR6397821	Canada
SRR6397822	Canada
SRR6397823	Canada
SRR6397915	Canada
SRR6397916	Canada
SRR6397917	Canada
SRR6397918	Canada
SRR6397919	Canada
SRR6397920	Canada
SRR6397921	Canada
SRR6397922	Canada
SRR6397923	Canada
SRR6397924	Canada
SRR6397925	Canada
SRR6397926	Canada
SRR6397927	Canada
SRR6397928	Canada
SRR6397929	Canada
SRR6397930	Canada
SRR6397931	Canada
SRR6397932	Canada
SRR6397933	Canada
SRR6397934	Canada
SRR6397935	Canada
SRR6397936	Canada
SRR6397937	Canada
SRR6397938	Canada
SRR6397939	Canada
SRR6397940	Canada
SRR6397941	Canada
SRR6397942	Canada
SRR6397943	Canada
SRR6397944	Canada
SRR6397945	Canada
SRR6397946	Canada
SRR6397947	Canada
SRR6397948	Canada
SRR6397949	Canada
SRR6397950	Canada

SRR6397951	Canada
SRR6397952	Canada
SRR6397953	Canada
SRR6397954	Canada
SRR6397955	Canada
SRR6397956	Canada
SRR6397957	Canada
SRR6397958	Canada
SRR6397959	Canada
SRR6397960	Canada
SRR6397961	Canada
SRR6397962	Canada
SRR6397963	Canada
SRR6397964	Canada
SRR6397965	Canada
SRR6397966	Canada
SRR6397967	Canada
SRR6397968	Canada
SRR6397969	Canada
SRR6397970	Canada
SRR6397971	Canada
SRR6397972	Canada
SRR6397973	Canada
SRR6397974	Canada
SRR6397975	Canada
SRR6397976	Canada
SRR6397977	Canada
SRR6397978	Canada
SRR6397979	Canada
SRR6397980	Canada
SRR6397981	Canada
SRR6397982	Canada
SRR6397983	Canada
SRR6397984	Canada
SRR6397985	Canada
SRR6397986	Canada
SRR6397987	Canada
SRR6397988	Canada
SRR6397989	Canada
SRR6397990	Canada
SRR6397991	Canada
SRR6397992	Canada
SRR6397993	Canada
SRR6397994	Canada
SRR6397995	Canada
SRR6397996	Canada
SRR6397997	Canada
SRR6397998	Canada
SRR6397999	Canada
SRR6398000	Canada

SRR6398001	Canada
SRR6398002	Canada
SRR6398003	Canada
SRR6398004	Canada
SRR6398005	Canada
SRR6398006	Canada
SRR6398007	Canada
SRR6398008	Canada
SRR6398009	Canada
SRR6398010	Canada
SRR6398011	Canada
SRR6398012	Canada
SRR6398013	Canada
SRR6398014	Canada
SRR6398015	Canada
SRR6398016	Canada
SRR6398017	Canada
SRR6398018	Canada
SRR6398019	Canada
SRR6398020	Canada
SRR6398021	Canada
SRR6398022	Canada
SRR6398023	Canada
SRR6398024	Canada
SRR6398025	Canada
SRR6398026	Canada
SRR6398027	Canada
SRR6398028	Canada
SRR6398029	Canada
SRR6398030	Canada
SRR6398031	Canada
SRR6398032	Canada
SRR6398033	Canada
SRR6398034	Canada
SRR6398035	Canada
SRR6398036	Canada
SRR6398037	Canada
SRR6398038	Canada
SRR6398039	Canada
SRR6398040	Canada
SRR6398041	Canada
SRR6398042	Canada
SRR6398043	Canada
SRR6398044	Canada
SRR6398045	Canada
SRR6398046	Canada
SRR6398047	Canada
SRR6398048	Canada
SRR6398049	Canada
SRR6398050	Canada

SRR6398051	Canada
SRR6398052	Canada
SRR6398053	Canada
SRR6398054	Canada
SRR6398055	Canada
SRR6398056	Canada
SRR6398057	Canada
SRR6398058	Canada
SRR6398059	Canada
SRR6398060	Canada
SRR6398061	Canada
SRR6398062	Canada
SRR6398063	Canada
SRR6398064	Canada
SRR6398065	Canada
SRR6398066	Canada
SRR6398067	Canada
SRR6398068	Canada
SRR6398069	Canada
SRR6398070	Canada
SRR6398071	Canada
SRR6398072	Canada
SRR6398073	Canada
SRR6398074	Canada
SRR6398075	Canada
SRR6398076	Canada
SRR6398077	Canada
SRR6398078	Canada
SRR6398079	Canada
SRR6398080	Canada
SRR6398081	Canada
SRR6398082	Canada
SRR6398083	Canada
SRR6398084	Canada
SRR6398085	Canada
SRR6398086	Canada
SRR6398087	Canada
SRR6398088	Canada
SRR6398089	Canada
SRR6398090	Canada
SRR6398091	Canada
SRR6398092	Canada
SRR6398093	Canada
SRR6398094	Canada
SRR6398095	Canada
SRR6398096	Canada
SRR6398097	Canada
SRR6398098	Canada
SRR6398099	Canada
SRR6398100	Canada

SRR6398101	Canada
SRR6398102	Canada
SRR6398103	Canada
SRR6398104	Canada
SRR6398105	Canada
SRR6398106	Canada
SRR6398107	Canada
SRR6398108	Canada
SRR6398109	Canada
SRR6398110	Canada
SRR6398111	Canada
SRR6398112	Canada
SRR6398113	Canada
SRR6398114	Canada
SRR6398115	Canada
SRR6398116	Canada
SRR6398117	Canada
SRR6398118	Canada
SRR6398119	Canada
SRR6398120	Canada
SRR6398121	Canada
SRR6398122	Canada
SRR6398123	Canada
SRR6398124	Canada
SRR6398125	Canada
SRR6398126	Canada
SRR6398127	Canada
SRR6398128	Canada
SRR6398129	Canada
SRR6398130	Canada
SRR6398131	Canada
SRR6398132	Canada
SRR6398133	Canada
SRR6398134	Canada
SRR6398135	Canada
SRR6398136	Canada
SRR6398137	Canada
SRR6398138	Canada
SRR6398139	Canada
SRR6398140	Canada
SRR6398141	Canada
SRR6398142	Canada
SRR6398143	Canada
SRR6398144	Canada
SRR6398145	Canada
SRR6398146	Canada
SRR6398147	Canada
SRR6398148	Canada
SRR6458383	Belarus
SRR6458384	Belarus

SRR6458385	Belarus
SRR6458386	Belarus
SRR6458387	Belarus
SRR6458388	Belarus
SRR6458389	Belarus
SRR6458390	Belarus
SRR6458391	Belarus
SRR6458392	Belarus
SRR6458393	Belarus
SRR6458394	Belarus
SRR6458395	Belarus
SRR6458396	Belarus
SRR6458397	Belarus
SRR6458398	Belarus
SRR6458399	Belarus
SRR6458400	Belarus
SRR6458401	Belarus
SRR6458402	Belarus
SRR6458403	Belarus
SRR6458404	Belarus
SRR6458405	Belarus
SRR6458406	Belarus
SRR6458407	Belarus
SRR6458408	Belarus
SRR6458409	Belarus
SRR6458410	Belarus
SRR6458411	Belarus
SRR6458412	Belarus
SRR6458413	Belarus
SRR6458414	Belarus
SRR6458415	Belarus
SRR6458416	Belarus
SRR6458417	Belarus
SRR6458418	Belarus
SRR6458419	Belarus
SRR6458420	Belarus
SRR6458421	Belarus
SRR6458422	Belarus
SRR6458423	Belarus
SRR6458424	Belarus
SRR6458425	Belarus
SRR6458426	Belarus
SRR6458427	Belarus
SRR6458428	Belarus
SRR6458429	Belarus
SRR6458430	Belarus
SRR6458431	Belarus
SRR6458432	Belarus
SRR6458433	Belarus
SRR6458434	Belarus

SRR6458435	Belarus
SRR6458436	Belarus
SRR6458437	Belarus
SRR6458438	Belarus
SRR6458439	Belarus
SRR6458440	Belarus
SRR6458441	Belarus
SRR6458442	Belarus
SRR6458443	Belarus
SRR6458444	Belarus
SRR6458445	Belarus
SRR6458446	Belarus
SRR6458447	Belarus
SRR6458448	Belarus
SRR6458449	Belarus
SRR6458450	Belarus
SRR6458451	Belarus
SRR6458452	Belarus
SRR6458453	Belarus
SRR6458454	Belarus
SRR6458455	Belarus
SRR6458456	Belarus
SRR6458457	NA
SRR6458458	NA
SRR6458459	NA
SRR6458460	NA
SRR6458461	NA
SRR6458462	NA
SRR6458463	NA
SRR6458464	NA
SRR6479534	NA
SRR6479535	NA
SRR6479536	NA
SRR6479537	NA
SRR6479538	NA
SRR6479539	NA
SRR6479540	NA
SRR6479541	NA
SRR6479542	NA
SRR6479543	NA
SRR6479544	NA
SRR6479545	NA
SRR6479546	NA
SRR6480313	NA
SRR6480314	NA
SRR6480315	NA
SRR6480316	NA
SRR6480317	NA
SRR6480318	NA
SRR6480319	NA

SRR6480320	NA
SRR6480321	NA
SRR6480322	NA
SRR6480323	NA
SRR6480324	NA
SRR6480325	NA
SRR6480326	NA
SRR6480327	NA
SRR6480328	NA
SRR6480329	NA
SRR6480330	NA
SRR6480331	NA
SRR6480332	NA
SRR6480333	NA
SRR6480334	NA
SRR6480335	NA
SRR6480336	NA
SRR6480337	NA
SRR6480338	NA
SRR6480339	NA
SRR6480340	NA
SRR6480341	NA
SRR6480342	NA
SRR6480343	NA
SRR6480344	NA
SRR6480345	NA
SRR6480346	NA
SRR6480347	NA
SRR6480348	NA
SRR6480349	NA
SRR6480350	NA
SRR6480351	NA
SRR6480352	NA
SRR6480353	NA
SRR6480354	NA
SRR6480355	NA
SRR6480356	NA
SRR6480357	Indonesia
SRR6480358	Indonesia
SRR6480359	Indonesia
SRR6480360	Indonesia
SRR6480361	Indonesia
SRR6480362	Indonesia
SRR6480363	Indonesia
SRR6480364	Indonesia
SRR6480365	Indonesia
SRR6480366	Indonesia
SRR6480367	Indonesia
SRR6480368	Indonesia
SRR6480369	Indonesia

SRR6480370	Indonesia
SRR6480371	Indonesia
SRR6480372	Indonesia
SRR6480373	Indonesia
SRR6480374	Indonesia
SRR6480375	Indonesia
SRR6480376	Indonesia
SRR6480377	Indonesia
SRR6480378	Indonesia
SRR6480379	Indonesia
SRR6480380	Indonesia
SRR6480381	Indonesia
SRR6480382	Indonesia
SRR6480383	Indonesia
SRR6480384	Indonesia
SRR6480385	Indonesia
SRR6480386	Indonesia
SRR6480387	Indonesia
SRR6480388	Indonesia
SRR6480389	Indonesia
SRR6480390	Indonesia
SRR6480391	Indonesia
SRR6480392	Indonesia
SRR6480393	Indonesia
SRR6480394	Indonesia
SRR6480395	Indonesia
SRR6480396	Indonesia
SRR6480397	Indonesia
SRR6480398	Indonesia
SRR6480399	Indonesia
SRR6480400	Indonesia
SRR6480401	Indonesia
SRR6480402	Indonesia
SRR6480403	Indonesia
SRR6480404	Indonesia
SRR6480405	Indonesia
SRR6480406	Indonesia
SRR6480407	Indonesia
SRR6480408	Indonesia
SRR6480409	Indonesia
SRR6480410	Indonesia
SRR6480411	Indonesia
SRR6480412	Indonesia
SRR6480413	Indonesia
SRR6480414	Indonesia
SRR6480415	Indonesia
SRR6480416	Indonesia
SRR6480417	Indonesia
SRR6480418	Indonesia
SRR6480419	Indonesia

SRR6480420	Indonesia
SRR6480421	Indonesia
SRR6480422	Indonesia
SRR6480423	Indonesia
SRR6480424	Indonesia
SRR6480425	Indonesia
SRR6480426	Indonesia
SRR6480427	Indonesia
SRR6480428	Indonesia
SRR6480429	Indonesia
SRR6480430	Indonesia
SRR6480431	Indonesia
SRR6480432	Indonesia
SRR6480433	Indonesia
SRR6480434	Indonesia
SRR6480435	Indonesia
SRR6480436	Indonesia
SRR6480437	Indonesia
SRR6480438	Indonesia
SRR6480439	Indonesia
SRR6480440	Indonesia
SRR6480441	Indonesia
SRR6480442	Indonesia
SRR6480443	Indonesia
SRR6480444	Indonesia
SRR6480445	Indonesia
SRR6480446	Indonesia
SRR6480447	Indonesia
SRR6480448	Indonesia
SRR6480449	Indonesia
SRR6480450	Indonesia
SRR6480451	Indonesia
SRR6480452	Indonesia
SRR6480453	Indonesia
SRR6480454	Indonesia
SRR6480455	Indonesia
SRR6480456	Indonesia
SRR6480457	Indonesia
SRR6480458	Indonesia
SRR6480459	Indonesia
SRR6480460	Indonesia
SRR6480461	Indonesia
SRR6480462	Indonesia
SRR6480463	Indonesia
SRR6480464	Indonesia
SRR6480465	Indonesia
SRR6480466	Indonesia
SRR6480467	Indonesia
SRR6480468	Indonesia
SRR6480469	Indonesia

SRR6480470	Indonesia
SRR6480471	Indonesia
SRR6480472	Indonesia
SRR6480473	Indonesia
SRR6480474	Indonesia
SRR6480475	Indonesia
SRR6480476	Indonesia
SRR6480477	Indonesia
SRR6480478	Indonesia
SRR6480479	Indonesia
SRR6480480	Indonesia
SRR6480481	Indonesia
SRR6480482	Indonesia
SRR6480483	Indonesia
SRR6480484	Indonesia
SRR6480485	Indonesia
SRR6480486	Indonesia
SRR6480487	Indonesia
SRR6480488	Indonesia
SRR6480489	Indonesia
SRR6480490	Indonesia
SRR6480491	Indonesia
SRR6480492	Indonesia
SRR6480493	Indonesia
SRR6480494	Indonesia
SRR6480495	Indonesia
SRR6480496	Indonesia
SRR6480497	Indonesia
SRR6480498	Indonesia
SRR6480499	Indonesia
SRR6480500	Indonesia
SRR6480501	Indonesia
SRR6480502	Indonesia
SRR6480503	Indonesia
SRR6480504	Indonesia
SRR6480505	Indonesia
SRR6480506	Indonesia
SRR6480507	Indonesia
SRR6480508	Indonesia
SRR6480509	Indonesia
SRR6480510	Indonesia
SRR6480511	Indonesia
SRR6480512	Indonesia
SRR6480513	Indonesia
SRR6480514	Indonesia
SRR6480515	Indonesia
SRR6480516	Indonesia
SRR6480517	Indonesia
SRR6480518	Indonesia
SRR6480519	Indonesia

SRR6480520	Indonesia
SRR6480521	Indonesia
SRR6480522	Indonesia
SRR6480523	Indonesia
SRR6480524	Indonesia
SRR6480525	Indonesia
SRR6480526	Indonesia
SRR6480527	Indonesia
SRR6480528	Indonesia
SRR6480529	Indonesia
SRR6480530	Indonesia
SRR6480531	Indonesia
SRR6480532	Indonesia
SRR6480533	Indonesia
SRR6480534	Indonesia
SRR6480535	Indonesia
SRR6480536	Indonesia
SRR6480537	Indonesia
SRR6480538	Indonesia
SRR6480539	Indonesia
SRR6480540	Indonesia
SRR6480541	Indonesia
SRR6480542	Indonesia
SRR6480543	Indonesia
SRR6480544	Indonesia
SRR6480545	Indonesia
SRR6480546	Indonesia
SRR6480547	Indonesia
SRR6480548	Indonesia
SRR6480549	Indonesia
SRR6480550	Indonesia
SRR6480551	Indonesia
SRR6480552	Indonesia
SRR6480553	Indonesia
SRR6480554	Indonesia
SRR6480555	Indonesia
SRR6480556	Indonesia
SRR6480557	Indonesia
SRR6480558	Indonesia
SRR6480559	Indonesia
SRR6480560	Indonesia
SRR6480561	Indonesia
SRR6480562	Indonesia
SRR6480563	Indonesia
SRR6480564	Indonesia
SRR6480565	Indonesia
SRR6480566	Indonesia
SRR6480567	Indonesia
SRR6480568	Indonesia
SRR6480569	Indonesia

SRR6480570	Indonesia
SRR6480571	Indonesia
SRR6480572	Indonesia
SRR6480573	Indonesia
SRR6480574	Indonesia
SRR6480575	Indonesia
SRR6480576	Indonesia
SRR6480577	Indonesia
SRR6480578	Indonesia
SRR6480579	Indonesia
SRR6480580	Indonesia
SRR6480581	Indonesia
SRR6480582	Indonesia
SRR6480583	Indonesia
SRR6480584	Indonesia
SRR6480585	Indonesia
SRR6480586	Indonesia
SRR6480587	Indonesia
SRR6480588	Indonesia
SRR6480589	Indonesia
SRR6480590	Indonesia
SRR6480591	Indonesia
SRR6480592	Indonesia
SRR6480593	Indonesia
SRR6480594	Indonesia
SRR6480595	Indonesia
SRR6480596	Indonesia
SRR6480597	Indonesia
SRR6480598	Indonesia
SRR6480599	Indonesia
SRR6480600	Indonesia
SRR6480601	Indonesia
SRR6480602	Indonesia
SRR6480603	Indonesia
SRR6480604	Indonesia
SRR6480605	Indonesia
SRR6480606	Indonesia
SRR6480607	Indonesia
SRR6480608	Indonesia
SRR6480609	Indonesia
SRR6480610	Indonesia
SRR6480611	Indonesia
SRR6480612	Indonesia
SRR6480613	Indonesia
SRR6480614	Indonesia
SRR6480615	Indonesia
SRR6480616	Indonesia
SRR6480617	Indonesia
SRR6480618	Indonesia
SRR6480619	Indonesia

SRR6480620	Indonesia
SRR6480621	Indonesia
SRR6480622	Indonesia
SRR6480623	Indonesia
SRR6480624	Indonesia
SRR6480625	Indonesia
SRR6480626	Indonesia
SRR6480627	Indonesia
SRR6480628	Indonesia
SRR6480629	Indonesia
SRR6480630	Indonesia
SRR6480631	Indonesia
SRR6480632	Indonesia
SRR6480633	Indonesia
SRR6480634	Indonesia
SRR6487127	South Africa
SRR671719	NA
SRR671720	NA
SRR671721	NA
SRR671722	NA
SRR671723	NA
SRR671724	NA
SRR671725	NA
SRR671726	NA
SRR671727	NA
SRR671728	NA
SRR671729	NA
SRR671730	NA
SRR671731	NA
SRR671732	NA
SRR671733	NA
SRR671734	NA
SRR671735	NA
SRR671736	NA
SRR671737	NA
SRR671738	NA
SRR671739	NA
SRR671740	NA
SRR671741	NA
SRR671742	NA
SRR671743	NA
SRR671744	NA
SRR671745	NA
SRR671746	NA
SRR671747	NA
SRR671748	NA
SRR671749	NA
SRR671750	NA
SRR671751	NA
SRR671752	NA

SRR671753	NA
SRR671754	NA
SRR671755	NA
SRR671756	NA
SRR671757	NA
SRR671758	NA
SRR671759	NA
SRR671760	NA
SRR671761	NA
SRR671762	NA
SRR671763	NA
SRR671764	NA
SRR671765	NA
SRR671766	NA
SRR671767	NA
SRR671768	NA
SRR671769	NA
SRR671770	NA
SRR671771	NA
SRR671772	NA
SRR671773	NA
SRR671774	NA
SRR671775	NA
SRR671776	NA
SRR671777	NA
SRR671778	NA
SRR671779	NA
SRR671780	NA
SRR671781	NA
SRR671782	NA
SRR671783	NA
SRR671784	NA
SRR671785	NA
SRR671786	NA
SRR671787	NA
SRR671788	NA
SRR671789	NA
SRR671790	NA
SRR671791	NA
SRR671792	NA
SRR671793	NA
SRR671794	NA
SRR671795	NA
SRR671796	NA
SRR671797	NA
SRR671798	NA
SRR671799	NA
SRR671800	NA
SRR671801	NA
SRR671802	NA

SRR671803	NA
SRR671804	NA
SRR671805	NA
SRR671806	NA
SRR671807	NA
SRR671808	NA
SRR671809	NA
SRR671810	NA
SRR671811	NA
SRR671812	NA
SRR671813	NA
SRR671814	NA
SRR671815	NA
SRR671816	NA
SRR671817	NA
SRR671818	NA
SRR671819	NA
SRR671820	NA
SRR671821	NA
SRR671822	NA
SRR671823	NA
SRR671824	NA
SRR671825	NA
SRR671826	NA
SRR671827	NA
SRR671828	NA
SRR671829	NA
SRR671830	NA
SRR671831	NA
SRR671832	NA
SRR671833	NA
SRR671834	NA
SRR671835	NA
SRR671836	NA
SRR671837	NA
SRR671838	NA
SRR671839	NA
SRR671840	NA
SRR671841	NA
SRR671842	NA
SRR671843	NA
SRR671844	NA
SRR671845	NA
SRR671846	NA
SRR671847	NA
SRR671848	NA
SRR671849	NA
SRR671850	NA
SRR671851	NA
SRR671852	NA

SRR671853	NA
SRR671854	NA
SRR671855	NA
SRR671856	NA
SRR671857	NA
SRR671858	NA
SRR671859	NA
SRR671860	NA
SRR671861	NA
SRR671862	NA
SRR671863	NA
SRR671864	NA
SRR671865	NA
SRR671866	NA
SRR671867	NA
SRR671868	NA
SRR671869	NA
SRR671870	NA
SRR671871	NA
SRR671872	NA
SRR671873	NA
SRR671874	NA
SRR671875	NA
SRR671876	NA
SRR671877	NA
SRR671878	NA
SRR671879	NA
SRR6789614	Botswana
SRR6789615	Botswana
SRR6789616	Botswana
SRR6789617	Botswana
SRR6789618	Botswana
SRR6789619	Botswana
SRR6789620	Botswana
SRR6789621	Botswana
SRR6789622	Botswana
SRR6789623	Botswana
SRR6789624	Botswana
SRR6789625	Botswana
SRR6789626	Botswana
SRR6789627	Botswana
SRR6789628	Botswana
SRR6789629	Botswana
SRR6789630	Botswana
SRR6789631	Botswana
SRR6789632	Botswana
SRR6789633	Botswana
SRR6789634	Botswana
SRR6789635	Botswana
SRR6789636	Botswana

SRR6789637	Botswana
SRR6807669	Moldova
SRR6807670	Moldova
SRR6807671	Moldova
SRR6807672	Moldova
SRR6807673	Moldova
SRR6807674	Moldova
SRR6807675	Moldova
SRR6807676	Moldova
SRR6807677	Moldova
SRR6807678	Moldova
SRR6807679	Moldova
SRR6807680	Moldova
SRR6807681	Moldova
SRR6807682	Moldova
SRR6807683	Moldova
SRR6807684	Moldova
SRR6807685	Moldova
SRR6807686	Moldova
SRR6807687	Moldova
SRR6807688	Moldova
SRR6807689	Moldova
SRR6807690	Moldova
SRR6807691	Moldova
SRR6807692	Moldova
SRR6807693	Moldova
SRR6807694	Moldova
SRR6807695	Moldova
SRR6807696	Moldova
SRR6807697	Moldova
SRR6807698	Moldova
SRR6807699	Moldova
SRR6807700	Moldova
SRR6807701	Moldova
SRR6807702	Moldova
SRR6807703	Moldova
SRR6807704	Moldova
SRR6807705	Moldova
SRR6807706	Moldova
SRR6807707	Moldova
SRR6807708	Moldova
SRR6807709	Moldova
SRR6807710	Moldova
SRR6807711	Moldova
SRR6807712	Moldova
SRR6807713	Moldova
SRR6807714	Moldova
SRR6807715	Moldova
SRR6807716	Moldova
SRR6807717	Moldova

SRR6807718	Moldova
SRR6807719	Moldova
SRR6807720	Moldova
SRR6807721	Moldova
SRR6807722	Moldova
SRR6807723	Moldova
SRR6807724	Moldova
SRR6807725	Moldova
SRR6807726	Moldova
SRR6807727	Moldova
SRR6807728	Moldova
SRR6807729	Moldova
SRR6807730	Moldova
SRR6807731	Moldova
SRR6807732	Moldova
SRR6807733	Moldova
SRR6807734	Moldova
SRR6807735	Moldova
SRR6807736	Moldova
SRR6807737	Moldova
SRR6807738	Moldova
SRR6807739	Moldova
SRR6807740	Moldova
SRR6807741	Moldova
SRR6807742	Moldova
SRR6807743	Moldova
SRR6807744	Moldova
SRR6807745	Moldova
SRR6807746	Moldova
SRR6807747	Moldova
SRR6807748	Moldova
SRR6807749	Moldova
SRR6807750	Moldova
SRR6807751	Moldova
SRR6807752	Moldova
SRR6807753	Moldova
SRR6807754	Moldova
SRR6807755	Moldova
SRR6807756	Moldova
SRR6807757	Moldova
SRR6807758	Moldova
SRR6807759	Moldova
SRR6807760	Moldova
SRR6807761	Moldova
SRR6807762	Moldova
SRR6807763	Moldova
SRR6848320	Argentina
SRR6848321	Argentina
SRR6848322	Argentina
SRR6848323	Argentina

SRR6854705	Italy
SRR6854706	Italy
SRR6914118	Russia
SRR6914119	Russia
SRR6914120	Russia
SRR6914121	Russia
SRR6914122	Russia
SRR6914123	Russia
SRR6916544	Azerbaijan
SRR7131013	Italy
SRR7131014	Italy
SRR7131036	Italy
SRR7131042	Italy
SRR7131043	Italy
SRR7131045	Italy
SRR7131076	Italy
SRR7131082	Italy
SRR7131086	Italy
SRR7131087	Italy
SRR7131188	Italy
SRR7131200	Italy
SRR7131262	Italy
SRR7131265	Italy
SRR7131266	Italy
SRR7131271	Italy
SRR7131272	Italy
SRR7232581	Australia
SRR7249136	NA
SRR7249137	NA
SRR7249138	NA
SRR7249139	NA
SRR7249140	NA
SRR7341637	Romania
SRR7341638	Romania
SRR7341639	Romania
SRR7341640	Romania
SRR7341641	Romania
SRR7341642	Romania
SRR7341643	Romania
SRR7341644	Romania
SRR7341645	Romania
SRR7341646	Romania
SRR7341647	Romania
SRR7341648	Romania
SRR7341649	Romania
SRR7341650	Romania
SRR7341651	Romania
SRR7341652	Romania
SRR7341653	Romania
SRR7341654	Romania

SRR7341655	Romania
SRR7341656	Romania
SRR7341657	Romania
SRR7341658	Romania
SRR7341659	Romania
SRR7341660	Romania
SRR7341661	Romania
SRR7341662	Romania
SRR7341663	Romania
SRR7341664	Romania
SRR7341665	Romania
SRR7341666	Romania
SRR7341667	Romania
SRR7341668	Romania
SRR7341669	Romania
SRR7341670	Romania
SRR7341671	Romania
SRR7341672	Romania
SRR7341673	Romania
SRR7341674	Romania
SRR7341675	Romania
SRR7341676	Romania
SRR7341677	Romania
SRR7341678	Romania
SRR7341679	Romania
SRR7341680	Romania
SRR7341681	Romania
SRR7341682	Romania
SRR7341683	Romania
SRR7341684	Romania
SRR7341685	Romania
SRR7341686	Romania
SRR7341687	Romania
SRR7341688	Romania
SRR7341689	Romania
SRR7341690	Romania
SRR7341691	Romania
SRR7341692	Romania
SRR7341693	Romania
SRR7341694	Romania
SRR7341695	Romania
SRR7341696	Romania
SRR7341697	Romania
SRR7341698	Romania
SRR7341699	Romania
SRR7341700	Romania
SRR7341701	Romania
SRR7341702	Romania
SRR7341703	Romania
SRR7341704	Romania

SRR7341705	Romania
SRR7341706	Romania
SRR7341707	Romania
SRR7341708	Romania
SRR7439412	Canada
SRR7439413	Canada
SRR7439416	Canada
SRR7439417	Canada
SRR7496471	United States of America
SRR7496472	United States of America
SRR7496473	United States of America
SRR7496474	United States of America
SRR7496475	United States of America
SRR7496476	United States of America
SRR7496477	United States of America
SRR7496478	United States of America
SRR7496479	United States of America
SRR7496480	United States of America
SRR7496481	United States of America
SRR7496482	United States of America
SRR7496483	United States of America
SRR7496484	United States of America
SRR7496485	United States of America
SRR7496486	United States of America
SRR7496487	United States of America
SRR7496488	United States of America
SRR7496489	United States of America
SRR7496490	United States of America
SRR7496491	United States of America
SRR7496492	United States of America
SRR7496493	United States of America
SRR7496494	United States of America
SRR7496495	United States of America
SRR7496496	United States of America
SRR7496497	United States of America
SRR7496498	United States of America
SRR7496499	United States of America
SRR7496500	United States of America
SRR7496501	United States of America
SRR7496502	United States of America
SRR7496503	United States of America
SRR7496504	United States of America
SRR7496505	United States of America
SRR7496506	United States of America
SRR7496507	United States of America
SRR7496508	United States of America
SRR7496509	United States of America
SRR7496510	United States of America
SRR7496511	United States of America
SRR7496512	United States of America

SRR7496513	United States of America
SRR7496514	United States of America
SRR7496515	United States of America
SRR7496516	United States of America
SRR7496517	United States of America
SRR7496518	United States of America
SRR7496519	United States of America
SRR7496520	United States of America
SRR7496521	United States of America
SRR7496522	United States of America
SRR7496523	United States of America
SRR7496524	United States of America
SRR7496525	United States of America
SRR7496526	United States of America
SRR7496527	United States of America
SRR7496528	United States of America
SRR7496529	United States of America
SRR7496530	United States of America
SRR7496531	United States of America
SRR7496532	United States of America
SRR7496533	United States of America
SRR7496534	United States of America
SRR7496535	United States of America
SRR7496536	United States of America
SRR7496537	United States of America
SRR7496538	United States of America
SRR7496539	United States of America
SRR7496540	United States of America
SRR7496541	United States of America
SRR7496543	United States of America
SRR7496544	United States of America
SRR7496545	United States of America
SRR7496546	United States of America
SRR7496547	United States of America
SRR7496548	United States of America
SRR7496549	United States of America
SRR7496550	United States of America
SRR7496551	United States of America
SRR7496552	United States of America
SRR7496553	United States of America
SRR7496554	United States of America
SRR7516288	Georgia
SRR7516289	Georgia
SRR7516290	Georgia
SRR7516291	Georgia
SRR7516292	Georgia
SRR7516293	Georgia
SRR7516294	Georgia
SRR7516295	Georgia
SRR7516296	Georgia

SRR7516297	Georgia
SRR7516298	Georgia
SRR7516299	Georgia
SRR7516300	Georgia
SRR7516301	Georgia
SRR7516302	Georgia
SRR7516303	Georgia
SRR7516304	Georgia
SRR7516305	Georgia
SRR7516306	Georgia
SRR7516307	Georgia
SRR7516308	Georgia
SRR7516309	Georgia
SRR7516310	Georgia
SRR7516311	Georgia
SRR7516312	Georgia
SRR7516313	Georgia
SRR7516314	Georgia
SRR7516315	Georgia
SRR7516316	Georgia
SRR7516317	Georgia
SRR7516318	Georgia
SRR7516319	Georgia
SRR7516320	Georgia
SRR7516321	Georgia
SRR7516322	Georgia
SRR7516323	Georgia
SRR7516324	Georgia
SRR7516325	Georgia
SRR7516326	Georgia
SRR7516327	Georgia
SRR7516328	Georgia
SRR7516329	Georgia
SRR7516330	Georgia
SRR7516331	Georgia
SRR7516332	Georgia
SRR7516333	Georgia
SRR7516334	Georgia
SRR7516335	Georgia
SRR7516336	Georgia
SRR7516337	Georgia
SRR7516338	Georgia
SRR7516339	Georgia
SRR7516340	Georgia
SRR7516341	Georgia
SRR7516342	Georgia
SRR7516343	Georgia
SRR7516344	Georgia
SRR7516345	Georgia
SRR7516346	Georgia

SRR7516347	Georgia
SRR7516348	Georgia
SRR7516349	Georgia
SRR7516350	Georgia
SRR7516351	Georgia
SRR7516352	Georgia
SRR7516353	Georgia
SRR7516354	Georgia
SRR7516355	Georgia
SRR7516356	Georgia
SRR7516357	Georgia
SRR7516358	Georgia
SRR7516359	Georgia
SRR7516360	Georgia
SRR7516361	Georgia
SRR7516362	Georgia
SRR7516363	Georgia
SRR7516364	Georgia
SRR7516365	Georgia
SRR7516366	Georgia
SRR7516367	Georgia
SRR7516368	Georgia
SRR7516369	Georgia
SRR7516370	Georgia
SRR7516371	Georgia
SRR7516372	Georgia
SRR7516373	Georgia
SRR7516374	Georgia
SRR7516375	Georgia
SRR7516376	Georgia
SRR7516377	Georgia
SRR7516378	Georgia
SRR7516379	Georgia
SRR7516380	Georgia
SRR7516381	Georgia
SRR7516382	Georgia
SRR7516383	Georgia
SRR7516384	Georgia
SRR7516385	Georgia
SRR7516386	Georgia
SRR7516387	Georgia
SRR7516388	Georgia
SRR7516389	Georgia
SRR7516390	Georgia
SRR7516391	Georgia
SRR7516392	Georgia
SRR7516393	Georgia
SRR7516394	Georgia
SRR7516395	Georgia
SRR7516396	Georgia

SRR7516397	Georgia
SRR7516398	Georgia
SRR7516399	Georgia
SRR7516400	Georgia
SRR7516401	Georgia
SRR7516402	Georgia
SRR7516403	Georgia
SRR7516404	Georgia
SRR7516405	Georgia
SRR7516406	Georgia
SRR7516407	Georgia
SRR7516408	Georgia
SRR7516409	Georgia
SRR7516410	Georgia
SRR7516411	Georgia
SRR7516412	Georgia
SRR7516413	Georgia
SRR7516414	Georgia
SRR7516415	Georgia
SRR7516416	Georgia
SRR7516417	Georgia
SRR7516418	Georgia
SRR7516419	Georgia
SRR7516420	Georgia
SRR7516421	Georgia
SRR7516422	Georgia
SRR7516423	Georgia
SRR7516424	Georgia
SRR7516425	Georgia
SRR7516426	Georgia
SRR7516427	Georgia
SRR7516428	Georgia
SRR7516429	Georgia
SRR7516430	Georgia
SRR7516431	Georgia
SRR7516432	Georgia
SRR7516433	Georgia
SRR7516434	Georgia
SRR7516435	Georgia
SRR7516436	Georgia
SRR7516437	Georgia
SRR7516438	Georgia
SRR7516439	Georgia
SRR7516440	Georgia
SRR7516441	Georgia
SRR7516442	Georgia
SRR7516443	Georgia
SRR7516444	Georgia
SRR7516445	Georgia
SRR7516446	Georgia

SRR7516447	Georgia
SRR7516448	Georgia
SRR7516449	Georgia
SRR7516450	Georgia
SRR7516451	Georgia
SRR7516452	Georgia
SRR7516453	Georgia
SRR7516454	Georgia
SRR7516455	Georgia
SRR7516456	Georgia
SRR7516457	Georgia
SRR7516458	Georgia
SRR7516459	Georgia
SRR7516460	Georgia
SRR7517678	Democratic Republic of the Congo
SRR7517679	Cote d'Ivoire
SRR7517680	Peru
SRR7517681	Cote d'Ivoire
SRR7517682	Cote d'Ivoire
SRR7517683	Cote d'Ivoire
SRR7517684	Cote d'Ivoire
SRR7517685	Cote d'Ivoire
SRR7517686	Cote d'Ivoire
SRR7517687	Cote d'Ivoire
SRR7517688	Switzerland
SRR7517689	Switzerland
SRR7517690	Switzerland
SRR7517691	Switzerland
SRR7517692	Switzerland
SRR7517693	Switzerland
SRR7517694	Switzerland
SRR7517695	Switzerland
SRR7517696	Switzerland
SRR7517697	Switzerland
SRR7517698	Switzerland
SRR7517699	Switzerland
SRR7517700	Switzerland
SRR7517701	Switzerland
SRR7517702	Switzerland
SRR7517703	Switzerland
SRR7517704	Switzerland
SRR7517705	Switzerland
SRR7517706	Switzerland
SRR7517707	Switzerland
SRR7517708	Switzerland
SRR7517709	Switzerland
SRR7517710	Switzerland
SRR7517711	Switzerland
SRR7517712	Switzerland
SRR7517713	Switzerland

SRR7517714	Switzerland
SRR7517715	Switzerland
SRR7517716	Switzerland
SRR7517717	Switzerland
SRR7517718	Switzerland
SRR7517719	Switzerland
SRR7517720	Switzerland
SRR7517721	Switzerland
SRR7517722	Switzerland
SRR7517723	Switzerland
SRR7517724	Switzerland
SRR7517725	Cote d'Ivoire
SRR7517726	Switzerland
SRR7517727	Switzerland
SRR7517728	Switzerland
SRR7517729	Switzerland
SRR7517730	Switzerland
SRR7517731	Switzerland
SRR7517732	Switzerland
SRR7517733	Cote d'Ivoire
SRR7517734	Switzerland
SRR7517735	Switzerland
SRR7517736	Switzerland
SRR7517737	Switzerland
SRR7517738	Switzerland
SRR7517739	Thailand
SRR7517740	Switzerland
SRR7517741	Peru
SRR7517742	Thailand
SRR7517743	Thailand
SRR7517744	Cote d'Ivoire
SRR7517745	South Africa
SRR7517746	Thailand
SRR7517747	Switzerland
SRR7517748	Switzerland
SRR7517749	Switzerland
SRR7517750	Switzerland
SRR7517751	Switzerland
SRR7517752	Switzerland
SRR7517753	Switzerland
SRR7517754	Switzerland
SRR7517755	Switzerland
SRR7517756	Switzerland
SRR7517757	Switzerland
SRR7517758	Switzerland
SRR7517759	Democratic Republic of the Congo
SRR7517760	Cote d'Ivoire
SRR7517761	Switzerland
SRR7517762	Switzerland
SRR7517763	Switzerland

SRR7517764	NA
SRR7517765	NA
SRR7517766	NA
SRR7517767	NA
SRR7517768	NA
SRR7517769	NA
SRR7517770	NA
SRR7517771	NA
SRR7517772	NA
SRR7517773	NA
SRR7517774	NA
SRR7517775	NA
SRR7517776	NA
SRR7517777	NA
SRR7517778	NA
SRR7517779	NA
SRR7517780	NA
SRR7517781	NA
SRR7517782	NA
SRR7517783	NA
SRR7517784	NA
SRR7517785	NA
SRR7517786	NA
SRR7517787	NA
SRR7517788	NA
SRR7517789	NA
SRR7517790	NA
SRR7517791	NA
SRR7517792	NA
SRR7517793	NA
SRR7517794	NA
SRR7517795	NA
SRR7517796	NA
SRR7517797	NA
SRR7517798	NA
SRR7517799	NA
SRR7517800	NA
SRR7517801	NA
SRR7517802	NA
SRR7517803	NA
SRR7517804	NA
SRR7517805	NA
SRR7517806	NA
SRR7517807	NA
SRR7517808	NA
SRR7517809	NA
SRR7517810	NA
SRR7517811	NA
SRR7517812	NA
SRR7517813	NA

SRR7517814	NA
SRR7517815	NA
SRR7517816	NA
SRR7517817	NA
SRR7517818	NA
SRR7517819	NA
SRR7517820	NA
SRR7517821	NA
SRR7517822	NA
SRR7517823	NA
SRR7517824	NA
SRR7517825	NA
SRR7517826	NA
SRR7517827	NA
SRR7517828	NA
SRR7517829	NA
SRR7517830	NA
SRR7517831	NA
SRR7517832	NA
SRR7517833	NA
SRR7517834	NA
SRR7517835	NA
SRR7517836	Peru
SRR7535046	NA
SRR7535047	NA
SRR7535048	NA
SRR7535049	NA
SRR7535050	NA
SRR7535051	NA
SRR7535052	NA
SRR7535054	NA
SRR7535055	NA
SRR7535056	NA
SRR7535057	NA
SRR7535058	NA
SRR7535059	NA
SRR7535060	NA
SRR7535061	NA
SRR7535062	NA
SRR7535063	NA
SRR7535064	NA
SRR7535065	NA
SRR7535066	NA
SRR7535067	NA
SRR7535068	NA
SRR7535069	NA
SRR7535070	NA
SRR7535071	NA
SRR7535072	NA
SRR7535073	NA

SRR7535074	NA
SRR7535077	NA
SRR7535079	NA
SRR7535080	NA
SRR7535081	NA
SRR7535082	NA
SRR7535083	NA
SRR7535084	NA
SRR7535085	NA
SRR7535086	NA
SRR7535087	NA
SRR7535088	NA
SRR7535089	NA
SRR7535090	NA
SRR7535091	NA
SRR7535092	NA
SRR7535093	NA
SRR7535094	NA
SRR7539263	Myanmar
SRR7585395	NA
SRR7585396	NA
SRR7585397	NA
SRR7585398	NA
SRR7585399	NA
SRR7585400	NA
SRR7585401	NA
SRR7585402	NA
SRR7585403	NA
SRR7585404	NA
SRR7585405	NA
SRR7585406	NA
SRR7585407	NA
SRR7585408	NA
SRR7585409	NA
SRR7585410	NA
SRR7585411	NA
SRR7585412	NA
SRR7585413	NA
SRR7585414	NA
SRR7585415	NA
SRR7585416	NA
SRR7585417	NA
SRR7585418	NA
SRR7585419	NA
SRR7585420	NA
SRR7585421	NA
SRR7585422	NA
SRR7585423	NA
SRR7585424	NA
SRR7585425	NA

SRR7585426	NA
SRR7585427	NA
SRR7585428	NA
SRR7585429	NA
SRR7585430	NA
SRR7585431	NA
SRR7585432	NA
SRR7585433	NA
SRR7585434	NA
SRR7585435	NA
SRR7585436	NA
SRR7585437	NA
SRR7585438	NA
SRR7585439	NA
SRR7585440	NA
SRR7585441	NA
SRR7585442	NA
SRR7585443	NA
SRR7592322	Romania
SRR7592323	Romania
SRR7592324	Romania
SRR7592325	Romania
SRR784917	NA
SRR786188	NA
SRR786373	NA
SRR786397	NA
SRR786502	NA
SRR786503	NA
SRR786667	NA
SRR786668	NA
SRR786669	NA
SRR786670	NA
SRR896604	United States of America
SRR921502	NA
SRR921503	NA
