# Supplementary material for: Using genomics to understand the origin and dispersion of multidrug and extensively drug resistant tuberculosis in Portugal
Source: Sci Rep. 2020 Feb 13;10:2600. doi: 10.1038/s41598-020-59558-3 (PMC7018963; doi:10.1038/s41598-020-59558-3)
Supplement: Supplementary file 4 — Supplementary Information 4. [file 41598_2020_59558_MOESM4_ESM.pdf]

Supplementary Table S3 - Mutations detected across *loci* associated with drug resistance, distribution across drug susceptible and resistant isolates, positive predictive value (PPV) and statistical association with drug resistance. Statistical analysis were performed for mutations detected in at least two isolates with significant *p* values (< 0.01) highlighted in bold.

Drug	Mutation	No. of Isolates			PPV	*p* (Fisher Exact Test)	log OR	*p* (Z-test)
		R	S	Total				
AMK	eispromoter:C-12T	0	2	2	0.00	1.00000	2.14	0.21774
AMK	eispromoter:G-10A	8	28	36	0.22	**0.00251**	2.27	**0.00567**
AMK	rrs:1076insT	6	15	21	0.29	**0.00164**	2.61	**0.00255**
AMK	rrs:A807C	0	1	1				
AMK	rrs:A1401G	30	0	30	1.00	**0.00000**	7.62	**0.00000**
AMK	rrs:A906G	0	1	1				
AMK	rrs:C1402A	0	1	1				
AMK	rrs:G1484T	2	1	3	0.67	**0.00672**	4.22	**0.00295**
AMK	rrs:C492T	0	1	1				
AMK	rrs:C517T	1	0	1				
AMK	eispromoter:C-14T	1	0	1				
AMK	rrs:C774A	0	1	1				
AMK	rrs:C924T	0	2	2	0.00	1.00000	2.14	0.21774
AMK	None	2	68	70				
CAP	idsA2promoter:A-65G	17	4	21	0.81	**0.00000**	5.17	**0.00000**
CAP	rrs:1076insT	17	4	21	0.81	**0.00000**	5.17	**0.00000**
CAP	rrs:A807C	0	1	1				
CAP	tlyA:752insTG	17	1	18	0.94	**0.00000**	6.56	**0.00000**
CAP	idsA2promoter:C-98T	0	1	1				
CAP	idsA2promoter:G-97A	0	1	1				
CAP	rrs:A1401G	29	1	30	0.97	**0.00000**	7.09	**0.00000**
CAP	rrs:A906G	0	1	1				
CAP	rrs:C1402A	1	0	1				
CAP	rrs:G1484T	3	0	3	1.00	**0.00009**	5.52	**0.00107**
CAP	rrs:C492T	0	1	1				
CAP	rrs:C517T	0	1	1				
CAP	rrs:C774A	0	1	1				
CAP	rrs:C924T	0	2	2	0.00	1.00000	2.34	0.17767
CAP	None	2	83	85				
EMB	embA:Thr608Asn	1	0	1				
EMB	embB:Met306Ile	8	5	13	0.62	**0.00000**	3.82	**0.00003**
EMB	embR:Leu313Arg	1	0	1				
EMB	embA:Val961Phe	0	1	1				
EMB	embC:Arg738Gln	0	2	2	0.00	1.00000	1.96	0.25831
EMB	embApromoter:-32delG	1	0	1				
EMB	embB:Gln497Arg	3	2	5	0.60	**0.00228**	3.76	**0.00123**
EMB	embC:Val981Leu	3	15	18	0.17	0.08024	1.74	0.06922
EMB	embApromoter:C-11A	17	4	21	0.81	**0.00000**	4.80	**0.00000**
EMB	embApromoter:C-12A	17	4	21	0.81	**0.00000**	4.80	**0.00000**
EMB	embA:Gly582Arg	1	1	2	0.50	0.09672	3.35	0.03475
EMB	embB:Pro397Thr	17	4	21	0.81	**0.00000**	4.80	**0.00000**
EMB	embApromoter:C-16A	1	0	1				
EMB	embApromoter:C-16G	3	1	4	0.75	**0.00098**	4.45	**0.00108**
EMB	embB:Ser538Pro	1	0	1				
EMB	embApromoter:C-16T	14	1	15	0.93	**0.00000**	5.99	**0.00000**
EMB	embB:Met306Val	48	15	63	0.76	**0.00000**	4.51	**0.00000**
EMB	embB:Met423Thr	27	8	35	0.77	**0.00000**	4.57	**0.00000**
EMB	embB:178del3bp	0	2	2	0.00	1.00000	1.96	0.25831
EMB	embB:Asp1017Asn	0	1	1				
EMB	embB:Asp354Ala	2	0	2	1.00	**0.00328**	4.74	**0.00640**
EMB	ubiA:Leu172Pro	0	1	1				
EMB	embB:Gln497Lys	1	0	1				
EMB	ubiA:Val148Ala	1	0	1				
EMB	embB:Gly406Asp	4	1	5	0.80	**0.00011**	4.74	**0.00037**
EMB	embB:Gly406Cys	1	0	1				
EMB	embB:Gly443Ser	0	1	1				
EMB	embC:Ile487Thr	0	1	1				
EMB	embB:Lys1011Thr	0	1	1				
EMB	embB:Ser297Ala	1	1	2	0.50	0.09672	3.35	0.03475
EMB	embC:Leu766Pro	1	0	1				
EMB	ubiA:Ala249Ser	0	1	1				
EMB	ubiA:Ala38Pro	0	1	1				

EMB	ubiA:Ala38Val	1	0	1				
EMB	ubiA:Met180Val	1	0	1				
EMB	embC:Ala774Ser	0	2	2	0.00	1.00000	1.96	0.25831
EMB	embC:Arg879Gly	0	1	1				
EMB	Rv2820c:Leu212Val	1	2	3	0.33	0.14043	2.66	0.06142
EMB	Rv2820c:Lys114Asn	8	3	11	0.73	**0.00000**	4.33	**0.00001**
EMB	embApromoter:C-8A	0	1	1				
EMB	embB:Gln445Arg	0	1	1				
EMB	embB:Gly406Ala	1	1	2	0.50	0.09672	3.35	0.03475
EMB	embB:Leu402Val	0	1	1				
EMB	embB:Met306Leu	1	0	1				
EMB	ubiA:Met180Ile	1	0	1				
EMB	Rv2820c:Lys75Thr	0	1	1				
EMB	Rv3300c:Arg215Ser	0	1	1				
EMB	Rv3300c:Asp84Gly	1	1	2	0.50	0.09672	3.35	0.03475
EMB	Rv3300c:Pro295Ala	0	1	1				
EMB	ubiA:Ala181Val	0	1	1				
EMB	None	2	57	59				
ETH	ethA:112delT	1	0	1				
ETH	ethA:182delG	1	0	1				
ETH	ethA:343delT	1	0	1				
ETH	ethA:Ser266Arg	1	0	1				
ETH	ethA:493insGG	0	1	1				
ETH	ethA:770delC	1	0	1				
ETH	ethA:886delA	1	0	1				
ETH	ethA:933del36bp	0	1	1				
ETH	ethA:Ala185Thr	1	0	1				
ETH	ethA:Cys403Arg	2	0	2	1.00	0.02016	3.58	0.03442
ETH	ethA:Glu400Asp	1	0	1				
ETH	ethA:Gly450Asp	1	0	1				
ETH	ethA:Ile221Met	0	1	1				
ETH	ethA:Ile339Asn	0	1	1				
ETH	ethA:Leu194Pro	1	0	1				
ETH	ethA:Lys224Ter	1	0	1				
ETH	ethA:Phe264Leu	0	1	1				
ETH	ethA:Tyr147Ter	0	1	1				
ETH	fabG1promoter:C-15T	95	1	96	0.99	**0.00000**	6.75	**0.00000**
ETH	ethA:His281Pro	2	0	2	1.00	0.02016	3.58	0.03442
ETH	inhA:Ile194Thr	30	0	30	1.00	**0.00000**	6.29	**0.00005**
ETH	inhA:Ser94Ala	62	0	62	1.00	**0.00000**	7.02	**0.00001**
ETH	fabG1promoter:T-8C	1	0	1				
ETH	ethA:Met1Leu	1	0	1				
ETH	inhA:Ile21Val	1	0	1				
ETH	None	3	27	30				
FQ	gyrA:Asn83Lys	0	1	1				
FQ	gyrA:Glu21Gln	61	86	147	0.41	**0.00000**	3.09	**0.00003**
FQ	gyrA:Gly668Asp	60	83	143	0.42	**0.00000**	3.11	**0.00003**
FQ	gyrA:Ser95Thr	60	83	143	0.42	**0.00000**	3.11	**0.00003**
FQ	gyrA:Asp94Ala	12	0	12	1.00	**0.00000**	6.61	**0.00004**
FQ	gyrB:Val301Leu	14	18	32	0.44	**0.00000**	3.18	**0.00007**
FQ	gyrA:Asp94Asn	1	0	1				
FQ	gyrA:Asp94Gly	20	0	20	1.00	**0.00000**	7.12	**0.00001**
FQ	gyrA:Asp94Val	1	0	1				
FQ	gyrA:Gly247Ser	1	1	2	0.50	0.08951	3.43	0.03040
FQ	gyrA:His70Arg	1	1	2	0.50	0.08951	3.43	0.03040
FQ	gyrA:Pro472Ser	0	2	2	0.00	1.00000	2.05	0.23837
FQ	gyrA:Ser91Pro	21	0	21	1.00	**0.00000**	7.17	**0.00001**
FQ	gyrA:Thr80Ala	0	1	1				
FQ	gyrB:Asp461His	2	0	2	1.00	**0.00280**	4.82	**0.00551**
FQ	None	2	62	64				
INH	ahpC:Pro44Arg	3	1	4	0.75	0.07604	2.25	0.07080
INH	ahpCpromoter:C-52T	1	0	1				
INH	katG:687del6bp	1	0	1				
INH	ahpCpromoter:G-48A	2	0	2	1.00	0.07977	2.54	0.12364
INH	katG:Gly494Ala	1	0	1				
INH	ahpCpromoter:G-88A	2	0	2	1.00	0.07977	2.54	0.12364
INH	katG:Arg463Leu	20	1	21	0.95	**0.00000**	4.15	**0.00023**

INH	katG:Ser315Thr	55	0	55	1.00	**0.00000**	5.85	**0.00009**
INH	fabG1promoter:C-15T	116	0	116	1.00	**0.00000**	6.60	**0.00001**
INH	inhA:Ile194Thr	36	0	36	1.00	**0.00000**	5.43	**0.00029**
INH	inhA:Ser94Ala	71	0	71	1.00	**0.00000**	6.11	**0.00004**
INH	kasA:Asn400Ser	0	1	1				
INH	kasA:Gly269Ser	4	1	5	0.80	0.03124	2.54	0.03621
INH	inhA:Gly183Ser	0	1	1				
INH	kasA:Ser31Cys	1	0	1				
INH	katG:1947insC	1	0	1				
INH	katG:270insA	1	0	1				
INH	katG:Ser315Asn	1	0	1				
INH	fabG1promoter:T-8C	1	0	1				
INH	katG:Ser460Asn	1	0	1				
INH	inhA:Ile21Val	1	0	1				
INH	katG:Thr380Ile	1	0	1				
INH	katGpromoter:A-6G	1	0	1				
INH	None	6	19	25				
KAN	eispromoter:C-12T	0	1	1				
KAN	eispromoter:C-14T	1	0	1				
KAN	rrs:C517T	1	0	1				
KAN	eispromoter:G-10A	19	4	23	0.83	**0.00000**	6.12	**0.00006**
KAN	rrs:1076insT	7	4	11	0.64	**0.00000**	5.12	**0.00097**
KAN	rrs:A807C	0	1	1				
KAN	rrs:A1401G	21	1	22	0.95	**0.00000**	7.61	**0.00001**
KAN	rrs:A906G	0	1	1				
KAN	rrs:C1402A	1	0	1				
KAN	rrs:G1484T	3	0	3	1.00	**0.00005**	6.36	**0.00232**
KAN	rrs:C774A	0	1	1				
KAN	rrs:C924T	0	2	2	0.00	1.00000	3.18	0.13499
KAN	None	0	48	48				
PZA	pncA:251insG	5	0	5	1.00	**0.00001**	4.99	**0.00122**
PZA	pncA:283insA	1	0	1				
PZA	pncA:393insCC	2	0	2	1.00	**0.00614**	4.07	0.01284
PZA	pncA:416del3bp	2	0	2	1.00	**0.00614**	4.07	0.01284
PZA	pncA:440insCG	0	1	1				
PZA	pncA:452del13bp	1	0	1				
PZA	pncA:485ins10bp	1	0	1				
PZA	pncA:Asp49Asn	1	0	1				
PZA	pncA:Asp49Glu	0	1	1				
PZA	pncA:Asp63Ala	3	0	3	1.00	**0.00057**	4.48	**0.00472**
PZA	pncA:Asp8Glu	1	0	1				
PZA	pncA:Gln141Pro	1	0	1				
PZA	pncA:Glu15Ter	1	0	1				
PZA	pncA:Gly78Ser	1	0	1				
PZA	pncA:Gly97Cys	1	1	2	0.50	0.14123	2.69	0.06880
PZA	pncA:Ile133Ser	1	0	1				
PZA	pncA:Ile133Thr	2	0	2	1.00	**0.00614**	4.07	0.01284
PZA	pncA:Leu120Pro	16	0	16	1.00	**0.00000**	6.15	**0.00004**
PZA	pncA:Leu159Pro	1	0	1				
PZA	pncA:Leu172Pro	2	0	2	1.00	**0.00614**	4.07	0.01284
PZA	pncA:Leu182Ser	1	0	1				
PZA	pncA:Leu35Arg	1	0	1				
PZA	pncA:Leu4Ser	1	0	1				
PZA	pncA:Met1Thr	5	0	5	1.00	**0.00001**	4.99	**0.00122**
PZA	pncA:Phe13Ile	1	0	1				
PZA	pncA:Phe13Leu	3	0	3	1.00	**0.00057**	4.48	**0.00472**
PZA	pncA:Pro62Leu	1	0	1				
PZA	pncA:Pro69Leu	1	0	1				
PZA	pncA:Thr135Pro	1	0	1				
PZA	pncA:Thr76Ile	1	0	1				
PZA	pncA:Thr76Pro	1	0	1				
PZA	pncA:Tyr103Ter	1	0	1				
PZA	pncA:Tyr41Ter	1	0	1				
PZA	pncA:Tyr95Ter	1	0	1				
PZA	pncA:Val125Gly	35	0	35	1.00	**0.00000**	6.93	**0.00000**
PZA	pncA:Val155Gly	1	0	1				
PZA	pncA:Val155Leu	0	1	1				

PZA	pncA:Val180Leu	0	1	1				
PZA	pncApromoter:A-11C	1	0	1				
PZA	pncApromoter:A-11G	3	0	3	1.00	**0.00057**	4.48	**0.00472**
PZA	rpsA:Val82Ala	0	1	1				
PZA	None	6	88	94				
RIF	rpoB:1282del9bp	1	0	1				
RIF	rpoB:Arg552His	2	0	2	1.00	**0.00490**	4.52	**0.00929**
RIF	rpoB:Ser450Leu	126	0	126	1.00	**0.00000**	8.66	**0.00000**
RIF	rpoB:Arg827Leu	1	0	1				
RIF	rpoC:Glu1092Asp	5	2	7	0.71	**0.00012**	4.05	**0.00025**
RIF	rpoB:Asp435Tyr	2	0	2	1.00	**0.00490**	4.52	**0.00929**
RIF	rpoB:Ser441Leu	1	0	1				
RIF	rpoC:Pro481Thr	1	0	1				
RIF	rpoC:Pro739Leu	1	0	1				
RIF	rpoB:Asp435Val	9	0	9	1.00	**0.00000**	6.03	**0.00020**
RIF	rpoB:Gln432Glu	1	0	1				
RIF	rpoB:Val170Leu	1	0	1				
RIF	rpoB:Asp634Gly	1	1	2	0.50	0.11755	3.14	0.04833
RIF	rpoC:Gly442Cys	1	0	1				
RIF	rpoB:Glu812Gly	1	0	1				
RIF	rpoB:His445Gln	1	0	1				
RIF	rpoB:Leu430Pro	1	0	1				
RIF	rpoB:Lys446Arg	1	0	1				
RIF	rpoB:His445Arg	1	0	1				
RIF	rpoB:His445Asn	1	1	2	0.50	0.11755	3.14	0.04833
RIF	rpoB:His445Asp	3	0	3	1.00	**0.00048**	4.93	**0.00354**
RIF	rpoC:Gly594Glu	14	4	18	0.78	**0.00000**	4.39	**0.00000**
RIF	rpoB:His445Leu	2	0	2	1.00	**0.00490**	4.52	**0.00929**
RIF	rpoB:Leu378Arg	1	0	1				
RIF	rpoB:His445Tyr	3	0	3	1.00	**0.00048**	4.93	**0.00354**
RIF	rpoC:Ala701Val	1	0	1				
RIF	rpoB:Ile873Phe	1	0	1				
RIF	rpoC:Lys1152Gln	21	0	21	1.00	**0.00000**	6.87	**0.00002**
RIF	rpoB:Leu731Pro	31	0	31	1.00	**0.00000**	7.26	**0.00001**
RIF	rpoB:Met587Thr	1	0	1				
RIF	rpoB:Pro45Ala	1	0	1				
RIF	rpoB:Pro802Leu	1	0	1				
RIF	rpoB:Val496Ala	5	0	5	1.00	**0.00001**	5.44	**0.00098**
RIF	rpoB:Val496Met	1	0	1				
RIF	rpoB:Val534Ala	1	0	1				
RIF	rpoC:Ala492Pro	1	0	1				
RIF	rpoC:Ala521Asp	1	0	1				
RIF	rpoC:Asn698Ser	1	0	1				
RIF	rpoC:Asp57Asn	2	0	2	1.00	**0.00490**	4.52	**0.00929**
RIF	rpoC:Asp747Gly	1	0	1				
RIF	rpoC:Val483Gly	5	0	5	1.00	**0.00001**	5.44	**0.00098**
RIF	rpoC:Glu49Gln	1	0	1				
RIF	rpoC:Glu750Gly	1	0	1				
RIF	rpoC:Gly973Asp	1	0	1				
RIF	rpoC:Ile491Val	1	0	1				
RIF	rpoC:Ile707Val	1	0	1				
RIF	rpoC:Met1012Leu	1	0	1				
RIF	rpoC:Pro1040Ala	1	0	1				
RIF	rpoC:Pro1040Arg	1	0	1				
RIF	rpoC:Ser1115Leu	1	0	1				
RIF	rpoC:Trp484Gly	2	0	2	1.00	**0.00490**	4.52	**0.00929**
RIF	rpoC:Val1252Leu	1	0	1				
RIF	rpoB:Ser450Trp	1	0	1				
RIF	None	2	46	48				
STR	gid:104delC	1	2	3	0.33	0.51648	0.92	0.52719
STR	gid:Gly130Ala	1	0	1				
STR	gid:117delG	1	0	1				
STR	gid:314ins3bp	0	1	1				
STR	gid:Leu16Arg	112	39	151	0.74	**0.00012**	2.66	**0.00082**
STR	gid:353delC	0	1	1				
STR	gid:Ala140Val	0	1	1				
STR	gid:Ala167Asp	0	1	1				

STR	gid:Ala80Pro	29	7	36	0.81	**0.00014**	3.03	**0.00059**
STR	gid:Leu59Arg	1	0	1				
STR	gid:Arg21Trp	1	0	1				
STR	gid:Cys191Phe	0	1	1				
STR	gid:Glu40Lys	0	1	1				
STR	gid:Glu92Asp	17	2	19	0.89	**0.00008**	3.75	**0.00050**
STR	gid:Glu92Gln	2	0	2	1.00	0.06593	3.00	0.08886
STR	gid:Gly157Arg	0	1	1				
STR	gid:Gly164Asp	0	1	1				
STR	gid:Gly34Glu	2	0	2	1.00	0.06593	3.00	0.08886
STR	gid:Gly76Asp	0	1	1				
STR	gid:Trp45Ter	1	0	1				
STR	gid:Val66Ala	0	1	1				
STR	gid:Leu79Ser	1	0	1				
STR	gid:Ser149Arg	1	0	1				
STR	gid:Val112Gly	1	0	1				
STR	gid:Val135Gly	0	1	1				
STR	rpsL:Lys43Arg	93	1	94	0.99	**0.00000**	6.14	**0.00000**
STR	rpsL:Lys88Arg	4	0	4	1.00	**0.00824**	3.69	0.02888
STR	rrs:1076insT	21	0	21	1.00	**0.00000**	5.35	**0.00102**
STR	rrs:A807C	1	0	1				
STR	rrs:A1401G	30	4	34	0.88	**0.00001**	3.62	**0.00012**
STR	gid:Arg102Ter	1	0	1				
STR	gid:Arg154Pro	1	0	1				
STR	gid:Arg83Pro	1	0	1				
STR	rrs:A906G	1	0	1				
STR	gid:603delT	1	0	1				
STR	rrs:C1402A	1	0	1				
STR	rrs:G1484T	3	0	3	1.00	0.02198	3.40	0.04705
STR	rrs:C492T	1	4	5	0.20	1.00000	0.22	0.86969
STR	rrs:C517T	1	0	1				
STR	rrs:C774A	0	1	1				
STR	gid:Ala134Gly	0	1	1				
STR	rrs:C924T	1	1	2	0.50	0.39560	1.61	0.31822
STR	gid:Ser136Ter	1	1	2	0.50	0.39560	1.61	0.31822
STR	rrspromoter:C-69T	0	1	1				
STR	None	2	10	12				
