# Supplementary material for: Using genomics to understand the origin and dispersion of multidrug and extensively drug resistant tuberculosis in Portugal
Source: Sci Rep. 2020 Feb 13;10:2600. doi: 10.1038/s41598-020-59558-3 (PMC7018963; doi:10.1038/s41598-020-59558-3)
Supplement: Supplementary file 5 — Supplementary Information 5. [file 41598_2020_59558_MOESM5_ESM.pdf]

Supplementary Table S4 - Allelic configurations/genotypes detected across *loci* associated with drug resistance, distribution across drug susceptible and resistant isolates, positive predictive value (PPV) and statistical association with drug resistance. Statistically significant *p values* are highlighted in bold.

Drug	Genotype	No. of Isolates			PPV	*p* (Fisher Exact Test)	log OR	*p* (Z-test)
		R	S	Total				
AMK	eispromoter:C-12T	0	2	2	0.00	1.0000	2.14	0.21774
AMK	eispromoter:G-10A	2	13	15	0.13	0.1416	1.65	0.11329
AMK	rrs:1076insT,eispromoter:G-10A	6	14	20	0.30	**0.0013**	2.68	**0.00202**
AMK	rrs:1076insT,rrs:A807C,eispromoter:G-10A	0	1	1	0.00	1.0000	2.83	0.13073
AMK	rrs:A1401G	30	0	30	1.00	**0.0000**	7.62	**0.00000**
AMK	rrs:A906G	0	1	1	0.00	1.0000	2.83	0.13073
AMK	rrs:C1402A,rrs:G1484T	0	1	1	0.00	1.0000	2.83	0.13073
AMK	rrs:C492T	0	1	1	0.00	1.0000	2.83	0.13073
AMK	rrs:C517T,eispromoter:C-14T	1	0	1	1.00	0.0423	4.22	0.02440
AMK	rrs:C774A	0	1	1	0.00	1.0000	2.83	0.13073
AMK	rrs:C924T	0	2	2	0.00	1.0000	2.14	0.21774
AMK	rrs:G1484T	2	0	2	1.00	**0.0023**	4.91	**0.00466**
AMK	None	2	68	70				
CAP	idsA2promoter:A-65G,rrs:1076insT	0	2	2	0.00	1.0000	2.34	0.17767
CAP	idsA2promoter:A-65G,rrs:1076insT,rrs:A807C	0	1	1	0.00	1.0000	3.03	0.10562
CAP	idsA2promoter:A-65G,tlyA:752insTG,rrs:1076insT	17	1	18	0.94	**0.0000**	6.56	**0.00000**
CAP	idsA2promoter:C-98T	0	1	1	0.00	1.0000	3.03	0.10562
CAP	idsA2promoter:G-97A	0	1	1	0.00	1.0000	3.03	0.10562
CAP	rrs:A1401G	29	1	30	0.97	**0.0000**	7.09	**0.00000**
CAP	rrs:A906G	0	1	1	0.00	1.0000	3.03	0.10562
CAP	rrs:C1402A,rrs:G1484T	1	0	1	1.00	0.0349	4.42	0.01838
CAP	rrs:C492T	0	1	1	0.00	1.0000	3.03	0.10562
CAP	rrs:C517T	0	1	1	0.00	1.0000	3.03	0.10562
CAP	rrs:C774A	0	1	1	0.00	1.0000	3.03	0.10562
CAP	rrs:C924T	0	2	2	0.00	1.0000	2.34	0.17767
CAP	rrs:G1484T	2	0	2	1.00	**0.0016**	5.11	**0.00322**
CAP	None	2	83	85				
EMB	embA:Thr608Asn,embB:Met306Ile,embR:Leu313Arg	1	0	1	1.00	0.0500	4.04	0.03111
EMB	embA:Val961Phe,embC:Arg738Gln	0	1	1	0.00	1.0000	2.66	0.15661
EMB	embApromoter:-32delG,embB:Gln497Arg,embC:Val981Leu	1	0	1	1.00	0.0500	4.04	0.03111
EMB	embApromoter:C-11A,embApromoter:C-12A,embA:Gly582Arg,embB:Pro397Thr	1	1	2	0.50	0.0967	3.35	0.03475
EMB	embApromoter:C-11A,embApromoter:C-12A,embB:Pro397Thr	16	3	19	0.84	**0.0000**	5.02	**0.00000**
EMB	embApromoter:C-16A	1	0	1	1.00	0.0500	4.04	0.03111
EMB	embApromoter:C-16G	1	1	2	0.50	0.0967	3.35	0.03475
EMB	embApromoter:C-16G,embB:Ser538Pro	1	0	1	1.00	0.0500	4.04	0.03111
EMB	embApromoter:C-16T,embB:Met306Val,embB:Met423Thr	14	1	15	0.93	**0.0000**	5.99	**0.00000**
EMB	embB:178del3bp	0	2	2	0.00	1.0000	1.96	0.25831
EMB	embB:Asp1017Asn	0	1	1	0.00	1.0000	2.66	0.15661
EMB	embB:Asp354Ala	1	0	1	1.00	0.0500	4.04	0.03111
EMB	embB:Gln497Arg	2	1	3	0.67	**0.0093**	4.04	**0.00442**
EMB	embB:Gln497Arg,embC:Val981Leu,ubiA:Leu172Pro	0	1	1	0.00	1.0000	2.66	0.15661
EMB	embB:Gln497Lys,ubiA:Val148Ala	1	0	1	1.00	0.0500	4.04	0.03111
EMB	embB:Gly406Asp	4	1	5	0.80	**0.0001**	4.74	**0.00037**
EMB	embB:Gly406Cys	1	0	1	1.00	0.0500	4.04	0.03111
EMB	embB:Gly443Ser,embC:Ile487Thr	0	1	1	0.00	1.0000	2.66	0.15661
EMB	embB:Lys1011Thr	0	1	1	0.00	1.0000	2.66	0.15661
EMB	embB:Met306Ile	1	3	4	0.25	0.1814	2.25	0.09797
EMB	embB:Met306Ile,embB:Met306Val	1	0	1	1.00	0.0500	4.04	0.03111
EMB	embB:Met306Ile,embB:Ser297Ala	1	0	1	1.00	0.0500	4.04	0.03111
EMB	embB:Met306Ile,embC:Leu766Pro	1	0	1	1.00	0.0500	4.04	0.03111
EMB	embB:Met306Ile,embC:Val981Leu,ubiA:Ala249Ser	0	1	1	0.00	1.0000	2.66	0.15661
EMB	embB:Met306Ile,embC:Val981Leu,ubiA:Ala38Pro	0	1	1	0.00	1.0000	2.66	0.15661
EMB	embB:Met306Val	14	8	22	0.64	**0.0000**	3.91	**0.00000**
EMB	embB:Met306Val,embB:Met423Thr	13	6	19	0.68	**0.0000**	4.12	**0.00000**
EMB	embB:Met306Val,embC:Val981Leu	1	0	1	1.00	0.0500	4.04	0.03111
EMB	embB:Met306Val,ubiA:Ala38Val	1	0	1	1.00	0.0500	4.04	0.03111
EMB	embB:Met306Val,ubiA:Met180Val	1	0	1	1.00	0.0500	4.04	0.03111
EMB	embB:Met423Thr	0	1	1	0.00	1.0000	2.66	0.15661
EMB	embB:Ser297Ala	0	1	1	0.00	1.0000	2.66	0.15661
EMB	embC:Ala774Ser	0	1	1	0.00	1.0000	2.66	0.15661
EMB	embC:Arg738Gln	0	1	1	0.00	1.0000	2.66	0.15661
EMB	embC:Arg879Gly	0	1	1	0.00	1.0000	2.66	0.15661
EMB	embC:Val981Leu	0	10	10	0.00	1.0000	0.35	0.82672
EMB	Rv2820c:Leu212Val	1	2	3	0.33	0.1404	2.66	0.06142
EMB	Rv2820c:Lys114Asn	0	1	1	0.00	1.0000	2.66	0.15661
EMB	Rv2820c:Lys114Asn,embApromoter:C-16G,embB:Met306Val	1	0	1	1.00	0.0500	4.04	0.03111
EMB	Rv2820c:Lys114Asn,embApromoter:C-8A,embB:Gln445Arg	0	1	1	0.00	1.0000	2.66	0.15661
EMB	Rv2820c:Lys114Asn,embB:Asp354Ala	1	0	1	1.00	0.0500	4.04	0.03111
EMB	Rv2820c:Lys114Asn,embB:Gly406Ala	1	0	1	1.00	0.0500	4.04	0.03111
EMB	Rv2820c:Lys114Asn,embB:Leu402Val	0	1	1	0.00	1.0000	2.66	0.15661
EMB	Rv2820c:Lys114Asn,embB:Met306Ile	2	0	2	1.00	**0.0033**	4.74	**0.00640**
EMB	Rv2820c:Lys114Asn,embB:Met306Leu,ubiA:Met180Ile	1	0	1	1.00	0.0500	4.04	0.03111
EMB	Rv2820c:Lys114Asn,embB:Met306Val	2	0	2	1.00	**0.0033**	4.74	**0.00640**
EMB	Rv2820c:Lys75Thr,embC:Val981Leu	0	1	1	0.00	1.0000	2.66	0.15661
EMB	Rv3300c:Arg215Ser	0	1	1	0.00	1.0000	2.66	0.15661
EMB	Rv3300c:Asp84Gly,embB:Gly406Ala,embC:Val981Leu	0	1	1	0.00	1.0000	2.66	0.15661
EMB	Rv3300c:Asp84Gly,embB:Met306Ile,embC:Val981Leu	1	0	1	1.00	0.0500	4.04	0.03111
EMB	Rv3300c:Pro295Ala,embC:Ala774Ser	0	1	1	0.00	1.0000	2.66	0.15661
EMB	ubiA:Ala181Val	0	1	1	0.00	1.0000	2.66	0.15661
EMB	None	2	57	59				
ETH	ethA:112delT	1	0	1	1.00	0.1290	2.89	0.11540
ETH	ethA:182delG	1	0	1	1.00	0.1290	2.89	0.11540
ETH	ethA:343delT,ethA:Ser266Arg	1	0	1	1.00	0.1290	2.89	0.11540
ETH	ethA:493insGG	0	1	1	0.00	1.0000	1.50	0.41263
ETH	ethA:770delC	1	0	1	1.00	0.1290	2.89	0.11540
ETH	ethA:886delA	1	0	1	1.00	0.1290	2.89	0.11540
ETH	ethA:933del36bp	0	1	1	0.00	1.0000	1.50	0.41263
ETH	ethA:Ala185Thr	1	0	1	1.00	0.1290	2.89	0.11540
ETH	ethA:Cys403Arg	2	0	2	1.00	0.0202	3.58	0.03442
ETH	ethA:Glu400Asp	1	0	1	1.00	0.1290	2.89	0.11540
ETH	ethA:Gly450Asp	1	0	1	1.00	0.1290	2.89	0.11540
ETH	ethA:Ile221Met	0	1	1	0.00	1.0000	1.50	0.41263
ETH	ethA:Ile339Asn	0	1	1	0.00	1.0000	1.50	0.41263
ETH	ethA:Leu194Pro	1	0	1	1.00	0.1290	2.89	0.11540
ETH	ethA:Lys224Ter	1	0	1	1.00	0.1290	2.89	0.11540
ETH	ethA:Phe264Leu	0	1	1	0.00	1.0000	1.50	0.41263
ETH	ethA:Tyr147Ter	0	1	1	0.00	1.0000	1.50	0.41263
ETH	fabG1promoter:C-15T	2	1	3	0.67	0.0532	2.89	0.03456
ETH	fabG1promoter:C-15T,ethA:His281Pro	2	0	2	1.00	0.0202	3.58	0.03442

ETH	fabG1promoter:C-15T,inhA:Ile194Thr	29	0	29	1.00	**0.0000**	6.26	**0.00005**
ETH	fabG1promoter:C-15T,inhA:Ser94Ala	62	0	62	1.00	**0.0000**	7.02	**0.00001**
ETH	fabG1promoter:T-8C,ethA:Met1Leu	1	0	1	1.00	0.1290	2.89	0.11540
ETH	inhA:Ile194Thr	1	0	1	1.00	0.1290	2.89	0.11540
ETH	inhA:Ile21Val	1	0	1	1.00	0.1290	2.89	0.11540
ETH	None	3	27	30				
FQ	gyrA:Asn83Lys,gyrA:Glu21Gln,gyrA:Gly668Asp,gyrA:Ser95Thr	0	1	1	0.00	1.0000	2.74	0.14383
FQ	gyrA:Asp94Ala,gyrA:Glu21Gln,gyrA:Gly668Asp,gyrA:Ser95Thr,gyrB:Val301Leu	12	0	12	1.00	**0.0000**	6.61	**0.00004**
FQ	gyrA:Asp94Asn,gyrA:Glu21Gln,gyrA:Gly668Asp,gyrA:Ser95Thr	1	0	1	1.00	0.0462	4.13	0.02774
FQ	gyrA:Asp94Gly,gyrA:Glu21Gln,gyrA:Gly668Asp,gyrA:Ser95Thr	20	0	20	1.00	**0.0000**	7.12	**0.00001**
FQ	gyrA:Asp94Val,gyrA:Glu21Gln,gyrA:Gly668Asp,gyrA:Ser95Thr,gyrB:Val301Leu	1	0	1	1.00	0.0462	4.13	0.02774
FQ	gyrA:Glu21Gln	0	3	3	0.00	1.0000	1.64	0.33062
FQ	gyrA:Glu21Gln,gyrA:Gly247Ser,gyrA:Gly668Asp,gyrA:Ser95Thr	0	1	1	0.00	1.0000	2.74	0.14383
FQ	gyrA:Glu21Gln,gyrA:Gly247Ser,gyrA:Gly668Asp,gyrA:Ser95Thr,gyrB:Val301Leu	1	0	1	1.00	0.0462	4.13	0.02774
FQ	gyrA:Glu21Gln,gyrA:Gly668Asp,gyrA:His70Arg,gyrA:Ser95Thr	1	1	2	0.50	0.0895	3.43	0.03040
FQ	gyrA:Glu21Gln,gyrA:Gly668Asp,gyrA:Pro472Ser,gyrA:Ser95Thr	0	2	2	0.00	1.0000	2.05	0.23837
FQ	gyrA:Glu21Gln,gyrA:Gly668Asp,gyrA:Ser91Pro,gyrA:Ser95Thr	21	0	21	1.00	**0.0000**	7.17	**0.00001**
FQ	gyrA:Glu21Gln,gyrA:Gly668Asp,gyrA:Ser95Thr	2	59	61	0.03	1.0000	0.05	0.96108
FQ	gyrA:Glu21Gln,gyrA:Gly668Asp,gyrA:Ser95Thr,gyrA:Thr80Ala	0	1	1	0.00	1.0000	2.74	0.14383
FQ	gyrA:Glu21Gln,gyrA:Gly668Asp,gyrA:Ser95Thr,gyrB:Asp461His	1	0	1	1.00	0.0462	4.13	0.02774
FQ	gyrA:Glu21Gln,gyrA:Gly668Asp,gyrA:Ser95Thr,gyrB:Val301Leu	0	18	18	0.00	1.0000	-0.15	0.92571
FQ	gyrA:Glu21Gln,gyrB:Asp461His	1	0	1	1.00	0.0462	4.13	0.02774
INH	ahpC:Pro44Arg	2	1	3	0.67	0.1880	1.85	0.15921
INH	ahpCpromoter:C-52T,katG:687del6bp	1	0	1	1.00	0.2692	1.85	0.30360
INH	ahpCpromoter:G-48A	1	0	1	1.00	0.2692	1.85	0.30360
INH	ahpCpromoter:G-48A,katG:Gly494Ala	1	0	1	1.00	0.2692	1.85	0.30360
INH	ahpCpromoter:G-88A,katG:Arg463Leu,katG:Ser315Thr	2	0	2	1.00	0.0798	2.54	0.12364
INH	fabG1promoter:C-15T	4	0	4	1.00	**0.0088**	3.23	0.03970
INH	fabG1promoter:C-15T,inhA:Ile194Thr	35	0	35	1.00	**0.0000**	5.40	**0.00032**
INH	fabG1promoter:C-15T,inhA:Ser94Ala	71	0	71	1.00	**0.0000**	6.11	**0.00004**
INH	kasA:Asn400Ser	0	1	1	0.00	1.0000	0.46	0.79786
INH	kasA:Gly269Ser	1	0	1	1.00	0.2692	1.85	0.30360
INH	kasA:Gly269Ser,fabG1promoter:C-15T	1	0	1	1.00	0.2692	1.85	0.30360
INH	kasA:Gly269Ser,inhA:Gly183Ser	0	1	1	0.00	1.0000	0.46	0.79786
INH	kasA:Gly269Ser,katG:Ser315Thr	2	0	2	1.00	0.0798	2.54	0.12364
INH	kasA:Ser31Cys,katG:Arg463Leu,katG:Ser315Thr	1	0	1	1.00	0.2692	1.85	0.30360
INH	katG:1947insC	1	0	1	1.00	0.2692	1.85	0.30360
INH	katG:270insA	1	0	1	1.00	0.2692	1.85	0.30360
INH	katG:Arg463Leu	0	1	1	0.00	1.0000	0.46	0.79786
INH	katG:Arg463Leu,katG:Ser315Thr	16	0	16	1.00	**0.0000**	4.62	**0.00223**
INH	katG:Arg463Leu,katG:Ser315Thr,fabG1promoter:C-15T	1	0	1	1.00	0.2692	1.85	0.30360
INH	katG:Ser315Asn	1	0	1	1.00	0.2692	1.85	0.30360
INH	katG:Ser315Thr	29	0	29	1.00	**0.0000**	5.21	**0.00052**
INH	katG:Ser315Thr,fabG1promoter:C-15T	2	0	2	1.00	0.0798	2.54	0.12364
INH	katG:Ser315Thr,fabG1promoter:T-8C	1	0	1	1.00	0.2692	1.85	0.30360
INH	katG:Ser315Thr,inhA:Ile194Thr	1	0	1	1.00	0.2692	1.85	0.30360
INH	katG:Ser460Asn,inhA:Ile21Val	1	0	1	1.00	0.2692	1.85	0.30360
INH	katG:Thr380Ile,fabG1promoter:C-15T	1	0	1	1.00	0.2692	1.85	0.30360
INH	katGpromoter:A-6G,ahpC:Pro44Arg,fabG1promoter:C-15T	1	0	1	1.00	0.2692	1.85	0.30360
INH	None	6	19	25				
KAN	eispromoter:C-12T	0	1	1	0.00	1.0000	3.87	0.08405
KAN	eispromoter:C-14T,rrs:C517T	1	0	1	1.00	0.0204	5.26	0.01896
KAN	eispromoter:G-10A	12	0	12	1.00	**0.0000**	7.74	**0.00013**
KAN	eispromoter:G-10A,rrs:1076insT	7	3	10	0.70	**0.0000**	5.41	**0.00062**
KAN	eispromoter:G-10A,rrs:1076insT,rrs:A807C	0	1	1	0.00	1.0000	3.87	0.08405
KAN	rrs:A1401G	21	1	22	0.95	**0.0000**	7.61	**0.00001**
KAN	rrs:A906G	0	1	1	0.00	1.0000	3.87	0.08405
KAN	rrs:C1402A,rrs:G1484T	1	0	1	1.00	0.0204	5.26	0.01896
KAN	rrs:C774A	0	1	1	0.00	1.0000	3.87	0.08405
KAN	rrs:C924T	0	2	2	0.00	1.0000	3.18	0.13499
KAN	rrs:G1484T	2	0	2	1.00	**0.0008**	5.95	**0.00513**
KAN	None	0	48	48				
PZA	pncA:251insG	5	0	5	1.00	**0.0000**	4.99	**0.00122**
PZA	pncA:283insA	1	0	1	1.00	0.0737	3.38	0.05806
PZA	pncA:393insCC	2	0	2	1.00	**0.0061**	4.07	0.01284
PZA	pncA:416del3bp	2	0	2	1.00	**0.0061**	4.07	0.01284
PZA	pncA:440insCG	0	1	1	0.00	1.0000	1.99	0.26372
PZA	pncA:452del13bp	1	0	1	1.00	0.0737	3.38	0.05806
PZA	pncA:485ins10bp	1	0	1	1.00	0.0737	3.38	0.05806
PZA	pncA:Asp49Asn	1	0	1	1.00	0.0737	3.38	0.05806
PZA	pncA:Asp49Glu	0	1	1	0.00	1.0000	1.99	0.26372
PZA	pncA:Asp63Ala	3	0	3	1.00	**0.0006**	4.48	**0.00472**
PZA	pncA:Asp8Glu	1	0	1	1.00	0.0737	3.38	0.05806
PZA	pncA:Gln141Pro	1	0	1	1.00	0.0737	3.38	0.05806
PZA	pncA:Glu15Ter	1	0	1	1.00	0.0737	3.38	0.05806
PZA	pncA:Gly78Ser	1	0	1	1.00	0.0737	3.38	0.05806
PZA	pncA:Gly97Cys	1	1	2	0.50	0.1412	2.69	0.06880
PZA	pncA:Ile133Ser	1	0	1	1.00	0.0737	3.38	0.05806
PZA	pncA:Ile133Thr	2	0	2	1.00	**0.0061**	4.07	0.01284
PZA	pncA:Leu120Pro	16	0	16	1.00	**0.0000**	6.15	**0.00004**
PZA	pncA:Leu159Pro	1	0	1	1.00	0.0737	3.38	0.05806
PZA	pncA:Leu172Pro	2	0	2	1.00	**0.0061**	4.07	0.01284
PZA	pncA:Leu182Ser	1	0	1	1.00	0.0737	3.38	0.05806
PZA	pncA:Leu35Arg	1	0	1	1.00	0.0737	3.38	0.05806
PZA	pncA:Leu4Ser	1	0	1	1.00	0.0737	3.38	0.05806
PZA	pncA:Met1Thr	5	0	5	1.00	**0.0000**	4.99	**0.00122**
PZA	pncA:Phe13Ile	1	0	1	1.00	0.0737	3.38	0.05806
PZA	pncA:Phe13Leu	3	0	3	1.00	**0.0006**	4.48	**0.00472**
PZA	pncA:Pro62Leu	1	0	1	1.00	0.0737	3.38	0.05806
PZA	pncA:Pro69Leu	1	0	1	1.00	0.0737	3.38	0.05806
PZA	pncA:Thr135Pro	1	0	1	1.00	0.0737	3.38	0.05806
PZA	pncA:Thr76Ile	1	0	1	1.00	0.0737	3.38	0.05806
PZA	pncA:Thr76Pro	1	0	1	1.00	0.0737	3.38	0.05806
PZA	pncA:Tyr103Ter	1	0	1	1.00	0.0737	3.38	0.05806
PZA	pncA:Tyr41Ter	1	0	1	1.00	0.0737	3.38	0.05806
PZA	pncA:Tyr95Ter	1	0	1	1.00	0.0737	3.38	0.05806
PZA	pncA:Val125Gly	35	0	35	1.00	**0.0000**	6.93	**0.00000**
PZA	pncA:Val155Gly	1	0	1	1.00	0.0737	3.38	0.05806
PZA	pncA:Val155Leu	0	1	1	0.00	1.0000	1.99	0.26372
PZA	pncA:Val180Leu	0	1	1	0.00	1.0000	1.99	0.26372
PZA	pncApromoter:A-11C	1	0	1	1.00	0.0737	3.38	0.05806
PZA	pncApromoter:A-11G	3	0	3	1.00	**0.0006**	4.48	**0.00472**
PZA	rpsA:Val82Ala	0	1	1	0.00	1.0000	1.99	0.26372
PZA	None	6	88	94				
RIF	rpoB:1282del9bp	1	0	1	1.00	0.0612	3.83	0.04133

RIF	rpoB:Arg552His,rpoB:Ser450Leu	2	0	2	1.00	**0.0049**	4.52	**0.00929**
RIF	rpoB:Arg827Leu,rpoB:Ser450Leu,rpoC:Glu1092Asp	1	0	1	1.00	0.0612	3.83	0.04133
RIF	rpoB:Asp435Tyr	1	0	1	1.00	0.0612	3.83	0.04133
RIF	rpoB:Asp435Tyr,rpoB:Ser441Leu,rpoC:Pro481Thr,rpoC:Pro739Leu	1	0	1	1.00	0.0612	3.83	0.04133
RIF	rpoB:Asp435Val	7	0	7	1.00	**0.0000**	5.77	**0.00040**
RIF	rpoB:Asp435Val,rpoB:Gln432Glu	1	0	1	1.00	0.0612	3.83	0.04133
RIF	rpoB:Asp435Val,rpoB:Val170Leu	1	0	1	1.00	0.0612	3.83	0.04133
RIF	rpoB:Asp634Gly	0	1	1	0.00	1.0000	2.44	0.19310
RIF	rpoB:Asp634Gly,rpoB:Ser450Leu,rpoC:Gly442Cys	1	0	1	1.00	0.0612	3.83	0.04133
RIF	rpoB:Glu812Gly,rpoB:His445Gln,rpoB:Leu430Pro,rpoB:Lys446Arg	1	0	1	1.00	0.0612	3.83	0.04133
RIF	rpoB:His445Arg	1	0	1	1.00	0.0612	3.83	0.04133
RIF	rpoB:His445Asn	1	1	2	0.50	0.1176	3.14	0.04833
RIF	rpoB:His445Asp,rpoC:Gly594Glu	3	0	3	1.00	**0.0005**	4.93	**0.00354**
RIF	rpoB:His445Leu	1	0	1	1.00	0.0612	3.83	0.04133
RIF	rpoB:His445Leu,rpoB:Leu378Arg	1	0	1	1.00	0.0612	3.83	0.04133
RIF	rpoB:His445Tyr,rpoC:Ala701Val	1	0	1	1.00	0.0612	3.83	0.04133
RIF	rpoB:His445Tyr,rpoC:Gly594Glu	2	0	2	1.00	**0.0049**	4.52	**0.00929**
RIF	rpoB:Ile873Phe,rpoB:Ser450Leu,rpoC:Lys1152Gln	1	0	1	1.00	0.0612	3.83	0.04133
RIF	rpoB:Leu731Pro,rpoB:Ser450Leu	31	0	31	1.00	**0.0000**	7.26	**0.00001**
RIF	rpoB:Met587Thr,rpoB:Ser450Leu,rpoC:Lys1152Gln	1	0	1	1.00	0.0612	3.83	0.04133
RIF	rpoB:Pro45Ala,rpoB:Ser450Leu	1	0	1	1.00	0.0612	3.83	0.04133
RIF	rpoB:Pro802Leu,rpoB:Ser450Leu,rpoC:Lys1152Gln	1	0	1	1.00	0.0612	3.83	0.04133
RIF	rpoB:Ser450Leu	36	0	36	1.00	**0.0000**	7.41	**0.00000**
RIF	rpoB:Ser450Leu,rpoB:Val496Ala	5	0	5	1.00	**0.0000**	5.44	**0.00098**
RIF	rpoB:Ser450Leu,rpoB:Val496Met,rpoC:Gly594Glu	1	0	1	1.00	0.0612	3.83	0.04133
RIF	rpoB:Ser450Leu,rpoB:Val534Ala,rpoC:Gly594Glu	1	0	1	1.00	0.0612	3.83	0.04133
RIF	rpoB:Ser450Leu,rpoC:Ala492Pro,rpoC:Glu1092Asp	1	0	1	1.00	0.0612	3.83	0.04133
RIF	rpoB:Ser450Leu,rpoC:Ala521Asp	1	0	1	1.00	0.0612	3.83	0.04133
RIF	rpoB:Ser450Leu,rpoC:Asn698Ser,rpoC:Glu1092Asp	1	0	1	1.00	0.0612	3.83	0.04133
RIF	rpoB:Ser450Leu,rpoC:Asp57Asn	2	0	2	1.00	**0.0049**	4.52	**0.00929**
RIF	rpoB:Ser450Leu,rpoC:Asp747Gly,rpoC:Gly594Glu	1	0	1	1.00	0.0612	3.83	0.04133
RIF	rpoB:Ser450Leu,rpoC:Glu1092Asp	1	0	1	1.00	0.0612	3.83	0.04133
RIF	rpoB:Ser450Leu,rpoC:Glu1092Asp,rpoC:Val483Gly	1	0	1	1.00	0.0612	3.83	0.04133
RIF	rpoB:Ser450Leu,rpoC:Glu49Gln,rpoC:Lys1152Gln	1	0	1	1.00	0.0612	3.83	0.04133
RIF	rpoB:Ser450Leu,rpoC:Glu750Gly,rpoC:Gly594Glu	1	0	1	1.00	0.0612	3.83	0.04133
RIF	rpoB:Ser450Leu,rpoC:Gly594Glu	4	0	4	1.00	**0.0001**	5.21	**0.00173**
RIF	rpoB:Ser450Leu,rpoC:Gly973Asp,rpoC:Lys1152Gln	1	0	1	1.00	0.0612	3.83	0.04133
RIF	rpoB:Ser450Leu,rpoC:Ile491Val	1	0	1	1.00	0.0612	3.83	0.04133
RIF	rpoB:Ser450Leu,rpoC:Ile707Val	1	0	1	1.00	0.0612	3.83	0.04133
RIF	rpoB:Ser450Leu,rpoC:Lys1152Gln	16	0	16	1.00	**0.0000**	6.60	**0.00004**
RIF	rpoB:Ser450Leu,rpoC:Met1012Leu	1	0	1	1.00	0.0612	3.83	0.04133
RIF	rpoB:Ser450Leu,rpoC:Pro1040Ala	1	0	1	1.00	0.0612	3.83	0.04133
RIF	rpoB:Ser450Leu,rpoC:Pro1040Arg	1	0	1	1.00	0.0612	3.83	0.04133
RIF	rpoB:Ser450Leu,rpoC:Ser1115Leu	1	0	1	1.00	0.0612	3.83	0.04133
RIF	rpoB:Ser450Leu,rpoC:Trp484Gly	2	0	2	1.00	**0.0049**	4.52	**0.00929**
RIF	rpoB:Ser450Leu,rpoC:Val1252Leu	1	0	1	1.00	0.0612	3.83	0.04133
RIF	rpoB:Ser450Leu,rpoC:Val483Gly	4	0	4	1.00	**0.0001**	5.21	**0.00173**
RIF	rpoB:Ser450Trp	1	0	1	1.00	0.0612	3.83	0.04133
RIF	rpoC:Glu1092Asp	0	2	2	0.00	1.0000	1.75	0.31429
RIF	rpoC:Gly594Glu	1	4	5	0.20	0.2617	1.75	0.18880
RIF	None	2	46	48				
STR	gid:104delC	0	2	2	0.00	1.0000	0.22	0.89915
STR	gid:104delC,gid:Gly130Ala	1	0	1	1.00	0.2308	2.30	0.22491
STR	gid:117delG	1	0	1	1.00	0.2308	2.30	0.22491
STR	gid:314ins3bp,gid:Leu16Arg	0	1	1	0.00	1.0000	0.92	0.62915
STR	gid:353delC	0	1	1	0.00	1.0000	0.92	0.62915
STR	gid:Ala140Val	0	1	1	0.00	1.0000	0.92	0.62915
STR	gid:Ala167Asp,gid:Leu16Arg	0	1	1	0.00	1.0000	0.92	0.62915
STR	gid:Ala80Pro,gid:Leu16Arg	5	4	9	0.56	0.1588	1.83	0.07371
STR	gid:Ala80Pro,gid:Leu16Arg,gid:Leu59Arg	1	0	1	1.00	0.2308	2.30	0.22491
STR	gid:Arg21Trp,gid:Leu16Arg	1	0	1	1.00	0.2308	2.30	0.22491
STR	gid:Cys191Phe,gid:Leu16Arg	0	1	1	0.00	1.0000	0.92	0.62915
STR	gid:Glu40Lys,gid:Leu16Arg	0	1	1	0.00	1.0000	0.92	0.62915
STR	gid:Glu92Asp	0	2	2	0.00	1.0000	0.22	0.89915
STR	gid:Glu92Gln	2	0	2	1.00	0.0659	3.00	0.08886
STR	gid:Gly157Arg,gid:Leu16Arg	0	1	1	0.00	1.0000	0.92	0.62915
STR	gid:Gly164Asp	0	1	1	0.00	1.0000	0.92	0.62915
STR	gid:Gly34Glu,gid:Leu16Arg	2	0	2	1.00	0.0659	3.00	0.08886
STR	gid:Gly76Asp	0	1	1	0.00	1.0000	0.92	0.62915
STR	gid:Leu16Arg	2	19	21	0.10	0.6100	-0.64	0.54994
STR	gid:Leu16Arg,gid:Trp45Ter	1	0	1	1.00	0.2308	2.30	0.22491
STR	gid:Leu16Arg,gid:Val66Ala	0	1	1	0.00	1.0000	0.92	0.62915
STR	gid:Leu79Ser	1	0	1	1.00	0.2308	2.30	0.22491
STR	gid:Ser149Arg	1	0	1	1.00	0.2308	2.30	0.22491
STR	gid:Val112Gly	1	0	1	1.00	0.2308	2.30	0.22491
STR	gid:Val135Gly	0	1	1	0.00	1.0000	0.92	0.62915
STR	rpsL:Lys43Arg	5	0	5	1.00	**0.0034**	3.91	0.01939
STR	rpsL:Lys43Arg,gid:Glu92Asp	14	0	14	1.00	**0.0000**	4.94	**0.00250**
STR	rpsL:Lys43Arg,gid:Leu16Arg	47	0	47	1.00	**0.0000**	6.15	**0.00014**
STR	rpsL:Lys88Arg	2	0	2	1.00	0.0659	3.00	0.08886
STR	rpsL:Lys88Arg,gid:Glu92Asp	1	0	1	1.00	0.2308	2.30	0.22491
STR	rrs:1076insT,rpsL:Lys43Arg,gid:Leu16Arg	20	0	20	1.00	**0.0000**	5.30	**0.00114**
STR	rrs:1076insT,rrs:A807C,rpsL:Lys43Arg,gid:Leu16Arg	1	0	1	1.00	0.2308	2.30	0.22491
STR	rrs:A1401G,gid:Ala80Pro,gid:Leu16Arg	23	3	26	0.88	**0.0000**	3.65	**0.00022**
STR	rrs:A1401G,gid:Arg102Ter,gid:Leu16Arg	1	0	1	1.00	0.2308	2.30	0.22491
STR	rrs:A1401G,gid:Arg154Pro,gid:Leu16Arg	1	0	1	1.00	0.2308	2.30	0.22491
STR	rrs:A1401G,gid:Arg83Pro,gid:Leu16Arg	1	0	1	1.00	0.2308	2.30	0.22491
STR	rrs:A1401G,gid:Leu16Arg	0	1	1	0.00	1.0000	0.92	0.62915
STR	rrs:A1401G,rpsL:Lys43Arg,gid:Glu92Asp	1	0	1	1.00	0.2308	2.30	0.22491
STR	rrs:A1401G,rpsL:Lys43Arg,gid:Leu16Arg	2	0	2	1.00	0.0659	3.00	0.08886
STR	rrs:A1401G,rpsL:Lys88Arg	1	0	1	1.00	0.2308	2.30	0.22491
STR	rrs:A906G,gid:603delT	1	0	1	1.00	0.2308	2.30	0.22491
STR	rrs:C1402A,rrs:G1484T,rpsL:Lys43Arg,gid:Leu16Arg	1	0	1	1.00	0.2308	2.30	0.22491
STR	rrs:C492T,gid:Leu16Arg	1	4	5	0.20	1.0000	0.22	0.86969
STR	rrs:C517T,gid:Glu92Asp	1	0	1	1.00	0.2308	2.30	0.22491
STR	rrs:C774A,gid:Ala134Gly,gid:Leu16Arg	0	1	1	0.00	1.0000	0.92	0.62915
STR	rrs:C924T,gid:Ser136Ter	1	1	2	0.50	0.3956	1.61	0.31822
STR	rrs:G1484T,rpsL:Lys43Arg,gid:Leu16Arg	2	0	2	1.00	0.0659	3.00	0.08886
STR	rrspromoter:C-69T,rpsL:Lys43Arg,gid:Leu16Arg	0	1	1	0.00	1.0000	0.92	0.62915
STR	None	2	10	12				
