# Supplementary material for: Using genomics to understand the origin and dispersion of multidrug and extensively drug resistant tuberculosis in Portugal
Source: Sci Rep. 2020 Feb 13;10:2600. doi: 10.1038/s41598-020-59558-3 (PMC7018963; doi:10.1038/s41598-020-59558-3)
Supplement: Supplementary file 6 — Supplementary Information 6. [file 41598_2020_59558_MOESM6_ESM.pdf]

Supplementary Table S5 - Distribution of candidate barcode SNPs per sub-lineage and country of origin

SNP[a]	No. of ENA Accessions	Sub-lineages	Country of Origin
208287	61	lineage2.2.1 (2), lineage2.2.2 (45), lineage3 (3), lineage4.3.4.2 (6), lineage4.6.1 (2), lineage4.8 (2), undetermined (1)	Brazil (2),Canada (11),China (1),Korea (5),Mali (2),Netherlands (2),Russia (1),Switzerland (3),Thailand (1),UK (4),United Kingdom (5),Unknown (18),USA (1),Vietnam (5)
405812	17	lineage3.1.2 (2), lineage4.3.4.2 (6), lineage4.4.1.2 (2), lineage4.6.1 (2), lineage4.6.2.1 (5)	Brazil (2),Mali (2),Switzerland (2),United Kingdom (7),Unknown (4)
740561	24	lineage2.2.2 (3), lineage4.1.2.1 (10), lineage4.2.1 (1), lineage4.3.2.1 (1), lineage4.3.3 (1), lineage4.3.4.2 (6), undetermined (2)	Brazil (2),Mali (2),Moldova (1),South Africa (2),Switzerland (2),Thailand (3),United Kingdom (2),Unknown (10)
1057412	6	lineage4.3.4.2 (6)	Brazil (2),Mali (2),Switzerland (2)
1153137	6	lineage4.3.4.2 (6)	Brazil (2),Mali (2),Switzerland (2)
1706439	6	lineage4.3.4.2 (6)	Brazil (2),Mali (2),Switzerland (2)
1885422	6	lineage4.3.4.2 (6)	Brazil (2),Mali (2),Switzerland (2)
2123086	153	lineage1.1.1.1 (3), lineage3 (4), lineage4.1 (1), lineage4.1.1.1 (8), lineage4.1.2.1 (1), lineage4.3.2.1 (121), lineage4.3.4 (2), lineage4.3.4.2 (7), lineage4.4.1.1 (3), lineage4.6.1.1 (1), lineage4.8 (2)	Australia (1),Brazil (4),Cote d'Ivoire (1),Indonesia (1),Mali (2),Saudi Arabia (1),South Africa (124),Switzerland (2),United Kingdom (2),Unknown (12),Vietnam (3)
2255294	6	lineage4.3.4.2 (6)	Brazil (2),Mali (2),Switzerland (2)
2264189	6	lineage4.3.4.2 (6)	Brazil (2),Mali (2),Switzerland (2)
3520172	1	lineage4.3.4.2 (1)	United Kingdom (1)
3544710	3584	lineage4.1 (126), lineage4.1.1 (58), lineage4.1.1.1 (369), lineage4.1.1.2 (83), lineage4.1.1.3 (589), lineage4.1.2 (810), lineage4.1.2.1 (1539), lineage4.3 (1), lineage4.3.4.2 (6), lineage4.4 (1), undetermined (2)	Albania (3),Argentina (254),Australia (4),Azerbaijan (1),Bangladesh (5),Botswana (2),Brazil (103),Bulgaria (1),Canada (485),Colombia (7),Cote d'Ivoire (11),Denmark (6),Djibouti (5),Georgia (13),Germany (3),Guatemala (4),Guinea (1),India (5),Indonesia (53),Ireland (1),Israel (2),Italy (15),Malawi (125),Mali (11),Moldova (13),Morocco (3),Mozambique (6),Nepal (1),Netherlands (33),Nigeria (8),Pakistan (1),Papua New Guinea (1),Peru (194),Romania (26),Russia (74),South Africa (544),South Korea (1),Switzerland (8),Thailand (8),Tunisia (8),Uganda (10),United Kingdom (336),Unknown (1108),USA (17),Vietnam (58),Zimbabwe (6)
4034894	11	lineage1.1.3 (1), lineage4.3.4.2 (6), lineage4.4.1.2 (3), undetermined (1)	Brazil (2),Mali (2),Switzerland (2),United Kingdom (2),Unknown (3)
4104091	8	lineage4.2 (2), lineage4.3.4.2 (6)	Brazil (2),Mali (2),Switzerland (2),United Kingdom (2)

[a] SNP Position relative to the genome position of *M. tuberculosis* H37Rv (GenBank Accession NC000962.3)
